# Sidechain structure–activity relationships of cyclobutane-based small molecule αvβ3 antagonists†

**DOI:** 10.1039/d4md00306c

**Published:** 2024-09-13

**Authors:** Adam Throup, Manar Saleh Zraikat, Andrew Gordon, Shohreh Jafarinejad Soumehsaraei, Kathrin D. Haase, Laurence H. Patterson, Patricia A. Cooper, Katherine Hanlon, Paul M. Loadman, Mark Sutherland, Steven D. Shnyder, Helen M. Sheldrake

**Affiliations:** a Institute of Cancer Therapeutics, University of Bradford Bradford BD7 1DP UK h.sheldrake@bradford.ac.uk

## Abstract

The integrin family of cell surface extracellular matrix binding proteins are key to several physiological processes involved in tissue development, as well as cancer proliferation and dissemination. They are therefore attractive targets for drug discovery with cancer and non-cancer applications. We have developed a new integrin antagonist chemotype incorporating a functionalised cyclobutane ring as the central scaffold in an arginine–glycine–aspartic acid mimetic structure. Here, we report the synthesis of cyclobutanecarboxylic acids and cyclobutylamines with tetrahydronaphthyridine and aminopyridine arginine mimetic sidechains and masked carboxylic acid aspartic acid mimetic sidechains of varying length. Effective αvβ3 antagonists and new aspartic acid mimetics were identified in cell-based adhesion and invasion assays. A lead compound selected based on *in vitro* activity (IC_50_ < 1 μM), stability (*t*_1/2_ > 80 minutes) and synthetic tractability was well-tolerated *in vivo*. These results show the promise of this synthetic approach for developing αvβ3 antagonists and provide a firm foundation to progress into advanced preclinical evaluation prior to progression towards the clinic. Additionally, they highlight the use of functionalised cyclobutanes as metabolically stable core structures and a straightforward and robust method for their synthesis. This important contribution to the medicinal chemists' toolbox paves the way for increased use of cyclobutanes in drug discovery.

## Abstract 2

Cyclobutanes provide a versatile scaffold for the synthesis of small molecule integrin antagonists with good metabolic stability and *in vivo* tolerability.

## Graphical abstract

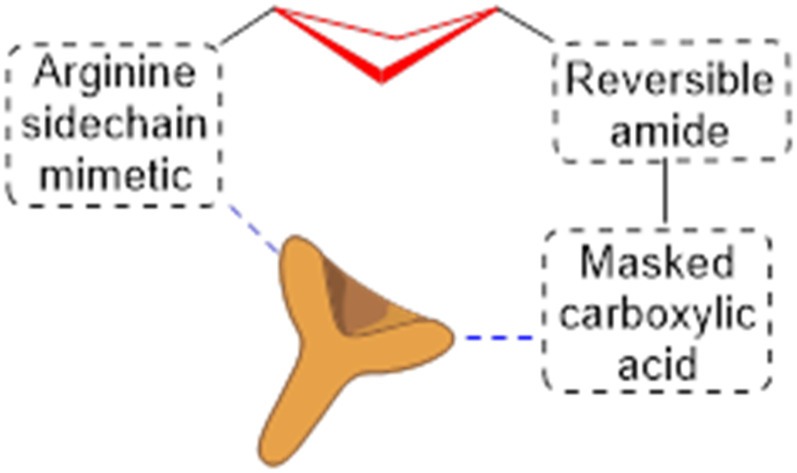

## Introduction

Metastasis is the major cause of death and disability from many cancers and remains a major challenge for cancer therapy.^1^ The identification of effective strategies to prevent metastatic dissemination and development of secondary tumours is an important goal for drug development.

Integrin receptors, particularly the subfamily which recognise the Arg–Gly–Asp (RGD) sequence in extracellular matrix proteins, have been shown to play key roles in the development of blood and lymph-borne metastases and therefore are attractive targets for drug development. Integrin αvβ3, the prototypical member of the RGD-recognising subfamily, supports tumour angiogenesis^2^ and is required for invasion and migration;^3^ its expression is increased during these processes,^4–7^ allowing tumour cells to interact with platelets and adhere to the metastatic site.^4,8^ Metastasis to bone is promoted by migration towards RGD-containing proteins in the bone microenvironment and indirectly enhancing osteoclast-mediated bone resorption.^9^ Lung metastasis is promoted through αvβ3-expressing cells interacting with fibronectin and fibrin to invade clots in the lung vasculature.^10^ Knockdown or pharmacological inhibition of αvβ3 reduces *in vivo* tumour growth and metastasis in a number of tumour types,^11–17^ however translation of these results to the clinic has so far been unsuccessful, often due to poor pharmacokinetics rendering it difficult to reach therapeutic concentrations for adequate time periods which can cause paradoxical effects.^18,19^

Antagonists of αvβ3 are usually competitive antagonists of the RGD recognition sequence comprising arginine and aspartic acid sidechain mimetics presented in the optimum binding conformation by a core scaffold. We hypothesised that a cyclobutane core would provide an appropriate skeleton to direct arginine and aspartate mimetic sidechains in the correct orientation for high αvβ3 affinity while potentially improving the antagonist's pharmacokinetic properties and *in vivo* effectiveness.^20^ Cyclobutanes are underutilised in medicinal chemistry due to the limited range of methods for the synthesis of functionalised rings^21^ particularly in the core of molecules.^20^ This work demonstrates a simple method that allows the synthesis of a wide range of novel RGD-mimetics and the first study of *in vivo* tolerability of the cyclobutane RGD-mimetic chemotype.

## Results and discussion

### Antagonist synthesis

#### Tetrahydronaphthyridines

**Fig. 1:**
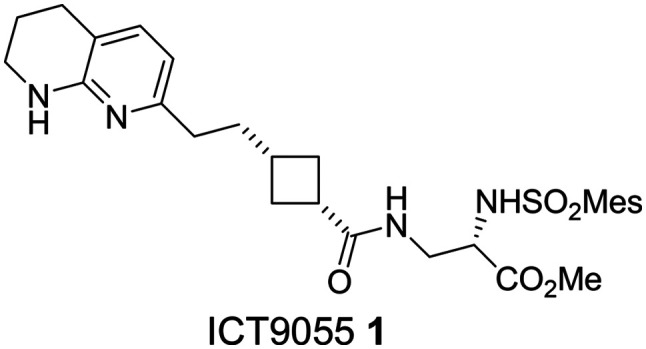
Structure of the cyclobutane-based αvβ3 integrin antagonist ICT9055.

**Scheme 1:**
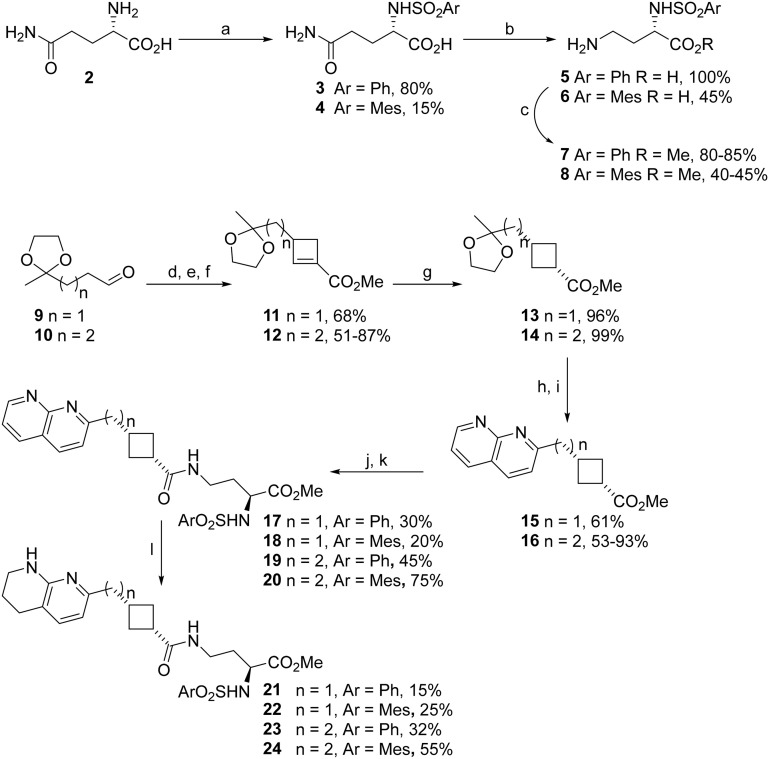
Synthesis of naphthyridine- and tetrahydronapthyridine–cyclobutane RGD mimetics with varying linker length. a. ArSO_2_Cl, NaOH, dioxane/H_2_O, RT, 4.5 h; b. Br_2_, NaOH, H_2_O, 0–90 °C, 1 h; c. SOCl_2_, MeOH, RT, 26 h; d. Et_2_NH, K_2_CO_3_, then methyl acrylate, MeCN, RT, 3 days; e. MeI, MeCN, RT, 24 h; f. DBU, CHCl_3_, reflux, 24 h; g. H_2_, Pd/C, EtOAc, RT, 23 h; h. HCl, MeOH, RT, 1.3 h; i. 2-amino-3-pyridinecarboxaldehyde, pyrrolidine, H_2_SO_4_, MeOH, RT, 24 h; j. aq. HCl, RT, 18–24 h; k. EDCI, HOBt, DIPEA, 7 or 8, DMF, RT, 24 h; l. H_2_, PtO_2_, MeOH, RT, 23 h.

ICT9055 1 (Fig. 1) was designed as a RGD-mimetic using the cyclobutane as core (Gly-mimetic) controlling the orientation of 2 sidechains bearing tetrahydronaphthyridine (Arg-mimetic) and ester (Asp-mimetic) sidechains.^22^ It is considerably more active than analogues with shorter carbon sidechains, establishing the minimum length required for anti-αvβ3 activity.^22^ Further investigation of the linker was planned to examine longer compounds and different orientation of hydrogen bonding groups within the molecule. Cyclobutanes 15 and 16 were synthesised as previously described: aldehydes 9 and 10 (obtained respectively from ethyl levulinate and ethyl-4-acetylbutyrate) were subjected to a one pot cyclisation–quaternisation–elimination reaction to give cyclobutenes 11 and 12 (Scheme 1).^22^ Reduction of the cyclobutene, deprotection of the sidechain ketone and Friedlander synthesis gave the cyclobutanes 15 and 16 bearing an Arg mimetic napththyridine sidechain. 15 and 16 were coupled with novel Asp sidechain mimetics 7 and 8 obtained from glutamine (Scheme 1) to afford naphthyridine RGD mimetics 17–20. The naphthyridine ring was selectively reduced with hydrogen/platinum oxide to give tetrahydronaphthyridine (THN) RGD mimetics 21–24.

#### Tetrahydronaphthyridine reversed amides

**Scheme 2:**
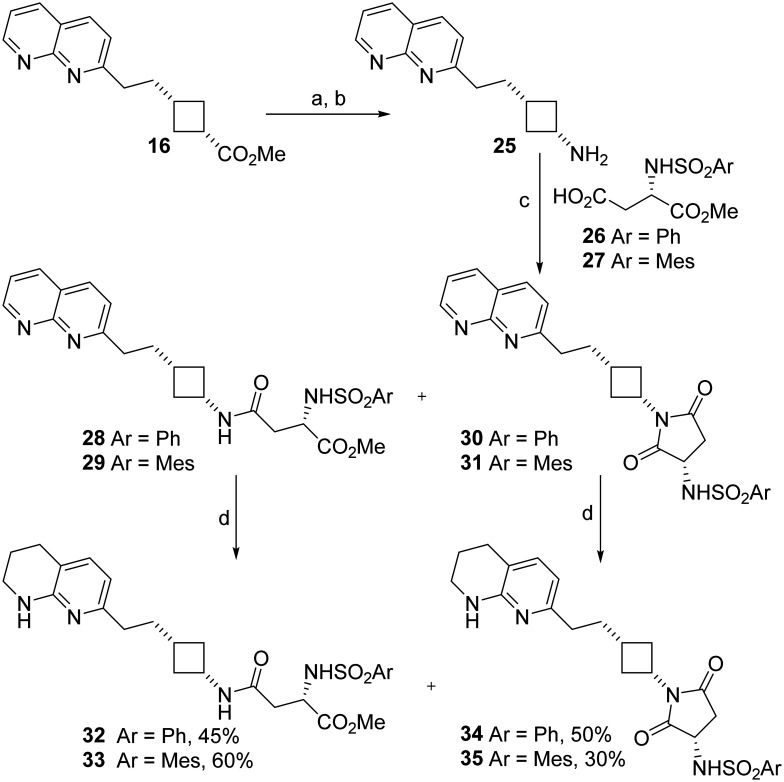
Synthesis of naphthyridine- and tetrahydronapthyridine–cyclobutane RGD mimetics containing a reversed amide cyclobutylamine skeleton. Spontaneous cyclisation occurred during the coupling reaction to yield a novel Asp mimetic. a. aq. HCl, RT, 24 h; b. NaN_3_, H_2_SO_4_, CHCl_3_, 45 °C, 5 h, 100% over 2 steps; c. EDCI, HOBt, DIPEA, 26 or 27, solvent, RT, see Table 2 for yields; d. H_2_, PtO_2_, MeOH, RT, 23 h.

Incorporation of a reversed amide may improve stability towards metabolism by proteases by further changing the structure from a RGD peptide sequence. Cyclobutylamine 25 was synthesised in quantitative yield from cyclobutane 16*via* hydrolysis of the ester followed by Curtius rearrangement (Scheme 2). The original method used for the rearrangement^23^ involving reaction at 45 °C followed by stirring at room temperature overnight, basification of the reaction mixture and extraction with diethyl ether, gave low (19–41%) yields, which were attributed to degradation of the product during the reaction and poor recovery of the water soluble cyclobutylamine. Changing the extraction solvent to DCM improved recovery and the long reaction time proved completely unnecessary: reaction at 45 °C followed by immediate workup gave 25 in quantitative yield (Table 1).

**Table 1:** Optimisation of the Curtius reaction

Entry	16 (mmol) : H_2_SO_4_ (ml) : CHCl_3_ (ml)	Time at 45 °C	Time at RT	Workup	Yield 25
1	1 : 1 : 2	5.5 h	17 h	Adjust pH to 13, extract with Et_2_O	19%
2	1 : 2 : 4	5 h	17 h	Evaporate, adjust pH to 14, extract with Et_2_O	41%
3	1 : 2 : 4	5 h	45 min	Evaporate, adjust pH to 14, extract with DCM	71%
4	1 : 2 : 4	5 h	0	Adjust pH to 14, extract with DCM	100%

Cyclobutylamine 25 was coupled with aspartate-derived α-sulphonamides 26 and 27 to yield the expected compounds 28 and 29 along with succinimide side products 30 and 31. We had previously observed that the solvent used was key to success of amide coupling reactions of cyclobutanecarboxylic acids: coupling with β-alanine gave high yields in DCM but sulfonamide-substituted acids only coupled in DMF.^22^ Comparing the two solvents here, better overall yields were obtained with DMF. Use of DCM reduced the amount of cyclised 30 and 31 formed but did not correspondingly increase recovery of 28 and 29 (Table 2).

**Table 2:** Effect of solvent on product distribution in coupling reactions of 25

Entry	Acid	Solvent	Yield amide	Yield succinimide	Ratio
1	26	DMF	34% (28)	22% (30)	1.55 : 1
2	26	DCM	44% (28)	18% (30)	2.44 : 1
3	27	DMF	44% (29)	46% (31)	0.96 : 1
4	27	DCM	43% (29)	10% (31)	4.3 : 1

The naphthyridine RGD mimetics 28–31 were hydrogenated to give the target tetrahydronaphthyridine RGD mimetics 32–35. All compounds 28–35 were tested for anti-integrin activity (Table 3); as amides 28, 29, 32, 33 and cyclic derivatives 30, 31, 34, 35 could interconvert under biological conditions as well as during synthesis we wished to screen the succinimides for toxicity and determine whether their presence could cause loss of integrin inhibition.

**Table 3:** Anti-αvβ3 activity

Compound	Adhesion % inhibition^a^@50 μM	Adhesion % inhibition^a^@5 μM	Adhesion IC_50_^a^/μM	Invasion % inhibition^b^@10 μM
17	13 ± 25	—	—	23.3 ± 6.2
18	—	—	—	17.4 ± 3.7
19	c	54.0 ± 15.0	—	—
20	c	43.0 ± 6.0	11.5 ± 5.3	55.7 ± 1.2
21	c	4.0 ± 9.0	29.4 ± 2.1	49.5 ± 8.8
22	c	83.0 ± 19.0	—	22.9 ± 5.7
23	c	57.0 ± 13.0	—	—
24	c	74.0 ± 10.0	—	35.0 ± 9.0
28	42.4 ± 16.3	—	—	28.5 ± 4.4
29	56.6 ± 15.3	—	—	70.4 ± 1.4
30	—	39.9 ± 23.8	—	22.6 ± 4.0
31	60.9 ± 23.8	13.8 ± 9.7	49.5 ± 29.8	—
32	—	45.4 ± 13.7	—	41.6 ± 4.7
33	—	89.5 ± 8.7	1.2 ± 0.9	33.7 ± 7.4
34	73.9 ± 24.6	60.2 ± 14.8	3.28 ± 0.01	69.9 ± 1.8
35	—	—d	1.8 ± 1.0	—
37	c	50 ± 17	13.6 ± 8.3	—
38	43 ± 31	—	—	—
39	c	71 ± 16	—	—
40	c	67 ± 16	—	—
53	—	85.0 ± 1.4	1.8 ± 0.4	62.9 ± 1.5
54	—	84.9 ± 11.2	0.6 ± 0.7	48.6 ± 1.0
1 ICT9055	—	98.4 ± 1.9	0.34 ± 0.33	60.2 ± 0.2
cRGDfV	—	61.5 ± 15.1	2.1 ± 0.8	41.2 ± 4.6

a Inhibition of αvβ3-mediated SK-Mel-2 melanoma cell adhesion to fibronectin by compounds at the stated concentration.

b Inhibition of U87-MG spheroid Matrigel invasion.

c >75% inhibition in an initial trial experiment; not tested further at this concentration.

d 45.6 ± 20.9 inhibition at 0.5 μM. Data are given as the mean ± SD of a minimum of 3 independent experiments. — Not tested.

**Scheme 3:**
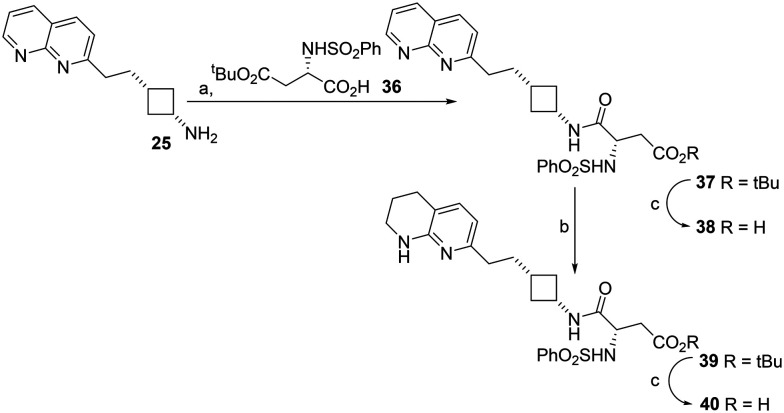
Synthesis of naphthyridine- and tetrahydronapthyridine–cyclobutane RGD mimetics containing a β-phenylsulfonamide D mimetic. a. 36, EDCI, HOBt, DIPEA, DCM, RT, 24 h, 50%; b. H_2_, PtO_2_, MeOH, RT, 23 h, 90%; c. TFA, DCM, RT, 24 h, 100%.

The potential interconversion of amides and cyclic succinimides could result in positional isomers with different locations of the sulfonamide substituent depending on the direction of ring opening. Asp mimetics β-substituted by aromatic rings have been reported previously^24^ however β-phenylsulfonamide Asp mimetics are unknown. The desired Asp mimetic 36 was readily available by reaction of commercially available aspartic acid 4-*tert*-butyl ester with phenylsulfonyl chloride. Unfortunately, we were unable to prepare the mesityl analogue of 36, presumably due to the greater steric bulk of the *tert*-butyl ester and mesityl groups. β-Phenylsulfonamide RGD mimetic analogues of 28 and 32 were synthesised by coupling cyclobutylamine 25 with Asp mimetic 36 to give naphthyridine RGD mimetic 37. The coupling reaction was carried out in DCM and no cyclised side-product was observed. Amide 37 was hydrogenated to give tetrahydronaphthyridine RGD mimetic 39 (Scheme 3). The respective carboxylic acid analogues 38 and 40 were also synthesised by treatment of the ester with TFA.

#### Aminopyridines

An aminopyridine Arg mimetic is a smaller, more flexible analogue of the tetrahydronaphthyridine group and avoids the potential issue of regioselectivity the naphthyridine reduction step. While our THN-containing molecules had acceptable (>95%) purity by LC–MS, an aminopyridine analogue is more desirable for reliable future scale-up.^25^

**Scheme 4:**
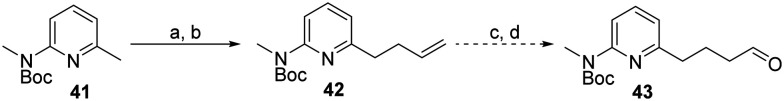
Originally proposed route to the aminopyridine sidechain. a. *n*BuLi, iPr_2_NH, THF, −78 °C; b. allyl bromide, −78 °C to RT, 0–70%; c. BH_3_·THF, then NaOH, H_2_O_2_; d. PCC.

The only active integrin antagonist cyclobutanes reported to date are all THN-based.^22,26^ We proposed that an aminopyridine sidechain could be obtained *via* the one-pot cyclobutene formation using aldehyde 43, which could be obtained by oxidising the corresponding primary alcohol, a known compound reported to be synthesised by alkylation of protected aminopyridine 41 with allyl bromide followed by hydroboration/oxidation (Scheme 4).^27^ In our hands, the route proved unreliable and did not consistently give acceptable yields despite meticulous attention to reaction conditions in the alkylation step.

**Scheme 5:**
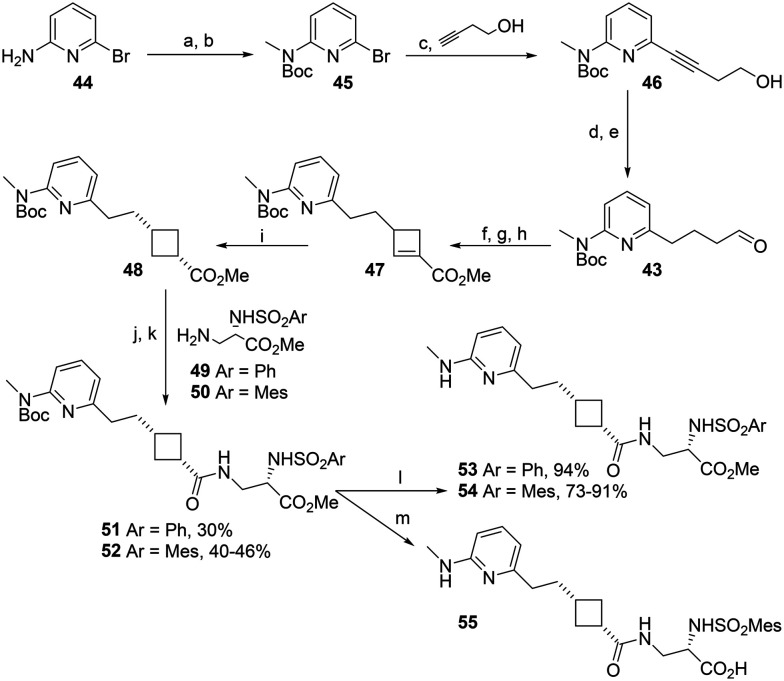
Synthesis of aminopyridine–cyclobutane RGD mimetics. a. Boc_2_O, DCM, RT, 24 h, 78%; b. NaH, DMF, 0 °C to RT, 1.25 h then MeI, RT, 18 h, 85%; c Pd(PPh_3_)_2_Cl_2_, CuI, Et_2_NH, 70 °C, 22 h, 94–97%; d. H_2_, PtO_2_, EtOH, RT, 22.5 h, 100%; e. TEMPO, BAIB, DCM, RT, 24 h, 50–77%; f. Et_2_NH, K_2_CO_3_, MeCN, RT, 3 h, then methyl acrylate, RT, 69 h; g. MeI, MeCN, RT, 24 h; h. DBU, CHCl_3_, reflux, 24 h, 63–75% over 3 steps; i. H_2_, Pd/C, EtOAc, RT, 22 h, 90–92%; j. NaOH, MeOH, reflux, 5 h; k. 49 or 50, EDCI, HOBt, DIPEA, DMF, RT, 18 h; l. TFA, DCM, RT, 23 h; m. 6 M HCl, RT, 20 h, 100%.

The alkylation of 41 became a barrier to obtaining enough material to continue the synthesis of aminopyridine-sidechain cyclobutanes. Therefore, a cross-coupling-based alternative route to 43 was developed (Scheme 5). Gratifyingly, palladium-catalysed cross-coupling of bromoaminopyridine 45 with but-3-*yn*-1-ol was consistently high yielding; initial reaction using 0.02 equivalents (eq.) Pd and 0.01 eq. Cu catalysts gave 76% yield and increasing the amount of CuI to 0.02 eq. reliably gave yields of 94–97% on a gram scale. Hydrogenation gave the protected pyridine aldehyde 43 which was readily converted to cyclobutane 48 and coupled with Asp mimetics 49/50. Deprotection with TFA gave the target antagonists 53 and 54 in good yield.

#### *In vitro* studies

Compounds were screened for non-specific cytotoxicity using the MTT assay before use in anti-αvβ3 functional assays. Unlike normal cells, cancer cell survival is often independent of αvβ3, and checking for cytotoxicity ensures inhibition of adhesion and invasion are truly from αvβ3 inhibition rather than cell death. The majority proved to be less toxic than 1 and control RGD mimetic peptide cycloRGDfV in αvβ3-expressing U87-MG glioblastoma and SK-Mel-2 melanoma cells (ESI† Table S1). In general, aminopyridines 53 and 54 were more cytotoxic than most THN-containing mimetics.

Initial screening for anti-αvβ3 activity was carried out using our previously established^22^ adhesion assay measuring the effect of compounds on αvβ3-mediated Sk-Mel-2 melanoma cells binding to immobilised fibronectin (Table 3). In general, naphthyridine compounds were considerably less active than their tetrahydronaphthyridine counterparts (*e.g.*20 < 24, 28 < 32, 31 < 35), and mesitylsulfonamides were more active than the phenylsulfonamides (*e.g.*21 < 22, 23 < 24, 34 < 35).

Increasing the length of the antagonist by switching the Asp mimetic from 2,3-diaminopropionate to 2,4-diaminobutanoate or extending the carbon chain between Arg mimetic and cyclobutane resulted in a decrease in anti-adhesive activity in some (24 < 1) but not all (23 < 21; 17 < 19; 22 ≈ 24) cases, in contrast to the total loss of activity observed by Kessler on substitution of glycine to β-alanine or aspartate to glutamate.^28^ As the saturated sidechains are relatively flexible the longer molecules may be able to bend into an appropriate configuration to fit the αvβ3 binding site. Reversing the amide (33*vs.*1) also had little effect on activity.

The carboxylic acid and hydrophobic sidechain in the Asp mimetic have been shown to be very important for β3 binding.^29^ The hydrophobic sidechain normally contains *S* α-sulfonamides or aromatic groups in the β position (for a review of peptidomimetic and exosite binding structures functional groups see ref. 30). β-Sulfonamides are practically unprecedented in integrin antagonists,^31^ and have not been investigated in the context of β3 integrin antagonism. A terminal carboxylic acid or an ester which is hydrolysed to reveal one in biological systems is generally considered mandatory for integrin binding. Unexpectedly, the succinimide side-products of coupling Asp mimetics with cyclobutylamine 25 had very similar (31*vs.*29, 35*vs.*33), or greater anti-adhesive activity (30*vs.*28, 34*vs.*32) than the original target reversed amides. We hypothesised that this activity resulted from ring-opening under the assay conditions to release an active carboxylic acid. As the ring-opening was unlikely to be regioselective, we compared β-sulfonamides 37–40 to the α-phenylsulfonamides (28, 32) and succinimides (30, 34). The β-sulfonamides showed very similar activity to the succinimides and α-sulfonamides when tested as free carboxylic acids, but surprisingly the t-butyl esters (37, 39) were also active and equally (39*vs.*40) or more (37*vs.*38) potent than the corresponding free acids (38, 40). Esters have been used as prodrugs to improve bioavailability of αIIbβ3 antagonists (for a review of examples see ref. 32) and ethyl esters have also been speculated to act as full β3 antagonists by binding to the inactive conformation of αIIbβ3 in a binding pocket created by the absence of a metal ion from the MIDAS.^33^ Given the steric bulk of the *t*-butyl group is significantly greater than the ethyl group, and that *t*-butylesterase enzymes have been reported,^34^ it is more likely that 37 and 39 are hydrolysed to 38 and 40 when exposed to SK-Mel-2 cells, and differences in potency are related to lipophilicity and solubility differences.

Methylaminopyridines 53 and 54 were both highly effective inhibitors of αvβ3-mediated cell adhesion, essentially equipotent with the most active THN antagonists 1, 33 and 35. By affording an active phenylsulfonamide (53 RMM 474), the methylaminopyridine Arg mimetic improves compliance with Lipinski's rules as well as synthetic tractability.

Selected compounds were further investigated for effects on αvβ3-mediated cell invasion to model a key step in tumour progression and metastasis. U87-MG glioblastoma cells; a well-known αvβ3-expressing cell line model,^35,36^ were used in a 3-D spheroid invasion model.^37^ Overall, inhibition of invasion was lower than inhibition of adhesion, with most compounds showing low to moderate inhibition at 10 μM. The most active anti-invasive compounds were the methylaminopyridines 53 and 54, THNs 1 and 34, and naphthyridine 29 which was significantly more effective at preventing invasion than adhesion. The 3-D spheroid invasion assay is superior to standard monolayer transwell chamber invasion assays as it more closely represents the cell–cell interactions and architecture are present in a solid tumour. The observed reduced activity for invasion compared to adhesion assays may therefore reflect the extra complexity of the 3D model requiring more compound to penetrate into the ‘tumour’ mass to have an effect.

The stability of the most active compounds 1 and 54 was investigated in mouse liver homogenates to give an initial indication whether they were likely to have a long enough half-life to give meaningful target exposure *in vivo*. The methyl esters in 1 and 54 were cleaved to the corresponding free acids with half-lives of 5.4 minutes and 3.6 minutes respectively (ESI†). This was expected as small esters are frequently used to mask carboxylic acids with rapid ester hydrolysis releasing the active carboxylic acid in biological systems. These free acids were highly stable (half-life >80 minutes), supporting the conjecture that cyclobutanes can provide good metabolic stability in drug molecules.^20,21^

**Fig. 2:**
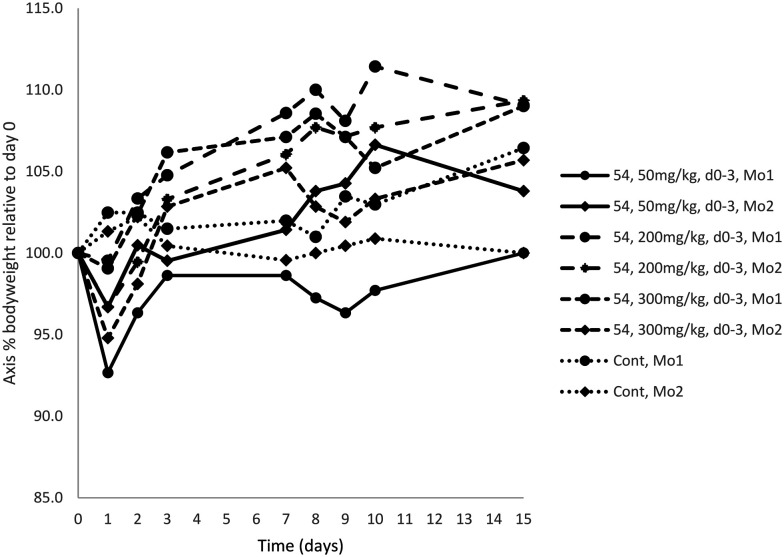
Compound 54 demonstrates negligible toxicity at its maximum soluble dose. Compound 54 was administered intraperitoneally three dose levels, up to its maximum soluble dose of 300 mg kg^−1^ per day, for 4 consecutive days to female C57BL/6 immunocompetent mice, and mice were observed daily, and bodyweight measured frequently. No deleterious effects were observed, and bodyweight remained within normal levels (*i.e.* a bodyweight loss of 15% over 2 days compared to starting bodyweight is considered toxic). Mo = mouse; Cont = solvent control.

Given compound 54's effectiveness at functional inhibition of αvβ3, its rapid conversion to a stable carboxylic acid, and our preference for developing an aminopyridine-based RGD mimetic over a THN-based one, 54 was therefore selected for *in vivo* dose tolerability studies in an immunocompetent C57BL/6 mouse model (Fig. 2).

Compound 54 was found to be non-toxic at all doses tested up to the maximum soluble dose of 300 mg kg^−1^. This is consistent with observations that other RGD-mimetic integrin antagonists are well-tolerated in clinical trials.^38,39^

Integrin ligand binding usually involves a conformational change in the protein from a low affinity to a high affinity active conformation. While our work was in progress, the structural features of small molecule integrin antagonists which stabilise the low affinity *vs.* high affinity conformation have recently been established; a hydrogen-bonding functional group, commonly a nitrogen atom β to the terminal carboxylic acid, is required to stabilise key water molecules in the integrin's ligand binding site.^40^ The cyclobutane-based antagonists described here contain a range of aspartate mimetics with different orientations of functional groups capable of hydrogen bonding. Importantly, none of the compounds described here showed any integrin-activating properties in cell-based assays: they did not promote adhesion or invasion at any concentration tested. Coupling of different aspartate mimetics with cyclobutylamine 25 could also afford new antagonists which stabilise the integrin's closed conformation.

## Conclusion

This work demonstrates the utility of cyclobutanes as a scaffold for pharmacologically active and metabolically stable molecules, using αvβ3 as an example target. The synthetic route used (Schemes 1, 2 and 5), which forms the central ring using a regiospecific thermal cyclisation reaction is an important contribution to the medicinal chemists' toolbox. By improving synthetic tractability,^21^ it paves the way for increased use of cyclobutanes in drug discovery. The vast majority of previously reported RGD-mimetics use an aryl or heterocyclic core as a Gly-mimetic spacer. There are very few cycloalkane-based integrin antagonists: specifically, 2–3 cyclohexane-based αIIbβ3 antagonists have been reported^41,42^ but there has been no systematic investigation of cyclohexane-based antagonists of other integrins or of smaller rings. Although the differences in assays used between studies prevent direct comparison it is clear from our results that cyclobutanes are a synthetically accessible skeleton for active integrin antagonists. Further studies are required to investigate the effect of ring size and orientation of sidechains on anti-integrin activity and selectivity and to determine whether the predicted pharmacokinetic advantages of small rings are true in practice.

**Fig. 3:**
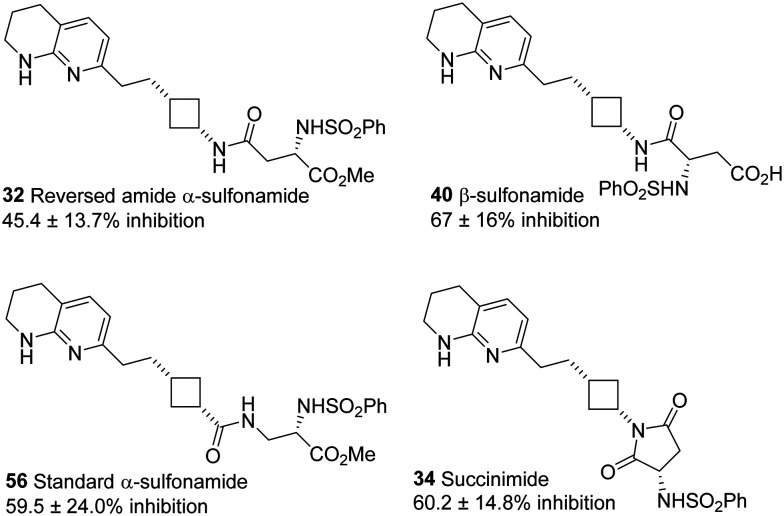
Summary of the effect of 5 μM novel Asp mimetic sidechains on αvβ3-mediated adhesion of Sk-Mel-2 cells.

Exploration of various Asp mimetic sidechains has provided the first demonstration that the β-sulfonamide sidechain can be used as an Asp mimetic, and the entirely unexpected discovery that succinimides could be used to prodrug this group. Novel RGD mimics 34 (succinimide) and 40 (β-sulfonamide) are essentially equipotent with each other and the traditional α-sulfonamide analogues 32 (this work) and 56 (ref. 22) (previous work) (Fig. 3). Further work is ongoing to explore the factors controlling the cyclisation of 32/40 to give 34 and the corresponding ring-opening.

Moving beyond THN-based antagonists, we have developed a robust gram-scale route to the aminopyridine–cyclobutane antagonist 54 which has good anti-αvβ3 activity in several melanoma cell line models. The cyclobutane structure is stable to metabolism in *ex vivo* liver homogenates and well-tolerated *in vivo*. This provides a promising starting point for the development of cyclobutane-based integrin antagonists as therapeutic agents for integrin-mediated diseases with unmet clinical need.

## Experimental methods

### General

Chemical reagents and anhydrous solvents were obtained from Sigma-Aldrich, Alfa Aesar or Fluorochem and used without further purification. All other solvents were supplied by VWR.

### Synthetic methods and characterisation data

For compounds 3–55 are reported in the ESI.†

### Biological characterisation

Cancer cell lines were obtained from ATCC (SK-Mel-2) or ECACC (U87-MG) and cultured in RPMI-1640 culture medium containing 10% foetal bovine serum (FBS), sodium pyruvate (1 mM) and l-glutamine (2 mM). All are human in origin. The αvβ3 adhesion assay was performed as previously described.^22,43^

### Invasion assay

Spheroids were formed from U87-MG cells (1000 cells per 30 μl hanging drop) according to the method of Nowicki and then embedded in collagen as follows.^37^ Collagen I (1400 μL of 3 mg mL^−1^) was added to a 1 mL Eppendorf tube on ice, supplemented with 0.2 mL of complete RPMI, and adjusted to pH 7.4 with 1 N NaOH. The collagen mixture was pipetted into each chamber of an 8-chamber cover glass and incubated at 37 °C for 45 minutes. Spheroids from hanging drops were transferred to each chamber of the cover glass, the medium allowed to evaporate, and then a 200 μL layer of collagen gel matrix was added to each chamber and incubated at 37 °C for 45 minutes. 200 μL of RPMI containing test compounds was added to each chamber and incubated at 37 °C, 5% CO_2_ for 7 days. The spheroids were observed frequently using inverted light microscopy at 10× objective lens, and images were captured and analysed using ImageJ.

### Metabolic stability

Compound metabolism in liver (*ex vivo*) was studied using (1 : 3) tissue homogenates of mouse liver. Mouse livers, supplied by the Institute of Cancer Therapeutics (ICT), under Home Office license guidelines (see below) were homogenized in phosphate-buffered saline. Liver homogenates were spiked with compound of interest (10 μM final concentration) and incubated at 37 °C. Reaction aliquots were removed over a 120 minute period, proteins precipitated using acetonitrile (1 : 3), and compound and metabolites analyzed by liquid chromatography-mass spectrometry (LC–MS) at the appropriate SIR (single ion recording) channel.

Detection was performed on a Waters Alliance system using a photodiode array detector, and a Waters Micromass ZQ quadrupole electrospray mass spectrometer connected in series. Compound and metabolites were separated on a RPB reversed-phase high-performance liquid chromatography column (HiChrom) using a mobile phase of acetonitrile/water/0.1% formic acid, with a gradient from 20% to 70% acetonitrile over 30 minutes at 1.1 mL min^−1^.

Any metabolic intermediates were detected as singularly charged ions and identified by mass spectrometry.

### *In vivo* studies

#### Ethics statement

Animal work was carried out in the UK, where nationwide licensing for animal work is in place. All animal procedures were performed in accordance with UK National Cancer Research Institute Guidelines for the Welfare of Animals^44^ under a UK Home Office Project License (40/3670, protocol 10), granted by the UK Home Office. All work was approved by the local University of Bradford Animal Welfare and Ethics Review Board.

Female adult C57BL/6 mice were used (Envigo, Blackthorn, UK). Mice all came from the same delivery and were randomly assigned to cages. They were kept in cages housed in isolation cabinets in an air-conditioned room with regular alternating cycles of light and darkness. They received Teklad 2018 diet (Envigo, Blackthorn, UK) and water *ad libitum*.

For evaluation of the safety of compound 54*in vivo*, the compound was dissolved in 15% DMSO/arachis oil, and then administered intraperitoneally as a single dose on four consecutive days, with the first day designated day 0, with 2 mice being treated at each dose level. The control was solvent administered intraperitoneally on the same schedule. Extensive experience^45–47^ has demonstrated that the safety of a particular dose can be determined using 2 animals per dose level, with 3 dose levels usually sufficient to determine the optimum dose, thus keeping the number of animals used to a minimum. Mice were observed daily for signs of deleterious effects, and bodyweight measured frequently.

## Abbreviations

Ar: Aryl
BAIB: Bis(acetoxy)iodobenzene
Boc: *tert*-Butyloxycarbonyl
DBU: 1,8-Diazabicyclo(5.4.0)undec-7-ene
DCM: Dichloromethane
DIPEA: Di-isopropylethylamine
DMF: Dimethylformamide
Ph: Phenyl
EDCI: 1-Ethyl-3-(3-dimethylaminopropyl)carbodiimide
HOBt: Hydroxybenzotriazole
Mes: Mesityl(2,4,6-trimethylphenyl)
RT: Room temperature
*t*Bu: *tert*-Butyl
TEMPO: 2,2,6,6-Tetramethylpiperidine 1-oxyl
TFA: Trifluoroacetic acid
THN: Tetrahydronaphthyridine
TLC: Thin layer chromatography

## Data availability

The data supporting this article have been included as part of the ESI.†

## Author contributions

Conceptualization: HMS, LHP. Methodology: HMS, MS, SDS, PML. Investigation: AT, KDH, MSZ, AG, SJS, PAC, KH, MS, HMS. Writing – original draft: AT, HMS. Writing – review & editing: HMS, SDS, PML, MS, AG, KDH. Supervision: HMS, PML, MS, SDS. Funding acquisition: HMS, SDS, MS, LHP.

## Conflicts of interest

AT, MS, LHP, SDS and HMS are inventors on UK patent application No. 2301024.2 related to this work.

## Supplementary Material

MD-OLF-D4MD00306C-s001

MD-OLF-D4MD00306C-s002

MD-OLF-D4MD00306C-s003
