# Supplementary material for: Sidechain structure–activity relationships of cyclobutane-based small molecule αvβ3 antagonists
Source: RSC Med Chem. 2024 Sep 13;15(10):3616–24. doi: 10.1039/d4md00306c (PMC11393731; doi:10.1039/d4md00306c)
Supplement: MD-015-D4MD00306C-s001 [file MD-015-D4MD00306C-s001.pdf]

Supplementary Information (SI) for RSC Medicinal Chemistry.
This journal is © The Royal Society of Chemistry 2024

Abbreviations: Ar Aryl; BAIB Bis(acetoxy)iodobenzene; Boc tert-butyloxycarbonyl; DBU 1,8-Diazabicyclo(5.4.0)undec-7-ene; DCM dichloromethane; DIPEA Di-isopropylethylamine; DMF Dimethylformamide; Ph Phenyl; EDCI 1-Ethyl-3-(3-dimethylaminopropyl)carbodiimide; HOBt Hydroxybenzotriazole; Mes Mesityl (2,4,6-trimethylphenyl); RT Room temperature; tBu tert-butyl; TEMPO 2,2,6,6-Tetramethylpiperidine 1-oxyl; TFA Trifluoroacetic acid; THN tetrahydronaphthyridine; TLC Thin layer chromatography.

General: Unless otherwise stated, reactions were carried out in anhydrous solvent and were not air sensitive. Petroleum ether (PE) refers to the fraction boiling between 60 and 80 °C. Flash chromatography was carried out on silica gel (Merck 9385 Kieselgel 60 (230-400 ASTM) (VWR) or Davisil 60A, 40-63μm (Fisher Scientific)). Analytical TLC was carried out on 0.25 mm thick aluminium plates precoated with Merck Kieselgel F_{254} silica gel (VWR) and visualised by UV and aqueous alkaline potassium permanganate solution. Preparative TLC was carried out on Analtech silica plates with UV245 indicator (Sigma-Aldrich). NMR spectra were recorded on a Bruker DPX400 spectrometer. Chemical shifts are reported in parts per million (δ, ppm). ^{1}H NMR chemical shifts are reported relative to residual proton signals of the solvent. Multiplets are indicated as s, singlet; d, doublet; t, triplet; q, quartet; qn, quintet; dd, double doublet; m, multiplet; br, broad, etc. Coupling constants (*J*) are expressed in Hertz (Hz). ^{13}C NMR chemical shifts are reported relative to the signal of the solvent. Wherever possible, original unedited images of spectra are provided (Supporting information file S2). Images showing integrals were produced using MestRe-C 2.3a. Mass spectra were carried out on a Micromass Quattro Ultima spectrometer in the electron impact (EI), chemical ionisation (AP) or electrospray (ES) mode as stated. Melting points were determined using a Gallenkamp melting point apparatus and are uncorrected. Purity was determined by LCMS analysis performed on a Waters e2695 Separation module using a HICHROM column (3.5RPB, 15cm

× 2.1mm), with a flow rate of 0.25 mL/min and mobile phase of water:MeOH:formic acid (5:5:0.1 at t = 0 mins,→1:9:0.1 at t = 7 mins, →5:5:0.1 at t = 13 mins), eluting compounds were analysed by Waters 2998 PDA Detector (*uv* spectroscopy 210-400 nm) and QDA Detector (mass spectrometry). Optical rotations were carried out on a Perkin Elmer polarimeter, model 341 and are reported in units of $°10^{-1}cm^2g^{-1}$. The concentration *c* is expressed in $g/0.1dm^3$.

(Phenylsulfonyl)-L-glutamine [Compound 3]: A solution of benzenesulfonyl chloride (4.08 mL, 5.65 g, 31.97 mmol) in dioxane (25 mL), and a solution of NaOH (1.81 g, 45.16 mmol) in H_2O (25 mL) were added to a stirred solution of (*S*)-glutamine **2** (5.83 g, 39.96 mmol) and NaOH (1.81 g, 45.16 mmol) in dioxane:H_2O (1:1, 50 mL) and stirred for 4 hour 45 minutes. The dioxane was removed *in vacuo* and the aqueous solution was extracted with EtOAc (2 × 30 mL). The aqueous layer was then acidified to pH 1 with concentrated HCl and cooled to 0 °C for 20 minutes, the resulting white precipitate was collected *via* filtration and dried in a desiccator to yield **3** as white crystals (7.16 g, 25.03 mmol, 78% yield); mp 175-177 °C (lit[1] 172-174 °C); $[\alpha]_D$ +0.187 (c 0.97, MeOH); ^1H NMR (400 MHz, DMSO-d_6) δ 8.17 (1H, d, *J* = 8.7 Hz, NH), 7.75-7.78 (2H, m, Ph), 7.64-7.59 (1H, tt, *J* = 7.1, 1.5 Hz, Ph), 7.53-7.57 (2H, m, Ph), 7.27 (1H, s, N*H*H'), 6.76 (1H, s, NH*H*'), 3.71 (1H, td, *J* = 8.7, 6.1 Hz, C*H*), 2.07 (2H, t, *J* = 7.7 Hz, H_2NCOCH_2), 1.89-1.77 (1H, m, CHC*H*H'), 1.72-1.58 (1H, m, CHCH*H*').

(Mesitylsulfonyl)-L-glutamine [Compound 4]: A solution of 2-mesitylenesulfonyl chloride (3.5 g, 16 mmol) in dioxane (25 mL), and a solution of NaOH (904 mg, 22.6 mmol) in H_2O (25 mL) were added to a stirred solution of (*S*)-glutamine **2** (2.92 g, 20.0 mmol) and NaOH (904 mg, 22.6 mmol) in dioxane:H_2O (1:1, 50 mL) and stirred for 4.5 hours. The dioxane was removed *in vacuo* and the aqueous solution was extracted with EtOAc (2 × 30 mL). The

aqueous layer was then acidified to pH 1 with concentrated aqueous HCl and cooled to 0 °C for 30 minutes. The solution was concentrated to ~15 mL and the resulting white precipitate was collected *via* filtration and dried in a desiccator to yield **4** as white crystals (713 mg, 2.17 mmol, 14%); mp 156-159 °C; $[\alpha]_D$ +0.400 (c 0.05, MeOH); ^{1}H NMR (400 MHz, DMSO-d_6) δ 8.01 (1H, d, *J* = 9.6 Hz, N*H*), 7.25 (1H, s, N*H*), 6.98 (2H, s, Mes-H3+H5), 6.76 (1H, s, N*H*), 3.59 (1H, dt, *J* = 9.1, 5.2 Hz, C*H*), 2.54 (6H, s, Mes-CH_3), 2.24 (3H, s, Mes-CH_3), 2.12-2.05 (2H, m, H_2NOCCH_2), 1.89-1.78 (1H, m, C*H*H'CH), 1.73-1.62 (1H, m, CH*H'*CH); ^{13}C NMR (101 MHz, DMSO-d_6) δ 173.2 (C), 172.7 (C), 141.2 (C), 138.4 (C), 134.6 (C), 131.4 (CH), 54.7 (CH), 30.8 (CH_2), 27.8 (CH_2), 22.6 (CH_3), 20.4 (CH_3); *m/z* (AP+) 312.1 ([M-NH_2]+, 100%), 329.1 ([M+H]+, 20%); HRMS Found ([M+H]+) 329.1167. $C_{14}H_{21}O_5N_2S$ req. 329.1166.

(*S*)-4-Amino-2-(phenylsulfonamido)butanoic acid [Compound 5]: Bromine (1.51 mL, 4.71 g, 29.46 mmol) was added to a stirred solution of NaOH (7.27 g, 181.7 mmol) in H_2O (20 mL) at 0 °C and stirred for 5 minutes. A solution of **3** (7.02 g, 24.55 mmol) and NaOH (2.06 g, 51.6 mmol) in H_2O (20 mL) was added and the resulting solution stirred for a further 20 minutes. The reaction mixture was then heated to 90 °C for 35 minutes. The reaction mixture was then cooled to 0 °C and carefully neutralised by dropwise addition of concentrated aqueous HCl and left in ice for 2 hours, the resultant white precipitate was collected *via* filtration to yield **5** as a white solid (6.28 g, 24.34 mmol, 99%); $[\alpha]_D$ -0.034 (c 0.82, DMSO); ^{1}H NMR (400 MHz, D_2O) δ 7.80 (2H, d, *J* = 7.7 Hz, Ph), 7.63 (1H, t, *J* = 7.7 Hz, Ph), 7.53 (2H, t, *J* = 7.7 Hz, Ph), 4.02 (1H, dd, *J* = 9.5, 4.6 Hz, C*H*), 2.99-3.05 (2H, m NCH_2), 2.06-2.15 (1H, m, C*H*H'CH), 1.86-1.95 (1H, m, CH*H'*CH); ^{13}C NMR (101 MHz, D_2O) δ 173.2 (C), 137.9 (C), 133.7 (CH), 129.4 (CH), 126.7 (CH), 53.3 (CH), 36.1 (CH_2), 29.6 (CH_2); *m/z* (ES+) 259.1 ([M+H]+, 100%); HRMS Found ([M+H]+) 259.0750. $C_{10}H_{15}O_4N_2S$ req. 259.0747.

(*S*)-4-Amino-2-(2,4,6-trimethylphenylsulfonamido)butanoic acid [Compound 6]: Bromine (0.13 mL, 401 mg, 2.51 mmol) was added to a stirred solution of NaOH (619 mg, 15.47 mmol) in H_2O (3 mL) at 0 °C and stirred for 5 minutes. A solution of **4** (684 mg, 2.09 mmol) and NaOH (176 mg, 4.39 mmol) in H_2O (3 mL) was added and the resulting solution stirred for a further 20 minutes. The reaction mixture was then heated to 90 °C for 35 minutes. The reaction mixture was then cooled to 0 °C and carefully neutralised by dropwise addition of concentrated aqueous HCl and left in ice for 20 minutes. The resultant white precipitate was collected *via* filtration to yield **6** as a white solid (287 mg, 0.96 mmol, 46%); $[\alpha]_D$ +0.24 (c 0.25, MeOH); ^{1}H NMR (400 MHz, D_2O) δ 6.96 (2H, s, Mes-H3+H5), 3.76 (1H, dd, *J* = 10.1, 4.8 Hz, C*H*), 2.83-2.98 (2H, m, NCH_2), 2.40 (6H, s, Mes-CH_3), 2.11 (3H, s, Mes-CH_3), 2.03-1.94 (1H, m, C*H*H'CH), 1.88-1.78 (1H, m, CH*H'*CH); ^{13}C NMR (101 MHz, D_2O) δ 170.4 (C), 144.3 (C), 139.4 (C), 131.9 (CH), 131.5 (C), 52.9 (CH), 36.1 (CH_2), 29.4 (CH_2), 22.0 (CH_3), 20.0 (CH_3); *m/z* (ES+) 301.1 ([M+H]+, 100%); HRMS Found ([M+H]+) 301.1212. $C_{13}H_{21}O_4N_2S$ req. 301.1217.

Methyl (*S*)-4-amino-2-(phenylsulfonamido)butanoate [Compound 7]: Thionyl chloride (0.19 mL, 2.73 mmol) was added to a stirred solution of (*S*)-4-amino-2-(phenylsulfonamido)butanoic acid **5** (640 mg, 2.48 mmol) in MeOH (25 mL) and the reaction mixture stirred for 26 hours at RT. The reaction mixture was concentrated *in vacuo* and the residue partitioned between DCM (30 mL) and saturated aqueous sodium bicarbonate solution (20 mL). The aqueous layer was further extracted with DCM (3 × 30 mL) and the combined organic layers dried ($MgSO_4$) and concentrated *in vacuo* to yield **7** as a clear oil (543 mg, 2.12 mmol, 86 % yield); ^{1}H NMR (400 MHz, $CDCl_3$) δ 7.97-7.67 (2H, m, Ph), 7.58-7.48 (3H, m, Ph), 4.10 (1H, dd, *J* = 8.1, 5.1 Hz, C*H*), 3.49 (3H, s, OCH_3), 2.91-2.78 (2H, m, NCH_2), 1.88-

1.71 (2H, m, CH_2CH); ^{13}C NMR (101 MHz, $CDCl_3$) δ 173.8 (C), 133.0 (C), 129.2 (CH), 129.0 (CH), 127.3 (CH), 53.4 (CH), 50.8 (CH_3), 39.1 (CH_2), 30.5 (CH_2); *m/z* (AP+) 241.2 ([M-OMe]+, 100%), 273.2 ([M+H]+, 90%); HRMS Found ([M+H]+) 273.0901. $C_{11}H_{17}O_4N_2S$ req. 273.0904.

Methyl (*S*)-4-amino-2-(2,4,6-trimethylphenylsulfonamido)butanoate [Compound 8]: Thionyl chloride (0.07 mL, 119 mg, 1.0 mmol) was added to a stirred solution of (*S*)-4-amino-2-(2,4,6-trimethylphenylsulfonamido)butanoic acid **6** (272 mg, 0.91 mmol) in MeOH (9 mL) and the reaction mixture stirred for 27 hours. The reaction mixture was concentrated *in vacuo*, partitioned between DCM (20 mL) and saturated aqueous sodium bicarbonate (20 mL). The aqueous layer was further extracted with DCM (20 mL) and the combined organic layers were dried ($MgSO_4$) and concentrated *in vacuo* to yield **8** as a clear oil (131 mg, 0.42 mmol, 46%); $[\alpha]_D$ -0.614 (c 0.44, $CHCl_3$); ^{1}H NMR (400 MHz, $CDCl_3$) δ 6.93 (2H, s, Mes-H3+H5), 4.01 (1H, dd, J = 7.1, 5.0 Hz, C*H*), 3.46 (3H, s, OCH_3), 2.85-2.80 (2H, m, NCH_2), 2.63 (6H, s, Mes-CH_3), 2.28 (3H, s, Mes-CH_3), 1.87-1.71 (2H, m, CH_2CH); ^{13}C NMR (101 MHz, $CDCl_3$) δ 172.4 (C), 142.3 (C), 139.2 (C), 133.6 (C), 131.9 (CH), 54.1 (CH), 52.3 (CH_3), 38.1 (CH_2), 35.1 (CH_2), 23.0 (CH_3), 21.0 (CH_3); *m/z* (ES+) 304.9 (100%), 315.1([M+H]+, 10%); HRMS Found ([M+H]+) 315.1377. $C_{14}H_{23}O_4N_2S$ req. 315.1373.

General procedure for coupling reactions.

Methyl (*S*)-4-((1*s,3*R**)-3-((1,8-naphthyridin-2-yl)methyl)cyclobutane-1-carboxamido)-2-(phenylsulfonamido)butanoate [Compound 17]:** Methyl (1*s**,3*s**)-3-((1,8-naphthyridin-2-yl)methyl)cyclobutane-1-carboxylate **15** (synthesised as previously described[2]) (82 mg, 0.32 mmol) was dissolved in 6 M aqueous HCl (2 mL) and stirred at RT for 18 hours. The reaction mixture was concentrated *in vacuo* to give the crude acid. The acid was dissolved in DMF (10

mL) and methyl (*S*)-4-amino-2-(phenylsulfonamido)butanoate **7** (82 mg, 0.32 mmol), EDCI (258 mg, 1.16 mmol), HOBt (150 mg, 1.16 mmol) and DIPEA (0.34 mL, mg, 1.95 mmol) were added and the reaction mixture stirred at RT under a blanket of N_2 for 16 hours. The reaction mixture was diluted with H_2O (10 mL), extracted with DCM (3 × 10 mL) and the combined organic layers washed with saturated ammonium chloride solution (2 × 10 mL), dried ($MgSO_4$) and concentrated *in vacuo*. The crude residue was purified *via* PTLC (95:5, DCM:MeOH) to yield **17** as a clear oil (50 mg, 0.10 mmol, 31% yield); R_f 0.32 (95:5, DCM:MeOH); $[\alpha]_D$ +0.12 (c 0.78, $CHCl_3$); IR ν_{max}/cm^{-1} 3337.2 (NH), 2930.5 (CH), 1701.1 (C=O ester), 1640.6 (C=O amide); ^1H NMR (400 MHz, $CDCl_3$) δ 9.11 (1H, dd, *J* = 4.2, 1.7 Hz, NapthH7), 8.18 (dd, 1H, *J* = 8.2, 1.7 Hz, NapthH5), 8.11 (1H, d, *J* = 8.2 Hz, NapthH4), 7.85 (2H, d, *J* = 7.4 Hz, Ph), 7.59 (1H, t, *J* = 7.4 Hz, Ph), 7.52 (2H, t, *J* = 7.4 Hz, Ph), 7.47 (1H, dd, *J* = 8.2, 4.2 Hz, NapthH6), 7.37 (1H, d, *J* = 8.2 Hz, NapthH3), 6.18 (1H, t, *J* = 5.7 Hz, N*H*), 5.76 (1H, d, *J* = 8.8 Hz, N*H*), 3.99-3.89 (1H, m, C*H*), 3.66-3.70 (1H, m, H1), 3.47 (3H, s, OCH_3), 3.34-3.23 (1H, m, H3), 3.18 (2H, d, *J* = 7.4 Hz, NapthCH_2), 3.01-2.82 (2H, m, CONHCH_2), 2.44-2.32 (2H, m, H2+H4), 2.22-2.05 (3H, m, H2'+H4'+C*H*H'CH), 1.70-1.80 (1H, m, CH*H'*CH); ^{13}C NMR (101 MHz, $CDCl_3$) δ 175.1 (C), 171.9 (C), 164.9 (C), 156.0 (C), 153.3 (CH), 139.3 (C), 137.0 (CH), 136.8 (CH), 133.0 (CH), 129.2 (CH), 127.2 (CH), 122.9 (CH), 121.5 (CH), 121.1 (C), 53.5 (CH), 52.6 (CH_3), 45.5 (CH_2), 36.6 (CH), 35.3 (CH_2), 32.6 (CH_2), 31.6 (CH_2), 31.5 (CH_2), 31.1 (CH); *m/z* (AP+) 125.0 (100%), 497.2 ([M+H]+, 15%); HRMS found ([M+H]+) 497.1852 $C_{25}H_{29}O_5N_4S$ req. 497.1853.

Methyl (*S*)-4-((1*s,3*R**)-3-((1,8-naphthyridin-2-yl)methyl)cyclobutane-1-carboxamido)-2-(2,4,6-trimethylphenylsulfonamido)butanoate [Compound 18]:** R_f 0.36 (95:5, DCM:MeOH); $[\alpha]_D$ +0.14 (c 0.92, $CHCl_3$); ^1H NMR (400 MHz, $CDCl_3$) δ 9.02 (1H, dd, *J* = 4.2, 1.9 Hz, NapthH7), 8.09 (1H, dd, *J* = 8.1, 1.9 Hz, NapthH5), 8.02 (1H, d, *J* = 8.2 Hz,

NapthH4), 7.38 (1H, dd, J = 8.2, 4.2 Hz, NapthH6), 7.28 (1H, d, J = 8.2 Hz, NapthH3), 6.87 (2H, s, Mes-H3+H5), 5.98 (1H, t, J = 5.9 Hz, N*H*), 5.56 (1H, br, N*H*), 3.70-3.76 (1H, m, H1), 3.56-3.60 (1H, m, C*H*), 3.39 (s, 3H, OCH_3), 3.18-3.05 (2H, m, H3+NapthCH_2), 2.89-2.74 (2H, m, CONHC*HH*'), 2.56 (6H, s, Mes-CH_3), 2.27 (2H, dt, J = 11.6, 8.1 Hz, CH_2CH), 2.21 (3H, s, Mes-CH_3), 2.08-1.88 (2H, m, H2+H4), 1.74-1.57 (2H, m, H2'+H4'); ^{13}C NMR (101 MHz, CDCl3) δ 175.0 (C), 172.1 (C), 164.9 (C), 156.1 (C), 153.3 (CH), 142.6 (C), 139.2 (C), 137.0 (CH), 136.8 (CH), 133.1 (C), 132.0 (CH), 122.9 (CH), 121.5 (CH), 121.1 (C), 52.9 (CH), 52.6 (CH), 45.5 (CH_2), 36.5 (CH), 35.2 (CH_2), 32.5 (CH_2), 31.6 (CH_2), 31.5 (CH_2), 31.0 (CH_3), 23.0 (CH_3), 20.9 (CH_3); *m/z* (AP+) 539.3 ([M+H]+, 80%), 91.1 (100%); HRMS found ([M+H]+) 539.2314, $C_{28}H_{35}N_4S$ req. 539.2323.

Methyl (*S*)-4-((1*r,3*R**)-3-(2-(1,8-naphthyridin-2-yl)ethyl)cyclobutane-1-carboxamido)-2-(phenylsulfonamido)butanoate [Compound 19]:** R_f 0.27 (95:5, DCM:MeOH); IR ν_{max}/cm^{-1} 3333.2 (NH), 2936.2 (CH), 1703.1 (C=O ester), 1639.2 (C=O amide); ^{1}H NMR (400 MHz, $CDCl_3$) δ 9.01 (1H, dd, J = 4.3, 2.0 Hz, NapthH7), 8.09 (1H, dd, J = 8.1, 2.0 Hz, NapthH5), 8.03 (1H, d, J = 8.1 Hz, NapthH4), 7.79-7.72 (2H, m, Ph), 7.56-7.41 (3H, m, Ph), 7.38 (1H, dd, J = 8.1, 4.3 Hz, NapthH6), 7.32 (1H, d, J = 8.1 Hz, NapthH3), 5.99 (1H, brt, J = 6.3 Hz, N*H*), 5.62 (1H, brd, J = 9.2 Hz, N*H*), 3.83 (1H, td, J = 9.6, 4.3 Hz), 3.67-3.51 (1H, m), 3.38 (3H, s, CH_3), 3.20-3.08 (1H, m), 2.91 (2H, dd, J = 7.6, 6.1 Hz), 2.81-2.68 (1H, m), 2.29-2.17 (3H, m), 2.02-1.97 (1H, ddd, J = 18.2, 9.6, 5.0 Hz), 1.91-1.96 (2H, m), 1.87-1.79 (2H, m), 1.61 (1H, tt, J = 9.6, 4.5 Hz); ^{13}C NMR (101 MHz, $CDCl_3$) δ 175.2 (C), 172.0 (C), 166.5 (C), 155.9 (C), 153.3 (CH), 139.1 (C), 137.0 (CH), 136.8 (CH), 133.1 (CH), 129.2 (CH), 127.1 (CH), 122.6 (CH), 121.5 (CH), 121.1 (C), 53.4 (CH), 52.7 (CH_3), 39.1 (CH_2), 36.7 (CH_2), 36.2 (CH), 36.1 (CH_2), 35.1 (CH_2), 32.5 (CH_2), 31.5 (CH_2), 31.2 (CH); *m/z* (AP-) 509.31 ([M-H]-, 10%),

140.86 (100%); HRMS found ([M-H]-) 509.1854. $C_{26}H_{29}O_5N_4S$ req. 509.1864; LCMS rt 3.56, *m*/*z* (ES+) 511.3 ([M+H]+, 100%), >95%.

Methyl (*S*)-4-((1*r,3*R**)-3-(2-(1,8-naphthyridin-2-yl)ethyl)cyclobutane-1-carboxamido)-2-(2,4,6-trimethylphenylsulfonamido)butanoate [Compound 20]:** R_f 0.31 (95:5, DCM:MeOH); $[\alpha]_D$ -0.057 (c 0.3, $CHCl_3$); IR ν_{max}/cm^{-1} 3329.5 (NH), 2931.2 (CH), 1706.1 (C=O ester), 1632.9 (C=O amide); ^1H NMR (400 MHz, $CDCl_3$) δ 9.02 (1H, br, NapthH7), 8.10 (1H, brd, *J* = 7.2 Hz, NapthH5), 8.03 (1H, d, *J* = 8.3 Hz, NapthH4), 7.38-7.40 (1H, brm, NapthH6), 7.32 (1H, d, *J* = 8.3 Hz, NapthH3), 6.88 (2H, s, Mes-H3+H5), 5.93 (1H, brt. *J* = 5.6 Hz, N*H*), 5.52 (1H, d, *J* = 9.4 Hz, N*H*), 3.72 (1H, td, *J* = 9.6, 4.3 Hz), 3.55-3.62 (1H, m), 3.38 (3H, s, OCH_3), 3.11 (1H, tt, *J* = 9.7, 4.9 Hz), 2.90 (2H, br), 2.74-2.65 (1H, m Hz), 2.57 (6H, s, Mes-CH_3), 2.22 (3H, s, MesCH_3), 2.17-2.22 (1H, m), 2.04-1.72 (7H, m), 1.63-1.55 (1H, m); ^{13}C NMR (101 MHz, $CDCl_3$) δ 175.05 (C), 172.16 (C), 166.54 (C), 155.83 (C), 153.30 (CH), 142.68 (C), 139.12 (C), 137.00 (CH+CH), 132.99 (C), 132.05 (CH), 122.59 (CH), 121.47 (CH), 121.06 (C), 52.78 (CH), 52.63 (CH_3), 36.68 (CH_2), 36.20 (CH), 36.00 (CH_2), 35.09 (CH_2), 32.50 (CH_2), 31.47 (CH_2), 31.40 (CH_2), 31.13 (CH), 23.01 (CH_3), 20.96 (CH_3); *m*/*z* (AP+) 553.23 ([M+H]+, 10%), 239.02 (100%); HRMS found ([M+H]+) 553.2469. $C_{29}H_{37}O_5N_4S$ req. 553.2479; LCMS rt 7.81, *m*/*z* (ES+) 553.4 ([M+H]+, 100%), >95%.

General procedure for tetrahydronapthyridine synthesis

Methyl (*S*)-2-(phenylsulfonamido)-4-((1*s,3*R**)-3-((5,6,7,8-tetrahydro-1,8-naphthyridin-2-yl)methyl)cyclobutane-1-carboxamido)butanoate [Compound 21]:** Methyl (*S*)-4-((1*s**,3*R**)-3-((1,8-naphthyridin-2-yl)methyl)cyclobutane-1-carboxamido)-2-(phenylsulfonamido)butanoate **17** (50 mg, 0.10 mmol) was dissolved in MeOH (5 mL), PtO_2 (5 mg) was added and the reaction mixture stirred under 1 atm of H_2 for 23 hours. The reaction

mixture was filtered through Celite® and concentrated *in vacuo*. The crude residue was purified *via* PTLC (95:5, DCM:MeOH) to yield **21** as a clear oil (8 mg, 0.016 mmol, 16%); R_f 0.29 (95:5, DCM:MeOH); $[\alpha]_D$ +0.11 (c 0.44, $CHCl_3$); IR ν_{max}/cm^{-1} 3318.6 (NH), 3284.8 (NH), 2928.6 (CH), 1733.5 (C=O ester), 1650.6 (C=O amide); ^{1}H NMR (400 MHz, $CDCl_3$) δ 7.81-7.71 (2H, m, Ph), 7.55-7.47 (1H, m, Ph), 7.44 (2H, t, *J* = 7.5 Hz, Ph), 7.01 (1H, d, *J* = 7.2 Hz, Ar), 6.24 (1H, d, *J* = 7.2 Hz, Ar), 6.00 (1H, brt, *J* = 5.6 Hz, N*H*), 5.50 (1H, vbrs, N*H*), 3.84 (1H, dd, *J* = 9.8, 4.2 Hz, H1), 3.60-3.52 (1H, m, C*H*), 3.39 (s, 3H, OCH_3), 3.33 (2H, brt, *J* = 4.6 Hz, CH_2N), 3.23-3.10 (1H, m, H3), 2.75 (1H, tt, *J* = 9.6, 8.6 Hz), 2.69-2.48 (5H, m, THN+CONHCH_2), 2.27-2.18 (2H, m), 2.06-2.97 (1H, m), 1.96-1.88 (2H, m, H2+H4), , 1.87-1.79 (2H, m, THN), 1.68-1.56 (2H, m, H2'+H4'); ^{13}C NMR (101 MHz, $CDCl_3$) δ 175.1 (C), 171.9 (C), 158.5 (C), 155.2 (C), 139.2 (C), 137.3 (CH), 133.0 (CH), 129.2 (CH), 127.2 (CH), 114.3 (C), 111.3 (CH), 53.5 (CH), 52.7 (CH_3), 41.5 (CH_2), 36.3 (CH), 35.3 (CH_2), 32.6 (CH_2), 31.5 (CH_2), 31.5 (CH_2), 31.3 (CH), 26.2 (CH_2), 21.1 (CH_2); *m/z* (AP+) 501.2 ([M+H]+, 100%); HRMS found ([M+H]+) 501.2161 $C_{25}H_{33}O_5N_4S$ req. 501.2166; LCMS rt 2.01, *m/z* (ES+) 501.3 ([M+H]+, 100%), >95%.

Methyl (*S*)-4-((1*s,3*R**)-3-((5,6,7,8-tetrahydro-1,8-naphthyridin-2-yl) methyl)cyclobutane-1-carboxamido)-2-(2,4,6-trimethylphenylsulfonamido)butanoate [Compound 22]:** R_f 0.34 (95:5, DCM:MeOH); $[\alpha]_D$ +0.09 (c 0.375, $CHCl_3$); IR ν_{max}/cm^{-1} 3274.8 (NH), 2932.8 (CH), 1737.5 (C=O ester), 1655.8 (C=O amide); ^{1}H NMR (400 MHz, $CDCl_3$) δ 7.00 (1H, d, *J* = 7.2 Hz, Ar), 6.88 (2H, s, Mes-H3,5), 6.23 (1H, d, *J* = 7.2 Hz, Ar), 5.90 (1H, brt, *J* = 6.1 Hz, N*H*), 5.51 (1H, br, N*H*), 3.73 (1H, dd, *J* = 9.8, 4.3 Hz, H1), 3.61-3.53 (1H, m, C*H*), 3.39 (3H, s, OCH_3), 3.37-3.31 (2H, m, THN), 3.15-3.07 (1H, m, H3), 2.71 (1H, tt, *J* = 9.6, 8.6 Hz), 2.62 (2H, t, *J* = 6.3 Hz, THN), 2.57 (6H, s, Mes-CH_3), 2.58-2.47 (2H, m CONHCH_2), 2.22 (3H, s, Mes-CH_3), 2.22-2.15 (2H, m), 2.05-1.95 (1H, m), 1.94-1.87

(2H, m, H2+H4), 1.87-1.79 (2H, m, THN), 1.66-1.55 (2H, m, H2'+H4'); ^{13}C NMR (101 MHz, $CDCl_3$) δ 175.0 (C), 172.1 (C), 155.3 (C), 142.7 (C), 139.2 (C), 137.1 (CH), 134.3 (C), 133.1 (C), 132.0 (CH), 114.1 (C), 111.4 (CH), 52.9 (CH), 52.6 (CH_3), 41.5 (CH_2), 36.3 (CH_2), 35.2 (CH), 32.5 (CH_2), 31.5 (CH_2), 31.5 (CH_2), 31.3 (CH), 29.7 (CH_2), 26.2 (CH_2), 23.0 (CH_3), 21.2 (CH_2), 20.9 (CH_3); *m/z* (AP+) 229.1 (100%), 543.2 ([M+H]+, 50%); HRMS Found ([M+H]+) 543.2633. $C_{28}H_{39}O_5N_4S$ req. 543.2636; LCMS rt 3.47, *m/z* (ES+) 543.4 ([M+H]+, 100%), >95%.

Methyl (*S*)-2-(phenylsulfonamido)-4-((1*r,3*R**)-3-(2-(5,6,7,8-tetrahydro-1,8-naphthyridin-2-yl)ethyl)cyclobutane-1-carboxamido)butanoate [Compound 23]:** Rf 0.23 (95:5, DCM:MeOH); $[\alpha]_D$ +0.16 (c 0.50, $CHCl_3$); IR ν_{max}/cm^{-1} 3286.9 (NH), 2933.5 (CH), 1739.2 (C=O ester), 1648.4 (C=O amide); ^{1}H NMR (400 MHz, $CDCl_3$) δ 7.79-7.73 (2H, m, Ph), 7.55-7.49 (1H, m, Ph), 7.49-7.42 (2H, m, Ph), 7.21 (1H, d, *J* = 7.4 Hz, Ar), 6.87 (1H, brs, N*H*), 6.32 (1H, d, *J* = 7.4 Hz, Ar), 6.14 (1H, t, *J* = 5.9 Hz, N*H*), 5.71 (1H, vbr, N*H*), 3.84 (1H, dd, *J* = 9.4, 4.3 Hz), 3.57-3.47 (1H, m), 3.42-3.40 (2H, m), 3.38 (3H, s, CH_3), 3.22-3.12 (1H, m), 2.77 (1H, tt, *J* = 9.6, 8.7 Hz), 2.67 (2H, t, *J* = 6.1 Hz), 2.52-2.46 (2H, m), 2.29-2.13 (3H, m), 2.03-1.95 (1H, m), 1.91-1.77 (5H, m), 1.77-1.60 (4H, m). ^{13}C NMR (101 MHz, $CDCl_3$) δ 175.2 (C), 171.9 (C), 152.7 (C), 151.7 (C), 139.8 (CH), 139.2 (C), 133.0 (CH), 129.2 (CH), 127.2 (CH), 117.0 (C), 110.5 (CH), 53.6 (CH), 52.7 (CH_3), 41.4 (CH_2), 36.1 (CH), 35.9 (CH_2), 35.3 (CH_2), 32.4 (CH_2), 32.0 (CH_2), 31.2 (CH_2), 31.2 (CH_2), 30.71 (CH), 25.8 (CH_2), 19.9 (CH_2); *m/z* (ES+) 515.4 ([M+H]+, 100%); LCMS rt 2.10, *m/z* (ES+) 515.4 ([M+H]+, 100%), >95%.

Methyl (*S*)-4-((1*r,3*R**)-3-(2-(5,6,7,8-tetrahydro-1,8-naphthyridin-2-yl)ethyl)cyclobutane-1-carboxamido)-2-(2,4,6-trimethylphenylsulfonamido)butanoate**

[Compound 24]: R_f 0.25 (95:5, DCM:MeOH); $[\alpha]_D$ +0.08 (c 0.8, $CHCl_3$); IR ν_{max}/cm^{-1} 3382.7 (NH), 3287.4 (NH), 2930.7 (CH), 1727.8 (C=O ester), 1640.7 (C=O amide); ^1H NMR (400 MHz, $CDCl_3$) δ 7.07 (1H,d, *J* = 7.3 Hz, Ar), 6.96 (2H, s, Mes-H3+H5), 6.32 (1H, d, *J* = 7.3 Hz, Ar), 5.99 (1H, brt, *J* = 5.6 Hz, N*H*), 5.61 (1H, brs, N*H*), 5.04 (1H, brs, N*H*), 3.79 (1H, dd, *J* = 9.8, 4.0 Hz), 3.72-3.60 (1H, m), 3.45 (3H, s, OCH_3), 3.43-3.38 (2H, m), 3.23-3.13 (1H, m), 2.81-2.72 (1H, m), 2.76 (1H, tt, *J* = 9.6, 8.6 Hz), 2.68 (2H, t, *J* = 6.1 Hz), 2.65 (6H, s, Mes-C*H*3), 2.50-2.42 (2H, dd, *J* = 8.1, 7.6 Hz), 2.30 (3H, s, Mes-C*H*3), 2.29-2.17 (3H, m), 2.01-2.09 (1H, m), 1.82-1.92 (4H, m), 1.75 (2H, dt, *J* = 8.1, 7.1 Hz), 1.70-1.61 (1H, tt, *J* = 14.1, 4.5 Hz); ^{13}C NMR (101 MHz, $CDCl_3$) δ 175.17 (C), 172.19 (C), 157.57 (C), 155.44 (C), 142.68 (C), 139.14 (C), 136.89 (CH), 133.00 (C), 132.05 (CH), 113.47 (C), 111.11 (CH), 52.77 (CH), 52.63 (CH_3), 41.58 (CH_2), 36.57 (CH_2), 36.28 (s), 35.08 (CH_2), 34.91 (CH_2), 32.50 (CH_2), 31.50 (CH_2), 31.43 (CH_2), 31.13 (CH), 26.30 (CH_2), 23.01 (CH_3), 21.39 (CH_2), 20.97 (CH_3); *m*/*z* (AP+) 557.3 ([M+H]+, 40%), 373.3 ([M-SO_2Mes]+, 20%), 341.3 (100%); HRMS found ([M+H]+) 557.2781. $C_{29}H_{41}O_5N_4S$ req. 557.2792; LCMS rt 3.42, *m*/*z* (ES+) 557.4 ([M+H]+, 100%), >95%.

(1*r, 3*s**)-3-(2-(1,8-Naphthyridin-2-yl)ethyl)cyclobutan-1-amine [Compound 25]:** Methyl (1*r**,3*s**)-3-(2-(1,8-naphthyridin-2-yl)ethyl)cyclobutane-1-carboxylate **16** (292 mg, 1.08 mmol) was dissolved in 6 M aqueous HCl (10 mL) and stirred at RT for 24 hours. The reaction mixture was concentrated *in vacuo* to give the crude acid. This was dissolved in $CHCl_3$ (4 mL) and conc. H_2SO_4 (2 mL) and heated to 45 °C. NaN_3 (140 mg, 2.16 mmol) was then added portionwise and the reaction mixture stirred for 5 hours. It was then allowed to cool to RT and the reaction was quenched with water (2 mL), extracted with DCM (10 mL) and the aqueous phase adjusted to pH 14 with 50% aqueous NaOH. The aqueous layer was then extracted with DCM (4 × 15 mL), dried ($MgSO_4$) and concentrated *in vacuo* to yield **25** as a yellow oil (245

mg, 1.08 mmol, 100%); ^{1}H NMR (400 MHz, $CDCl_3$) δ 9.00 (1H, dd, *J* = 4.2, 1.9 Hz, NapthH7), 8.07 (1H, dd, *J* = 8.2, 1.9 Hz, NapthH5), 8.01 (1H, d, *J* = 8.2 Hz, NapthH4), 7.36 (1H, dd, *J* = 8.2, 4.2 Hz, NapthH6), 7.29 (1H, d, *J* = 8.2 Hz, NapthH3), 3.14 (1H, tt, *J* = 8.6, 7.1 Hz, H1), 2.92-2.86 (2H, t, *J* =7.6 Hz, NapthCH_2), 2.41-2.31 (2H, m), 1.90 (2H, dt, *J* = 9.1, 7.5 Hz), 1.85-1.76 (1H, m, H3), 1.43 (2H, brs, NH_2), 1.26-1.18 (2H, m); ^{13}C NMR (101 MHz, $CDCl_3$) δ 166.7 (C), 156.0 (C), 153.3 (CH), 136.8 (CH), 136.7 (CH), 122.6 (CH), 121.4 (CH), 121.0 (C), 44.9 (CH), 40.5 (CH_2), 37.3 (CH_2), 36.8 (CH_2), 27.4 (CH); *m/z* (AP+) 227.8 ([M+H]+, 100%); HRMS Found ([M+H]+) 228.1499. $C_{14}H_{18}N_3$ req. 228.1495.

(*S*)-4-Methoxy-4-oxo-3-(phenylsulfonamido)butanoic acid [Compound 26]: Sodium bicarbonate (53 mg, 0.63 mmol) was added to a stirred suspension of (*S*)-Asp-(OMe) (74 mg, 0.5 mmol) in H_2O:dioxane (1:1, 3 mL) and the mixture stirred for 30 minutes at RT. Phenylsulfonyl chloride (0.05 mL, 74 mg, 0.42 mmol) was added and the mixture stirred for a further 22 hours. The dioxane was removed *in vacuo*, the reaction mixture acidified with 3 M aqueous HCl resulting in a fine white precipitate and the mixture was extracted with DCM (5 × 20 mL). The combined organic layers were dried ($MgSO_4$) and concentrated *in vacuo* to yield **26** as a clear oil (75 mg, 0.26 mmol, 62%); ^{1}H NMR (400 MHz, $CDCl_3$) δ 8.00 (1H, vbrs), 7.83-7.78 (2H, m, Ph), 7.52 (1H, tt, *J* = 2.5, 7.6 Hz, Ph), 7.47-7.42 (2H, m, Ph), 6.21 (1H, brd, *J* = 8.4 Hz, N*H*), 4.17-4.06 (1H, brm, C*H*), 3.49 (3H, s, OCH_3), 3.00 (1H, dd, *J* = 17.5, 4.2 Hz, C*H*H'), 2.83 (1H, dd, *J* = 17.5, 4.7 Hz, CH*H'*); ^{13}C NMR (101 MHz, $CDCl_3$) δ 174.7 (C), 170.3 (C), 139.6 (C), 133.0 (CH), 129.1 (CH), 127.2 (CH), 53.0 (CH_3), 51.9 (CH), 37.8 (C); *m/z* (AP+) 288.0 ([M+H]+, 100%); HRMS Found ([M+H]+) 288.0542. $C_{11}H_{14}O_6NS$ req. 288.0536.

(*S*)-4-Methoxy-4-oxo-3-(2,4,6-trimethylphenylsulfonamido)butanoic acid [Compound 27] was prepared according to the same procedure using 2,4,6-trimethylphenylsulfonyl chloride (92 mg, 0.42 mmol). The crude product was purified *via* PTLC (95:5, DCM:MeOH) to yield **27** as a clear oil (36 mg, 0.11 mmol, 26%); ^{1}H NMR (400 MHz, $CDCl_3$) δ 6.88 (2H, s, Mes-H3+H5), 5.96 (1H, d, *J* = 6.2 Hz, N*H*), 4.04-3.96 (1H, dt, *J* = 4.5, 7.6 Hz, C*H*), 3.55 (3H, s, OCH_3), 2.94 (1H, dd, *J* = 17.2, 4.5 Hz, C*H*H'), 2.81 (1H, dd, *J* = 17.2, 4.5 Hz, CH*H'*), 2.57 (6H, s, Mes-CH_3), 2.22 (3H,s, Mes-CH_3); ^{13}C NMR (101 MHz, $CDCl_3$) δ 175.1 (C), 170.6 (C), 142.6 (C), 139.32 (C), 133.3 (C), 132.0 (CH), 53.1 (CH_3), 51.6 (CH), 37.6 (CH_2), 22.9 (CH_3), 21.0 (CH_3); *m/z* (AP+) 330.1 ([M+H]+, 100%). (The purified material had a broad ^{1}H NMR spectrum. Multiplet and coupling constant analysis is from the crude product).

Methyl *N*^{4}-((1*r,3*R**)-3-(2-(1,8-naphthyridin-2-yl)ethyl)cyclobutyl)-*N*^{2}-(phenylsulfonyl)-*L*-asparaginate [Compound 28]** and ***N*-((*S*)-1-((1*r**,3*R**)-3-(2-(1,8-Naphthyridin-2-yl)ethyl)cyclobutyl)-2,5-dioxopyrrolidin-3-yl)benzenesulfonamide [Compound 30]:** EDCI (103 mg, 0.54 mmol), HOBt (73 mg, 0.54 mmol) and DIPEA (0.16 mL, 116 mg, 0.90 mmol) were added to a stirred solution of (1*r**,3*s**)-3-(2-(1,8-naphthyridin-2-yl)ethyl)cyclobutan-1-amine **19** (41 mg, 0.18 mmol) and (*S*)-4-methoxy-4-oxo-3-(phenylsulfonamido)butanoic acid **26** (51 mg, 0.18 mmol) in DCM (4 mL) and stirred at RT under a blanket of N_2 for 24 hours. The reaction mixture was diluted with water (10 mL) and extracted with DCM (3 × 15 mL). The combined organic layers were washed with saturated aqueous ammonium chloride (10 mL), dried ($MgSO_4$) and concentrated *in vacuo*. The crude residue was purified *via* PTLC (95:5, DCM:MeOH) to yield **28** as a clear oil (39 mg, 0.079 mmol, 44%); R_f 0.17 (95:5, DCM:MeOH); $[\alpha]_D$ +0.135 (c 1.1, $CHCl_3$); IR ν_{max}/cm^{-1} 3260.6 (NH), 2923.4 (CH), 1735.0 (C=O ester), 1632.4 (C=O amide); ^{1}H NMR (400 MHz, $CDCl_3$) δ 9.08 (1H, dd, *J* = 4.1, 1.9 Hz, NapthH7), 8.17 (1H, dd, *J* = 8.1, 1.9 Hz, NapthH5), 8.10 (1H, d, *J* = 8.2 Hz, NapthH4),

7.86 (2H, d, *J* = 7.2 Hz, Ph), 7.59-7.54 (1H, m, Ph), 7.52-7.44 (3H, m, Ph+Ar-H6), 7.37 (1H,d, *J* = 8.2 Hz, NapthH3), 6.07 (2H, brt, *J* = 7.0 Hz, 2N*H*), 4.19-4.07 (2H, m, H1+C*H*), 3.53 (3H, s, OCH_3), 2.96 (2H, t, *J* = 7.5 Hz, NapthCH_2), 2.87 (1H, dd, *J* = 15.7, 4.4 Hz, C*H*H'), 2.64 (1H, dd, *J* = 15.7, 4.6 Hz, CH*H'*), 2.51-2.39 (2H, m, NapthCH_2CH_2), 2.09-1.94 (m, 3H, H2+H3+H4), 1.43-1.51 (2H, m, H2'+H4'); ^{13}C NMR (101 MHz, $CDCl_3$) δ 170.8 (C), 168.1 (C), 166.4 (C), 155.9 (C), 153.3 (CH), 140.0 (C), 137.1 (CH), 136.9 (CH), 132.8 (CH), 129.0 (CH), 127.1 (CH), 122.7 (CH), 121.6 (CH), 121.1 (C), 52.8 (CH_3), 52.6 (CH), 41.2 (CH), 38.9 (CH_2), 37.0 (CH_2), 36.9 (CH_2), 36.9 (CH_2), 36.2 (CH_2), 28.7 (CH); *m/z* (ES+) 497.0 ([M+H]+, 100%); HRMS Found ([M+H]+) 497.1848. $C_{25}H_{29}O_5N_4S$ req. 497.1853; LCMS rt 3.52, *m/z* (ES+) 497.3 ([M+H]+, 100%), >95%. And **30** as a white solid (15 mg, 0.032 mmol, 18%); R_f 0.25 (95:5, DCM:MeOH); $[\alpha]_D$ +0.150 (c 0.9, $CHCl_3$); IR ν_{max}/cm^{-1} 3310.8 (NH), 2928.1 (CH), 1737.9 (C=O succinimide); ^{1}H NMR (400 MHz, $CDCl_3$) δ 8.96 (1H, dd, *J* = 4.3, 2.0 Hz, NapthH7), 8.08 (1H, dd, *J* = 8.2, 2.0 Hz, NapthH5), 8.01 (1H, d, *J* = 8.2 Hz, NapthH4), 7.847.82 (2H, dt, *J* = 7.0, 1.5 Hz, Ph), 7.555 (1H, tt, *J* = 7.0, 1.5 Hz, Ph), 7.47 (2H, tt, *J* = 7.0, 1.5 Hz, Ph), 7.37 (1H, dd, *J* = 8.2, 4.3 Hz, NapthH6), 7.29 (1H, d, *J* = 8.2 Hz, NapthH3), 6.20 (1H, s, N*H*), 4.26 (1H, tt, *J* = 9.6, 8.1 Hz, H1), 4.03 (1H, dd, *J* = 8.6, 6.1 Hz, C*H*), 2.94 (1H, dd, J = 18.1, 9.1 Hz, C*H*H'), 2.88-2.86 (2H, t, *J* =7.6 Hz, NapthCH_2), 2.78 (1H, dd, *J* = 18.1, 6.1 Hz, CH*H'*), 2.34-2.21 (2H, m, H2,4), 2.18-2.07 (2H, m, H2,4), 2.00-1.93 (3H, m, H3 + NapthCH_2CH_2); ^{13}C NMR (101 MHz, $CDCl_3$) δ 174.7 (C), 174.0 (C), 166.4 (C), 155.8 (C), 153.2 (CH), 139.1 (C), 137.1 (CH), 136.8 (CH), 133.3 (CH), 129.4 (CH), 127.3 (CH), 122.6 (CH), 121.5 (CH), 121.0 (C), 51.3 (CH), 42.5 (CH), 36.9 (CH_2), 36.8 (CH_2), 35.9 (CH_2), 32.3 (CH_2), 32.0 (CH_2), 28.8 (CH); *m/z* (ES+) 465.0 ([M+H]+, 100%); HRMS Found ([M+H]+) 465.1587. $C_{24}H_{25}O_4N_4S$ req. 465.1591; LCMS rt 3.56, *m/z* (ES+) 465.3 ([M+H]+, 100%), >95%.

Methyl *N*^{4}-((1*r,3*R**)-3-(2-(1,8-naphthyridin-2-yl)ethyl)cyclobutyl)-*N*^{2}-(mesitylsulfonyl)-*L*-asparaginate [Compound 29]** and ***N*-((*S*)-1-((1*r**,3*R**)-3-(2-(1,8-Naphthyridin-2-yl)ethyl)cyclobutyl)-2,5-dioxopyrrolidin-3-yl)-2,4,6-trimethylbenzenesulfonamide [Compound 31]** were obtained according to the same procedure using **25** (25 mg, 0.11 mmol) and (*S*)-4-methoxy-4-oxo-3-(2,4,6-trimethylphenylsulfonamido)butanoic acid **27** (36 mg, 0.11 mmol) to yield **29** as a yellow oil (26 mg, 0.048 mmol, 44%); R_f 0.17 (95:5, DCM:MeOH); $[\alpha]_D$ +0.234 (c 0.87, $CHCl_3$); IR ν_{max}/cm^{-1} 3274.2 (NH), 2937.1 (CH), 1742.6 (C=O ester), 1644.3 (C=O amide); ^1H NMR (400 MHz, $CDCl_3$) δ 9.02 (1H, dd, *J* = 4.3, 1.9 Hz, NapthH7), 8.09 (1H, dd, *J* = 8.2, 1.9 Hz, NapthH5), 8.03 (1H, d, *J* = 8.2 Hz, NapthH4), 7.39 (1H, dd, *J* = 8.2, 4.3 Hz, NapthH6), 7.29 (1H, d, *J* = 8.2 Hz, NapthH3), 6.86 (2H, s, Mes-H3+H5), 5.99 (1H, d, *J* = 7.7 Hz, N*H*), 5.85 (1H, d, *J* = 7.8 Hz, N*H*), 4.06 (1H, tt, *J* = 8.6, 7.6 Hz, H1), 3.98 (1H, dt, *J* = 7.6, 4.5 Hz, C*H*), 3.50 (3H, s, OCH_3), 2.89 (2H, t, *J* = 7.4 Hz, NapthCH_2), 2.76 (1H, dd, *J* = 15.7, 4.5 Hz, C*H*H'), 2.56 (6H, s, MesCH_3), 2.52 (1H, dd, *J* = 15.7, 4.5 Hz, CH*H'*), 2.43-2.35 (2H, m, H2+H4), 2.21 (3H, s, MesCH_3), 1.96-1.88 (3H, m, H3+NapthCH_2CH_2), 1.43-1.36 (2H, m, H2'+H4'); ^{13}C NMR (101 MHz, $CDCl_3$) δ 171.1 (C), 168.2 (C), 166.3 (C), 155.9 (C), 153.4 (CH), 142.3 (C), 139.1 (C), 137.1 (CH), 136.8 (CH), 133.9 (C), 131.9 (CH), 122.7 (CH), 121.5 (CH), 121.1 (C), 52.8 (CH_3), 52.3 (CH), 41.2 (CH), 38.7 (CH_2), 37.0 (CH_2), 36.9 (CH_2), 36.2 (CH_2), 28.7 (CH), 22.9 (CH_3), 21.0 (CH_3); *m/z* (ES+) 539.0 ([M+H]+, 100%); HRMS Found ([M+H]+) 539.2318. $C_{28}H_{35}O_5N_4S$ req. 539.2323; LCMS rt 7.48, *m/z* (ES+) 539.3 ([M+H]+, 100%), >95%. And **31** as a yellow oil (26 mg, 0.051 mmol, 46%); R_f 0.22 (95:5, DCM:MeOH); $[\alpha]_D$ +0.034 (c 0.87, $CHCl_3$); IR ν_{max}/cm^{-1} 2923.1 (CH), 1735.0 (C=O succinimide); ^1H NMR (400 MHz, $CDCl_3$) δ 9.00 (1H, dd, *J* = 4.2, 2.0 Hz, NapthH7), 8.09 (1H, dd, *J* = 8.1, 2.0 Hz, NapthH5), 8.03 (1H, d, *J* = 8.1 Hz, NapthH4), 7.38 (1H, dd, *J* = 8.1, 4.2 Hz, NapthH6), 7.31 (1H, d, *J* = 8.1 Hz, NapthH3), 6.92 (2H, s, Mes-H3+H5), 5.55 (1H, brs, N*H*), 4.28 (1H, tt, *J* = 9.5, 8.1 Hz, H1), 3.87 (1H, dd, *J* = 8.5, 6.2 Hz, C*H*), 2.90 (2H, t, *J* =

7.6 Hz, NapthC*H*$_2$), 2.86 (1H, dd, *J* = 18.2, 8.5 Hz, C*H*H'), 2.62 (1H, dd, *J* = 18.2, 6.2 Hz, CH*H'*), 2.59 (6H,s, Mes-CH$_3$), 2.38-2.28 (2H, m, H2+H4), 2.24 (3H, s, Mes-CH$_3$), 2.23-2.15 (2H, m, H2'+H4'), 1.98-2.20 (3H, m, NapthCH$_2$C*H*$_2$+H3); ^{13}C NMR (101 MHz, CDCl$_3$) δ 174.9 (C), 173.9 (C), 166.3 (C), 155.9 (C), 153.3 (CH), 143.2 (C), 139.4 (C), 137.1 (CH), 136.8 (CH), 132.3 (CH), 132.2 (C), 122.5 (CH), 121.5 (CH), 121.0 (C), 51.1 (CH), 42.6 (CH), 36.9 (CH$_2$), 36.8 (CH$_2$), 35.8 (CH$_2$), 32.4 (CH$_2$), 32.0 (CH$_2$), 28.8 (CH), 22.9 (CH$_3$), 21.0 (CH$_3$); *m/z* (ES+) 507.0 ([M+H]+, 100%); HRMS Found ([M+H]+) 507.2053. $C_{27}H_{31}O_4N_4S$ req. 507.206; LCMS rt 8.12, *m*/*z* (ES+) 507.3 ([M+H]+, 100%), >95%.

Methyl *N*^{2}-(phenylsulfonyl)-*N*^{4}-((1*r,3*R**)-3-(2-(5,6,7,8-tetrahydro-1,8-naphthyridin-2-yl)ethyl)cyclobutyl)-*L*-asparaginate [Compound 32]** was prepared from **28** (63 mg, 0.13 mmol) using the general procedure for tetrahydronapthyridine synthesis to yield **32** as a white solid (30 mg, 0.06 mmol, 46%); R$_f$ 0.36 (95:5, DCM:MeOH); [α]$_D$ +0.170 (c 1.00, CHCl$_3$); IR ν_{max}/cm^{-1} 3252.8 (NH), 2923.8 (CH), 1743.4 (C=O ester), 1644.8 (C=O amide); ^{1}H NMR (400 MHz, CDCl$_3$) δ 7.79 (2H, dd, *J* = 7.6, 2.0 Hz, Ph), 7.50 (1H, tt, *J* = 7.6, 2.0 Hz, Ph), 7.42 (2H, td, *J* = 7.6, 2.0 Hz, Ph), 7.01 (1H, d, *J* = 7.2 Hz, Ar), 6.24 (1H, d, *J* = 7.2 Hz, Ar), 5.92 (1H, brs, N*H*), 5.19 (1H, br, N*H*), 4.10-4.00 (2H, m, H1+C*H*), 3.46 (3H, s, OC*H*$_3$), 3.34-3.31 (2H, m, THN), 2.76 (1H, dd, *J* = 15.6, 4.4 Hz, C*H*H'), 2.62 (2H, t, *J* = 6.2 Hz, THN), 2.55 (1H, dd, *J* = 15.6, 4.7 Hz, CH*H'*), 2.42-2.33 (4H, m, ArC*H*$_2$+H2+H4), 1.84 (3H, m, H2'+H3+H4'), 1.67 (2H, dt, *J* = 15.3, 7.5 Hz, THN), 1.42-1.31 (2H, m, ArCH$_2$C*H*$_2$); ^{13}C NMR (101 MHz, CDCl$_3$) δ 170.8 (C), 168.1 (C), 156.8 (C), 155.3 (C), 140.0 (C), 137.2 (CH), 132.7 (CH), 129.0 (CH), 127.1 (CH), 113.9 (C), 111.1 (CH), 52.8 (CH$_3$), 52.7 (CH), 41.5 (CH$_2$), 41.4 (CH), 38.9 (CH$_2$), 36.9 (CH$_2$), 36.8 (CH$_2$), 34.9 (CH$_2$), 28.6 (CH), 26.2 (CH$_2$), 21.2 (CH$_2$); *m/z* (AP+) 501.3 ([M+H]+ 100%), 469.2 ([M-OMe]+ 20%); HRMS Found ([M+H]+) 501.2158. $C_{25}H_{33}O_5N_4S$ req. 501.2166; LCMS rt 1.88, *m*/*z* (ES+) 501.3 ([M+H]+, 100%), >95%.

Methyl *N*^{2}-(mesitylsulfonyl)-*N*^{4}-((1*r,3*R**)-3-(2-(5,6,7,8-tetrahydro-1,8-naphthyridin-2-yl)ethyl)cyclobutyl)-*L*-asparaginate [Compound 33]** was prepared from **29** (35 mg, 0.065 mmol) using the general procedure for tetrahydronapthyridine synthesis to yield **33** as a pale yellow gummy solid (21 mg, 0.039 mmol, 60%): R_f 0.30 (95:5, DCM:MeOH); $[\alpha]_D$ +0.202 (c 1.05, $CHCl_3$); IR ν_{max}/cm^{-1} 3310.4 (NH), 2927.9 (CH), 1737.8 (C=O ester), 1639.1 (C=O amide); ^1H NMR (400 MHz, $CDCl_3$) δ 7.03 (1H, d, *J* = 7.3 Hz, Ar), 6.86 (2H, s, Mes-H3,5), 6.24 (1H, d, *J* = 7.3 Hz, Ar), 5.94 (1H, brd, *J* = 7.6 Hz, N*H*), 5.42 (1H, brs, N*H*), 4.06 (1H, tt, *J* = 8.6, 7.6 Hz, H1), 3.98 (1H, t, *J* = 4.5 Hz, C*H*), 3.50 (3H, s, OCH_3), 3.37-3.30 (2H, m, THN), 2.74 (1H, dd, *J* = 15.6, 4.5 Hz, C*H*H'), 2.62 (2H, t, *J* = 6.2 Hz, THN), 2.56 (6H, s, Mes-CH_3), 2.52 (1H, dd, *J* = 15.6, 4.5 Hz, CH*H'*), 2.42-2.33 (4H, m, ArCH_2+H2+H4), 2.21 (3H, s, Mes-CH_3), 1.89-1.81 (3H, m, THN+H3), 1.69 (2H, dt, *J* = 15.4, 7.5 Hz, $ArCH_2CH_2$), 1.42-1.35 (2H, m, H2'+H4'); ^{13}C NMR (101 MHz, $CDCl_3$) δ 171.1 (C), 168.2 (C), 156.1 (C), 155.0 (C), 142.2 (C), 139.1 (C), 137.5 (CH), 134.0 (C), 131.9 (CH), 114.3 (C), 111.0 (CH), 52.7 (CH_3), 52.4 (CH), 41.5 (CH_2), 41.4 (CH), 38.7 (CH_2), 36.8 (CH_2), 36.8 (CH_2), 34.6 (CH_2), 28.6 (CH), 26.2 (CH_2), 22.9 (CH_3), 21.1 (CH_2), 20.9 (CH_3); *m/z* (AP+) 543.2 ([M+H]+ 10%), 511.1 ([M-OMe]+ 80%); HRMS Found ([M+H]+) 543.2629. $C_{28}H_{39}O_5N_4S$ req. 543.2636; LCMS rt 3.46, *m*/*z* (ES+) 543.4 ([M+H]+, 100%), >95%.

***N*-((*S*)-2,5-dioxo-1-((1*r**,3*R**)-3-(2-(5,6,7,8-tetrahydro-1,8-naphthyridin-2-yl)ethyl)cyclobutyl)pyrrolidin-3-yl)benzenesulfonamide [Compound 34]** was prepared from **30** (30 mg, 0.065 mmol) using the general procedure for tetrahydronapthyridine synthesis to yield **34** as a white solid (15 mg, 0.032 mmol, 49%): R_f 0.33 (95:5, DCM:MeOH); $[\alpha]_D$ +0.075 (c 0.735, $CHCl_3$); IR ν_{max}/cm^{-1} 2937.4 (CH), 1700.3 (C=O succinimide); ^1H NMR (400 MHz, $CDCl_3$) δ 7.86-7.81 (2H, dd, *J* = 7.6, 1.5 Hz, Ph), 7.55 (1H, tt, *J* = 7.6, 1.5 Hz, Ph), 7.48

(2H, t, J = 7.6 Hz, Ph), 6.98 (1H, d, J = 7.3 Hz, Ar), 6.22 (1H, d, J = 7.3 Hz, Ar), 4.94 (1H, brs, N*H*), 4.26 (1H, tt, J = 9.6, 8.6 Hz, H1), 3.95 (1H, dd, J = 8.7, 6.0 Hz, C*H*), 3.31 (2H, brt, J = 5.5 Hz, THN), 2.95 (1H, dd, J = 18.2, 8.7 Hz, C*H*H'), 2.68 (1H, dd, J = 18.2, 6.0 Hz, CH*H'*), 2.61 (2H, t, J = 6.3 Hz, THN), 2.37 (2H, t, J = 7.5 Hz, ArCH_2), 2.31-2.23 (2H, m, H2+H4), 2.21-2.13 (2H, m, H2'+H4'), 1.95-1.87 (1H, m, H3), 1.85-1.78 (2H, m, THN), 1.74 (2H, dt, J = 15.3, 7.5 Hz, ArCH$_2$CH_2); ^{13}C NMR (101 MHz, CDCl$_3$) δ 174.7 (C), 173.7 (C), 157.4 (C), 155.5 (C), 138.8 (C), 136.8 (CH), 133.4 (CH), 129.4 (CH), 127.3 (CH), 113.5 (C), 111.1 (CH), 51.3 (CH), 42.7 (CH), 41.6 (CH$_2$), 37.0 (CH$_2$), 36.3 (CH$_2$), 35.1 (CH$_2$), 32.4 (CH$_2$), 32.1 (CH$_2$), 28.8 (CH), 26.3 (CH$_2$), 21.4 (CH$_2$); *m/z* (ES+) 469.0 ([M+H]+ 100%); HRMS Found ([M+H]+) 469.1900. $C_{24}H_{29}O_4N_4S$ req. 469.1904; LCMS rt 2.33, *m/z* (ES+) 469.3 ([M+H]+, 100%), >95%.

***N*-((*S*)-2,5-Dioxo-1-((1*r**,3*R**)-3-(2-(5,6,7,8-tetrahydro-1,8-naphthyridin-2-yl)ethyl)cyclobutyl)pyrrolidin-3-yl)-2,4,6-trimethylbenzenesulfonamide [Compound 35]** was prepared from **31** (10 mg, 0.02 mmol) using the general procedure for tetrahydronapthyridine synthesis to yield **35** as a yellow oil (3 mg, 0.0065 mmol, 33%): R_f 0.30 (95:5, DCM:MeOH); $[\alpha]_D$ +0.079 (c 0.165, CHCl$_3$); IR ν_{max}/cm^{-1} 2939.9 (CH), 1706.1 (C=O succinimide); ^{1}H NMR (400 MHz, CDCl$_3$) δ 6.99 (1H, d, J = 7.3 Hz, Ar), 6.92 (2H, s, Mes-H3,5), 6.24 (1H, d, J = 7.3 Hz, Ar), 5.10 (1H, brs, N*H*), 4.27 (1H, tt. J = 10.1, 8.6 Hz, H1), 3.83 (1H, dd, J = 8.6, 6.0 Hz, C*H*), 3.36-3.29 (2H, m, THN), 2.85 (1H, dd, J = 18.2, 8.6 Hz, C*H*H'), 2.61 (1H, dd, J = 18.2, 6.0 Hz, CHH'), 2.59 (6H, s, Mes-CH_3), 2.59-2.56 (2H, m, THN), 2.39 (2H, brt, J = 7.8 Hz, ArCH_2), 2.33-2.25 (2H, m, H2+H4), 2.24 (3H, s, Mes-CH_3), 2.22-15 (2H, m, H2'+H4'), 1.97-1.88 (1H, m, H3), 1.86-1.79 (2H, m, THN), 1.76 (2H, dt, J = 15.3, 7.5 Hz, ArCH$_2$CH_2); ^{13}C NMR (101 MHz, CDCl$_3$) δ 175.0 (C), 174.0 (C), 156.2 (C), 155.2 (C), 143.3 (C), 139.6 (C), 137.4 (CH), 132.4 (CH), 132.2 (C), 113.4 (C), 111.0 (CH),

51.2 (CH), 42.8 (CH), 41.6 (CH_2), 37.0 (CH_2), 36.3 (CH_2), 32.5 (CH_2), 32.2 (CH_2), 28.9 (CH), 26.3 (CH_2), 23.0 (CH_3), 21.3 (CH_2), 21.1 (CH_3); *m/z* (ES+) 511.2 ([M+H]+ 100%); HRMS Found ([M+H]+) 511.2367. $C_{27}H_{35}O_4N_4S$ req. 511.2374.

(*S*)-4-(*tert*-butoxy)-4-oxo-2-(phenylsulfonamido)butanoic acid [Compound 36]: $NaHCO_3$ (105 mg, 1.25 mmol) was added to a stirred suspension of (*S*)-Asp(O^{t}Bu)-OH (189 mg, 1 mmol) in dioxane:water (1:1, 6 mL) and stirred at RT for 45 minutes. Phenylsulfonyl chloride (0.108 mL, 150 mg, 0.85 mmol) was added and the reaction mixture stirred for a further 28 hours. The dioxane was removed *in vacuo*, the aqueous layer was acidified with 3 M HCl, extracted with DCM (4 × 20 mL), dried ($MgSO_4$) and concentrated *in vacuo*. The crude residue was purified *via* PTLC (95:5, DCM:MeOH) to yield **36** as a white foam (131 mg, 0.40 mmol, 40%); R_f 0.26 (95:5, DCM:MeOH); $[\alpha]_D$ +0.330 (c 0.63, $CHCl_3$); ^{1}H NMR (400 MHz, $CDCl_3$) δ 7.80 (2H, d, *J* = 7.4 Hz, Ph), 7.51 (1H, t, *J* = 7.3 Hz, Ph), 7.43 (2H, t, *J* = 7.5 Hz, Ph), 5.74 (1H, d, *J* = 8.4 Hz, N*H*), 4.09 (1H, dt, *J* = 8.4, 5.0 Hz, C*H*), 2.82 (1H, dd, *J* = 17.1, 5.0 Hz, C*H*H'), 2.67 (1H, dd, *J* = 17.1, 5.0 Hz, CH*H*'), 1.34 (9H,s, ^{t}Bu); ^{13}C NMR (101 MHz, $CDCl_3$) δ 174.25 (C), 169.76 (C), 139.69 (C), 133.00 (CH), 129.15 (CH), 127.14 (CH), 82.56 (C), 52.02 (CH), 38.76 (CH_2), 27.97 (CH_3); *m*/*z* (ES-) 328.1 ([M-H]-, 100%); HRMS found ([M+NH_4]+) 347.1270 $C_{14}H_{23}O_6N_2S$ req. 347.1271.

tert-butyl (S)-4-(((1*r,3*R**)-3-(2-(1,8-naphthyridin-2-yl)ethyl)cyclobutyl)amino)-4-oxo-3-(phenylsulfonamido)butanoate [Compound 37]:** EDCI (172 mg, 0.9 mmol), HOBt (122 mg, 0.9 mmol) and DIPEA (0.26 mL, 194 mg, 1.5 mmol) were added to a stirred solution of (1*r**,3*s**)-3-(2-(1,8-naphthyridin-2-yl)ethyl)cyclobutan-1-amine **25** (68 mg, 0.3 mmol) and (*S*)-4-(*tert*-butoxy)-4-oxo-2-(phenylsulfonamido)butanoic acid **36** (100 mg, 0.3 mmol) in DCM (8 mL) and stirred at RT, under a blanket of N_2 for 22 hours 30 minutes. The reaction

mixture was diluted with water (15 mL) and extracted with DCM (3 × 15 mL). The combined organic layers were washed with saturated aqueous ammonium chloride (10 mL), dried ($MgSO_4$) and concentrated *in vacuo*. The crude residue was purified *via* PTLC (97:3, DCM:MeOH) to yield **37** as a yellow oil (80 mg, 0.15 mmol, 50%); R_f 0.45 (95:5, DCM:MeOH); $[\alpha]_D$ +0.21 (c 1.0, $CHCl_3$); IR ν_{max}/cm^{-1} 2973.2 (CH), 1724.8 (C=O ester), 1655.3 (C=O amide); ^1H NMR (400 MHz, $CDCl_3$) δ 9.03 (1H, dd, *J* = 4.2, 2.0 Hz, NapthH7), 8.10 (1H, dd, *J* = 8.1, 2.0 Hz, NapthH5), 8.04 (1H, d, *J* = 8.2 Hz, Ar-H4), 7.79 (2H, d, *J* = 7.4 Hz, Ph), 7.53 (1H, tt, *J* = 7.4, 2.0 Hz, Ph), 7.45 (2H, t, *J* = 7.4 Hz, Ph), 7.39 (1H, dd, *J* = 8.1, 4.2 Hz, NapthH6), 7.30 (1H, d, *J* = 8.1 Hz, NapthH3), 6.59 (1H, brd, *J* = 7.9 Hz, N*H*), 6.23 (1H, d, *J* = 8.7 Hz, N*H*), 3.96 (1H, dt, *J* = 8.7, 8.0 Hz, NHC*H*), 3.91 (1H, ddd, J = 8.6, 6.6, 4.0 Hz), 2.89 (2H, t, *J* = 7.3 Hz, NapthCH_2), 2.78 (1H, dd, *J* = 17.2, 4.0 Hz), 2.37-2.27 (2H, m), 2.19 (1H, dd, *J* = 17.2, 6.6 Hz), 2.02-1.86 (4H, m), 1.53-1.25 (1H, m), 1.32 (9H, s, ^{t}Bu); ^{13}C NMR (101 MHz, $CDCl_3$) δ 171.12 (C), 168.29 (C), 166.33 (C), 155.91 (C), 153.37 (CH), 139.78 (C), 137.06 (CH), 136.82 (CH), 133.16 (CH), 129.40 (CH), 127.06 (CH), 122.60 (CH), 121.54 (CH), 121.06 (C), 82.30 (C), 52.94 (CH), 41.30 (CH), 37.17 (CH_2), 36.92 (CH_2), 36.70 (CH_2), 36.02 (CH_2), 28.62 (CH), 27.96 (CH_3); *m/z* (AP+) 228.0 (100%), 539.3 ([M+H]+, 20%); HRMS Found [M+H]+ 539.2320. $C_{28}H_{35}O_5N_4S$ req. 539.2323; LCMS rt 7.30, *m/z* (ES+) 539.4 ([M+H]+, 100%), >95%.

tert-butyl (*S*)-4-oxo-3-(phenylsulfonamido)-4-(((1*r,3*R**)-3-(2-(5,6,7,8-tetrahydro-1,8-naphthyridin-2-yl)ethyl)cyclobutyl)amino)butanoate [Compound 39]** was prepared from **37** (56 mg, 0.10 mmol) using the general procedure for tetrahydronapthyridine synthesis to yield **39** as a clear oil (48 mg, 0.089 mmol, 89%); R_f 0.42 (95:5, DCM:MeOH); $[\alpha]_D$ +0.177 (c 0.96, $CHCl_3$); IR ν_{max}/cm^{-1} 3287.2 (NH), 2927.7 (CH), 1725.1 (C=O ester), 1654.7 (C=O amide); ^1H NMR (400 MHz, $CDCl_3$) δ 7.79 (2H, d, *J* = 7.6 Hz, Ph), 7.54 (1H, t, *J* = 7.6 Hz,

Ph), 7.46 (2H, t, J = 7.6 Hz, Ph), 7.00 (1H, d, J = 7.1 Hz, Ar), 6.50 (1H, brd, J = 7.8 Hz, N*H*), 6.23 (1H, d, J = 7.1 Hz, Ar), 4.95 (1H, brs, N*H*), 3.94 (1H, tt, J = 8.7, 8.0 Hz), 3.90-3.87 (1H, m), 3.37-3.30 (2H, m), 2.79 (1H, dd, J = 17.3, 3.9 Hz), 2.62 (2H, t, J = 6.2 Hz), 2.36 (2H, appt, J = 7.8 Hz), 2.33-2.26 (2H, m), 2.15 (1H, dd, J = 17.3, 6.7 Hz), 1.88-1.79 (3H, m), 1.64 (2H, q, J = 7.6 Hz), 1.31 (9H, s, ^{t}Bu), 1.29-1.15 (3H, m); ^{13}C NMR (101 MHz, $CDCl_3$) δ 171.18 (C), 168.20 (C), 157.36 (C), 155.46 (C), 139.74 (C), 136.91 (CH), 133.18 (CH), 129.42 (CH), 127.06 (CH), 113.57 (C), 111.12 (CH), 82.30 (C), 53.47 (CH_2), 52.86 (CH), 41.58 (CH_2), 41.37 (CH), 37.02 (CH_2), 36.81 (CH_2), 35.24 (CH_2), 28.52 (CH), 27.96 (CH_3), 26.30 (CH_2), 21.36 (CH_2); *m/z* (AP+) 543.3 ([M+H]+, 100%); HRMS Found ([M+H]+) 543.2629. $C_{28}H_{39}O_5N_4S$ req. 543.2636; LCMS rt 2.94, *m/z* (ES+) 543.4 ([M+H]+, 100%), >95%.

(*S*)-4-(((1r*,3R*)-3-(2-(1,8-naphthyridin-2-yl)ethyl)cyclobutyl)amino)-4-oxo-3-(phenylsulfonamido)butanoic acid [Compound 38]: *tert*-butyl (*S*)-4-(((1*r**,3*R**)-3-(2-(1,8-naphthyridin-2-yl)ethyl)cyclobutyl)amino)-4-oxo-3-(phenylsulfonamido)butanoate **37** (21 mg, 0.039 mmol) was dissolved in DCM (2 mL) and TFA (0.3 mL) and the reaction mixture stirred for 24 hours. The reaction mixture was concentrated *in vacuo* to yield **38** (quant) as a yellow solid; R_f 0.24 (95:5, DCM:MeOH); $[\alpha]_D$ +0.24 (c 1.12, $CHCl_3$); IR ν_{max}/cm^{-1} 3261.9 (NH), 2919.7 (CH), 1731.1 (C=O acid), 1637.3 (C=O amide); ^{1}H NMR (400 MHz, MeOD) δ 9.16 (1H, dd, J = 4.9, 1.5 Hz, NapthH7), 8.87 (1H, dd, J = 8.4, 1.5 Hz, NapthH5), 8.81 (1H, d, J = 8.4 Hz, NapthH4), 7.92 (1H, dd, J = 8.3, 4.9 Hz, NapthH6), 7.90 (1H, dd, J = 8.3 Hz, NapthH3), 7.75 (2H, dd, J = 7.3, 1.5 Hz, Ph), 7.50 (1H, tt, J = 7.3, 1.5 Hz, Ph), 7.43 (2H, tt, J = 7.3, 1.5 Hz, Ph), 3.99 (1H, t, J = 6.6 Hz,), 3.77 (1H, tt, J = 8.8, 7.8 Hz), 3.09-3.02 (2H, m, NapthCH_2), 2.45 (1H, dd, J = 16.4, 6.7 Hz), 2.35 (1H, dd, J = 16.4, 6.7 Hz), 2.30-2.15 (2H, m), 1.98-1.85 (3H, m), 1.49-1.32 (2H, m), 1.30-1.19 (2H, m); ^{13}C NMR (101 MHz, MeOD) δ 153.5 (CH), 144.2 (CH), 144.1 (CH), 133.9 (CH), 130.2 (CH), 128.2 (CH), 126.3 (CH), 125.0 (CH),

124.4 (C), 54.6 (CH), 42.4 (CH), 37.9 (CH_2), 37.1 (CH_2), 37.0 (CH_2), 36.9 (CH_2), 35.8 (CH_2), 29.7 (CH) (not all carbons could be detected as the sample began to react with the solvent at longer experiment times); *m*/*z* (ES+) 483.3 ([M+H]+, 100%). HRMS was not obtained as the acid began reacting with MeOD to form methyl esters *in situ*; LCMS rt 2.92, *m*/*z* (ES+) 483.3 ([M+H]+, 100%), >95%.

(*S*)-4-oxo-3-(phenylsulfonamido)-4-(((1r*,3R*)-3-(2-(5,6,7,8-tetrahydro-1,8-naphthyridin-2-yl)ethyl)cyclobutyl)amino)butanoic acid [Compound 40] was prepared from **39** (13 mg, 0.024 mmol) according to the method described for **38** above to yield **40** (quant) as a yellow solid (16 mg, 0.039 mmol, 100%); R_f 0.10 (95:5, DCM:MeOH); $[\alpha]_D$ +0.102 (c 1.00, $CHCl_3$); IR ν_{max}/cm^{-1} 3253.5 (NH), 2931.9 (CH), 1725.0 (C=O ester), 1636.4 (C=O amide); ^1H NMR (400 MHz, MeOD) δ 7.77 (2H, dd, *J* = 8.6, 1.5 Hz, Ph), 7.58-7.40 (5H, m, Ph+Ar), 6.51 (1H, d, *J* = 7.3 Hz, Ar), 4.01 (1H, t, *J* = 6.6 Hz,), 3.78 (1H, tt, *J* = 8.6, 7.6 Hz), 3.45-3.34 (2H, m, ArCH_2), 2.78-2.67 (2H, m), 2.55-2.48 (2H, m), 2.45 (1H, dd, *J* = 16.5, 6.6 Hz), 2.37 (1H, dd, *J* = 16.5, 6.6 Hz), 2.31-2.18 (2H, m), 1.91-1.81 (4H, m), 1.71-1.65 (2H, m), 1.44-1.34 (2H, m); ^{13}C NMR (101 MHz, MeOD) δ 173.1 (C), 171.5 (C), 149.6 (C), 142.9 (CH), 142.0 (C), 133.9 (CH), 130.2 (CH), 128.2 (CH), 120.6 (C), 111.8 (CH), 54.6 (CH), 42.4 (CH), 42.3 (CH_2), 38.0 (CH_2), 36.9 (CH_2), 36.8 (CH_2), 31.6 (CH_2), 29.4 (CH), 28.3 (CH_2), 26.4 (CH_2), 20.5 (CH_2); *m*/*z* (ES+) 487.3 ([M+H]+, 100%). HRMS was not obtained as the acid began reacting with MeOD to form methyl esters *in situ*; LCMS rt 2.01, *m*/*z* (ES+) 487.3 ([M+H]+, 100%), >95%.

***Tert*-butyl (6-bromopyridin-2-yl)(methyl)carbamate [Compound 45]:** Triethylamine (5.75 mL, 4.17 g, 38.01 mmol) and DMAP (775 mg; 6.35 mmol) were added to a stirred suspension of 2-amino-6-bromopyridine **44** (5.49 g, 31.73 mmol) in DCM (46 mL). A solution of Boc_2O

(8.31 g; 38.0 mmol) in DCM (14 mL) was then added and the reaction mixture stirred at RT for 24 hours. The reaction mixture was concentrated *in vacuo* and purified by flash chromatography column (EtOAc:PE, 0:1→1:9) to yield the intermediate *tert*-butyl (6-bromopyridin-2-yl)carbamate as a white powder (6.71 g, 24.58 mmol, 78%). This intermediate was dissolved in anhydrous DMF (25 mL) and added slowly to a stirred suspension of NaH (60% dispersion in oil, 2.06 g, 51.62 mmol) in anhydrous DMF (50 mL) at 0°C. The mixture was stirred for 15 minutes, then allowed to warm to RT and stirred for 1 hour. MeI (3.44 mL, 7.85 g, 55.30 mmol) was added and the reaction mixture stirred for 18 hours. The reaction mixture quenched with water (150 mL) and extracted with EtOAc (3 × 50 mL). The combined organic layers were dried ($MgSO_4$), concentrated *in vacuo*, and the residue purified by chromatography column (EtOAc:PE 1:9) to yield **45** as a yellow oil (5.87 g, 83%); ^1H NMR (400 MHz, $CDCl_3$) δ 7.49 (1H, dd, *J* = 8.1, 7.6 Hz, ArH-4), 7.37 (1H, d, *J* = 8.1 Hz, Ar, ArH-3), 6.85 (1H, d, *J* = 7.6 Hz, Ar, ArH-5), 3.38 (3H, s, NCH_3), 1.50 (9H, s, tBu). Identical to literature.[3]

***tert*-butyl (6-(4-hydroxy-but-1-yn-1-yl)-pyridin-2-yl)(methyl)carbamate [Compound 46]:** 3-butyn-1-ol (1.91 mL, 1.62 g, 22.50 mmol), $Pd(PPh_3)_2Cl_2$ (287 mg, 0.41 mmol) and CuI (78 mg, 0.41 mmol) were added to a stirred solution of *tert*-butyl (6-bromopyridin-2-yl)(methyl)carbamate **45** (5.87 g, 20.45 mmol) in diethylamine (59 mL) and the reaction mixture heated to 70 °C for 22 h. The reaction mixture was concentrated *in vacuo* and purified by flash column chromatography (EtOAc:PE, 3:7 →1:1) to yield **46** (5.11 g, 91%) as a yellow oil; R_f 0.20 (EtOAc:PE, 3:7); ^1H (400 MHz, $CDCl_3$) δ 7.59-7.52 (2H, m, ArH-4,3), 7.11 (1H, dd, *J* = 1.5, 6.5 Hz, ArH-5), 3.83 (2H, t, *J* = 6.4 Hz, CH_2OH), 3.37 (3H, s, NCH_3), 2.70 (2H, t, *J* = 6.4 Hz, CH_2CH_2OH), 2.37 (1H, brs, O*H*), 1.49 (9H, s, tBu); ^{13}C (100 MHz, $CDCl_3$) 171.2 (C), 171.1 (C), 155.2 (C), 154.3 (C), 140.9 (C), 137.1 (CH), 122.8 (CH), 119.0 (CH), 86.9 (C),

81.8 (C), 81.3 (C), 60.8 (CH_2), 34.4 (CH_3), 28.3 (CH_2), 14.2 (CH_3); m/z (AP+) 277 [M+H]+, 100%); HRMS found 277.1545. $C_{15}H_{21}O_3N_2$ req. 277.1547.

***tert*-butyl methyl(6-(4-oxobutyl)pyridin-2-yl)carbamate [Compound 43]:** PtO_2 (336 mg) was added to a stirred solution of *tert*-butyl (6-(4-hydroxy-but-1-yn-1-yl)-pyridin-2-yl)(methyl)carbamate **46** (5.11g, 18.58 mmol) in EtOH (200 mL) and the reaction mixture stirred under 1 atm H_2 for 21.5 h, then filtered through 3 cm silica and concentrated *in vacuo* to yield tert-butyl (6-(4-hydroxybutyl)pyridine-2yl)(methyl)carbamate (4.77 g, 17.10 mmol, 92%) as a grey oil; R_f 0.15 (EtOAc:PE, 3:7); ^{1}H (400 MHz, $CDCl_3$) 7.52 (1H, t, J = 7.5 Hz, ArH-4), 7.41 (1H, d, J = 7.5 Hz, ArH-3), 6.84 (1H, d, J = 7.5 Hz, ArH-5), 3.68 (2H, t, J = 7.5 Hz, CH_2OH), 3.37 (3H, s, NCH_3), 2.76 (2H, t, J = 7.5 Hz, PyrCH_2), 1.81 (2H, qn, J = 7.5 Hz), 1.70 (1H, brs, O*H*), 1.63 (2H, qn, J = 7.5 Hz), 1.50 (9H, s, tBu). Identical to literature data.[4] This was dissolved in DCM (157 mL), TEMPO (400 mg, 2.56 mmol) and BAIB (6.06 g, 18.81 mmol) added and the reaction mixture stirred at RT. 3 further portions of TEMPO (400 mg, 2.56 mmol) were added at 2.5 hourly intervals then the reaction mixture stirred at room temperature overnight. The reaction mixture was washed with saturated aqueous sodium thiosulfate solution (100 mL), dried ($MgSO_4$) and concentrated *in vacuo*. The residue was purified by flash column chromatography (EtOAc:PE, 15:75) to yield **43** as a yellow oil (3.66 g, 77%). ^{1}H NMR (400 MHz, $CDCl_3$) δ 9.71 (1H, t, J = 1.5 Hz, C*H*O), 7.49-7.36 (2H, m, Py-H3+H4), 6.76 (1H, d, J = 7.1 Hz, Py-H5), 3.31 (3H, s, NCH_3), 2.69 (2H, t, J = 7.5 Hz, PyrCH_2), 2.43 (2H, td, J = 7.5, 1.5 Hz, CH_2COH), 2.01 (2H, dt, J = 15.1, 7.5 Hz, CH_2), 1.44 (9H, s, ^{t}Bu); ^{13}C NMR (101 MHz, $CDCl_3$) δ 202.4 (CH), 158.8 (C), 154.7 (C), 154.5 (C), 137.2 (CH), 118.1 (CH), 116.6 (CH), 81.0 (C), 43.2 (CH_2), 36.8 (CH_2), 34.2 (CH_3), 28.3 (CH_3), 21.6 (CH_2); *m/z* (AP+) 278.9 ([M+H]+, 60%), 223.0 (100%); HRMS found ([M+H]+) 279.1711. $C_{15}H_{23}O_3N_2$ req. 279.1703.

Methyl 3-(2-(6-((*tert*-butoxycarbonyl)(methyl)amino)pyridin-2-yl)ethyl)cyclobut-1-ene-1-carboxylate [Compound 47]: Diethylamine (2.73 mL, 1.93 g, 26.43 mmol) was added to a stirred solution of *tert*-butyl methyl (6-(4-oxobutyl)pyridin-2-yl)carbamate **43** (3.66 g, 13.21 mmol) and K_2CO_3 (3.65 g, 26.43 mmol) in MeCN (70 mL) and the reaction mixture stirred at RT for 3 h. Methyl acrylate (3.0 mL, 2.27 g, 26.43 mmol) was added and the reaction mixture stirred for 69 hours. The reaction mixture was filtered through Celite® and concentrated *in vacuo*. The residue was dissolved in MeCN (78 mL) and MeI (4.1 mL, 9.38 g, 66.1 mmol) was added and the reaction mixture stirred at RT for 24 hours. The reaction mixture was concentrated *in vacuo* and the residue redissolved in $CHCl_3$ (78 mL). DBU (1.98 mL, 2.01 g, 13.21 mmol) was added and the reaction mixture heated to reflux for 22 hours. The reaction mixture was concentrated *in vacuo* and the residue was purified *via* column chromatography (EtOAc:PE , 15:85) to yield **47** as a yellow oil (2.88 g, 63%); R_f 0.49 (3:7, EtOAc:PE); ^1H NMR (400 MHz, $CDCl_3$) δ 7.45 (1H, dd, J = 8.1, 7.2 Hz, Py-H4), 7.37 (1H, d, J = 8.1 Hz, Py-H3), 6.76 (1H, d, J = 7.2 Hz, Py-H5), 6.75 (1H, d, J = 1.0 Hz, H2), 3.66 (3H, s, OCH_3), 3.31 (3H, s, NCH_3), 2.76 (1H, dd, J = 13.2, 4.3 Hz, H4), 2.71-2.69 (1H, m, H3), 2.70 (2H, t, J = 7.7 Hz, PyCH_2), 2.21 (1H, dd, J = 13.2, 1.0 Hz, H4'), 1.91-1.79 (2H, m, PyCH$_2$CH_2), 1.44 (9H,s, ^{t}Bu); ^{13}C NMR (101 MHz, $CDCl_3$) δ 172.0 (C), 163.1 (C), 159.5 (C), 154.7 (C), 154.5 (C), 150.0 (CH), 137.2 (CH), 118.0 (CH), 116.5 (CH), 80.9 (C), 51.3 (CH_3), 39.7 (CH), 36.2 (CH_2), 34.7 (CH_2), 34.3 (CH_3), 32.9 (CH_2), 28.3 (CH_3); *m/z* (AP+) 347.2 ([M+H]+ 100%); HRMS found (M+) 346.1885. $C_{19}H_{26}O_4N_2$ req. 346.1887.

Methyl (1*r,3*s**)-3-(2-(6-((tert-butoxycarbonyl)(methyl)amino)pyridin-2-yl)ethyl) cyclobutane-1-carboxylate [Compound 48]:** 10% Pd/C (0.29 g) was added to a solution of methyl 3-(2-(6-((tert-butoxycarbonyl)(methyl)amino)pyridin-2-yl)ethyl)cyclobut-1-ene-1-

carboxylate **47** (2.88 g, 8.32 mmol) in EtOAc (250 mL) and the reaction mixture stirred under 1 atm H_2 for 23 hours. The reaction mixture was filtered through Celite® and concentrated *in vacuo* to yield **48** as a clear oil (2.67g, 92%); ^{1}H NMR (400 MHz, $CDCl_3$) δ 7.50 (1H, dd, *J* = 8.1, 7.1 Hz, Py-H4), 7.40 (1H, d, *J* = 8.1Hz, Py-H3), 6.81 (1H, d, *J* = 7.1 Hz, Py-H5), 3.65 (3H, s, OCH_3), 3.38 (3H, s, NCH_3), 2.95 (1H, tt, *J* = 9.6, 8.1 Hz, H1), 2.62 (2H, t, *J* = 7.7 Hz, $PyCH_2$), 2.32-2.18 (3H, m, $PyCH_2CH_2$+H3), 1.93-1.85 (2H, m, H2,4), 1.83-1.78 (2H, m, H2,4), 1.50 (9H, s, ^{t}Bu); ^{13}C NMR (101 MHz, $CDCl_3$) δ 175.8 (C), 159.9 (C), 154.4 (C), 151.4 (C), 137.1 (CH), 117.9 (CH), 116.4 (CH), 80.7 (C), 51.6 (CH_3), 36.3 (CH_2), 35.4 (CH_2), 34.3 (CH_3), 34.3 (CH), 31.5 (CH_2), 31.4 (CH), 28.3 (CH_3); *m/z* (AP+) 349.2 ([M+H]+ 100%); HRMS found ([M+H]+) 349.2118. $C_{19}H_{29}O_4N_2$ req. 349.2122.

Methyl (*S*)-3-((1*r,3*R**)-3-(2-(6-((tert-butoxycarbonyl)(methyl)amino)pyridin-2-yl)ethyl)cyclobutane-1-carboxamido)-2-(phenylsulfonamido)propanoate [Compound 51]:** NaOH (1 M in water, 0.55 mL, 0.55 mmol) was added to a stirred solution of methyl (1*r**,3*s**)-3-(2-(6-((tert-butoxycarbonyl)(methyl)amino)pyridin-2-yl)ethyl)cyclobutane-1-carboxylate **48** (40 mg, 0.11 mmol) in MeOH (5 mL) and the reaction mixture heated at reflux for 5 hours. The mixture was allowed to cool, quenched with HCl (4 M in dioxane, 0.14 mL, 0.55 mmol), diluted with EtOAc (5 mL), dried ($MgSO_4$) and concentrated *in vacuo* to give the crude acid. The acid was dissolved in DMF (3 mL) and methyl (*S*)-3-amino-2-(phenylsulfonamido)propanoate **49**[2] (28 mg, 0.11 mmol), EDCI (63 mg, 0.33 mmol), HOBt (45 mg, 0.33 mmol) and DIPEA (0.096 mL, 71 mg, 0.55 mmol) were added and the reaction mixture stirred at RT under a blanket of N_2 for 18 hours. The reaction mixture was diluted with H_2O (10 mL), extracted with EtOAc (3 × 15 mL) and the combined organic layers washed with H_2O (2 × 10 mL), followed by saturated aqueous ammonium chloride (10 mL), dried ($MgSO_4$) and concentrated *in vacuo*. The crude residue was purified *via* PTLC (EtOAc) to yield **51** as a

clear oil (19 mg, 0.033 mmol, 30%); R_f 0.55 (EtOAc); $[\alpha]_D$ +0.28 (c 0.94, $CHCl_3$); ^{1}H NMR (400 MHz, $CDCl_3$) δ 7.83 (2H, dd, J = 7.1, 2.0 Hz, , Ph), 7.58 (1H, tt, *J* = 7.6, 2.0 Hz, Ph), 7.52-7.48 (3H, m, Ph+Py-H4), 7.40 (1H, d, *J* = 8.2 Hz, Py-H3), 6.80 (1H, d, *J* = 7.3 Hz, Py-H5), 5.92 (1H, t, *J* = 6.0 Hz, N*H*), 5.79 (1H, d, *J* = 7.1 Hz, N*H*), 3.97 (1H, td, *J* = 6.6, 4.0 Hz, C*H*), 3.61 (2H, ddd, *J* = 14.1, 6.0. 4.0 Hz), 3.56 (3H, s, OCH_3), 3.50-3.48 (1H, dd, *J* = 14.1, 6.6 Hz), 3.37 (3H, s, NCH_3), 2.77 (1H, tt, *J* = 9.6, 8.6 Hz, H1), 2.61 (2H, brt, *J* = 7.6 Hz, PyCH_2), 2.27-2.12 (3H, m, H2+H3+H4), 1.89-1.76 (4H m, PyCH_2CH_2+H2'+H4'), 1.49 (9H, s, ^{t}Bu); ^{13}C NMR (101 MHz, $CDCl_3$) δ 175.7 (C), 170.0 (C), 159.9 (C), 154.6 (C), 154.5 (C), 139.2 (C), 137.1 (CH), 133.1 (CH), 129.2 (CH), 127.2 (CH), 117.9 (CH), 116.4 (CH), 80.8 (C), 55.8 (CH), 53.1 (CH_3), 41.7 (CH_2), 36.2 (CH_2), 36.0 (CH), 35.3 (CH_2), 34.3 (CH_3), 31.4 (CH_2), 31.3 (CH_2), 31.0 (CH), 28.3 (CH_3); *m/z* (AP+) 575.2 ([M+H]+ 10%), 475.1 ([M-Boc]+ 100%); HRMS found ([M+H]+) 575.2523. $C_{28}H_{39}O_7N_4S$ req. 575.2534.

Methyl (*S*)-3-((1*r,3*R**)-3-(2-(6-((*tert*-butoxycarbonyl)(methyl)amino)pyridin-2-yl)ethyl)cyclobutane-1-carboxamido)-2-(2,4,6-trimethylphenylsulfonamido)propanoate [Compound 52]** was prepared according to the same procedure using **48** (490 mg, 1.41 mmol) and **50**[2] (461 mg, 1.55 mmol) to yield **2** as a brown oil (411 mg, 47%); R_f 0.48 (3:1, EtOAc:PE); $[\alpha]_D$ +0.28 (c 1.03, $CHCl_3$); ^{1}H NMR (400 MHz, $CDCl_3$) δ 7.44 (1H, dd, *J* = 8.1, 7.1 Hz, Py-H4), 7.34 (1H, d, *J* = 8.1 Hz, Py-H3), 6.88 (2H, s, Mes-H3+H5), 6.75 (1H, d, *J* = 7.1 Hz, Py-H5), 5.86 (1H, t, *J* = 6.1 Hz, N*H*), 5.73 (1H, d, *J* = 6.8 Hz, N*H*), 3.79 (1H, td, *J* = 6.8, 4.1 Hz, C*H*), 3.55 (1H, *J* = 13.6, 6.8, 4.1 Hz, ddd, C*H*H'), 3.51 (3H, s, OCH_3), 3.48-3.40 (1H, dt, *J* = 13.6, 6.1 Hz, CH*H'*), 3.31 (3H, s, NCH_3), 2.70 (1H, tt, *J* = 9.6, 8.1 Hz, H-1), 2.57-2.53 (2H, m, PyCH_2), 2.55 (6H, s, Mes-CH_3), 2.21 (3H, s, Mes-CH_3), 2.20-2.09 (3H,m, H2+H3+H4), 1.84-1.68 (4H, m, PyCH_2CH_2+H2'+H4'), 1.44 (9H, s, ^{t}Bu); ^{13}C NMR (101 MHz, $CDCl_3$) δ 175.5 (C), 170.3 (C), 159.9 (C), 154.5 (C), 154.5 (C), 142.7 (C), 139.2 (C), 137.1 (CH), 133.1 (C),

132.1 (CH), 117.9 (CH), 116.4 (CH), 80.8 (C), 55.3 (CH), 53.0 (CH_3), 41.7 (CH_2), 36.2 (CH_2), 36.0 (CH), 35.3 (CH_2), 34.3 (CH_3), 31.4 (CH_2), 31.3 (CH_2), 31.1 (CH), 28.3 (CH_3), 22.9 (CH_3), 21.0 (CH_3); *m/z* (AP+) 617.0 ([M+H]+ 100%); HRMS found ([M+H]+) 617.3000. $C_{31}H_{45}O_7N_4S$ req. 617.3003.

Methyl (*S*)-3-((1*r,3*R**)-3-(2-(6-(methylamino)pyridin-2-yl)ethyl)cyclobutane-1-carboxamido)-2-(phenylsulfonamido)propanoate [Compound 53]:** TFA (0.5 mL) was added to a stirred solution of methyl (*S*)-3-((1*r**,3*R**)-3-(2-(6-((*tert*-butoxycarbonyl)(methyl)amino)pyridin-2-yl)ethyl)cyclobutane-1-carboxamido)-2-(phenylsulfonamido)propanoate **51** (19 mg, 0.033 mmol) in DCM (4 mL) and stirred for 23 hours. The reaction mixture was then concentrated *in vacuo* and the residue purified *via* PTLC (5:95, MeOH:DCM) to yield **53** as a clear oil (14.5 mg, 0.031 mmol, 94%); R_f 0.22 (95:5 DCM:MeOH); $[\alpha]_D$ +0.38 (c 0.73, $CHCl_3$); IR ν_{max}/cm^{-1} 3319.9 (NH), 3248.9 (NH), 2925.3 (CH), 1743.0 (C=O ester), 1651.5 (C=O amide); ^1H NMR (400 MHz, $CDCl_3$) δ 7.84 (2H, dt, *J* = 7.4, 1.5 Hz, Ph), 7.57 (1H, tt, *J* = 7.4, 1.5 Hz, Ph), 7.50 (2H, td, *J* = 7.4, 1.5 Hz, Ph), 7.39 (1H, dd, *J* = 8.1, 7.6 Hz, Py-H4), 6.42 (1H, d, *J* = 7.3 Hz, Py-H3), 6.22 (1H,d, *J* = 8.6 Hz, Py-H5), 6.07 (1H, brt, *J* = 5.8 Hz, N*H*), 4.99 (1H, brs, N*H*), 4.00 (1H, dd, *J* = 6.5, 4.5 Hz), 3.59 (1H, ddd, *J* = 14.1, 5.8, 4.5 Hz, C*H*H'), 3.57 (3H, s, OCH_3), 3.55 (1H, dt, *J* = 14.1, 5.8, Hz, CH*H'*), 2.88 (3H, s, NCH_3), 2.76 (1H, tt, *J* = 9.1, 8.6 Hz, H1), 2.49 (2H, t, *J* = 7.6 Hz, PyCH_2), 2.28-2.14 (3H, m, H2+H3+H4), 1.89-1.80 (2H, m, H2'+H4'), 1.76 (2H, dt, *J* = 9.1, 7.2 Hz, PyCH_2CH_2); ^{13}C NMR (101 MHz, $CDCl_3$) δ 175.8 (C), 170.2 (C), 159.9 (C), 159.1 (C), 139.5 (C), 138.4 (CH), 133.0 (CH), 129.2 (CH), 127.1 (CH), 111.4 (CH), 102.6 (CH), 55.8 (CH), 52.9 (CH_3), 41.6 (CH_2), 36.4 (CH_2), 36.0 (CH), 35.1 (CH_2), 31.4 (CH_2), 31.3 (CH_2), 31.1 (CH), 29.3 (CH_3); *m/z* (AP+) 475.2 ([M+H]+ 100%), 333.1 ([M-SO_2Ph]+ 75%); HRMS found

([M+Na]+) 497.1816. $C_{23}H_{30}O_5N_4SNa$ req. 497.1829; LCMS rt 1.83, *m/z* (ES+) 475.3 ([M+H]+, 100%), >95%.

Methyl (*S*)-3-((1r*,3R*)-3-(2-(6-(methylamino)pyridin-2-yl)ethyl)cyclobutane-1-carboxamido)-2-(2,4,6-trimethylphenylsulfonamido)propanoate [Compound 54] was prepared from **52** (393 mg, 0.64 mmol) according to the same procedure. Purification by flash column chromatography (MeOH:DCM, 5:95) gave **54** as a clear oil (300 mg, 91%); R_f 0.25 (95:5 DCM:MeOH); $[\alpha]_D$ +0.28 (c 1.27, $CHCl_3$); IR ν_{max}/cm^{-1} 3300.9 (NH), 2937.4 (CH), 1740.9 (C=O ester), 1653.5 (C=O amide); ^{1}H NMR (400 MHz, $CDCl_3$) δ 7.36 (1H, dd, *J* = 8.3, 7.3 Hz, Py-H4), 6.93 (2H, s, Mes-H3+H5), 6.40 (1H, d, *J* = 7.3 Hz, Py-H3), 6.20 (1H, d, *J* = 8.3 Hz, Py-H5), 5.99 (1H, t, *J* = 6.3 Hz, N*H*), 4.74 (1H, brs, N*H*), 3.87 (1H, dd, *J* = 6.3, 4.1 Hz, C*H*), 3.60 (1H, ddd, *J* = 13.6, 6.3, 4.1 Hz, C*H*H'), 3.56 (3H, s, OCH_3), 3.52 (1H, dt, *J* = 13.6, 6.3 Hz, CH*H'*), 2.87 (3H, d, *J* = 4.3 Hz, NCH_3), 2.75 (1H, tt, J = 9.6, 8.1 Hz, H1), 2.61 (6H, s, Mes-CH_3), 2.49 (2H, t, *J* = 7.6 Hz, PyCH_2), 2.27 (3H, s, Mes-CH_3), 2.27-2.11 (3H, m, H2+H3+H4), 1.87-1.81 (2H, m, H2'+H4'), 1.75 (2H, dt, *J* = 8.1, 7.1 Hz, PyCH$_2$CH_2); ^{13}C NMR (101 MHz, $CDCl_3$) δ 175.6 (C), 170.3 (C), 160.2 (C), 159.2 (C), 142.6 (C), 139.2 (C), 138.1 (CH), 133.2 (C), 132.0 (CH), 111.5 (CH), 102.5 (CH), 55.4 (CH), 52.9 (CH_3), 41.7 (CH_2), 36.3 (CH_2), 36.1 (CH), 35.3 (CH_2), 31.4 (CH_2), 31.3 (CH_2), 31.2 (CH), 29.3 (CH_3), 22.9 (CH_3), 20.9 (CH_3); *m/z* (AP+) 517.0 ([M+H]+ 100%); HRMS found ([M+H]+) 517.2473. $C_{26}H_{37}O_5N_4S$ req. 517.2479; LCMS rt 2.56, *m/z* (ES+) 517.4 ([M+H]+, 100%), >95%.

(*S*)-3-((1*r,3*R**)-3-(2-(6-(methylamino)pyridin-2-yl)ethyl)cyclobutane-1-carboxamido)-2-(2,4,6-trimethylphenylsulfonamido)propanoic acid [Compound 55]:** (*S*)-methyl 3-((1*r**,3*R**)-3-(2-(6-((tert-butoxycarbonyl)(methyl)amino)pyridin-2-yl)ethyl)cyclobutane-1-carboxamido)-2-(2,4,6-trimethylphenylsulfonamido)propanoate (18 mg, 0.029 mmol) **54** was

dissolved in 6 M aqueous HCl (0.5 mL) and stirred at room temperature for 20 hours. The reaction mixture was then concentrated *in vacuo* to yield **55** (quant) as a yellow oil; ^{1}H NMR (400 MHz, CD_3OD) δ 8.81 (1H, t, *J* = 7.6, 8.6 Hz, Py-H4), 7.96 (2H, s, Mes-H3+H5), 7.84 (1H, d, *J* = 8.6 Hz, Py-H3), 7.70 (1H, d, *J* = 7.6 Hz, Py-H5), 4.93 (1H, dd, *J* = 8.6, 5.1 Hz, C*H*), 4.50 (1H, dd, *J* = 13.6, 5.1 Hz, C*H*H'), 4.24 (1H, dd, *J* = 13.6, 8.6 Hz, CH*H*'), 4.10 (3H, s, CH_3), 3.80-3.89 (1H, m, H1), 3.69 (2H, brt, *J* = 8.1 Hz, PyCH_2), 3.58 (6H, s, Mes-CH_3), 3.25 (3H, s, Mes-CH_3), 3.19-3.27 (3H, m, H2+H3+H4), 2.80-2.85 (2H, m, H2'+H4'), 2.75-2.80 (2H, m, PyCH_2CH_2); ^{13}C NMR (101 MHz, CD_3OD) δ 178.1 (C), 172.7 (C), 155.8 (C), 143.6 (C), 140.6 (C), 140.5 (C), 135.5 (C), 132.9 (3CH), 112.4 (CH), 56.1 (CH/CH_3), 42.7 (CH_2), 36.7 (CH/CH_3), 36.7 (CH_2), 32.1 (CH/CH_3), 31.9 (CH_2), 31.5 (CH_2), 29.2 (CH/CH_3), 23.4 (CH/CH_3), 23.2 (CH/CH_3), 21.0 (CH/CH_3); *m/z* (ES-) 501 (100%, $[M-H]^-$); HRMS found 503.2314. $C_{25}H_{35}O_5H_4S$ req. 503.2323.

The free acid of **1** (ICT9064) was prepared by the same method, as previously described.[2]

1. O. O. Ajani, O. B. Familoni, J. O. Echeme, F. Wu and Z. Sujiang, *Am. Chem. Sci. J.*, 2012, **3**, 34-49.
2. M. Sutherland, A. Gordon, F. O. Al-Shammari, A. Throup, A. Cilia La Corte, H. Philippou, S. D. Shnyder, L. H. Patterson and H. M. Sheldrake, *Cancers*, 2023, **15**, 4023.
3. K. J. Duffy, D. Chai, M. Colon, D. M. Fitch, N. W. Johnson, S. R. Kind, A. N. Shaw, R. Tedesco, K. Wiggall and M. N. Zimmerman, 2006, WO2006/127485 A127482.
4. J. Arnould, B. Delouvrie and R. Ducray, 2007, WO2007141473 A2007141471.
