# Supplementary material for: Sidechain structure–activity relationships of cyclobutane-based small molecule αvβ3 antagonists
Source: RSC Med Chem. 2024 Sep 13;15(10):3616–24. doi: 10.1039/d4md00306c (PMC11393731; doi:10.1039/d4md00306c)
Supplement: MD-015-D4MD00306C-s002 [file MD-015-D4MD00306C-s002.pdf]

Supplementary Information (SI) for RSC Medicinal Chemistry.
This journal is © The Royal Society of Chemistry 2024

Supplementary data: spectra

Section 1 ^{1}H and ^{13}C NMR spectra of novel compounds. Pages 2-106

Section 2: HRMS data of novel compounds. Pages 107 onwards

(Mesitylsulfonyl)-L-glutamine [Compound 4]

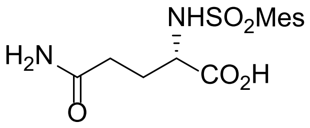

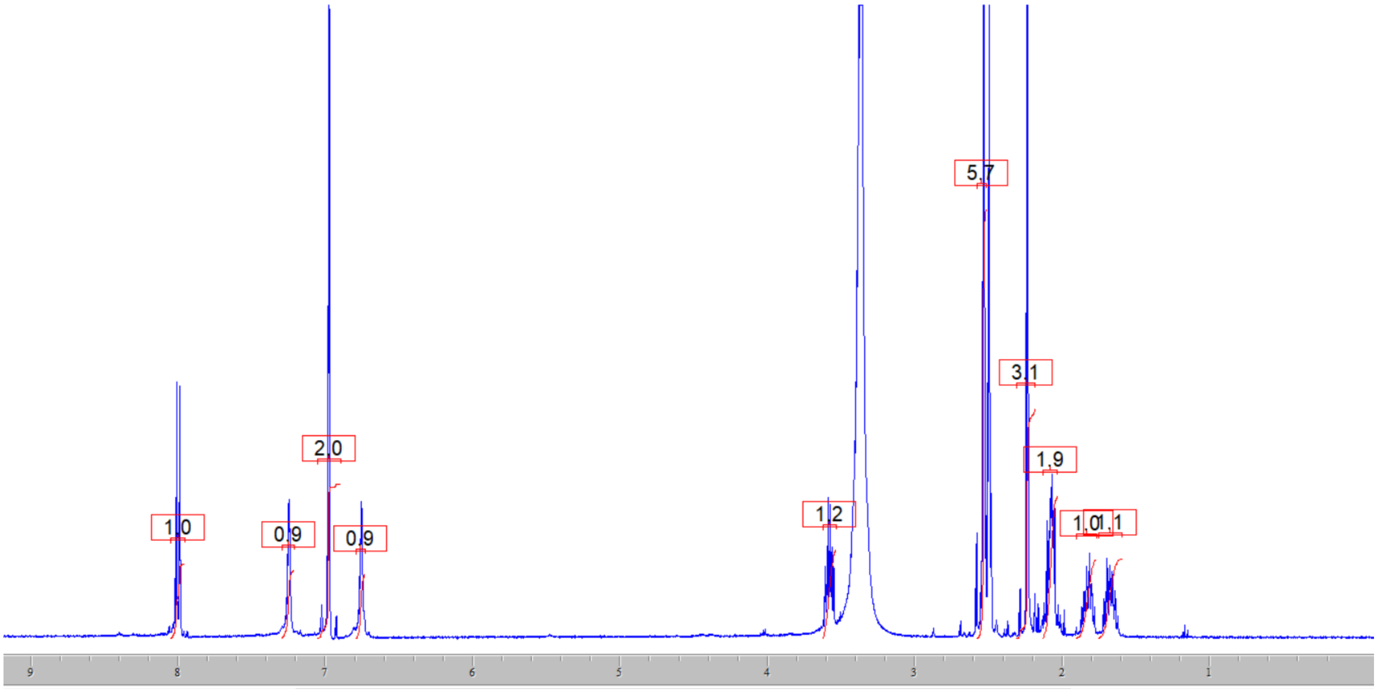

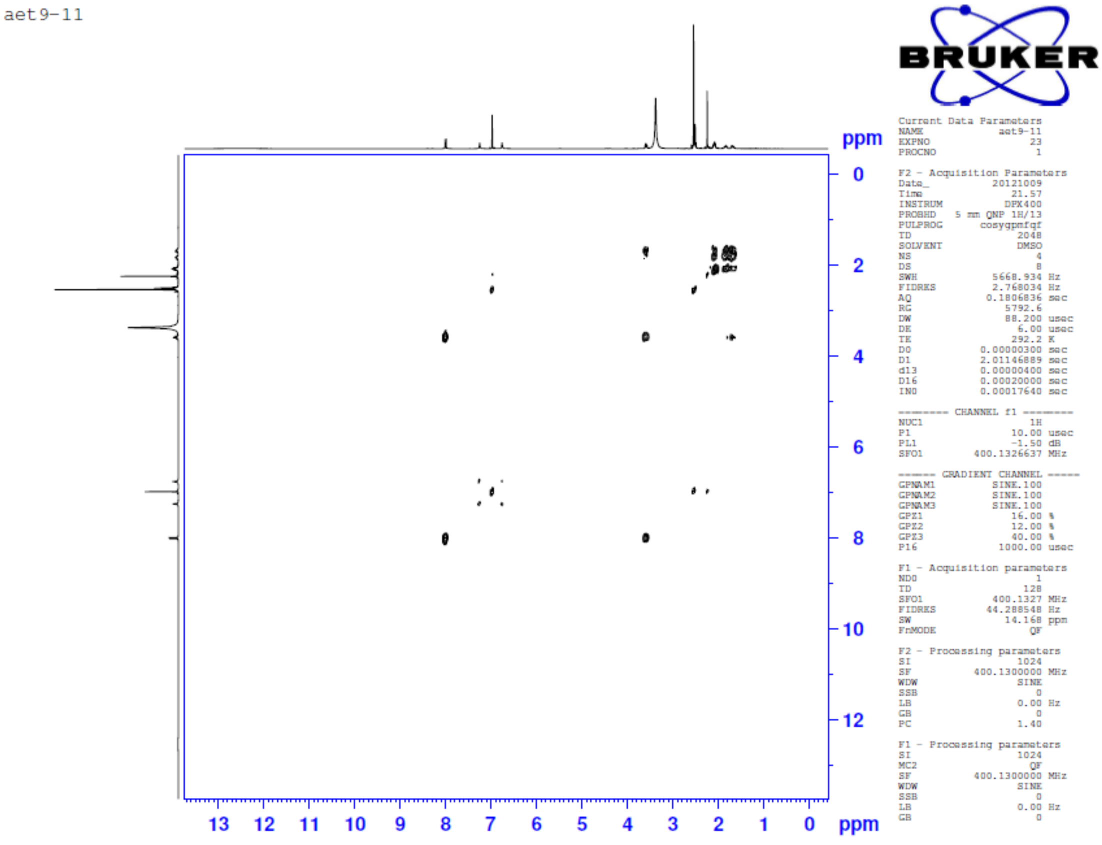

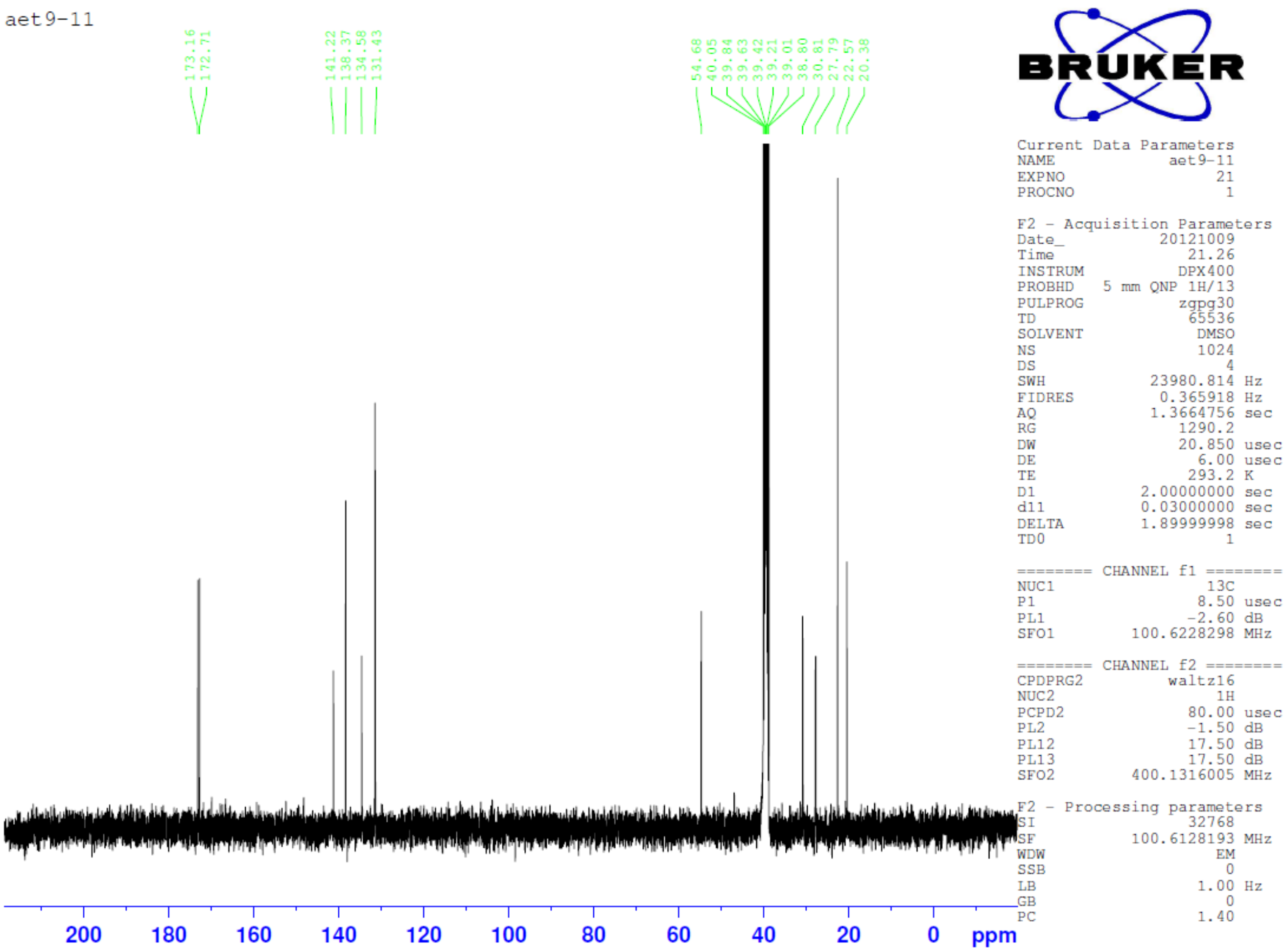
aet9-11
173.16
172.71
141.22
138.37
134.58
131.43
54.68
40.05
39.84
39.63
39.42
39.21
39.01
38.80
30.81
27.79
22.57
20.38
Current Data Parameters
NAME aet9-11
EXPNO 21
PROCNO 1
F2 - Acquisition Parameters
Date_ 20121009
Time 21.26
INSTRUM DPX400
PROBHD 5 mm QNP 1H/13
PULPROG zgpg30
TD 65536
SOLVENT DMSO
NS 1024
DS 4
SWH 23980.814 Hz
FIDRES 0.365918 Hz
AQ 1.3664756 sec
RG 1290.2
DW 20.850 usec
DE 6.00 usec
TE 293.2 K
D1 2.00000000 sec
d11 0.03000000 sec
DELTA 1.89999998 sec
TD0 1
======== CHANNEL f1 ========
NUC1 13C
P1 8.50 usec
PL1 -2.60 dB
SFO1 100.6228298 MHz
======== CHANNEL f2 ========
CPDPRG2 waltz16
NUC2 1H
PCPD2 80.00 usec
PL2 -1.50 dB
PL12 17.50 dB
PL13 17.50 dB
SFO2 400.1316005 MHz
F2 - Processing parameters
SI 32768
SF 100.6128193 MHz
WDW EM
SSB 0
LB 1.00 Hz
GB 0
PC 1.40
200
180
160
140
120
100
80
60
40
20
0
ppm

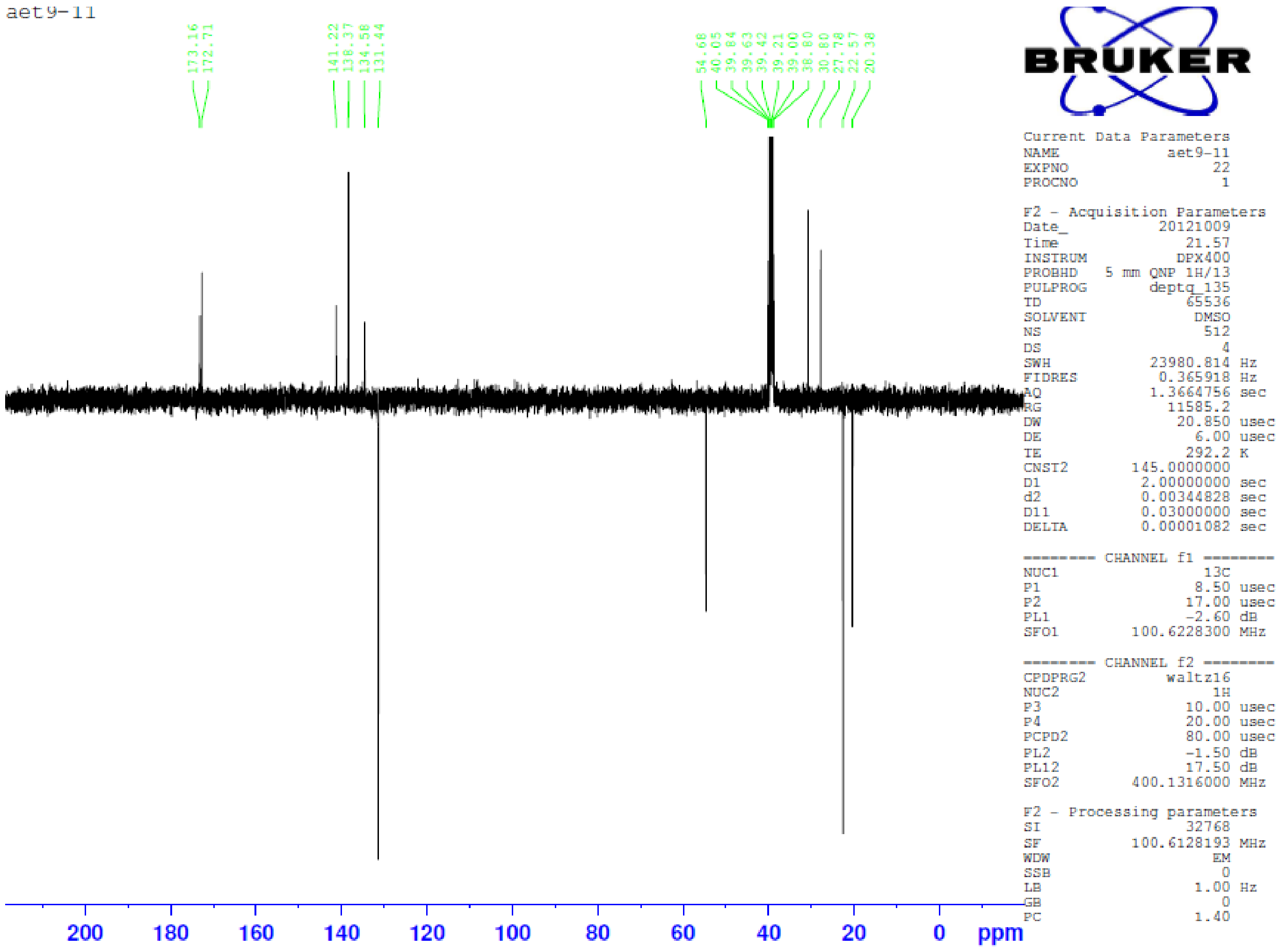
aet9-11
173.16
172.71
141.22
138.37
134.58
131.44
54.68
40.05
39.84
39.63
39.42
39.21
39.00
38.80
30.80
27.78
22.57
20.38
Current Data Parameters
NAME aet9-11
EXPNO 22
PROCNO 1
F2 - Acquisition Parameters
Date_ 20121009
Time 21.57
INSTRUM DPX400
PROBHD 5 mm QNP 1H/13
PULPROG deptq_135
TD 65536
SOLVENT DMSO
NS 512
DS 4
SWH 23980.814 Hz
FIDRES 0.365918 Hz
AQ 1.3664756 sec
RG 11585.2
DW 20.850 usec
DE 6.00 usec
TE 292.2 K
CNST2 145.0000000
D1 2.00000000 sec
d2 0.00344828 sec
D11 0.03000000 sec
DELTA 0.00001082 sec
======== CHANNEL f1 ========
NUC1 13C
P1 8.50 usec
P2 17.00 usec
PL1 -2.60 dB
SFO1 100.6228300 MHz
======== CHANNEL f2 ========
CPDPRG2 waltz16
NUC2 1H
P3 10.00 usec
P4 20.00 usec
PCPD2 80.00 usec
PL2 -1.50 dB
PL12 17.50 dB
SFO2 400.1316000 MHz
F2 - Processing parameters
SI 32768
SF 100.6128193 MHz
WDW EM
SSB 0
LB 1.00 Hz
GB 0
PC 1.40
200
180
160
140
120
100
80
60
40
20
0
ppm

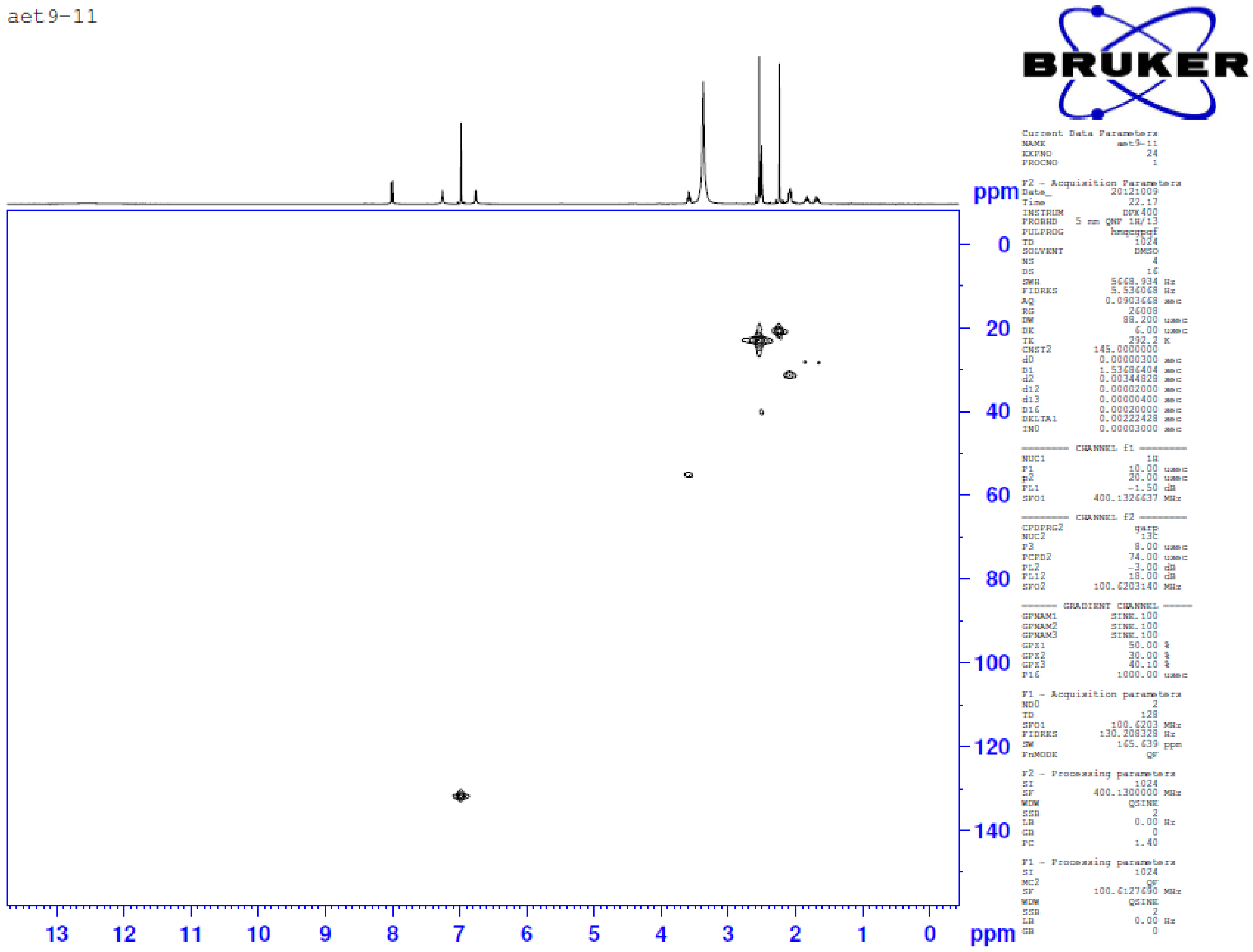
aet9-11
ppm
0
20
40
60
80
100
120
140
13
12
11
10
9
8
7
6
5
4
3
2
1
0
ppm
Current Data Parameters
NAME aet9-11
EXPNO 24
PROCNO 1
F2 - Acquisition Parameters
Date_ 20121009
Time 22.17
INSTRUM DPX400
PROBHD 5 mm QNP 1H/13
PULPROG hmqcgpqf
TD 1024
SOLVENT DMSO
NS 4
DS 16
SWH 5668.934 Hz
FIDRES 5.536068 Hz
AQ 0.0903668 sec
RG 26008
DW 88.200 usec
DE 6.00 usec
TE 292.2 K
CNST2 145.0000000
d0 0.00000300 sec
D1 1.53686404 sec
d2 0.00344828 sec
d12 0.00002000 sec
d13 0.00000400 sec
D16 0.00020000 sec
DELTA1 0.00222428 sec
IN0 0.00003000 sec
======== CHANNEL f1 ========
NUC1 1H
P1 10.00 usec
p2 20.00 usec
PL1 -1.50 dB
SFO1 400.1326637 MHz
======== CHANNEL f2 ========
CPDPRG2 garp
NUC2 13C
P3 8.00 usec
PCPD2 74.00 usec
PL2 -3.00 dB
PL12 18.00 dB
SFO2 100.6203140 MHz
====== GRADIENT CHANNEL =====
GPNAM1 SINE.100
GPNAM2 SINE.100
GPNAM3 SINE.100
GPZ1 50.00 %
GPZ2 30.00 %
GPZ3 40.10 %
P16 1000.00 usec
F1 - Acquisition parameters
ND0 2
TD 128
SFO1 100.6203 MHz
FIDRES 130.208328 Hz
SW 165.639 ppm
FnMODE QF
F2 - Processing parameters
SI 1024
SF 400.1300000 MHz
WDW QSINE
SSB 2
LB 0.00 Hz
GB 0
PC 1.40
F1 - Processing parameters
SI 1024
MC2 QF
SF 100.6127690 MHz
WDW QSINE
SSB 2
LB 0.00 Hz
GB 0

(*S*)-4-Amino-2-(phenylsulfonamido)butanoic acid 5

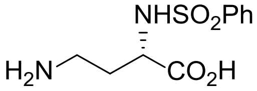

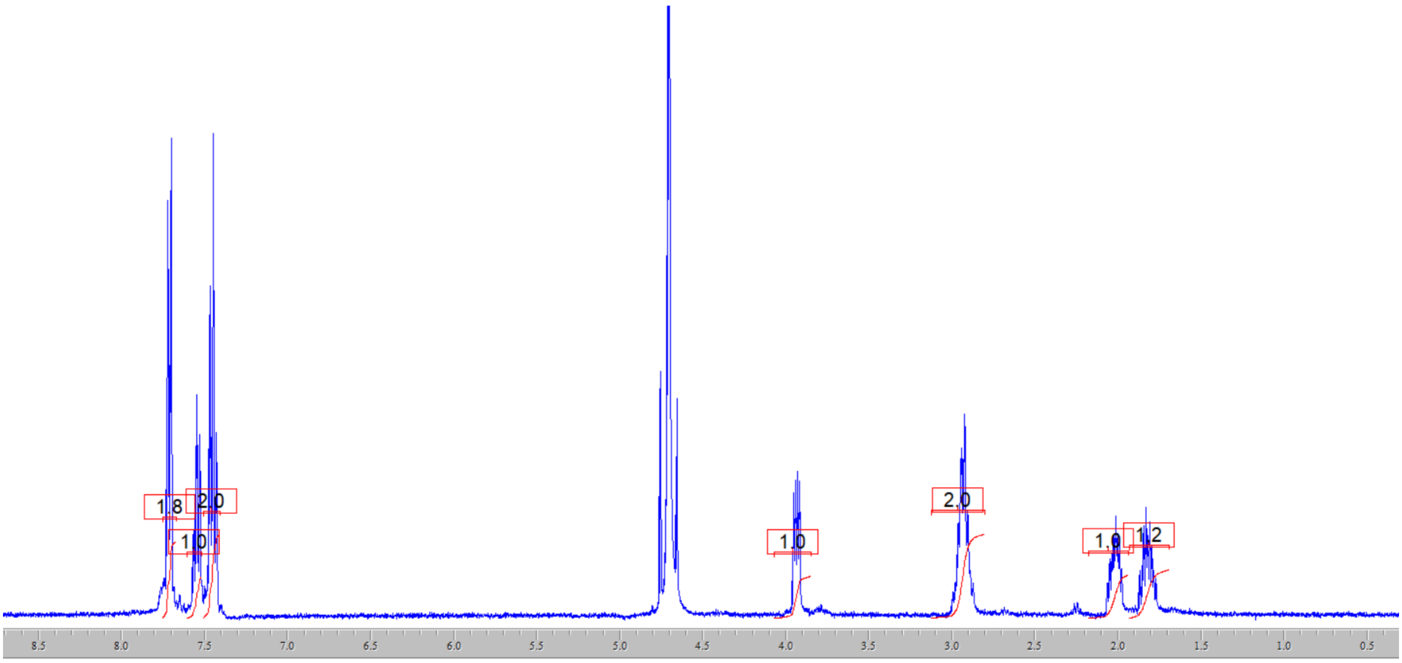

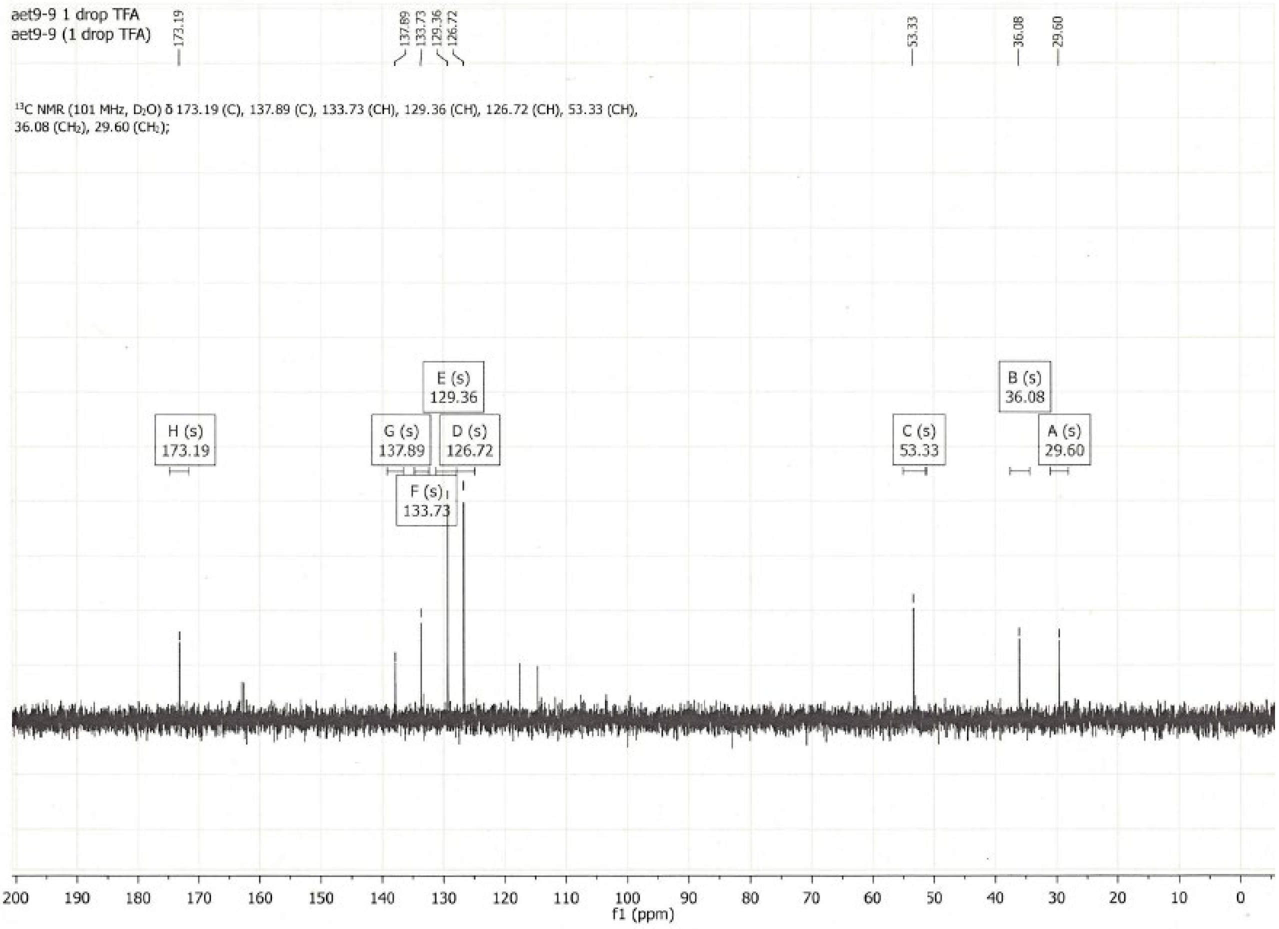
aet9-9 1 drop TFA
aet9-9 (1 drop TFA)
173.19
137.89
133.73
129.36
126.72
53.33
36.08
29.60
^{13}C NMR (101 MHz, D_2O) δ 173.19 (C), 137.89 (C), 133.73 (CH), 129.36 (CH), 126.72 (CH), 53.33 (CH), 36.08 (CH_2), 29.60 (CH_2);
H (s) 173.19
G (s) 137.89
F (s) 133.73
E (s) 129.36
D (s) 126.72
C (s) 53.33
B (s) 36.08
A (s) 29.60
200 190 180 170 160 150 140 130 120 110 100 90 80 70 60 50 40 30 20 10 0
f1 (ppm)

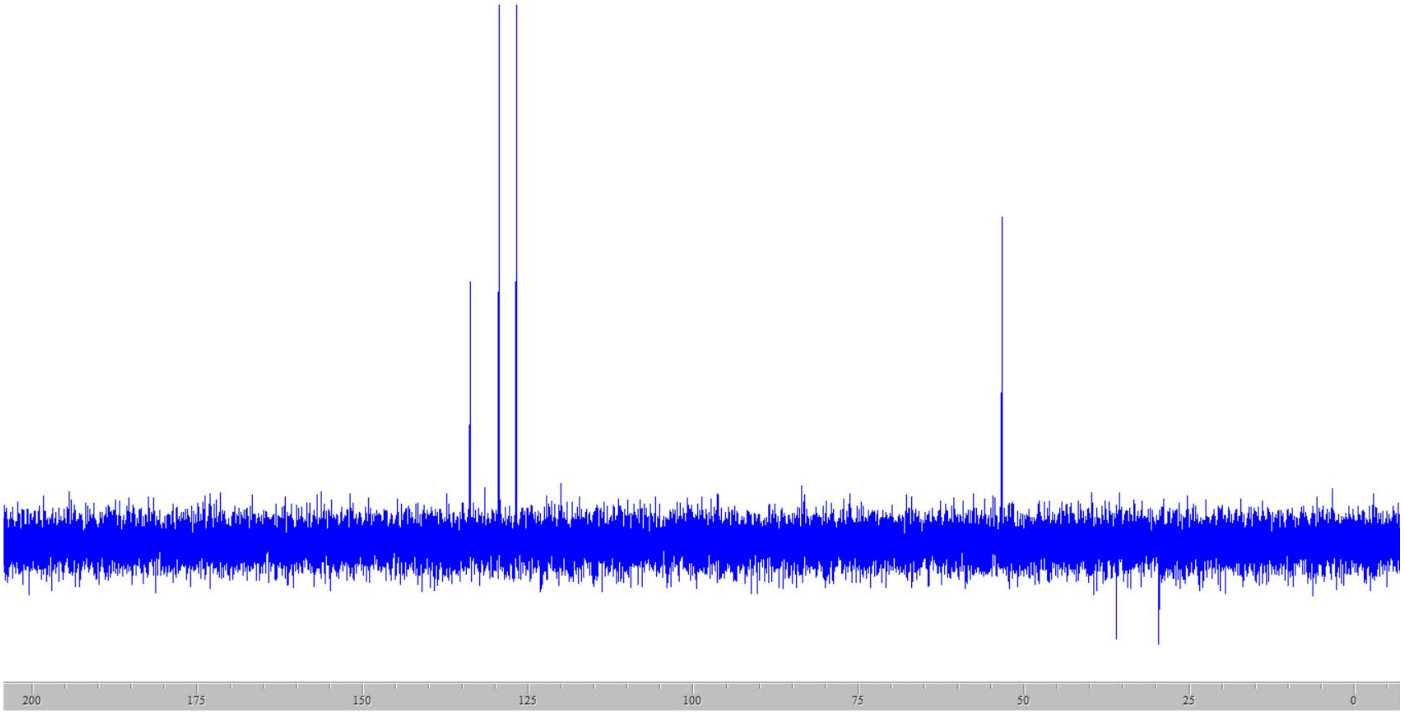
200 175 150 125 100 75 50 25 0

(*S*)-4-Amino-2-(2,4,6-trimethylphenylsulfonamido)butanoic acid [Compound 6]:

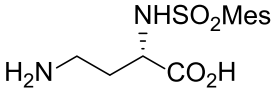

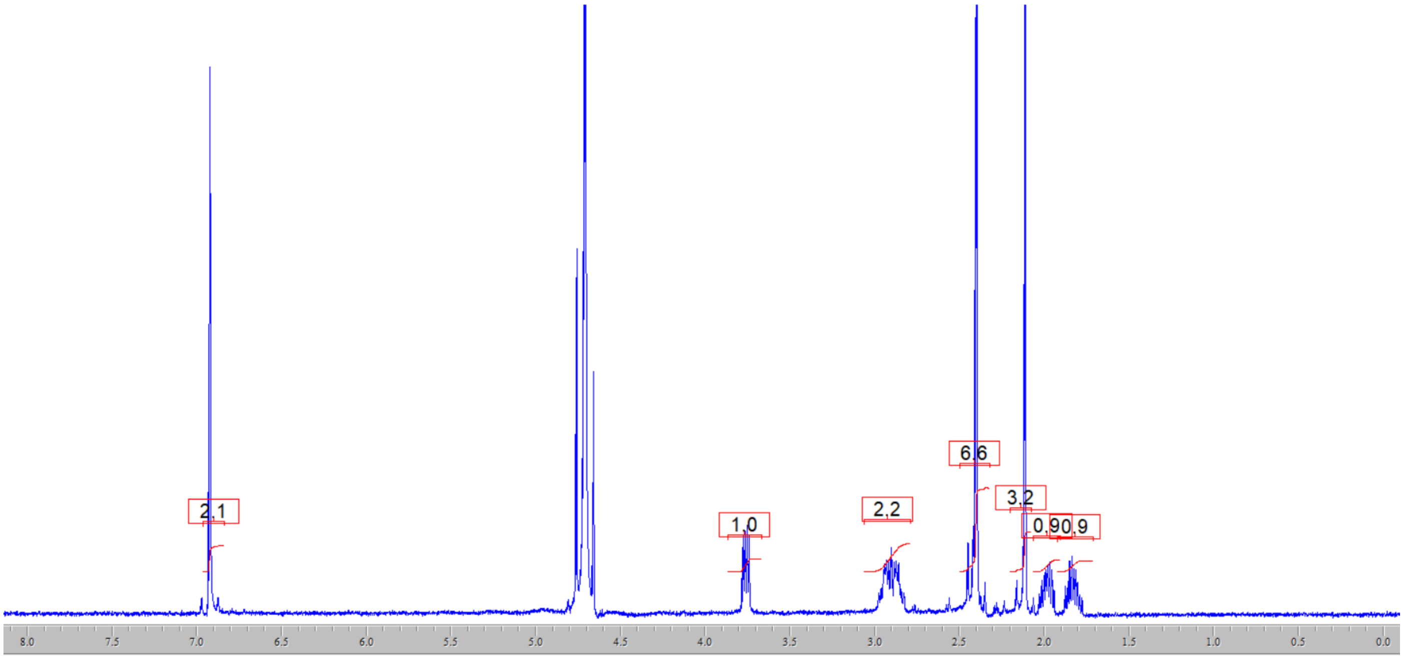

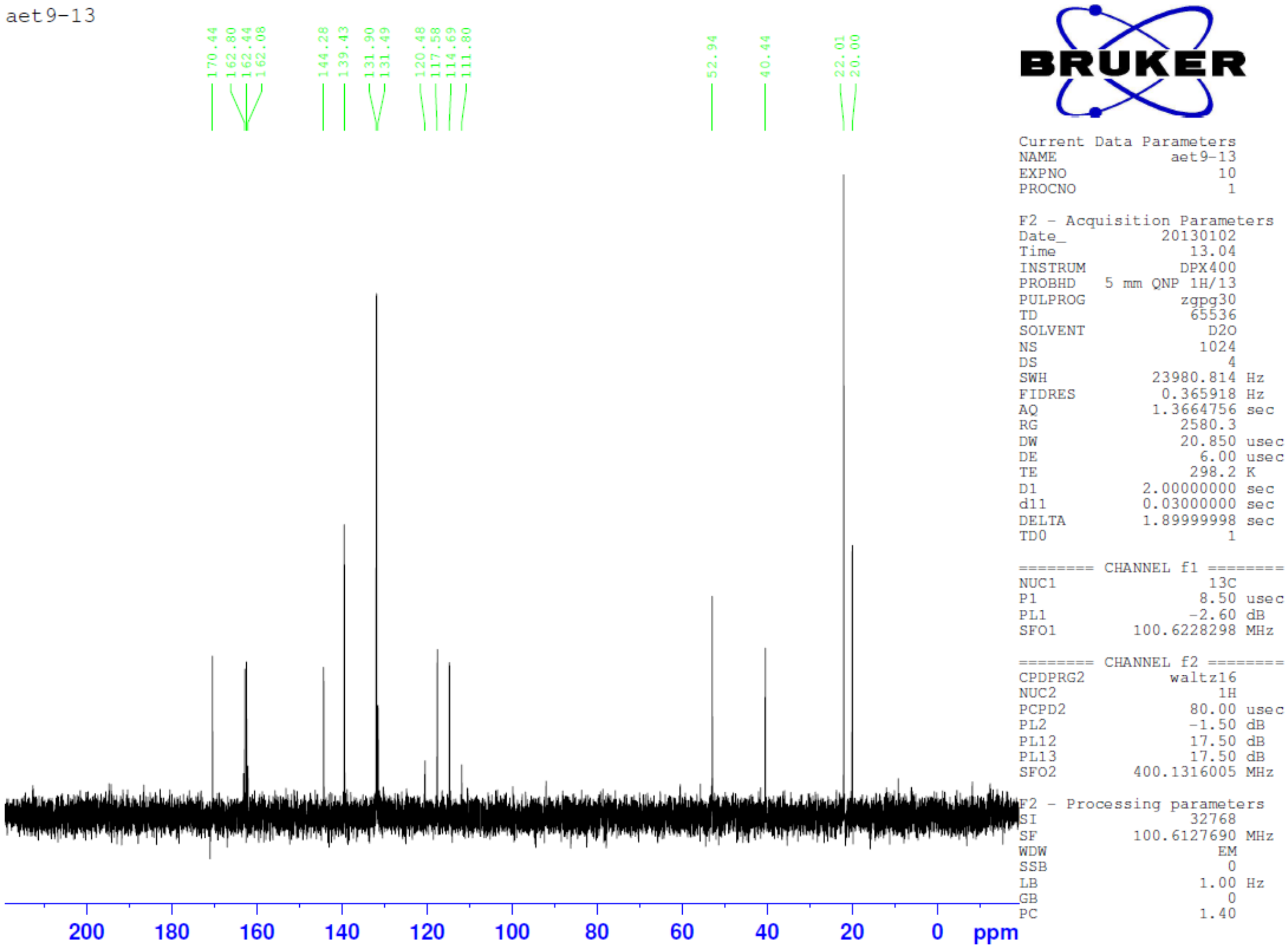

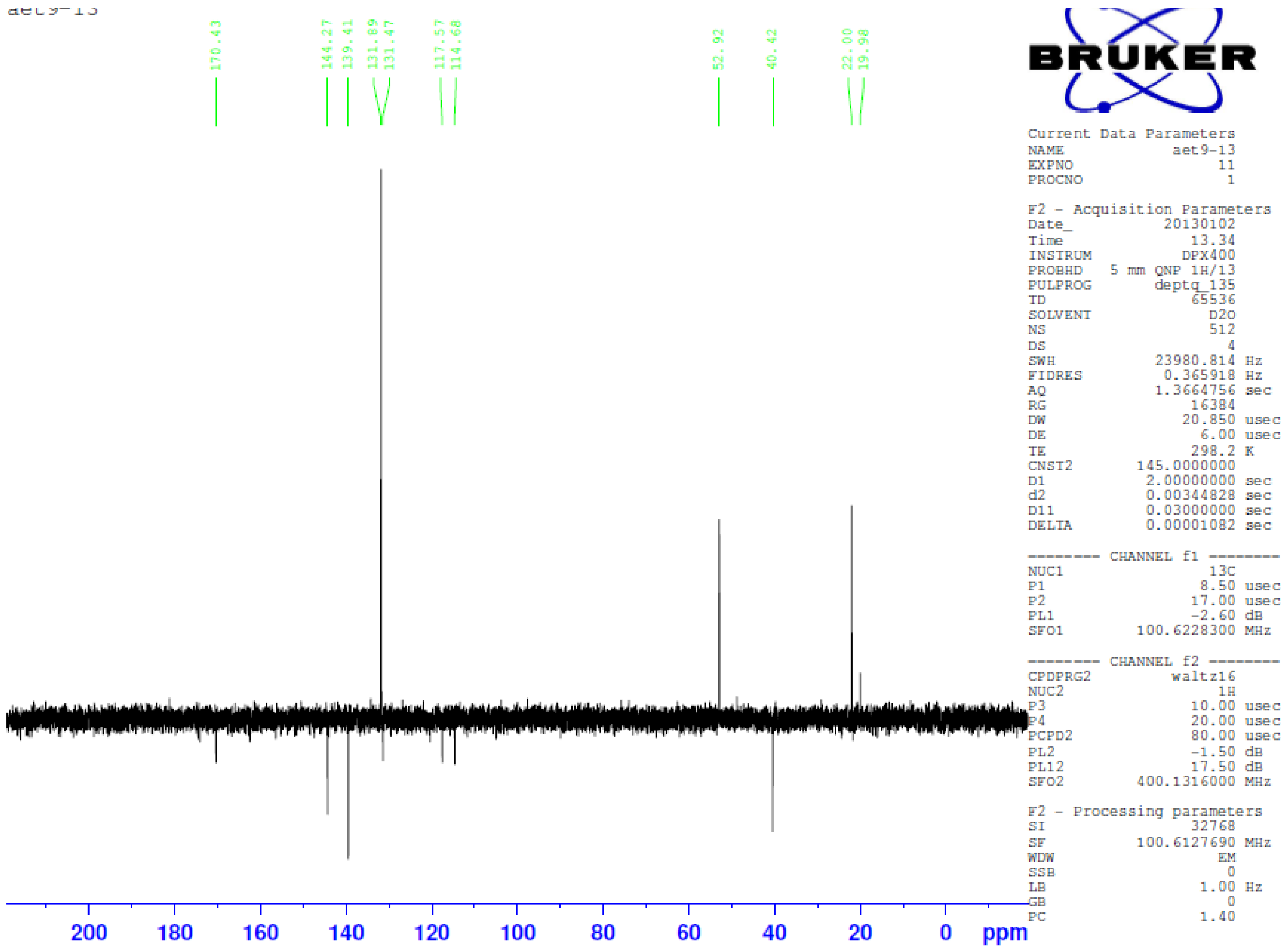
170.43
144.27
139.41
131.89
131.47
117.57
114.68
52.92
40.42
22.00
19.98
BRUKER
Current Data Parameters
NAME aet9-13
EXPNO 11
PROCNO 1
F2 - Acquisition Parameters
Date_ 20130102
Time 13.34
INSTRUM DPX400
PROBHD 5 mm QNP 1H/13
PULPROG deptq_135
TD 65536
SOLVENT D2O
NS 512
DS 4
SWH 23980.814 Hz
FIDRES 0.365918 Hz
AQ 1.3664756 sec
RG 16384
DW 20.850 usec
DE 6.00 usec
TE 298.2 K
CNST2 145.0000000
D1 2.00000000 sec
d2 0.00344828 sec
D11 0.03000000 sec
DELTA 0.00001082 sec
======== CHANNEL f1 ========
NUC1 13C
P1 8.50 usec
P2 17.00 usec
PL1 -2.60 dB
SFO1 100.6228300 MHz
======== CHANNEL f2 ========
CPDPRG2 waltz16
NUC2 1H
P3 10.00 usec
P4 20.00 usec
PCPD2 80.00 usec
PL2 -1.50 dB
PL12 17.50 dB
SFO2 400.1316000 MHz
F2 - Processing parameters
SI 32768
SF 100.6127690 MHz
WDW EM
SSB 0
LB 1.00 Hz
GB 0
PC 1.40
200
180
160
140
120
100
80
60
40
20
0
ppm

Methyl (*S*)-4-amino-2-(phenylsulfonamido)butanoate [Compound 7]

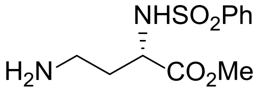

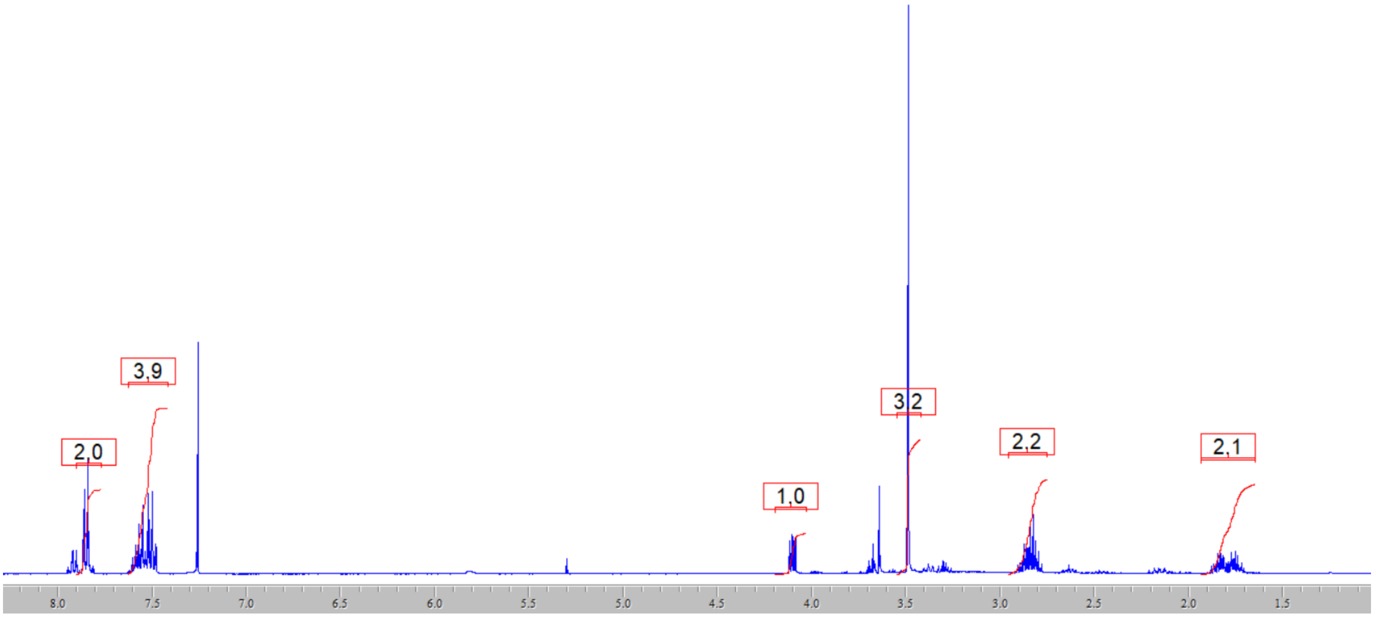

Methyl (*S*)-4-amino-2-(2,4,6-trimethylphenylsulfonamido)butanoate [Compound 8]

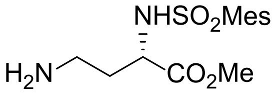

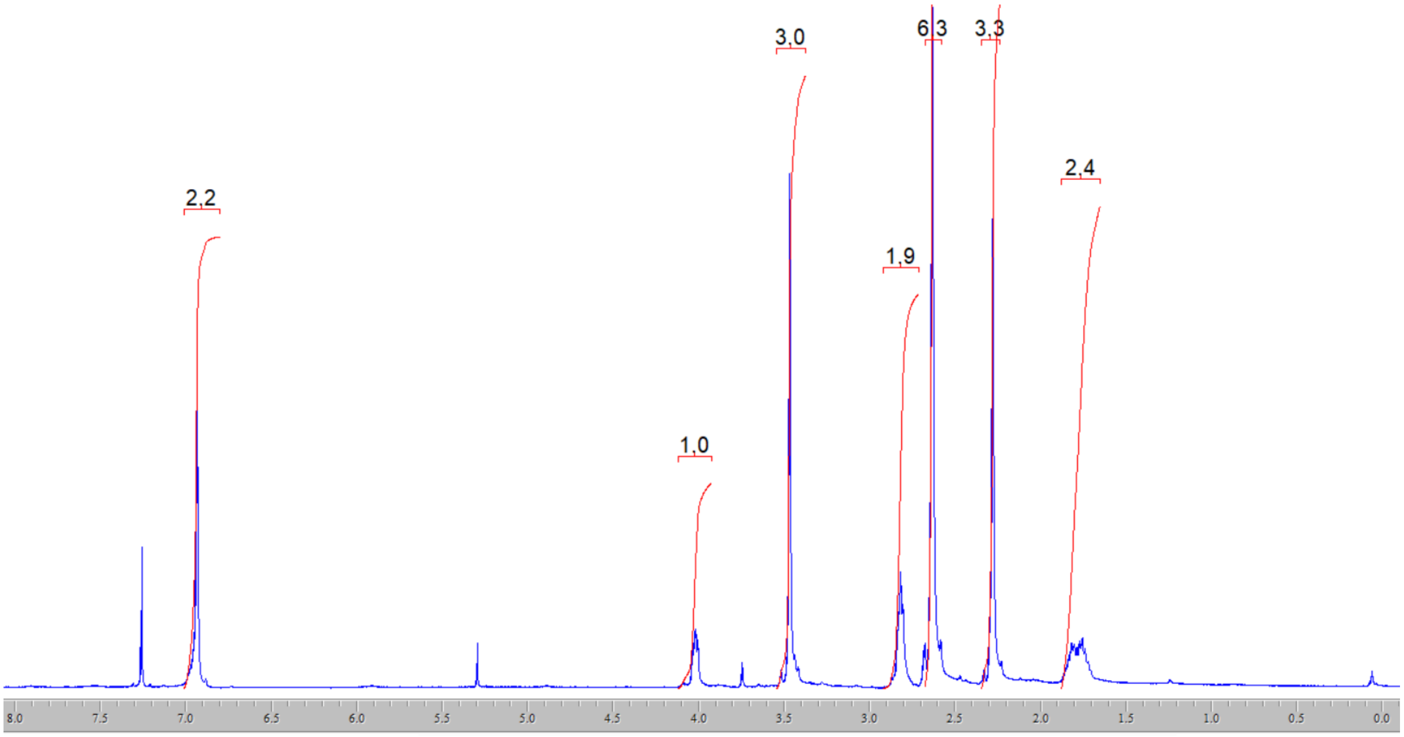

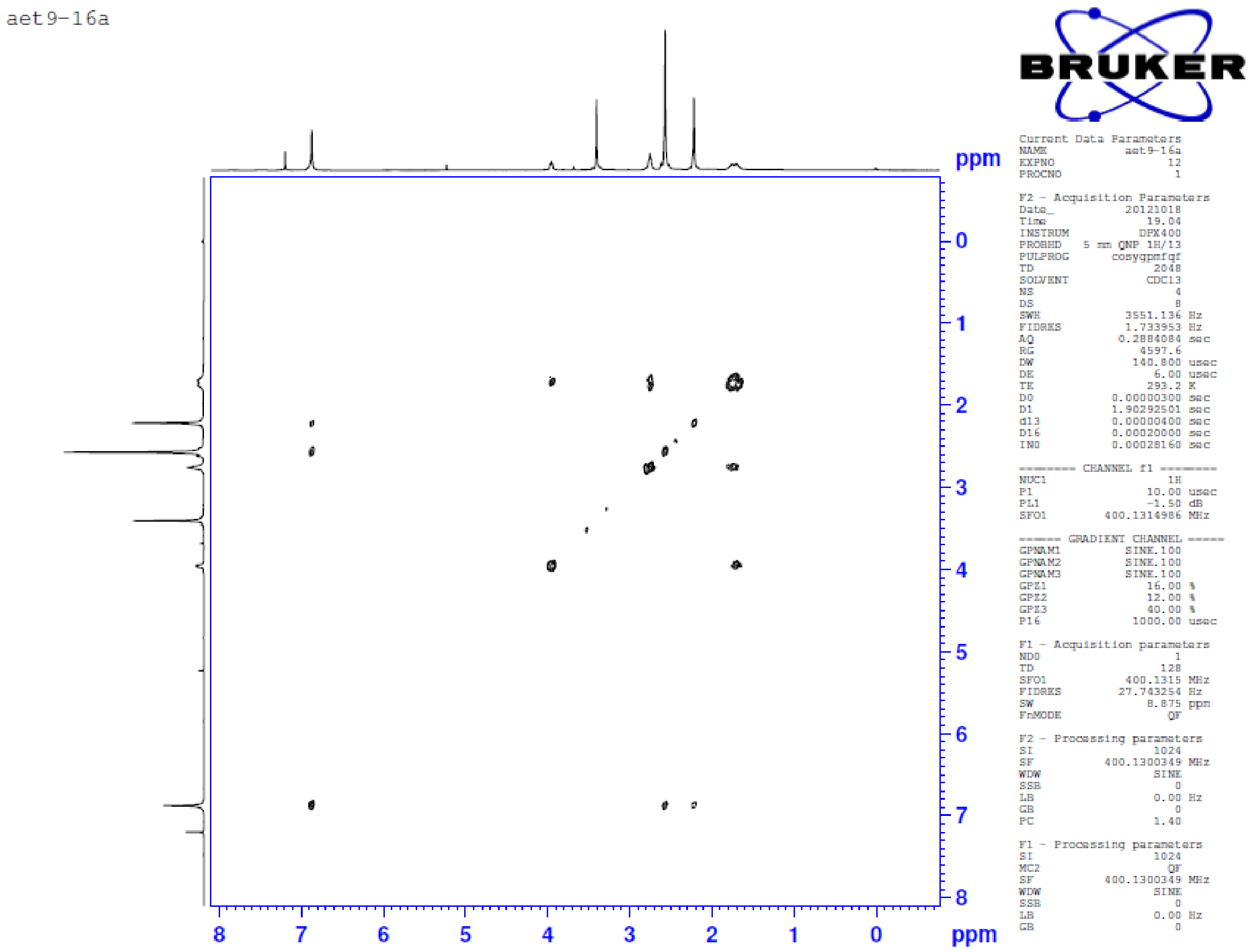

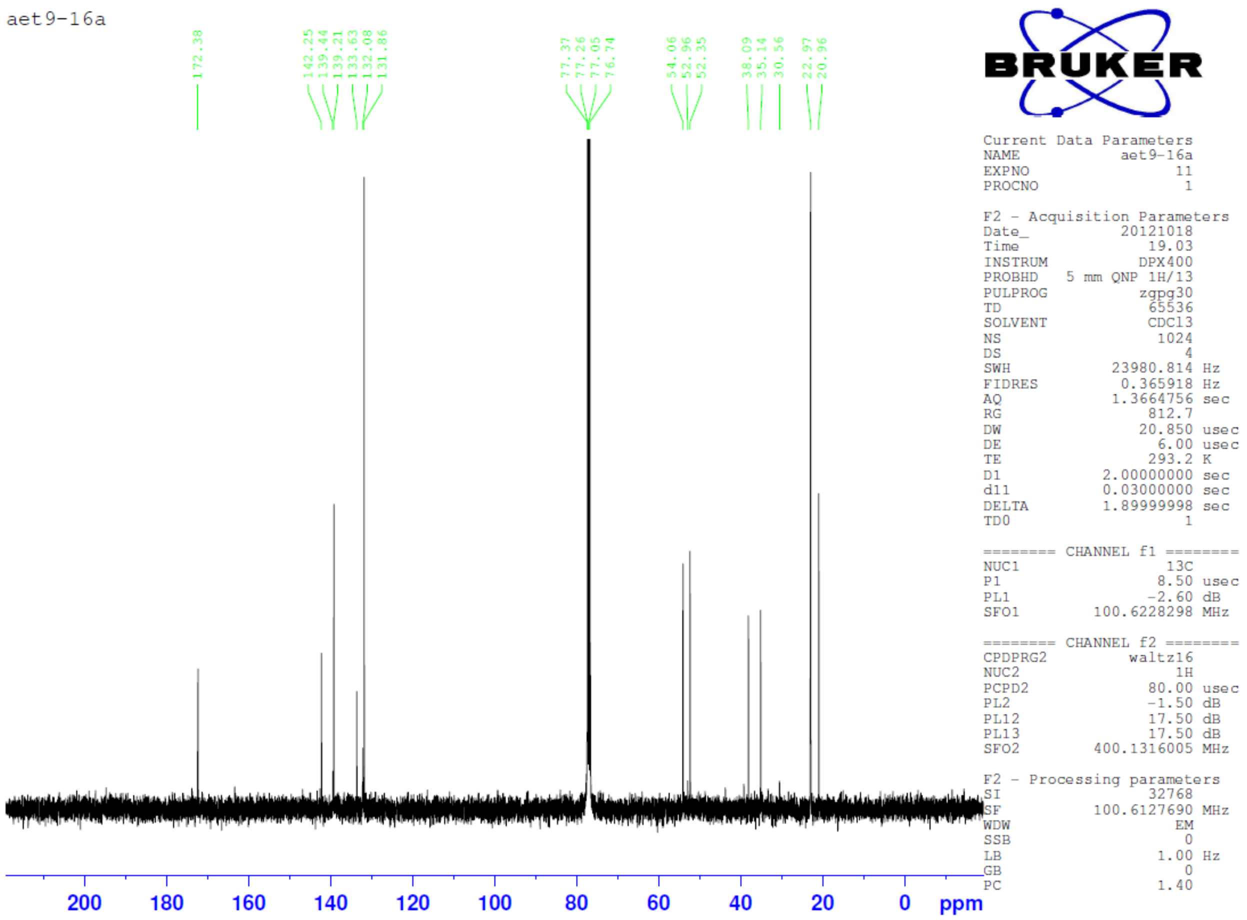
aet9-16a
172.38
142.25
139.44
139.21
133.63
132.08
131.86
77.37
77.26
77.05
76.74
54.06
52.96
52.35
38.09
35.14
30.56
22.97
20.96
BRUKER
Current Data Parameters
NAME aet9-16a
EXPNO 11
PROCNO 1
F2 - Acquisition Parameters
Date_ 20121018
Time 19.03
INSTRUM DPX400
PROBHD 5 mm QNP 1H/13
PULPROG zgpg30
TD 65536
SOLVENT CDCl3
NS 1024
DS 4
SWH 23980.814 Hz
FIDRES 0.365918 Hz
AQ 1.3664756 sec
RG 812.7
DW 20.850 usec
DE 6.00 usec
TE 293.2 K
D1 2.00000000 sec
d11 0.03000000 sec
DELTA 1.89999998 sec
TD0 1
======== CHANNEL f1 ========
NUC1 13C
P1 8.50 usec
PL1 -2.60 dB
SFO1 100.6228298 MHz
======== CHANNEL f2 ========
CPDPRG2 waltz16
NUC2 1H
PCPD2 80.00 usec
PL2 -1.50 dB
PL12 17.50 dB
PL13 17.50 dB
SFO2 400.1316005 MHz
F2 - Processing parameters
SI 32768
SF 100.6127690 MHz
WDW EM
SSB 0
LB 1.00 Hz
GB 0
PC 1.40
200
180
160
140
120
100
80
60
40
20
0
ppm

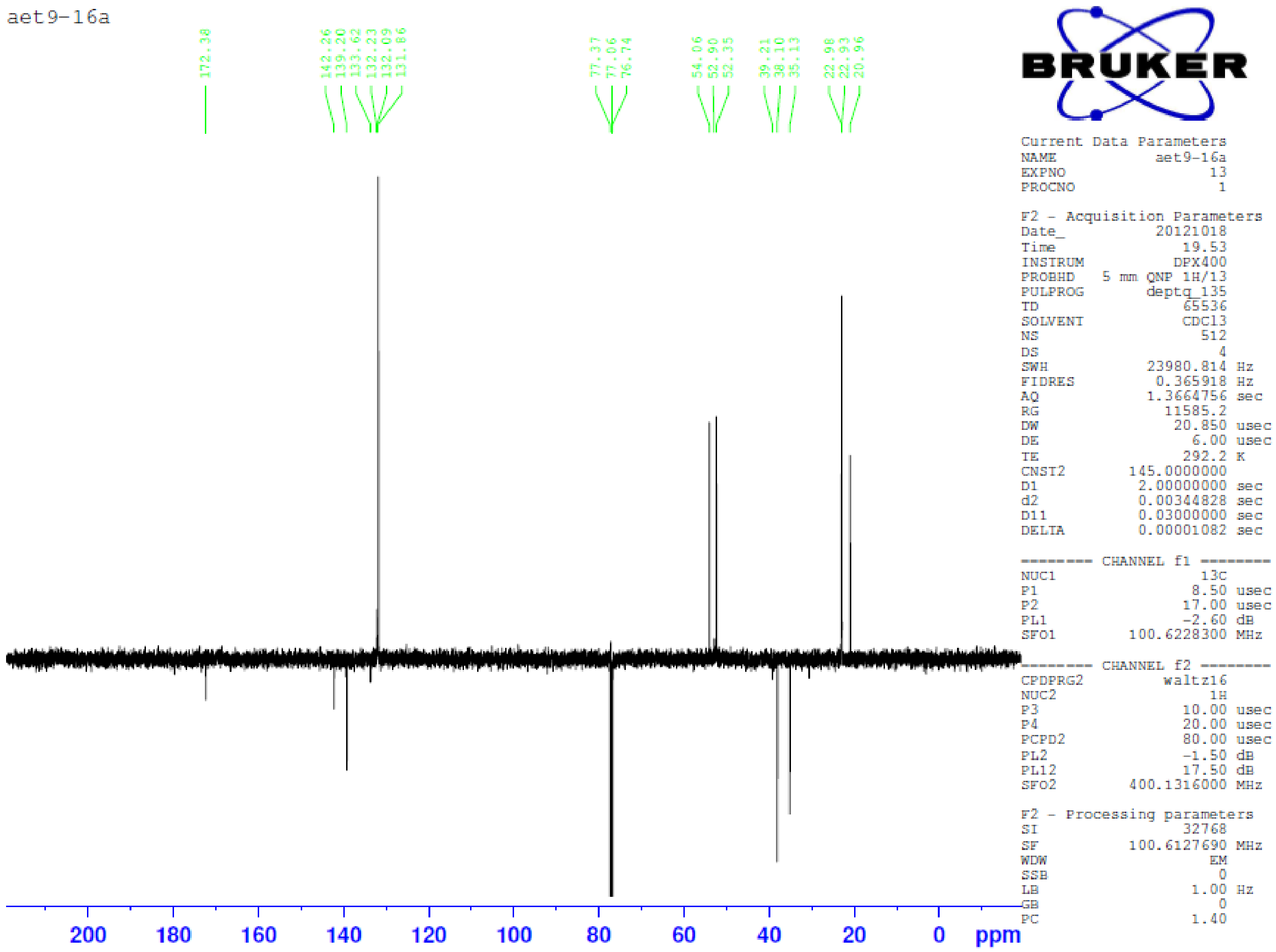
aet9-16a
172.38
142.26
139.20
133.62
132.23
132.09
131.86
77.37
77.06
76.74
54.06
52.90
52.35
39.21
38.10
35.13
22.98
22.93
20.96
BRUKER
Current Data Parameters
NAME aet9-16a
EXPNO 13
PROCNO 1
F2 - Acquisition Parameters
Date_ 20121018
Time 19.53
INSTRUM DPX400
PROBHD 5 mm QNP 1H/13
PULPROG deptq_135
TD 65536
SOLVENT CDCl3
NS 512
DS 4
SWH 23980.814 Hz
FIDRES 0.365918 Hz
AQ 1.3664756 sec
RG 11585.2
DW 20.850 usec
DE 6.00 usec
TE 292.2 K
CNST2 145.0000000
D1 2.00000000 sec
d2 0.00344828 sec
D11 0.03000000 sec
DELTA 0.00001082 sec
======== CHANNEL f1 ========
NUC1 13C
P1 8.50 usec
P2 17.00 usec
PL1 -2.60 dB
SFO1 100.6228300 MHz
======== CHANNEL f2 ========
CPDPRG2 waltz16
NUC2 1H
P3 10.00 usec
P4 20.00 usec
PCPD2 80.00 usec
PL2 -1.50 dB
PL12 17.50 dB
SFO2 400.1316000 MHz
F2 - Processing parameters
SI 32768
SF 100.6127690 MHz
WDW EM
SSB 0
LB 1.00 Hz
GB 0
PC 1.40
200
180
160
140
120
100
80
60
40
20
0
ppm

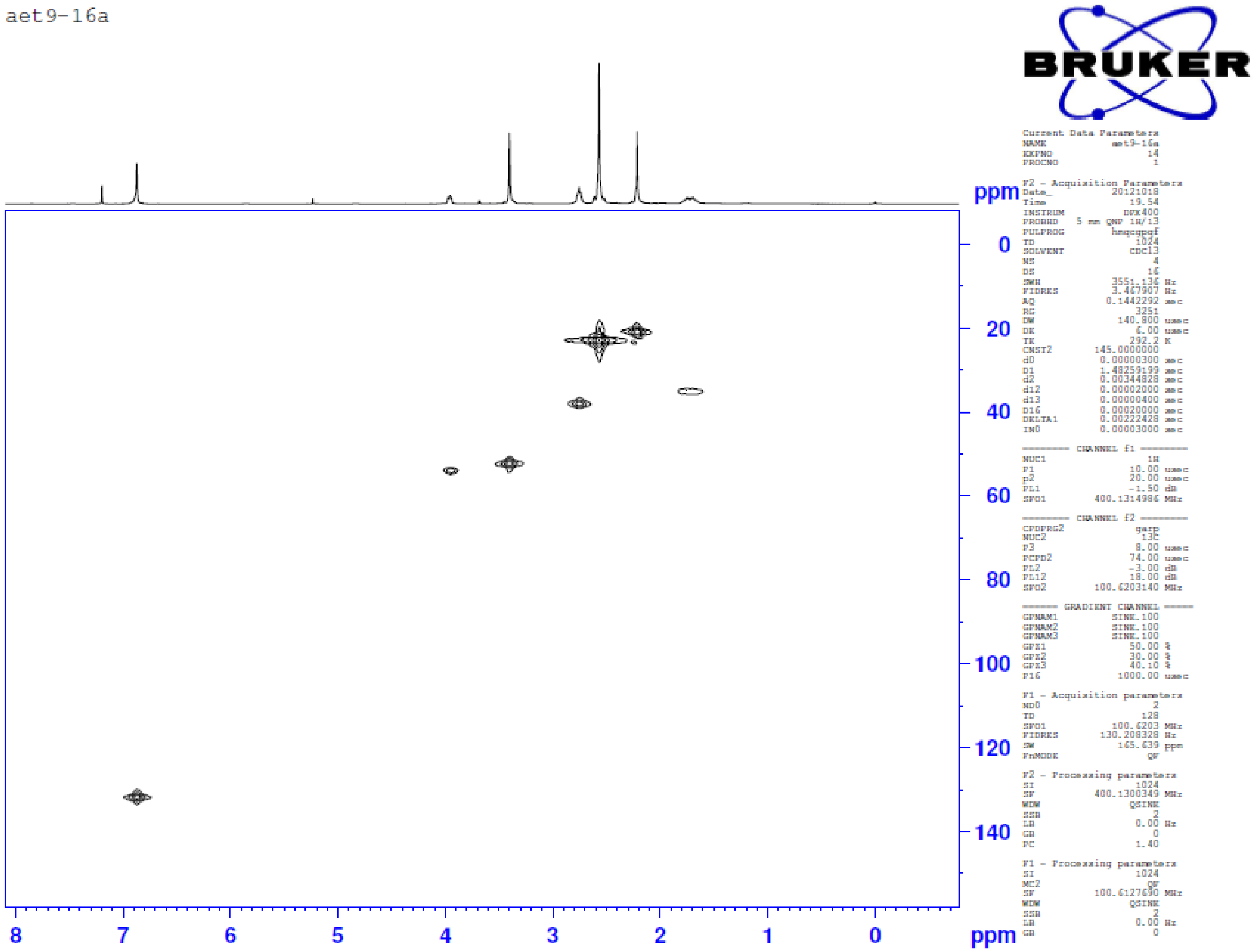
aet9-16a
ppm
0
20
40
60
80
100
120
140
8
7
6
5
4
3
2
1
0
ppm
Current Data Parameters
NAME aet9-16a
EXPNO 14
PROCNO 1
F2 - Acquisition Parameters
Date_ 20121018
Time 19.54
INSTRUM DPX400
PROBHD 5 mm QNP 1H/13
PULPROG hsqcgpqf
TD 1024
SOLVENT CDCl3
NS 4
DS 16
SWH 3551.136 Hz
FIDRES 3.467907 Hz
AQ 0.1442292 sec
RG 3251
DW 140.800 usec
DE 6.00 usec
TE 292.2 K
CNST2 145.0000000
d0 0.00000300 sec
D1 1.48259199 sec
d2 0.00344828 sec
d12 0.00002000 sec
d13 0.00000400 sec
D16 0.00020000 sec
DELTA1 0.00222428 sec
IN0 0.00003000 sec
======== CHANNEL f1 ========
NUC1 1H
P1 10.00 usec
p2 20.00 usec
PL1 -1.50 dB
SFO1 400.1314986 MHz
======== CHANNEL f2 ========
CPDPRG2 garp
NUC2 13C
P3 8.00 usec
PCPD2 74.00 usec
PL2 -3.00 dB
PL12 18.00 dB
SFO2 100.6203140 MHz
====== GRADIENT CHANNEL =====
GPNAM1 SINE.100
GPNAM2 SINE.100
GPNAM3 SINE.100
GPZ1 50.00 %
GPZ2 30.00 %
GPZ3 40.10 %
P16 1000.00 usec
F1 - Acquisition parameters
ND0 2
TD 128
SFO1 100.6203 MHz
FIDRES 130.208328 Hz
SW 165.639 ppm
FnMODE QF
F2 - Processing parameters
SI 1024
SF 400.1300349 MHz
WDW QSINE
SSB 2
LB 0.00 Hz
GB 0
PC 1.40
F1 - Processing parameters
SI 1024
MC2 QF
SF 100.6127690 MHz
WDW QSINE
SSB 2
LB 0.00 Hz
GB 0

Methyl (*S*)-4-((1*s,3*R**)-3-((1,8-naphthyridin-2-yl)methyl)cyclobutane-1-carboxamido)-2-(phenylsulfonamido)butanoate [Compound 17]**

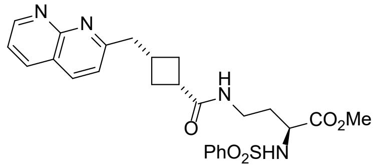

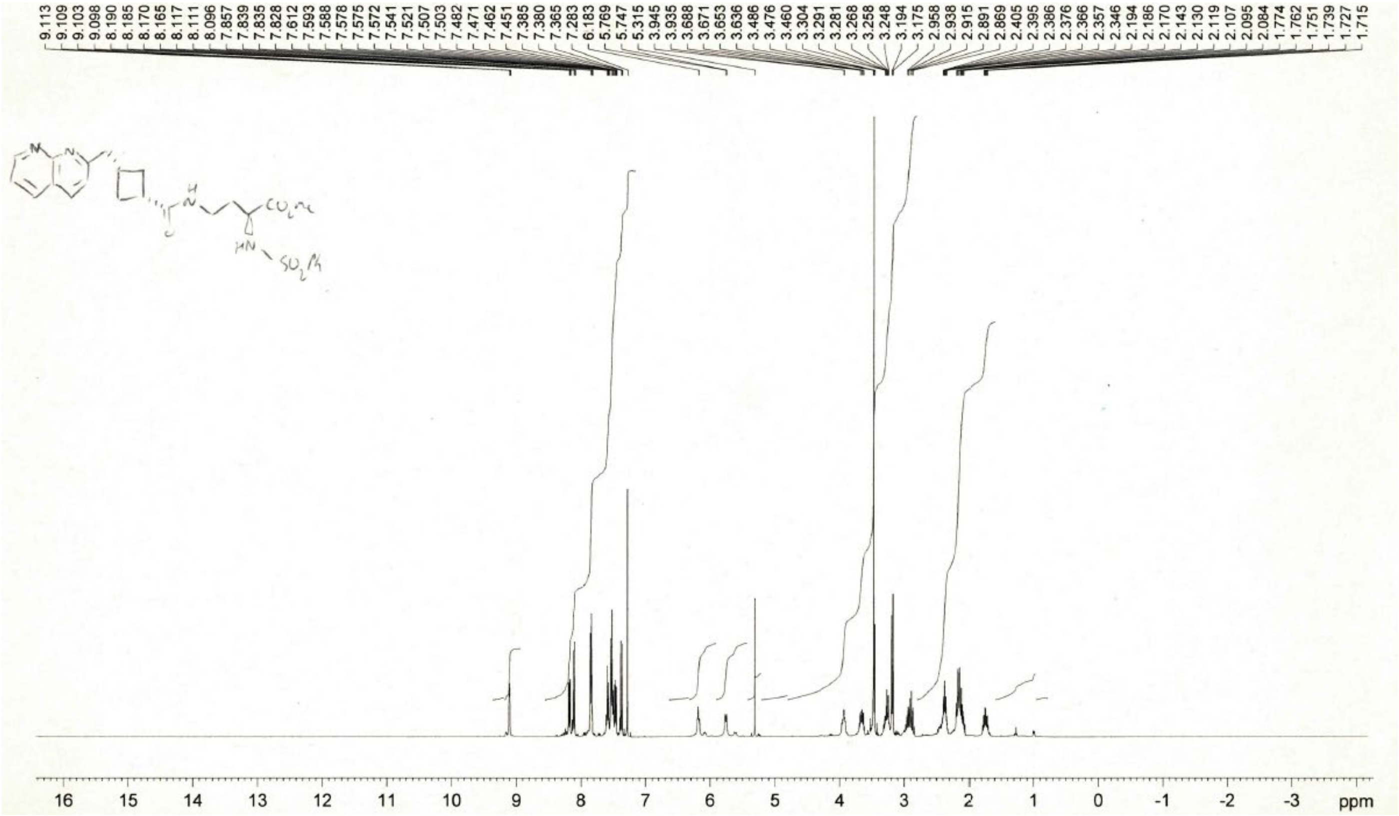

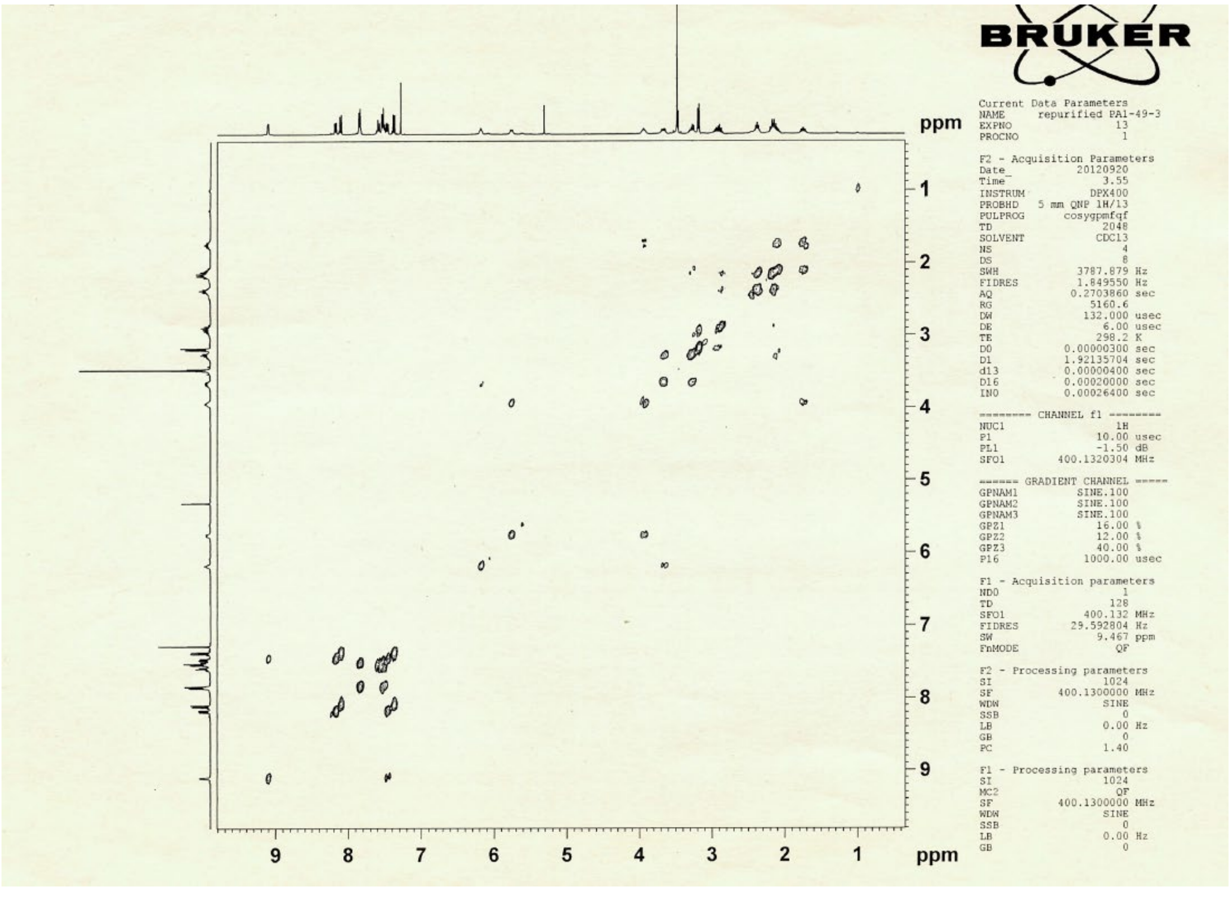

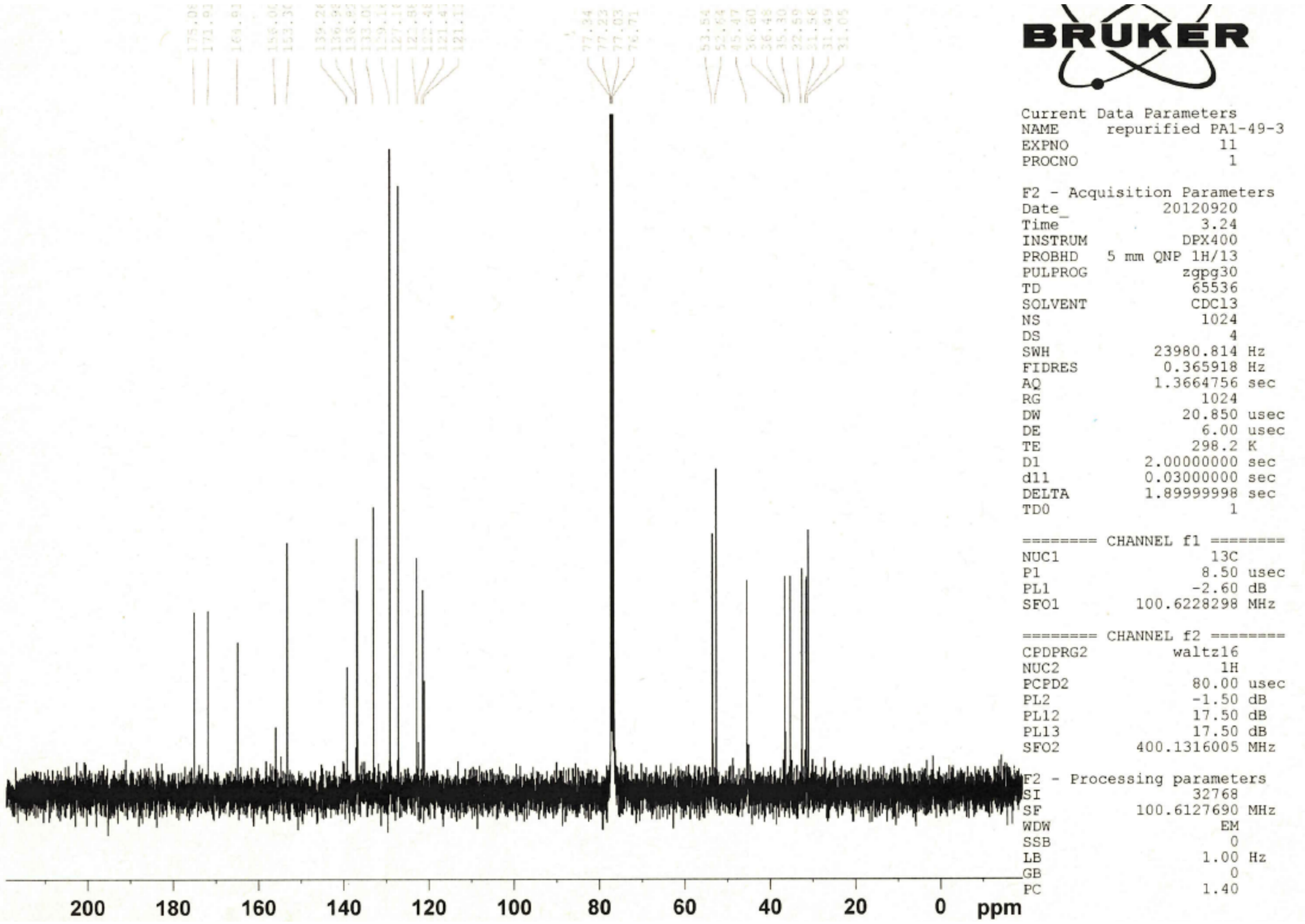
BRUKER
Current Data Parameters
NAME repurified PA1-49-3
EXPNO 11
PROCNO 1
F2 - Acquisition Parameters
Date_ 20120920
Time 3.24
INSTRUM DPX400
PROBHD 5 mm QNP 1H/13
PULPROG zgpg30
TD 65536
SOLVENT CDCl3
NS 1024
DS 4
SWH 23980.814 Hz
FIDRES 0.365918 Hz
AQ 1.3664756 sec
RG 1024
DW 20.850 usec
DE 6.00 usec
TE 298.2 K
D1 2.00000000 sec
d11 0.03000000 sec
DELTA 1.89999998 sec
TD0 1
======== CHANNEL f1 ========
NUC1 13C
P1 8.50 usec
PL1 -2.60 dB
SFO1 100.6228298 MHz
======== CHANNEL f2 ========
CPDPRG2 waltz16
NUC2 1H
PCPD2 80.00 usec
PL2 -1.50 dB
PL12 17.50 dB
PL13 17.50 dB
SFO2 400.1316005 MHz
F2 - Processing parameters
SI 32768
SF 100.6127690 MHz
WDW EM
SSB 0
LB 1.00 Hz
GB 0
PC 1.40
200 180 160 140 120 100 80 60 40 20 0 ppm

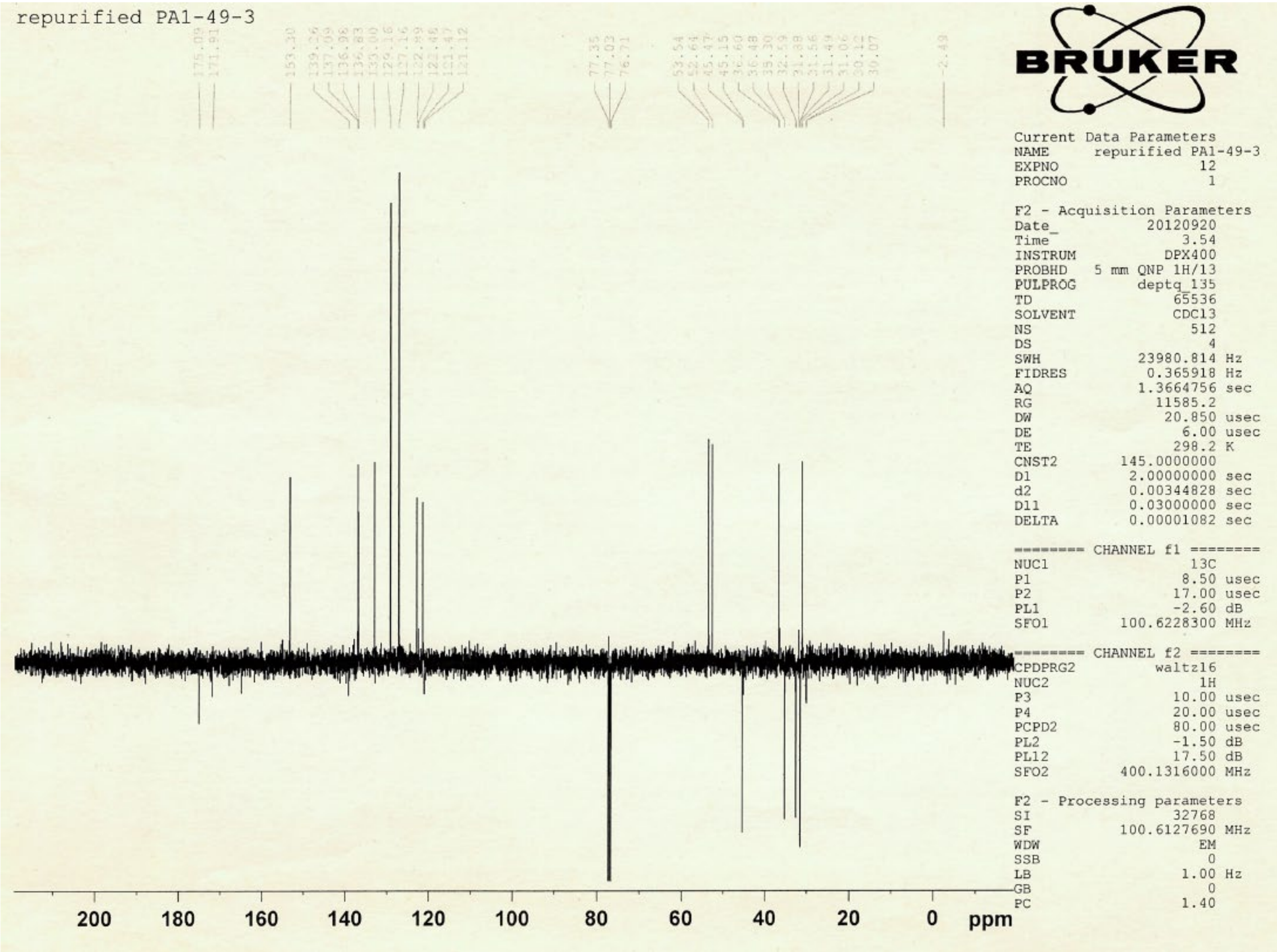
repurified PA1-49-3
BRUKER
Current Data Parameters
NAME repurified PA1-49-3
EXPNO 12
PROCNO 1
F2 - Acquisition Parameters
Date_ 20120920
Time 3.54
INSTRUM DPX400
PROBHD 5 mm QNP 1H/13
PULPROG deptq_135
TD 65536
SOLVENT CDCl3
NS 512
DS 4
SWH 23980.814 Hz
FIDRES 0.365918 Hz
AQ 1.3664756 sec
RG 11585.2
DW 20.850 usec
DE 6.00 usec
TE 298.2 K
CNST2 145.0000000
D1 2.00000000 sec
d2 0.00344828 sec
D11 0.03000000 sec
DELTA 0.00001082 sec
======== CHANNEL f1 ========
NUC1 13C
P1 8.50 usec
P2 17.00 usec
PL1 -2.60 dB
SFO1 100.6228300 MHz
======== CHANNEL f2 ========
CPDPRG2 waltz16
NUC2 1H
P3 10.00 usec
P4 20.00 usec
PCPD2 80.00 usec
PL2 -1.50 dB
PL12 17.50 dB
SFO2 400.1316000 MHz
F2 - Processing parameters
SI 32768
SF 100.6127690 MHz
WDW EM
SSB 0
LB 1.00 Hz
GB 0
PC 1.40
200 180 160 140 120 100 80 60 40 20 0 ppm

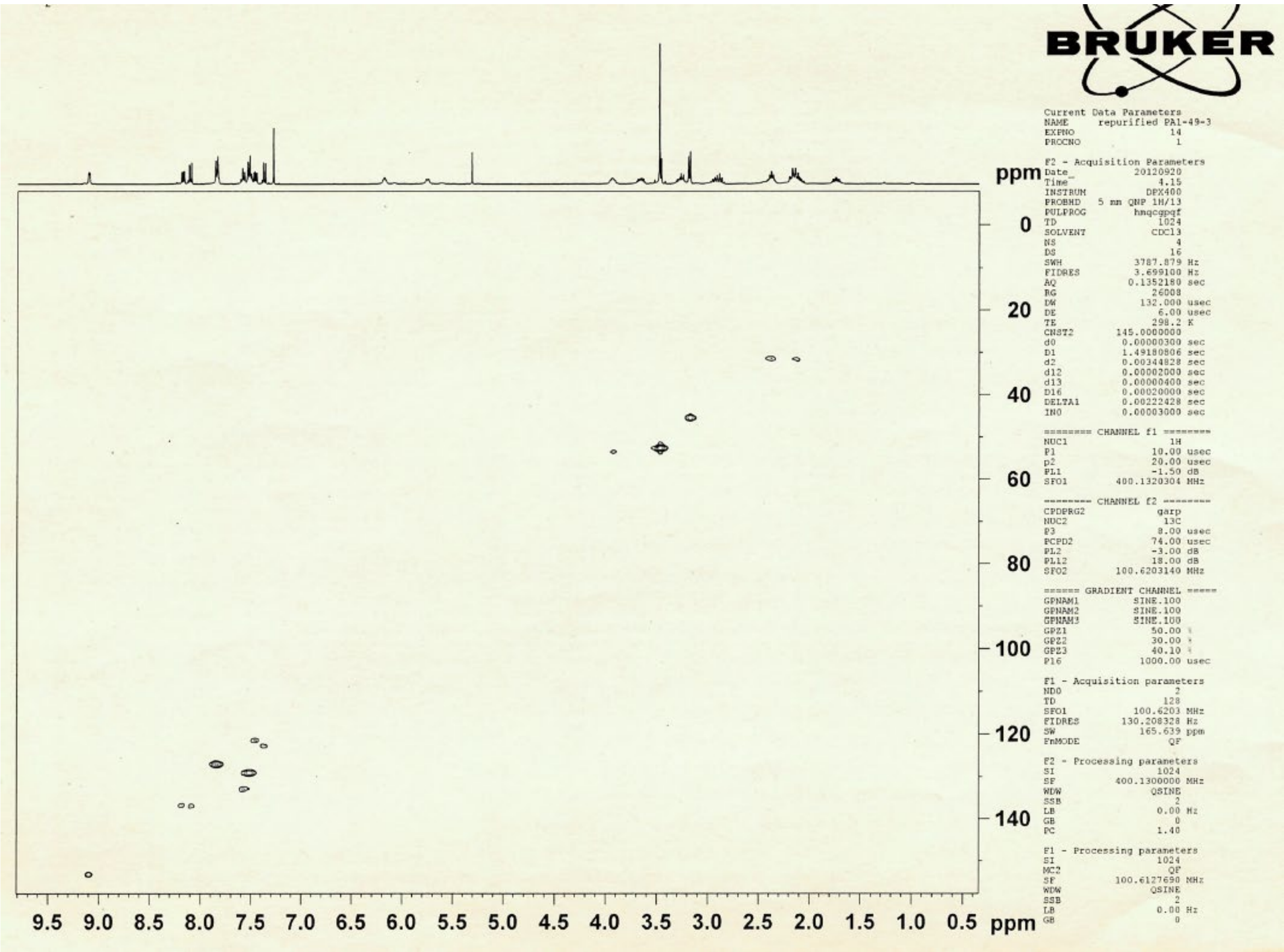

BRUKER
Current Data Parameters
NAME repurified PA1-49-3
EXPNO 14
PROCNO 1
F2 - Acquisition Parameters
Date_ 20120920
Time 4.15
INSTRUM DPX400
PROBHD 5 mm QNP 1H/13
PULPROG hmqcgpqf
TD 1024
SOLVENT CDCl3
NS 4
DS 16
SWH 3787.879 Hz
FIDRES 3.699100 Hz
AQ 0.1352180 sec
RG 26008
DW 132.000 usec
DE 6.00 usec
TE 298.2 K
CNST2 145.0000000
d0 0.00000300 sec
D1 1.49180806 sec
d2 0.00344828 sec
d12 0.00002000 sec
d13 0.00000400 sec
D16 0.00020000 sec
DELTA1 0.00222428 sec
IN0 0.00003000 sec
======== CHANNEL f1 ========
NUC1 1H
P1 10.00 usec
p2 20.00 usec
PL1 -1.50 dB
SFO1 400.1320304 MHz
======== CHANNEL f2 ========
CPDPRG2 garp
NUC2 13C
P3 8.00 usec
PCPD2 74.00 usec
PL2 -3.00 dB
PL12 18.00 dB
SFO2 100.6203140 MHz
====== GRADIENT CHANNEL =====
GPNAM1 SINE.100
GPNAM2 SINE.100
GPNAM3 SINE.100
GPZ1 50.00 %
GPZ2 30.00 %
GPZ3 40.10 %
P16 1000.00 usec
F1 - Acquisition parameters
ND0 2
TD 128
SFO1 100.6203 MHz
FIDRES 130.208328 Hz
SW 165.639 ppm
FnMODE QF
F2 - Processing parameters
SI 1024
SF 400.1300000 MHz
WDW QSINE
SSB 2
LB 0.00 Hz
GB 0
PC 1.40
F1 - Processing parameters
SI 1024
MC2 QF
SF 100.6127690 MHz
WDW QSINE
SSB 2
LB 0.00 Hz
GB 0
ppm
0
20
40
60
80
100
120
140
9.5 9.0 8.5 8.0 7.5 7.0 6.5 6.0 5.5 5.0 4.5 4.0 3.5 3.0 2.5 2.0 1.5 1.0 0.5 ppm

Methyl (*S*)-4-((1*s,3*R**)-3-((1,8-naphthyridin-2-yl)methyl)cyclobutane-1-carboxamido)-2-(2,4,6-trimethylphenylsulfonamido)butanoate [Compound 18]**

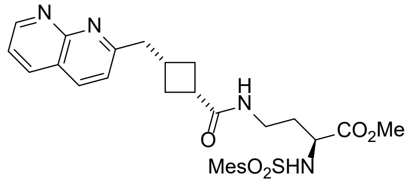

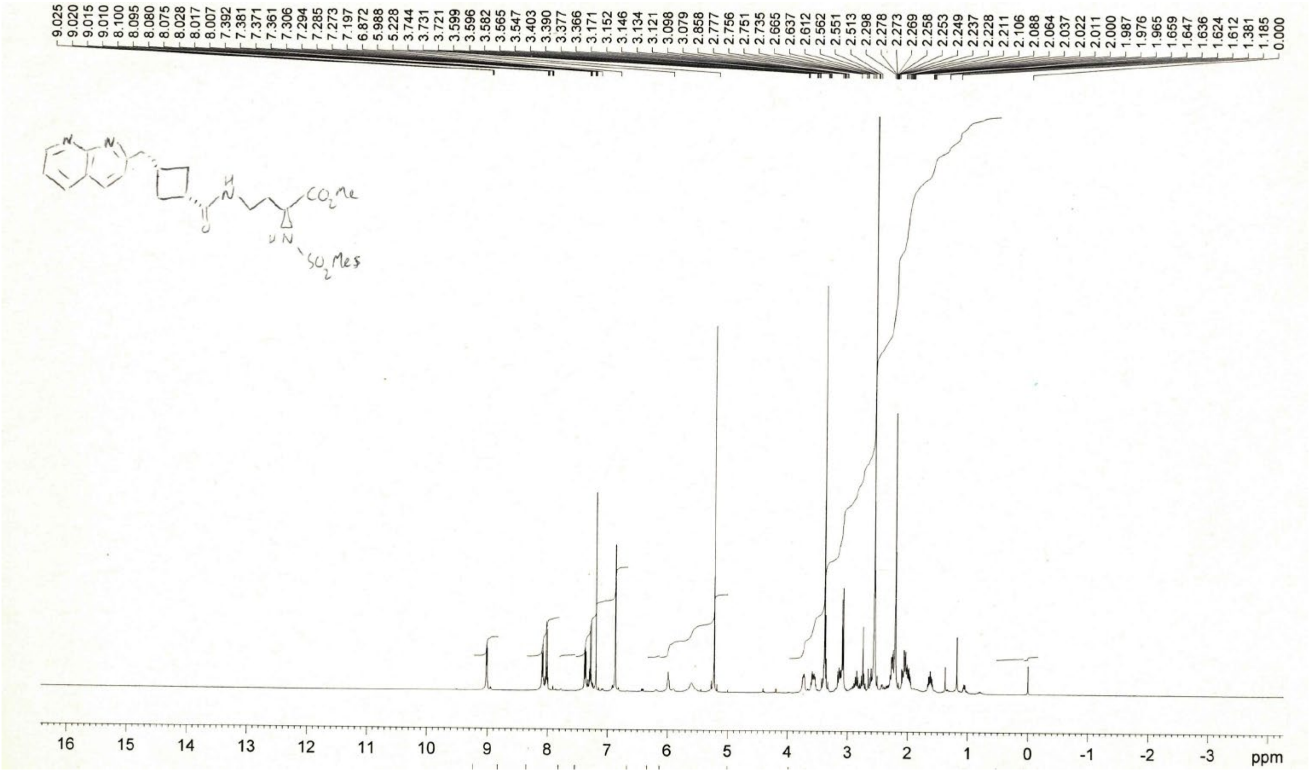

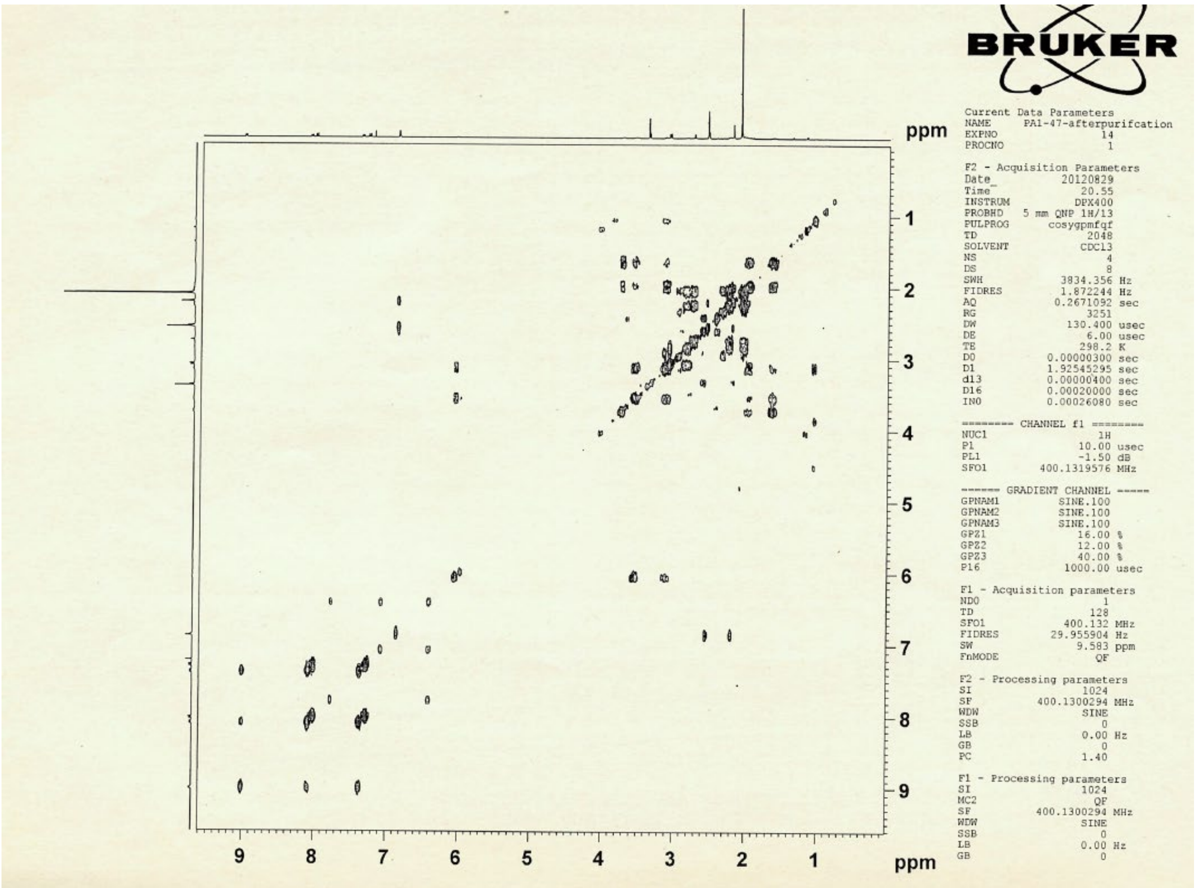

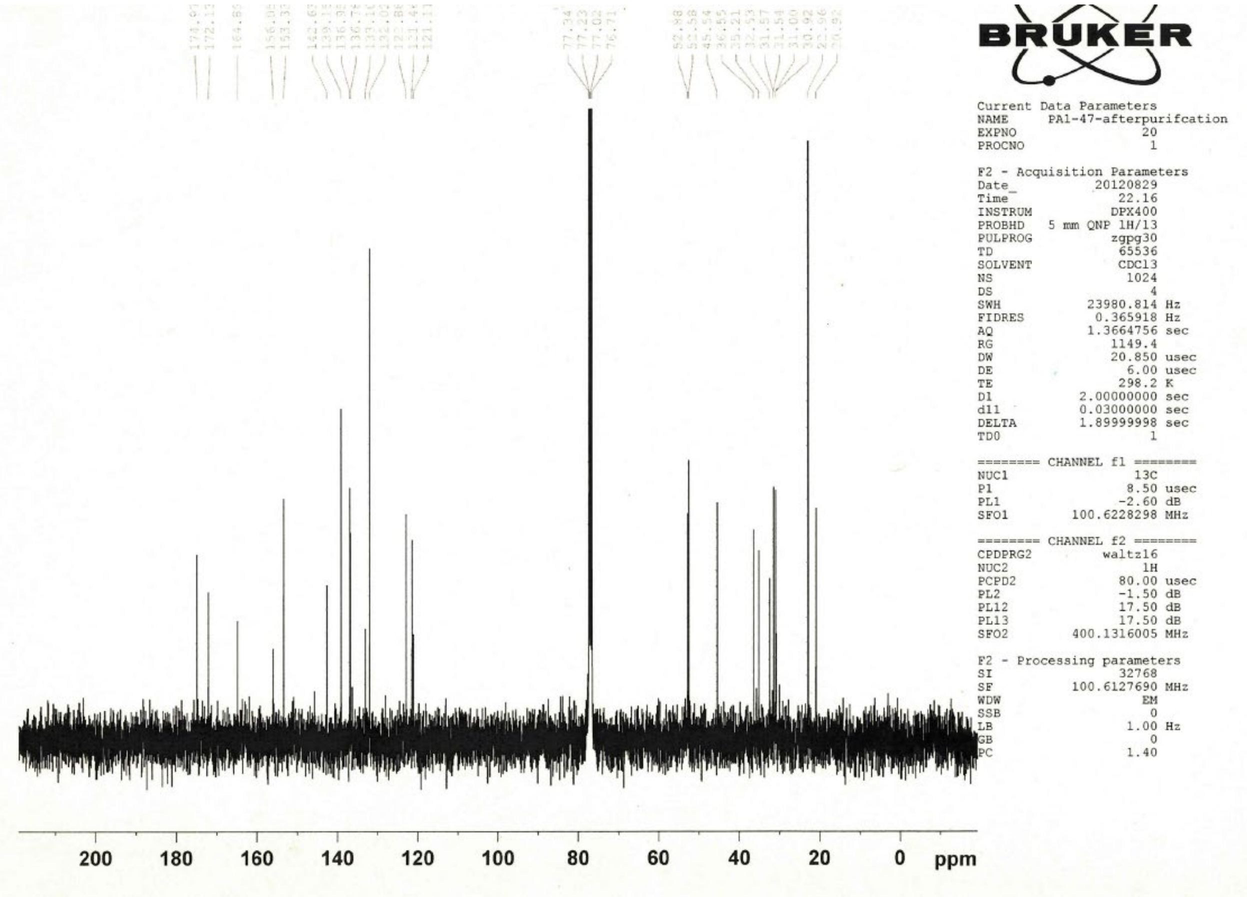
BRUKER
Current Data Parameters
NAME PA1-47-afterpurifcation
EXPNO 20
PROCNO 1
F2 - Acquisition Parameters
Date_ 20120829
Time 22.16
INSTRUM DPX400
PROBHD 5 mm QNP 1H/13
PULPROG zgpg30
TD 65536
SOLVENT CDCl3
NS 1024
DS 4
SWH 23980.814 Hz
FIDRES 0.365918 Hz
AQ 1.3664756 sec
RG 1149.4
DW 20.850 usec
DE 6.00 usec
TE 298.2 K
D1 2.00000000 sec
d11 0.03000000 sec
DELTA 1.89999998 sec
TD0 1
======== CHANNEL f1 ========
NUC1 13C
P1 8.50 usec
PL1 -2.60 dB
SFO1 100.6228298 MHz
======== CHANNEL f2 ========
CPDPRG2 waltz16
NUC2 1H
PCPD2 80.00 usec
PL2 -1.50 dB
PL12 17.50 dB
PL13 17.50 dB
SFO2 400.1316005 MHz
F2 - Processing parameters
SI 32768
SF 100.6127690 MHz
WDW EM
SSB 0
LB 1.00 Hz
GB 0
PC 1.40
200 180 160 140 120 100 80 60 40 20 0 ppm

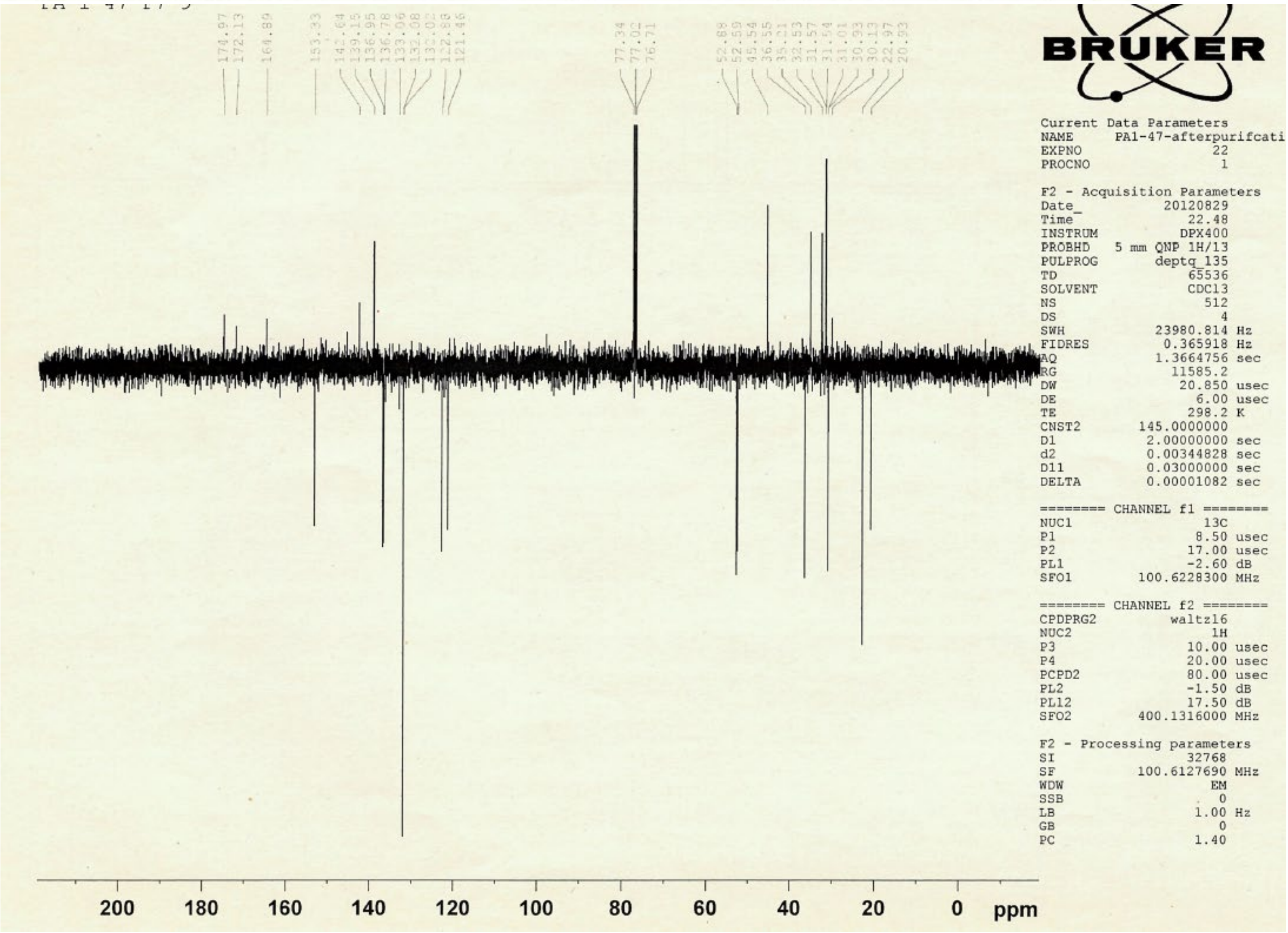
BRUKER
Current Data Parameters
NAME PA1-47-afterpurifcati
EXPNO 22
PROCNO 1
F2 - Acquisition Parameters
Date_ 20120829
Time 22.48
INSTRUM DPX400
PROBHD 5 mm QNP 1H/13
PULPROG deptq_135
TD 65536
SOLVENT CDCl3
NS 512
DS 4
SWH 23980.814 Hz
FIDRES 0.365918 Hz
AQ 1.3664756 sec
RG 11585.2
DW 20.850 usec
DE 6.00 usec
TE 298.2 K
CNST2 145.0000000
D1 2.00000000 sec
d2 0.00344828 sec
D11 0.03000000 sec
DELTA 0.00001082 sec
======== CHANNEL f1 ========
NUC1 13C
P1 8.50 usec
P2 17.00 usec
PL1 -2.60 dB
SFO1 100.6228300 MHz
======== CHANNEL f2 ========
CPDPRG2 waltz16
NUC2 1H
P3 10.00 usec
P4 20.00 usec
PCPD2 80.00 usec
PL2 -1.50 dB
PL12 17.50 dB
SFO2 400.1316000 MHz
F2 - Processing parameters
SI 32768
SF 100.6127690 MHz
WDW EM
SSB 0
LB 1.00 Hz
GB 0
PC 1.40
200 180 160 140 120 100 80 60 40 20 0 ppm

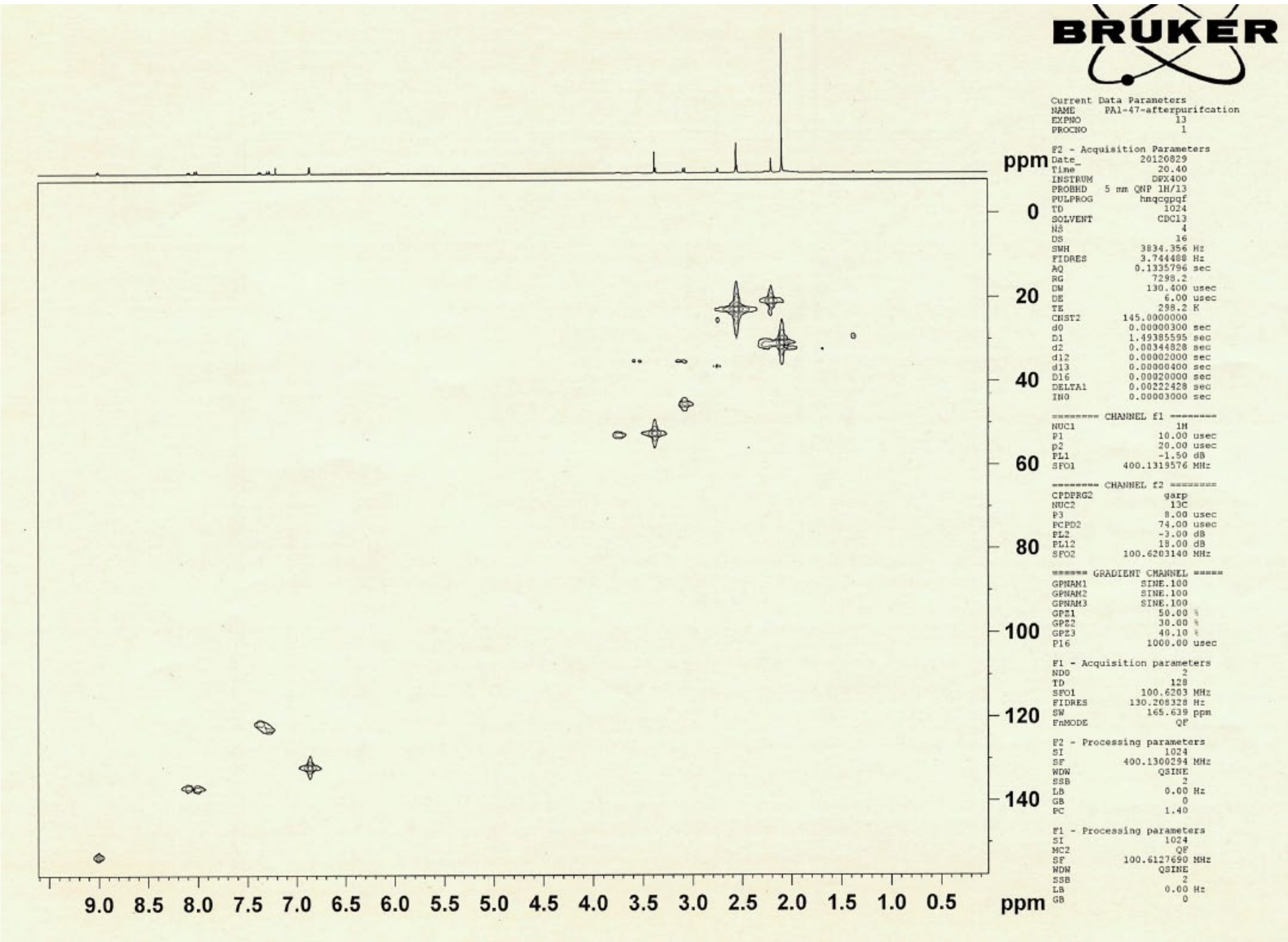
BRUKER
Current Data Parameters
NAME PA1-47-afterpurifcation
EXPNO 13
PROCNO 1
F2 - Acquisition Parameters
Date_ 20120829
Time 20.40
INSTRUM DPX400
PROBHD 5 mm QNP 1H/13
PULPROG hmqcgpqf
TD 1024
SOLVENT CDCl3
NS 4
DS 16
SWH 3834.356 Hz
FIDRES 3.744488 Hz
AQ 0.1335796 sec
RG 7298.2
DW 130.400 usec
DE 6.00 usec
TE 298.2 K
CNST2 145.0000000
d0 0.00000300 sec
D1 1.49385595 sec
d2 0.00344828 sec
d12 0.00002000 sec
d13 0.00000400 sec
D16 0.00020000 sec
DELTA1 0.00222428 sec
IN0 0.00003000 sec
======== CHANNEL f1 ========
NUC1 1H
P1 10.00 usec
p2 20.00 usec
PL1 -1.50 dB
SFO1 400.1319576 MHz
======== CHANNEL f2 ========
CPDPRG2 garp
NUC2 13C
P3 8.00 usec
PCPD2 74.00 usec
PL2 -3.00 dB
PL12 18.00 dB
SFO2 100.6203140 MHz
====== GRADIENT CHANNEL =====
GPNAM1 SINE.100
GPNAM2 SINE.100
GPNAM3 SINE.100
GPZ1 50.00 %
GPZ2 30.00 %
GPZ3 40.10 %
P16 1000.00 usec
F1 - Acquisition parameters
ND0 2
TD 128
SFO1 100.6203 MHz
FIDRES 130.208328 Hz
SW 165.639 ppm
FnMODE QF
F2 - Processing parameters
SI 1024
SF 400.1300294 MHz
WDW QSINE
SSB 2
LB 0.00 Hz
GB 0
PC 1.40
F1 - Processing parameters
SI 1024
MC2 QF
SF 100.6127690 MHz
WDW QSINE
SSB 2
LB 0.00 Hz
GB 0
ppm
0
20
40
60
80
100
120
140
9.0 8.5 8.0 7.5 7.0 6.5 6.0 5.5 5.0 4.5 4.0 3.5 3.0 2.5 2.0 1.5 1.0 0.5
ppm

Methyl (*S*)-4-((1*r,3*R**)-3-(2-(1,8-naphthyridin-2-yl)ethyl)cyclobutane-1-carboxamido)-2-(phenylsulfonamido)butanoate [Compound 19]**

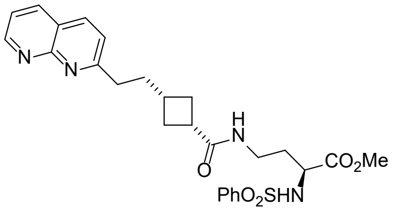

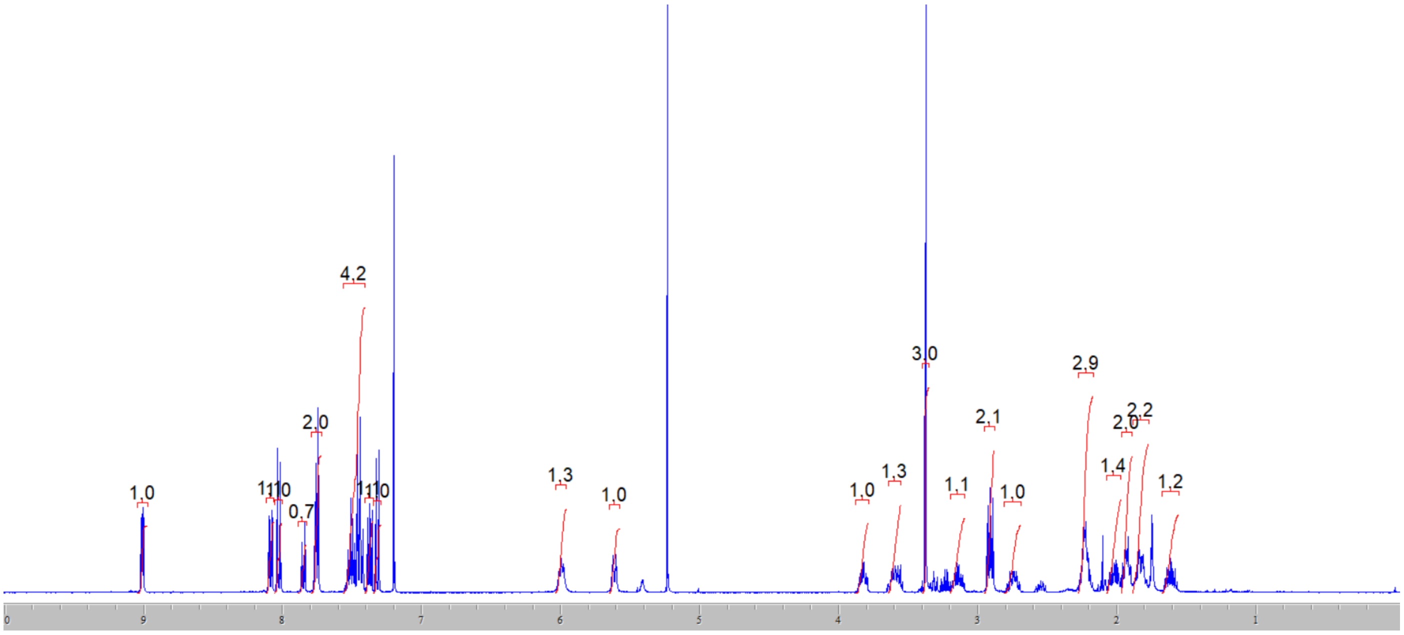

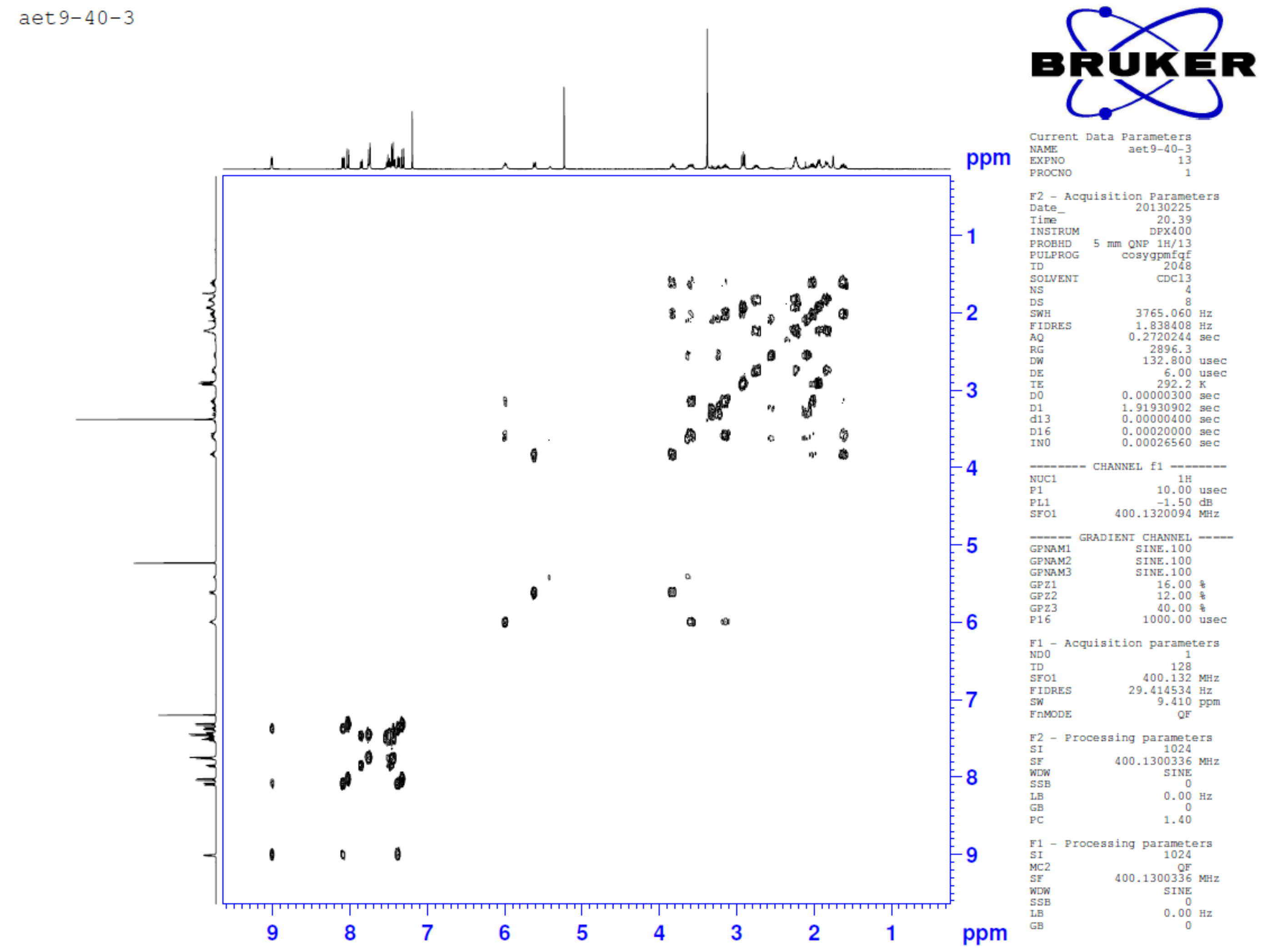

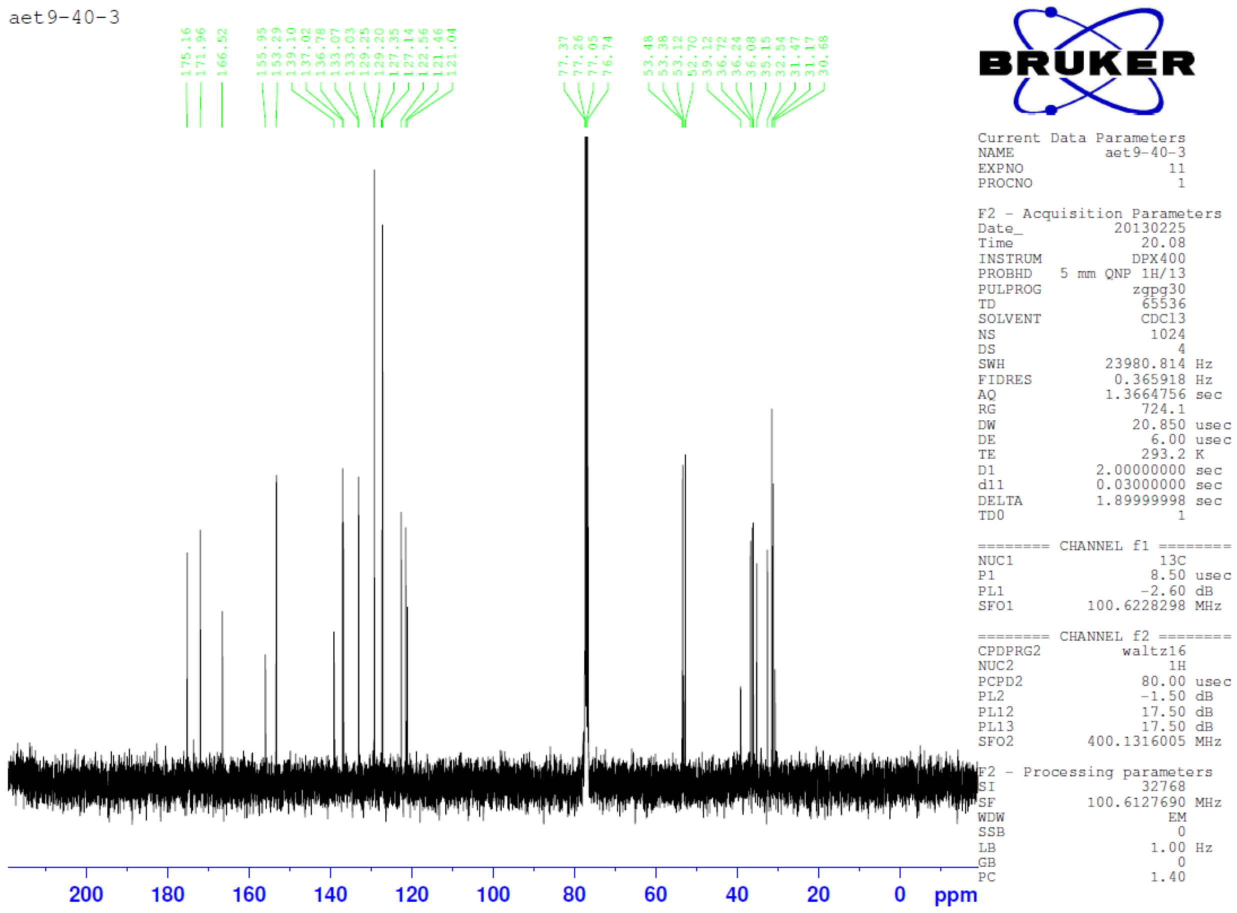
aet9-40-3
175.16
171.96
166.52
155.95
153.29
139.10
137.02
136.78
133.07
133.03
129.25
129.20
127.35
127.14
122.56
121.46
121.04
77.37
77.26
77.05
76.74
53.48
53.38
53.12
52.70
39.12
36.72
36.24
36.08
35.15
32.54
31.47
31.17
30.68
BRUKER
Current Data Parameters
NAME aet9-40-3
EXPNO 11
PROCNO 1
F2 - Acquisition Parameters
Date_ 20130225
Time 20.08
INSTRUM DPX400
PROBHD 5 mm QNP 1H/13
PULPROG zgpg30
TD 65536
SOLVENT CDCl3
NS 1024
DS 4
SWH 23980.814 Hz
FIDRES 0.365918 Hz
AQ 1.3664756 sec
RG 724.1
DW 20.850 usec
DE 6.00 usec
TE 293.2 K
D1 2.00000000 sec
d11 0.03000000 sec
DELTA 1.89999998 sec
TD0 1
======== CHANNEL f1 ========
NUC1 13C
P1 8.50 usec
PL1 -2.60 dB
SFO1 100.6228298 MHz
======== CHANNEL f2 ========
CPDPRG2 waltz16
NUC2 1H
PCPD2 80.00 usec
PL2 -1.50 dB
PL12 17.50 dB
PL13 17.50 dB
SFO2 400.1316005 MHz
F2 - Processing parameters
SI 32768
SF 100.6127690 MHz
WDW EM
SSB 0
LB 1.00 Hz
GB 0
PC 1.40
200
180
160
140
120
100
80
60
40
20
0
ppm

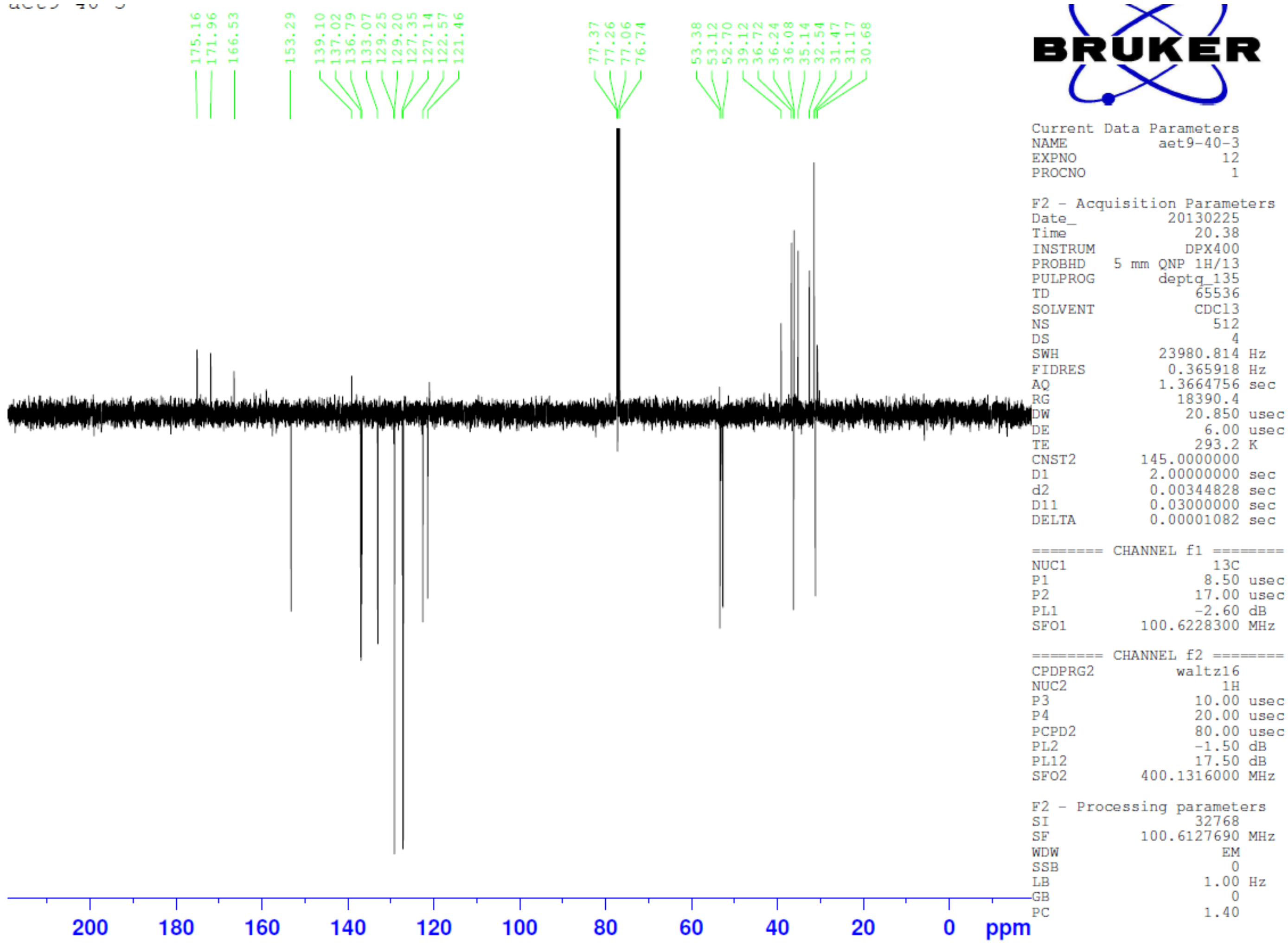
175.16
171.96
166.53
153.29
139.10
137.02
136.79
133.07
129.25
129.20
127.35
127.14
122.57
121.46
77.37
77.26
77.06
76.74
53.38
53.12
52.70
39.12
36.72
36.24
36.08
35.14
32.54
31.47
31.17
30.68
BRUKER
Current Data Parameters
NAME aet9-40-3
EXPNO 12
PROCNO 1
F2 - Acquisition Parameters
Date_ 20130225
Time 20.38
INSTRUM DPX400
PROBHD 5 mm QNP 1H/13
PULPROG deptq_135
TD 65536
SOLVENT CDCl3
NS 512
DS 4
SWH 23980.814 Hz
FIDRES 0.365918 Hz
AQ 1.3664756 sec
RG 18390.4
DW 20.850 usec
DE 6.00 usec
TE 293.2 K
CNST2 145.0000000
D1 2.00000000 sec
d2 0.00344828 sec
D11 0.03000000 sec
DELTA 0.00001082 sec
======== CHANNEL f1 ========
NUC1 13C
P1 8.50 usec
P2 17.00 usec
PL1 -2.60 dB
SFO1 100.6228300 MHz
======== CHANNEL f2 ========
CPDPRG2 waltz16
NUC2 1H
P3 10.00 usec
P4 20.00 usec
PCPD2 80.00 usec
PL2 -1.50 dB
PL12 17.50 dB
SFO2 400.1316000 MHz
F2 - Processing parameters
SI 32768
SF 100.6127690 MHz
WDW EM
SSB 0
LB 1.00 Hz
GB 0
PC 1.40
200
180
160
140
120
100
80
60
40
20
0
ppm

aet9-40-3

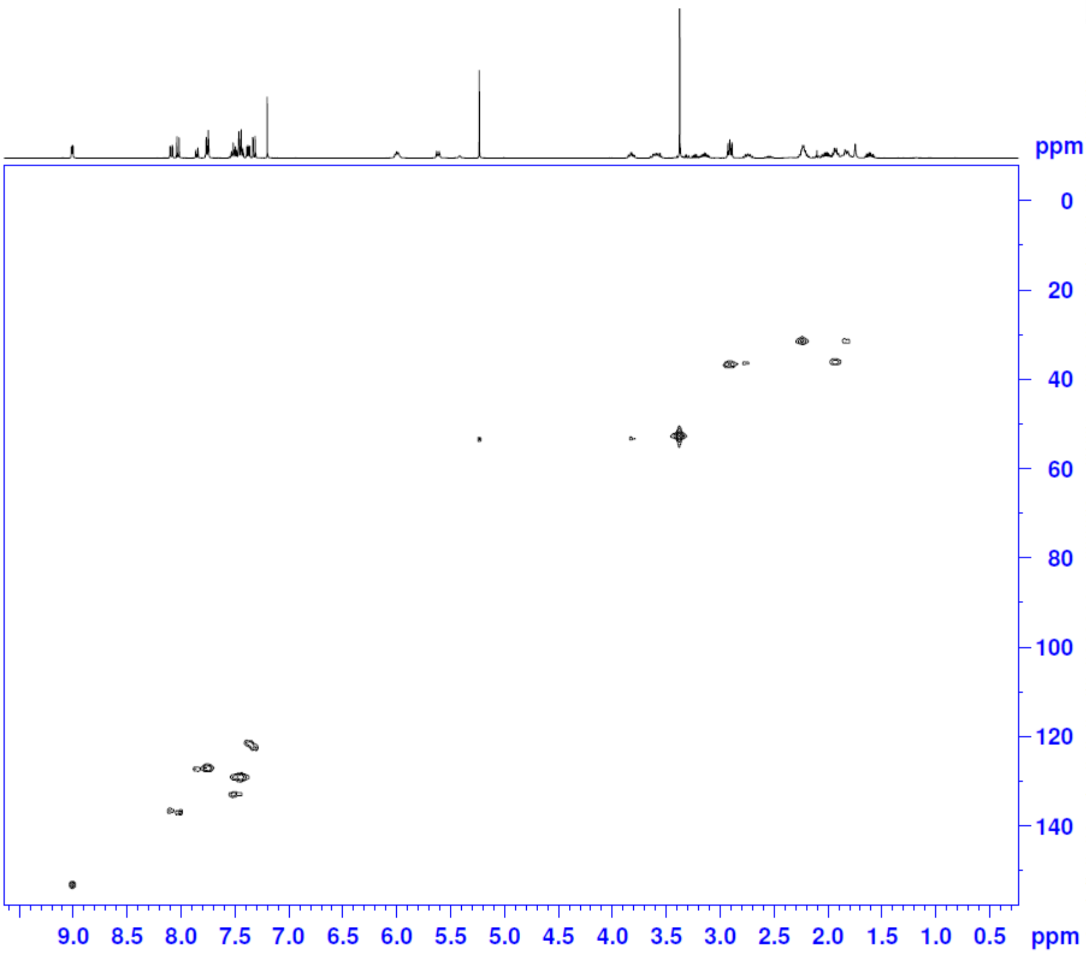


```
Current Data Parameters
NAME            aet9-40-3
EXPNO                  14
PROCNO                  1

F2 - Acquisition Parameters
Date_            20130225
Time                20.59
INSTRUM            DPX400
PROBHD   5 mm QNP 1H/13
PULPROG          hmqcgpqf
TD                   1024
SOLVENT             CDCl3
NS                      4
DS                     16
SWH              3765.060 Hz
FIDRES           3.676817 Hz
AQ              0.1360372 sec
RG                 5792.6
DW                132.800 usec
DE                   6.00 usec
TE                  292.2 K
CNST2         145.0000000
d0             0.00000300 sec
D1             1.49078405 sec
d2             0.00344828 sec
d12            0.00002000 sec
d13            0.00000400 sec
D16            0.00020000 sec
DELTA1         0.00222428 sec
IN0            0.00003000 sec

======== CHANNEL f1 ========
NUC1                   1H
P1                  10.00 usec
p2                  20.00 usec
PL1                 -1.50 dB
SFO1          400.1320094 MHz

======== CHANNEL f2 ========
CPDPRG2              garp
NUC2                  13C
P3                   8.00 usec
PCPD2               74.00 usec
PL2                 -3.00 dB
PL12                18.00 dB
SFO2          100.6203140 MHz

===== GRADIENT CHANNEL =====
GPNAM1           SINE.100
GPNAM2           SINE.100
GPNAM3           SINE.100
GPZ1                50.00 %
GPZ2                30.00 %
GPZ3                40.10 %
P16               1000.00 usec

F1 - Acquisition parameters
ND0                     2
TD                    128
SFO1             100.6203 MHz
FIDRES         130.208328 Hz
SW                165.639 ppm
FnMODE                 QF

F2 - Processing parameters
SI                   1024
SF            400.1300336 MHz
WDW                 QSINE
SSB                     2
LB                   0.00 Hz
GB                      0
PC                   1.40

F1 - Processing parameters
SI                   1024
MC2                    QF
SF            100.6127690 MHz
WDW                 QSINE
SSB                     2
LB                   0.00 Hz
GB                      0
```

Methyl (*S*)-4-((1*r,3*R**)-3-(2-(1,8-naphthyridin-2-yl)ethyl)cyclobutane-1-carboxamido)-2-(2,4,6-trimethylphenylsulfonamido)butanoate [Compound 20]**

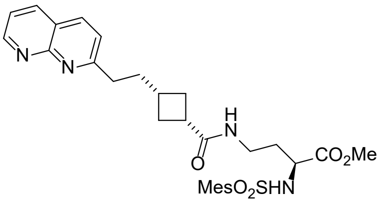

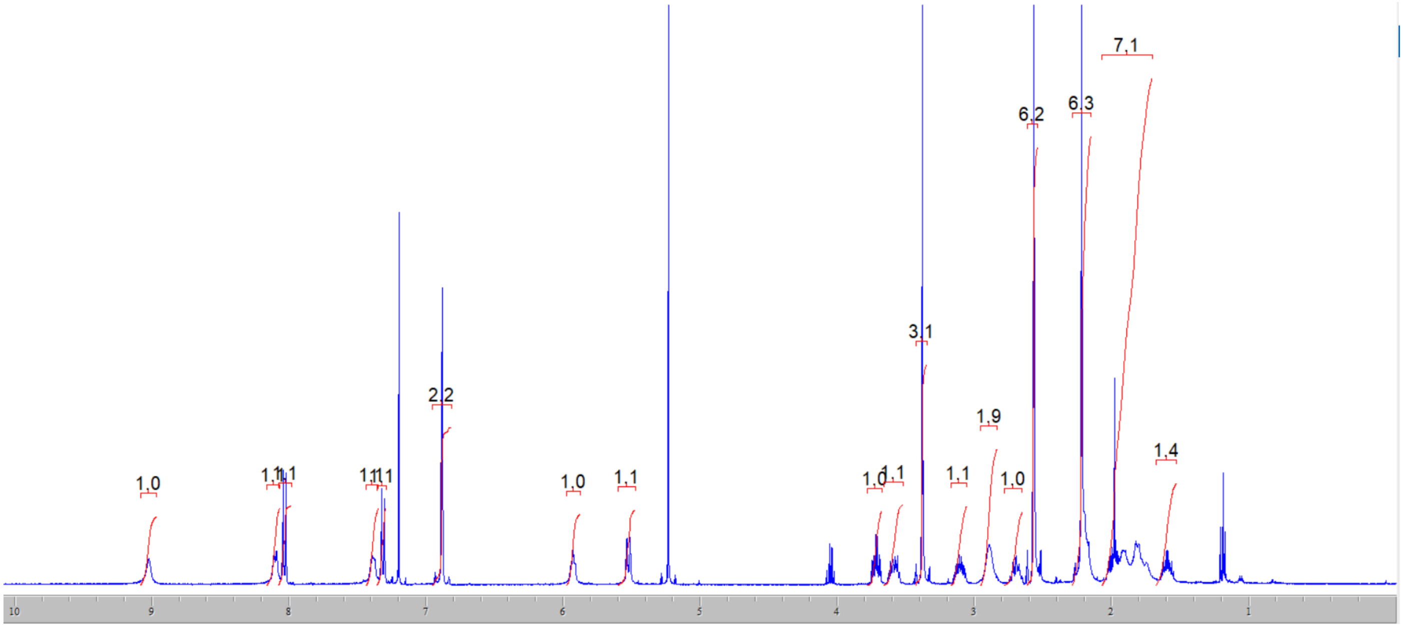

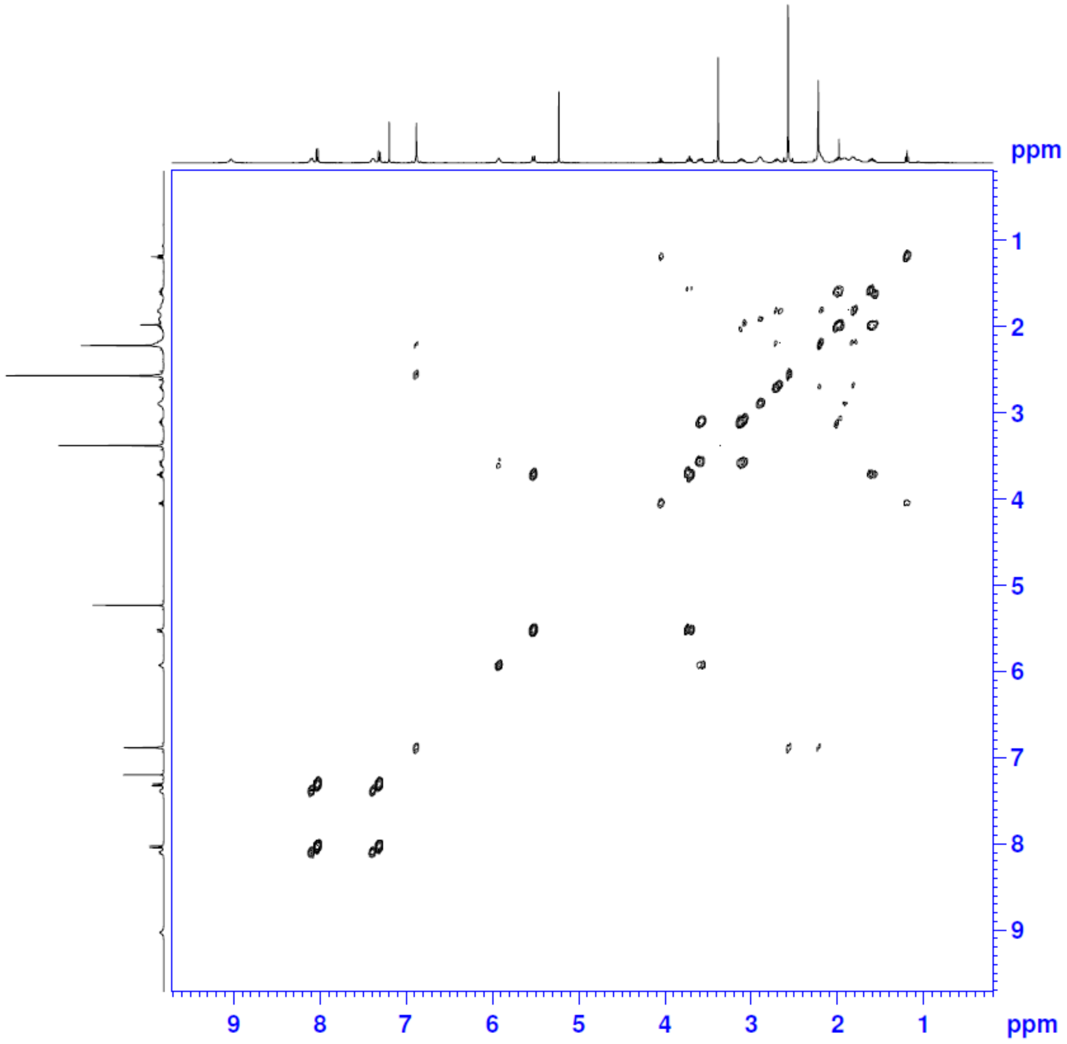


```
Current Data Parameters
NAME             aet9-19-1
EXPNO                   12
PROCNO                   1

F2 - Acquisition Parameters
Date_             20121031
Time                 19.04
INSTRUM             DPX400
PROBHD   5 mm QNP 1H/13
PULPROG        cosygpmfqf
TD                    2048
SOLVENT              CDCl3
NS                       4
DS                       8
SWH               3810.976 Hz
FIDRES            1.860828 Hz
AQ               0.2687476 sec
RG                  5792.6
DW                 131.200 usec
DE                    6.00 usec
TE                   292.2 K
D0              0.00000300 sec
D1              1.92340505 sec
d13             0.00000400 sec
D16             0.00020000 sec
IN0             0.00026240 sec

======== CHANNEL f1 ========
NUC1                    1H
P1                   10.00 usec
PL1                  -1.50 dB
SFO1           400.1320160 MHz

====== GRADIENT CHANNEL =====
GPNAM1           SINE.100
GPNAM2           SINE.100
GPNAM3           SINE.100
GPZ1                16.00 %
GPZ2                12.00 %
GPZ3                40.00 %
P16               1000.00 usec

F1 - Acquisition parameters
ND0                      1
TD                     128
SFO1               400.132 MHz
FIDRES           29.773247 Hz
SW                   9.524 ppm
FnMODE                  QF

F2 - Processing parameters
SI                    1024
SF             400.1300337 MHz
WDW                   SINE
SSB                      0
LB                    0.00 Hz
GB                       0
PC                    1.40

F1 - Processing parameters
SI                    1024
MC2                     QF
SF             400.1300337 MHz
WDW                   SINE
SSB                      0
LB                    0.00 Hz
GB                       0
```

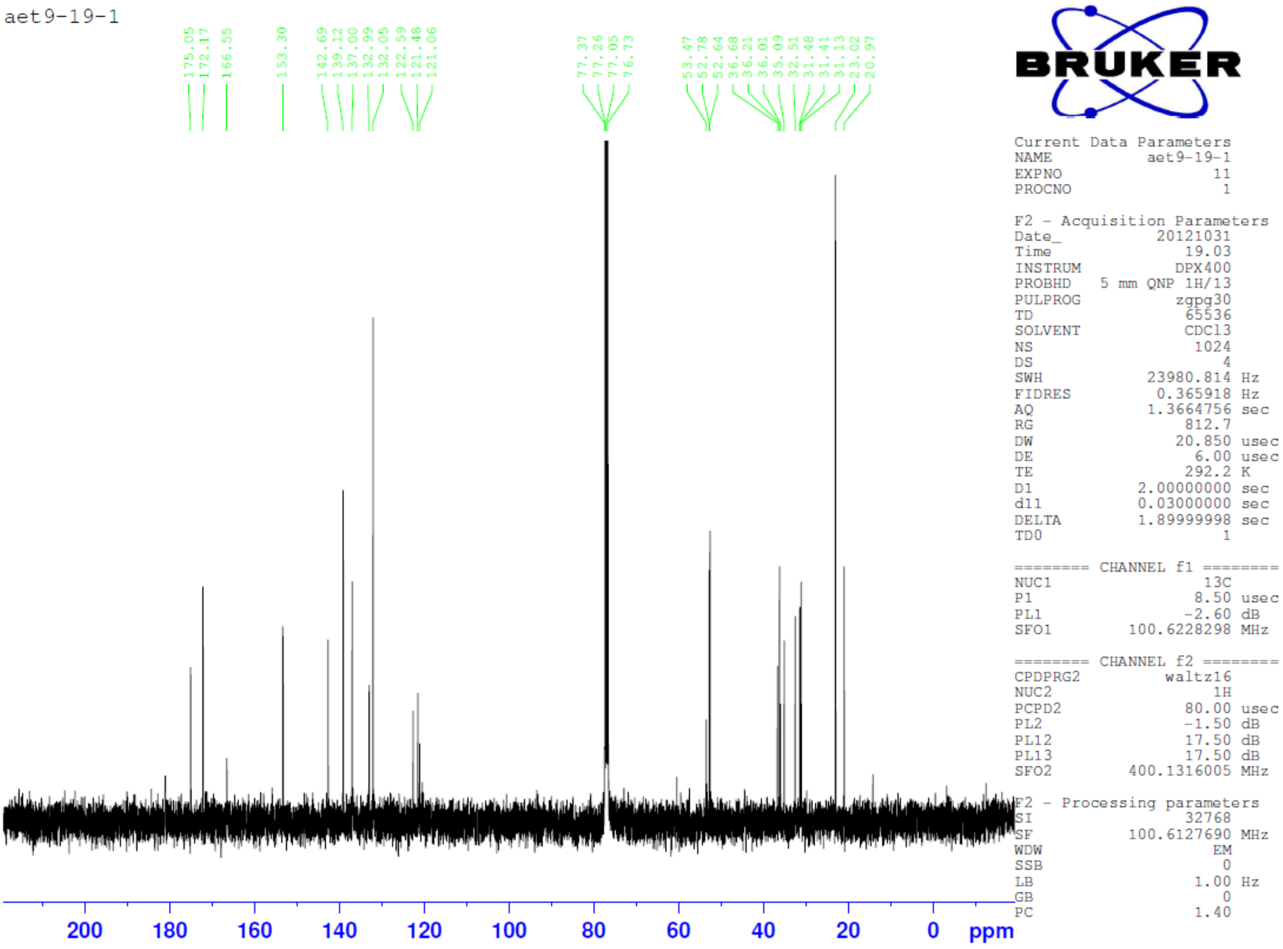
aet9-19-1
175.05 172.17 166.55 153.30 142.69 139.12 137.00 132.99 132.05 122.59 121.48 121.06 77.37 77.26 77.05 76.73 53.47 52.78 52.64 36.68 36.21 36.01 35.09 32.51 31.48 31.41 31.13 23.02 20.97
BRUKER
Current Data Parameters
NAME aet9-19-1
EXPNO 11
PROCNO 1
F2 - Acquisition Parameters
Date_ 20121031
Time 19.03
INSTRUM DPX400
PROBHD 5 mm QNP 1H/13
PULPROG zgpg30
TD 65536
SOLVENT CDCl3
NS 1024
DS 4
SWH 23980.814 Hz
FIDRES 0.365918 Hz
AQ 1.3664756 sec
RG 812.7
DW 20.850 usec
DE 6.00 usec
TE 292.2 K
D1 2.00000000 sec
d11 0.03000000 sec
DELTA 1.89999998 sec
TD0 1
======== CHANNEL f1 ========
NUC1 13C
P1 8.50 usec
PL1 -2.60 dB
SFO1 100.6228298 MHz
======== CHANNEL f2 ========
CPDPRG2 waltz16
NUC2 1H
PCPD2 80.00 usec
PL2 -1.50 dB
PL12 17.50 dB
PL13 17.50 dB
SFO2 400.1316005 MHz
F2 - Processing parameters
SI 32768
SF 100.6127690 MHz
WDW EM
SSB 0
LB 1.00 Hz
GB 0
PC 1.40
200 180 160 140 120 100 80 60 40 20 0 ppm

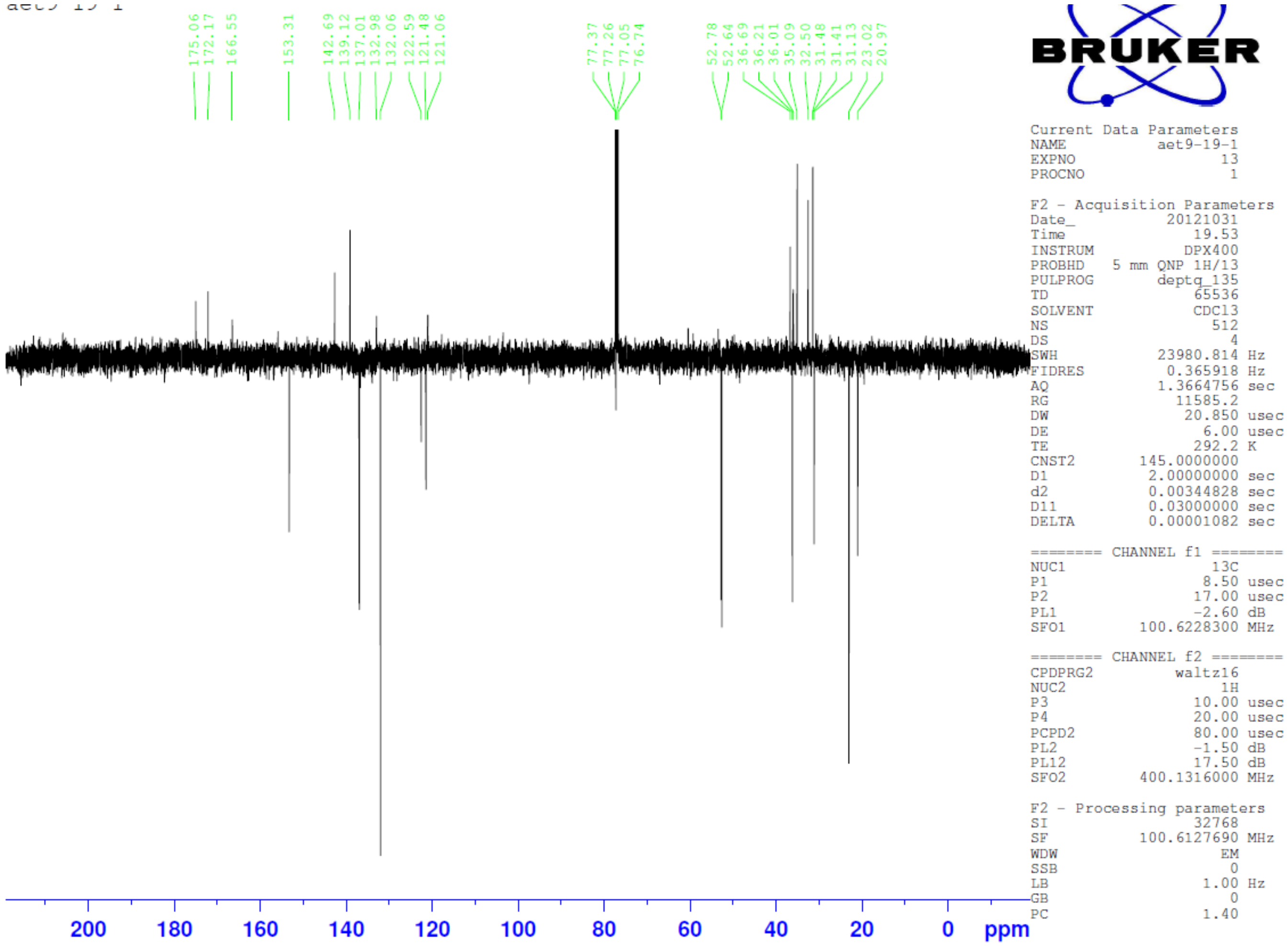
175.06 172.17 166.55 153.31 142.69 139.12 137.01 132.98 132.06 122.59 121.48 121.06 77.37 77.26 77.05 76.74 52.78 52.64 36.69 36.21 36.01 35.09 32.50 31.48 31.41 31.13 23.02 20.97
BRUKER
Current Data Parameters
NAME aet9-19-1
EXPNO 13
PROCNO 1
F2 - Acquisition Parameters
Date_ 20121031
Time 19.53
INSTRUM DPX400
PROBHD 5 mm QNP 1H/13
PULPROG deptq_135
TD 65536
SOLVENT CDCl3
NS 512
DS 4
SWH 23980.814 Hz
FIDRES 0.365918 Hz
AQ 1.3664756 sec
RG 11585.2
DW 20.850 usec
DE 6.00 usec
TE 292.2 K
CNST2 145.0000000
D1 2.00000000 sec
d2 0.00344828 sec
D11 0.03000000 sec
DELTA 0.00001082 sec
======== CHANNEL f1 ========
NUC1 13C
P1 8.50 usec
P2 17.00 usec
PL1 -2.60 dB
SFO1 100.6228300 MHz
======== CHANNEL f2 ========
CPDPRG2 waltz16
NUC2 1H
P3 10.00 usec
P4 20.00 usec
PCPD2 80.00 usec
PL2 -1.50 dB
PL12 17.50 dB
SFO2 400.1316000 MHz
F2 - Processing parameters
SI 32768
SF 100.6127690 MHz
WDW EM
SSB 0
LB 1.00 Hz
GB 0
PC 1.40
200 180 160 140 120 100 80 60 40 20 0 ppm

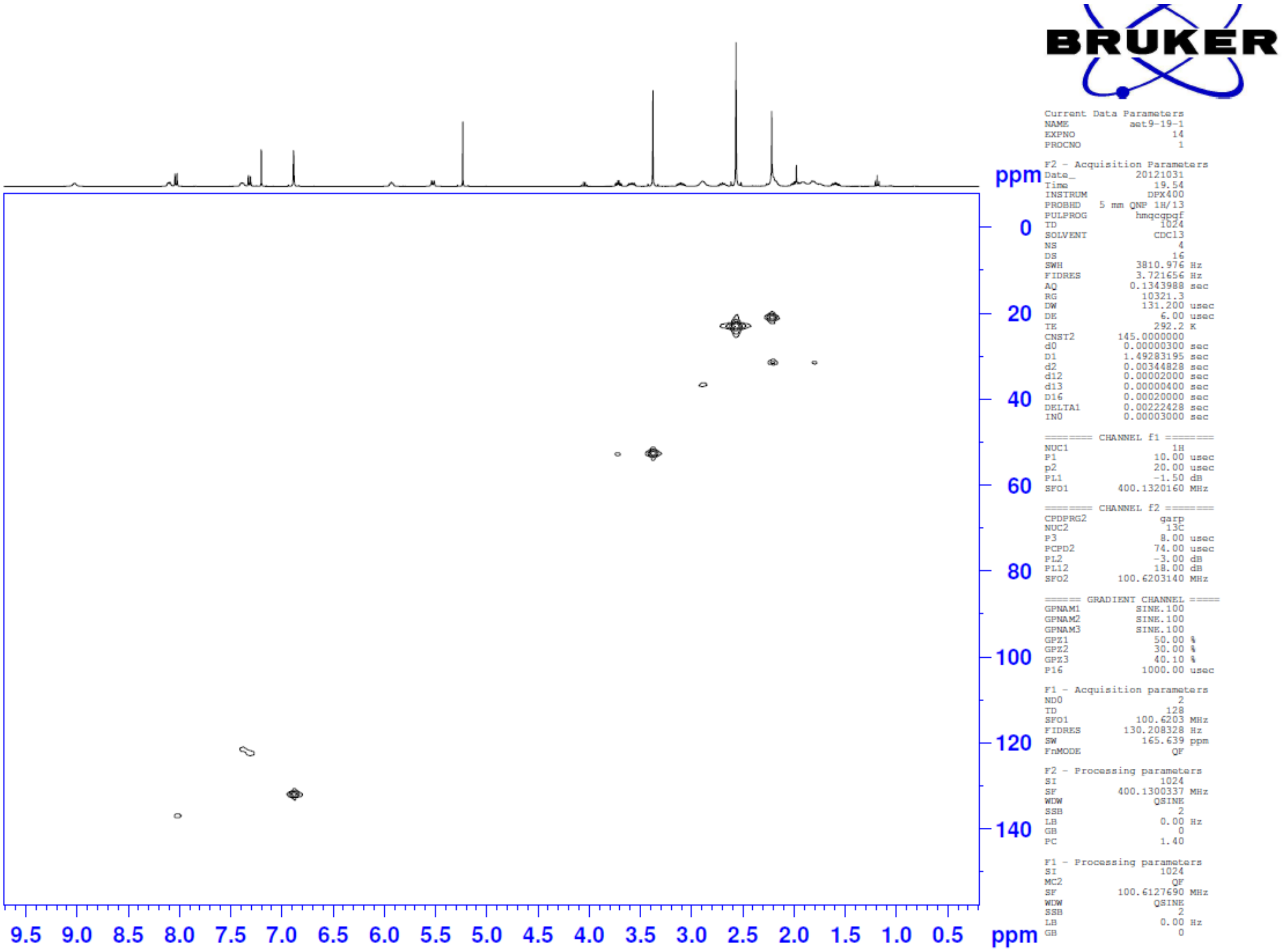
BRUKER
Current Data Parameters
NAME aet9-19-1
EXPNO 14
PROCNO 1
F2 - Acquisition Parameters
Date_ 20121031
Time 19.54
INSTRUM DPX400
PROBHD 5 mm QNP 1H/13
PULPROG hmqcgpqf
TD 1024
SOLVENT CDCl3
NS 4
DS 16
SWH 3810.976 Hz
FIDRES 3.721656 Hz
AQ 0.1343988 sec
RG 10321.3
DW 131.200 usec
DE 6.00 usec
TE 292.2 K
CNST2 145.0000000
d0 0.00000300 sec
D1 1.49283195 sec
d2 0.00344828 sec
d12 0.00002000 sec
d13 0.00000400 sec
D16 0.00020000 sec
DELTA1 0.00222428 sec
IN0 0.00003000 sec
======= CHANNEL f1 =======
NUC1 1H
P1 10.00 usec
p2 20.00 usec
PL1 -1.50 dB
SFO1 400.1320160 MHz
======= CHANNEL f2 =======
CPDPRG2 garp
NUC2 13C
P3 8.00 usec
PCPD2 74.00 usec
PL2 -3.00 dB
PL12 18.00 dB
SFO2 100.6203140 MHz
===== GRADIENT CHANNEL =====
GPNAM1 SINE.100
GPNAM2 SINE.100
GPNAM3 SINE.100
GPZ1 50.00 %
GPZ2 30.00 %
GPZ3 40.10 %
P16 1000.00 usec
F1 - Acquisition parameters
ND0 2
TD 128
SFO1 100.6203 MHz
FIDRES 130.208328 Hz
SW 165.639 ppm
FnMODE QF
F2 - Processing parameters
SI 1024
SF 400.1300337 MHz
WDW QSINE
SSB 2
LB 0.00 Hz
GB 0
PC 1.40
F1 - Processing parameters
SI 1024
MC2 QF
SF 100.6127690 MHz
WDW QSINE
SSB 2
LB 0.00 Hz
GB 0
ppm
0
20
40
60
80
100
120
140
9.5 9.0 8.5 8.0 7.5 7.0 6.5 6.0 5.5 5.0 4.5 4.0 3.5 3.0 2.5 2.0 1.5 1.0 0.5
ppm

Methyl (*S*)-2-(phenylsulfonamido)-4-((1*s,3*R**)-3-((5,6,7,8-tetrahydro-1,8-naphthyridin-2-yl)methyl)cyclobutane-1-carboxamido)butanoate [Compound 21]**

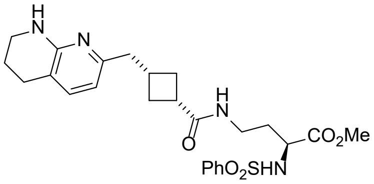

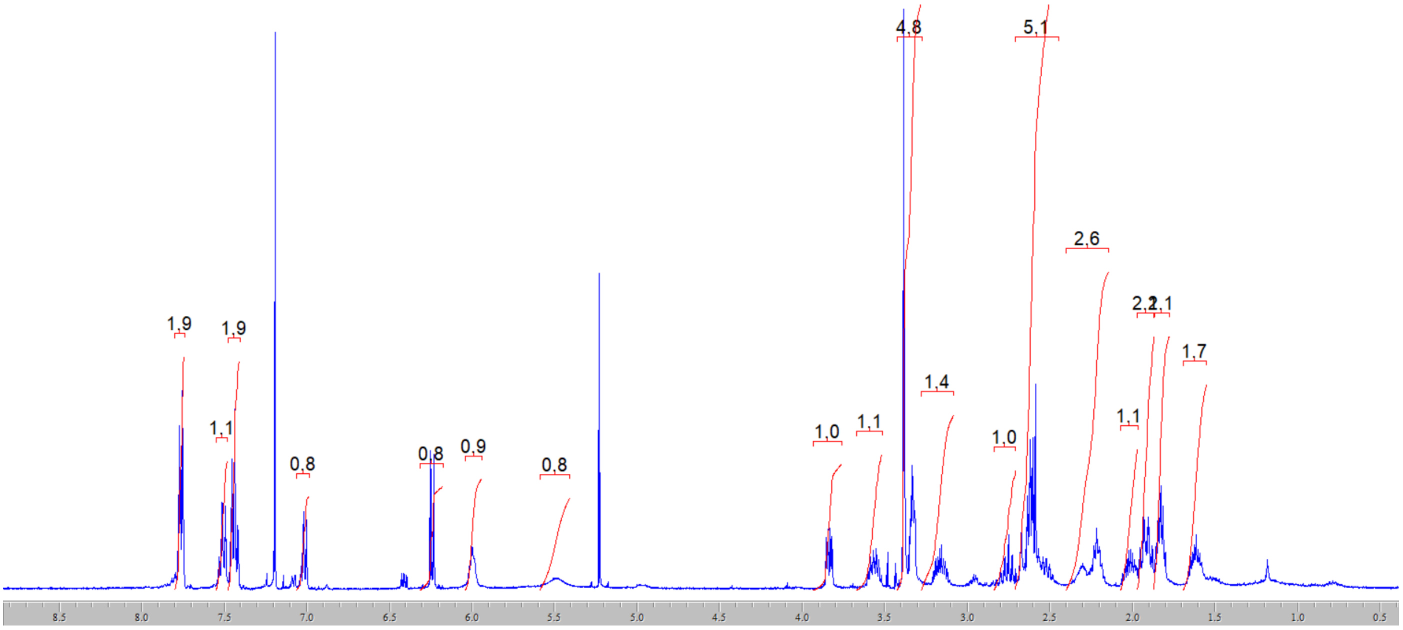

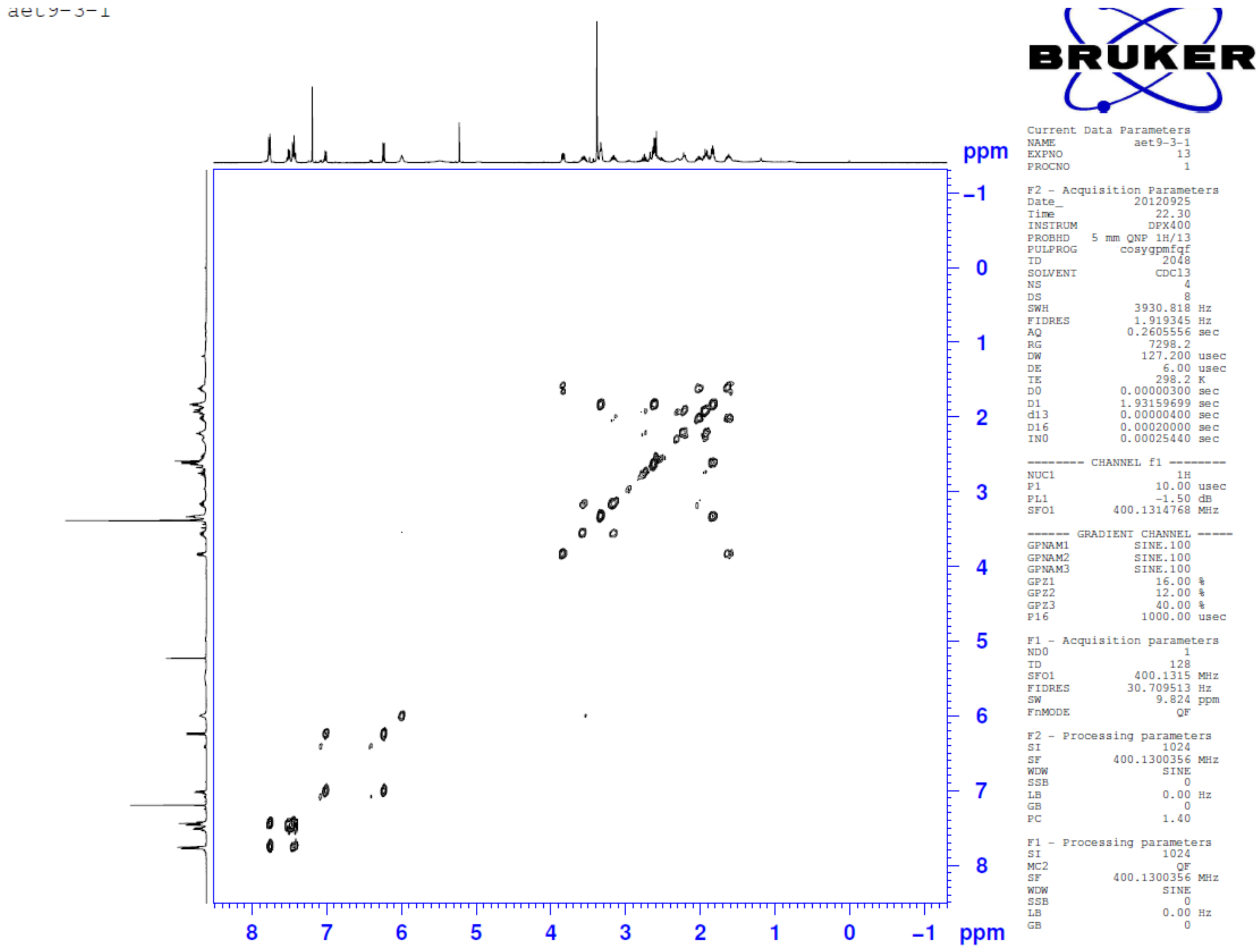

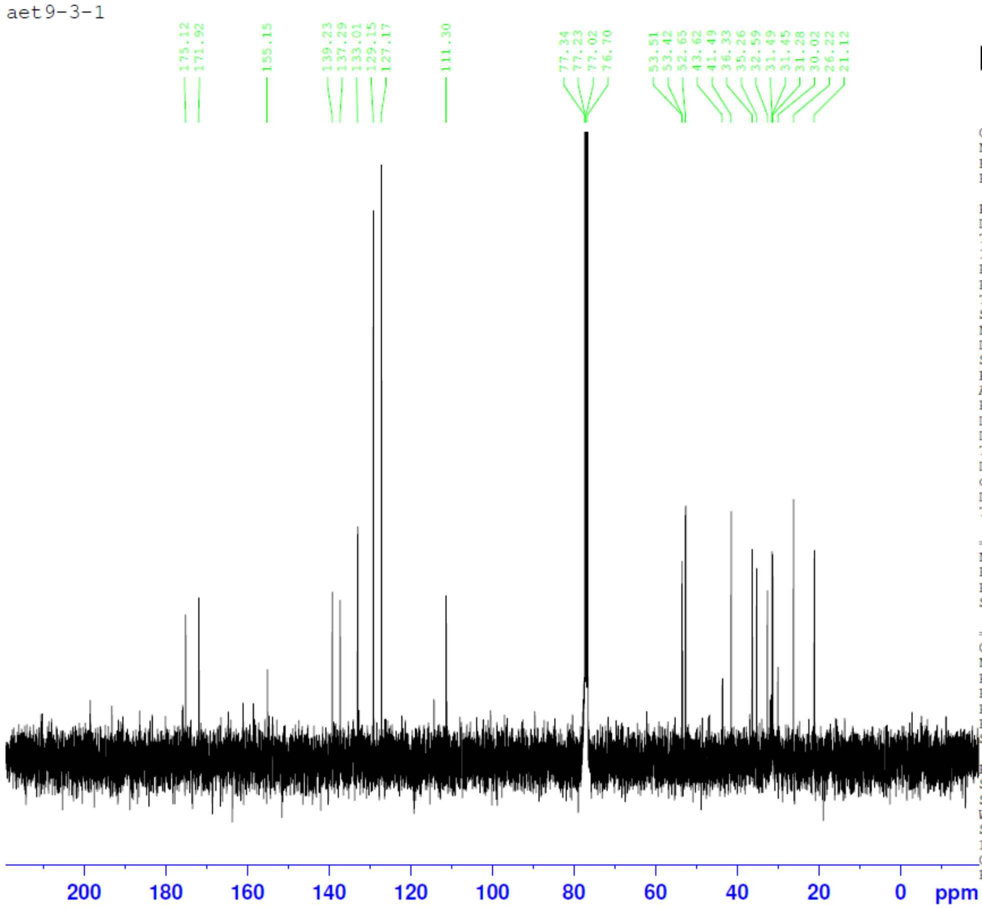

Current Data Parameters
NAME aet9-3-1
EXPNO 11
PROCNO 1

F2 - Acquisition Parameters
Date_ 20120925
Time 21.59
INSTRUM DPX400
PROBHD 5 mm QNP 1H/13
PULPROG zgpg30
TD 65536
SOLVENT CDCl3
NS 1024
DS 4
SWH 23980.814 Hz
FIDRES 0.365918 Hz
AQ 1.3664756 sec
RG 912.3
DW 20.850 usec
DE 6.00 usec
TE 298.2 K
D1 2.00000000 sec
d11 0.03000000 sec
DELTA 1.89999998 sec
TD0 1

======== CHANNEL f1 ========
NUC1 13C
P1 8.50 usec
PL1 -2.60 dB
SFO1 100.6228298 MHz

======== CHANNEL f2 ========
CPDPRG2 waltz16
NUC2 1H
PCPD2 80.00 usec
PL2 -1.50 dB
PL12 17.50 dB
PL13 17.50 dB
SFO2 400.1316005 MHz

F2 - Processing parameters
SI 32768
SF 100.6127690 MHz
WDW EM
SSB 0
LB 1.00 Hz
GB 0
PC 1.40

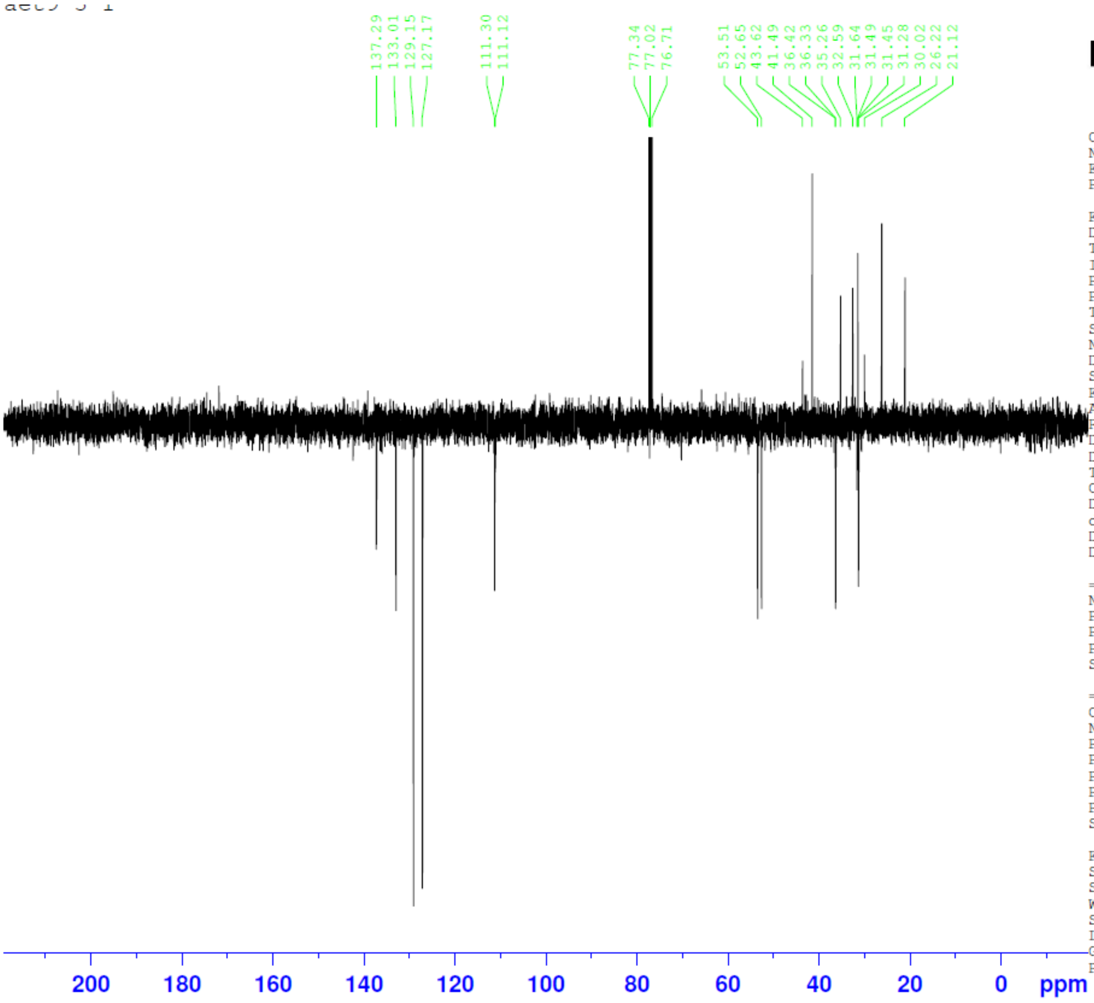

Current Data Parameters
NAME aet9-3-1
EXPNO 12
PROCNO 1

F2 - Acquisition Parameters
Date_ 20120925
Time 22.29
INSTRUM DPX400
PROBHD 5 mm QNP 1H/13
PULPROG deptq_135
TD 65536
SOLVENT CDCl3
NS 512
DS 4
SWH 23980.814 Hz
FIDRES 0.365918 Hz
AQ 1.3664756 sec
RG 16384
DW 20.850 usec
DE 6.00 usec
TE 298.2 K
CNST2 145.0000000
D1 2.00000000 sec
d2 0.00344828 sec
D11 0.03000000 sec
DELTA 0.00001082 sec

======== CHANNEL f1 ========
NUC1 13C
P1 8.50 usec
P2 17.00 usec
PL1 -2.60 dB
SFO1 100.6228300 MHz

======== CHANNEL f2 ========
CPDPRG2 waltz16
NUC2 1H
P3 10.00 usec
P4 20.00 usec
PCPD2 80.00 usec
PL2 -1.50 dB
PL12 17.50 dB
SFO2 400.1316000 MHz

F2 - Processing parameters
SI 32768
SF 100.6127690 MHz
WDW EM
SSB 0
LB 1.00 Hz
GB 0
PC 1.40

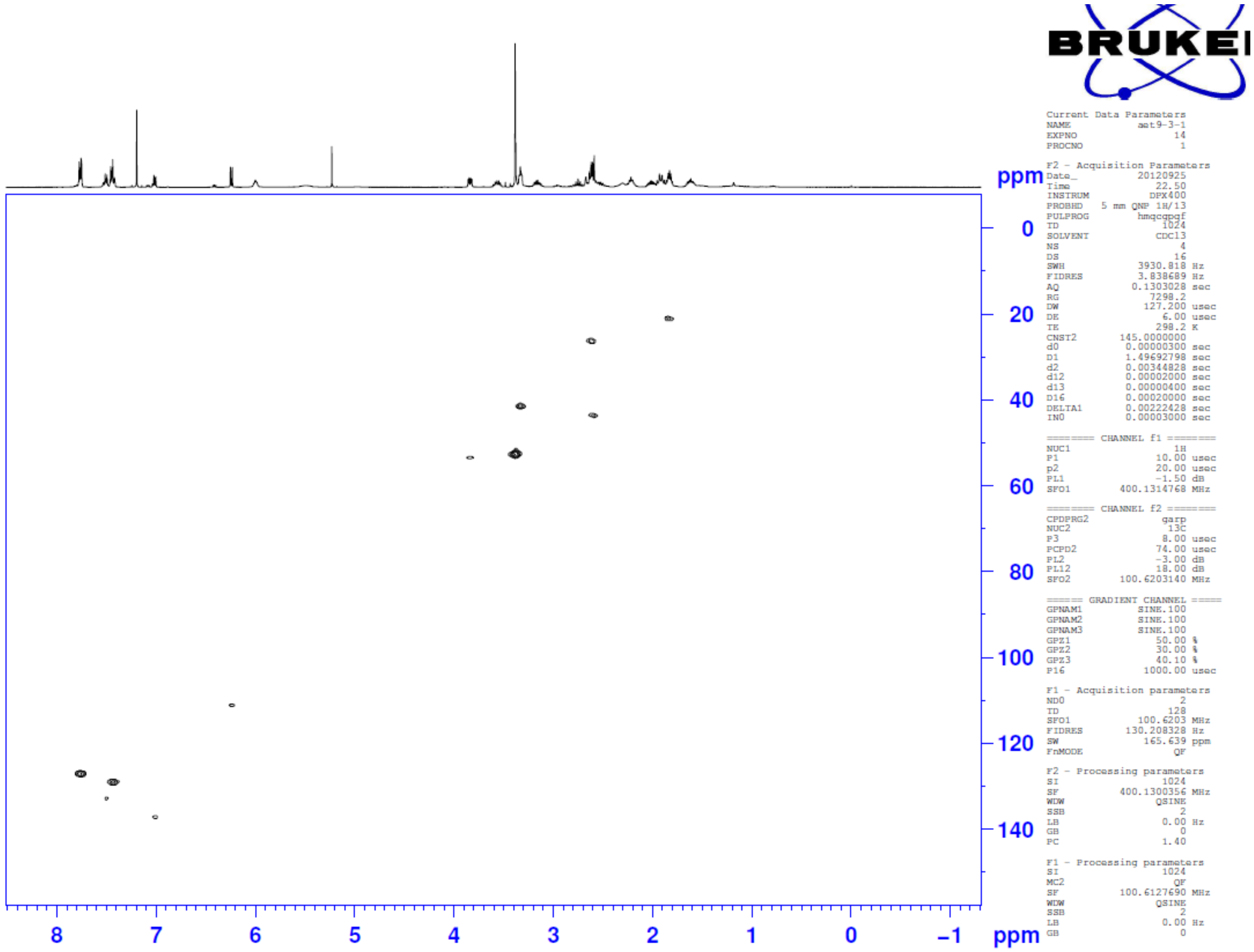
ppm
0
20
40
60
80
100
120
140
8
7
6
5
4
3
2
1
0
−1
ppm
Current Data Parameters
NAME aet9-3-1
EXPNO 14
PROCNO 1
F2 - Acquisition Parameters
Date_ 20120925
Time 22.50
INSTRUM DPX400
PROBHD 5 mm QNP 1H/13
PULPROG hmqcgpqf
TD 1024
SOLVENT CDCl3
NS 4
DS 16
SWH 3930.818 Hz
FIDRES 3.838689 Hz
AQ 0.1303028 sec
RG 7298.2
DW 127.200 usec
DE 6.00 usec
TE 298.2 K
CNST2 145.0000000
d0 0.00000300 sec
D1 1.49692798 sec
d2 0.00344828 sec
d12 0.00002000 sec
d13 0.00000400 sec
D16 0.00020000 sec
DELTA1 0.00222428 sec
IN0 0.00003000 sec
======== CHANNEL f1 ========
NUC1 1H
P1 10.00 usec
p2 20.00 usec
PL1 -1.50 dB
SFO1 400.1314768 MHz
======== CHANNEL f2 ========
CPDPRG2 garp
NUC2 13C
P3 8.00 usec
PCPD2 74.00 usec
PL2 -3.00 dB
PL12 18.00 dB
SFO2 100.6203140 MHz
====== GRADIENT CHANNEL =====
GPNAM1 SINE.100
GPNAM2 SINE.100
GPNAM3 SINE.100
GPZ1 50.00 %
GPZ2 30.00 %
GPZ3 40.10 %
P16 1000.00 usec
F1 - Acquisition parameters
ND0 2
TD 128
SFO1 100.6203 MHz
FIDRES 130.208328 Hz
SW 165.639 ppm
FnMODE QF
F2 - Processing parameters
SI 1024
SF 400.1300356 MHz
WDW QSINE
SSB 2
LB 0.00 Hz
GB 0
PC 1.40
F1 - Processing parameters
SI 1024
MC2 QF
SF 100.6127690 MHz
WDW QSINE
SSB 2
LB 0.00 Hz
GB 0

Methyl (*S*)-4-((1*s,3*R**)-3-((5,6,7,8-tetrahydro-1,8-naphthyridin-2-yl) methyl)cyclobutane-1-carboxamido)-2-(2,4,6-trimethylphenylsulfonamido)butanoate [Compound 22]**

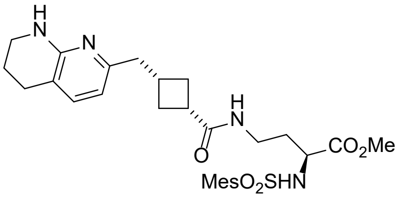

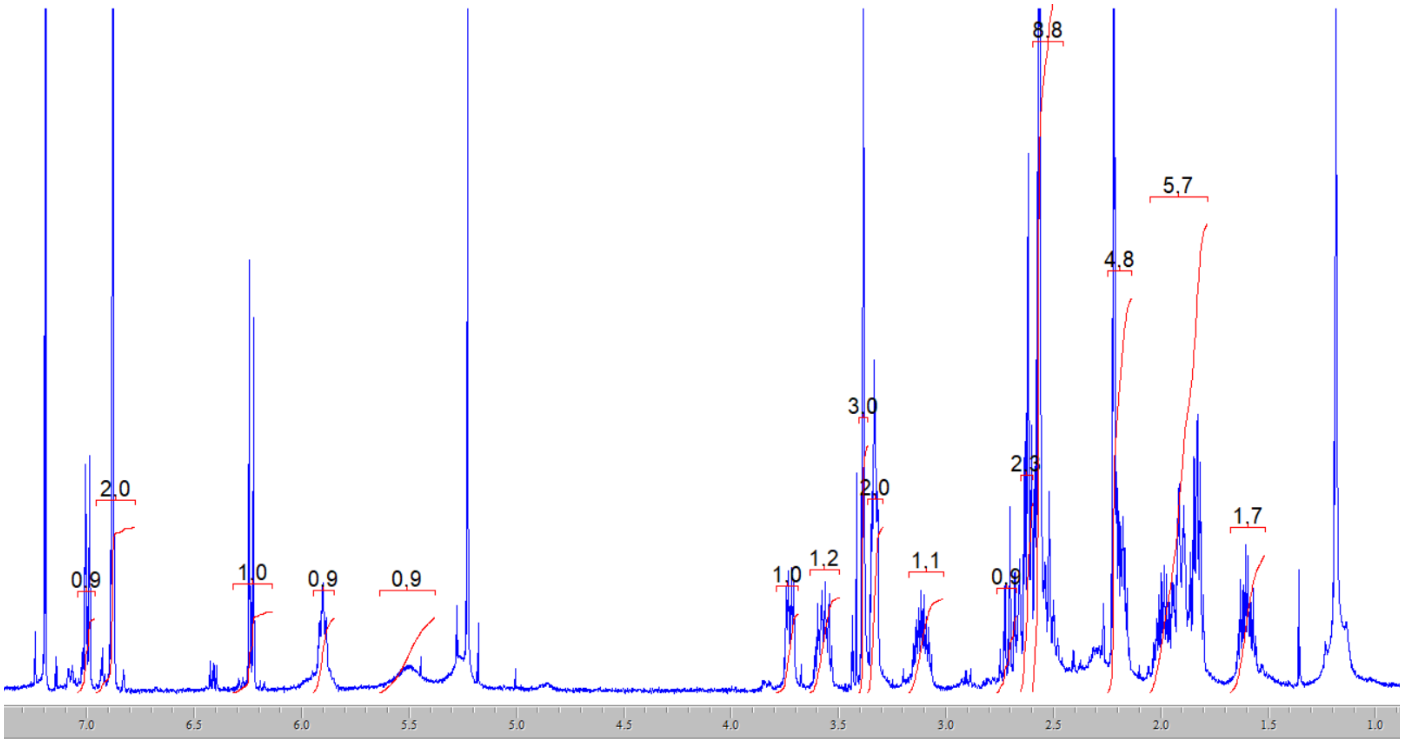

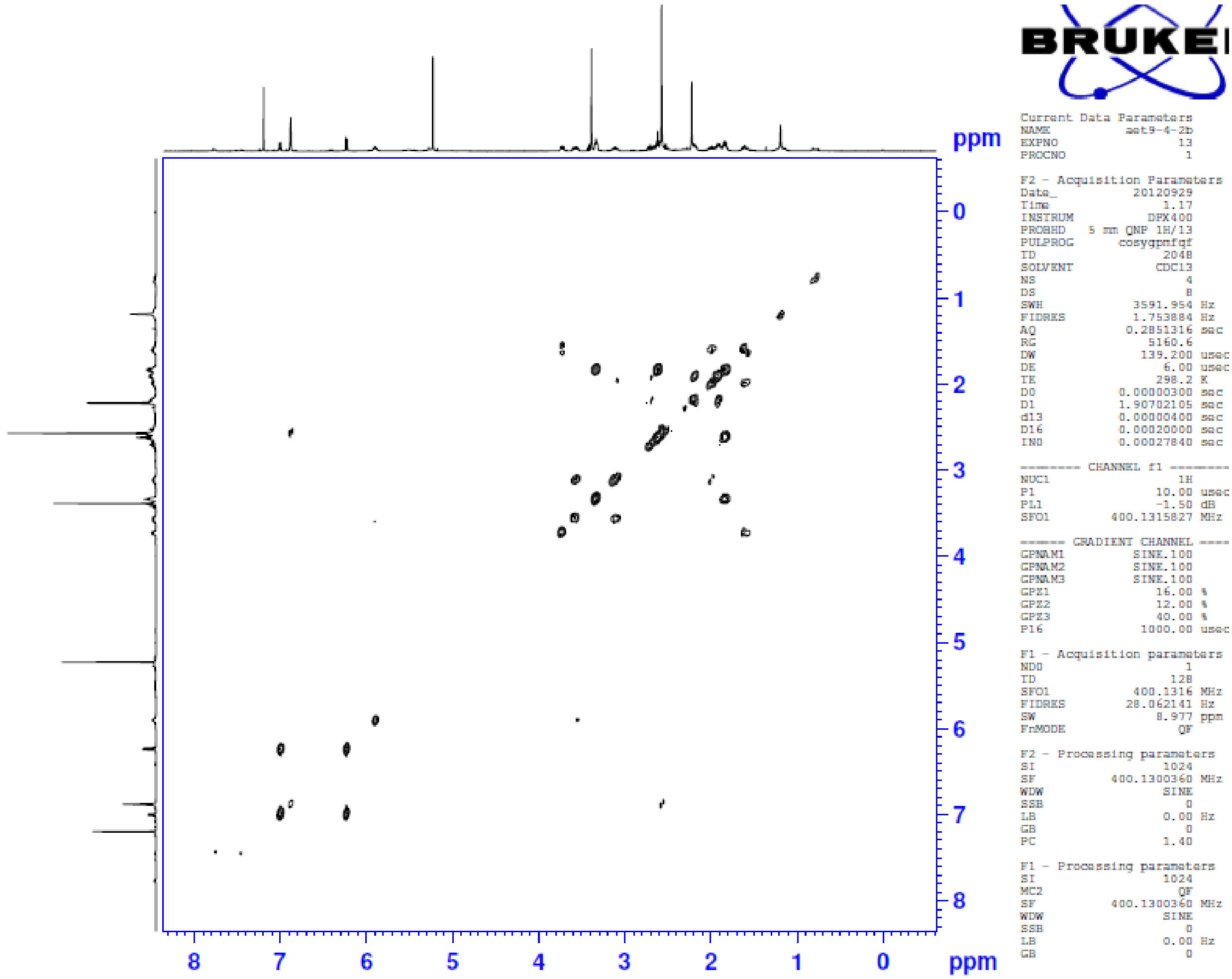

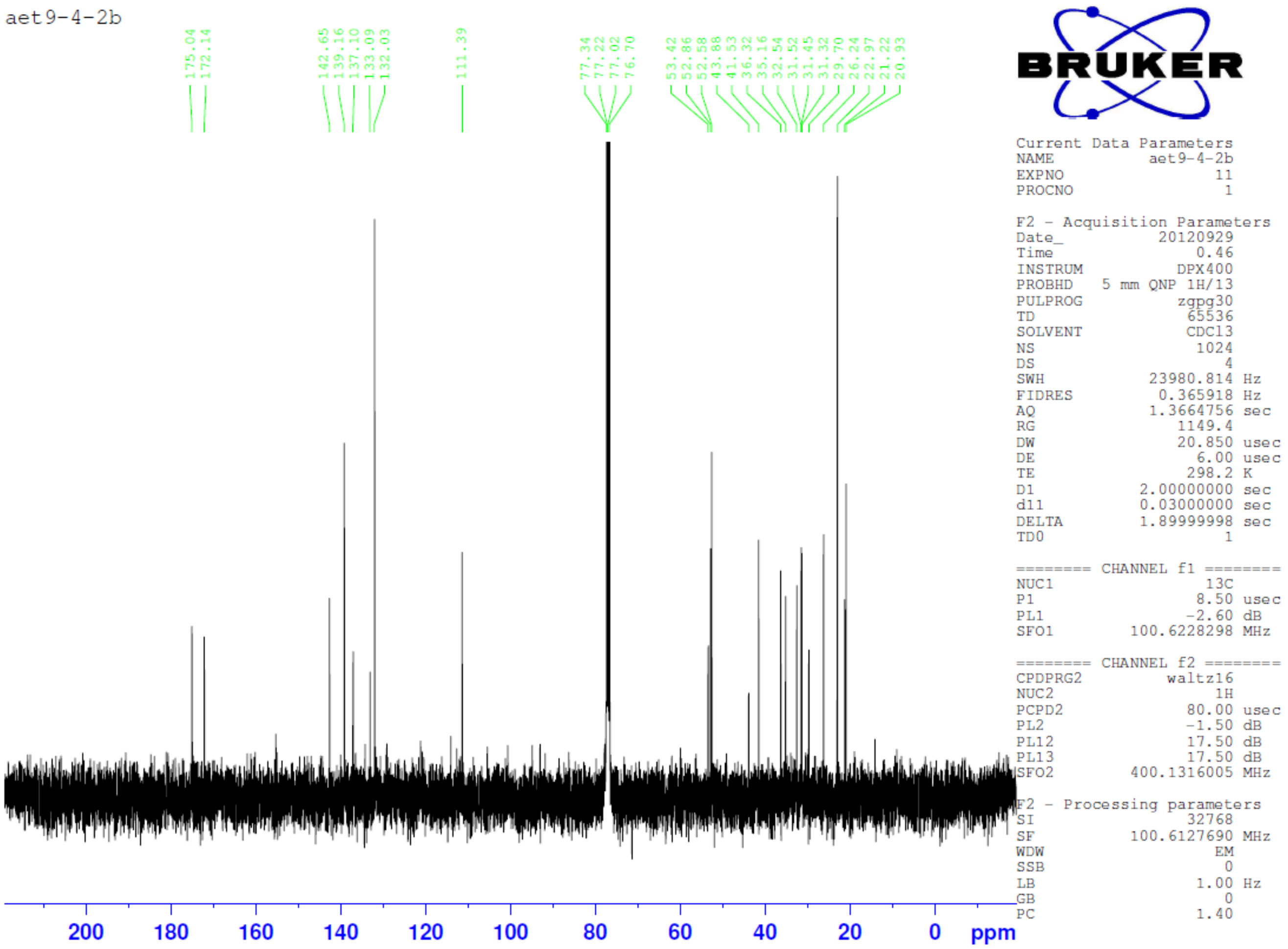
aet9-4-2b
175.04
172.14
142.65
139.16
137.10
133.09
132.03
111.39
77.34
77.22
77.02
76.70
53.42
52.86
52.58
43.88
41.53
36.32
35.16
32.54
31.52
31.45
31.32
29.70
26.24
22.97
21.22
20.93
BRUKER
Current Data Parameters
NAME aet9-4-2b
EXPNO 11
PROCNO 1
F2 - Acquisition Parameters
Date_ 20120929
Time 0.46
INSTRUM DPX400
PROBHD 5 mm QNP 1H/13
PULPROG zgpg30
TD 65536
SOLVENT CDCl3
NS 1024
DS 4
SWH 23980.814 Hz
FIDRES 0.365918 Hz
AQ 1.3664756 sec
RG 1149.4
DW 20.850 usec
DE 6.00 usec
TE 298.2 K
D1 2.00000000 sec
d11 0.03000000 sec
DELTA 1.89999998 sec
TD0 1
======== CHANNEL f1 ========
NUC1 13C
P1 8.50 usec
PL1 -2.60 dB
SFO1 100.6228298 MHz
======== CHANNEL f2 ========
CPDPRG2 waltz16
NUC2 1H
PCPD2 80.00 usec
PL2 -1.50 dB
PL12 17.50 dB
PL13 17.50 dB
SFO2 400.1316005 MHz
F2 - Processing parameters
SI 32768
SF 100.6127690 MHz
WDW EM
SSB 0
LB 1.00 Hz
GB 0
PC 1.40
200 180 160 140 120 100 80 60 40 20 0 ppm

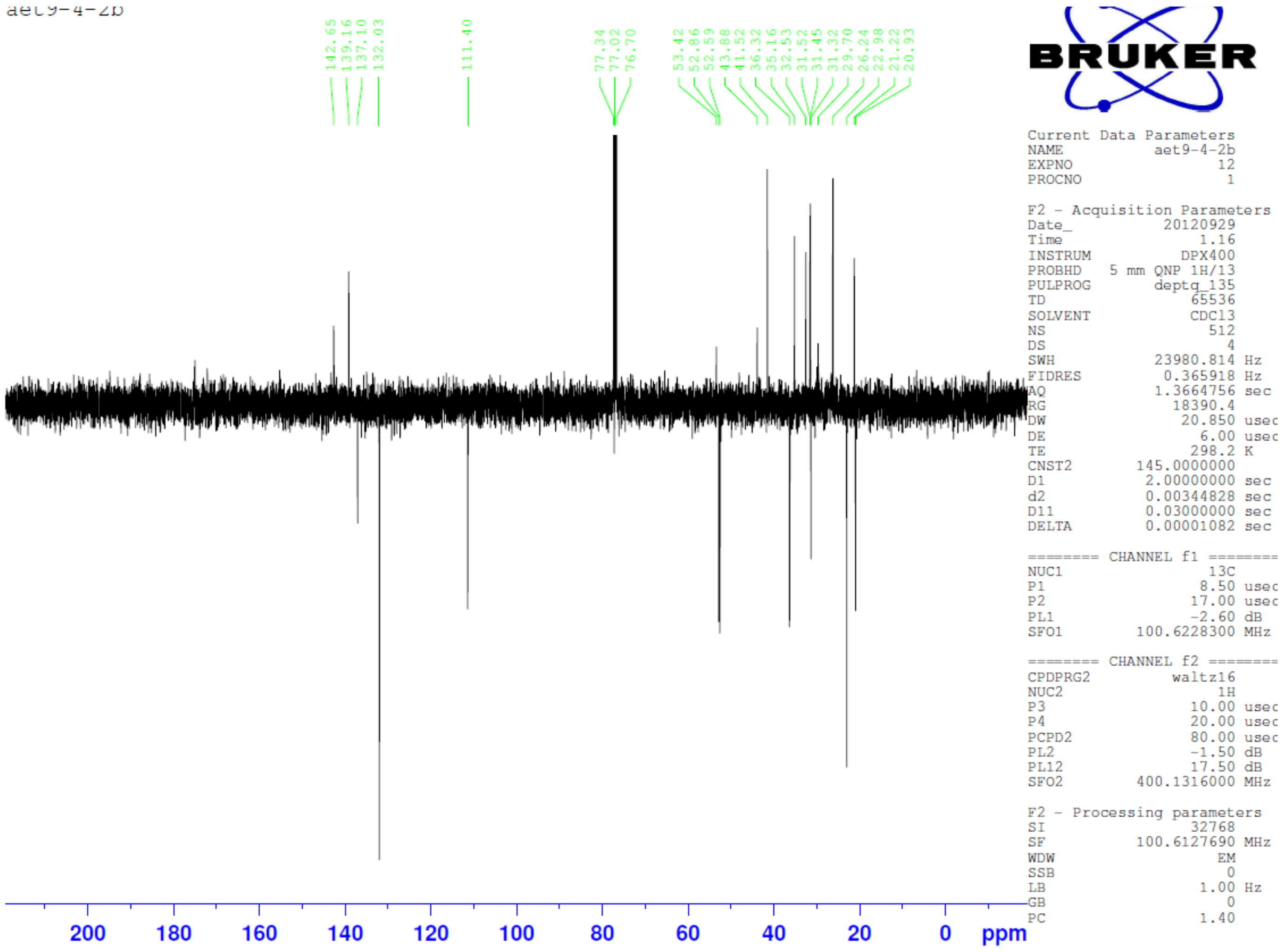
aet9-4-2b
142.65
139.16
137.10
132.03
111.40
77.34
77.02
76.70
53.42
52.86
52.59
43.88
41.52
36.32
35.16
32.53
31.52
31.45
31.32
29.70
26.24
22.98
21.22
20.93
BRUKER
Current Data Parameters
NAME aet9-4-2b
EXPNO 12
PROCNO 1
F2 - Acquisition Parameters
Date_ 20120929
Time 1.16
INSTRUM DPX400
PROBHD 5 mm QNP 1H/13
PULPROG deptq_135
TD 65536
SOLVENT CDCl3
NS 512
DS 4
SWH 23980.814 Hz
FIDRES 0.365918 Hz
AQ 1.3664756 sec
RG 18390.4
DW 20.850 usec
DE 6.00 usec
TE 298.2 K
CNST2 145.0000000
D1 2.00000000 sec
d2 0.00344828 sec
D11 0.03000000 sec
DELTA 0.00001082 sec
======== CHANNEL f1 ========
NUC1 13C
P1 8.50 usec
P2 17.00 usec
PL1 -2.60 dB
SFO1 100.6228300 MHz
======== CHANNEL f2 ========
CPDPRG2 waltz16
NUC2 1H
P3 10.00 usec
P4 20.00 usec
PCPD2 80.00 usec
PL2 -1.50 dB
PL12 17.50 dB
SFO2 400.1316000 MHz
F2 - Processing parameters
SI 32768
SF 100.6127690 MHz
WDW EM
SSB 0
LB 1.00 Hz
GB 0
PC 1.40
200 180 160 140 120 100 80 60 40 20 0 ppm

Methyl (*S*)-2-(phenylsulfonamido)-4-((1*r,3*R**)-3-(2-(5,6,7,8-tetrahydro-1,8-naphthyridin-2-yl)ethyl)cyclobutane-1-carboxamido)butanoate [Compound 23]**

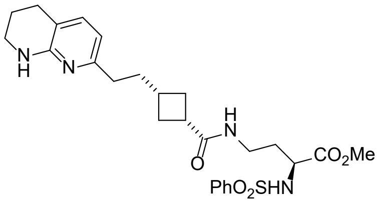

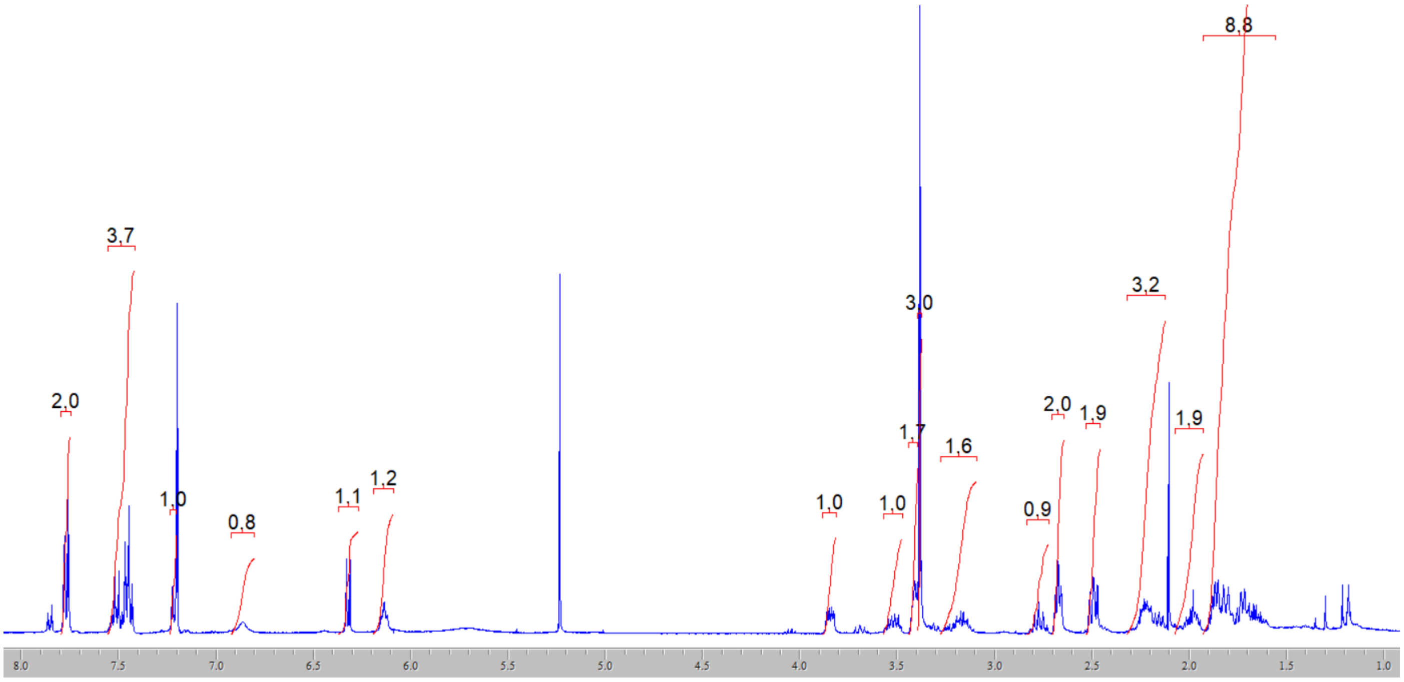

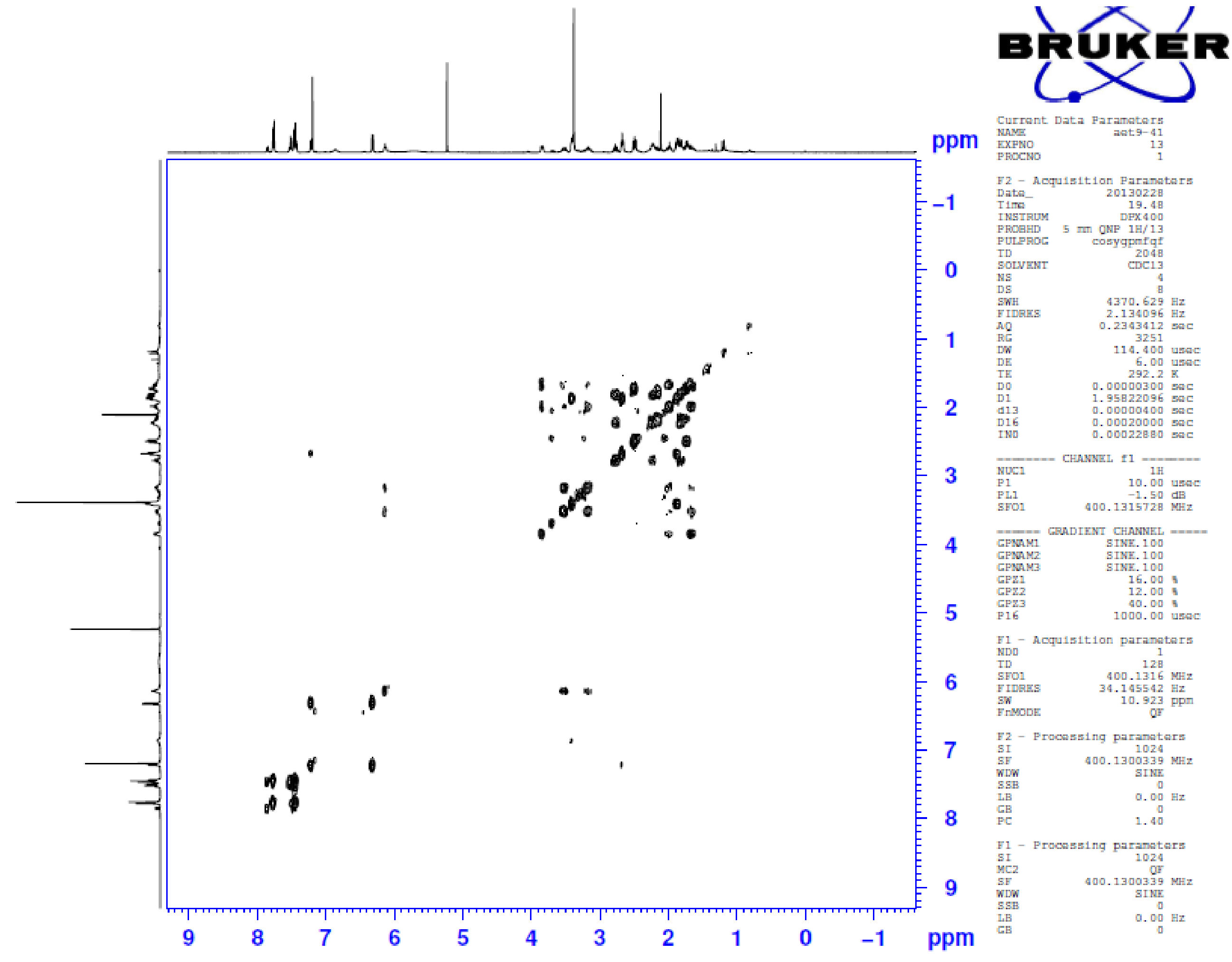

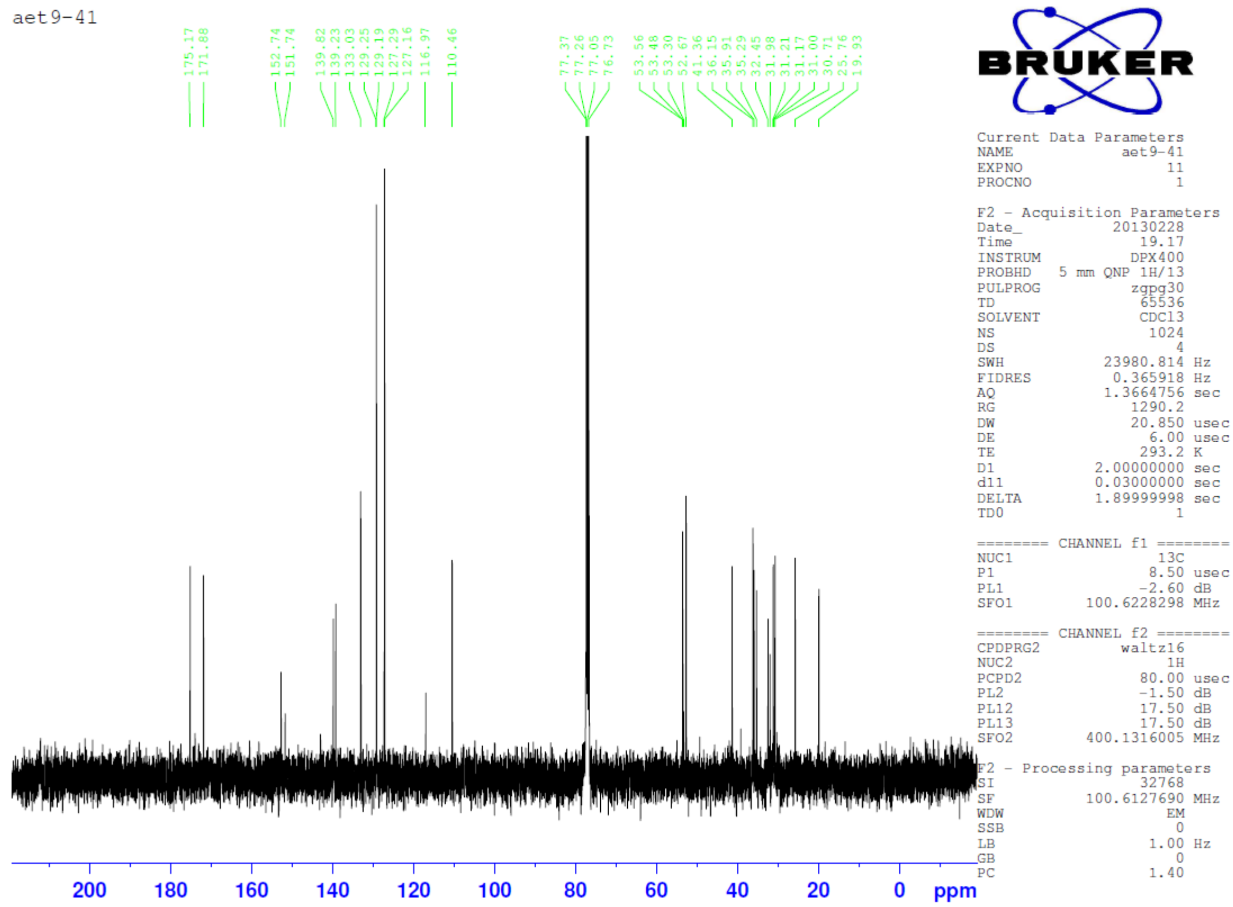
aet9-41
175.17
171.88
152.74
151.74
139.82
139.23
133.03
129.25
129.19
127.29
127.16
116.97
110.46
77.37
77.26
77.05
76.73
53.56
53.48
53.30
52.67
41.36
36.15
35.91
35.29
32.45
31.98
31.21
31.17
31.00
30.71
25.76
19.93
BRUKER
Current Data Parameters
NAME aet9-41
EXPNO 11
PROCNO 1
F2 - Acquisition Parameters
Date_ 20130228
Time 19.17
INSTRUM DPX400
PROBHD 5 mm QNP 1H/13
PULPROG zgpg30
TD 65536
SOLVENT CDCl3
NS 1024
DS 4
SWH 23980.814 Hz
FIDRES 0.365918 Hz
AQ 1.3664756 sec
RG 1290.2
DW 20.850 usec
DE 6.00 usec
TE 293.2 K
D1 2.00000000 sec
d11 0.03000000 sec
DELTA 1.89999998 sec
TD0 1
======== CHANNEL f1 ========
NUC1 13C
P1 8.50 usec
PL1 -2.60 dB
SFO1 100.6228298 MHz
======== CHANNEL f2 ========
CPDPRG2 waltz16
NUC2 1H
PCPD2 80.00 usec
PL2 -1.50 dB
PL12 17.50 dB
PL13 17.50 dB
SFO2 400.1316005 MHz
F2 - Processing parameters
SI 32768
SF 100.6127690 MHz
WDW EM
SSB 0
LB 1.00 Hz
GB 0
PC 1.40
200 180 160 140 120 100 80 60 40 20 0 ppm

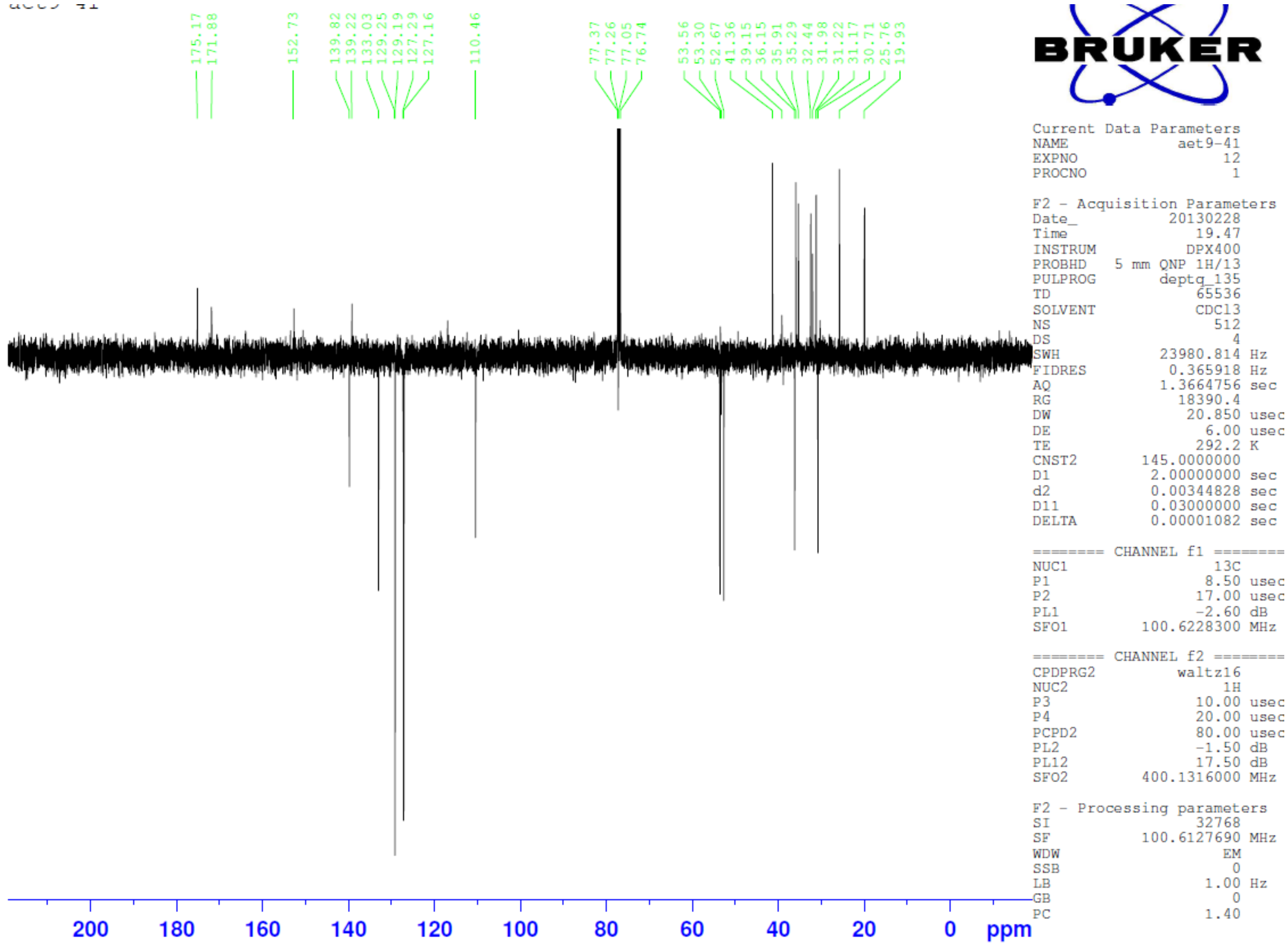
175.17
171.88
152.73
139.82
139.22
133.03
129.25
129.19
127.29
127.16
110.46
77.37
77.26
77.05
76.74
53.56
53.30
52.67
41.36
39.15
36.15
35.91
35.29
32.44
31.98
31.22
31.17
30.71
25.76
19.93
BRUKER
Current Data Parameters
NAME aet9-41
EXPNO 12
PROCNO 1
F2 - Acquisition Parameters
Date_ 20130228
Time 19.47
INSTRUM DPX400
PROBHD 5 mm QNP 1H/13
PULPROG deptq_135
TD 65536
SOLVENT CDCl3
NS 512
DS 4
SWH 23980.814 Hz
FIDRES 0.365918 Hz
AQ 1.3664756 sec
RG 18390.4
DW 20.850 usec
DE 6.00 usec
TE 292.2 K
CNST2 145.0000000
D1 2.00000000 sec
d2 0.00344828 sec
D11 0.03000000 sec
DELTA 0.00001082 sec
======== CHANNEL f1 ========
NUC1 13C
P1 8.50 usec
P2 17.00 usec
PL1 -2.60 dB
SFO1 100.6228300 MHz
======== CHANNEL f2 ========
CPDPRG2 waltz16
NUC2 1H
P3 10.00 usec
P4 20.00 usec
PCPD2 80.00 usec
PL2 -1.50 dB
PL12 17.50 dB
SFO2 400.1316000 MHz
F2 - Processing parameters
SI 32768
SF 100.6127690 MHz
WDW EM
SSB 0
LB 1.00 Hz
GB 0
PC 1.40
200 180 160 140 120 100 80 60 40 20 0 ppm

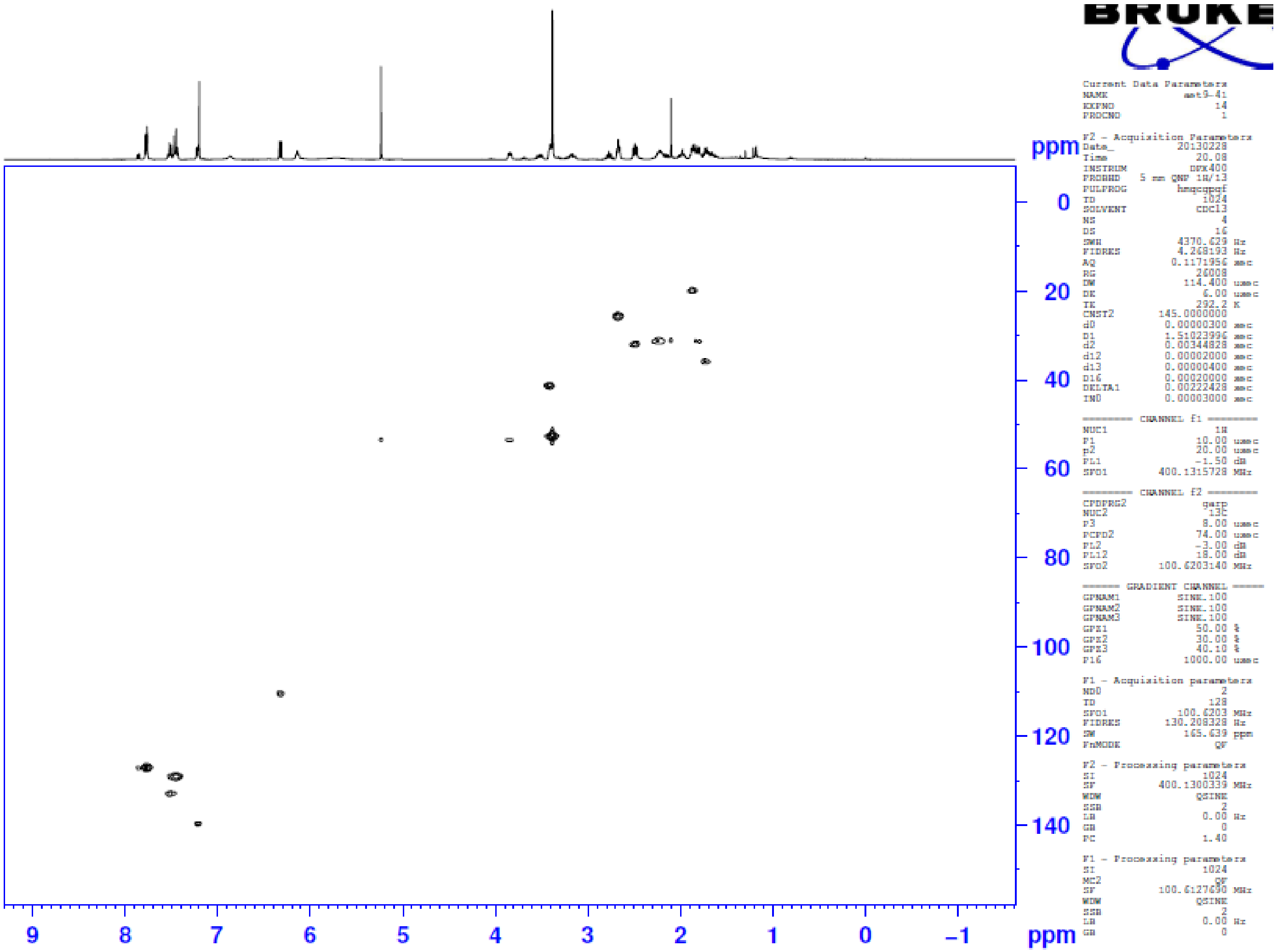
ppm
0
20
40
60
80
100
120
140
9
8
7
6
5
4
3
2
1
0
−1
ppm
Current Data Parameters
NAME mst9-41
EXPNO 14
PROCNO 1
F2 - Acquisition Parameters
Date_ 20130228
Time 20.08
INSTRUM DPX400
PROBHD 5 mm QNP 1H/13
PULPROG hmqcgpqf
TD 1024
SOLVENT CDCl3
NS 4
DS 16
SWH 4370.629 Hz
FIDRES 4.268193 Hz
AQ 0.1171956 sec
RG 26008
DW 114.400 usec
DE 6.00 usec
TE 292.2 K
CNST2 145.0000000
d0 0.00000300 sec
D1 1.51023996 sec
d2 0.00344828 sec
d12 0.00002000 sec
d13 0.00000400 sec
D16 0.00020000 sec
DELTA1 0.00222428 sec
IN0 0.00003000 sec
======== CHANNEL f1 ========
NUC1 1H
P1 10.00 usec
p2 20.00 usec
PL1 -1.50 dB
SFO1 400.1315728 MHz
======== CHANNEL f2 ========
CPDPRG2 garp
NUC2 13C
P3 8.00 usec
PCPD2 74.00 usec
PL2 -3.00 dB
PL12 18.00 dB
SFO2 100.6203140 MHz
====== GRADIENT CHANNEL =====
GPNAM1 SINE.100
GPNAM2 SINE.100
GPNAM3 SINE.100
GPZ1 50.00 %
GPZ2 30.00 %
GPZ3 40.10 %
P16 1000.00 usec
F1 - Acquisition parameters
ND0 2
TD 128
SFO1 100.6203 MHz
FIDRES 130.208328 Hz
SW 165.639 ppm
FnMODE QF
F2 - Processing parameters
SI 1024
SF 400.1300339 MHz
WDW QSINE
SSB 2
LB 0.00 Hz
GB 0
PC 1.40
F1 - Processing parameters
SI 1024
MC2 QF
SF 100.6127690 MHz
WDW QSINE
SSB 2
LB 0.00 Hz
GB 0

Methyl (*S*)-4-((1*r,3*R**)-3-(2-(5,6,7,8-tetrahydro-1,8-naphthyridin-2-yl)ethyl)cyclobutane-1-carboxamido)-2-(2,4,6-trimethylphenylsulfonamido)butanoate [Compound 24]**

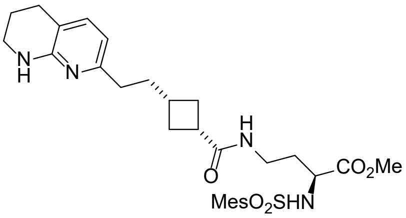

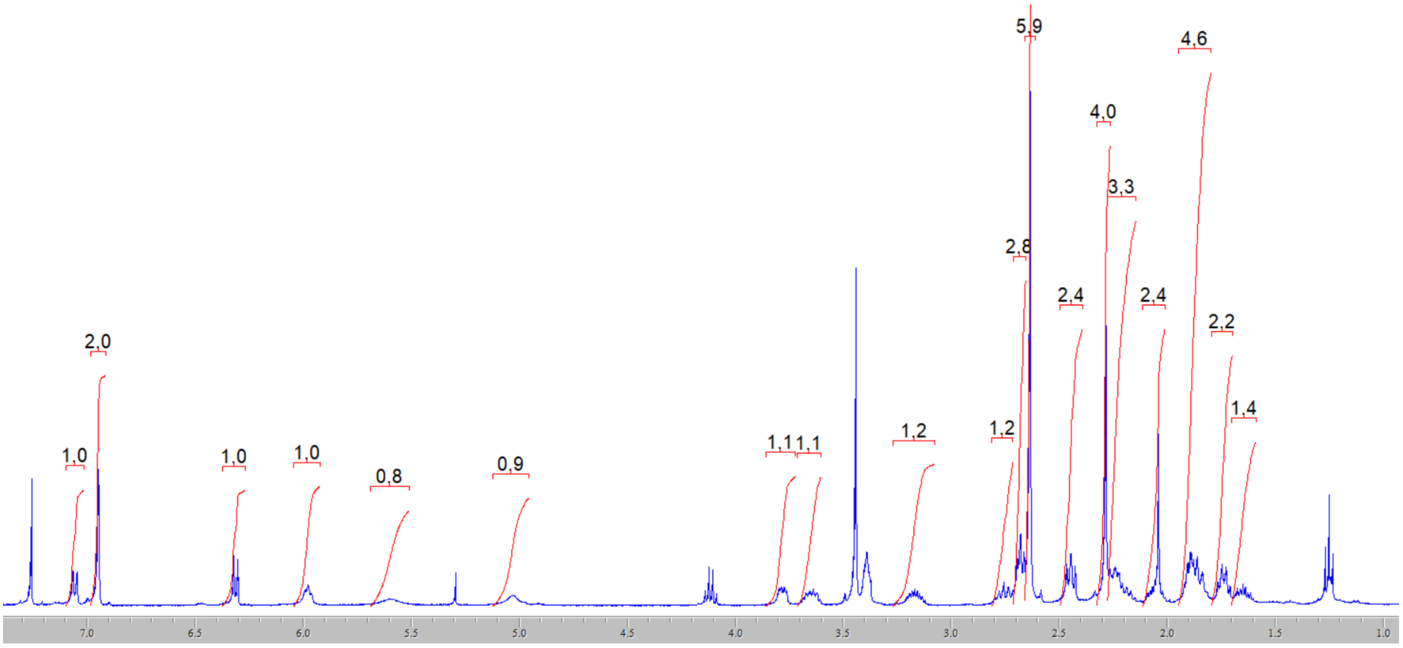

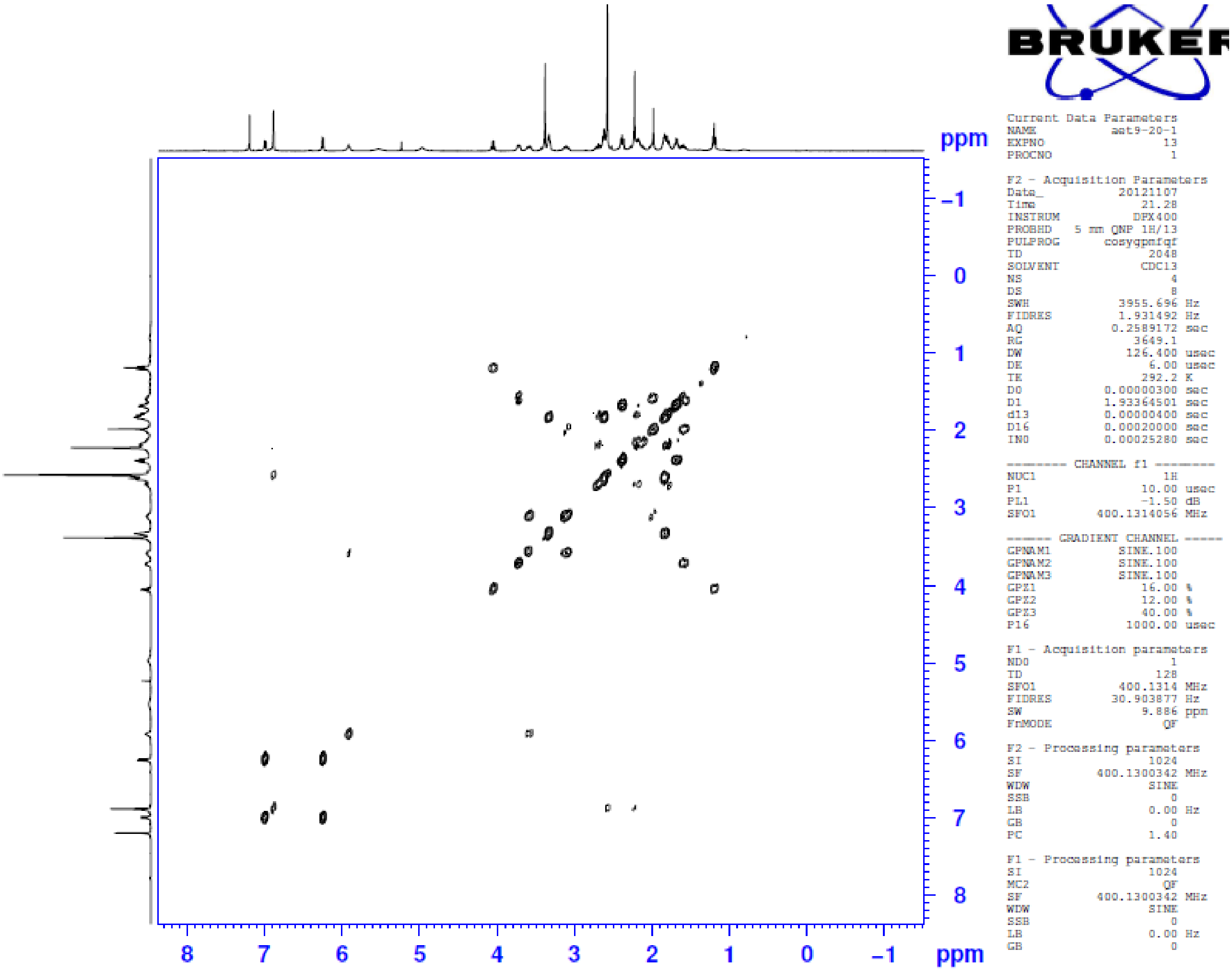

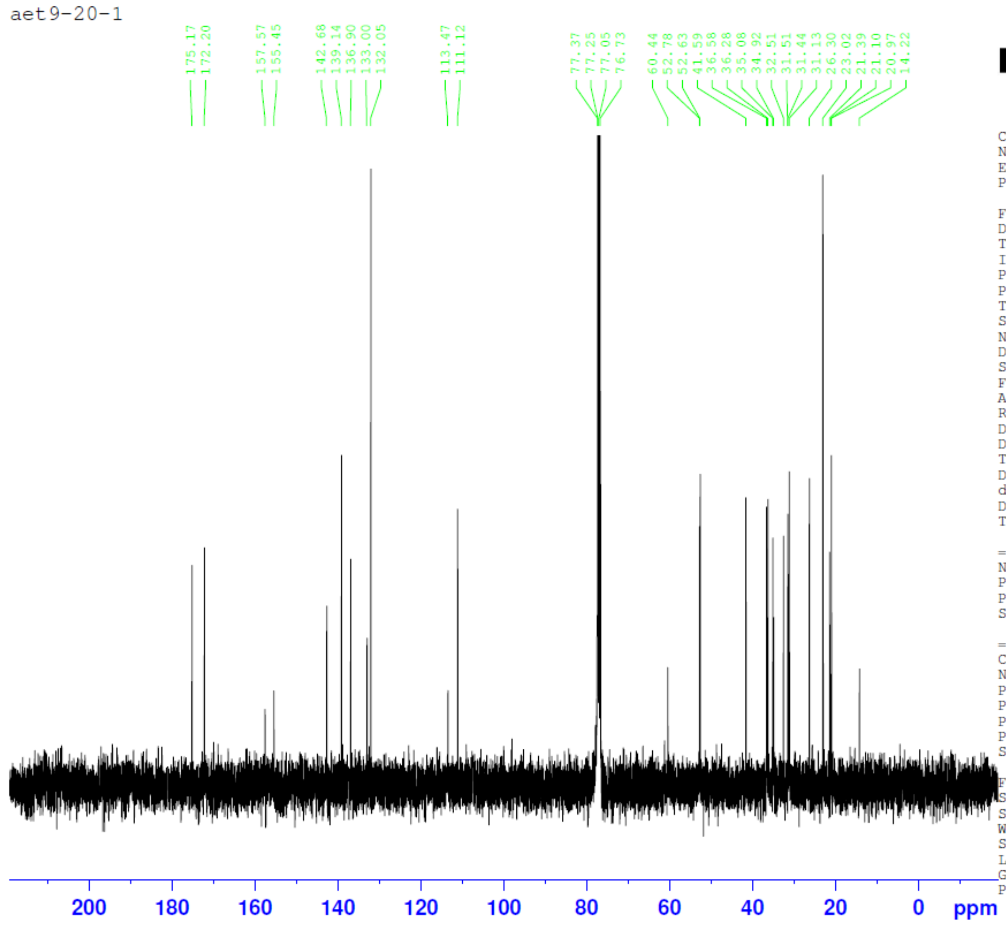

Current Data Parameters

NAME	aet9-20-1
EXPNO	11
PROCNO	1

F2 - Acquisition Parameters

Date_	20121107
Time	20.57
INSTRUM	DPX400
PROBHD	5 mm QNP 1H/13
PULPROG	zgpg30
TD	65536
SOLVENT	CDCl3
NS	1024
DS	4
SWH	23980.814 Hz
FIDRES	0.365918 Hz
AQ	1.3664756 sec
RG	512
DW	20.850 usec
DE	6.00 usec
TE	292.2 K
D1	2.00000000 sec
d11	0.03000000 sec
DELTA	1.89999998 sec
TD0	1

======== CHANNEL f1 ========

NUC1	13C
P1	8.50 usec
PL1	-2.60 dB
SFO1	100.6228298 MHz

======== CHANNEL f2 ========

CPDPRG2	waltz16
NUC2	1H
PCPD2	80.00 usec
PL2	-1.50 dB
PL12	17.50 dB
PL13	17.50 dB
SFO2	400.1316005 MHz

F2 - Processing parameters

SI	32768
SF	100.6127690 MHz
WDW	EM
SSB	0
LB	1.00 Hz
GB	0
PC	1.40

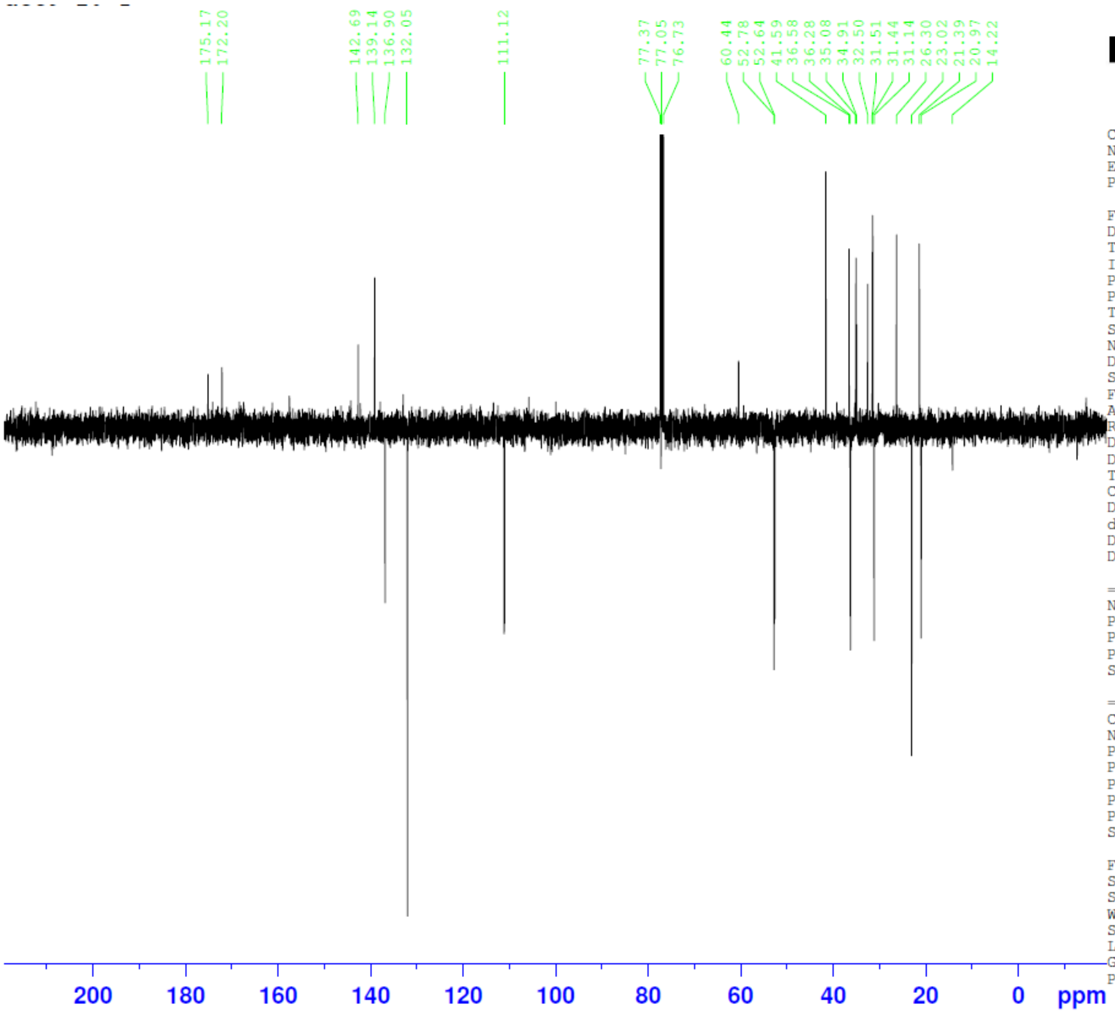

Current Data Parameters

NAME	aet9-20-1
EXPNO	12
PROCNO	1

F2 - Acquisition Parameters

Date_	20121107
Time	21.27
INSTRUM	DPX400
PROBHD	5 mm QNP 1H/13
PULPROG	deptq_135
TD	65536
SOLVENT	CDCl3
NS	512
DS	4
SWH	23980.814 Hz
FIDRES	0.365918 Hz
AQ	1.3664756 sec
RG	20642.5
DW	20.850 usec
DE	6.00 usec
TE	292.2 K
CNST2	145.0000000
D1	2.00000000 sec
d2	0.00344828 sec
D11	0.03000000 sec
DELTA	0.00001082 sec

======== CHANNEL f1 ========

NUC1	13C
P1	8.50 usec
P2	17.00 usec
PL1	-2.60 dB
SFO1	100.6228300 MHz

======== CHANNEL f2 ========

CPDPRG2	waltz16
NUC2	1H
P3	10.00 usec
P4	20.00 usec
PCPD2	80.00 usec
PL2	-1.50 dB
PL12	17.50 dB
SFO2	400.1316000 MHz

F2 - Processing parameters

SI	32768
SF	100.6127690 MHz
WDW	EM
SSB	0
LB	1.00 Hz
GB	0
PC	1.40

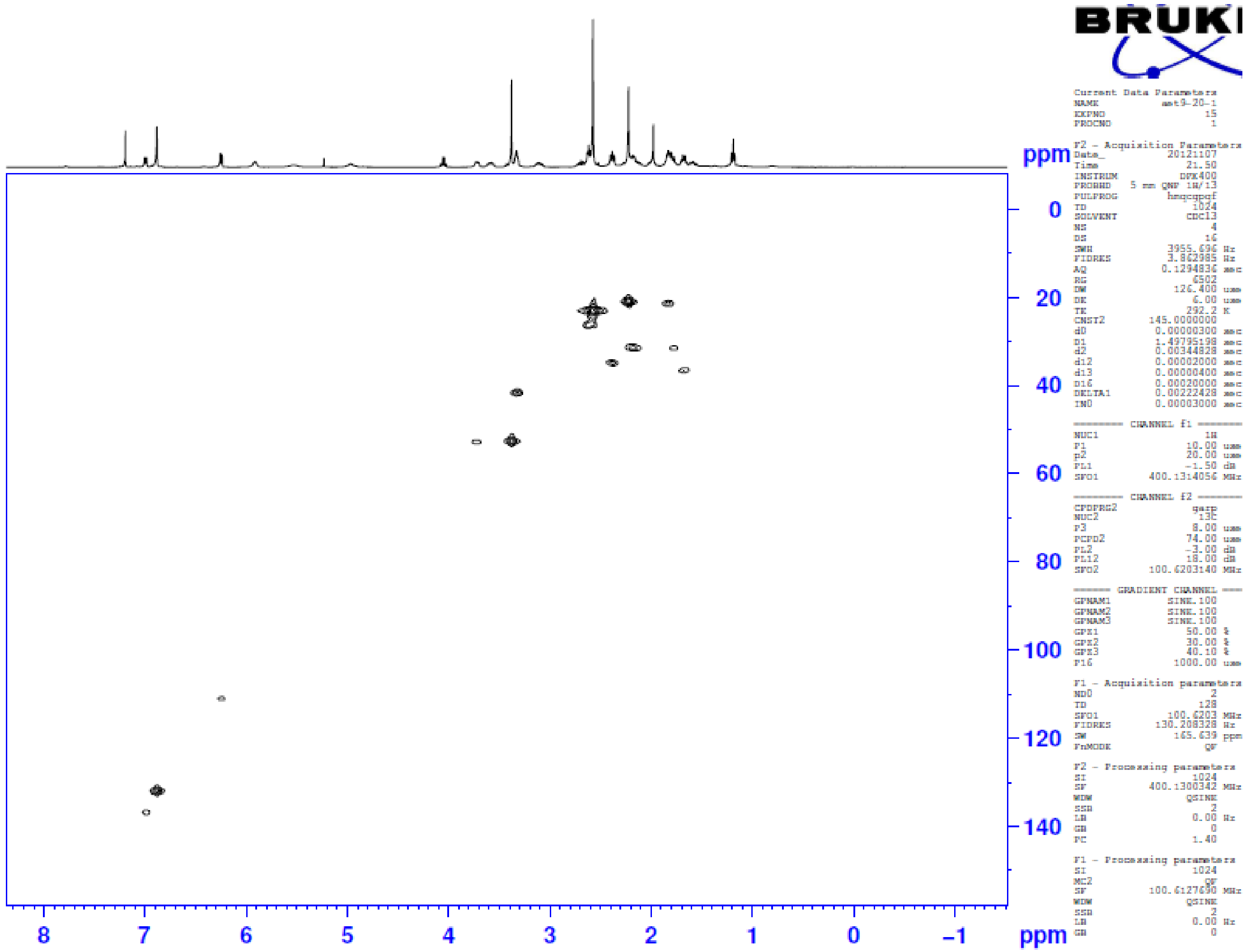
ppm
0
20
40
60
80
100
120
140
8
7
6
5
4
3
2
1
0
–1
ppm
Current Data Parameters
NAME amt9-20-1
EXPNO 15
PROCNO 1
F2 - Acquisition Parameters
Date_ 20121107
Time 21.50
INSTRUM DPX400
PROBHD 5 mm QNP 1H/13
PULPROG hmqcgpqf
TD 1024
SOLVENT CDCl3
NS 4
DS 16
SWH 3955.696 Hz
FIDRES 3.862985 Hz
AQ 0.1294836 sec
RG 6502
DW 126.400 usec
DE 6.00 usec
TE 292.2 K
CNST2 145.0000000
d0 0.00000300 sec
D1 1.49795198 sec
d2 0.00344828 sec
d12 0.00002000 sec
d13 0.00000400 sec
D16 0.00020000 sec
DELTA1 0.00222428 sec
IN0 0.00003000 sec
CHANNEL f1
NUC1 1H
P1 10.00 usec
p2 20.00 usec
PL1 -1.50 dB
SFO1 400.1314056 MHz
CHANNEL f2
CPDPRG2 garp
NUC2 13C
P3 8.00 usec
PCPD2 74.00 usec
PL2 -3.00 dB
PL12 18.00 dB
SFO2 100.6203140 MHz
GRADIENT CHANNEL
GPNAM1 SINE.100
GPNAM2 SINE.100
GPNAM3 SINE.100
GPZ1 50.00 %
GPZ2 30.00 %
GPZ3 40.10 %
P16 1000.00 usec
F1 - Acquisition parameters
ND0 2
TD 128
SFO1 100.6203 MHz
FIDRES 130.208328 Hz
SW 165.639 ppm
FnMODE QF
F2 - Processing parameters
SI 1024
SF 400.1300342 MHz
WDW QSINE
SSB 2
LB 0.00 Hz
GB 0
PC 1.40
F1 - Processing parameters
SI 1024
MC2 QF
SF 100.6127690 MHz
WDW QSINE
SSB 2
LB 0.00 Hz
GB 0

(1*r, 3*s**)-3-(2-(1,8-Naphthyridin-2-yl)ethyl)cyclobutan-1-amine [Compound 25]:**

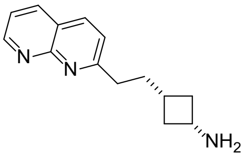

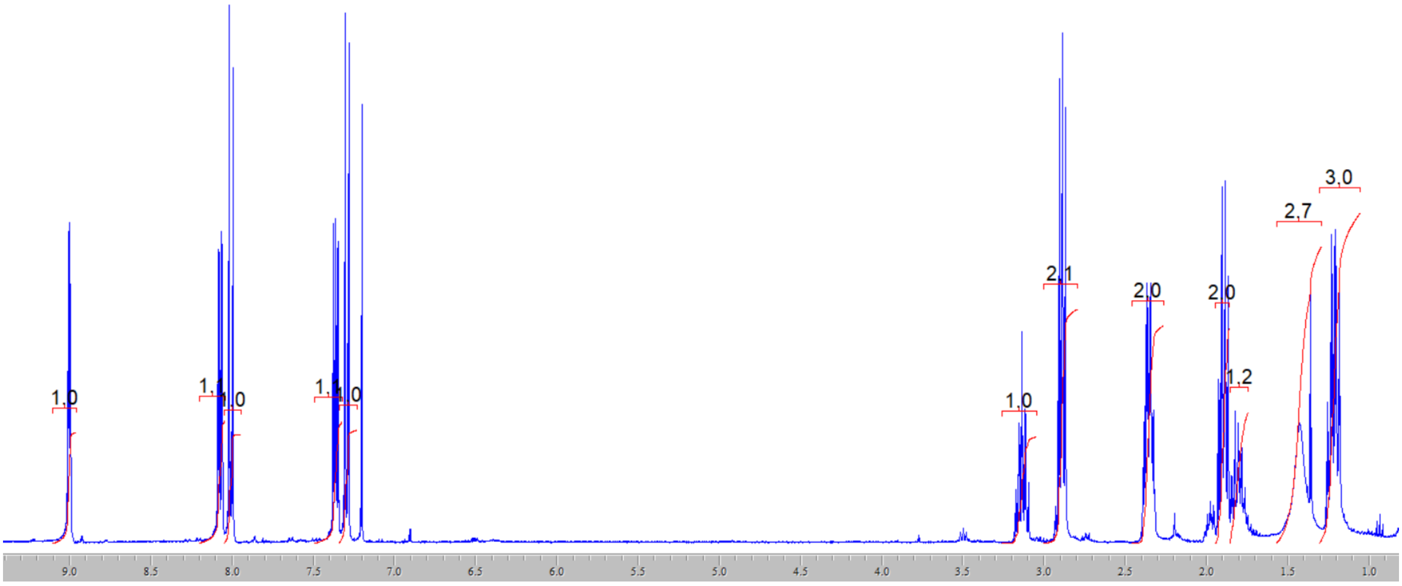

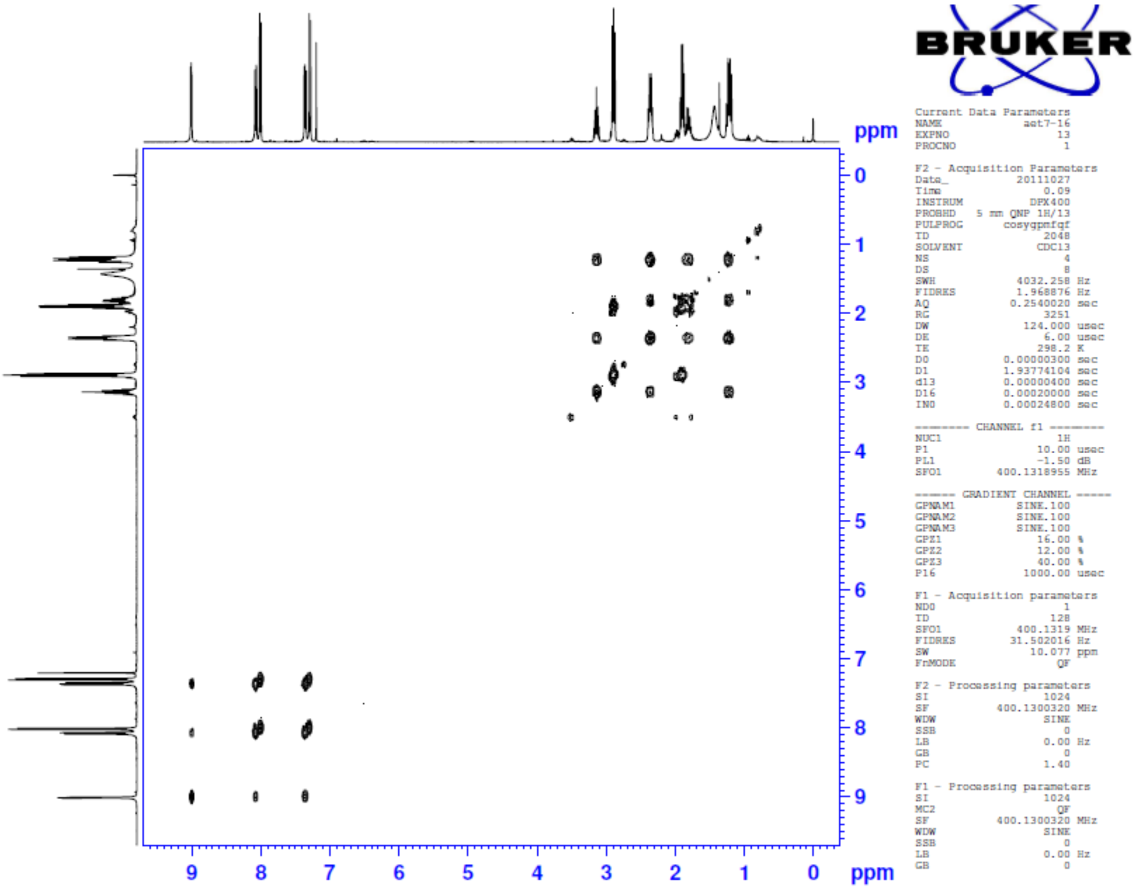

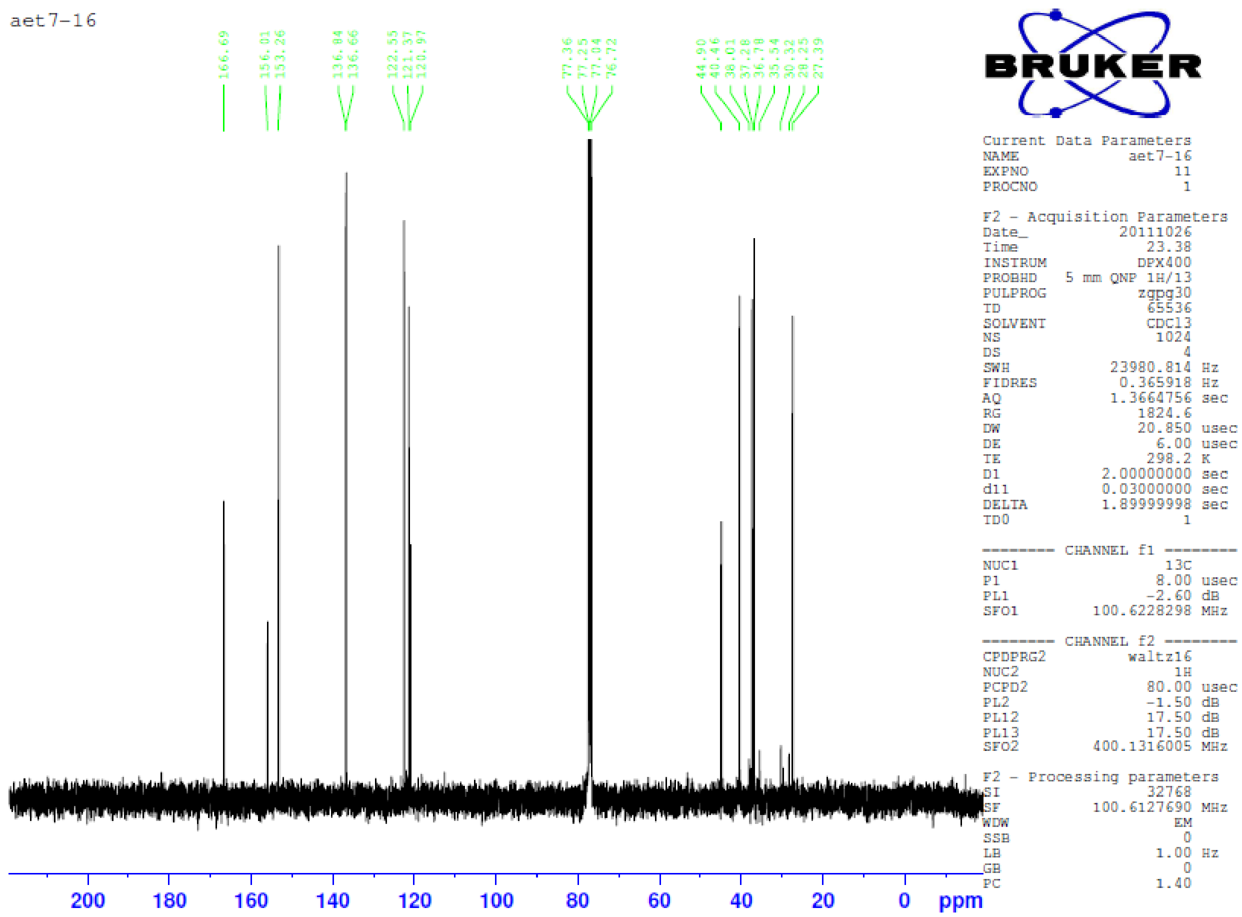
aet7-16
166.69
156.01
153.26
136.84
136.66
122.55
121.37
120.97
77.36
77.25
77.04
76.72
44.90
40.46
38.01
37.28
36.78
35.54
30.32
28.25
27.39
200
180
160
140
120
100
80
60
40
20
0
ppm
Current Data Parameters
NAME aet7-16
EXPNO 11
PROCNO 1
F2 - Acquisition Parameters
Date_ 20111026
Time 23.38
INSTRUM DPX400
PROBHD 5 mm QNP 1H/13
PULPROG zgpg30
TD 65536
SOLVENT CDCl3
NS 1024
DS 4
SWH 23980.814 Hz
FIDRES 0.365918 Hz
AQ 1.3664756 sec
RG 1824.6
DW 20.850 usec
DE 6.00 usec
TE 298.2 K
D1 2.00000000 sec
d11 0.03000000 sec
DELTA 1.89999998 sec
TD0 1
======== CHANNEL f1 ========
NUC1 13C
P1 8.00 usec
PL1 -2.60 dB
SFO1 100.6228298 MHz
======== CHANNEL f2 ========
CPDPRG2 waltz16
NUC2 1H
PCPD2 80.00 usec
PL2 -1.50 dB
PL12 17.50 dB
PL13 17.50 dB
SFO2 400.1316005 MHz
F2 - Processing parameters
SI 32768
SF 100.6127690 MHz
WDW EM
SSB 0
LB 1.00 Hz
GB 0
PC 1.40

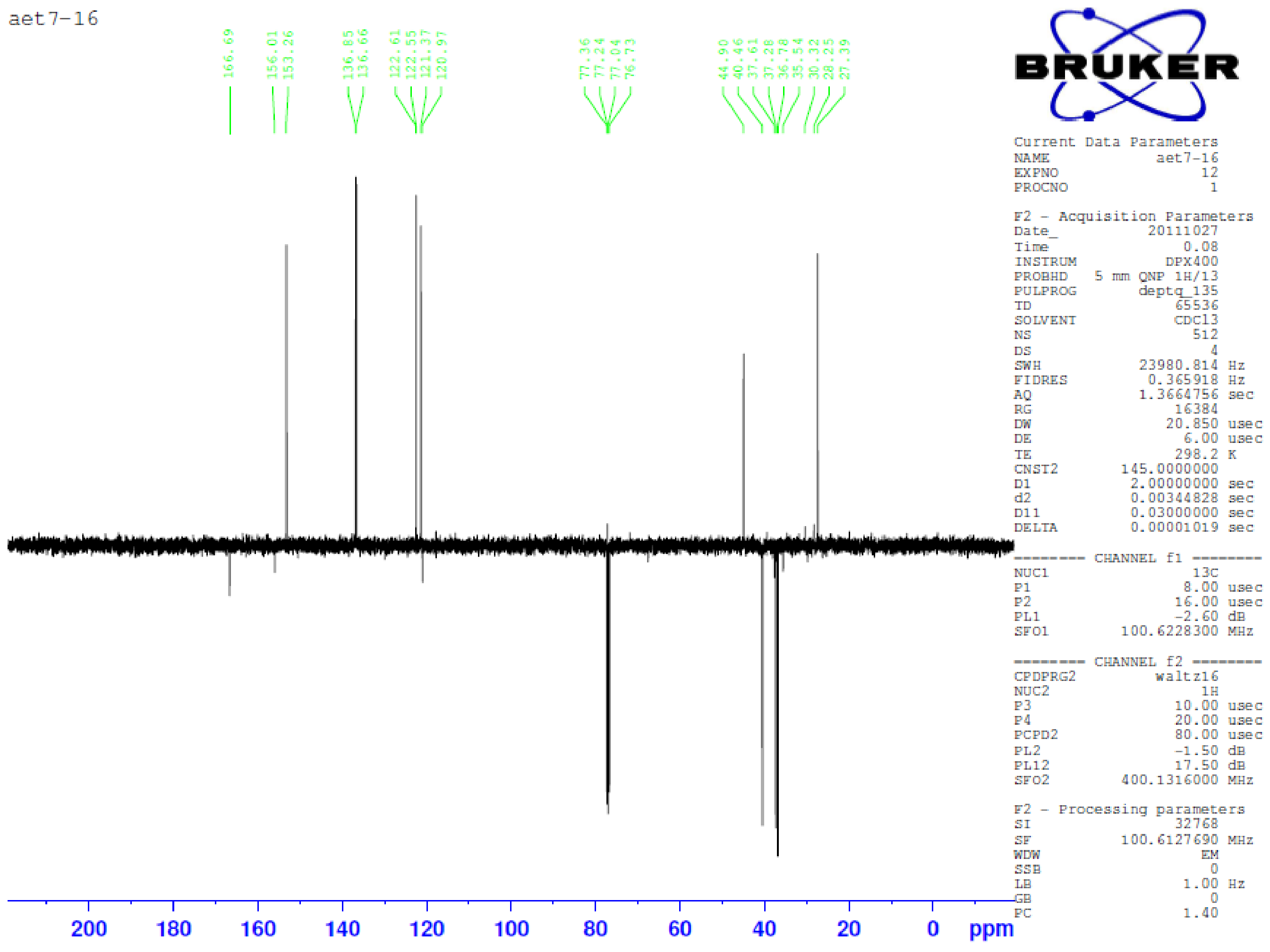
aet7-16
166.69
156.01
153.26
136.85
136.66
122.61
122.55
121.37
120.97
77.36
77.24
77.04
76.73
44.90
40.46
37.61
37.28
36.78
35.54
30.32
28.25
27.39
200
180
160
140
120
100
80
60
40
20
0
ppm
Current Data Parameters
NAME aet7-16
EXPNO 12
PROCNO 1
F2 - Acquisition Parameters
Date_ 20111027
Time 0.08
INSTRUM DPX400
PROBHD 5 mm QNP 1H/13
PULPROG deptq_135
TD 65536
SOLVENT CDCl3
NS 512
DS 4
SWH 23980.814 Hz
FIDRES 0.365918 Hz
AQ 1.3664756 sec
RG 16384
DW 20.850 usec
DE 6.00 usec
TE 298.2 K
CNST2 145.0000000
D1 2.00000000 sec
d2 0.00344828 sec
D11 0.03000000 sec
DELTA 0.00001019 sec
======== CHANNEL f1 ========
NUC1 13C
P1 8.00 usec
P2 16.00 usec
PL1 -2.60 dB
SFO1 100.6228300 MHz
======== CHANNEL f2 ========
CPDPRG2 waltz16
NUC2 1H
P3 10.00 usec
P4 20.00 usec
PCPD2 80.00 usec
PL2 -1.50 dB
PL12 17.50 dB
SFO2 400.1316000 MHz
F2 - Processing parameters
SI 32768
SF 100.6127690 MHz
WDW EM
SSB 0
LB 1.00 Hz
GB 0
PC 1.40

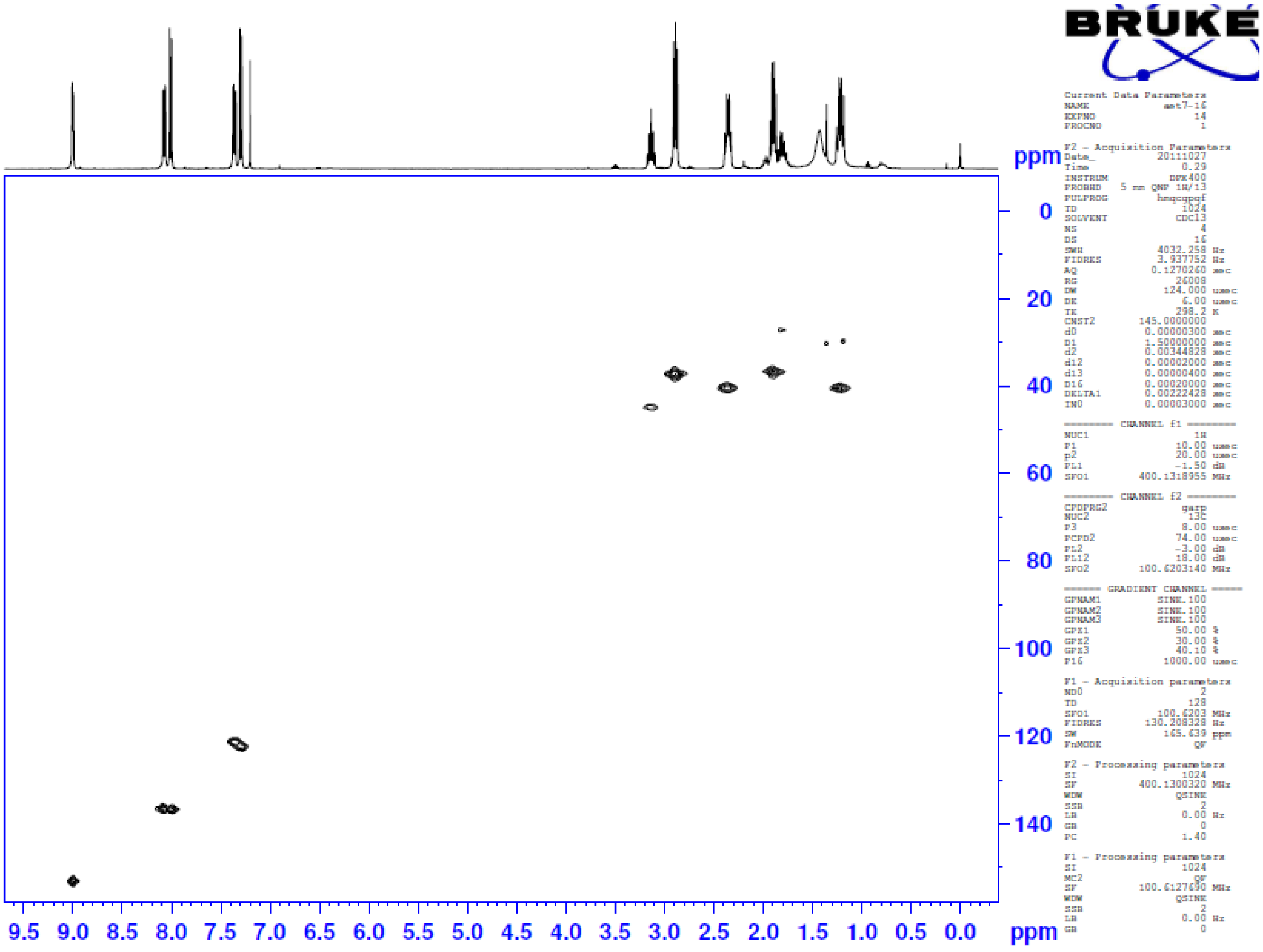
ppm
0
20
40
60
80
100
120
140
9.5 9.0 8.5 8.0 7.5 7.0 6.5 6.0 5.5 5.0 4.5 4.0 3.5 3.0 2.5 2.0 1.5 1.0 0.5 0.0
ppm
Current Data Parameters
NAME amt7-16
EXPNO 14
PROCNO 1
F2 - Acquisition Parameters
Date_ 20111027
Time 0.29
INSTRUM DPX400
PROBHD 5 mm QNP 1H/13
PULPROG hsqcgpqf
TD 1024
SOLVENT CDCl3
NS 4
DS 16
SWH 4032.258 Hz
FIDRES 3.937752 Hz
AQ 0.1270260 sec
RG 26008
DW 124.000 usec
DE 6.00 usec
TE 298.2 K
CNST2 145.0000000
d0 0.00000300 sec
D1 1.50000000 sec
d2 0.00344828 sec
d12 0.00002000 sec
d13 0.00000400 sec
D16 0.00020000 sec
DELTA1 0.00222428 sec
IN0 0.00003000 sec
======== CHANNEL f1 ========
NUC1 1H
P1 10.00 usec
p2 20.00 usec
PL1 -1.50 dB
SFO1 400.1318955 MHz
======== CHANNEL f2 ========
CPDPRG2 garp
NUC2 13C
P3 8.00 usec
PCPD2 74.00 usec
PL2 -3.00 dB
PL12 18.00 dB
SFO2 100.6203140 MHz
====== GRADIENT CHANNEL =====
GPNAM1 SINE.100
GPNAM2 SINE.100
GPNAM3 SINE.100
GPZ1 50.00 %
GPZ2 30.00 %
GPZ3 40.10 %
P16 1000.00 usec
F1 - Acquisition parameters
ND0 2
TD 128
SFO1 100.6203 MHz
FIDRES 130.208328 Hz
SW 165.639 ppm
FnMODE QF
F2 - Processing parameters
SI 1024
SF 400.1300320 MHz
WDW QSINE
SSB 2
LB 0.00 Hz
GB 0
PC 1.40
F1 - Processing parameters
SI 1024
MC2 QF
SF 100.6127690 MHz
WDW QSINE
SSB 2
LB 0.00 Hz
GB 0

(*S*)-4-Methoxy-4-oxo-3-(phenylsulfonamido)butanoic acid [Compound 26]

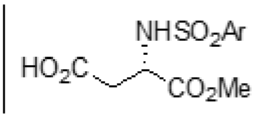

Ar = Ph

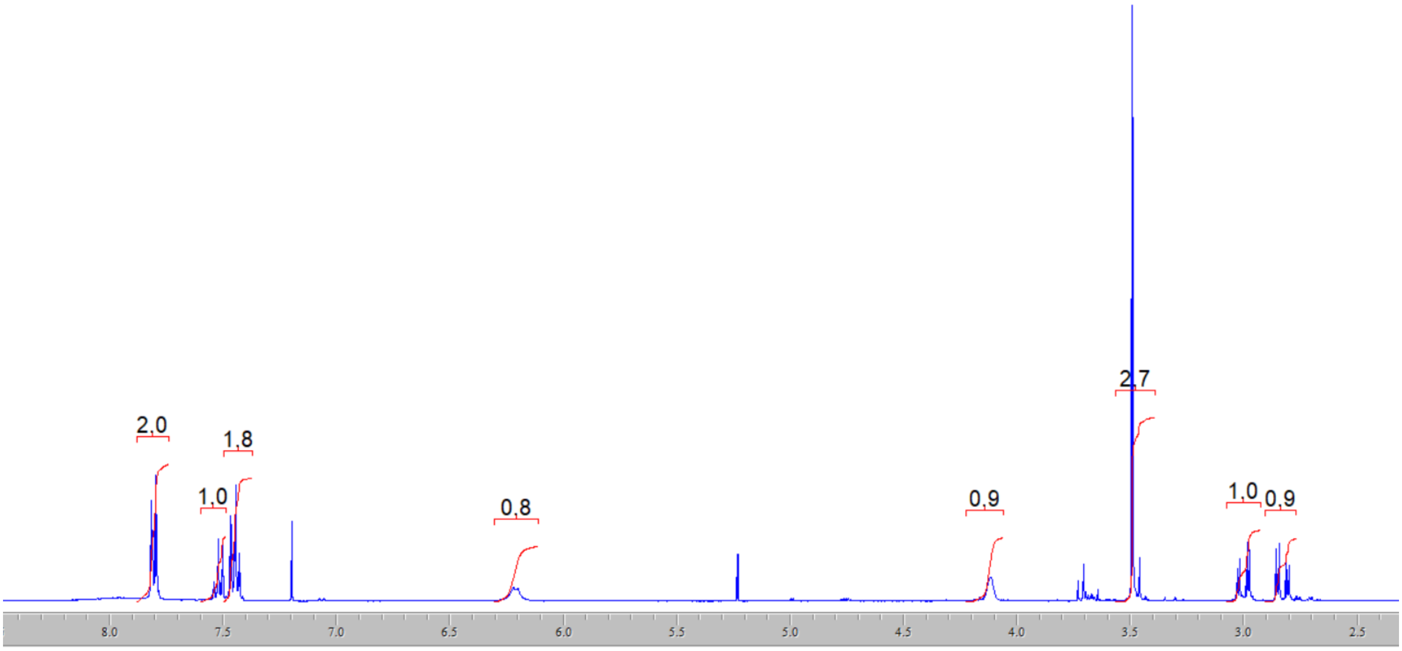

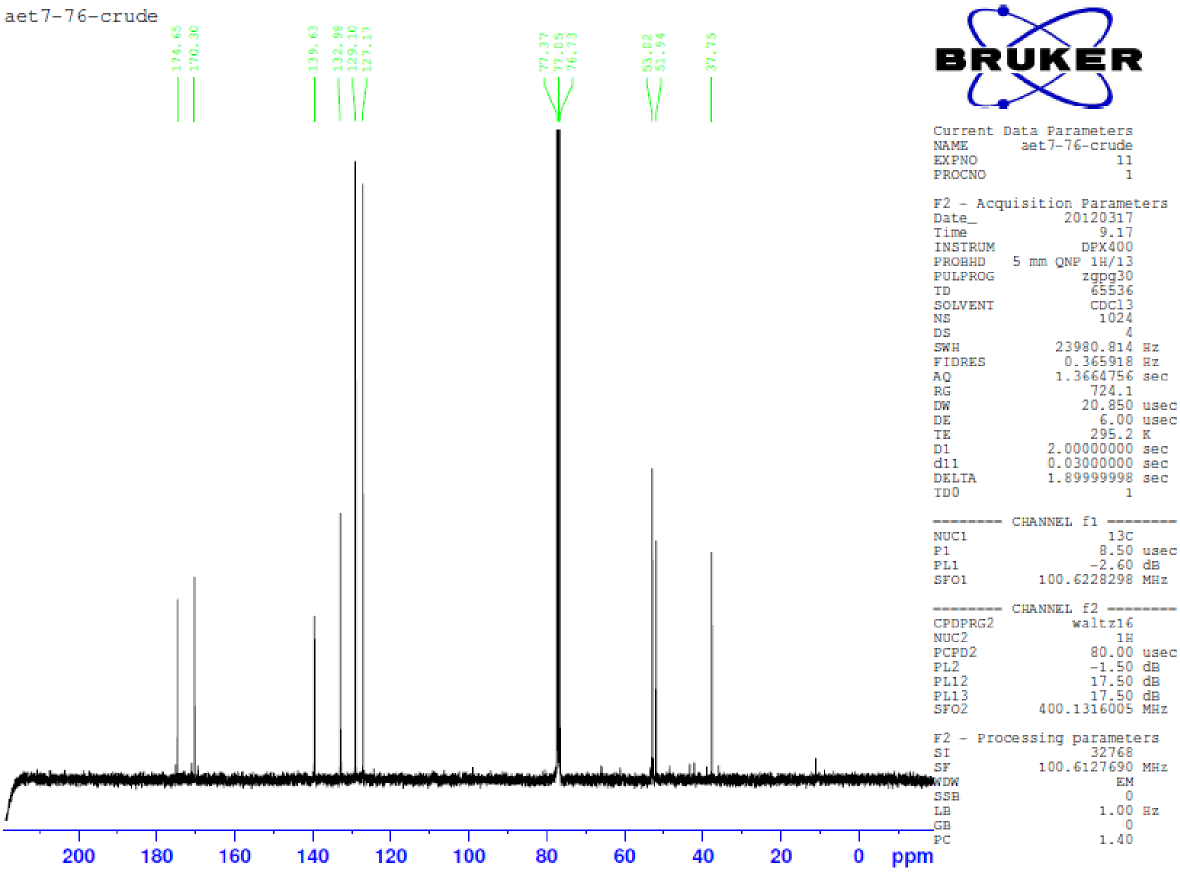
aet7-76-crude
174.65
170.30
139.63
132.98
129.10
127.17
77.37
77.05
76.73
53.02
51.94
37.75
BRUKER
Current Data Parameters
NAME aet7-76-crude
EXPNO 11
PROCNO 1
F2 - Acquisition Parameters
Date_ 20120317
Time 9.17
INSTRUM DPX400
PROBHD 5 mm QNP 1H/13
PULPROG zgpg30
TD 65536
SOLVENT CDCl3
NS 1024
DS 4
SWH 23980.814 Hz
FIDRES 0.365918 Hz
AQ 1.3664756 sec
RG 724.1
DW 20.850 usec
DE 6.00 usec
TE 295.2 K
D1 2.00000000 sec
d11 0.03000000 sec
DELTA 1.89999998 sec
TD0 1
======== CHANNEL f1 ========
NUC1 13C
P1 8.50 usec
PL1 -2.60 dB
SFO1 100.6228298 MHz
======== CHANNEL f2 ========
CPDPRG2 waltz16
NUC2 1H
PCPD2 80.00 usec
PL2 -1.50 dB
PL12 17.50 dB
PL13 17.50 dB
SFO2 400.1316005 MHz
F2 - Processing parameters
SI 32768
SF 100.6127690 MHz
WDW EM
SSB 0
LB 1.00 Hz
GB 0
PC 1.40
200 180 160 140 120 100 80 60 40 20 0 ppm

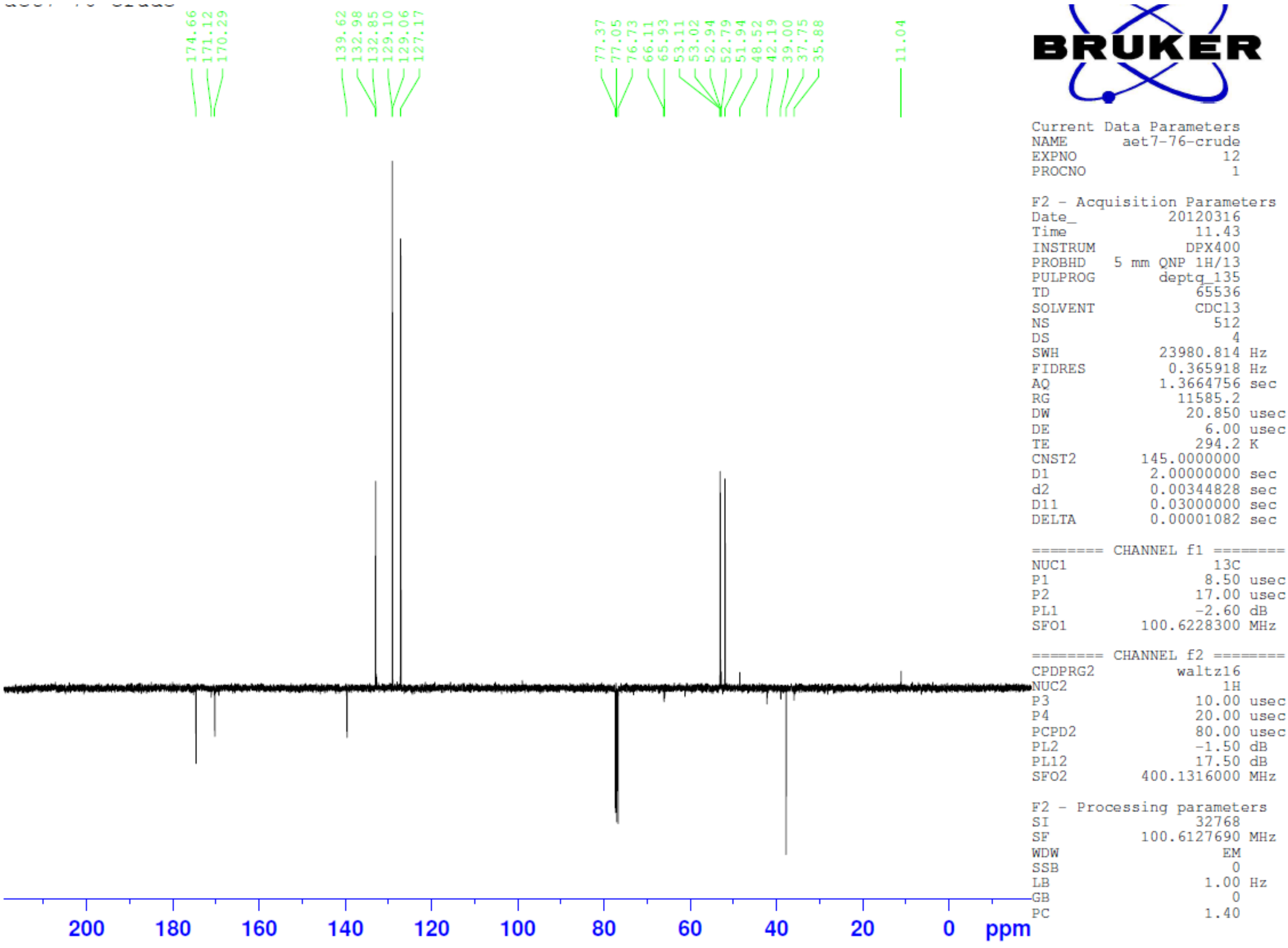
174.66
171.12
170.29
139.62
132.98
132.85
129.10
129.06
127.17
77.37
77.05
76.73
66.11
65.93
53.11
53.02
52.94
52.79
51.94
48.52
42.19
39.00
37.75
35.88
11.04
BRUKER
Current Data Parameters
NAME aet7-76-crude
EXPNO 12
PROCNO 1
F2 - Acquisition Parameters
Date_ 20120316
Time 11.43
INSTRUM DPX400
PROBHD 5 mm QNP 1H/13
PULPROG deptq_135
TD 65536
SOLVENT CDCl3
NS 512
DS 4
SWH 23980.814 Hz
FIDRES 0.365918 Hz
AQ 1.3664756 sec
RG 11585.2
DW 20.850 usec
DE 6.00 usec
TE 294.2 K
CNST2 145.0000000
D1 2.00000000 sec
d2 0.00344828 sec
D11 0.03000000 sec
DELTA 0.00001082 sec
======== CHANNEL f1 ========
NUC1 13C
P1 8.50 usec
P2 17.00 usec
PL1 -2.60 dB
SFO1 100.6228300 MHz
======== CHANNEL f2 ========
CPDPRG2 waltz16
NUC2 1H
P3 10.00 usec
P4 20.00 usec
PCPD2 80.00 usec
PL2 -1.50 dB
PL12 17.50 dB
SFO2 400.1316000 MHz
F2 - Processing parameters
SI 32768
SF 100.6127690 MHz
WDW EM
SSB 0
LB 1.00 Hz
GB 0
PC 1.40
200 180 160 140 120 100 80 60 40 20 0 ppm

(*S*)-4-Methoxy-4-oxo-3-(2,4,6-trimethylphenylsulfonamido)butanoic acid [Compound 27]

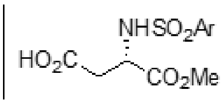

Ar = Mes

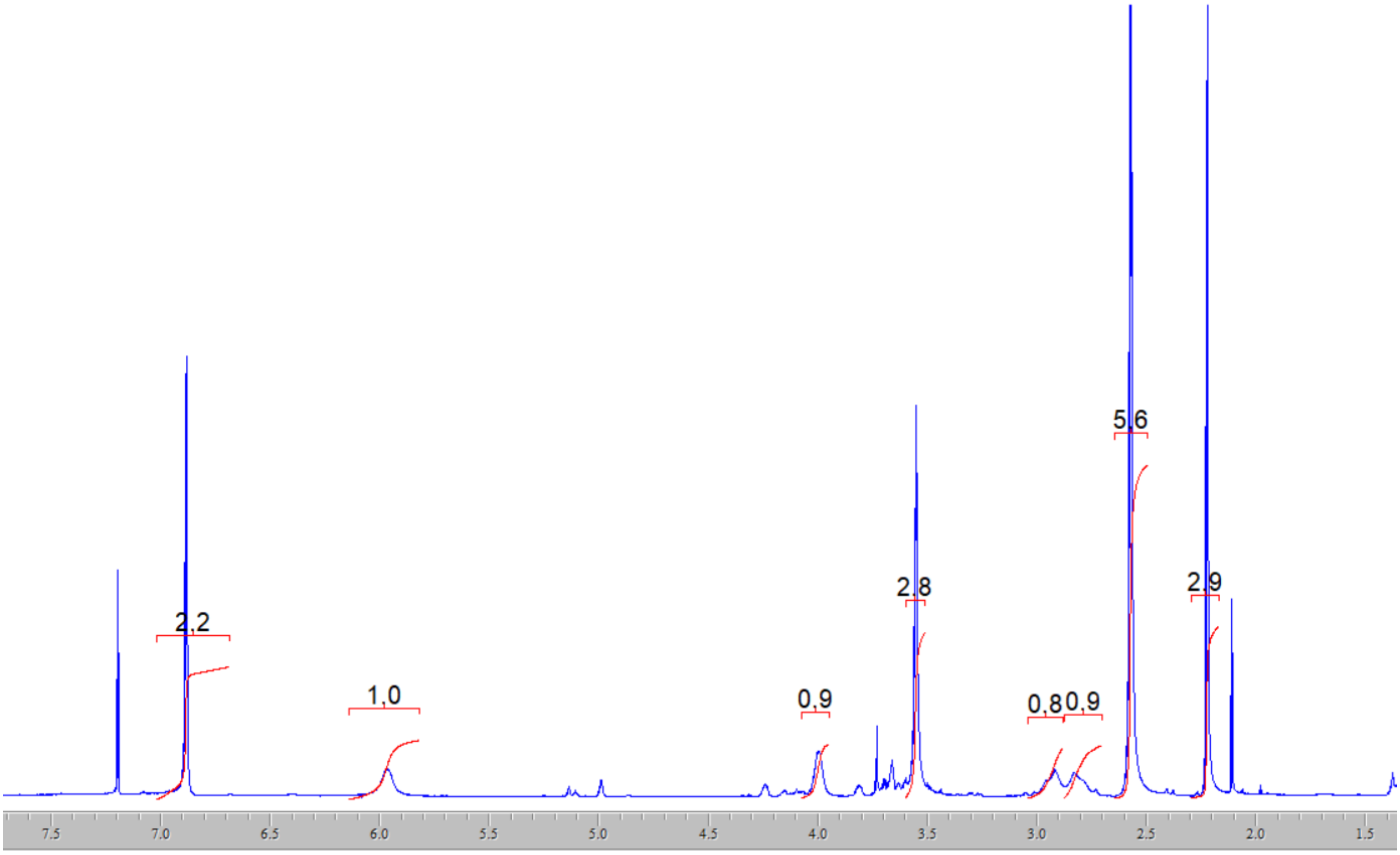

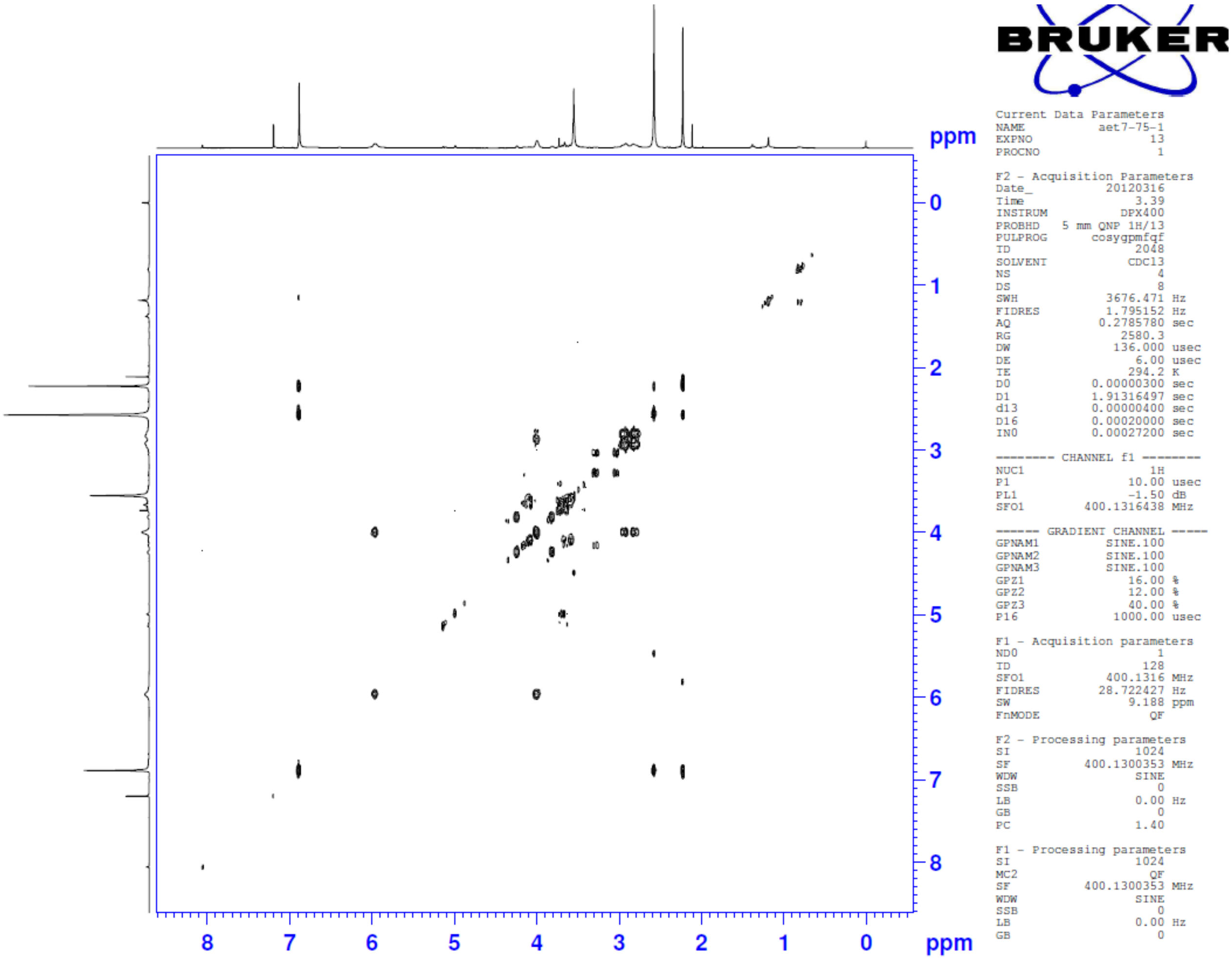

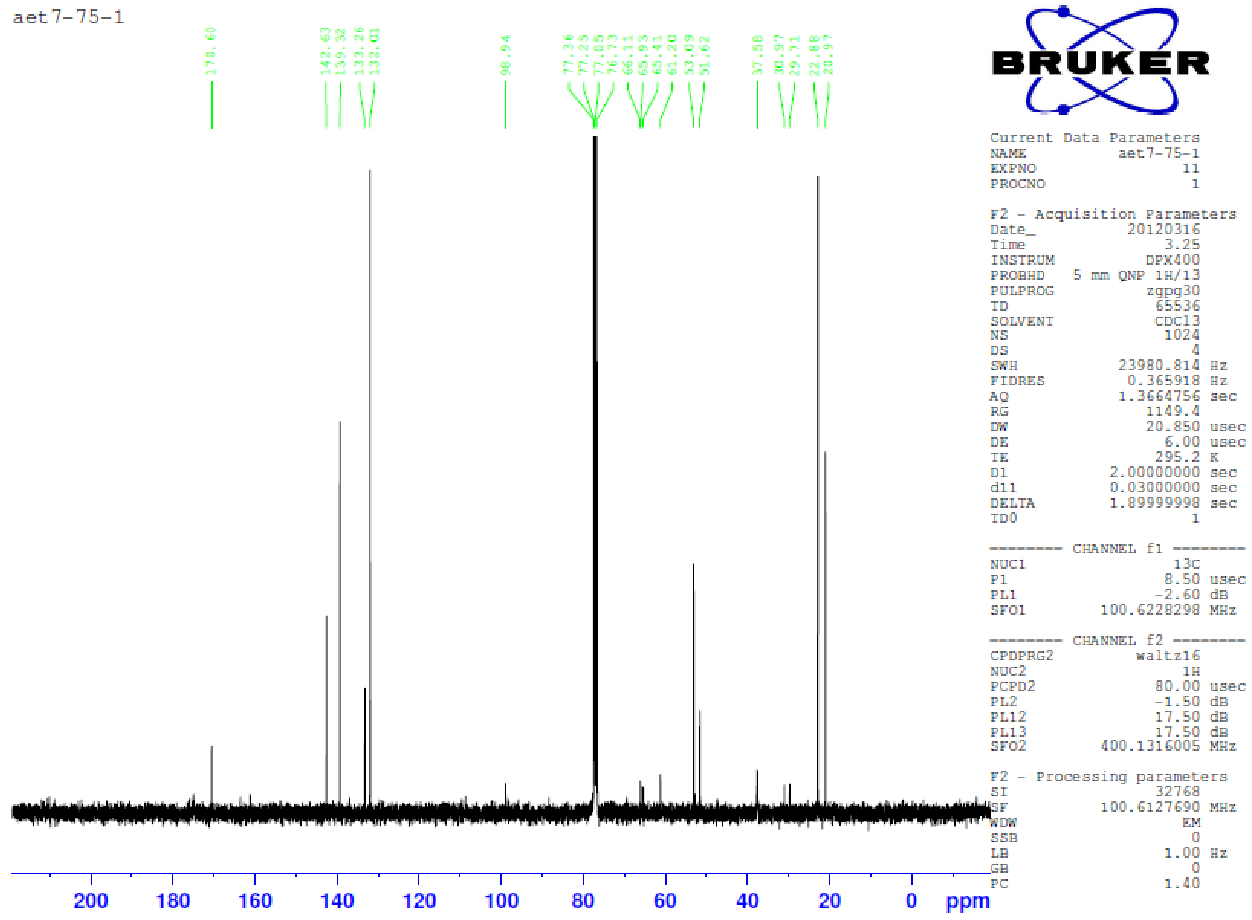
aet7-75-1
170.60
142.63
139.32
133.26
132.01
98.94
77.36
77.25
77.05
76.73
66.11
65.93
65.41
61.20
53.09
51.62
37.58
30.97
29.71
22.88
20.97
BRUKER
Current Data Parameters
NAME aet7-75-1
EXPNO 11
PROCNO 1
F2 - Acquisition Parameters
Date_ 20120316
Time 3.25
INSTRUM DPX400
PROBHD 5 mm QNP 1H/13
PULPROG zgpg30
TD 65536
SOLVENT CDCl3
NS 1024
DS 4
SWH 23980.814 Hz
FIDRES 0.365918 Hz
AQ 1.3664756 sec
RG 1149.4
DW 20.850 usec
DE 6.00 usec
TE 295.2 K
D1 2.00000000 sec
d11 0.03000000 sec
DELTA 1.89999998 sec
TD0 1
======== CHANNEL f1 ========
NUC1 13C
P1 8.50 usec
PL1 -2.60 dB
SFO1 100.6228298 MHz
======== CHANNEL f2 ========
CPDPRG2 waltz16
NUC2 1H
PCPD2 80.00 usec
PL2 -1.50 dB
PL12 17.50 dB
PL13 17.50 dB
SFO2 400.1316005 MHz
F2 - Processing parameters
SI 32768
SF 100.6127690 MHz
WDW EM
SSB 0
LB 1.00 Hz
GB 0
PC 1.40
200
180
160
140
120
100
80
60
40
20
0
ppm

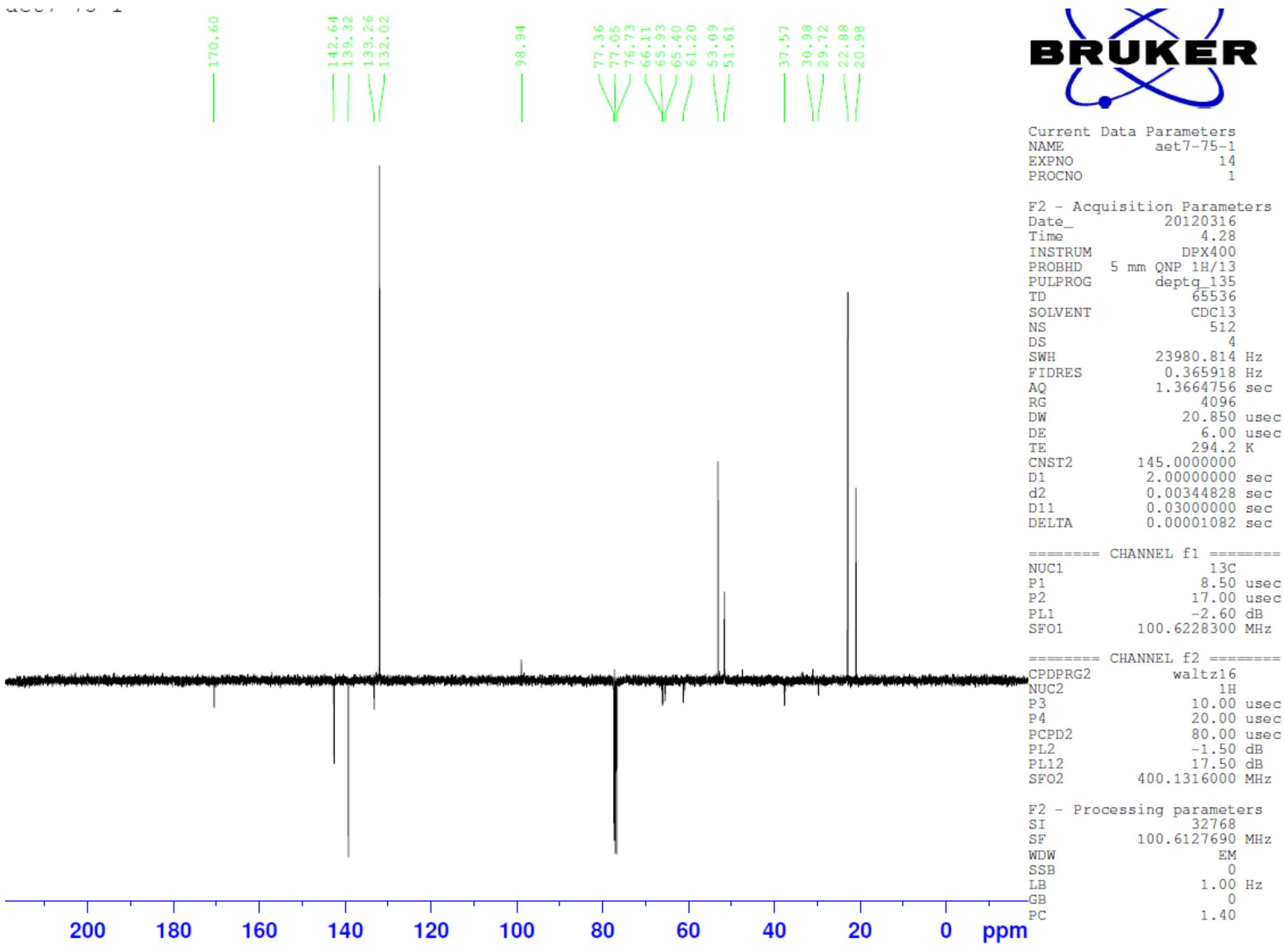
170.60
142.64
139.32
133.26
132.02
98.94
77.36
77.05
76.73
66.11
65.93
65.40
61.20
53.09
51.61
37.57
30.98
29.72
22.88
20.98
BRUKER
Current Data Parameters
NAME aet7-75-1
EXPNO 14
PROCNO 1
F2 - Acquisition Parameters
Date_ 20120316
Time 4.28
INSTRUM DPX400
PROBHD 5 mm QNP 1H/13
PULPROG deptq_135
TD 65536
SOLVENT CDCl3
NS 512
DS 4
SWH 23980.814 Hz
FIDRES 0.365918 Hz
AQ 1.3664756 sec
RG 4096
DW 20.850 usec
DE 6.00 usec
TE 294.2 K
CNST2 145.0000000
D1 2.00000000 sec
d2 0.00344828 sec
D11 0.03000000 sec
DELTA 0.00001082 sec
======== CHANNEL f1 ========
NUC1 13C
P1 8.50 usec
P2 17.00 usec
PL1 -2.60 dB
SFO1 100.6228300 MHz
======== CHANNEL f2 ========
CPDPRG2 waltz16
NUC2 1H
P3 10.00 usec
P4 20.00 usec
PCPD2 80.00 usec
PL2 -1.50 dB
PL12 17.50 dB
SFO2 400.1316000 MHz
F2 - Processing parameters
SI 32768
SF 100.6127690 MHz
WDW EM
SSB 0
LB 1.00 Hz
GB 0
PC 1.40
200
180
160
140
120
100
80
60
40
20
0
ppm

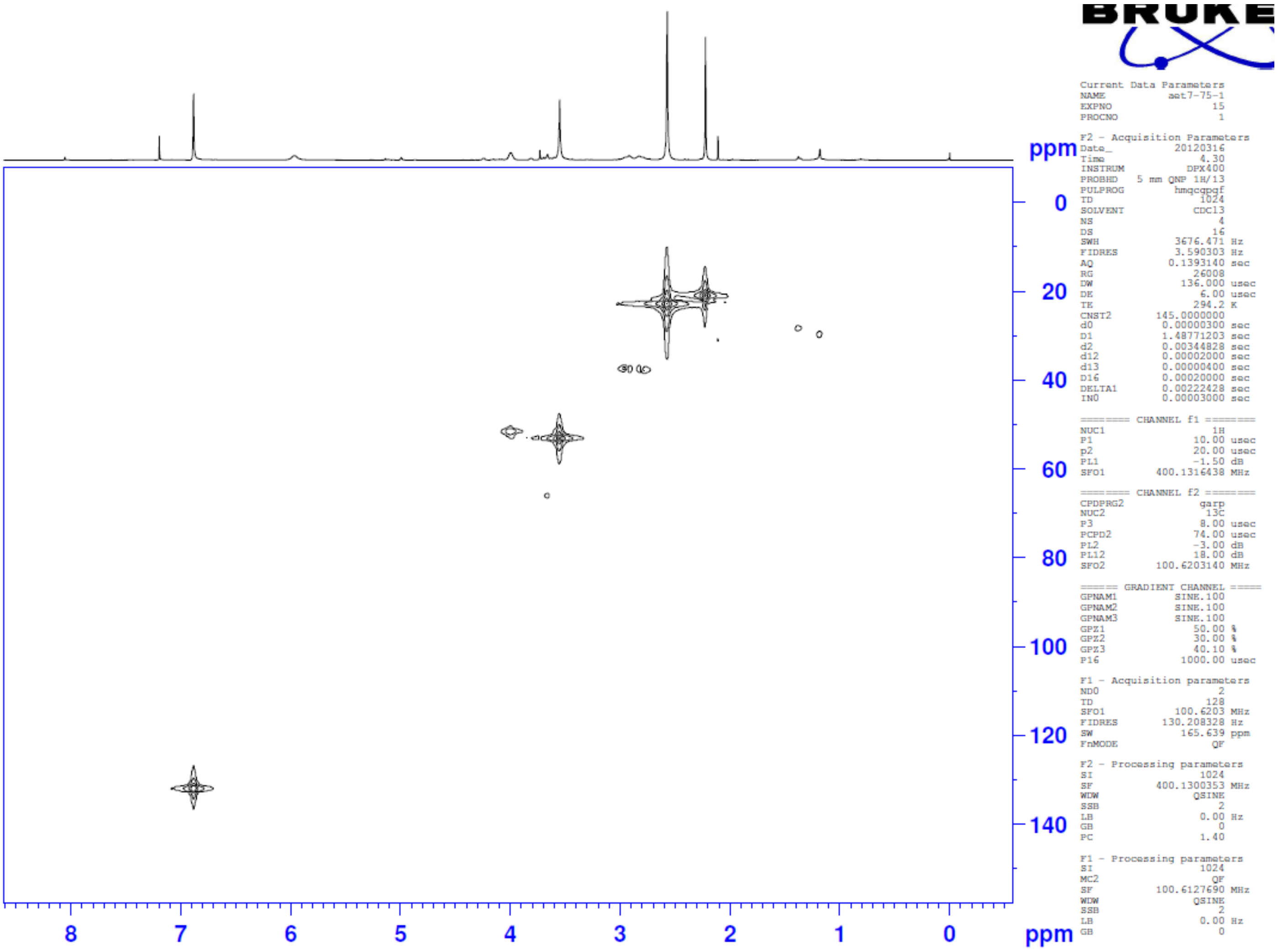

ppm
0
20
40
60
80
100
120
140
8
7
6
5
4
3
2
1
0
ppm
Current Data Parameters
NAME aet7-75-1
EXPNO 15
PROCNO 1
F2 - Acquisition Parameters
Date_ 20120316
Time 4.30
INSTRUM DPX400
PROBHD 5 mm QNP 1H/13
PULPROG hmqcgpqf
TD 1024
SOLVENT CDCl3
NS 4
DS 16
SWH 3676.471 Hz
FIDRES 3.590303 Hz
AQ 0.1393140 sec
RG 26008
DW 136.000 usec
DE 6.00 usec
TE 294.2 K
CNST2 145.0000000
d0 0.00000300 sec
D1 1.48771203 sec
d2 0.00344828 sec
d12 0.00002000 sec
d13 0.00000400 sec
D16 0.00020000 sec
DELTA1 0.00222428 sec
IN0 0.00003000 sec
======= CHANNEL f1 =======
NUC1 1H
P1 10.00 usec
p2 20.00 usec
PL1 -1.50 dB
SFO1 400.1316438 MHz
======= CHANNEL f2 =======
CPDPRG2 garp
NUC2 13C
P3 8.00 usec
PCPD2 74.00 usec
PL2 -3.00 dB
PL12 18.00 dB
SFO2 100.6203140 MHz
===== GRADIENT CHANNEL =====
GPNAM1 SINE.100
GPNAM2 SINE.100
GPNAM3 SINE.100
GPZ1 50.00 %
GPZ2 30.00 %
GPZ3 40.10 %
P16 1000.00 usec
F1 - Acquisition parameters
ND0 2
TD 128
SFO1 100.6203 MHz
FIDRES 130.208328 Hz
SW 165.639 ppm
FnMODE QF
F2 - Processing parameters
SI 1024
SF 400.1300353 MHz
WDW QSINE
SSB 2
LB 0.00 Hz
GB 0
PC 1.40
F1 - Processing parameters
SI 1024
MC2 QF
SF 100.6127690 MHz
WDW QSINE
SSB 2
LB 0.00 Hz
GB 0

Methyl *N*[4]-((1*r,3*R**)-3-(2-(1,8-naphthyridin-2-yl)ethyl)cyclobutyl)-*N*[2]-(phenylsulfonyl)-*L*-asparaginate [Compound 28]**

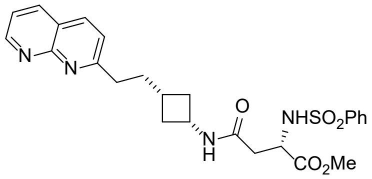

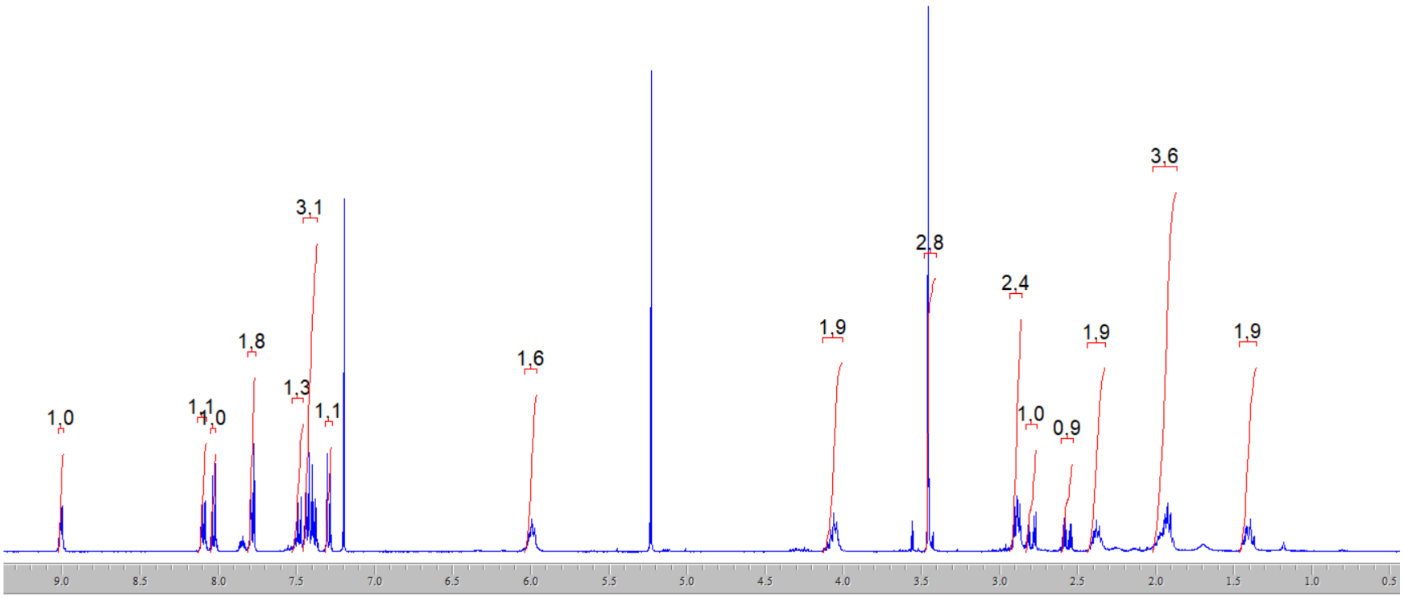

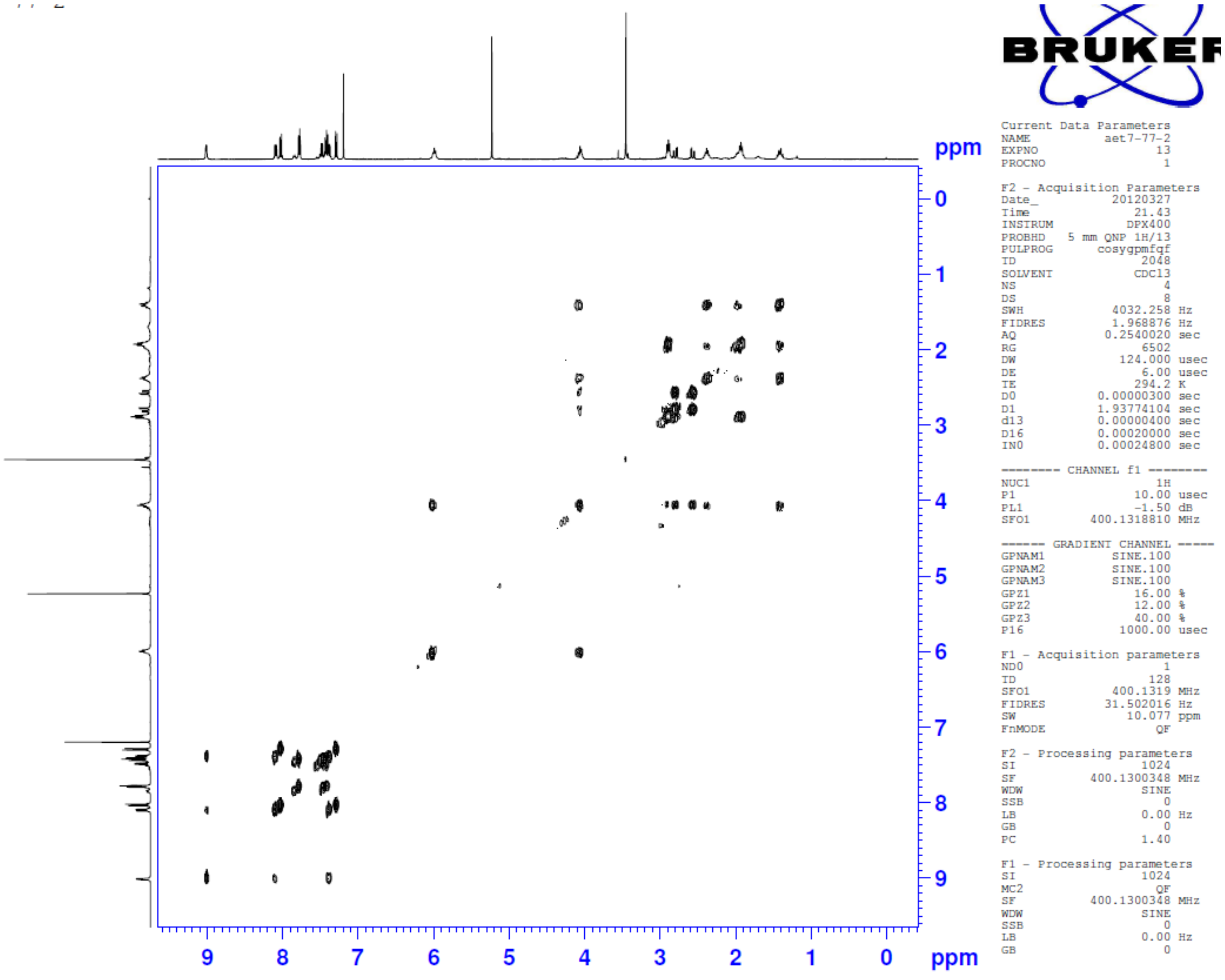

aet7-77-2

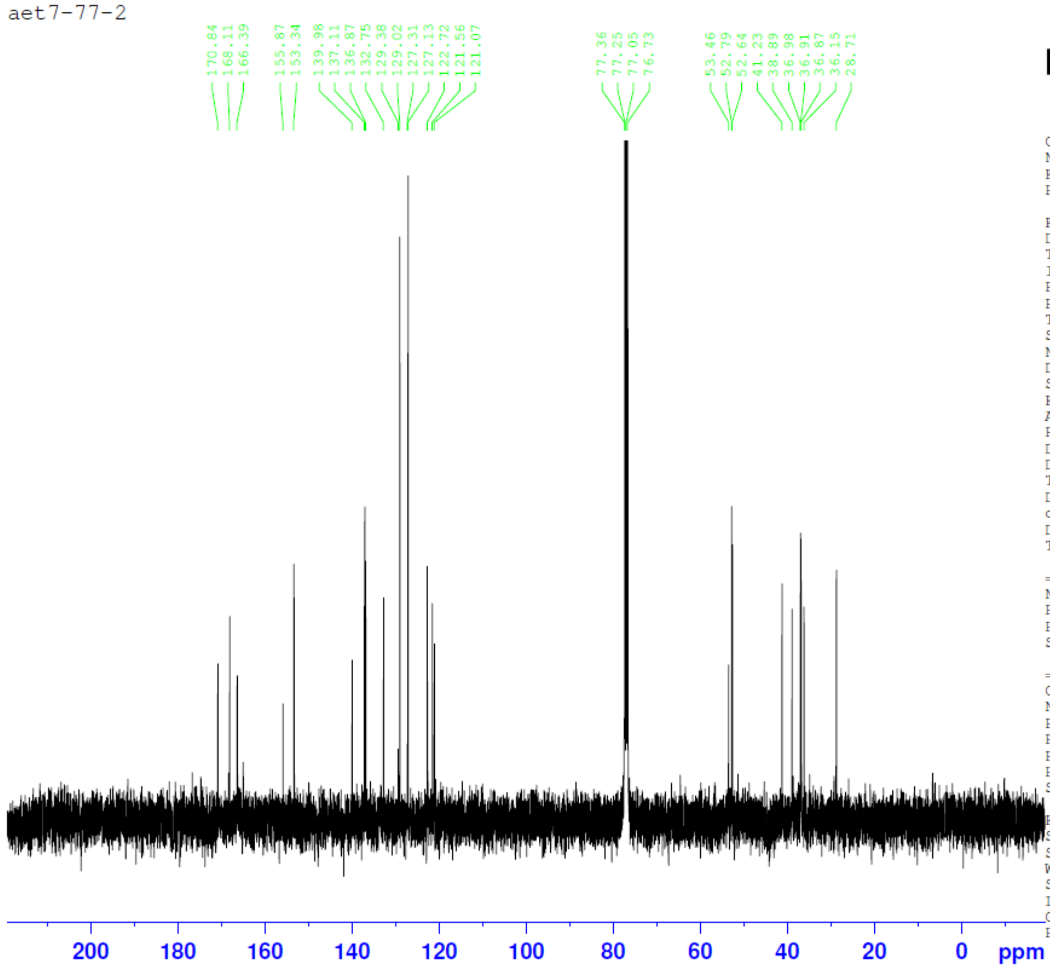


```
Current Data Parameters
NAME            aet7-77-2
EXPNO                  11
PROCNO                  1

F2 - Acquisition Parameters
Date_            20120327
Time                21.12
INSTRUM            DPX400
PROBHD    5 mm QNP 1H/13
PULPROG            zgpg30
TD                  65536
SOLVENT             CDCl3
NS                   1024
DS                      4
SWH             23980.814 Hz
FIDRES           0.365918 Hz
AQ              1.3664756 sec
RG                  724.1
DW                 20.850 usec
DE                   6.00 usec
TE                  294.2 K
D1             2.00000000 sec
d11            0.03000000 sec
DELTA          1.89999998 sec
TD0                     1

======== CHANNEL f1 ========
NUC1                  13C
P1                   8.50 usec
PL1                 -2.60 dB
SFO1          100.6228298 MHz

======== CHANNEL f2 ========
CPDPRG2           waltz16
NUC2                   1H
PCPD2               80.00 usec
PL2                 -1.50 dB
PL12                17.50 dB
PL13                17.50 dB
SFO2          400.1316005 MHz

F2 - Processing parameters
SI                  32768
SF            100.6127690 MHz
WDW                    EM
SSB                     0
LB                   1.00 Hz
GB                      0
PC                   1.40
```

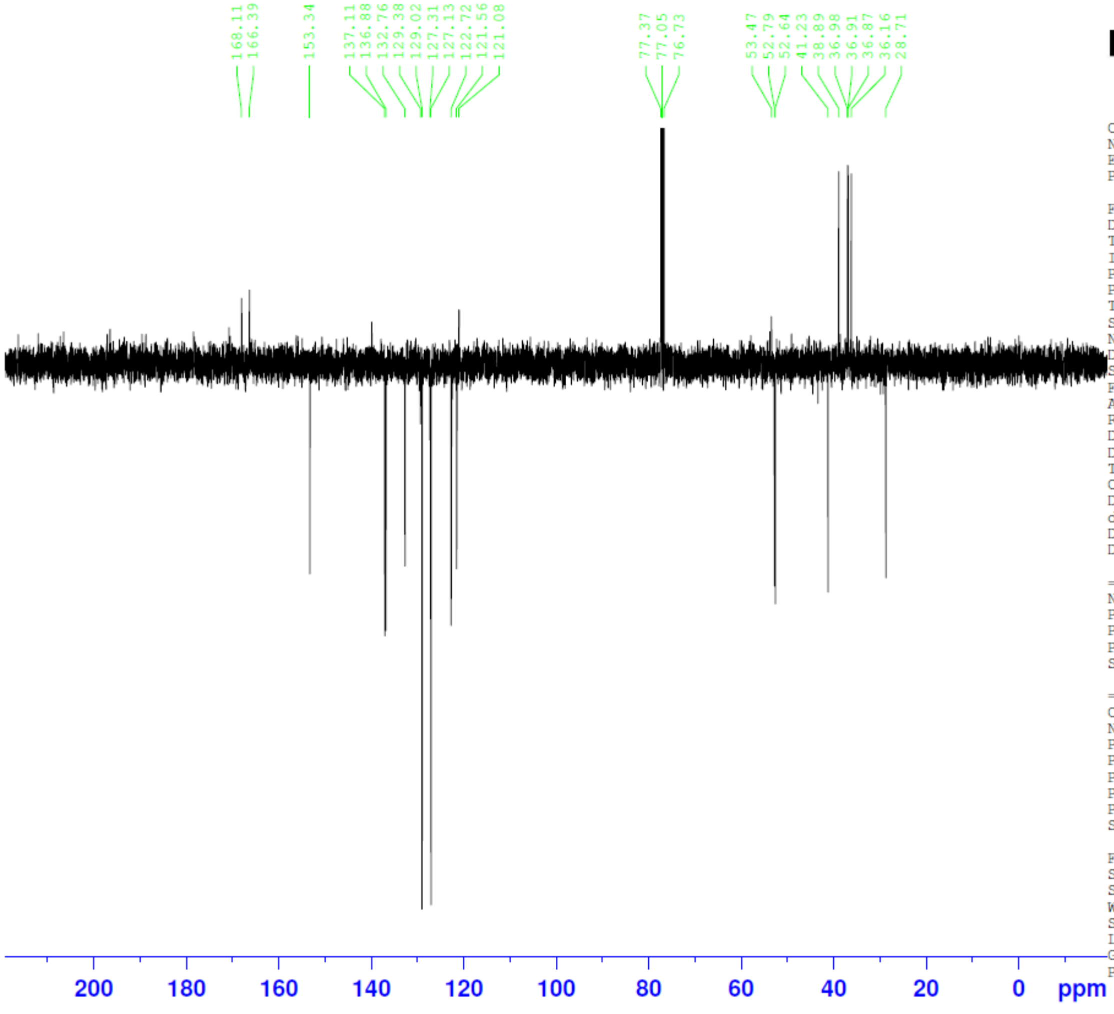


```
Current Data Parameters
NAME            aet7-77-2
EXPNO                  12
PROCNO                  1

F2 - Acquisition Parameters
Date_            20120327
Time                21.42
INSTRUM            DPX400
PROBHD    5 mm QNP 1H/13
PULPROG         deptq_135
TD                  65536
SOLVENT             CDCl3
NS                    512
DS                      4
SWH             23980.814 Hz
FIDRES           0.365918 Hz
AQ              1.3664756 sec
RG                11585.2
DW                 20.850 usec
DE                   6.00 usec
TE                  294.2 K
CNST2         145.0000000
D1             2.00000000 sec
d2             0.00344828 sec
D11            0.03000000 sec
DELTA          0.00001082 sec

======== CHANNEL f1 ========
NUC1                  13C
P1                   8.50 usec
P2                  17.00 usec
PL1                 -2.60 dB
SFO1          100.6228300 MHz

======== CHANNEL f2 ========
CPDPRG2           waltz16
NUC2                   1H
P3                  10.00 usec
P4                  20.00 usec
PCPD2               80.00 usec
PL2                 -1.50 dB
PL12                17.50 dB
SFO2          400.1316000 MHz

F2 - Processing parameters
SI                  32768
SF            100.6127690 MHz
WDW                    EM
SSB                     0
LB                   1.00 Hz
GB                      0
PC                   1.40
```

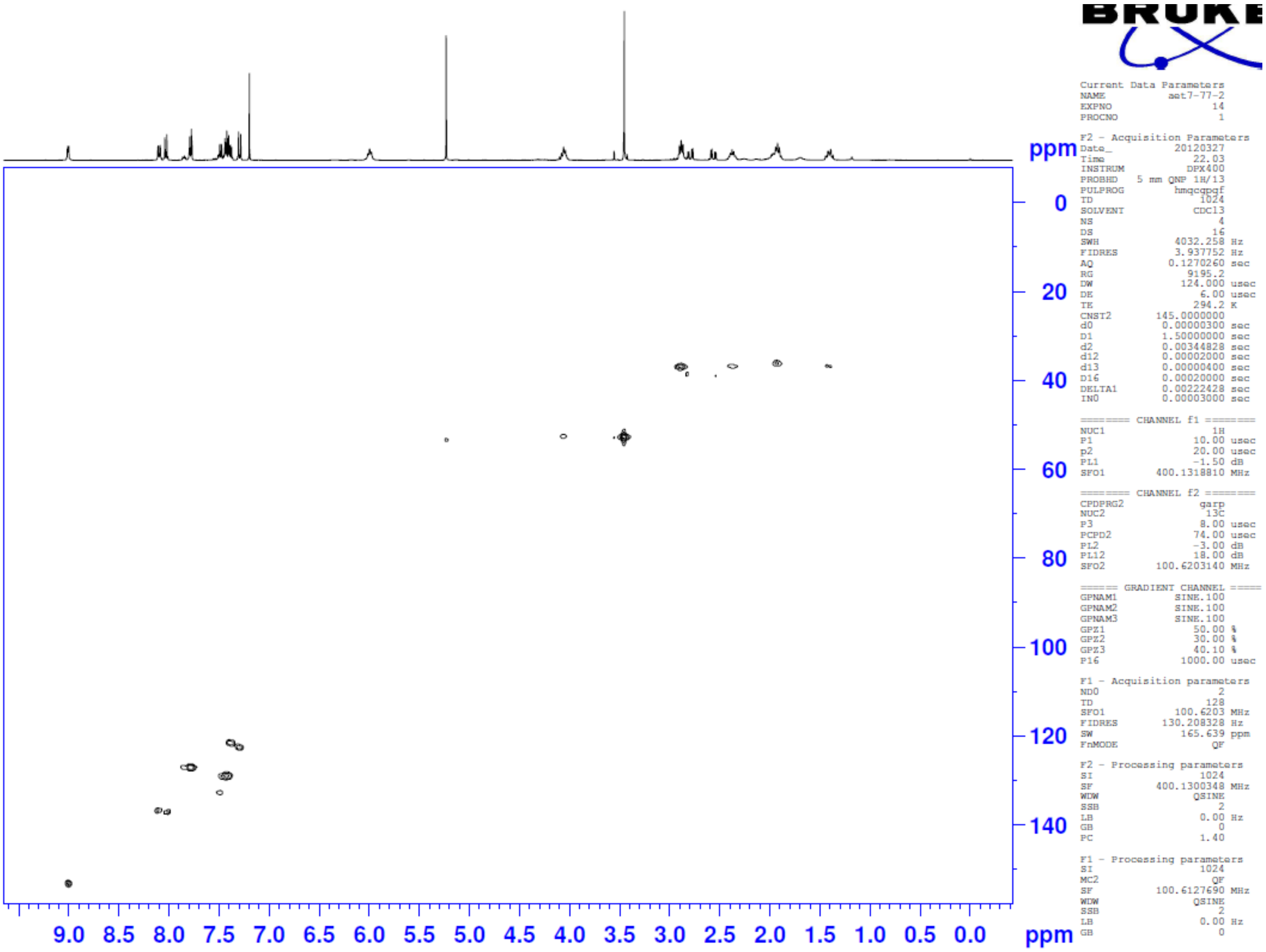

BRUKER
Current Data Parameters
NAME aet7-77-2
EXPNO 14
PROCNO 1
F2 - Acquisition Parameters
Date_ 20120327
Time 22.03
INSTRUM DPX400
PROBHD 5 mm QNP 1H/13
PULPROG hmqcgpqf
TD 1024
SOLVENT CDCl3
NS 4
DS 16
SWH 4032.258 Hz
FIDRES 3.937752 Hz
AQ 0.1270260 sec
RG 9195.2
DW 124.000 usec
DE 6.00 usec
TE 294.2 K
CNST2 145.0000000
d0 0.00000300 sec
D1 1.50000000 sec
d2 0.00344828 sec
d12 0.00002000 sec
d13 0.00000400 sec
D16 0.00020000 sec
DELTA1 0.00222428 sec
IN0 0.00003000 sec
======== CHANNEL f1 ========
NUC1 1H
P1 10.00 usec
p2 20.00 usec
PL1 -1.50 dB
SFO1 400.1318810 MHz
======== CHANNEL f2 ========
CPDPRG2 garp
NUC2 13C
P3 8.00 usec
PCPD2 74.00 usec
PL2 -3.00 dB
PL12 18.00 dB
SFO2 100.6203140 MHz
===== GRADIENT CHANNEL =====
GPNAM1 SINE.100
GPNAM2 SINE.100
GPNAM3 SINE.100
GPZ1 50.00 %
GPZ2 30.00 %
GPZ3 40.10 %
P16 1000.00 usec
F1 - Acquisition parameters
ND0 2
TD 128
SFO1 100.6203 MHz
FIDRES 130.208328 Hz
SW 165.639 ppm
FnMODE QF
F2 - Processing parameters
SI 1024
SF 400.1300348 MHz
WDW QSINE
SSB 2
LB 0.00 Hz
GB 0
PC 1.40
F1 - Processing parameters
SI 1024
MC2 QF
SF 100.6127690 MHz
WDW QSINE
SSB 2
LB 0.00 Hz
GB 0
ppm
0
20
40
60
80
100
120
140
9.0 8.5 8.0 7.5 7.0 6.5 6.0 5.5 5.0 4.5 4.0 3.5 3.0 2.5 2.0 1.5 1.0 0.5 0.0
ppm

Methyl N^4-((1*r,3*R**)-3-(2-(1,8-naphthyridin-2-yl)ethyl)cyclobutyl)-N^2-(mesitylsulfonyl)-*L*-asparaginate [Compound 29]**

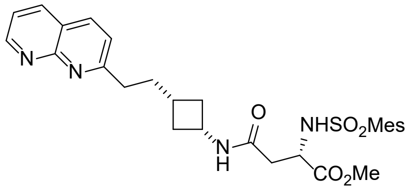

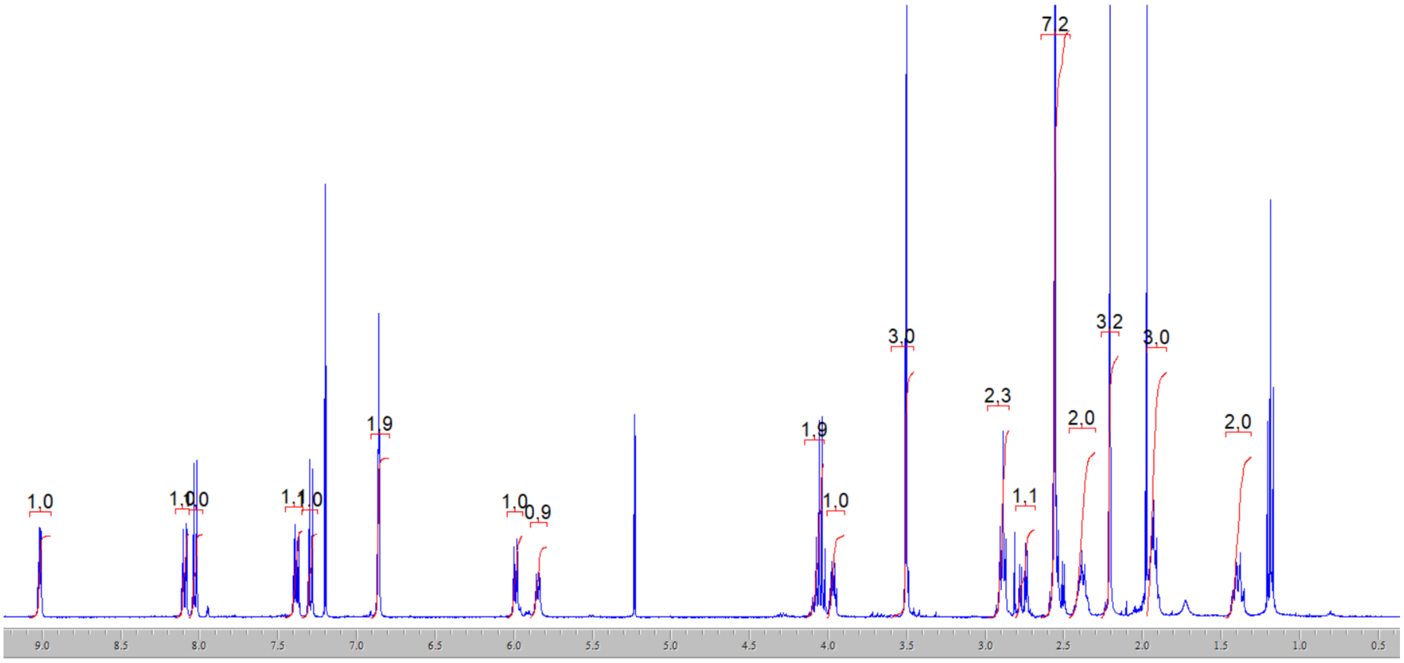

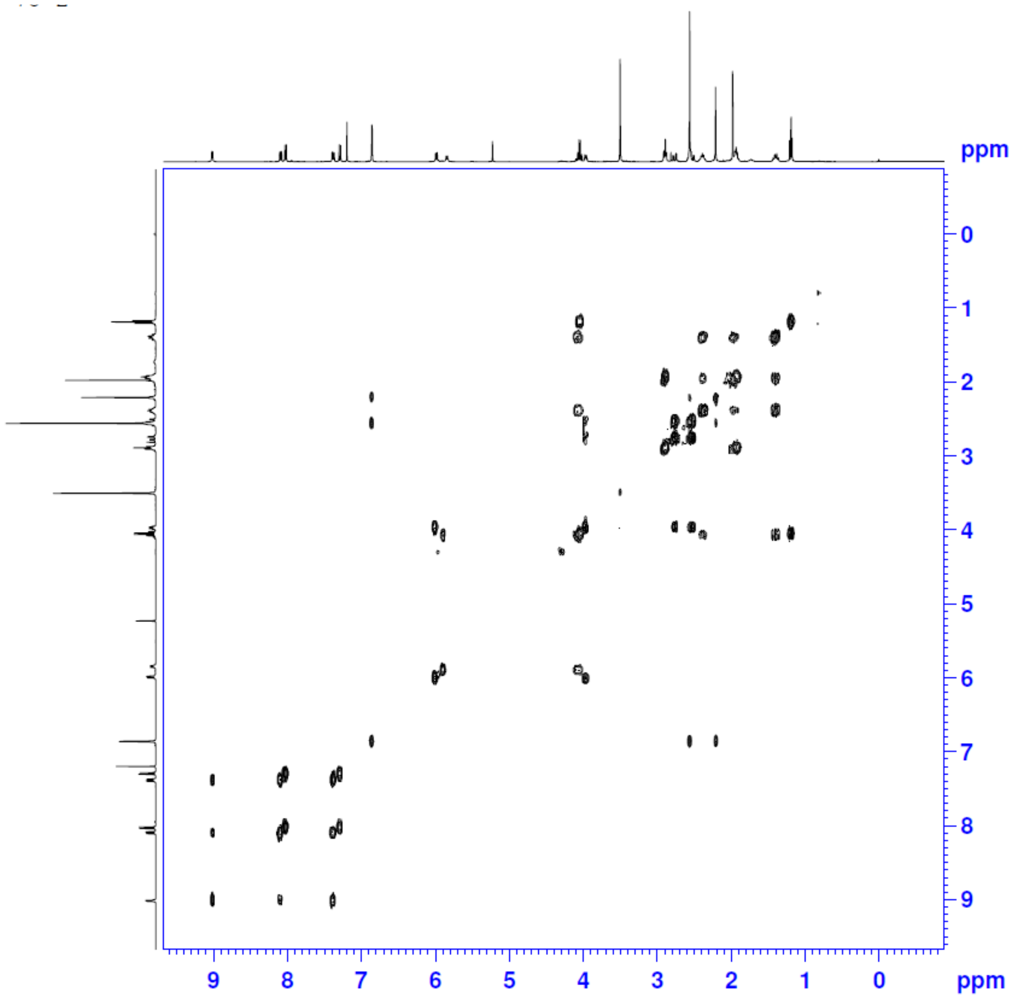


```
Current Data Parameters
NAME          aet7-78-2
EXPNO                13
PROCNO                1

F2 - Acquisition Parameters
Date_          20120329
Time               1.57
INSTRUM          DPX400
PROBHD   5 mm QNP 1H/13
PULPROG     cosygpmfqf
TD                 2048
SOLVENT           CDCl3
NS                    4
DS                    8
SWH            4222.973 Hz
FIDRES         2.061999 Hz
AQ            0.2425332 sec
RG              11585.2
DW              118.400 usec
DE                 6.00 usec
TE                294.2 K
D0           0.00000300 sec
D1           1.95002902 sec
d13          0.00000400 sec
D16          0.00020000 sec
IN0          0.00023680 sec

======= CHANNEL f1 =======
NUC1                 1H
P1                10.00 usec
PL1               -1.50 dB
SFO1        400.1317944 MHz

====== GRADIENT CHANNEL =====
GPNAM1         SINE.100
GPNAM2         SINE.100
GPNAM3         SINE.100
GPZ1              16.00 %
GPZ2              12.00 %
GPZ3              40.00 %
P16             1000.00 usec

F1 - Acquisition parameters
ND0                   1
TD                  128
SFO1           400.1318 MHz
FIDRES        32.991978 Hz
SW               10.554 ppm
FnMODE               QF

F2 - Processing parameters
SI                 1024
SF          400.1300342 MHz
WDW                SINE
SSB                   0
LB                 0.00 Hz
GB                    0
PC                 1.40

F1 - Processing parameters
SI                 1024
MC2                  QF
SF          400.1300342 MHz
WDW                SINE
SSB                   0
LB                 0.00 Hz
GB                    0
```

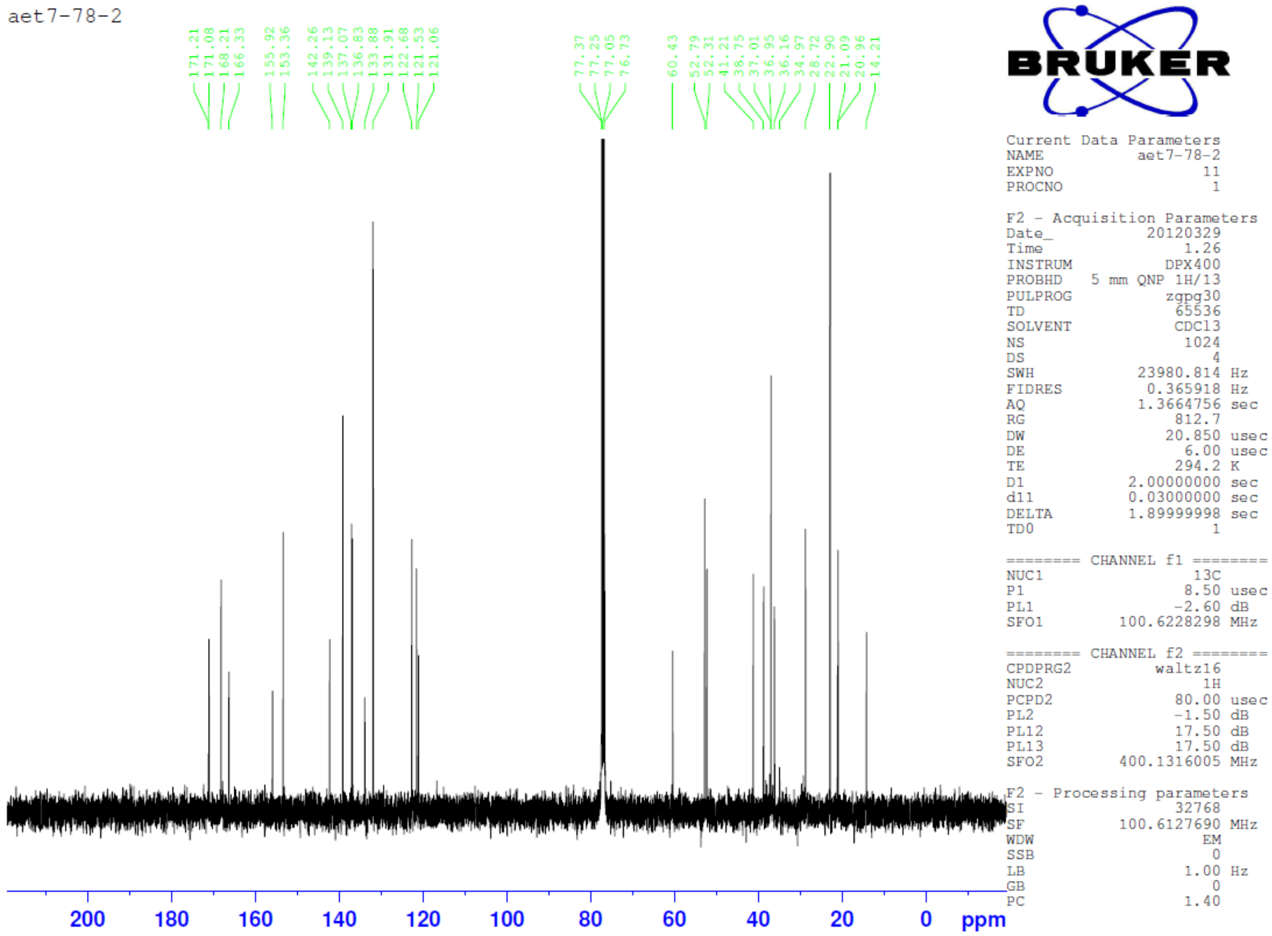
aet7-78-2
171.21
171.08
168.21
166.33
155.92
153.36
142.26
139.13
137.07
136.83
133.88
131.91
122.68
121.53
121.06
77.37
77.25
77.05
76.73
60.43
52.79
52.31
41.21
38.75
37.01
36.95
36.16
34.97
28.72
22.90
21.09
20.96
14.21
BRUKER
Current Data Parameters
NAME aet7-78-2
EXPNO 11
PROCNO 1
F2 - Acquisition Parameters
Date_ 20120329
Time 1.26
INSTRUM DPX400
PROBHD 5 mm QNP 1H/13
PULPROG zgpg30
TD 65536
SOLVENT CDCl3
NS 1024
DS 4
SWH 23980.814 Hz
FIDRES 0.365918 Hz
AQ 1.3664756 sec
RG 812.7
DW 20.850 usec
DE 6.00 usec
TE 294.2 K
D1 2.00000000 sec
d11 0.03000000 sec
DELTA 1.89999998 sec
TD0 1
======== CHANNEL f1 ========
NUC1 13C
P1 8.50 usec
PL1 -2.60 dB
SFO1 100.6228298 MHz
======== CHANNEL f2 ========
CPDPRG2 waltz16
NUC2 1H
PCPD2 80.00 usec
PL2 -1.50 dB
PL12 17.50 dB
PL13 17.50 dB
SFO2 400.1316005 MHz
F2 - Processing parameters
SI 32768
SF 100.6127690 MHz
WDW EM
SSB 0
LB 1.00 Hz
GB 0
PC 1.40
200
180
160
140
120
100
80
60
40
20
0
ppm

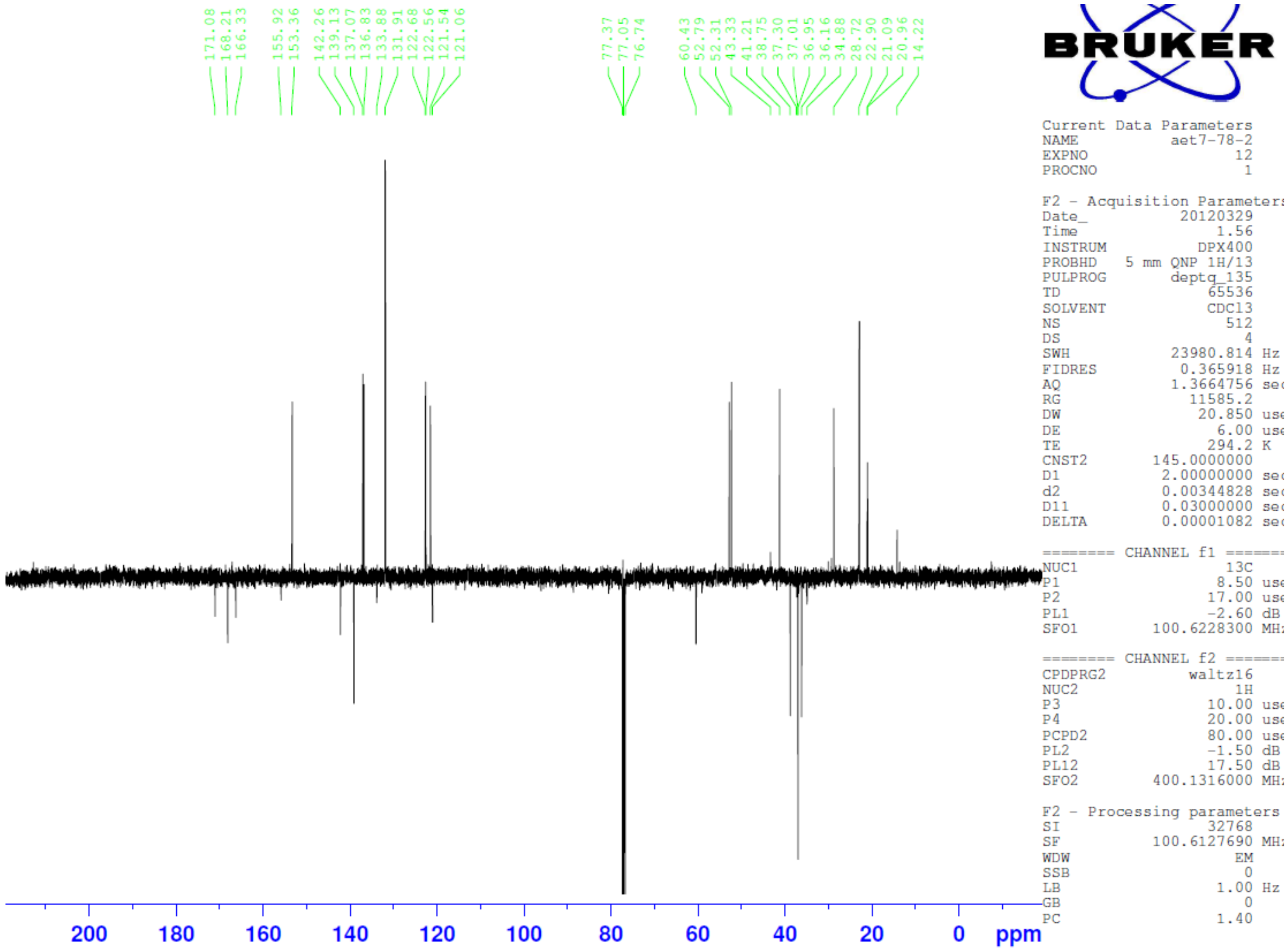
171.08
168.21
166.33
155.92
153.36
142.26
139.13
137.07
136.83
133.88
131.91
122.68
122.56
121.54
121.06
77.37
77.05
76.74
60.43
52.79
52.31
43.33
41.21
38.75
37.30
37.01
36.95
36.16
34.88
28.72
22.90
21.09
20.96
14.22
BRUKER
Current Data Parameters
NAME aet7-78-2
EXPNO 12
PROCNO 1
F2 - Acquisition Parameter
Date_ 20120329
Time 1.56
INSTRUM DPX400
PROBHD 5 mm QNP 1H/13
PULPROG deptq_135
TD 65536
SOLVENT CDCl3
NS 512
DS 4
SWH 23980.814 Hz
FIDRES 0.365918 Hz
AQ 1.3664756
RG 11585.2
DW 20.850
DE 6.00
TE 294.2 K
CNST2 145.0000000
D1 2.00000000
d2 0.00344828
D11 0.03000000
DELTA 0.00001082
======== CHANNEL f1
NUC1 13C
P1 8.50
P2 17.00
PL1 -2.60 dB
SFO1 100.6228300
======== CHANNEL f2
CPDPRG2 waltz16
NUC2 1H
P3 10.00
P4 20.00
PCPD2 80.00
PL2 -1.50 dB
PL12 17.50 dB
SFO2 400.1316000
F2 - Processing parameters
SI 32768
SF 100.6127690
WDW EM
SSB 0
LB 1.00 Hz
GB 0
PC 1.40
200
180
160
140
120
100
80
60
40
20
0
ppm

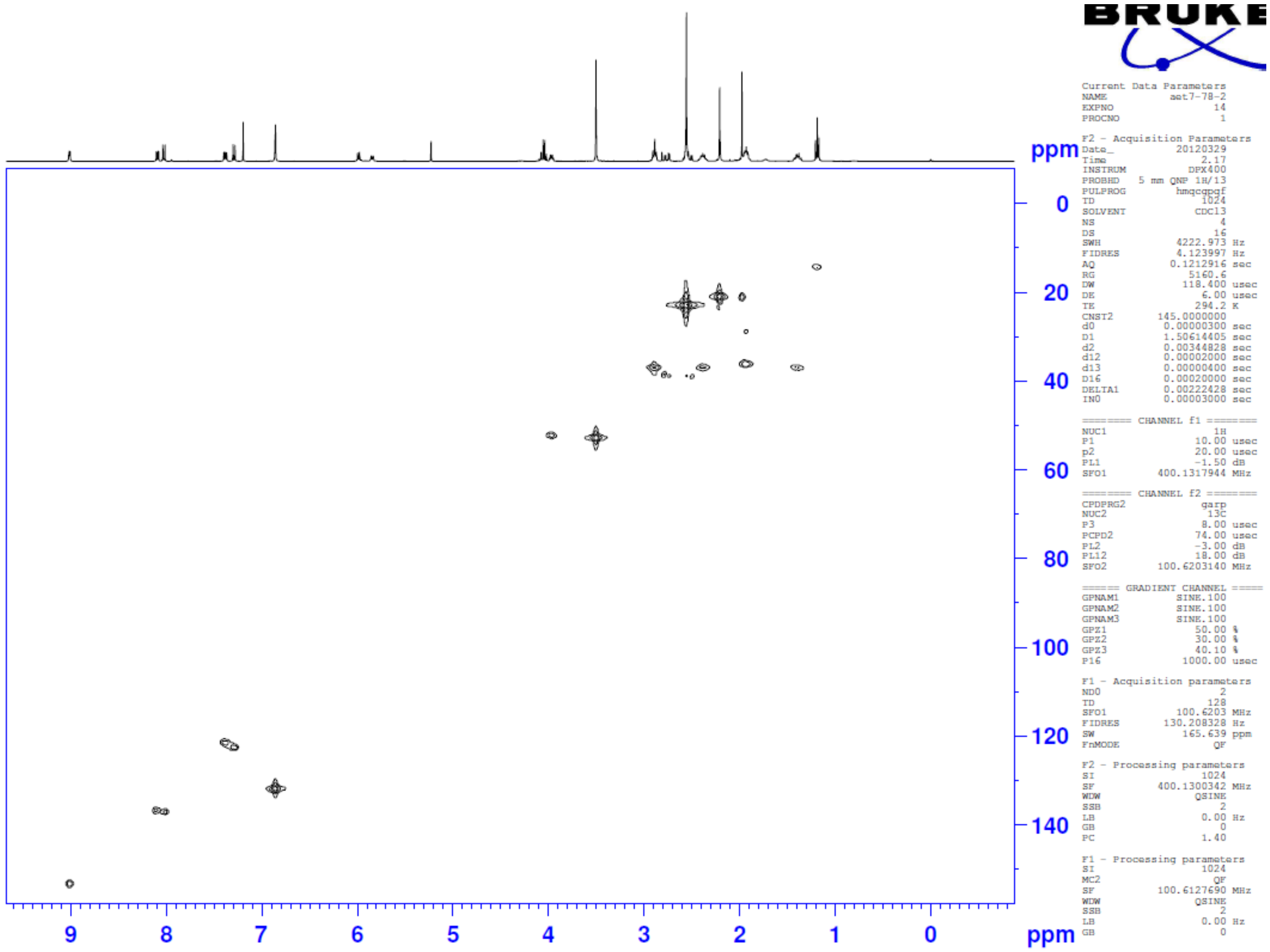

ppm
0
20
40
60
80
100
120
140
9
8
7
6
5
4
3
2
1
0
ppm
Current Data Parameters
NAME aet7-78-2
EXPNO 14
PROCNO 1
F2 - Acquisition Parameters
Date_ 20120329
Time 2.17
INSTRUM DPX400
PROBHD 5 mm QNP 1H/13
PULPROG hmqcgpqf
TD 1024
SOLVENT CDCl3
NS 4
DS 16
SWH 4222.973 Hz
FIDRES 4.123997 Hz
AQ 0.1212916 sec
RG 5160.6
DW 118.400 usec
DE 6.00 usec
TE 294.2 K
CNST2 145.0000000
d0 0.00000300 sec
D1 1.50614405 sec
d2 0.00344828 sec
d12 0.00002000 sec
d13 0.00000400 sec
D16 0.00020000 sec
DELTA1 0.00222428 sec
IN0 0.00003000 sec
======= CHANNEL f1 =======
NUC1 1H
P1 10.00 usec
p2 20.00 usec
PL1 -1.50 dB
SFO1 400.1317944 MHz
======= CHANNEL f2 =======
CPDPRG2 garp
NUC2 13C
P3 8.00 usec
PCPD2 74.00 usec
PL2 -3.00 dB
PL12 18.00 dB
SFO2 100.6203140 MHz
===== GRADIENT CHANNEL =====
GPNAM1 SINE.100
GPNAM2 SINE.100
GPNAM3 SINE.100
GPZ1 50.00 %
GPZ2 30.00 %
GPZ3 40.10 %
P16 1000.00 usec
F1 - Acquisition parameters
ND0 2
TD 128
SFO1 100.6203 MHz
FIDRES 130.208328 Hz
SW 165.639 ppm
FnMODE QF
F2 - Processing parameters
SI 1024
SF 400.1300342 MHz
WDW QSINE
SSB 2
LB 0.00 Hz
GB 0
PC 1.40
F1 - Processing parameters
SI 1024
MC2 QF
SF 100.6127690 MHz
WDW QSINE
SSB 2
LB 0.00 Hz
GB 0

_N_-((_S_)-1-((1_r_*,3_R_*)-3-(2-(1,8-Naphthyridin-2-yl)ethyl)cyclobutyl)-2,5-dioxopyrrolidin-3-yl)benzenesulfonamide [Compound 30]

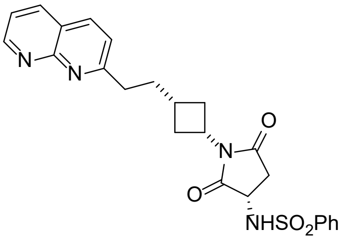

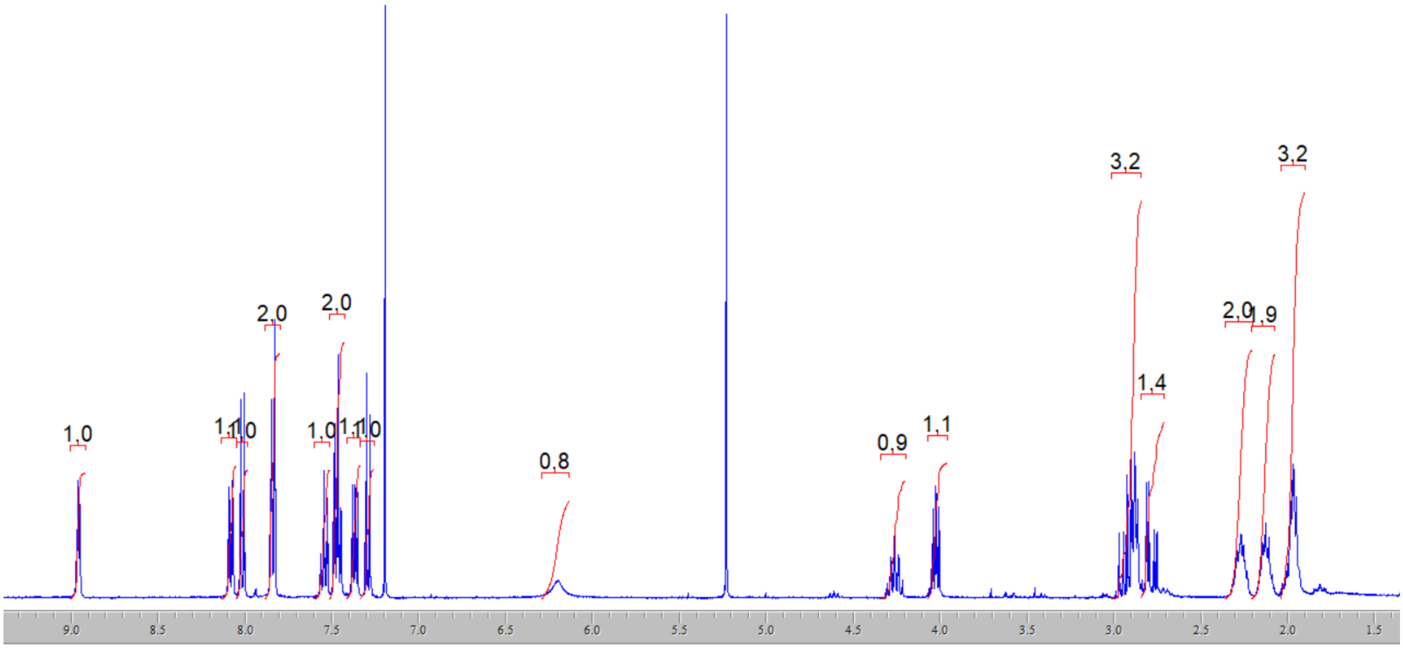

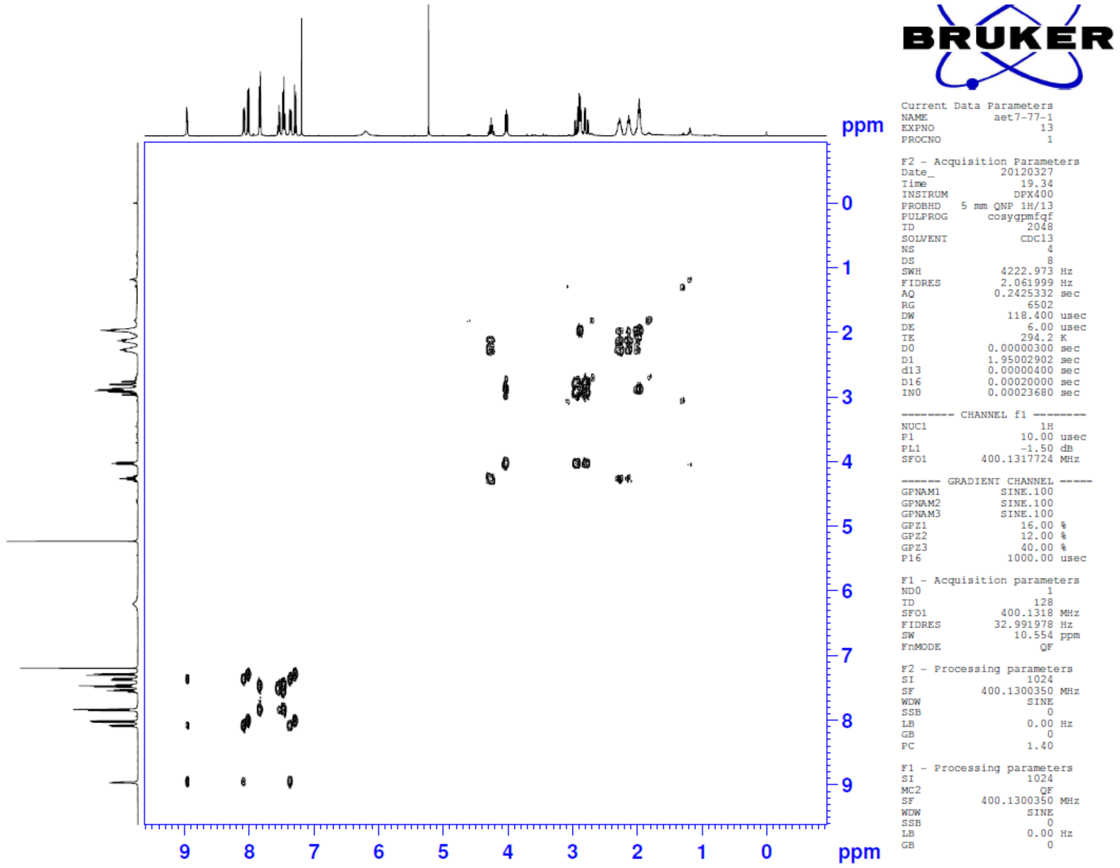

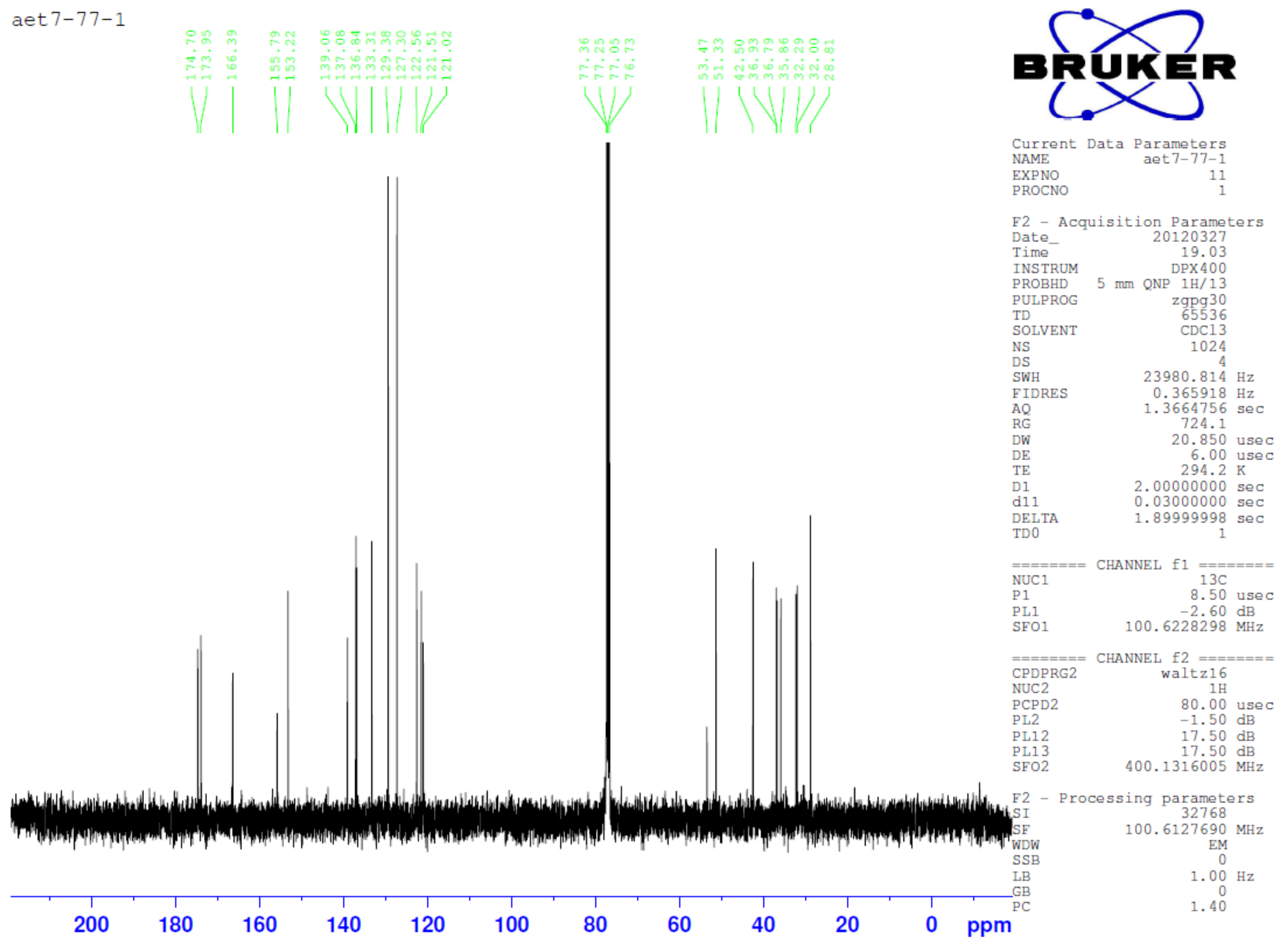
aet7-77-1
174.70
173.95
166.39
155.79
153.22
139.06
137.08
136.84
133.31
129.38
127.30
122.56
121.51
121.02
77.36
77.25
77.05
76.73
53.47
51.33
42.50
36.93
36.79
35.86
32.29
32.00
28.81
200 180 160 140 120 100 80 60 40 20 0 ppm
Current Data Parameters
NAME aet7-77-1
EXPNO 11
PROCNO 1
F2 - Acquisition Parameters
Date_ 20120327
Time 19.03
INSTRUM DPX400
PROBHD 5 mm QNP 1H/13
PULPROG zgpg30
TD 65536
SOLVENT CDCl3
NS 1024
DS 4
SWH 23980.814 Hz
FIDRES 0.365918 Hz
AQ 1.3664756 sec
RG 724.1
DW 20.850 usec
DE 6.00 usec
TE 294.2 K
D1 2.00000000 sec
d11 0.03000000 sec
DELTA 1.89999998 sec
TD0 1
======== CHANNEL f1 ========
NUC1 13C
P1 8.50 usec
PL1 -2.60 dB
SFO1 100.6228298 MHz
======== CHANNEL f2 ========
CPDPRG2 waltz16
NUC2 1H
PCPD2 80.00 usec
PL2 -1.50 dB
PL12 17.50 dB
PL13 17.50 dB
SFO2 400.1316005 MHz
F2 - Processing parameters
SI 32768
SF 100.6127690 MHz
WDW EM
SSB 0
LB 1.00 Hz
GB 0
PC 1.40

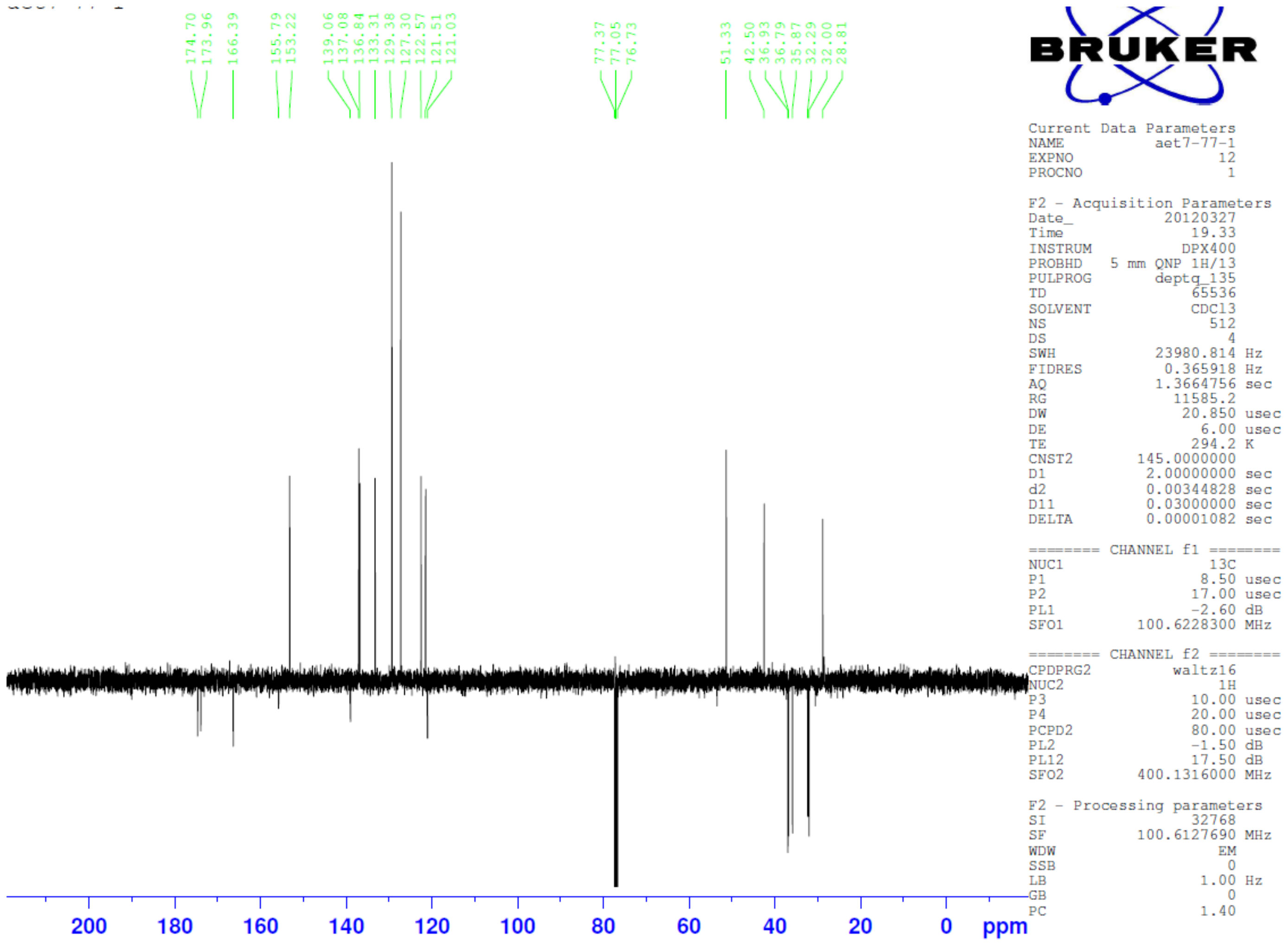
174.70
173.96
166.39
155.79
153.22
139.06
137.08
136.84
133.31
129.38
127.30
122.57
121.51
121.03
77.37
77.05
76.73
51.33
42.50
36.93
36.79
35.87
32.29
32.00
28.81
200 180 160 140 120 100 80 60 40 20 0 ppm
Current Data Parameters
NAME aet7-77-1
EXPNO 12
PROCNO 1
F2 - Acquisition Parameters
Date_ 20120327
Time 19.33
INSTRUM DPX400
PROBHD 5 mm QNP 1H/13
PULPROG deptq_135
TD 65536
SOLVENT CDCl3
NS 512
DS 4
SWH 23980.814 Hz
FIDRES 0.365918 Hz
AQ 1.3664756 sec
RG 11585.2
DW 20.850 usec
DE 6.00 usec
TE 294.2 K
CNST2 145.0000000
D1 2.00000000 sec
d2 0.00344828 sec
D11 0.03000000 sec
DELTA 0.00001082 sec
======== CHANNEL f1 ========
NUC1 13C
P1 8.50 usec
P2 17.00 usec
PL1 -2.60 dB
SFO1 100.6228300 MHz
======== CHANNEL f2 ========
CPDPRG2 waltz16
NUC2 1H
P3 10.00 usec
P4 20.00 usec
PCPD2 80.00 usec
PL2 -1.50 dB
PL12 17.50 dB
SFO2 400.1316000 MHz
F2 - Processing parameters
SI 32768
SF 100.6127690 MHz
WDW EM
SSB 0
LB 1.00 Hz
GB 0
PC 1.40

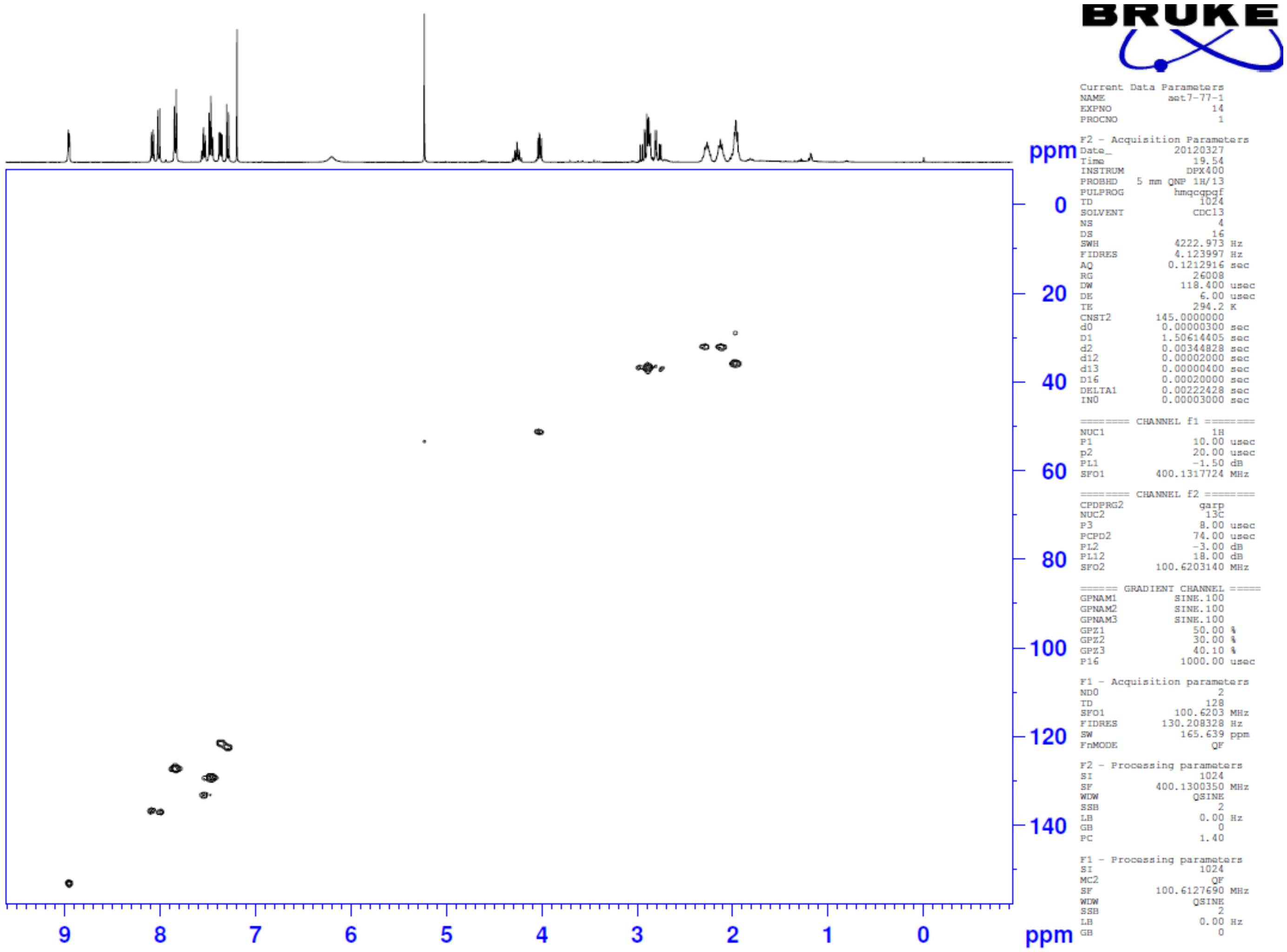

BRUKE
Current Data Parameters
NAME aet7-77-1
EXPNO 14
PROCNO 1
F2 - Acquisition Parameters
Date_ 20120327
Time 19.54
INSTRUM DPX400
PROBHD 5 mm QNP 1H/13
PULPROG hmqcgpqf
TD 1024
SOLVENT CDCl3
NS 4
DS 16
SWH 4222.973 Hz
FIDRES 4.123997 Hz
AQ 0.1212916 sec
RG 26008
DW 118.400 usec
DE 6.00 usec
TE 294.2 K
CNST2 145.0000000
d0 0.00000300 sec
D1 1.50614405 sec
d2 0.00344828 sec
d12 0.00002000 sec
d13 0.00000400 sec
D16 0.00020000 sec
DELTA1 0.00222428 sec
IN0 0.00003000 sec
======== CHANNEL f1 ========
NUC1 1H
P1 10.00 usec
p2 20.00 usec
PL1 -1.50 dB
SFO1 400.1317724 MHz
======== CHANNEL f2 ========
CPDPRG2 garp
NUC2 13C
P3 8.00 usec
PCPD2 74.00 usec
PL2 -3.00 dB
PL12 18.00 dB
SFO2 100.6203140 MHz
====== GRADIENT CHANNEL =====
GPNAM1 SINE.100
GPNAM2 SINE.100
GPNAM3 SINE.100
GPZ1 50.00 %
GPZ2 30.00 %
GPZ3 40.10 %
P16 1000.00 usec
F1 - Acquisition parameters
ND0 2
TD 128
SFO1 100.6203 MHz
FIDRES 130.208328 Hz
SW 165.639 ppm
FnMODE QF
F2 - Processing parameters
SI 1024
SF 400.1300350 MHz
WDW QSINE
SSB 2
LB 0.00 Hz
GB 0
PC 1.40
F1 - Processing parameters
SI 1024
MC2 QF
SF 100.6127690 MHz
WDW QSINE
SSB 2
LB 0.00 Hz
GB 0
ppm
0
20
40
60
80
100
120
140
9
8
7
6
5
4
3
2
1
0
ppm

_N_-((_S_)-1-((1_r_*,3_R_*)-3-(2-(1,8-Naphthyridin-2-yl)ethyl)cyclobutyl)-2,5-dioxopyrrolidin-3-yl)-2,4,6-trimethylbenzenesulfonamide [Compound 31]

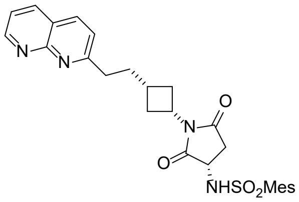

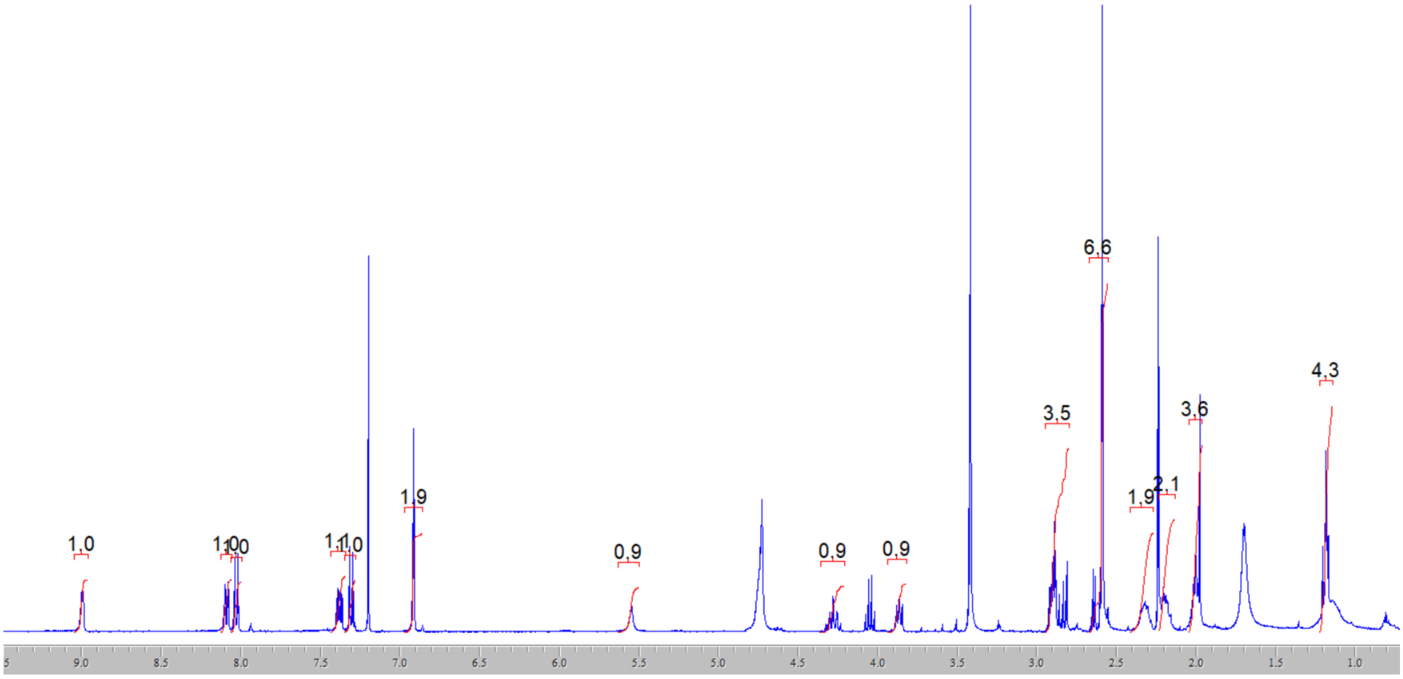

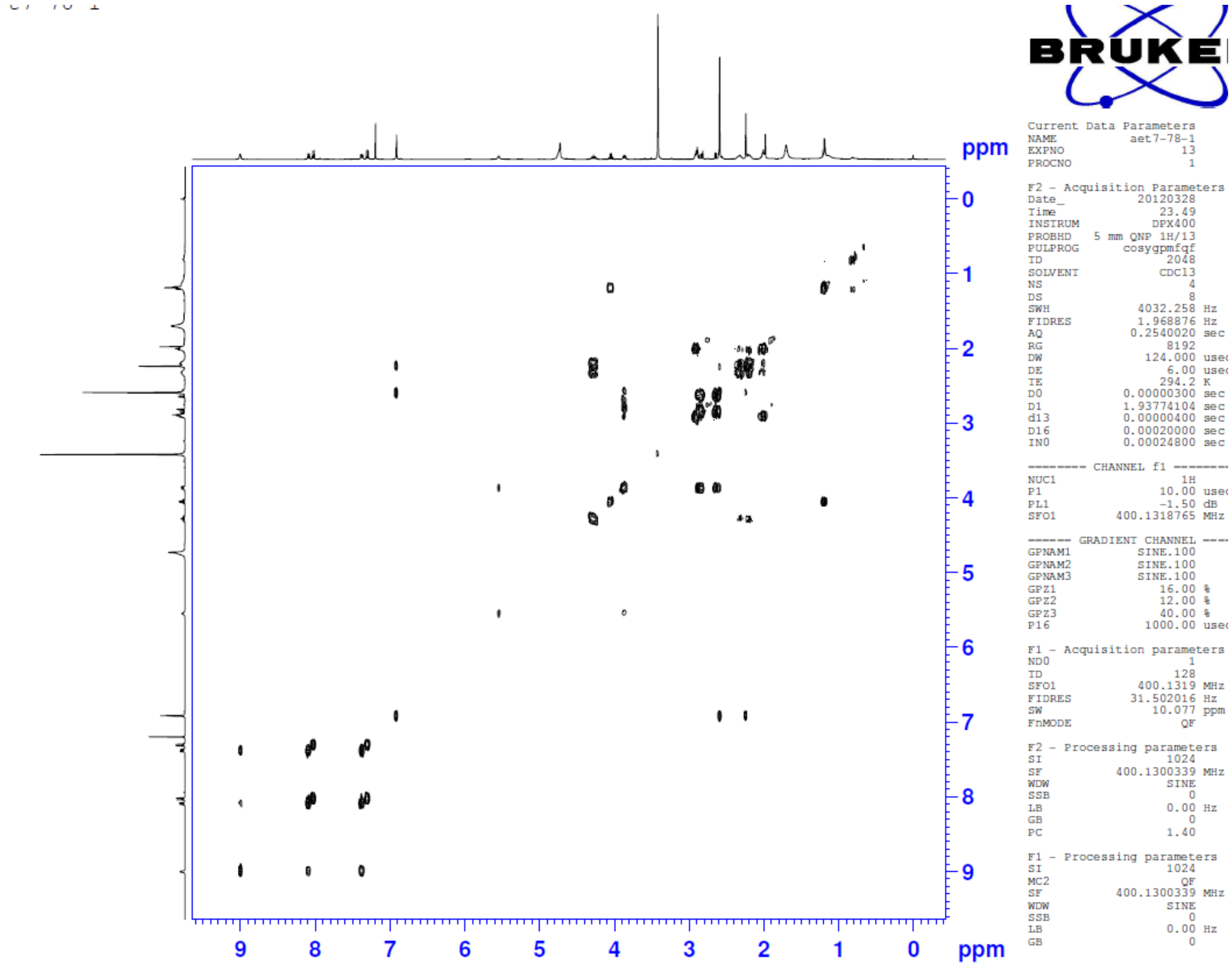

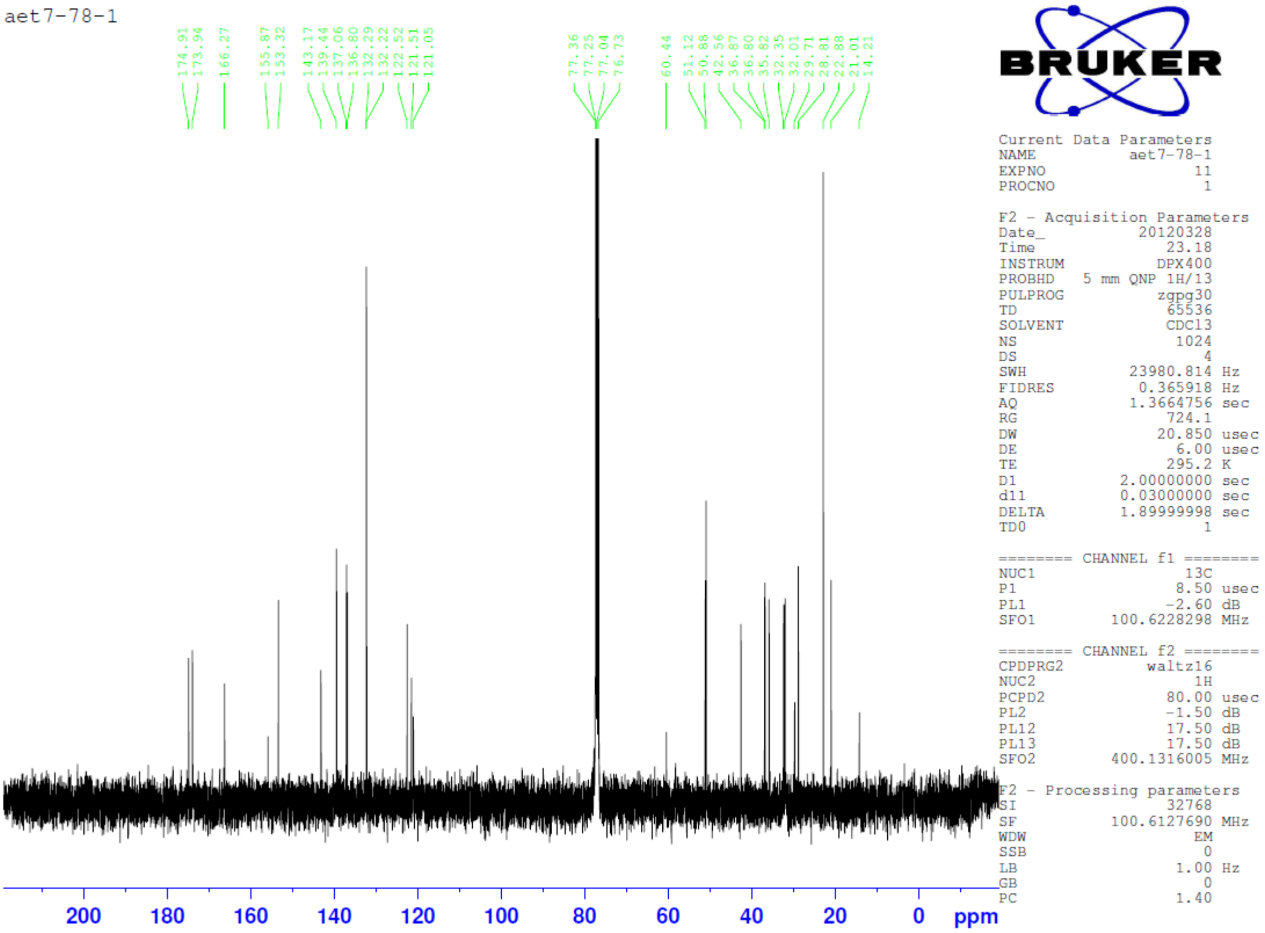
aet7-78-1
174.91 173.94 166.27 155.87 153.32 143.17 139.44 137.06 136.80 132.29 132.22 122.52 121.51 121.05 77.36 77.25 77.04 76.73 60.44 51.12 50.88 42.56 36.87 36.80 35.82 32.35 32.01 29.71 28.81 22.88 21.01 14.21
BRUKER
Current Data Parameters
NAME aet7-78-1
EXPNO 11
PROCNO 1
F2 - Acquisition Parameters
Date_ 20120328
Time 23.18
INSTRUM DPX400
PROBHD 5 mm QNP 1H/13
PULPROG zgpg30
TD 65536
SOLVENT CDCl3
NS 1024
DS 4
SWH 23980.814 Hz
FIDRES 0.365918 Hz
AQ 1.3664756 sec
RG 724.1
DW 20.850 usec
DE 6.00 usec
TE 295.2 K
D1 2.00000000 sec
d11 0.03000000 sec
DELTA 1.89999998 sec
TD0 1
======== CHANNEL f1 ========
NUC1 13C
P1 8.50 usec
PL1 -2.60 dB
SFO1 100.6228298 MHz
======== CHANNEL f2 ========
CPDPRG2 waltz16
NUC2 1H
PCPD2 80.00 usec
PL2 -1.50 dB
PL12 17.50 dB
PL13 17.50 dB
SFO2 400.1316005 MHz
F2 - Processing parameters
SI 32768
SF 100.6127690 MHz
WDW EM
SSB 0
LB 1.00 Hz
GB 0
PC 1.40
200 180 160 140 120 100 80 60 40 20 0 ppm

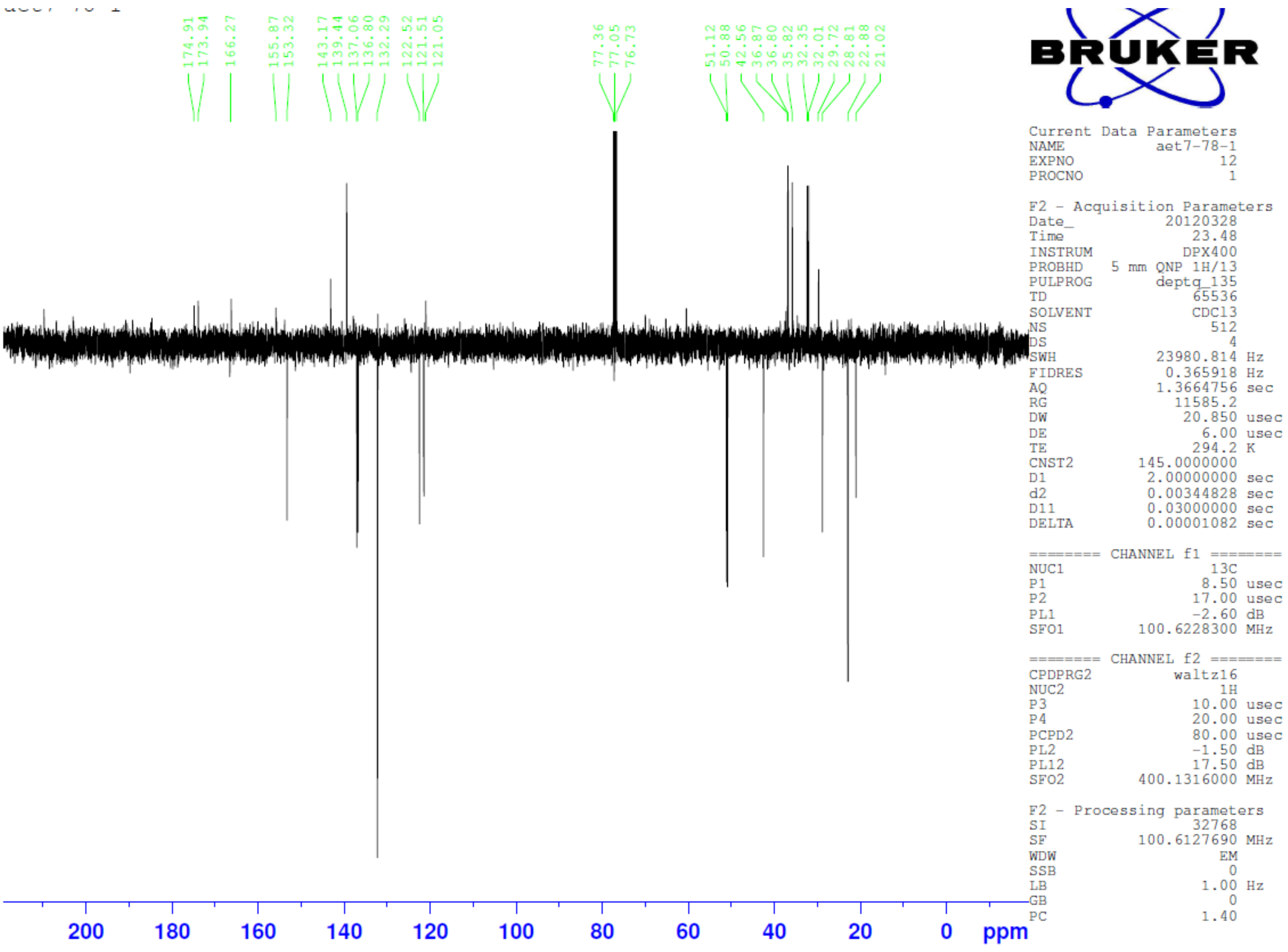
174.91 173.94 166.27 155.87 153.32 143.17 139.44 137.06 136.80 132.29 122.52 121.51 121.05 77.36 77.05 76.73 51.12 50.88 42.56 36.87 36.80 35.82 32.35 32.01 29.72 28.81 22.88 21.02
BRUKER
Current Data Parameters
NAME aet7-78-1
EXPNO 12
PROCNO 1
F2 - Acquisition Parameters
Date_ 20120328
Time 23.48
INSTRUM DPX400
PROBHD 5 mm QNP 1H/13
PULPROG deptq_135
TD 65536
SOLVENT CDCl3
NS 512
DS 4
SWH 23980.814 Hz
FIDRES 0.365918 Hz
AQ 1.3664756 sec
RG 11585.2
DW 20.850 usec
DE 6.00 usec
TE 294.2 K
CNST2 145.0000000
D1 2.00000000 sec
d2 0.00344828 sec
D11 0.03000000 sec
DELTA 0.00001082 sec
======== CHANNEL f1 ========
NUC1 13C
P1 8.50 usec
P2 17.00 usec
PL1 -2.60 dB
SFO1 100.6228300 MHz
======== CHANNEL f2 ========
CPDPRG2 waltz16
NUC2 1H
P3 10.00 usec
P4 20.00 usec
PCPD2 80.00 usec
PL2 -1.50 dB
PL12 17.50 dB
SFO2 400.1316000 MHz
F2 - Processing parameters
SI 32768
SF 100.6127690 MHz
WDW EM
SSB 0
LB 1.00 Hz
GB 0
PC 1.40
200 180 160 140 120 100 80 60 40 20 0 ppm

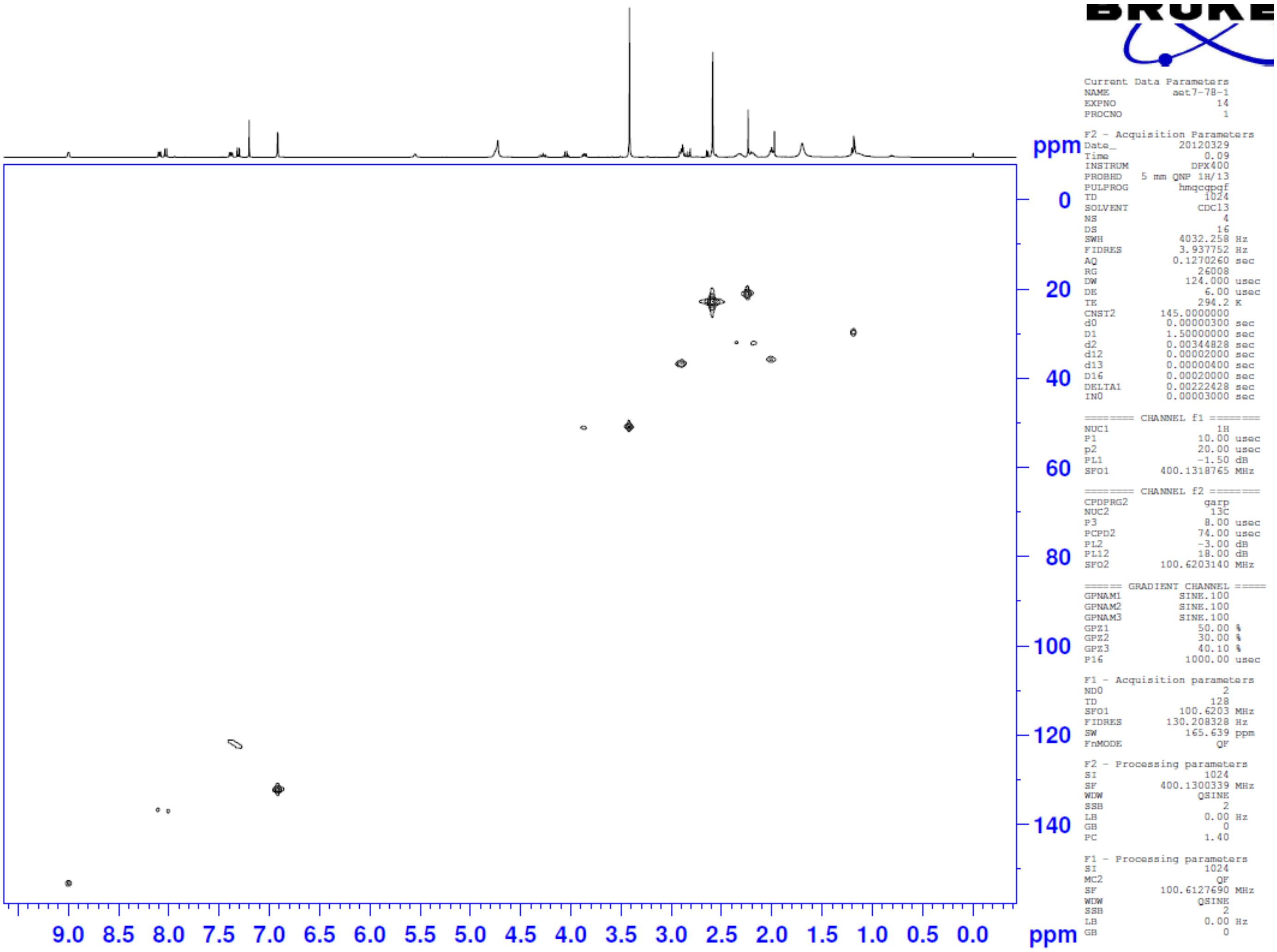
Current Data Parameters
NAME aet7-78-1
EXPNO 14
PROCNO 1
F2 - Acquisition Parameters
Date_ 20120329
Time 0.09
INSTRUM DPX400
PROBHD 5 mm QNP 1H/13
PULPROG hmqcgpqf
TD 1024
SOLVENT CDCl3
NS 4
DS 16
SWH 4032.258 Hz
FIDRES 3.937752 Hz
AQ 0.1270260 sec
RG 26008
DW 124.000 usec
DE 6.00 usec
TE 294.2 K
CNST2 145.0000000
d0 0.00000300 sec
D1 1.50000000 sec
d2 0.00344828 sec
d12 0.00002000 sec
d13 0.00000400 sec
D16 0.00020000 sec
DELTA1 0.00222428 sec
IN0 0.00003000 sec
======== CHANNEL f1 ========
NUC1 1H
P1 10.00 usec
p2 20.00 usec
PL1 -1.50 dB
SFO1 400.1318765 MHz
======== CHANNEL f2 ========
CPDPRG2 garp
NUC2 13C
P3 8.00 usec
PCPD2 74.00 usec
PL2 -3.00 dB
PL12 18.00 dB
SFO2 100.6203140 MHz
===== GRADIENT CHANNEL =====
GPNAM1 SINE.100
GPNAM2 SINE.100
GPNAM3 SINE.100
GPZ1 50.00 %
GPZ2 30.00 %
GPZ3 40.10 %
P16 1000.00 usec
F1 - Acquisition parameters
ND0 2
TD 128
SFO1 100.6203 MHz
FIDRES 130.208328 Hz
SW 165.639 ppm
FnMODE QF
F2 - Processing parameters
SI 1024
SF 400.1300339 MHz
WDW QSINE
SSB 2
LB 0.00 Hz
GB 0
PC 1.40
F1 - Processing parameters
SI 1024
MC2 QF
SF 100.6127690 MHz
WDW QSINE
SSB 2
LB 0.00 Hz
GB 0
ppm
0
20
40
60
80
100
120
140
9.0 8.5 8.0 7.5 7.0 6.5 6.0 5.5 5.0 4.5 4.0 3.5 3.0 2.5 2.0 1.5 1.0 0.5 0.0
ppm

Methyl N^2-(phenylsulfonyl)-N^4-((1*r,3*R**)-3-(2-(5,6,7,8-tetrahydro-1,8-naphthyridin-2-yl)ethyl)cyclobutyl)-*L*-asparaginate [Compound 32]**

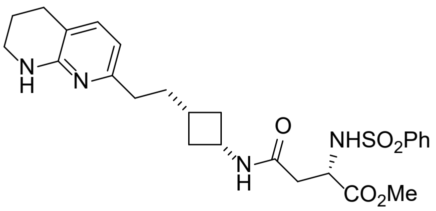

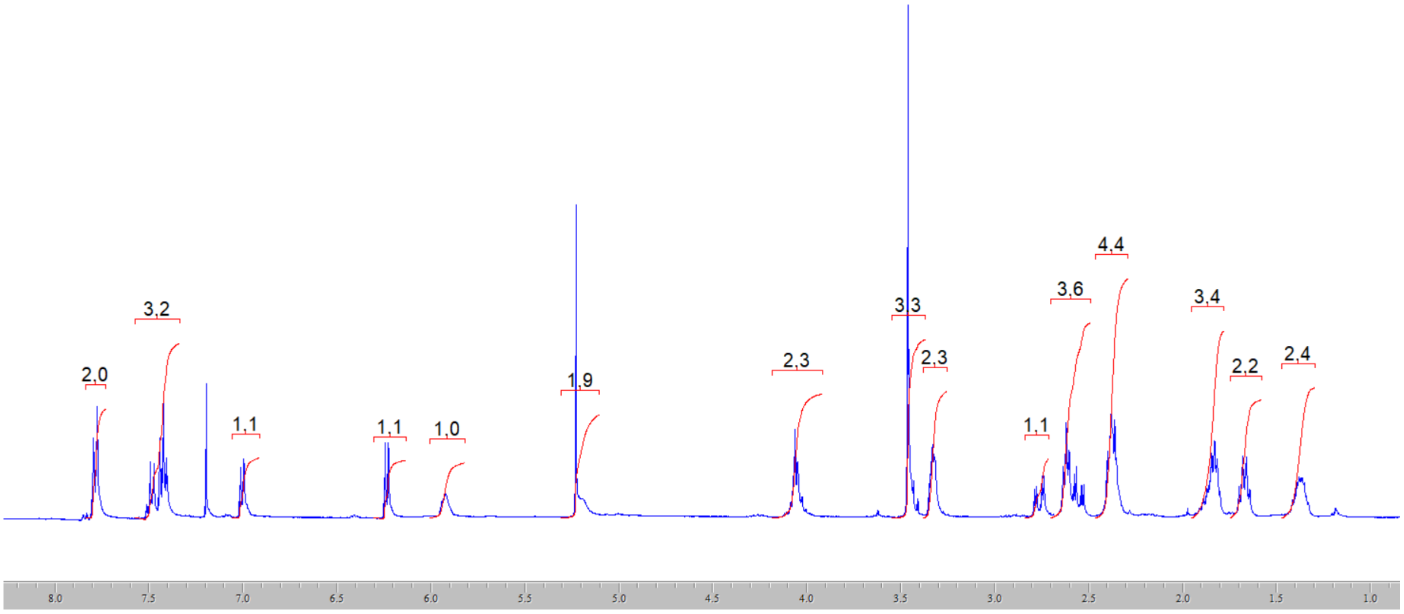

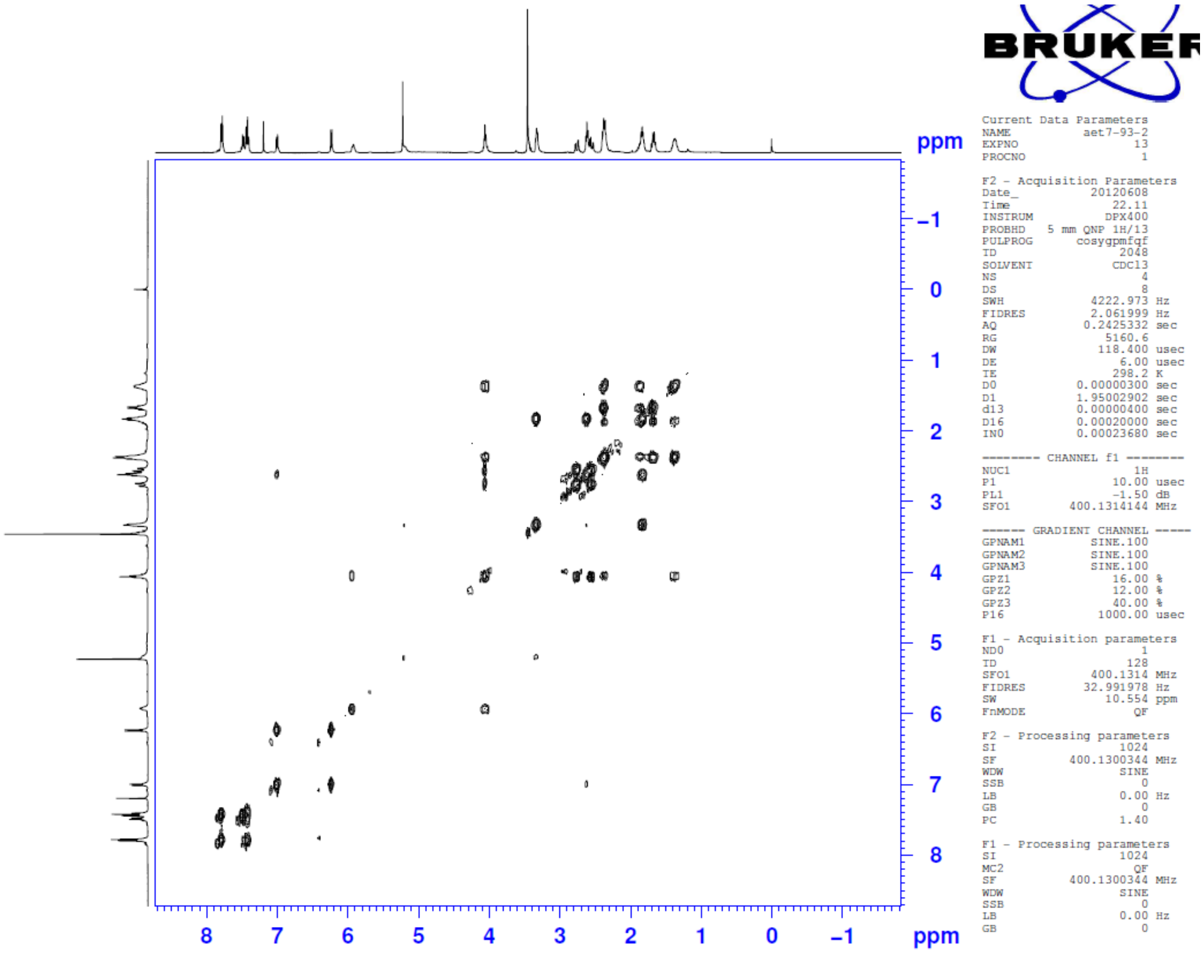

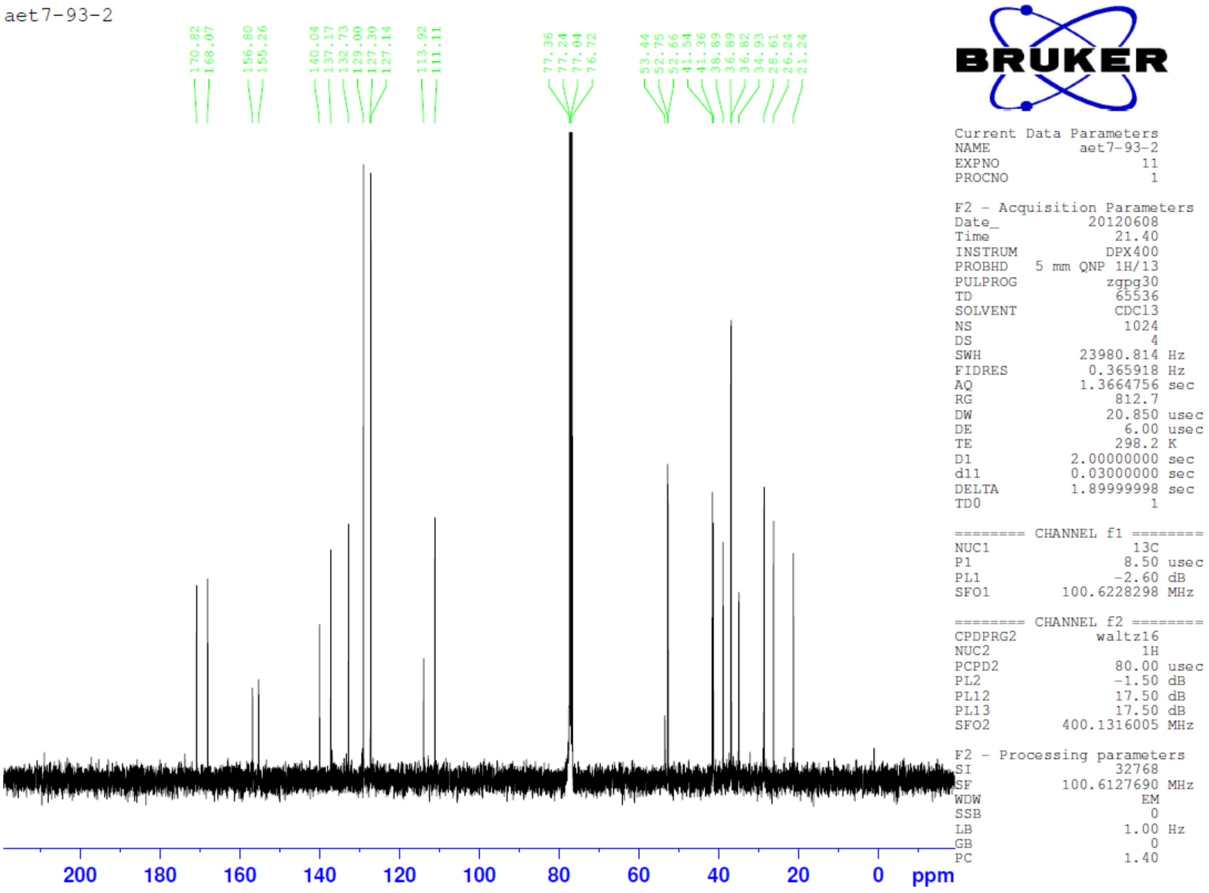
aet7-93-2
170.82 168.07 156.80 155.26 140.04 137.17 132.73 129.00 127.30 127.14 113.92 111.11 77.36 77.24 77.04 76.72 53.44 52.75 52.66 41.54 41.36 38.89 36.82 34.93 28.61 26.24 21.24
BRUKER
Current Data Parameters
NAME aet7-93-2
EXPNO 11
PROCNO 1
F2 - Acquisition Parameters
Date_ 20120608
Time 21.40
INSTRUM DPX400
PROBHD 5 mm QNP 1H/13
PULPROG zgpg30
TD 65536
SOLVENT CDCl3
NS 1024
DS 4
SWH 23980.814 Hz
FIDRES 0.365918 Hz
AQ 1.3664756 sec
RG 812.7
DW 20.850 usec
DE 6.00 usec
TE 298.2 K
D1 2.00000000 sec
d11 0.03000000 sec
DELTA 1.89999998 sec
TD0 1
======== CHANNEL f1 ========
NUC1 13C
P1 8.50 usec
PL1 -2.60 dB
SFO1 100.6228298 MHz
======== CHANNEL f2 ========
CPDPRG2 waltz16
NUC2 1H
PCPD2 80.00 usec
PL2 -1.50 dB
PL12 17.50 dB
PL13 17.50 dB
SFO2 400.1316005 MHz
F2 - Processing parameters
SI 32768
SF 100.6127690 MHz
WDW EM
SSB 0
LB 1.00 Hz
GB 0
PC 1.40
200 180 160 140 120 100 80 60 40 20 0 ppm

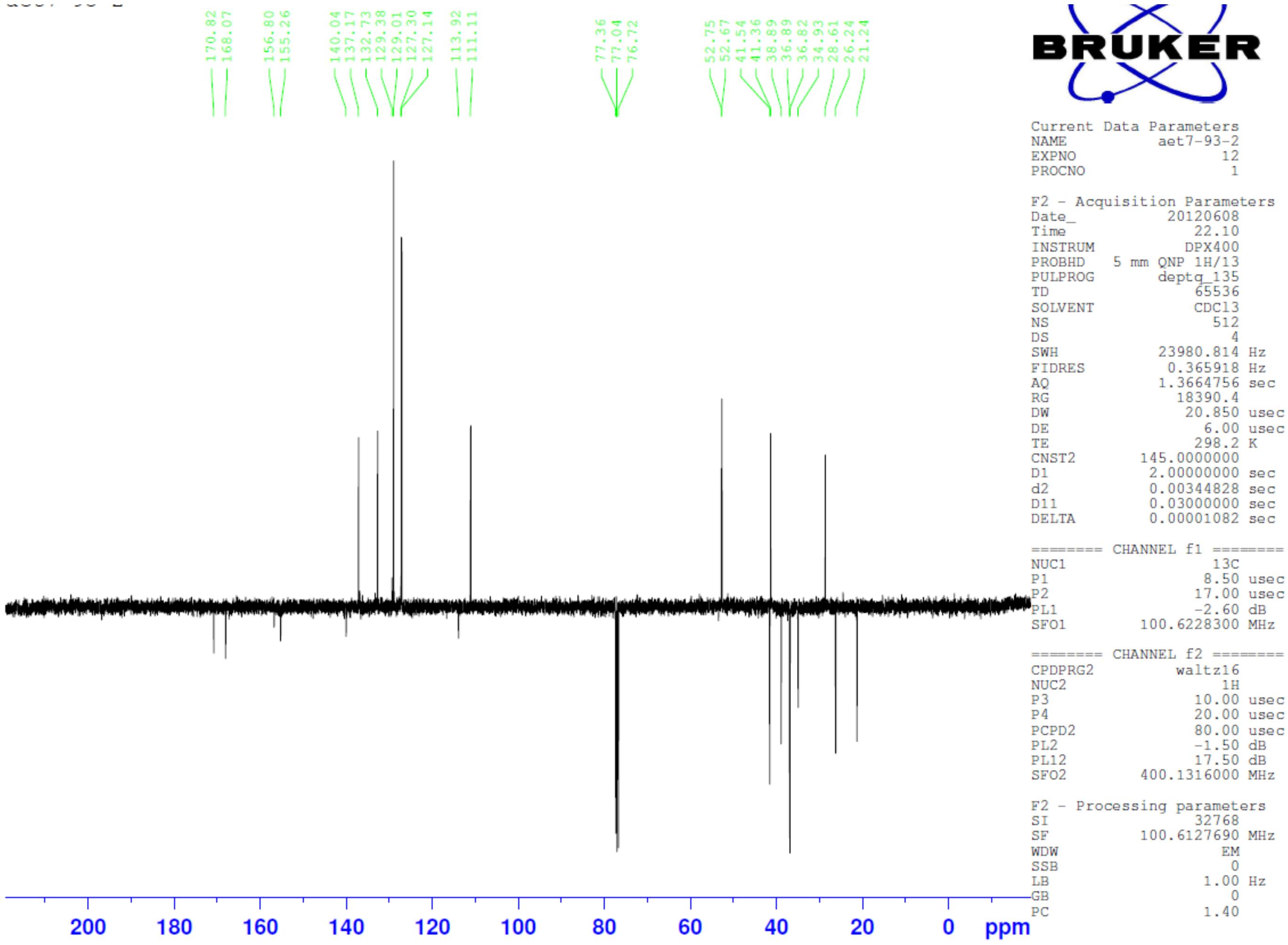
170.82 168.07 156.80 155.26 140.04 137.17 132.73 129.38 129.01 127.30 127.14 113.92 111.11 77.36 77.04 76.72 52.75 52.67 41.54 41.36 38.89 36.82 34.93 28.61 26.24 21.24
BRUKER
Current Data Parameters
NAME aet7-93-2
EXPNO 12
PROCNO 1
F2 - Acquisition Parameters
Date_ 20120608
Time 22.10
INSTRUM DPX400
PROBHD 5 mm QNP 1H/13
PULPROG deptq_135
TD 65536
SOLVENT CDCl3
NS 512
DS 4
SWH 23980.814 Hz
FIDRES 0.365918 Hz
AQ 1.3664756 sec
RG 18390.4
DW 20.850 usec
DE 6.00 usec
TE 298.2 K
CNST2 145.0000000
D1 2.00000000 sec
d2 0.00344828 sec
D11 0.03000000 sec
DELTA 0.00001082 sec
======== CHANNEL f1 ========
NUC1 13C
P1 8.50 usec
P2 17.00 usec
PL1 -2.60 dB
SFO1 100.6228300 MHz
======== CHANNEL f2 ========
CPDPRG2 waltz16
NUC2 1H
P3 10.00 usec
P4 20.00 usec
PCPD2 80.00 usec
PL2 -1.50 dB
PL12 17.50 dB
SFO2 400.1316000 MHz
F2 - Processing parameters
SI 32768
SF 100.6127690 MHz
WDW EM
SSB 0
LB 1.00 Hz
GB 0
PC 1.40
200 180 160 140 120 100 80 60 40 20 0 ppm

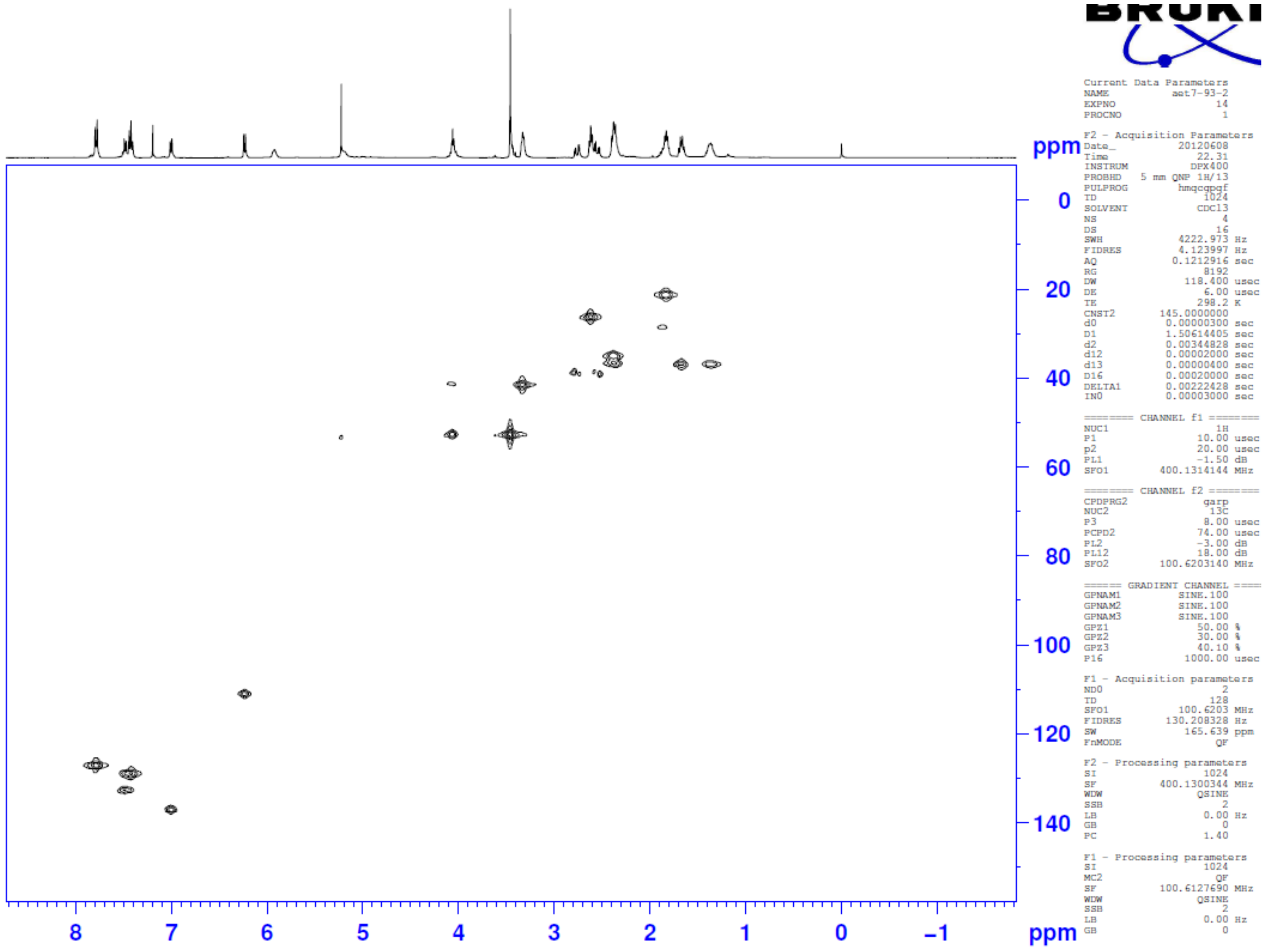
ppm
0
20
40
60
80
100
120
140
8
7
6
5
4
3
2
1
0
–1
ppm
Current Data Parameters
NAME aet7-93-2
EXPNO 14
PROCNO 1
F2 - Acquisition Parameters
Date_ 20120608
Time 22.31
INSTRUM DPX400
PROBHD 5 mm QNP 1H/13
PULPROG hmqcgpqf
TD 1024
SOLVENT CDCl3
NS 4
DS 16
SWH 4222.973 Hz
FIDRES 4.123997 Hz
AQ 0.1212916 sec
RG 8192
DW 118.400 usec
DE 6.00 usec
TE 298.2 K
CNST2 145.0000000
d0 0.00000300 sec
D1 1.50614405 sec
d2 0.00344828 sec
d12 0.00002000 sec
d13 0.00000400 sec
D16 0.00020000 sec
DELTA1 0.00222428 sec
IN0 0.00003000 sec
======= CHANNEL f1 =======
NUC1 1H
P1 10.00 usec
p2 20.00 usec
PL1 -1.50 dB
SFO1 400.1314144 MHz
======= CHANNEL f2 =======
CPDPRG2 garp
NUC2 13C
P3 8.00 usec
PCPD2 74.00 usec
PL2 -3.00 dB
PL12 18.00 dB
SFO2 100.6203140 MHz
===== GRADIENT CHANNEL ====
GPNAM1 SINE.100
GPNAM2 SINE.100
GPNAM3 SINE.100
GPZ1 50.00 %
GPZ2 30.00 %
GPZ3 40.10 %
P16 1000.00 usec
F1 - Acquisition parameters
ND0 2
TD 128
SFO1 100.6203 MHz
FIDRES 130.208328 Hz
SW 165.639 ppm
FnMODE QF
F2 - Processing parameters
SI 1024
SF 400.1300344 MHz
WDW QSINE
SSB 2
LB 0.00 Hz
GB 0
PC 1.40
F1 - Processing parameters
SI 1024
MC2 QF
SF 100.6127690 MHz
WDW QSINE
SSB 2
LB 0.00 Hz
GB 0

Methyl N^2-(mesitylsulfonyl)-N^4-((1*r,3*R**)-3-(2-(5,6,7,8-tetrahydro-1,8-naphthyridin-2-yl)ethyl)cyclobutyl)-*L*-asparaginate [Compound 33]**

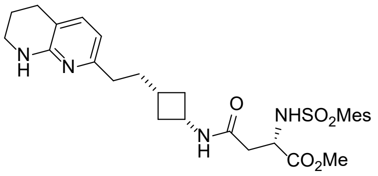

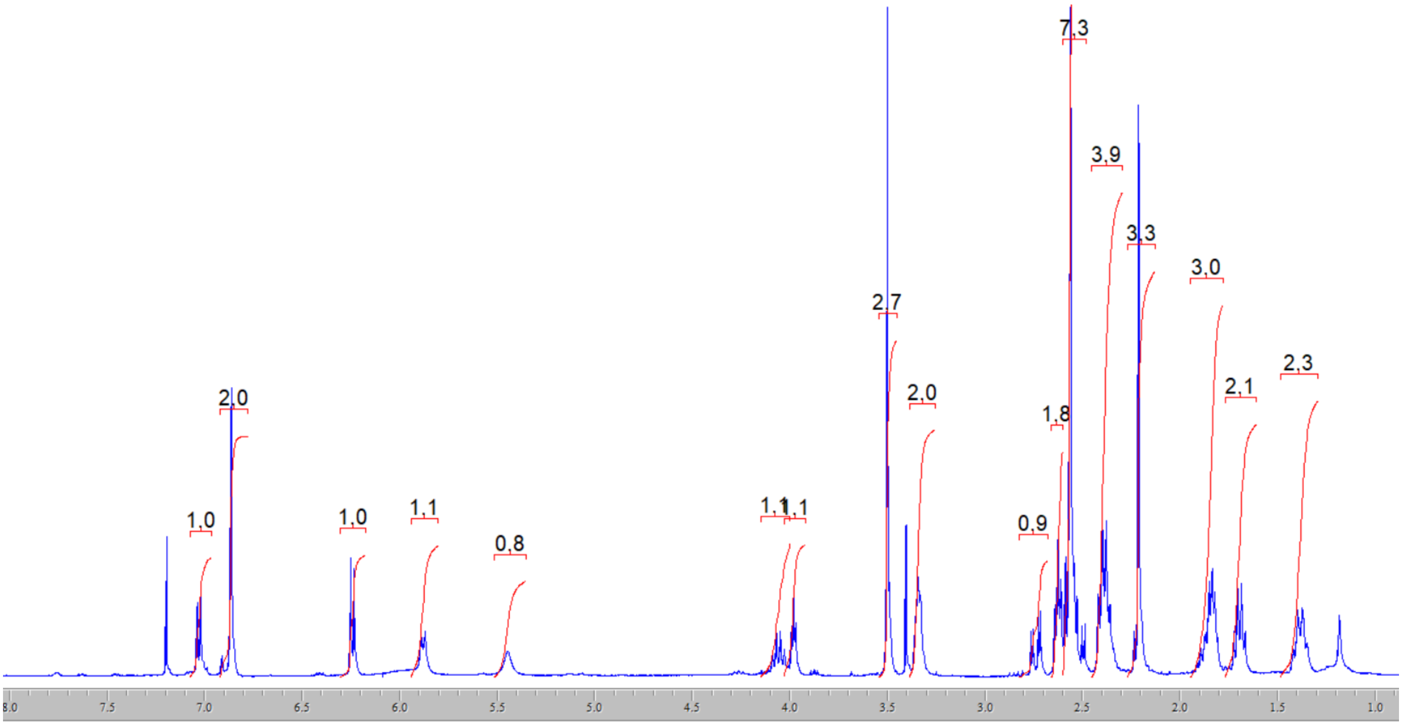

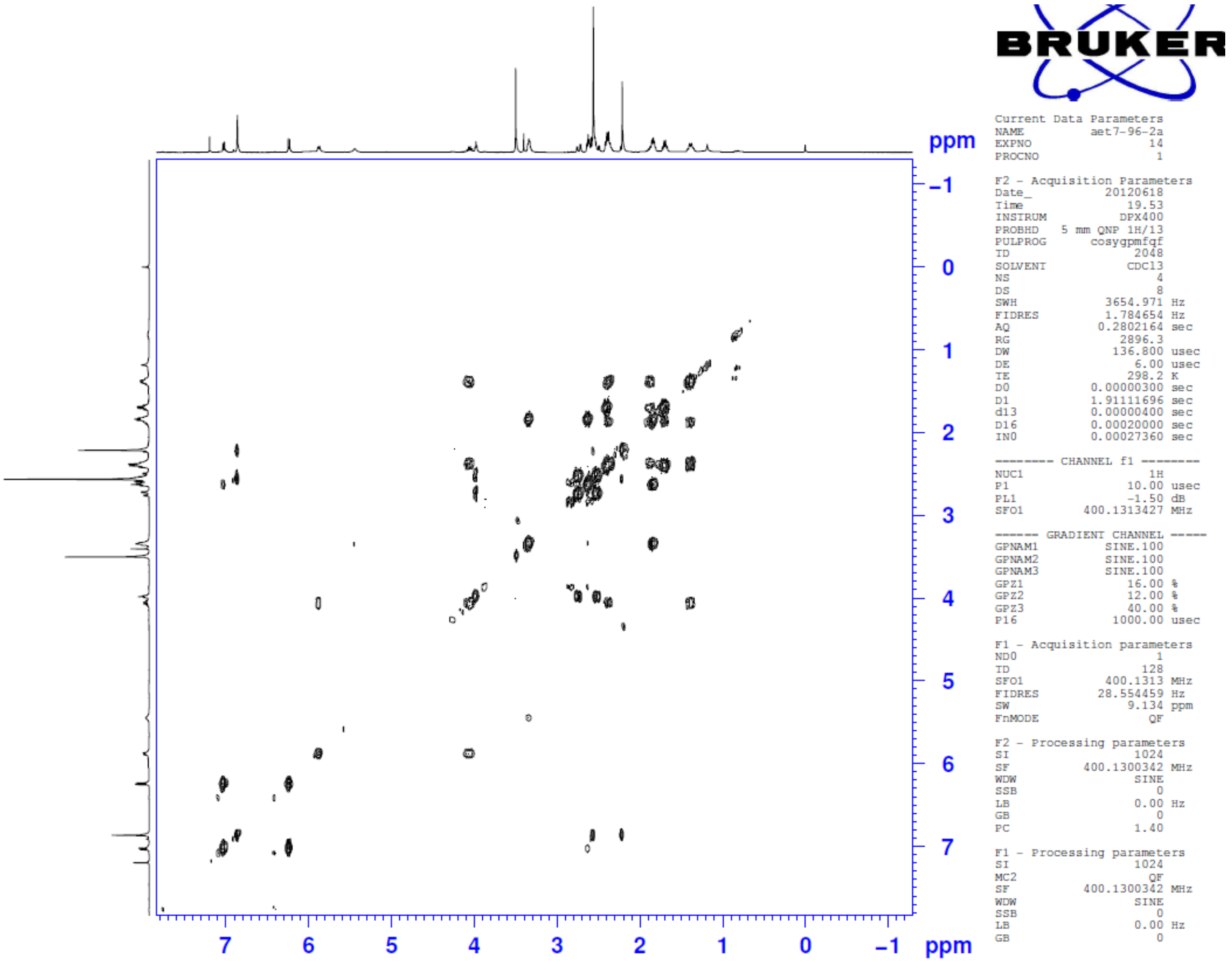

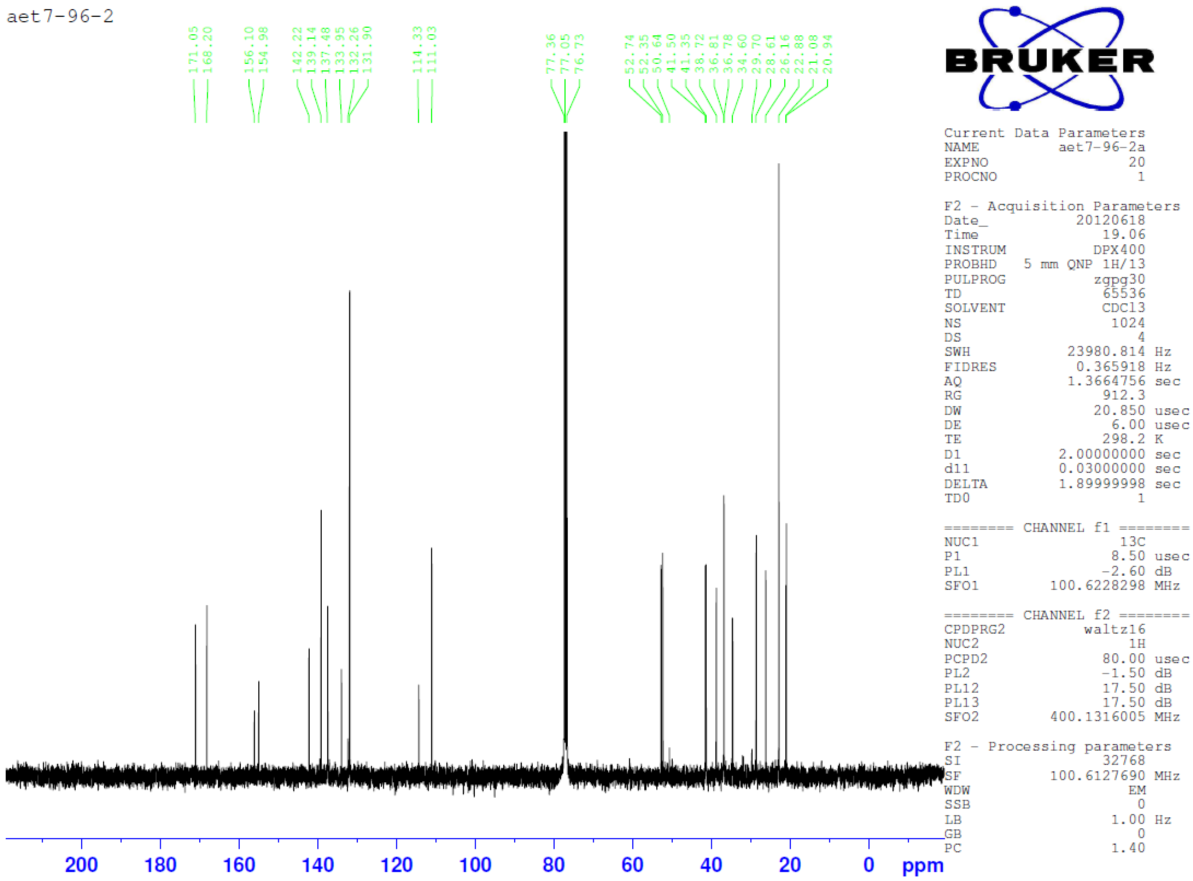
aet7-96-2
171.05
168.20
156.10
154.98
142.22
139.14
137.48
133.95
132.26
131.90
114.33
111.03
77.36
77.05
76.73
52.74
52.35
50.64
41.50
41.35
38.72
36.81
36.78
34.60
29.70
28.61
26.16
22.88
21.08
20.94
BRUKER
Current Data Parameters
NAME aet7-96-2a
EXPNO 20
PROCNO 1
F2 - Acquisition Parameters
Date_ 20120618
Time 19.06
INSTRUM DPX400
PROBHD 5 mm QNP 1H/13
PULPROG zgpg30
TD 65536
SOLVENT CDCl3
NS 1024
DS 4
SWH 23980.814 Hz
FIDRES 0.365918 Hz
AQ 1.3664756 sec
RG 912.3
DW 20.850 usec
DE 6.00 usec
TE 298.2 K
D1 2.00000000 sec
d11 0.03000000 sec
DELTA 1.89999998 sec
TD0 1
======== CHANNEL f1 ========
NUC1 13C
P1 8.50 usec
PL1 -2.60 dB
SFO1 100.6228298 MHz
======== CHANNEL f2 ========
CPDPRG2 waltz16
NUC2 1H
PCPD2 80.00 usec
PL2 -1.50 dB
PL12 17.50 dB
PL13 17.50 dB
SFO2 400.1316005 MHz
F2 - Processing parameters
SI 32768
SF 100.6127690 MHz
WDW EM
SSB 0
LB 1.00 Hz
GB 0
PC 1.40
200
180
160
140
120
100
80
60
40
20
0
ppm

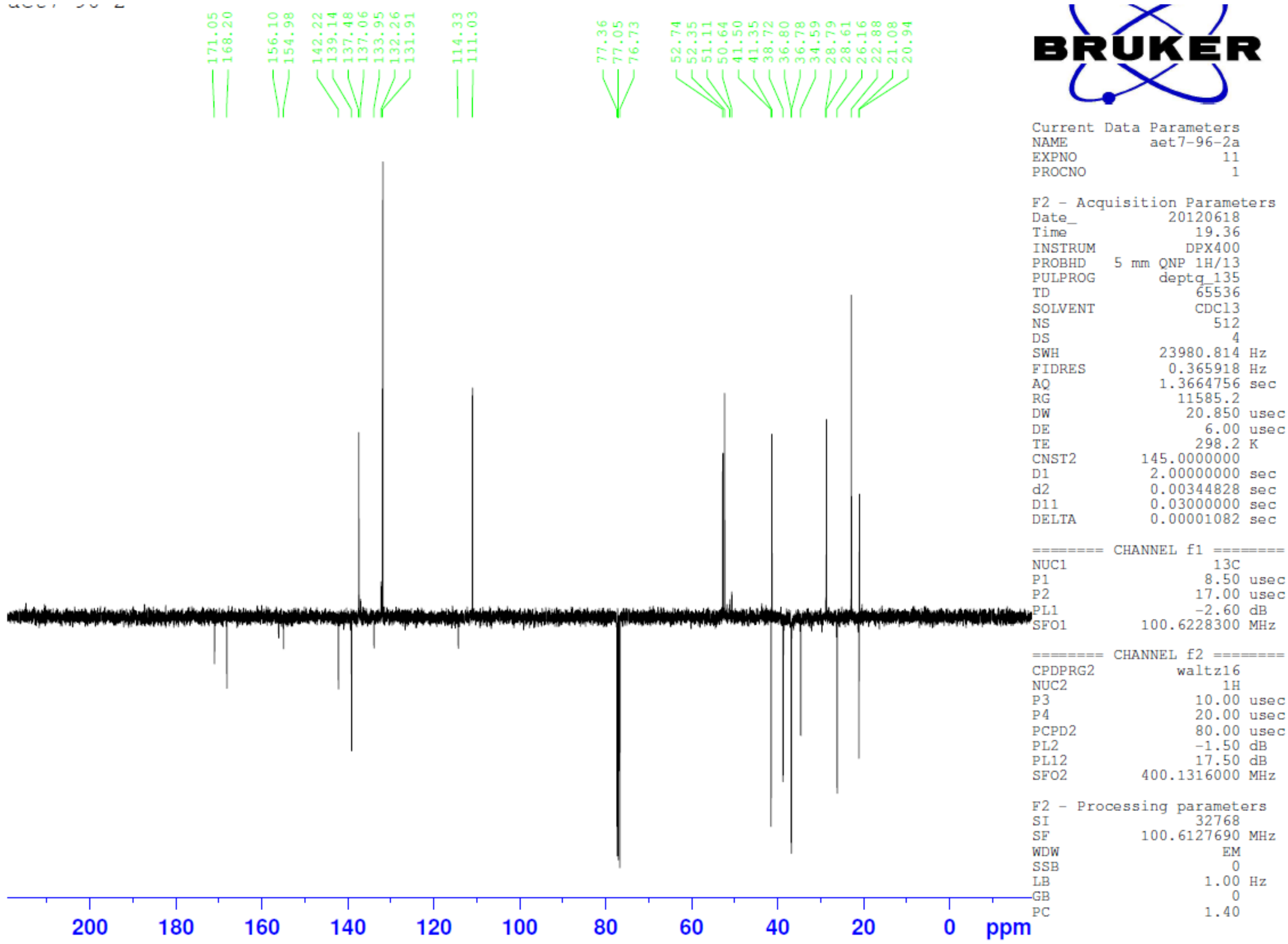
171.05
168.20
156.10
154.98
142.22
139.14
137.48
137.06
133.95
132.26
131.90
114.33
111.03
77.36
77.05
76.73
52.74
52.35
51.11
50.64
41.50
41.35
38.72
36.80
36.78
34.59
28.79
28.61
26.16
22.88
21.08
20.94
BRUKER
Current Data Parameters
NAME aet7-96-2a
EXPNO 11
PROCNO 1
F2 - Acquisition Parameters
Date_ 20120618
Time 19.36
INSTRUM DPX400
PROBHD 5 mm QNP 1H/13
PULPROG deptq_135
TD 65536
SOLVENT CDCl3
NS 512
DS 4
SWH 23980.814 Hz
FIDRES 0.365918 Hz
AQ 1.3664756 sec
RG 11585.2
DW 20.850 usec
DE 6.00 usec
TE 298.2 K
CNST2 145.0000000
D1 2.00000000 sec
d2 0.00344828 sec
D11 0.03000000 sec
DELTA 0.00001082 sec
======== CHANNEL f1 ========
NUC1 13C
P1 8.50 usec
P2 17.00 usec
PL1 -2.60 dB
SFO1 100.6228300 MHz
======== CHANNEL f2 ========
CPDPRG2 waltz16
NUC2 1H
P3 10.00 usec
P4 20.00 usec
PCPD2 80.00 usec
PL2 -1.50 dB
PL12 17.50 dB
SFO2 400.1316000 MHz
F2 - Processing parameters
SI 32768
SF 100.6127690 MHz
WDW EM
SSB 0
LB 1.00 Hz
GB 0
PC 1.40
200
180
160
140
120
100
80
60
40
20
0
ppm

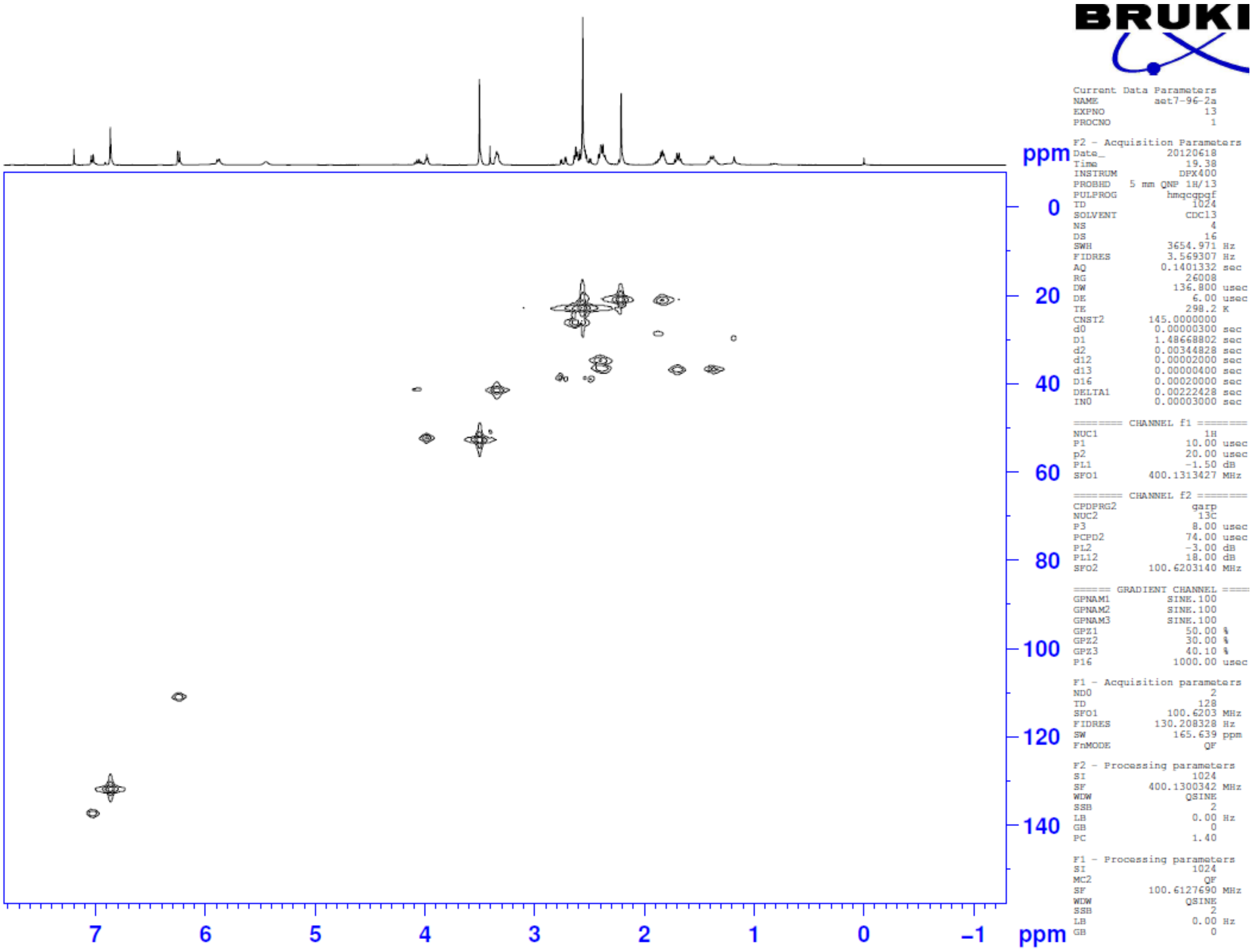

BRUKI
Current Data Parameters
NAME aet7-96-2a
EXPNO 13
PROCNO 1
F2 - Acquisition Parameters
Date_ 20120618
Time 19.38
INSTRUM DPX400
PROBHD 5 mm QNP 1H/13
PULPROG hmqcgpqf
TD 1024
SOLVENT CDCl3
NS 4
DS 16
SWH 3654.971 Hz
FIDRES 3.569307 Hz
AQ 0.1401332 sec
RG 26008
DW 136.800 usec
DE 6.00 usec
TE 298.2 K
CNST2 145.0000000
d0 0.00000300 sec
D1 1.48668802 sec
d2 0.00344828 sec
d12 0.00002000 sec
d13 0.00000400 sec
D16 0.00020000 sec
DELTA1 0.00222428 sec
IN0 0.00003000 sec
======= CHANNEL f1 =======
NUC1 1H
P1 10.00 usec
p2 20.00 usec
PL1 -1.50 dB
SFO1 400.1313427 MHz
======= CHANNEL f2 =======
CPDPRG2 garp
NUC2 13C
P3 8.00 usec
PCPD2 74.00 usec
PL2 -3.00 dB
PL12 18.00 dB
SFO2 100.6203140 MHz
===== GRADIENT CHANNEL =====
GPNAM1 SINE.100
GPNAM2 SINE.100
GPNAM3 SINE.100
GPZ1 50.00 %
GPZ2 30.00 %
GPZ3 40.10 %
P16 1000.00 usec
F1 - Acquisition parameters
ND0 2
TD 128
SFO1 100.6203 MHz
FIDRES 130.208328 Hz
SW 165.639 ppm
FnMODE QF
F2 - Processing parameters
SI 1024
SF 400.1300342 MHz
WDW QSINE
SSB 2
LB 0.00 Hz
GB 0
PC 1.40
F1 - Processing parameters
SI 1024
MC2 QF
SF 100.6127690 MHz
WDW QSINE
SSB 2
LB 0.00 Hz
GB 0
ppm
0
20
40
60
80
100
120
140
7
6
5
4
3
2
1
0
–1
ppm

N-((S)-2,5-dioxo-1-((1r*,3R*)-3-(2-(5,6,7,8-tetrahydro-1,8-naphthyridin-2-yl)ethyl)cyclobutyl)pyrrolidin-3-yl)benzenesulfonamide [Compound 34]

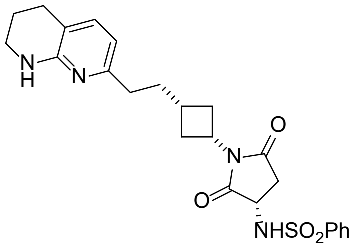

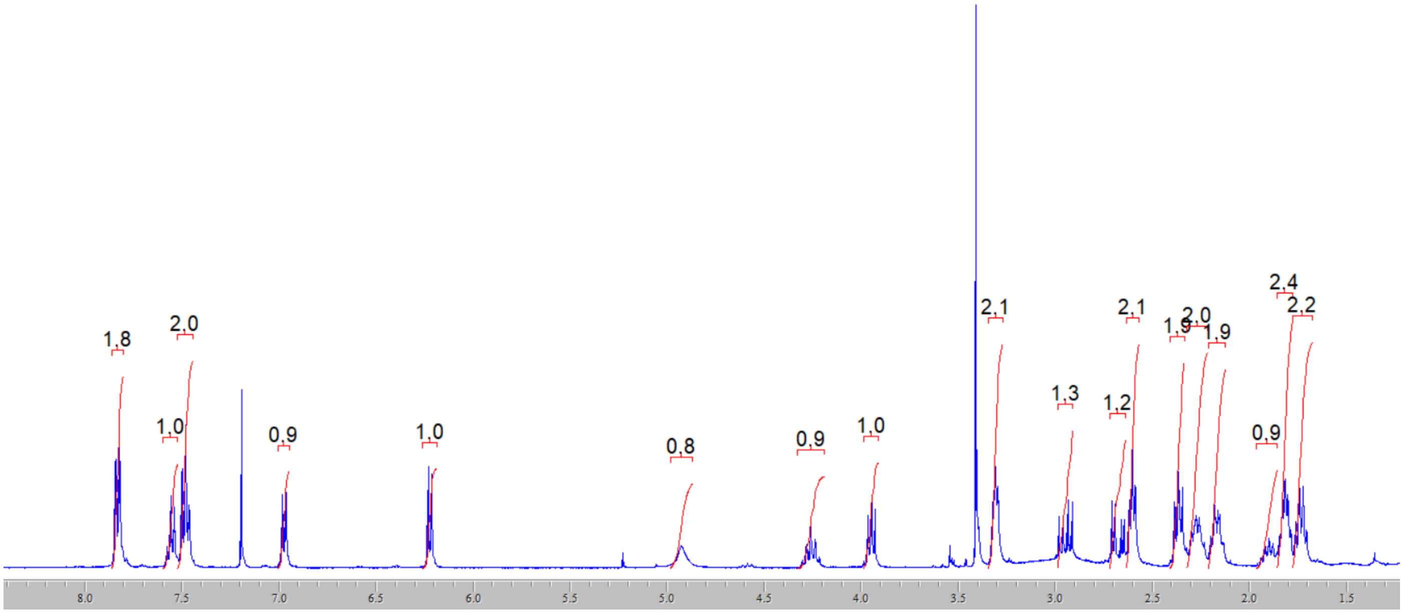

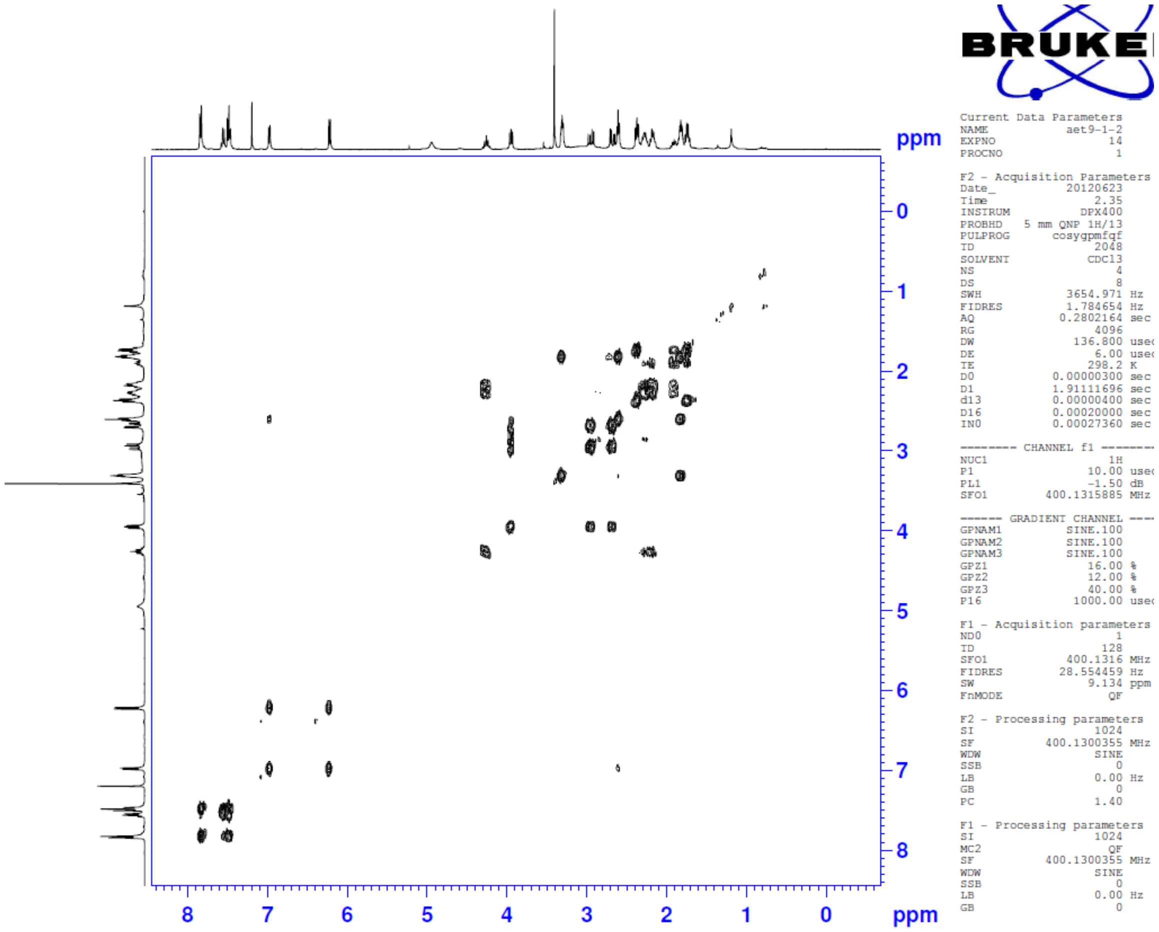

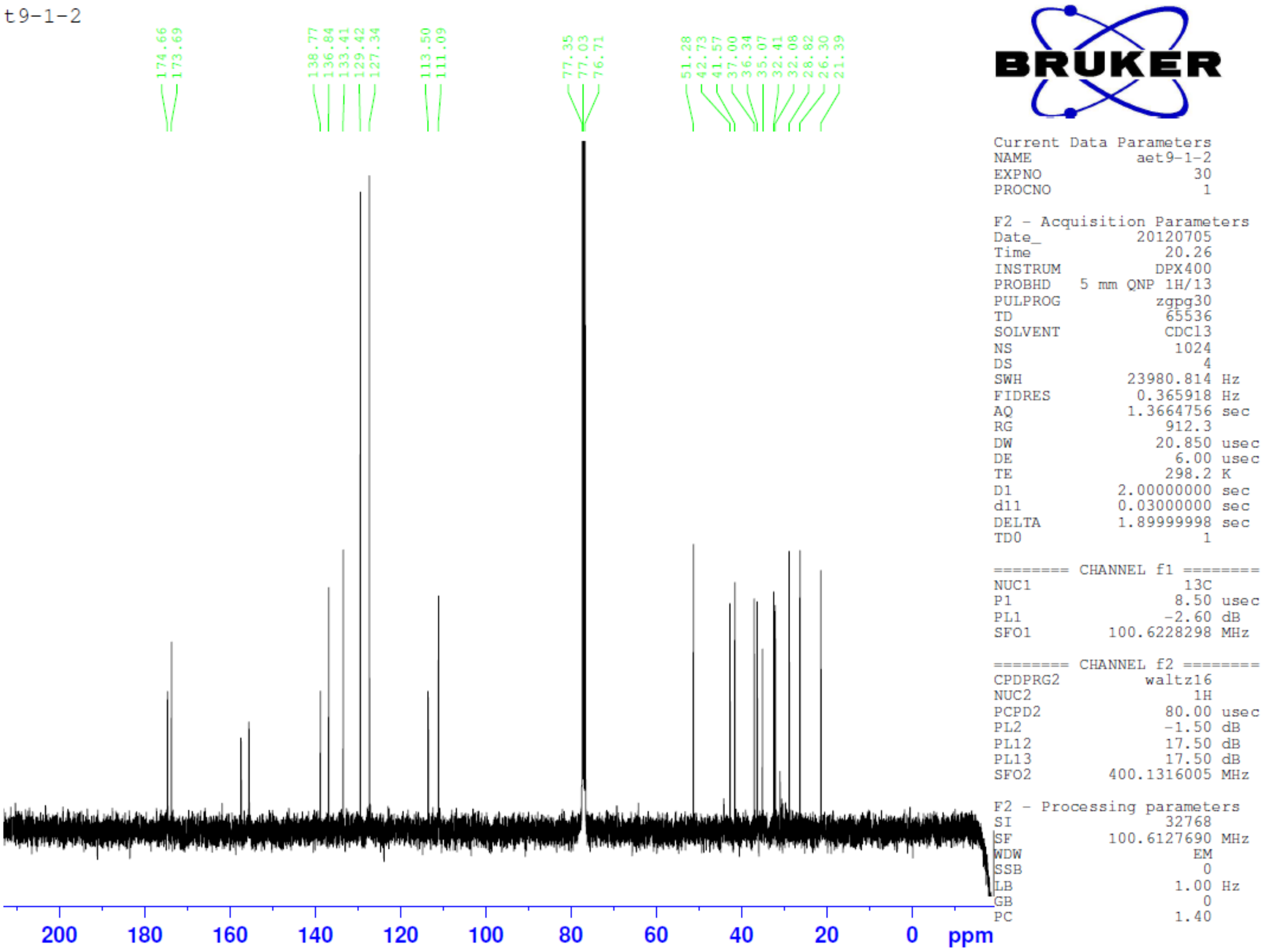
t9-1-2
174.66
173.69
138.77
136.84
133.41
129.42
127.34
113.50
111.09
77.35
77.03
76.71
51.28
42.73
41.57
37.00
36.34
35.07
32.41
32.08
28.82
26.30
21.39
200
180
160
140
120
100
80
60
40
20
0
ppm
Current Data Parameters
NAME aet9-1-2
EXPNO 30
PROCNO 1
F2 - Acquisition Parameters
Date_ 20120705
Time 20.26
INSTRUM DPX400
PROBHD 5 mm QNP 1H/13
PULPROG zgpg30
TD 65536
SOLVENT CDCl3
NS 1024
DS 4
SWH 23980.814 Hz
FIDRES 0.365918 Hz
AQ 1.3664756 sec
RG 912.3
DW 20.850 usec
DE 6.00 usec
TE 298.2 K
D1 2.00000000 sec
d11 0.03000000 sec
DELTA 1.89999998 sec
TD0 1
======== CHANNEL f1 ========
NUC1 13C
P1 8.50 usec
PL1 -2.60 dB
SFO1 100.6228298 MHz
======== CHANNEL f2 ========
CPDPRG2 waltz16
NUC2 1H
PCPD2 80.00 usec
PL2 -1.50 dB
PL12 17.50 dB
PL13 17.50 dB
SFO2 400.1316005 MHz
F2 - Processing parameters
SI 32768
SF 100.6127690 MHz
WDW EM
SSB 0
LB 1.00 Hz
GB 0
PC 1.40

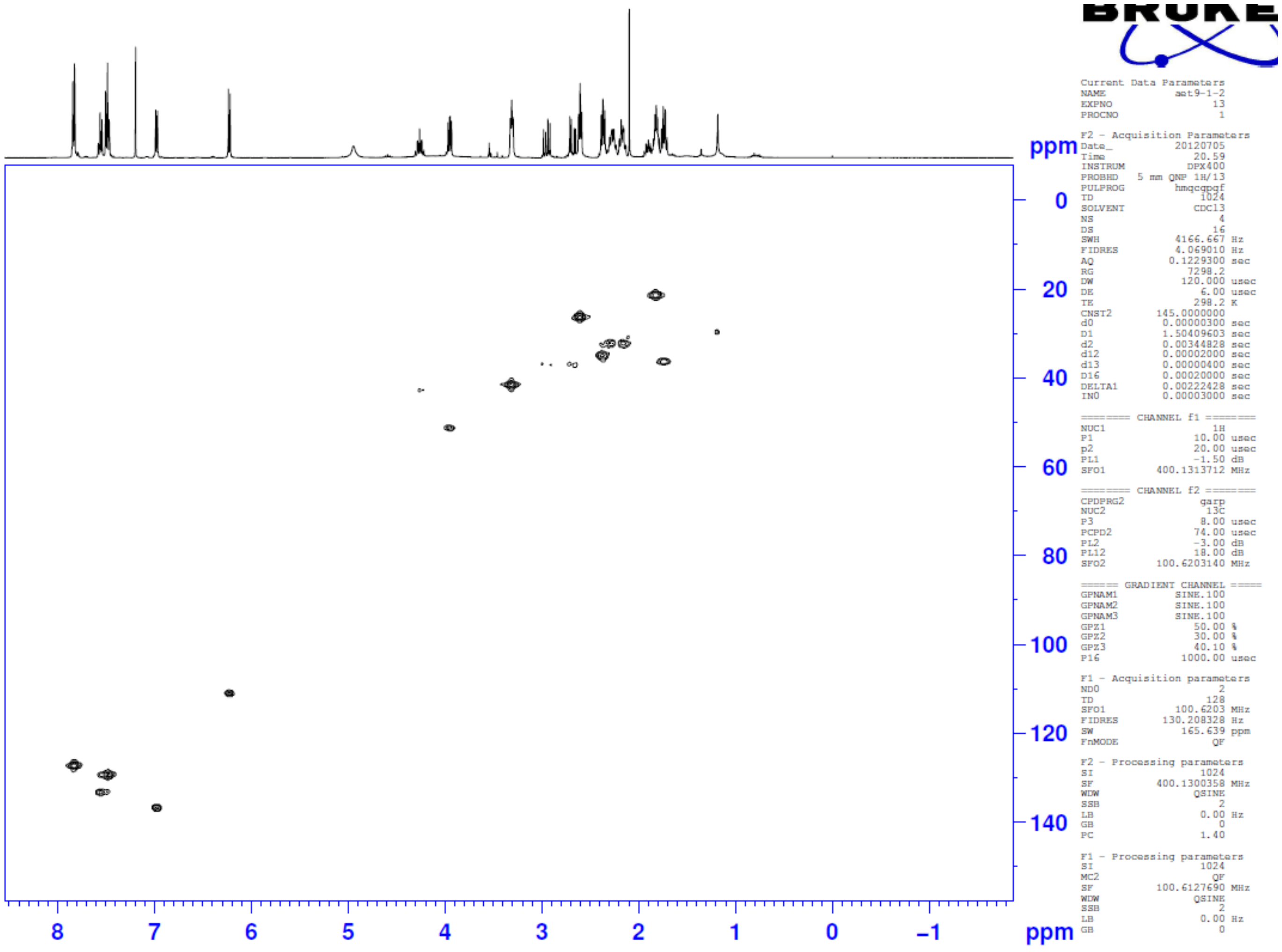
ppm
0
20
40
60
80
100
120
140
8
7
6
5
4
3
2
1
0
-1
ppm
Current Data Parameters
NAME aet9-1-2
EXPNO 13
PROCNO 1
F2 - Acquisition Parameters
Date_ 20120705
Time 20.59
INSTRUM DPX400
PROBHD 5 mm QNP 1H/13
PULPROG hmqcgpqf
TD 1024
SOLVENT CDCl3
NS 4
DS 16
SWH 4166.667 Hz
FIDRES 4.069010 Hz
AQ 0.1229300 sec
RG 7298.2
DW 120.000 usec
DE 6.00 usec
TE 298.2 K
CNST2 145.0000000
d0 0.00000300 sec
D1 1.50409603 sec
d2 0.00344828 sec
d12 0.00002000 sec
d13 0.00000400 sec
D16 0.00020000 sec
DELTA1 0.00222428 sec
IN0 0.00003000 sec
======== CHANNEL f1 ========
NUC1 1H
P1 10.00 usec
p2 20.00 usec
PL1 -1.50 dB
SFO1 400.1313712 MHz
======== CHANNEL f2 ========
CPDPRG2 garp
NUC2 13C
P3 8.00 usec
PCPD2 74.00 usec
PL2 -3.00 dB
PL12 18.00 dB
SFO2 100.6203140 MHz
===== GRADIENT CHANNEL =====
GPNAM1 SINE.100
GPNAM2 SINE.100
GPNAM3 SINE.100
GPZ1 50.00 %
GPZ2 30.00 %
GPZ3 40.10 %
P16 1000.00 usec
F1 - Acquisition parameters
ND0 2
TD 128
SFO1 100.6203 MHz
FIDRES 130.208328 Hz
SW 165.639 ppm
FnMODE QF
F2 - Processing parameters
SI 1024
SF 400.1300358 MHz
WDW QSINE
SSB 2
LB 0.00 Hz
GB 0
PC 1.40
F1 - Processing parameters
SI 1024
MC2 QF
SF 100.6127690 MHz
WDW QSINE
SSB 2
LB 0.00 Hz
GB 0

***N*-((*S*)-2,5-Dioxo-1-((1*r**,3*R**)-3-(2-(5,6,7,8-tetrahydro-1,8-naphthyridin-2-yl)ethyl)cyclobutyl)pyrrolidin-3-yl)-2,4,6-trimethylbenzenesulfonamide [Compound 35]**

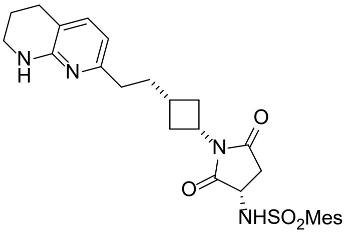

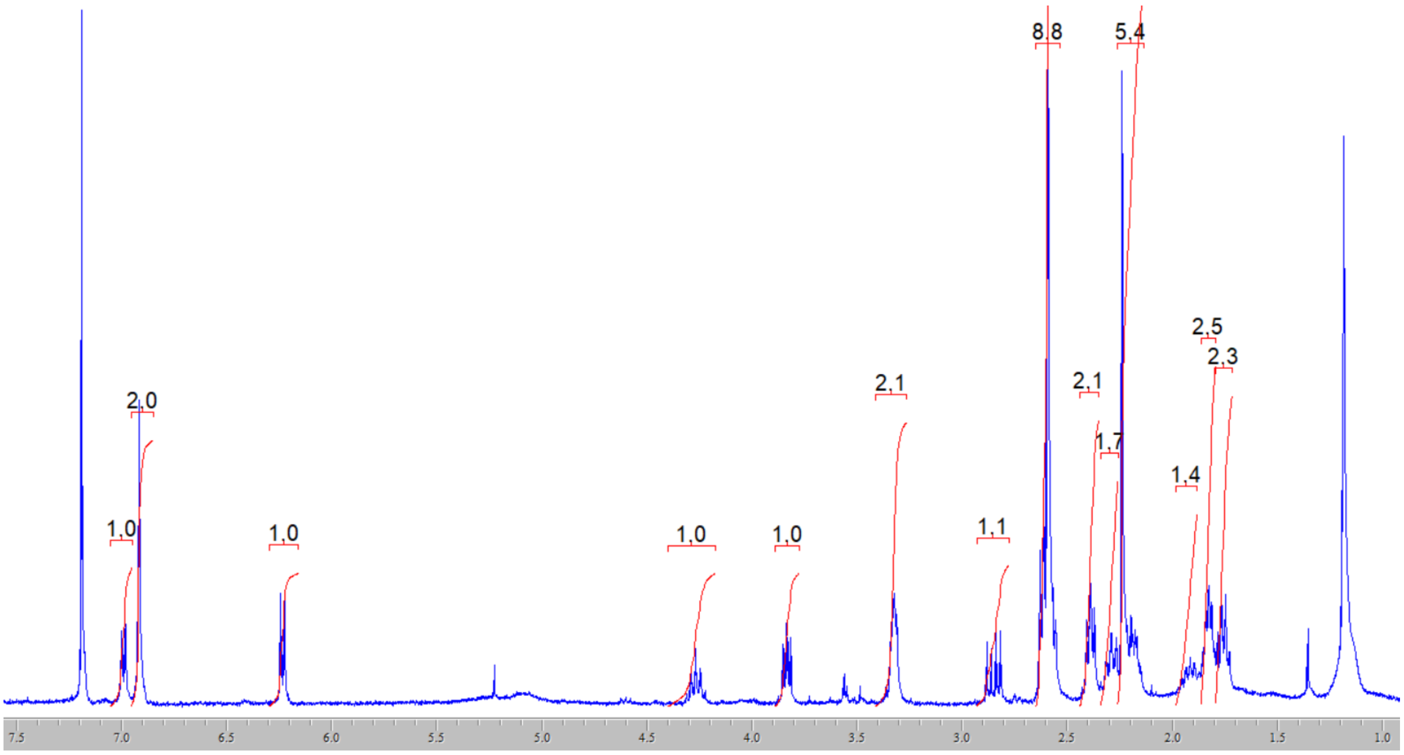

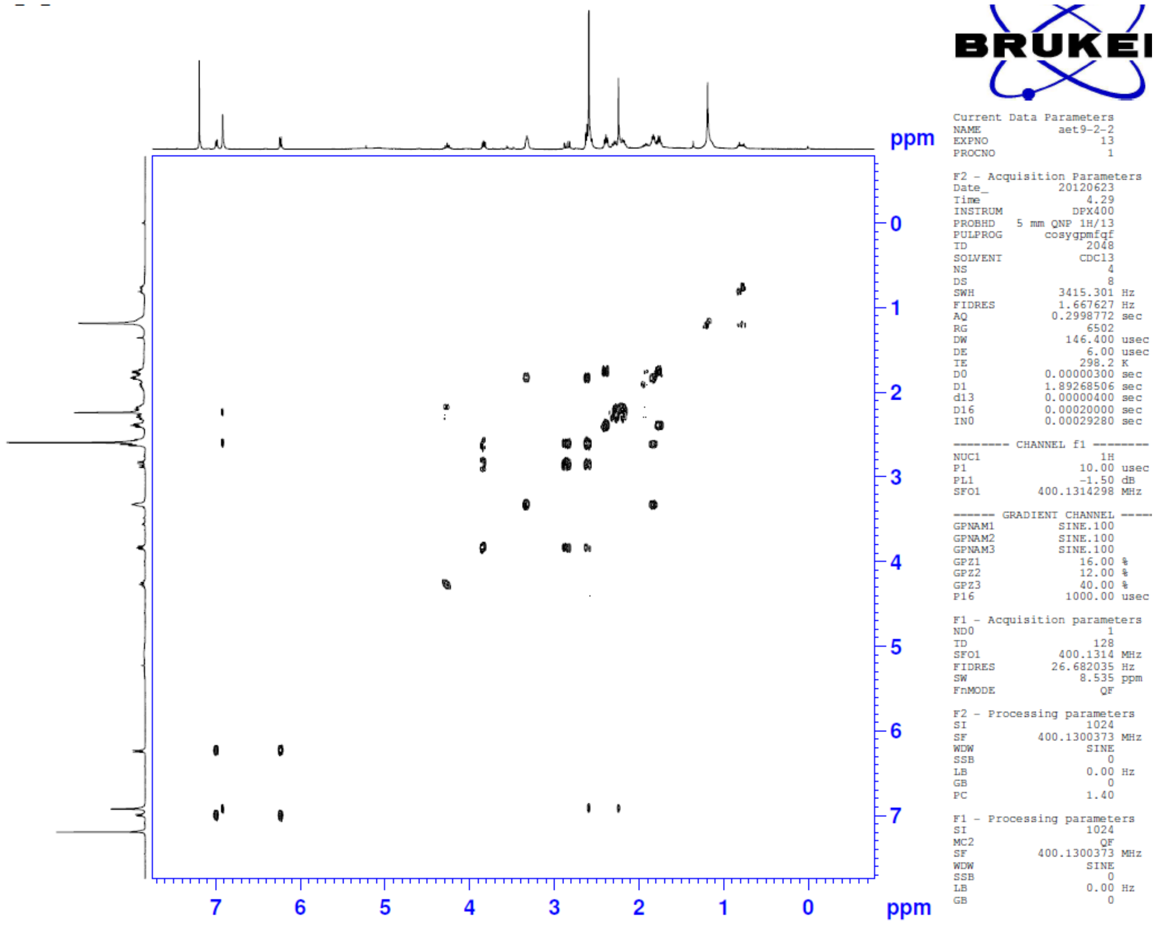

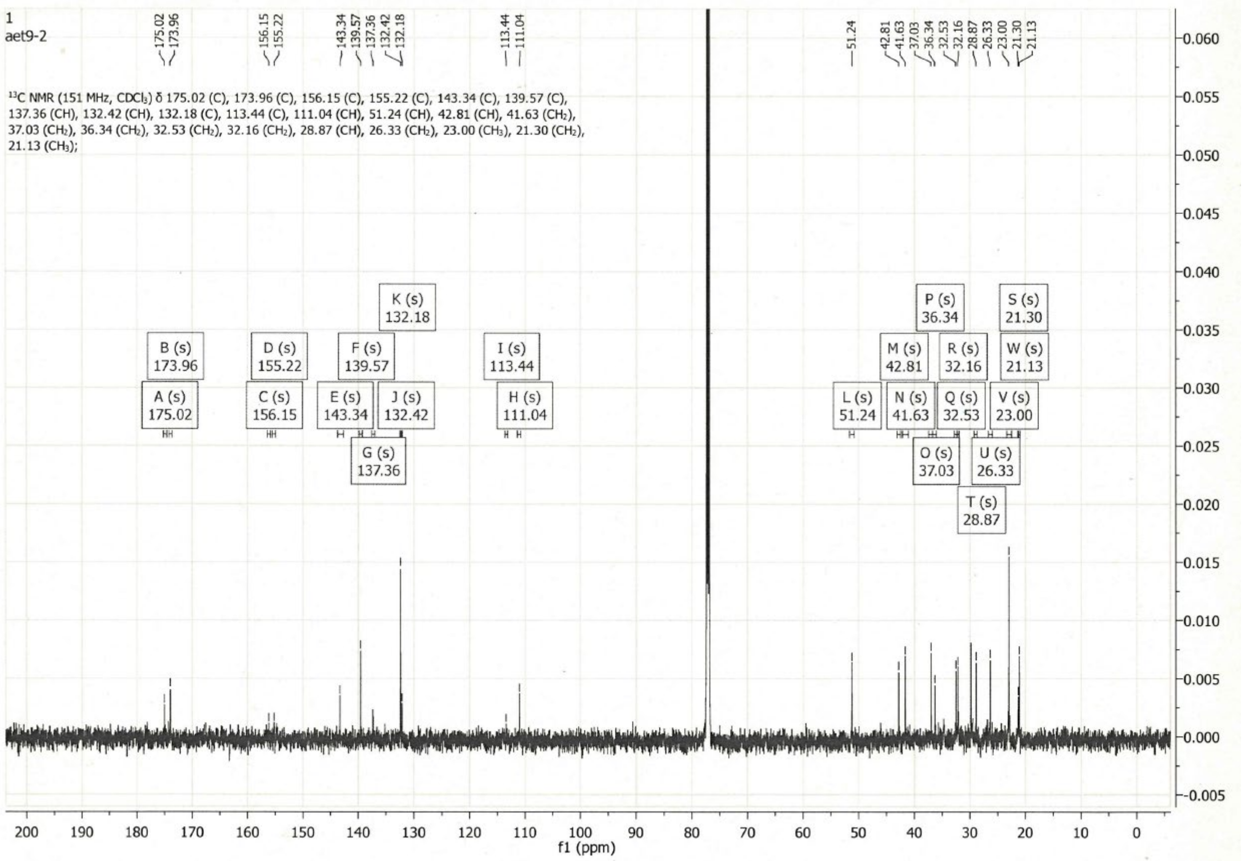

1
aet9-2
^{13}C NMR (151 MHz, CDCl$_3$) δ 175.02 (C), 173.96 (C), 156.15 (C), 155.22 (C), 143.34 (C), 139.57 (C), 137.36 (CH), 132.42 (CH), 132.18 (C), 113.44 (C), 111.04 (CH), 51.24 (CH), 42.81 (CH), 41.63 (CH$_2$), 37.03 (CH$_2$), 36.34 (CH$_2$), 32.53 (CH$_2$), 32.16 (CH$_2$), 28.87 (CH), 26.33 (CH$_2$), 23.00 (CH$_3$), 21.30 (CH$_2$), 21.13 (CH$_3$);
f1 (ppm)

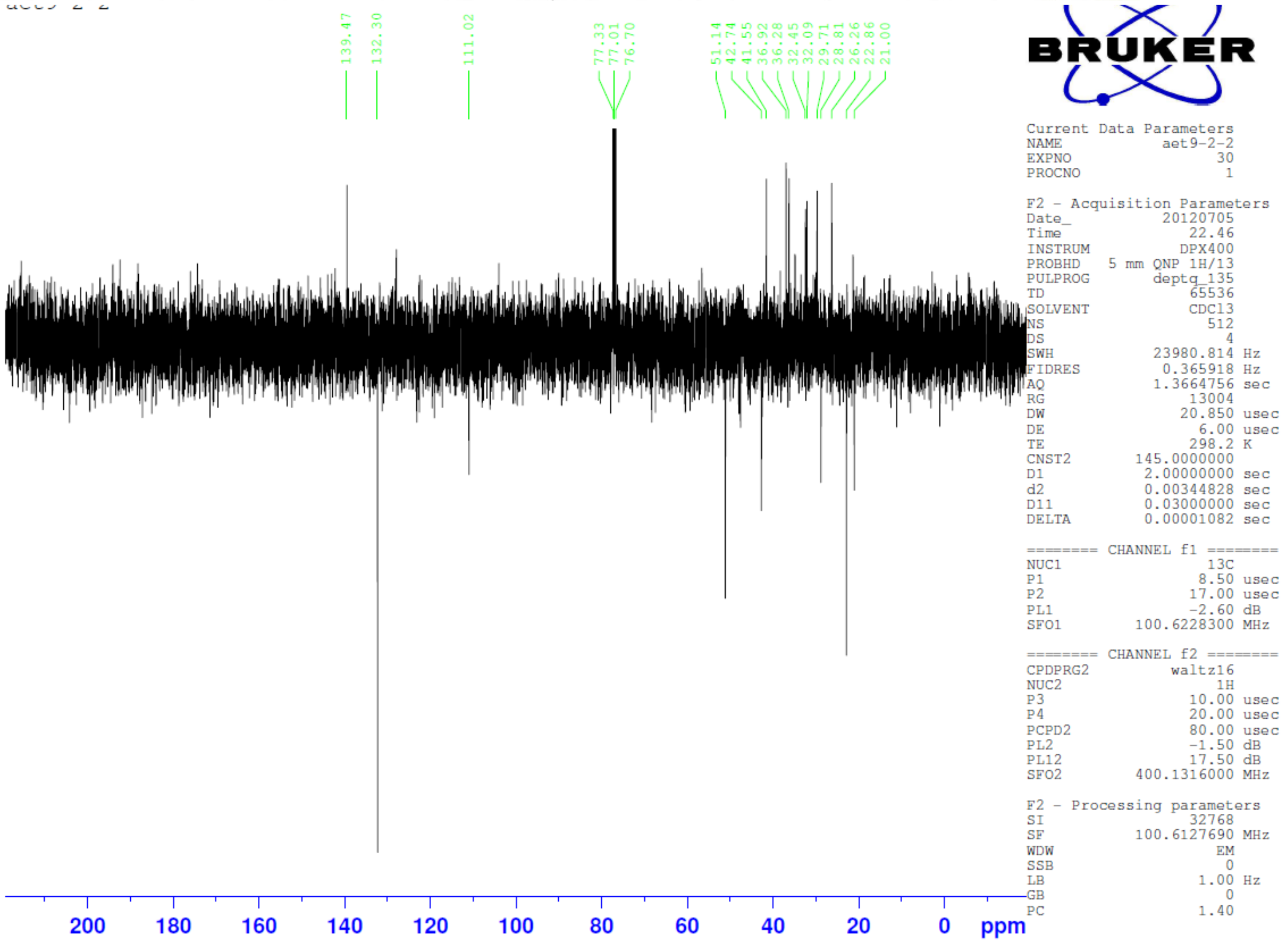

aet9-2-2
BRUKER
Current Data Parameters
NAME aet9-2-2
EXPNO 30
PROCNO 1
F2 - Acquisition Parameters
Date_ 20120705
Time 22.46
INSTRUM DPX400
PROBHD 5 mm QNP 1H/13
PULPROG deptq_135
TD 65536
SOLVENT CDCl3
NS 512
DS 4
SWH 23980.814 Hz
FIDRES 0.365918 Hz
AQ 1.3664756 sec
RG 13004
DW 20.850 usec
DE 6.00 usec
TE 298.2 K
CNST2 145.0000000
D1 2.00000000 sec
d2 0.00344828 sec
D11 0.03000000 sec
DELTA 0.00001082 sec
======== CHANNEL f1 ========
NUC1 13C
P1 8.50 usec
P2 17.00 usec
PL1 -2.60 dB
SFO1 100.6228300 MHz
======== CHANNEL f2 ========
CPDPRG2 waltz16
NUC2 1H
P3 10.00 usec
P4 20.00 usec
PCPD2 80.00 usec
PL2 -1.50 dB
PL12 17.50 dB
SFO2 400.1316000 MHz
F2 - Processing parameters
SI 32768
SF 100.6127690 MHz
WDW EM
SSB 0
LB 1.00 Hz
GB 0
PC 1.40
ppm

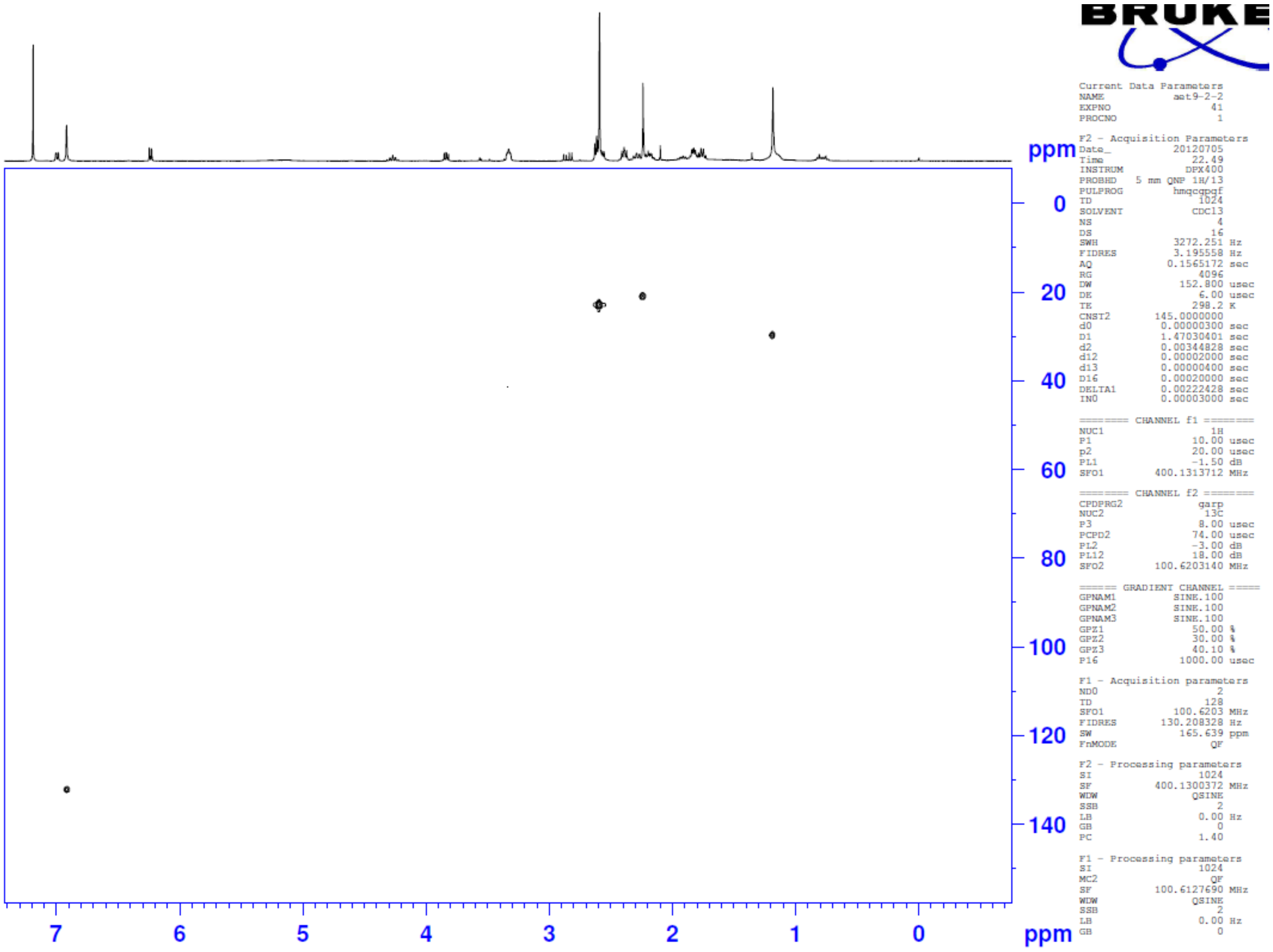
BRUKE
Current Data Parameters
NAME aet9-2-2
EXPNO 41
PROCNO 1
F2 - Acquisition Parameters
Date_ 20120705
Time 22.49
INSTRUM DPX400
PROBHD 5 mm QNP 1H/13
PULPROG hmqcgpqf
TD 1024
SOLVENT CDCl3
NS 4
DS 16
SWH 3272.251 Hz
FIDRES 3.195558 Hz
AQ 0.1565172 sec
RG 4096
DW 152.800 usec
DE 6.00 usec
TE 298.2 K
CNST2 145.0000000
d0 0.00000300 sec
D1 1.47030401 sec
d2 0.00344828 sec
d12 0.00002000 sec
d13 0.00000400 sec
D16 0.00020000 sec
DELTA1 0.00222428 sec
IN0 0.00003000 sec
======== CHANNEL f1 ========
NUC1 1H
P1 10.00 usec
p2 20.00 usec
PL1 -1.50 dB
SFO1 400.1313712 MHz
======== CHANNEL f2 ========
CPDPRG2 garp
NUC2 13C
P3 8.00 usec
PCPD2 74.00 usec
PL2 -3.00 dB
PL12 18.00 dB
SFO2 100.6203140 MHz
===== GRADIENT CHANNEL =====
GPNAM1 SINE.100
GPNAM2 SINE.100
GPNAM3 SINE.100
GPZ1 50.00 %
GPZ2 30.00 %
GPZ3 40.10 %
P16 1000.00 usec
F1 - Acquisition parameters
ND0 2
TD 128
SFO1 100.6203 MHz
FIDRES 130.208328 Hz
SW 165.639 ppm
FnMODE QF
F2 - Processing parameters
SI 1024
SF 400.1300372 MHz
WDW QSINE
SSB 2
LB 0.00 Hz
GB 0
PC 1.40
F1 - Processing parameters
SI 1024
MC2 QF
SF 100.6127690 MHz
WDW QSINE
SSB 2
LB 0.00 Hz
GB 0
ppm
0
20
40
60
80
100
120
140
7
6
5
4
3
2
1
0
ppm

(*S*)-4-(*tert*-butoxy)-4-oxo-2-(phenylsulfonamido)butanoic acid [Compound 36]

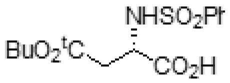

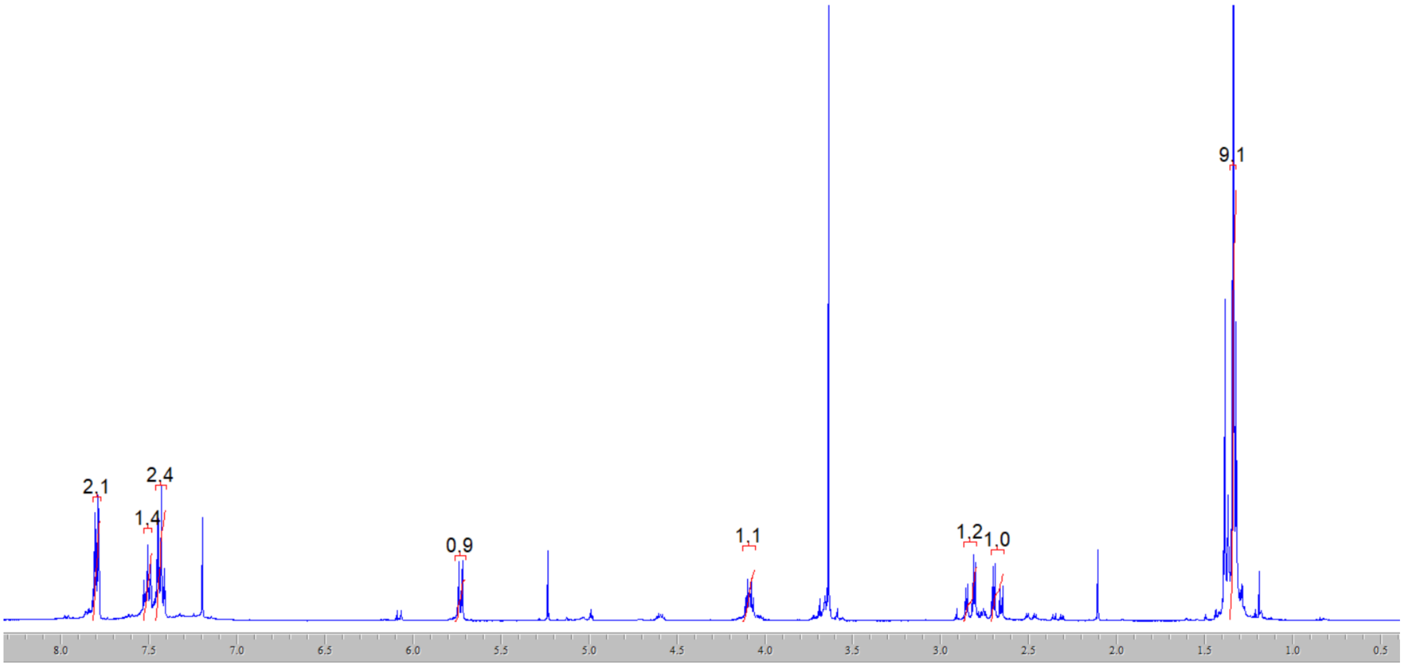

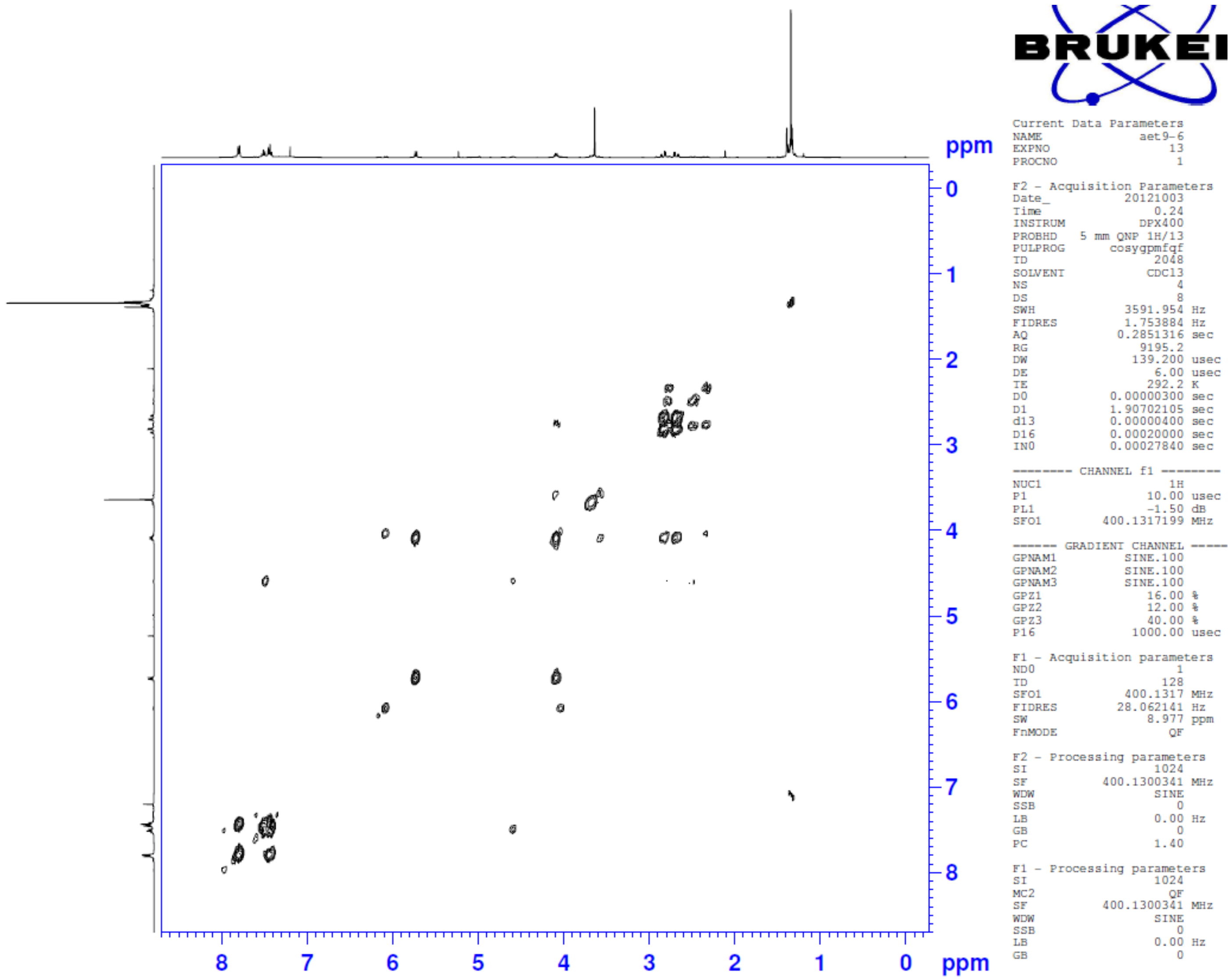

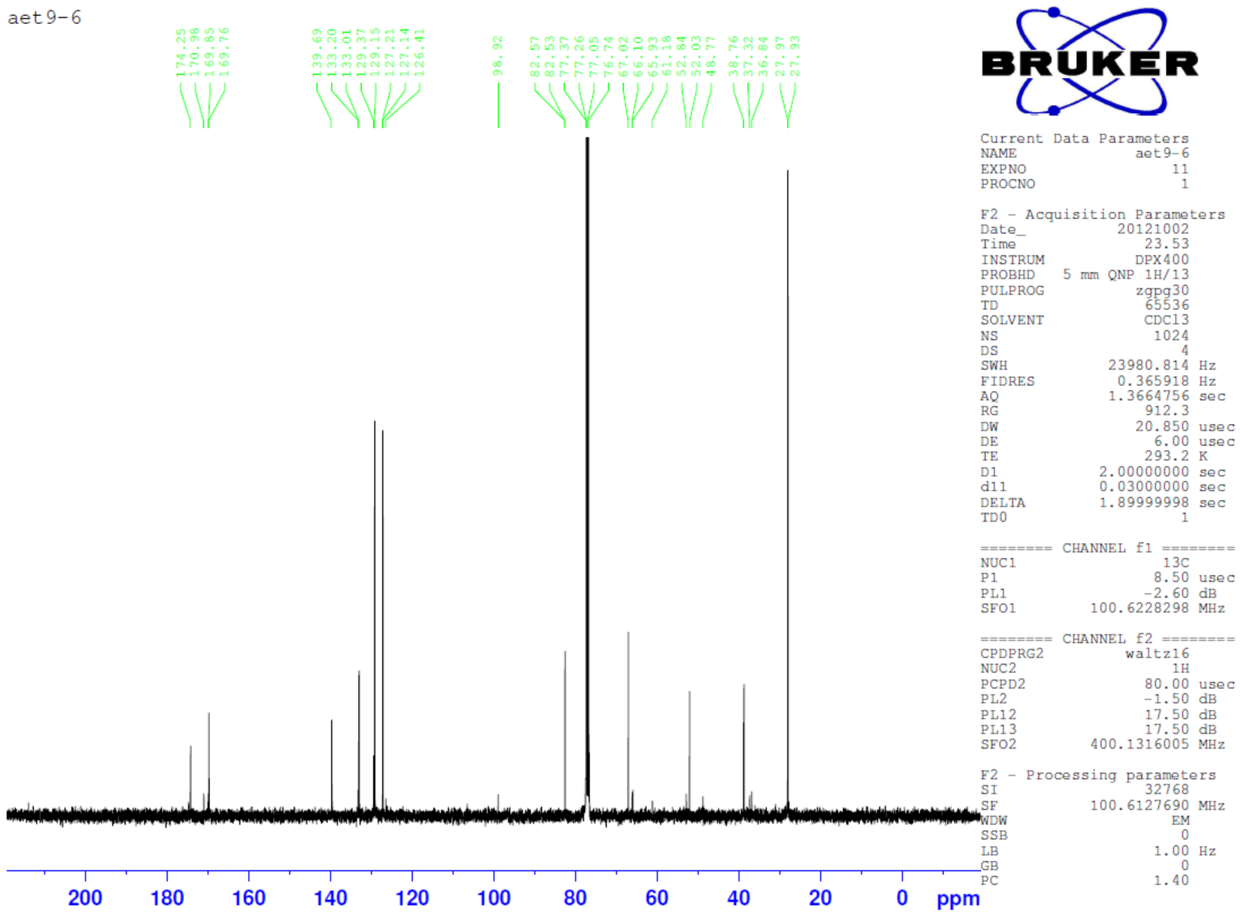
aet9-6
174.25 170.98 169.85 169.76 139.69 133.20 133.01 129.37 129.15 127.21 127.14 126.41 98.92 82.57 82.53 77.37 77.26 77.05 76.74 67.02 66.10 65.93 61.18 52.84 52.03 48.77 38.76 37.32 36.84 27.97 27.93
200 180 160 140 120 100 80 60 40 20 0 ppm
BRUKER
Current Data Parameters
NAME aet9-6
EXPNO 11
PROCNO 1
F2 - Acquisition Parameters
Date_ 20121002
Time 23.53
INSTRUM DPX400
PROBHD 5 mm QNP 1H/13
PULPROG zgpg30
TD 65536
SOLVENT CDCl3
NS 1024
DS 4
SWH 23980.814 Hz
FIDRES 0.365918 Hz
AQ 1.3664756 sec
RG 912.3
DW 20.850 usec
DE 6.00 usec
TE 293.2 K
D1 2.00000000 sec
d11 0.03000000 sec
DELTA 1.89999998 sec
TD0 1
======== CHANNEL f1 ========
NUC1 13C
P1 8.50 usec
PL1 -2.60 dB
SFO1 100.6228298 MHz
======== CHANNEL f2 ========
CPDPRG2 waltz16
NUC2 1H
PCPD2 80.00 usec
PL2 -1.50 dB
PL12 17.50 dB
PL13 17.50 dB
SFO2 400.1316005 MHz
F2 - Processing parameters
SI 32768
SF 100.6127690 MHz
WDW EM
SSB 0
LB 1.00 Hz
GB 0
PC 1.40

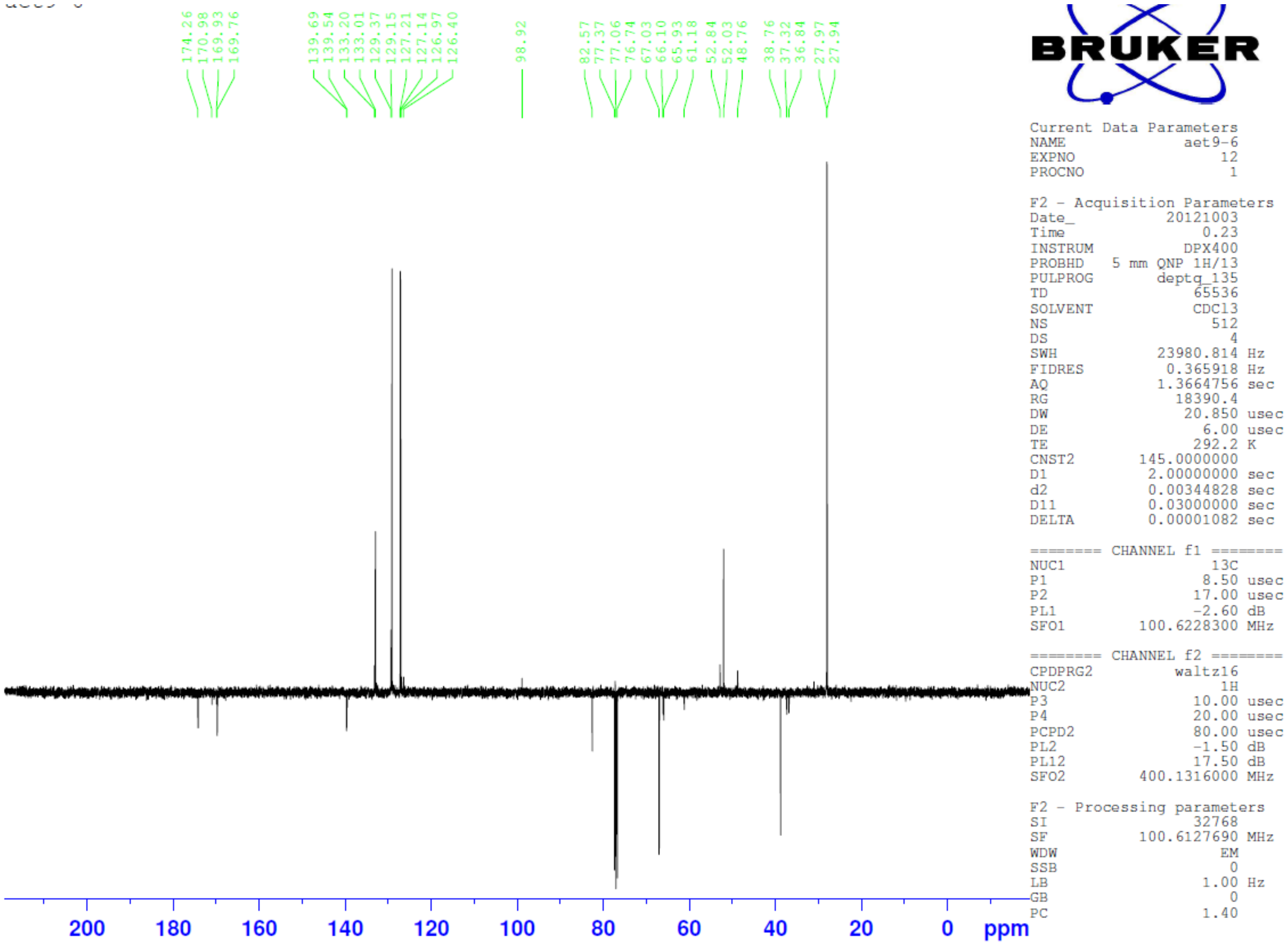
174.26 170.98 169.93 169.76 139.69 139.54 133.20 133.01 129.37 129.15 127.21 127.14 126.97 126.40 98.92 82.57 77.37 77.06 76.74 67.03 66.10 65.93 61.18 52.84 52.03 48.76 38.76 37.32 36.84 27.97 27.94
200 180 160 140 120 100 80 60 40 20 0 ppm
BRUKER
Current Data Parameters
NAME aet9-6
EXPNO 12
PROCNO 1
F2 - Acquisition Parameters
Date_ 20121003
Time 0.23
INSTRUM DPX400
PROBHD 5 mm QNP 1H/13
PULPROG deptq_135
TD 65536
SOLVENT CDCl3
NS 512
DS 4
SWH 23980.814 Hz
FIDRES 0.365918 Hz
AQ 1.3664756 sec
RG 18390.4
DW 20.850 usec
DE 6.00 usec
TE 292.2 K
CNST2 145.0000000
D1 2.00000000 sec
d2 0.00344828 sec
D11 0.03000000 sec
DELTA 0.00001082 sec
======== CHANNEL f1 ========
NUC1 13C
P1 8.50 usec
P2 17.00 usec
PL1 -2.60 dB
SFO1 100.6228300 MHz
======== CHANNEL f2 ========
CPDPRG2 waltz16
NUC2 1H
P3 10.00 usec
P4 20.00 usec
PCPD2 80.00 usec
PL2 -1.50 dB
PL12 17.50 dB
SFO2 400.1316000 MHz
F2 - Processing parameters
SI 32768
SF 100.6127690 MHz
WDW EM
SSB 0
LB 1.00 Hz
GB 0
PC 1.40

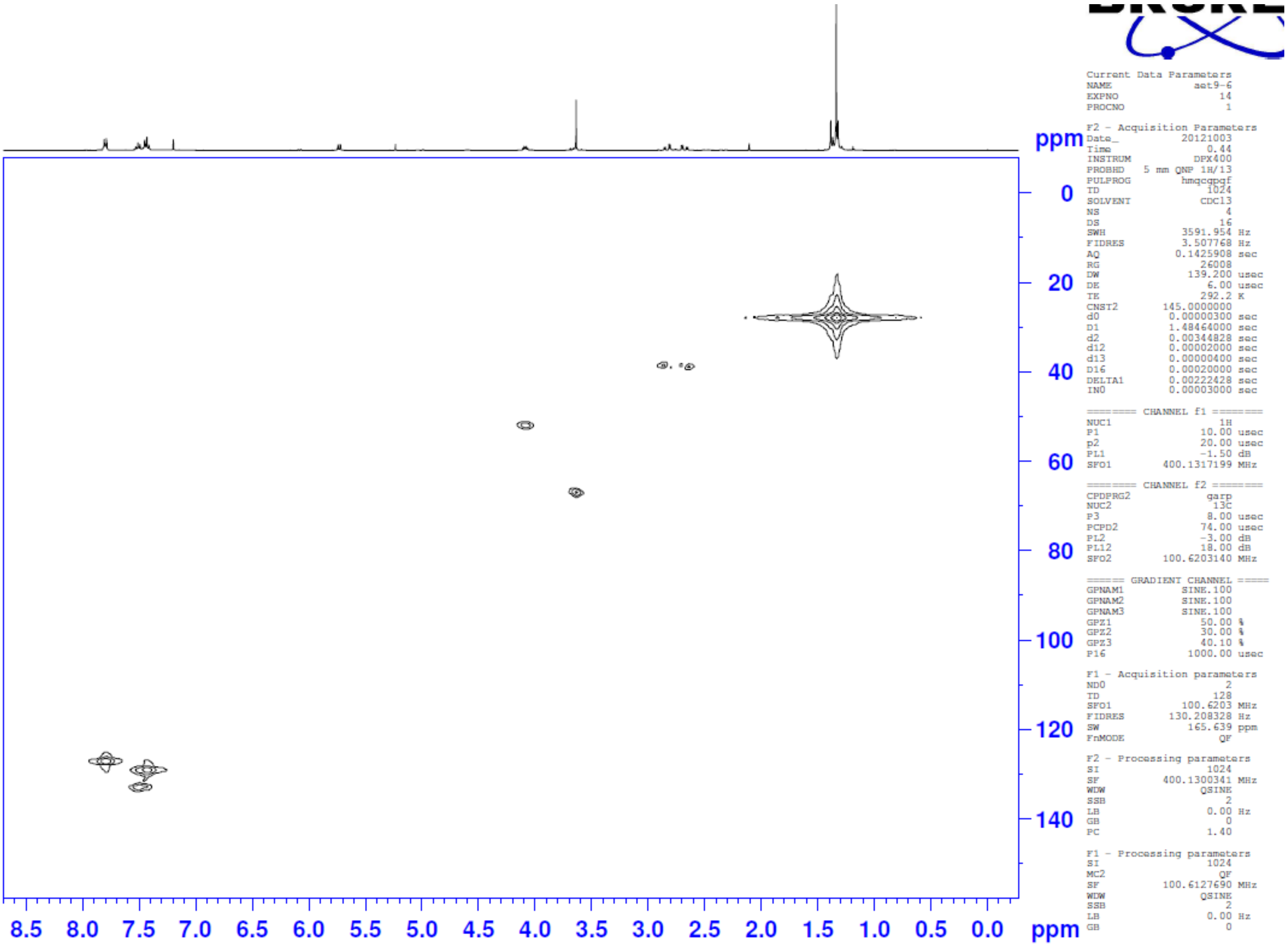

ppm
0
20
40
60
80
100
120
140
8.5 8.0 7.5 7.0 6.5 6.0 5.5 5.0 4.5 4.0 3.5 3.0 2.5 2.0 1.5 1.0 0.5 0.0
ppm
Current Data Parameters
NAME aet9-6
EXPNO 14
PROCNO 1
F2 - Acquisition Parameters
Date_ 20121003
Time 0.44
INSTRUM DPX400
PROBHD 5 mm QNP 1H/13
PULPROG hmqcgpqf
TD 1024
SOLVENT CDCl3
NS 4
DS 16
SWH 3591.954 Hz
FIDRES 3.507768 Hz
AQ 0.1425908 sec
RG 26008
DW 139.200 usec
DE 6.00 usec
TE 292.2 K
CNST2 145.0000000
d0 0.00000300 sec
D1 1.48464000 sec
d2 0.00344828 sec
d12 0.00002000 sec
d13 0.00000400 sec
D16 0.00020000 sec
DELTA1 0.00222428 sec
IN0 0.00003000 sec
======= CHANNEL f1 =======
NUC1 1H
P1 10.00 usec
p2 20.00 usec
PL1 -1.50 dB
SFO1 400.1317199 MHz
======= CHANNEL f2 =======
CPDPRG2 garp
NUC2 13C
P3 8.00 usec
PCPD2 74.00 usec
PL2 -3.00 dB
PL12 18.00 dB
SFO2 100.6203140 MHz
===== GRADIENT CHANNEL =====
GPNAM1 SINE.100
GPNAM2 SINE.100
GPNAM3 SINE.100
GPZ1 50.00 %
GPZ2 30.00 %
GPZ3 40.10 %
P16 1000.00 usec
F1 - Acquisition parameters
ND0 2
TD 128
SFO1 100.6203 MHz
FIDRES 130.208328 Hz
SW 165.639 ppm
FnMODE QF
F2 - Processing parameters
SI 1024
SF 400.1300341 MHz
WDW QSINE
SSB 2
LB 0.00 Hz
GB 0
PC 1.40
F1 - Processing parameters
SI 1024
MC2 QF
SF 100.6127690 MHz
WDW QSINE
SSB 2
LB 0.00 Hz
GB 0

tert-butyl (S)-4-(((1*r,3*R**)-3-(2-(1,8-naphthyridin-2-yl)ethyl)cyclobutyl)amino)-4-oxo-3-(phenylsulfonamido)butanoate [Compound 37]**

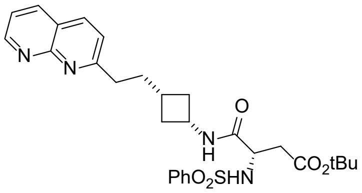

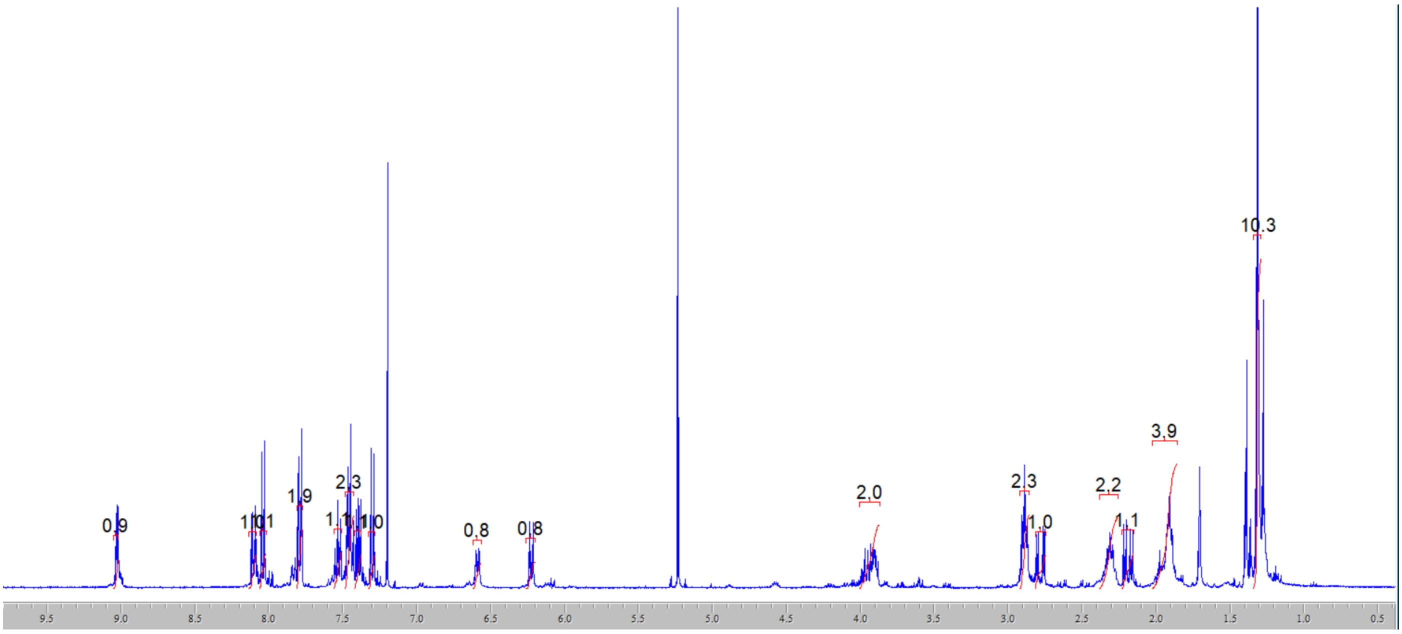

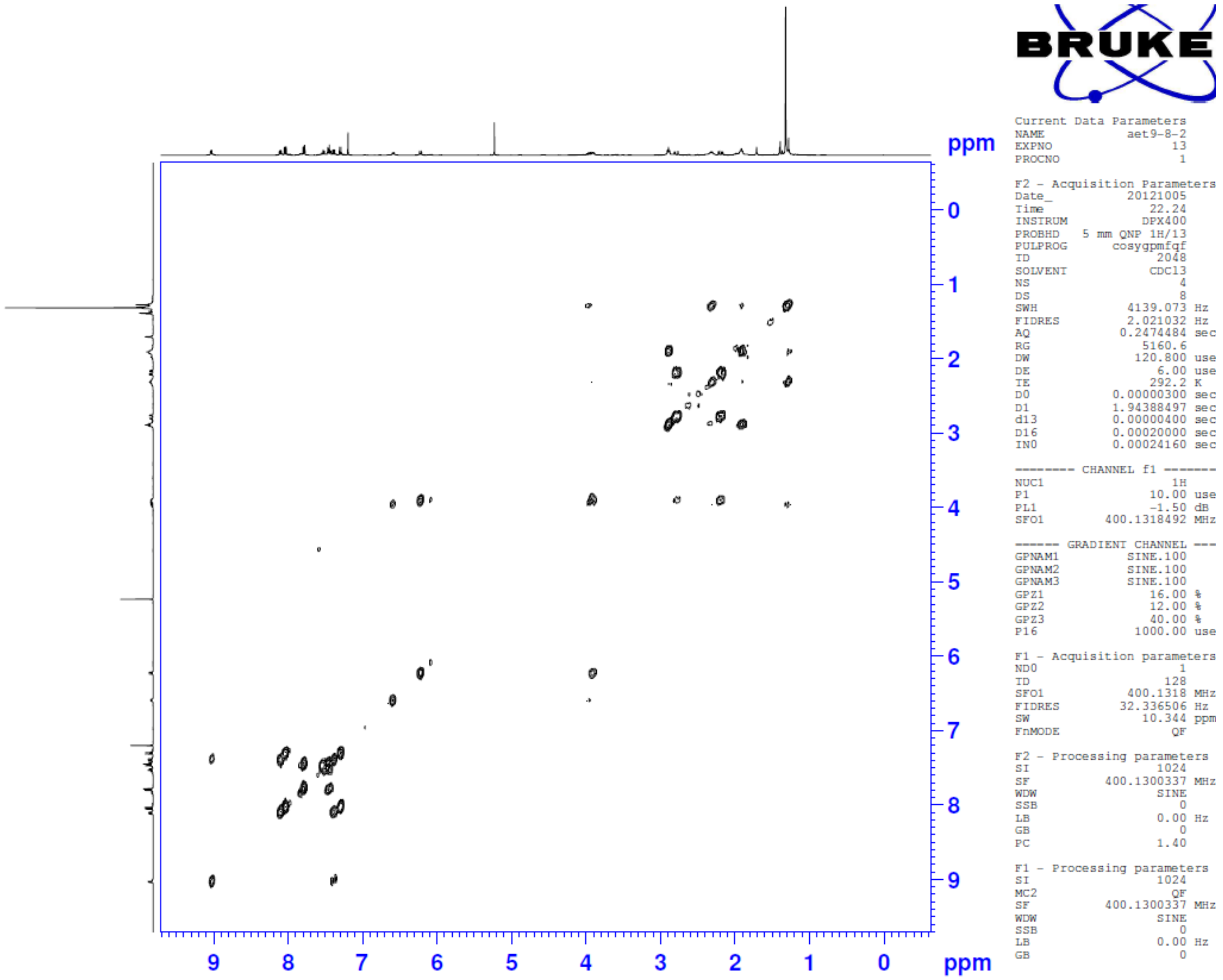

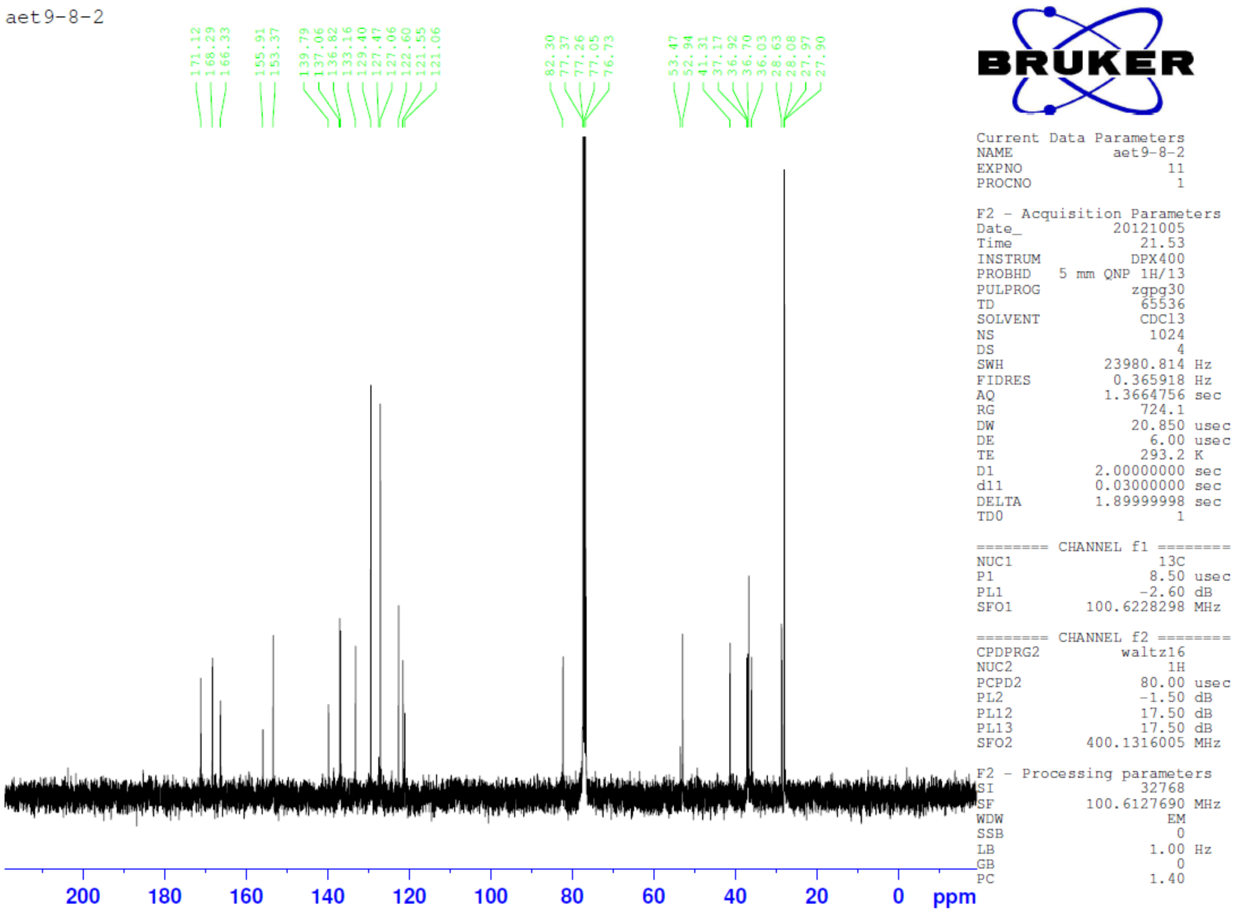
aet9-8-2
171.12
168.29
166.33
155.91
153.37
139.79
137.06
136.82
133.16
129.40
127.47
127.06
122.60
121.55
121.06
82.30
77.37
77.26
77.05
76.73
53.47
52.94
41.31
37.17
36.92
36.70
36.03
28.63
28.08
27.97
27.90
BRUKER
Current Data Parameters
NAME aet9-8-2
EXPNO 11
PROCNO 1
F2 - Acquisition Parameters
Date_ 20121005
Time 21.53
INSTRUM DPX400
PROBHD 5 mm QNP 1H/13
PULPROG zgpg30
TD 65536
SOLVENT CDCl3
NS 1024
DS 4
SWH 23980.814 Hz
FIDRES 0.365918 Hz
AQ 1.3664756 sec
RG 724.1
DW 20.850 usec
DE 6.00 usec
TE 293.2 K
D1 2.00000000 sec
d11 0.03000000 sec
DELTA 1.89999998 sec
TD0 1
======== CHANNEL f1 ========
NUC1 13C
P1 8.50 usec
PL1 -2.60 dB
SFO1 100.6228298 MHz
======== CHANNEL f2 ========
CPDPRG2 waltz16
NUC2 1H
PCPD2 80.00 usec
PL2 -1.50 dB
PL12 17.50 dB
PL13 17.50 dB
SFO2 400.1316005 MHz
F2 - Processing parameters
SI 32768
SF 100.6127690 MHz
WDW EM
SSB 0
LB 1.00 Hz
GB 0
PC 1.40
200 180 160 140 120 100 80 60 40 20 0 ppm

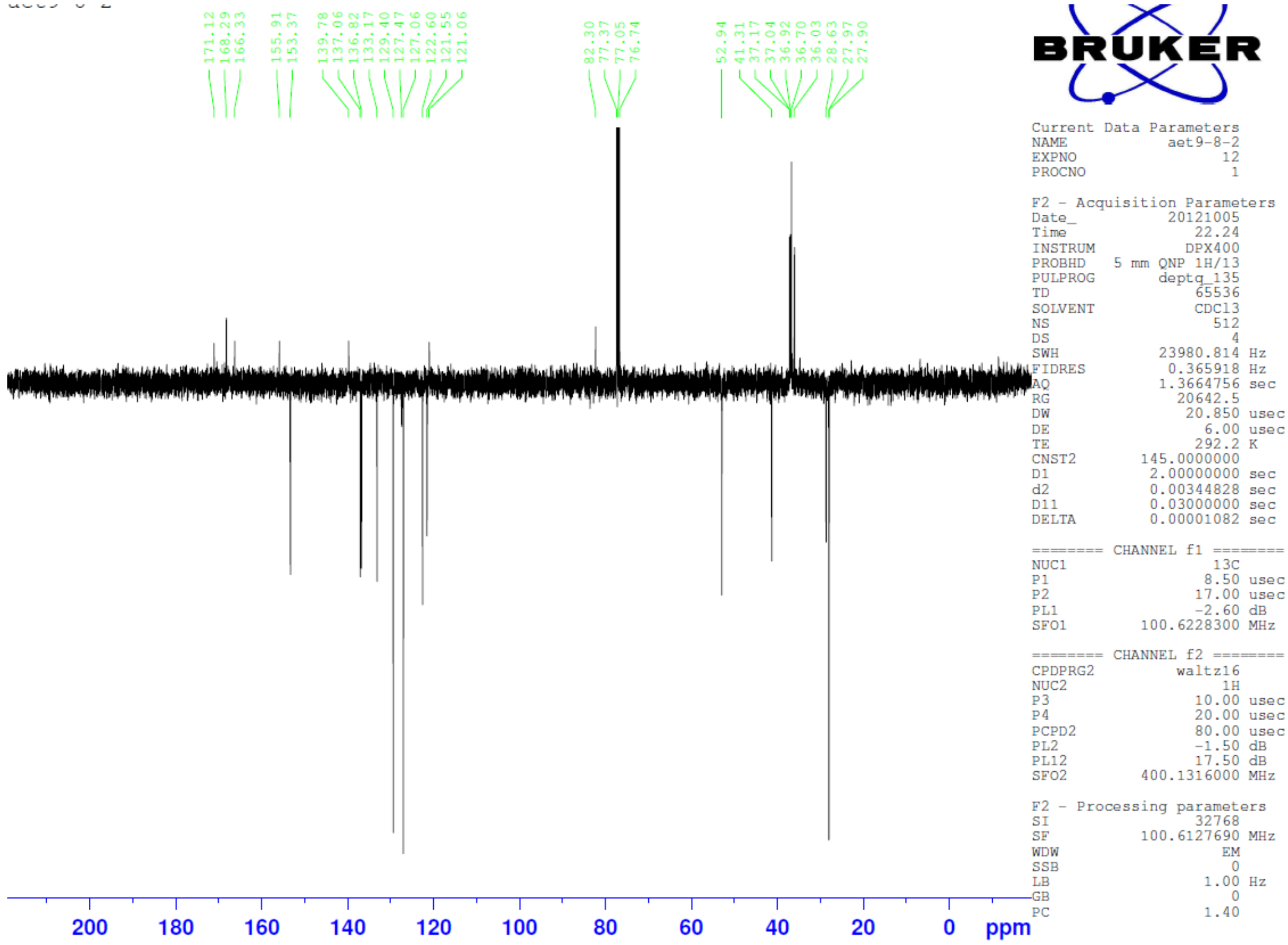
171.12
168.29
166.33
155.91
153.37
139.78
137.06
136.82
133.17
129.40
127.47
127.06
122.60
121.55
121.06
82.30
77.37
77.05
76.74
52.94
41.31
37.17
37.04
36.92
36.70
36.03
28.63
27.97
27.90
BRUKER
Current Data Parameters
NAME aet9-8-2
EXPNO 12
PROCNO 1
F2 - Acquisition Parameters
Date_ 20121005
Time 22.24
INSTRUM DPX400
PROBHD 5 mm QNP 1H/13
PULPROG deptq_135
TD 65536
SOLVENT CDCl3
NS 512
DS 4
SWH 23980.814 Hz
FIDRES 0.365918 Hz
AQ 1.3664756 sec
RG 20642.5
DW 20.850 usec
DE 6.00 usec
TE 292.2 K
CNST2 145.0000000
D1 2.00000000 sec
d2 0.00344828 sec
D11 0.03000000 sec
DELTA 0.00001082 sec
======== CHANNEL f1 ========
NUC1 13C
P1 8.50 usec
P2 17.00 usec
PL1 -2.60 dB
SFO1 100.6228300 MHz
======== CHANNEL f2 ========
CPDPRG2 waltz16
NUC2 1H
P3 10.00 usec
P4 20.00 usec
PCPD2 80.00 usec
PL2 -1.50 dB
PL12 17.50 dB
SFO2 400.1316000 MHz
F2 - Processing parameters
SI 32768
SF 100.6127690 MHz
WDW EM
SSB 0
LB 1.00 Hz
GB 0
PC 1.40
200 180 160 140 120 100 80 60 40 20 0 ppm

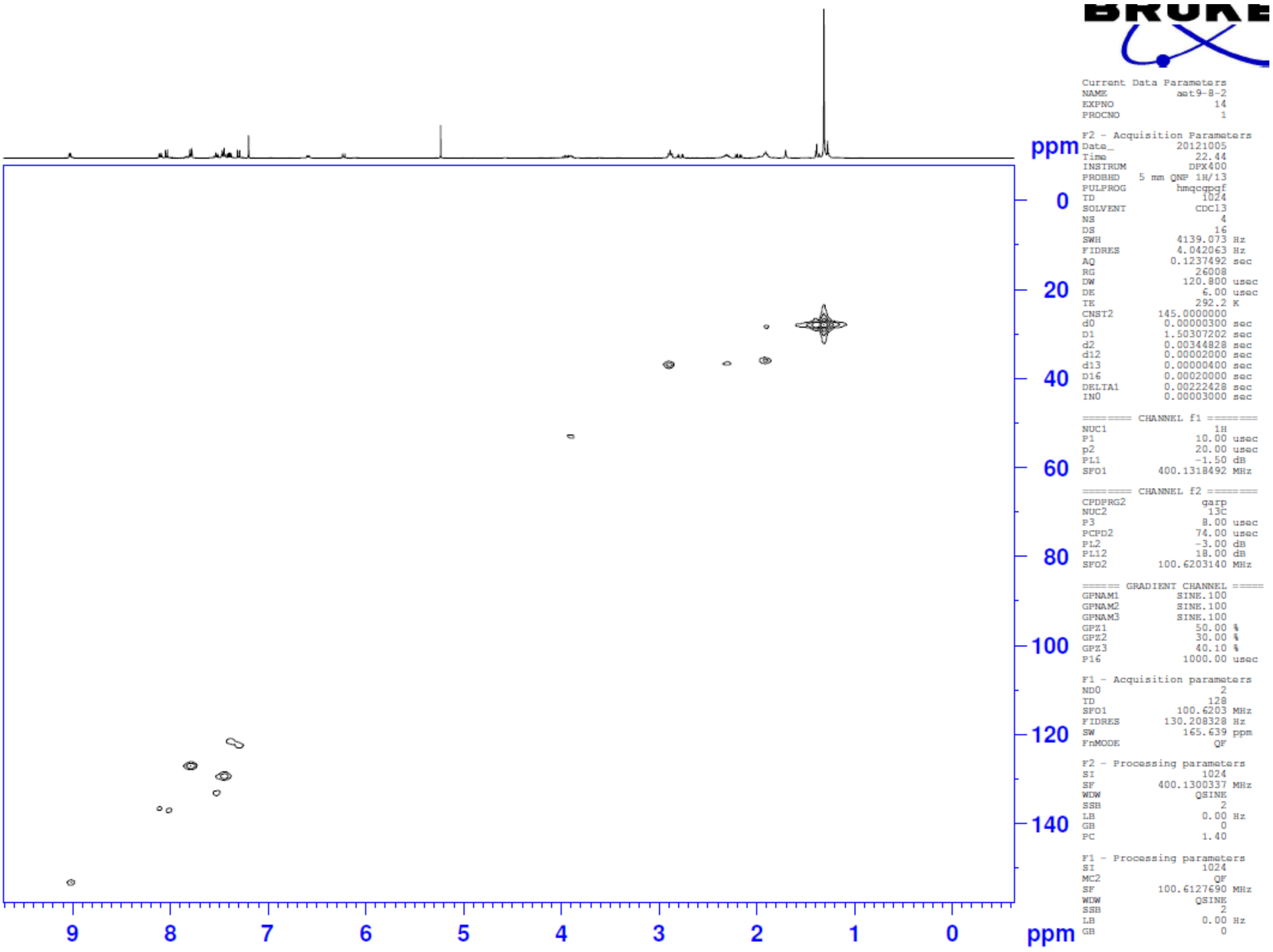
ppm
0
20
40
60
80
100
120
140
9
8
7
6
5
4
3
2
1
0
ppm
Current Data Parameters
NAME aet9-8-2
EXPNO 14
PROCNO 1
F2 - Acquisition Parameters
Date_ 20121005
Time 22.44
INSTRUM DPX400
PROBHD 5 mm QNP 1H/13
PULPROG hmqcgpqf
TD 1024
SOLVENT CDCl3
NS 4
DS 16
SWH 4139.073 Hz
FIDRES 4.042063 Hz
AQ 0.1237492 sec
RG 26008
DW 120.800 usec
DE 6.00 usec
TE 292.2 K
CNST2 145.0000000
d0 0.00000300 sec
D1 1.50307202 sec
d2 0.00344828 sec
d12 0.00002000 sec
d13 0.00000400 sec
D16 0.00020000 sec
DELTA1 0.00222428 sec
IN0 0.00003000 sec
======== CHANNEL f1 ========
NUC1 1H
P1 10.00 usec
p2 20.00 usec
PL1 -1.50 dB
SFO1 400.1318492 MHz
======== CHANNEL f2 ========
CPDPRG2 garp
NUC2 13C
P3 8.00 usec
PCPD2 74.00 usec
PL2 -3.00 dB
PL12 18.00 dB
SFO2 100.6203140 MHz
===== GRADIENT CHANNEL =====
GPNAM1 SINE.100
GPNAM2 SINE.100
GPNAM3 SINE.100
GPZ1 50.00 %
GPZ2 30.00 %
GPZ3 40.10 %
P16 1000.00 usec
F1 - Acquisition parameters
ND0 2
TD 128
SFO1 100.6203 MHz
FIDRES 130.208328 Hz
SW 165.639 ppm
FnMODE QF
F2 - Processing parameters
SI 1024
SF 400.1300337 MHz
WDW QSINE
SSB 2
LB 0.00 Hz
GB 0
PC 1.40
F1 - Processing parameters
SI 1024
MC2 QF
SF 100.6127690 MHz
WDW QSINE
SSB 2
LB 0.00 Hz
GB 0

(*S*)-4-(((1r*,3R*)-3-(2-(1,8-naphthyridin-2-yl)ethyl)cyclobutyl)amino)-4-oxo-3-(phenylsulfonamido)butanoic acid [Compound 38]

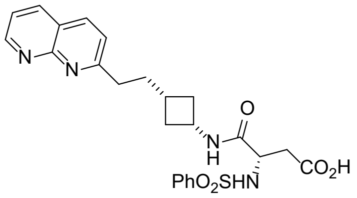

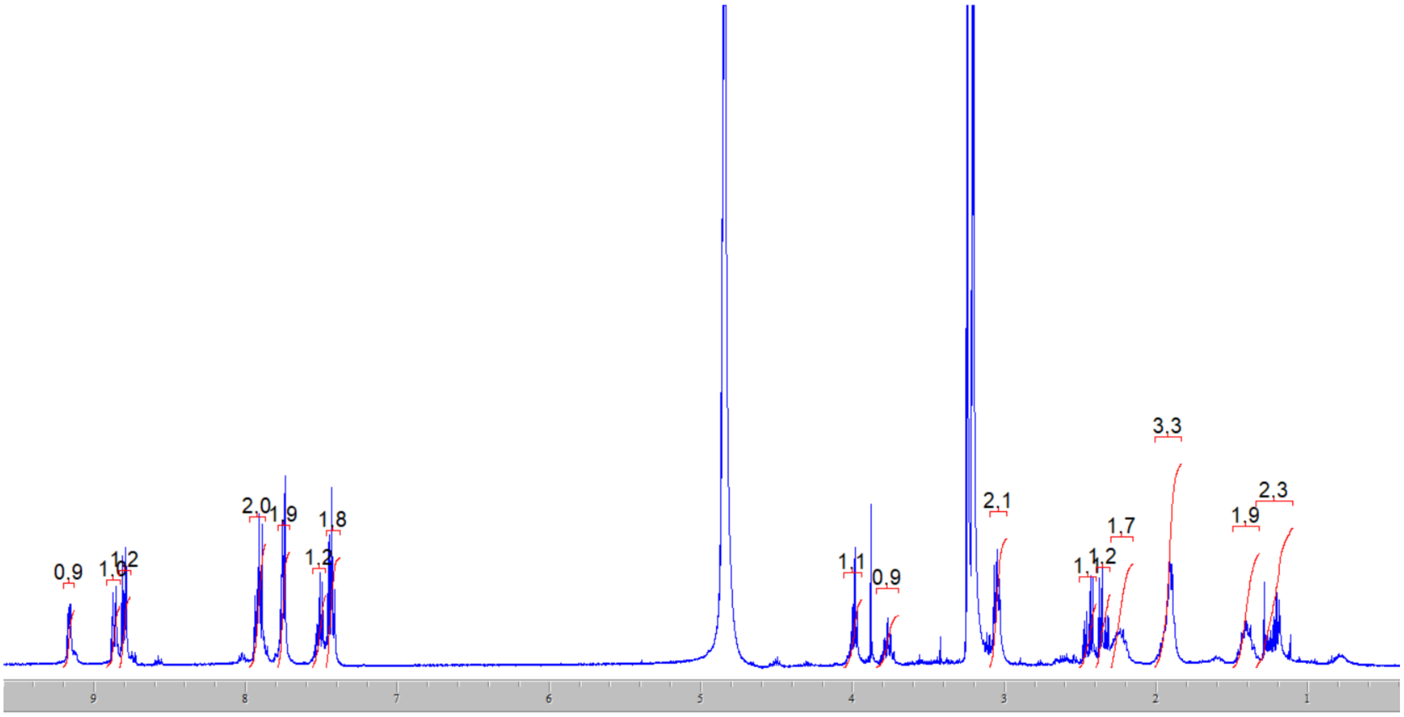

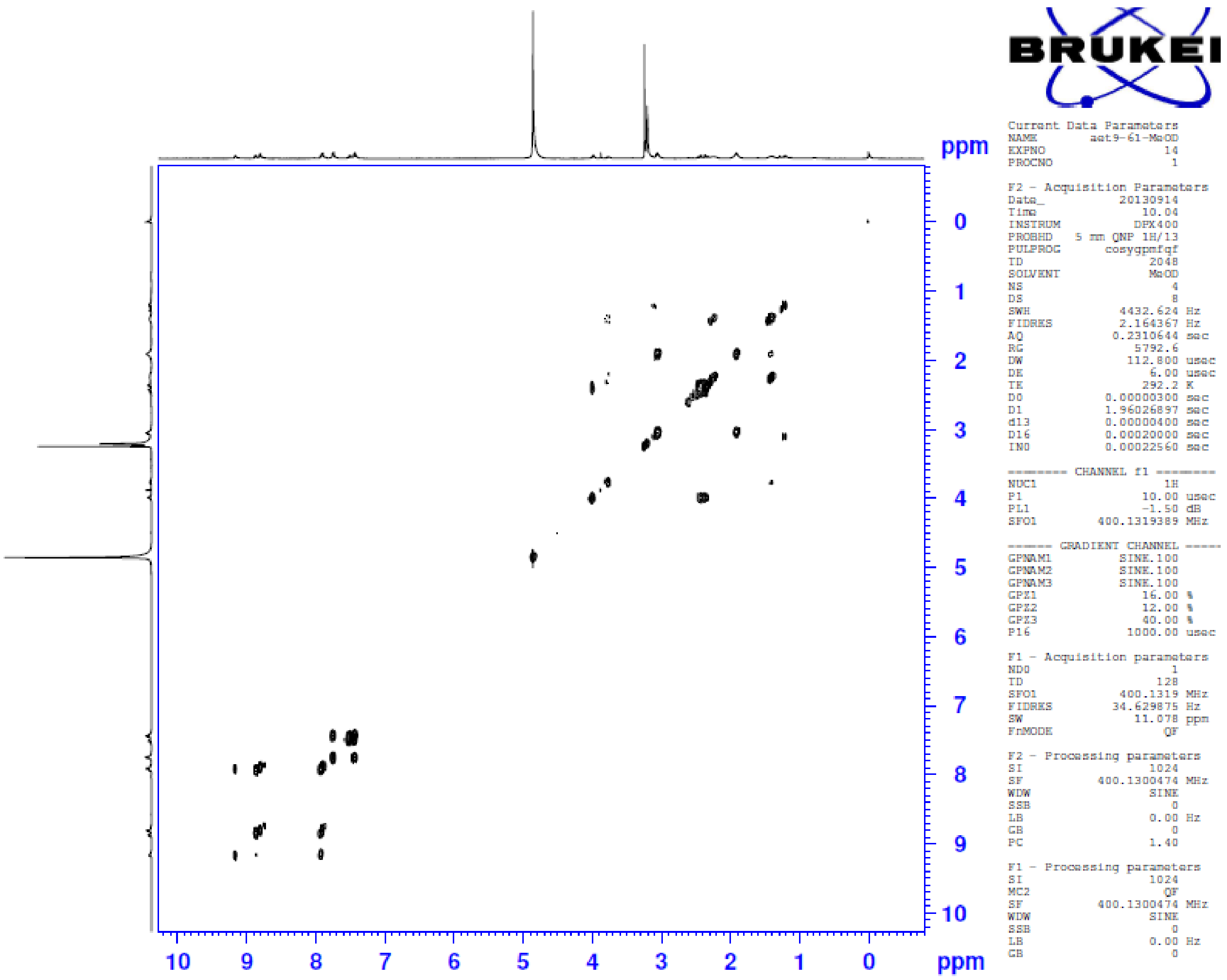

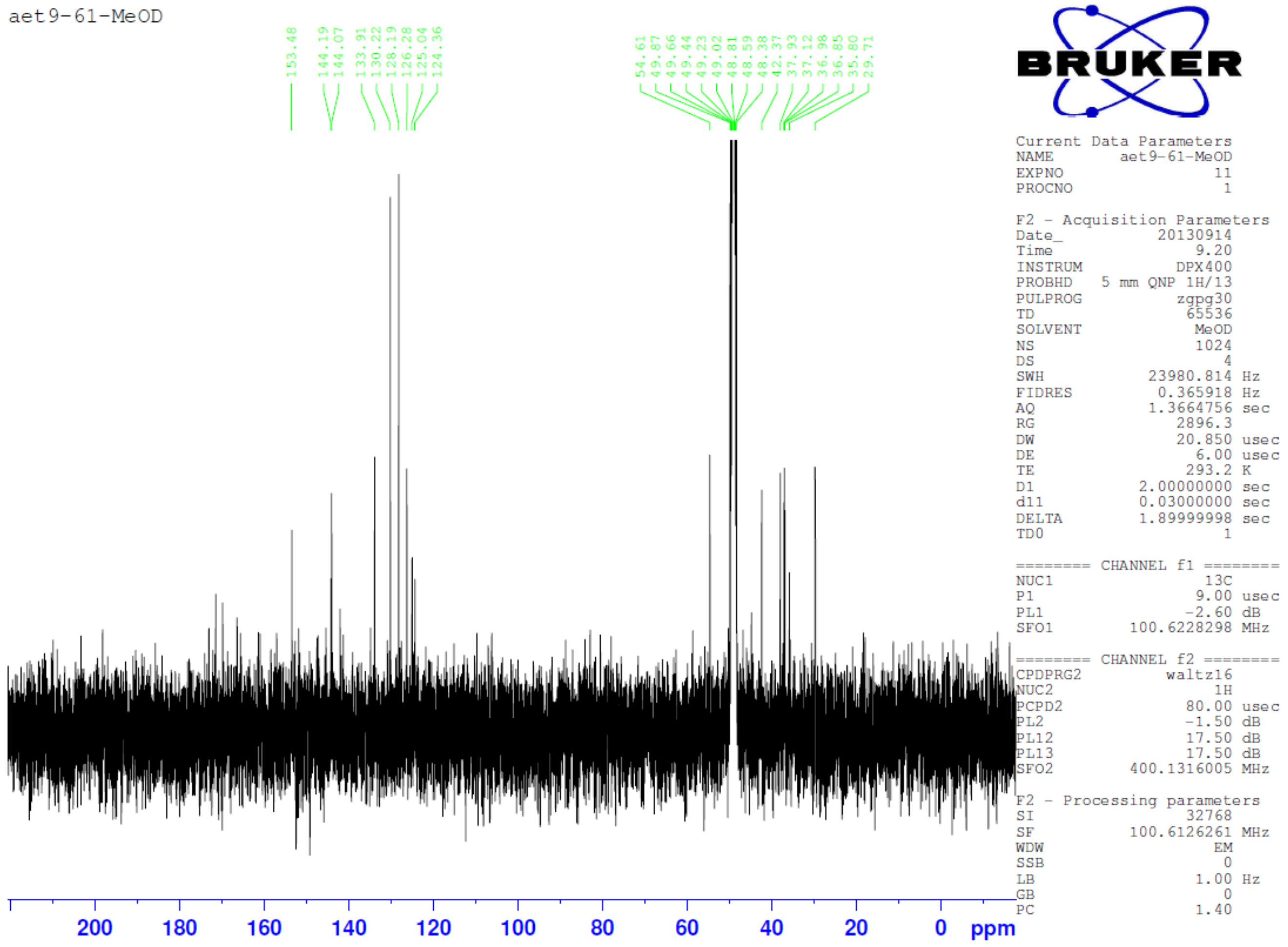
aet9-61-MeOD
153.48
144.19
144.07
133.91
130.22
128.19
126.28
125.04
124.36
54.61
49.87
49.66
49.44
49.23
49.02
48.81
48.59
48.38
42.37
37.93
37.12
36.98
36.85
35.80
29.71
Current Data Parameters
NAME aet9-61-MeOD
EXPNO 11
PROCNO 1
F2 - Acquisition Parameters
Date_ 20130914
Time 9.20
INSTRUM DPX400
PROBHD 5 mm QNP 1H/13
PULPROG zgpg30
TD 65536
SOLVENT MeOD
NS 1024
DS 4
SWH 23980.814 Hz
FIDRES 0.365918 Hz
AQ 1.3664756 sec
RG 2896.3
DW 20.850 usec
DE 6.00 usec
TE 293.2 K
D1 2.00000000 sec
d11 0.03000000 sec
DELTA 1.89999998 sec
TD0 1
======== CHANNEL f1 ========
NUC1 13C
P1 9.00 usec
PL1 -2.60 dB
SFO1 100.6228298 MHz
======== CHANNEL f2 ========
CPDPRG2 waltz16
NUC2 1H
PCPD2 80.00 usec
PL2 -1.50 dB
PL12 17.50 dB
PL13 17.50 dB
SFO2 400.1316005 MHz
F2 - Processing parameters
SI 32768
SF 100.6126261 MHz
WDW EM
SSB 0
LB 1.00 Hz
GB 0
PC 1.40
200 180 160 140 120 100 80 60 40 20 0 ppm

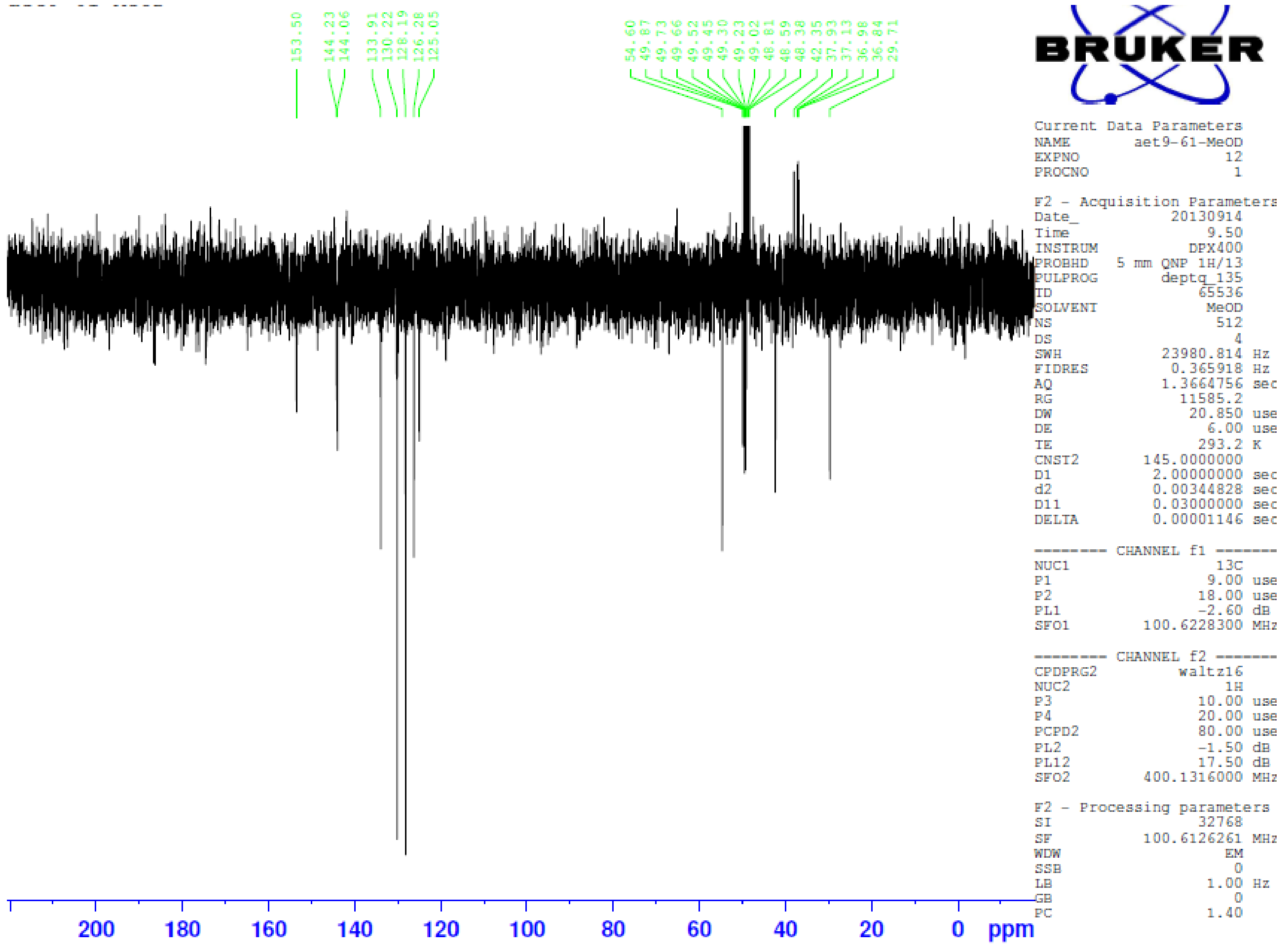
153.50
144.23
144.06
133.91
130.22
128.19
126.28
125.05
54.60
49.87
49.73
49.66
49.52
49.45
49.30
49.23
49.02
48.81
48.59
48.38
42.35
37.93
37.13
36.98
36.84
29.71
Current Data Parameters
NAME aet9-61-MeOD
EXPNO 12
PROCNO 1
F2 - Acquisition Parameters
Date_ 20130914
Time 9.50
INSTRUM DPX400
PROBHD 5 mm QNP 1H/13
PULPROG deptq_135
TD 65536
SOLVENT MeOD
NS 512
DS 4
SWH 23980.814 Hz
FIDRES 0.365918 Hz
AQ 1.3664756 sec
RG 11585.2
DW 20.850 use
DE 6.00 use
TE 293.2 K
CNST2 145.0000000
D1 2.00000000 sec
d2 0.00344828 sec
D11 0.03000000 sec
DELTA 0.00001146 sec
======== CHANNEL f1 ========
NUC1 13C
P1 9.00 use
P2 18.00 use
PL1 -2.60 dB
SFO1 100.6228300 MHz
======== CHANNEL f2 ========
CPDPRG2 waltz16
NUC2 1H
P3 10.00 use
P4 20.00 use
PCPD2 80.00 use
PL2 -1.50 dB
PL12 17.50 dB
SFO2 400.1316000 MHz
F2 - Processing parameters
SI 32768
SF 100.6126261 MHz
WDW EM
SSB 0
LB 1.00 Hz
GB 0
PC 1.40
200 180 160 140 120 100 80 60 40 20 0 ppm

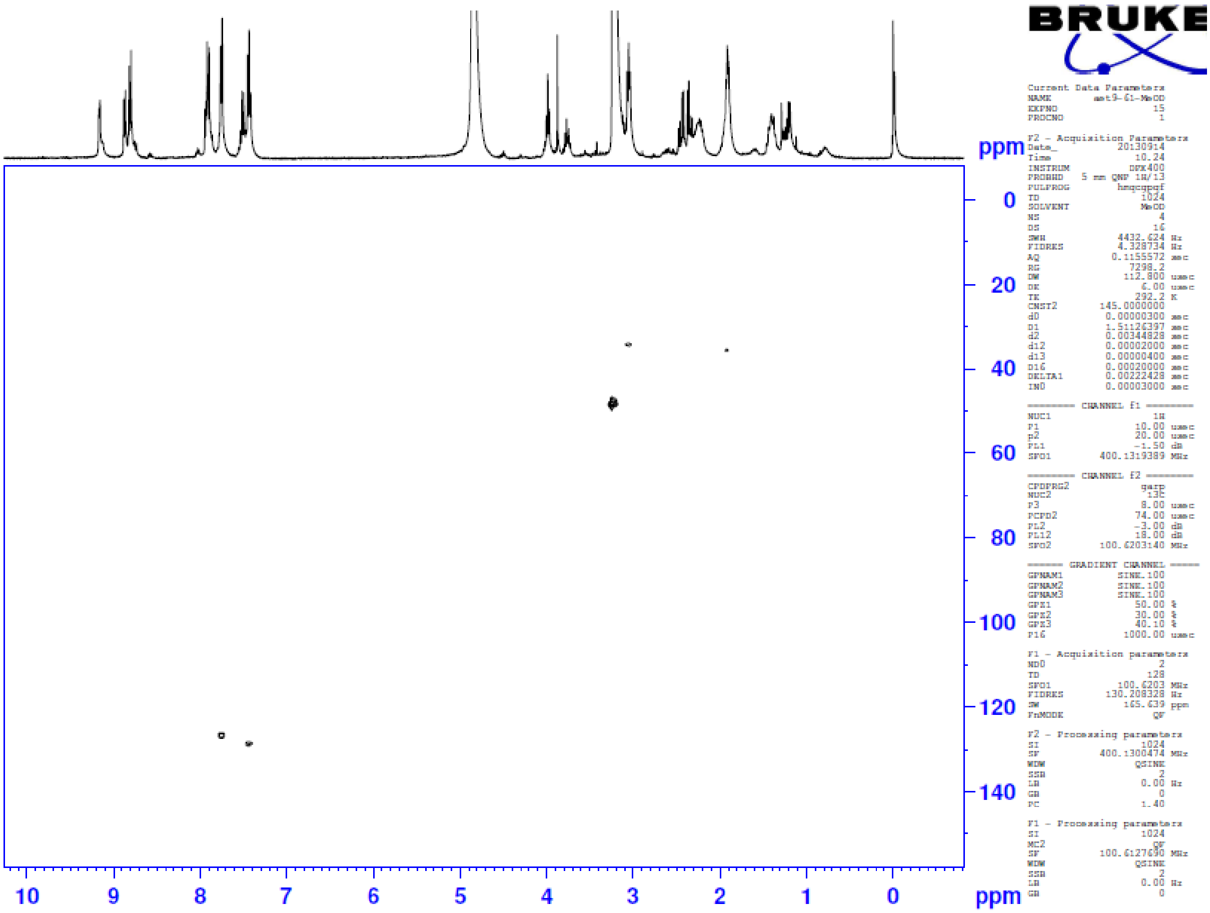

ppm
0
20
40
60
80
100
120
140
10
9
8
7
6
5
4
3
2
1
0
ppm
Current Data Parameters
NAME amt9-61-MeOD
EXPNO 15
PROCNO 1
F2 - Acquisition Parameters
Date_ 20130914
Time 10.24
INSTRUM DPX400
PROBHD 5 mm QNP 1H/13
PULPROG hmqcgpqf
TD 1024
SOLVENT MeOD
NS 4
DS 16
SWH 4432.624 Hz
FIDRES 4.328734 Hz
AQ 0.1155572 sec
RG 7298.2
DW 112.800 usec
DE 6.00 usec
TE 292.2 K
CNST2 145.0000000
d0 0.00000300 sec
D1 1.51126397 sec
d2 0.00344828 sec
d12 0.00002000 sec
d13 0.00000400 sec
D16 0.00020000 sec
DELTA1 0.00222428 sec
IN0 0.00003000 sec
======== CHANNEL f1 ========
NUC1 1H
P1 10.00 usec
p2 20.00 usec
PL1 -1.50 dB
SFO1 400.1319389 MHz
======== CHANNEL f2 ========
CPDPRG2 garp
NUC2 13C
P3 8.00 usec
PCPD2 74.00 usec
PL2 -3.00 dB
PL12 18.00 dB
SFO2 100.6203140 MHz
====== GRADIENT CHANNEL =====
GPNAM1 SINE.100
GPNAM2 SINE.100
GPNAM3 SINE.100
GPZ1 50.00 %
GPZ2 30.00 %
GPZ3 40.10 %
P16 1000.00 usec
F1 - Acquisition parameters
ND0 2
TD 128
SFO1 100.6203 MHz
FIDRES 130.208328 Hz
SW 165.639 ppm
FnMODE QF
F2 - Processing parameters
SI 1024
SF 400.1300474 MHz
WDW QSINE
SSB 2
LB 0.00 Hz
GB 0
PC 1.40
F1 - Processing parameters
SI 1024
MC2 QF
SF 100.6127690 MHz
WDW QSINE
SSB 2
LB 0.00 Hz
GB 0

tert-butyl (*S*)-4-oxo-3-(phenylsulfonamido)-4-(((1*r,3*R**)-3-(2-(5,6,7,8-tetrahydro-1,8-naphthyridin-2-yl)ethyl)cyclobutyl)amino)butanoate [Compound 39]**

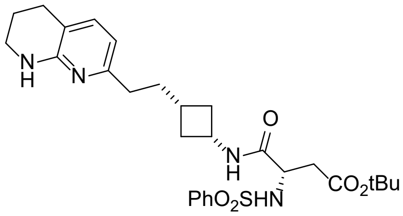

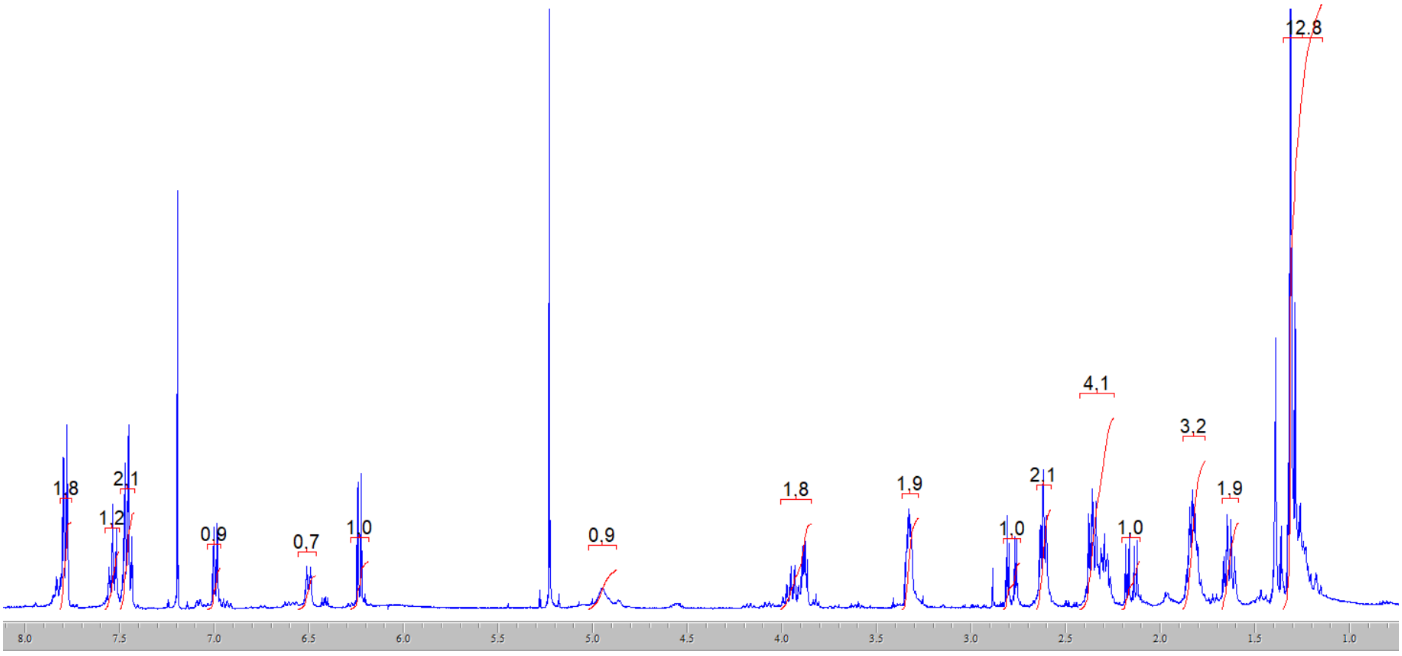

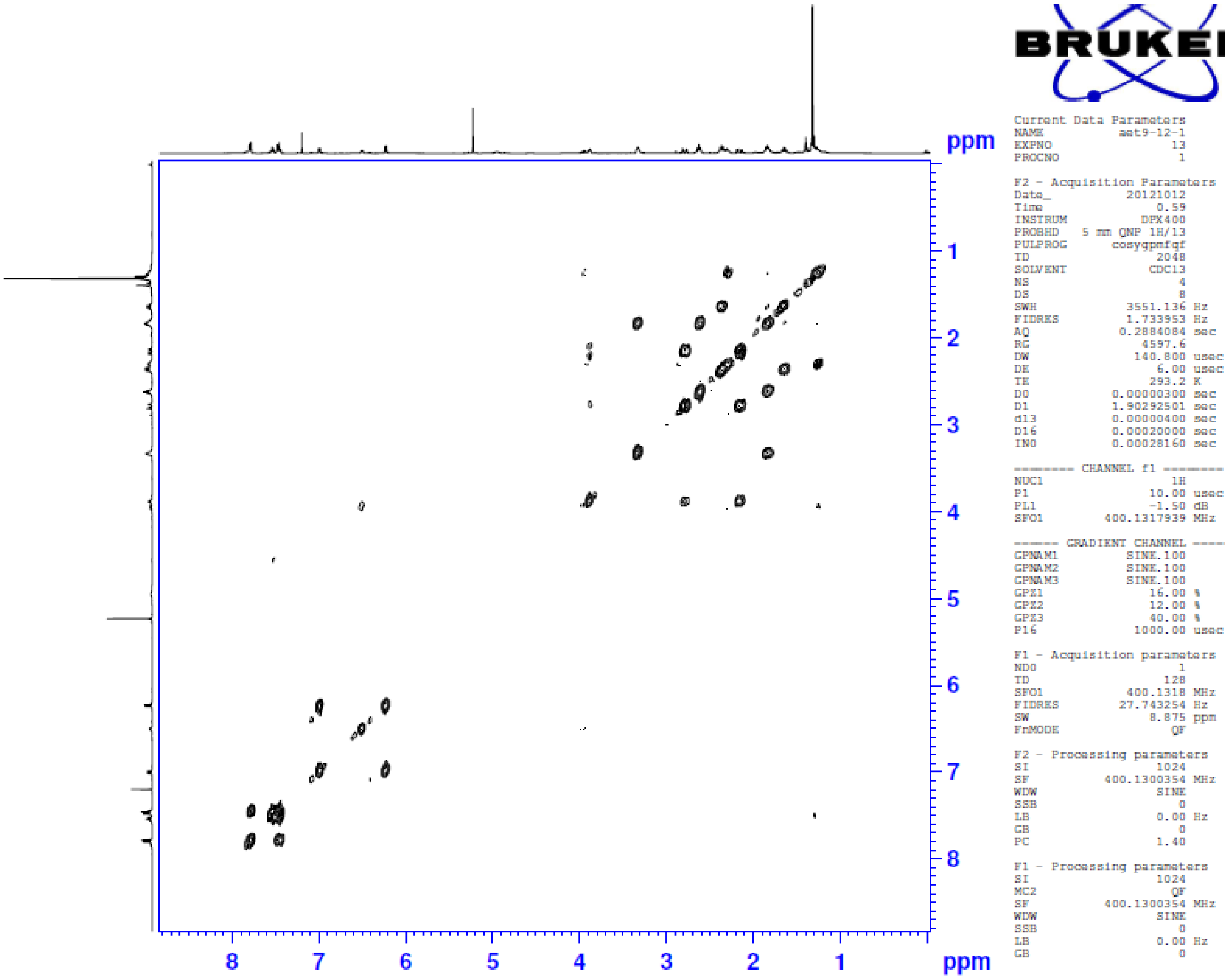

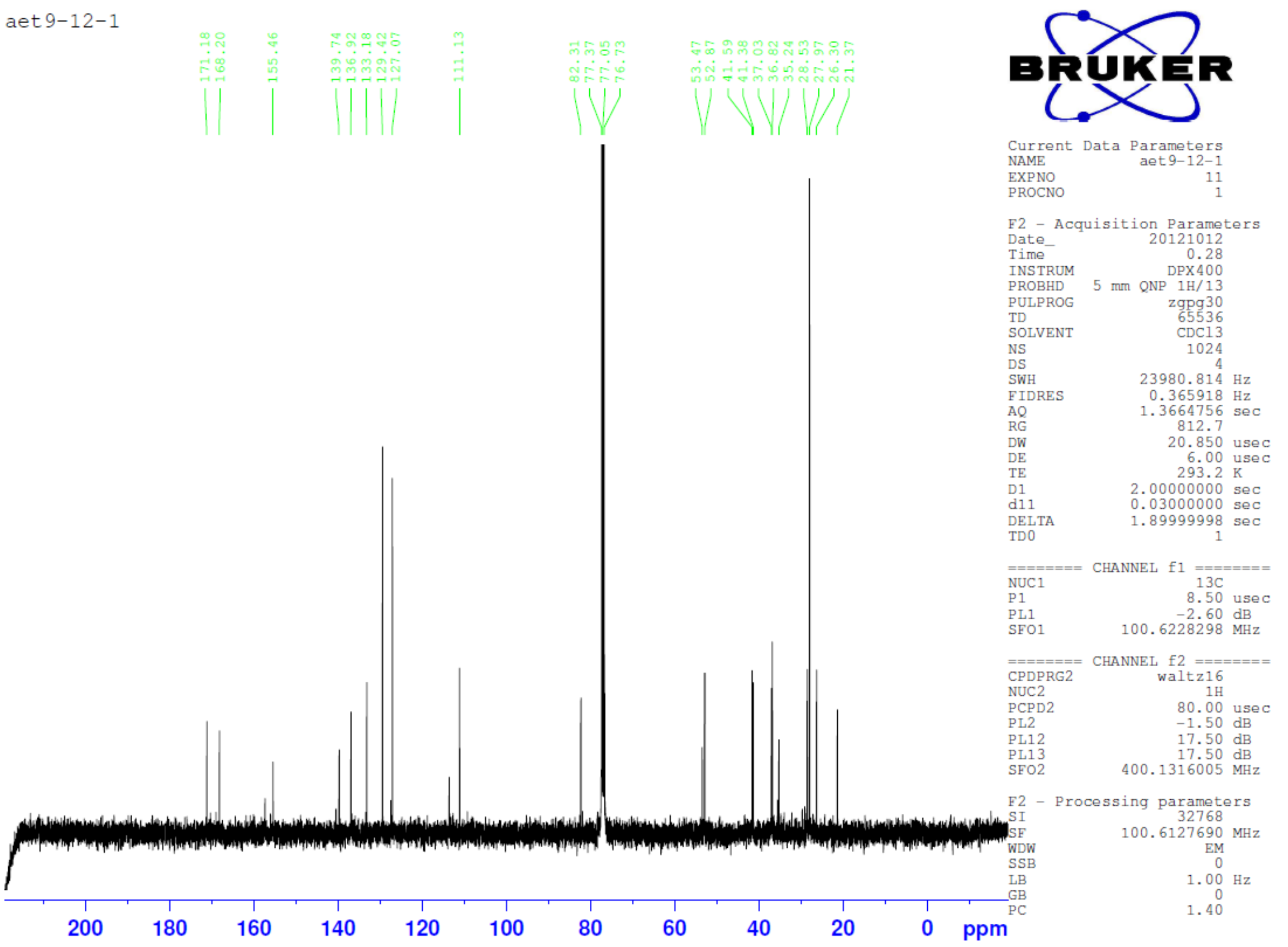
aet9-12-1
171.18
168.20
155.46
139.74
136.92
133.18
129.42
127.07
111.13
82.31
77.37
77.05
76.73
53.47
52.87
41.59
41.38
37.03
36.82
35.24
28.53
27.97
26.30
21.37
BRUKER
Current Data Parameters
NAME aet9-12-1
EXPNO 11
PROCNO 1
F2 - Acquisition Parameters
Date_ 20121012
Time 0.28
INSTRUM DPX400
PROBHD 5 mm QNP 1H/13
PULPROG zgpg30
TD 65536
SOLVENT CDCl3
NS 1024
DS 4
SWH 23980.814 Hz
FIDRES 0.365918 Hz
AQ 1.3664756 sec
RG 812.7
DW 20.850 usec
DE 6.00 usec
TE 293.2 K
D1 2.00000000 sec
d11 0.03000000 sec
DELTA 1.89999998 sec
TD0 1
======== CHANNEL f1 ========
NUC1 13C
P1 8.50 usec
PL1 -2.60 dB
SFO1 100.6228298 MHz
======== CHANNEL f2 ========
CPDPRG2 waltz16
NUC2 1H
PCPD2 80.00 usec
PL2 -1.50 dB
PL12 17.50 dB
PL13 17.50 dB
SFO2 400.1316005 MHz
F2 - Processing parameters
SI 32768
SF 100.6127690 MHz
WDW EM
SSB 0
LB 1.00 Hz
GB 0
PC 1.40
200
180
160
140
120
100
80
60
40
20
0
ppm

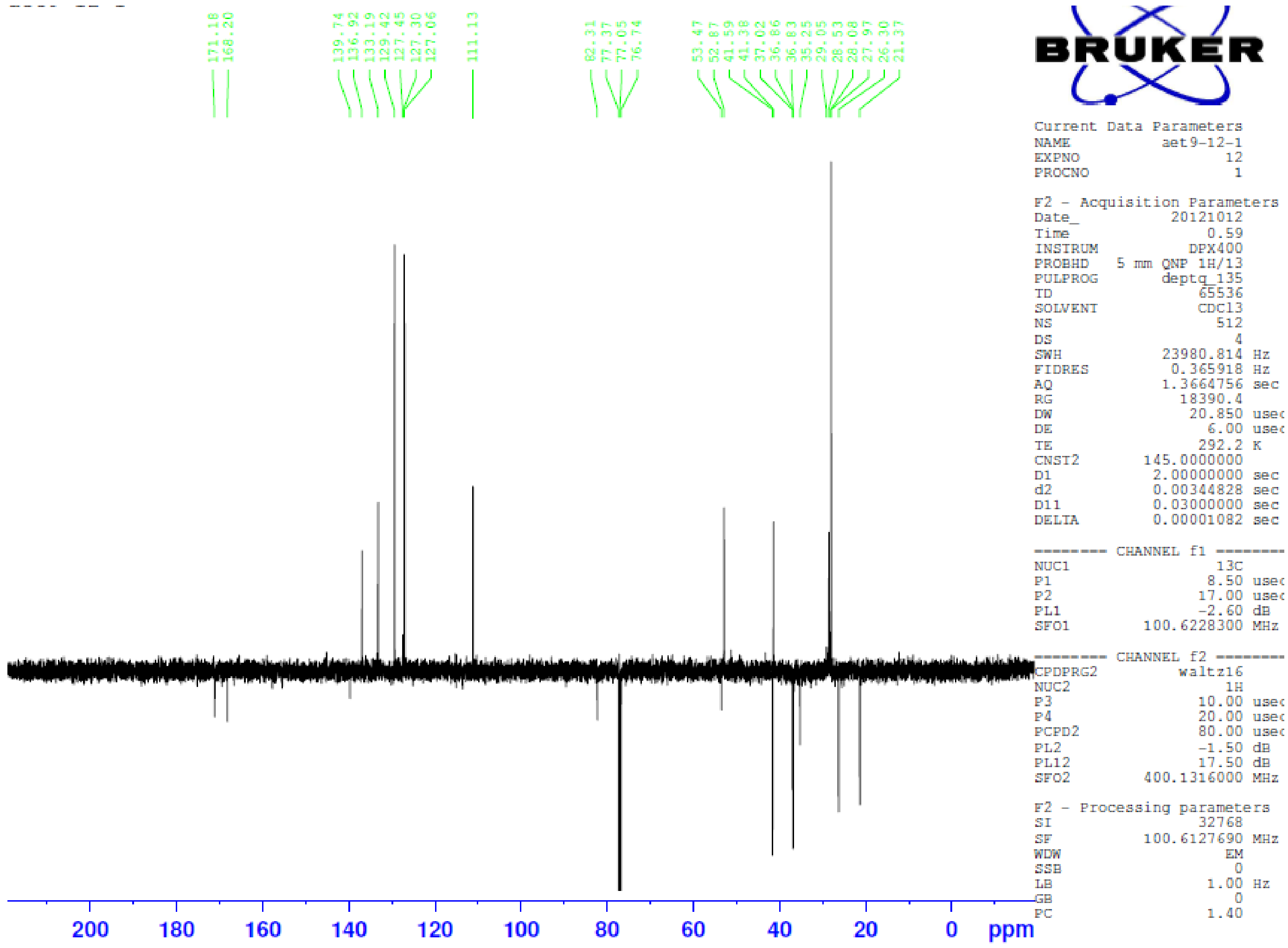
171.18
168.20
139.74
136.92
133.19
129.42
127.45
127.30
127.06
111.13
82.31
77.37
77.05
76.74
53.47
52.87
41.59
41.38
37.02
36.86
36.83
35.25
29.05
28.53
28.08
27.97
26.30
21.37
BRUKER
Current Data Parameters
NAME aet9-12-1
EXPNO 12
PROCNO 1
F2 - Acquisition Parameters
Date_ 20121012
Time 0.59
INSTRUM DPX400
PROBHD 5 mm QNP 1H/13
PULPROG deptq_135
TD 65536
SOLVENT CDCl3
NS 512
DS 4
SWH 23980.814 Hz
FIDRES 0.365918 Hz
AQ 1.3664756 sec
RG 18390.4
DW 20.850 usec
DE 6.00 usec
TE 292.2 K
CNST2 145.0000000
D1 2.00000000 sec
d2 0.00344828 sec
D11 0.03000000 sec
DELTA 0.00001082 sec
======== CHANNEL f1 ========
NUC1 13C
P1 8.50 usec
P2 17.00 usec
PL1 -2.60 dB
SFO1 100.6228300 MHz
======== CHANNEL f2 ========
CPDPRG2 waltz16
NUC2 1H
P3 10.00 usec
P4 20.00 usec
PCPD2 80.00 usec
PL2 -1.50 dB
PL12 17.50 dB
SFO2 400.1316000 MHz
F2 - Processing parameters
SI 32768
SF 100.6127690 MHz
WDW EM
SSB 0
LB 1.00 Hz
GB 0
PC 1.40
200
180
160
140
120
100
80
60
40
20
0
ppm

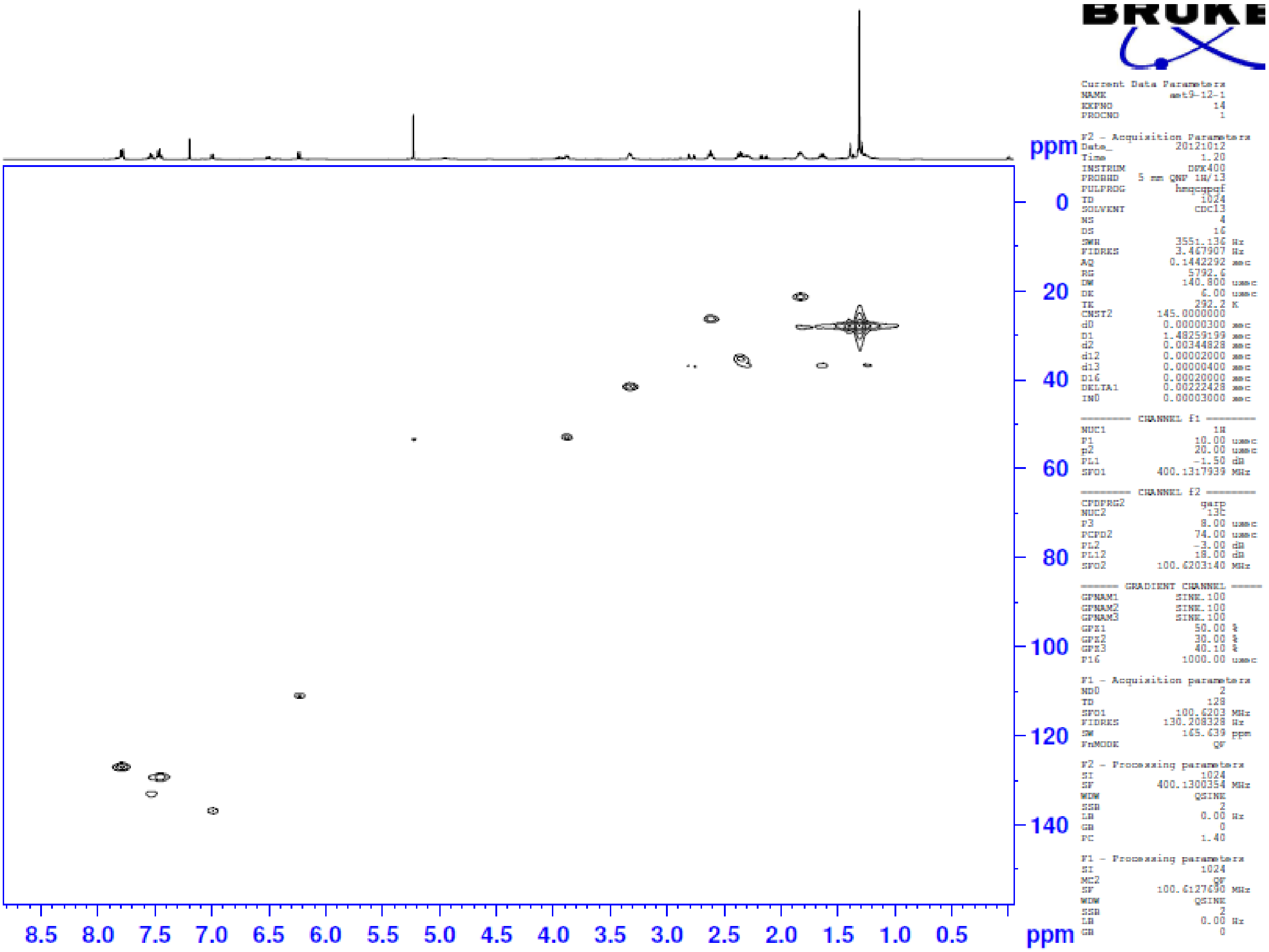

ppm
0
20
40
60
80
100
120
140
8.5 8.0 7.5 7.0 6.5 6.0 5.5 5.0 4.5 4.0 3.5 3.0 2.5 2.0 1.5 1.0 0.5
ppm
Current Data Parameters
NAME met9-12-1
EXPNO 14
PROCNO 1
F2 - Acquisition Parameters
Date_ 20121012
Time 1.20
INSTRUM DPX400
PROBHD 5 mm QNP 1H/13
PULPROG hmqcgpqf
TD 1024
SOLVENT CDCl3
NS 4
DS 16
SWH 3551.136 Hz
FIDRES 3.467907 Hz
AQ 0.1442292 sec
RG 5792.6
DW 140.800 usec
DE 6.00 usec
TE 292.2 K
CNST2 145.0000000
d0 0.00000300 sec
D1 1.48259199 sec
d2 0.00344828 sec
d12 0.00002000 sec
d13 0.00000400 sec
D16 0.00020000 sec
DELTA1 0.00222428 sec
IN0 0.00003000 sec
======== CHANNEL f1 ========
NUC1 1H
P1 10.00 usec
p2 20.00 usec
PL1 -1.50 dB
SFO1 400.1317939 MHz
======== CHANNEL f2 ========
CPDPRG2 garp
NUC2 13C
P3 8.00 usec
PCPD2 74.00 usec
PL2 -3.00 dB
PL12 18.00 dB
SFO2 100.6203140 MHz
====== GRADIENT CHANNEL =====
GPNAM1 SINE.100
GPNAM2 SINE.100
GPNAM3 SINE.100
GPZ1 50.00 %
GPZ2 30.00 %
GPZ3 40.10 %
P16 1000.00 usec
F1 - Acquisition parameters
ND0 2
TD 128
SFO1 100.6203 MHz
FIDRES 130.208328 Hz
SW 165.639 ppm
FnMODE QF
F2 - Processing parameters
SI 1024
SF 400.1300354 MHz
WDW QSINE
SSB 2
LB 0.00 Hz
GB 0
PC 1.40
F1 - Processing parameters
SI 1024
MC2 QF
SF 100.6127690 MHz
WDW QSINE
SSB 2
LB 0.00 Hz
GB 0

(*S*)-4-oxo-3-(phenylsulfonamido)-4-(((1r*,3R*)-3-(2-(5,6,7,8-tetrahydro-1,8-naphthyridin-2-yl)ethyl)cyclobutyl)amino)butanoic acid [Compound 40]

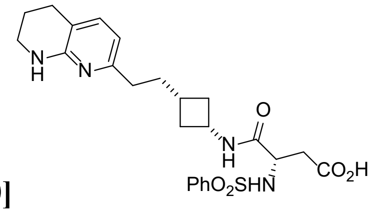

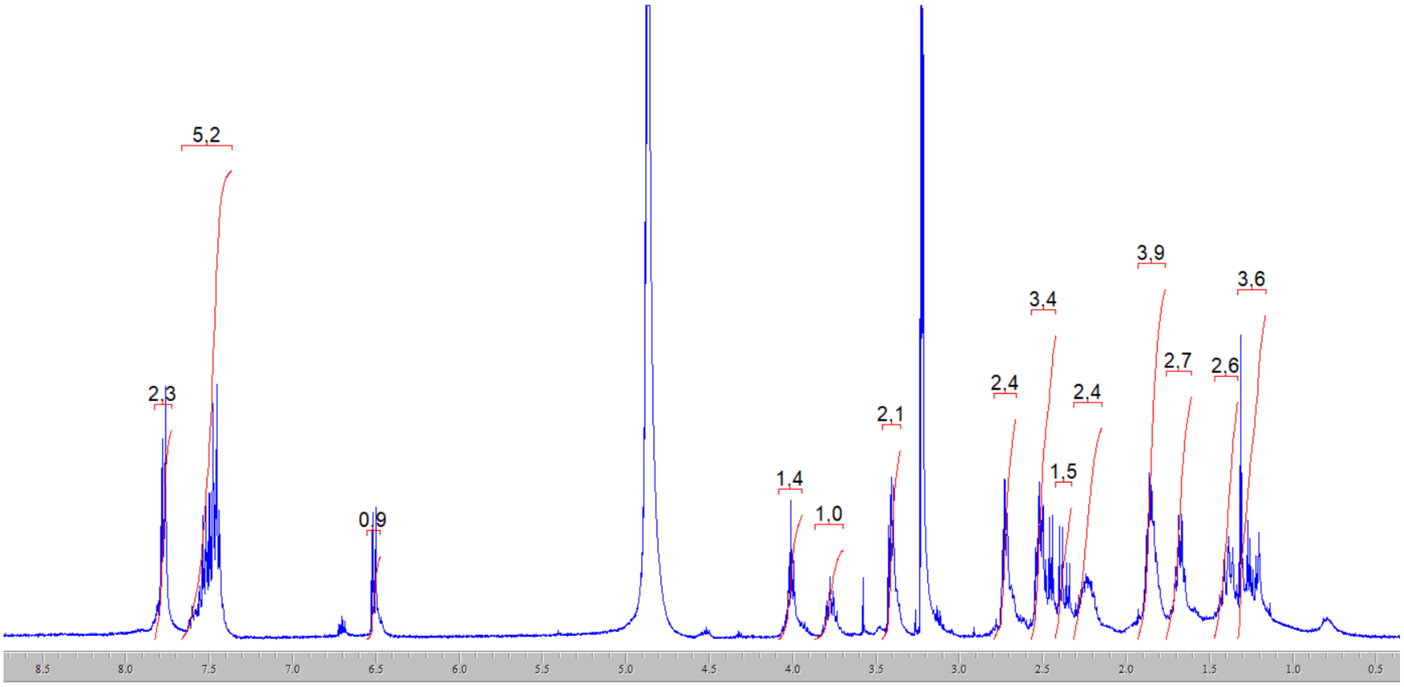

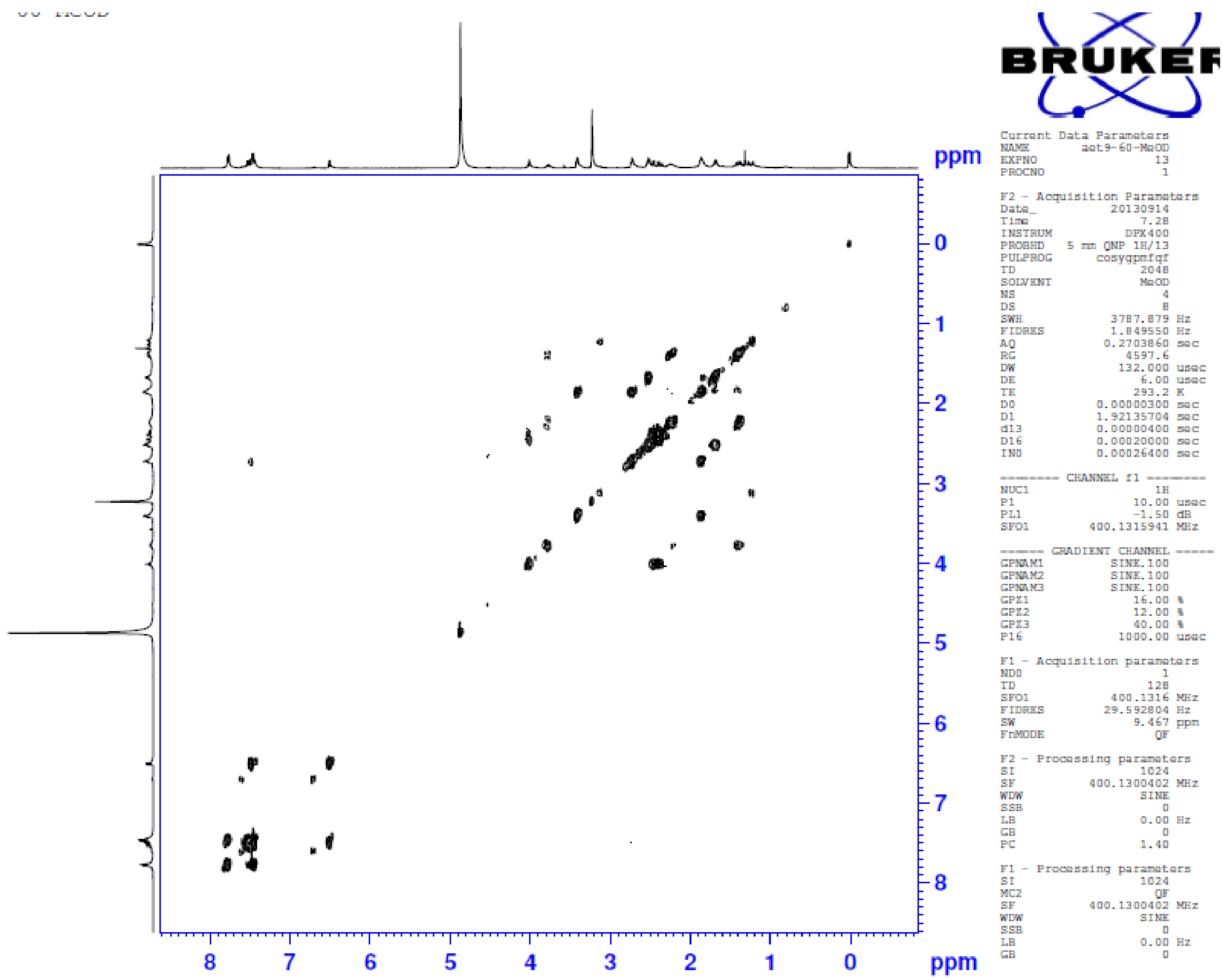

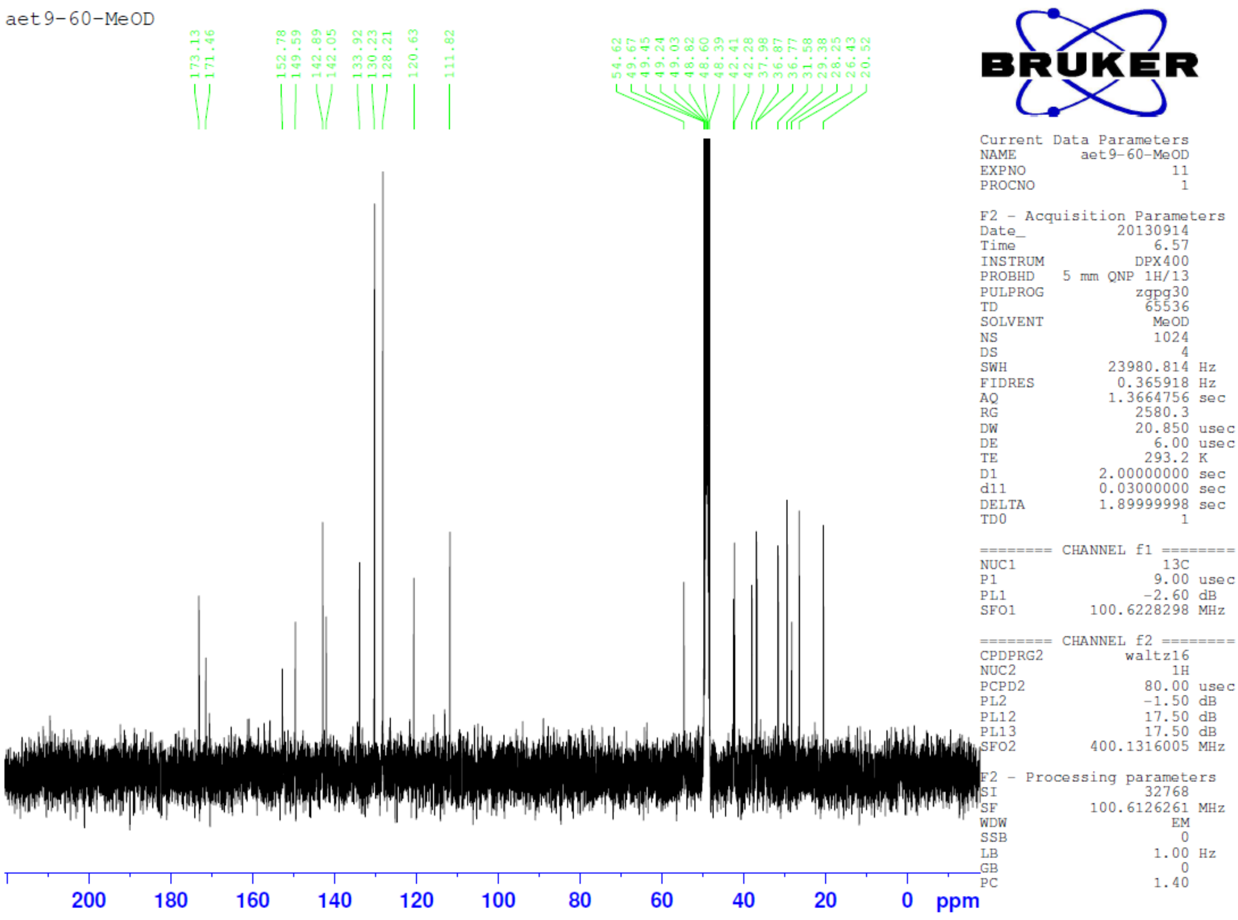

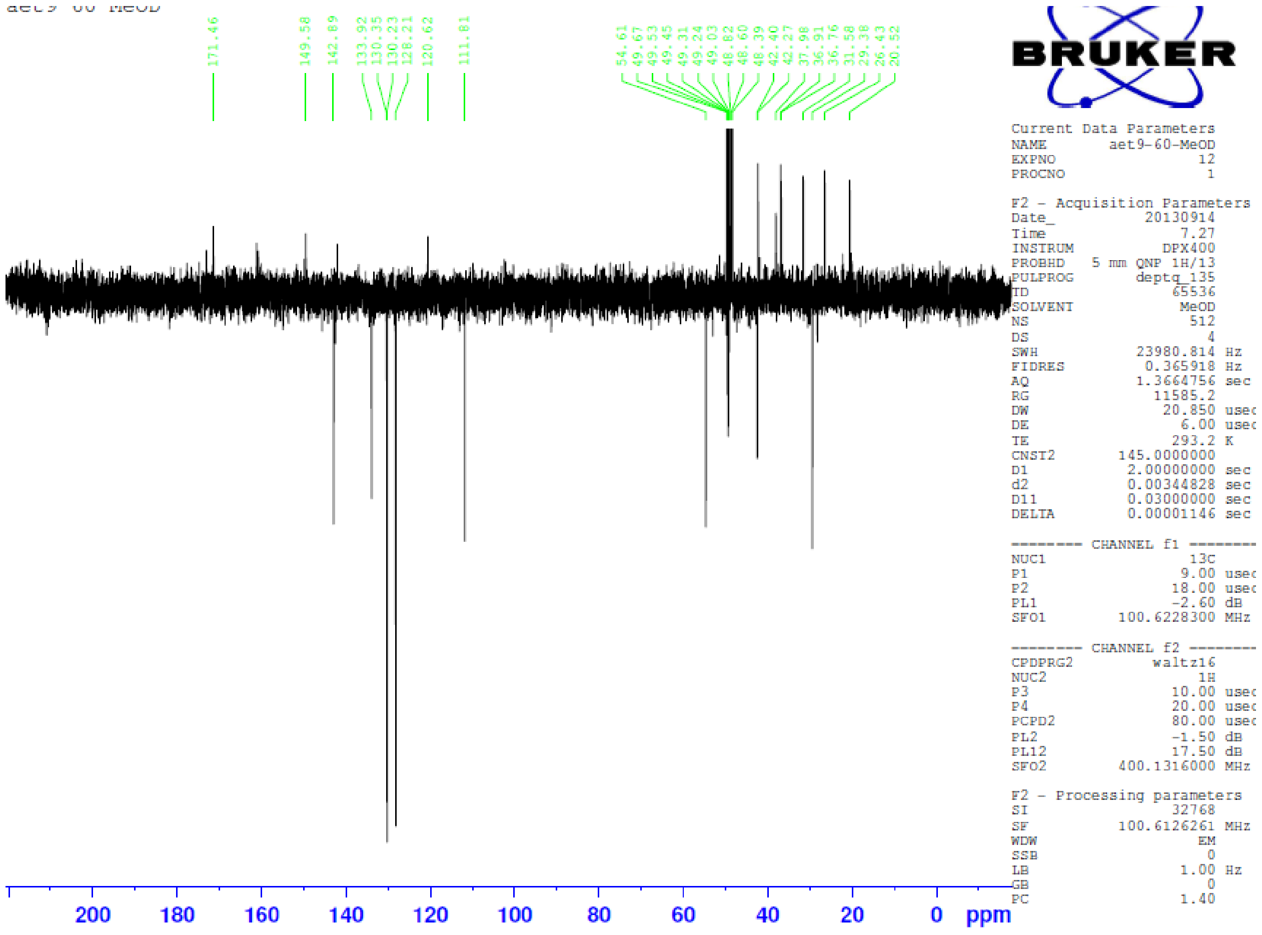

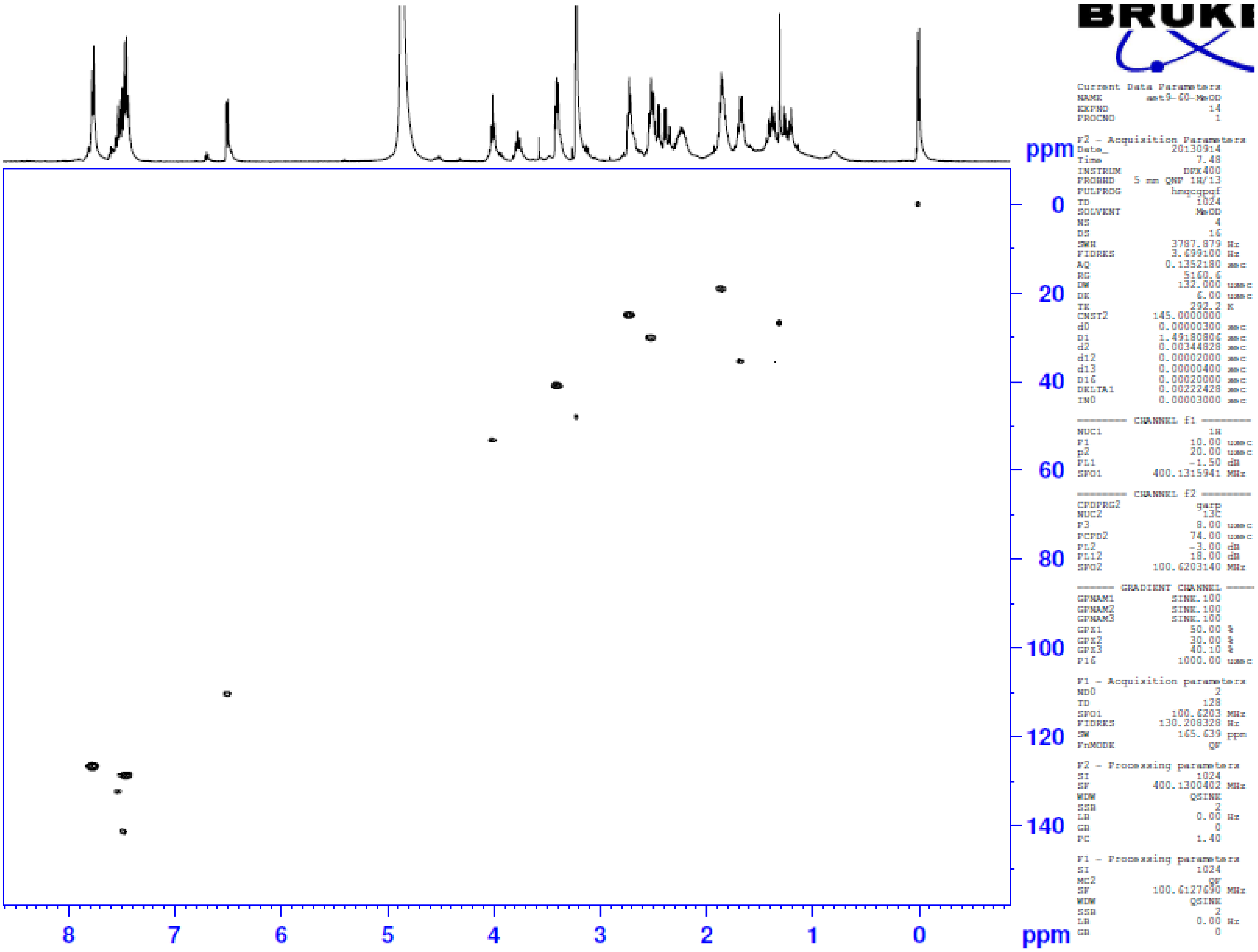

ppm
0
20
40
60
80
100
120
140
8
7
6
5
4
3
2
1
0
ppm
Current Data Parameters
EXPNO 14
PROCNO 1
F2 - Acquisition Parameters
Date_ 20130914
Time 7.48
INSTRUM DPX400
PROBHD 5 mm QNP 1H/13
PULPROG hmqcgpqf
TD 1024
SOLVENT MeOD
NS 4
DS 16
SWH 3787.879 Hz
FIDRES 3.699100 Hz
AQ 0.1352180 sec
RG 5160.6
DW 132.000 usec
DE 6.00 usec
TE 292.2 K
CNST2 145.0000000
d0 0.00000300 sec
D1 1.49180806 sec
d2 0.00344828 sec
d12 0.00002000 sec
d13 0.00000400 sec
D16 0.00020000 sec
DELTA1 0.00222428 sec
IN0 0.00003000 sec
CHANNEL f1
NUC1 1H
P1 10.00 usec
p2 20.00 usec
PL1 -1.50 dB
SFO1 400.1315941 MHz
CHANNEL f2
CPDPRG2 garp
NUC2 13C
P3 8.00 usec
PCPD2 74.00 usec
PL2 -3.00 dB
PL12 18.00 dB
SFO2 100.6203140 MHz
GRADIENT CHANNEL
GPNAM1 SINE.100
GPNAM2 SINE.100
GPNAM3 SINE.100
GPZ1 50.00 %
GPZ2 30.00 %
GPZ3 40.10 %
P16 1000.00 usec
F1 - Acquisition parameters
ND0 2
TD 128
SFO1 100.6203 MHz
FIDRES 130.208328 Hz
SW 165.639 ppm
FnMODE QF
F2 - Processing parameters
SI 1024
SF 400.1300402 MHz
WDW QSINE
SSB 2
LB 0.00 Hz
GB 0
PC 1.40
F1 - Processing parameters
SI 1024
MC2 QF
SF 100.6127690 MHz
WDW QSINE
SSB 2
LB 0.00 Hz
GB 0

***tert*-butyl (6-(4-hydroxy-but-1-yn-1-yl)-pyridin-2-yl)(methyl)carbamate [Compound 46]**

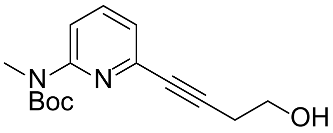

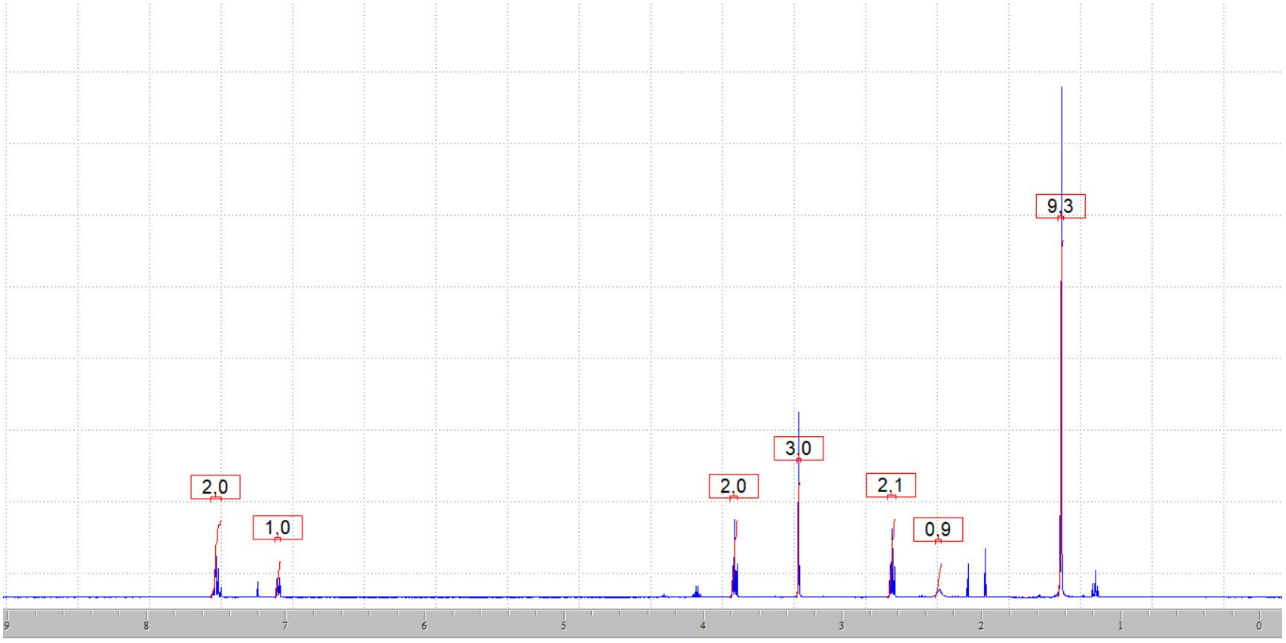

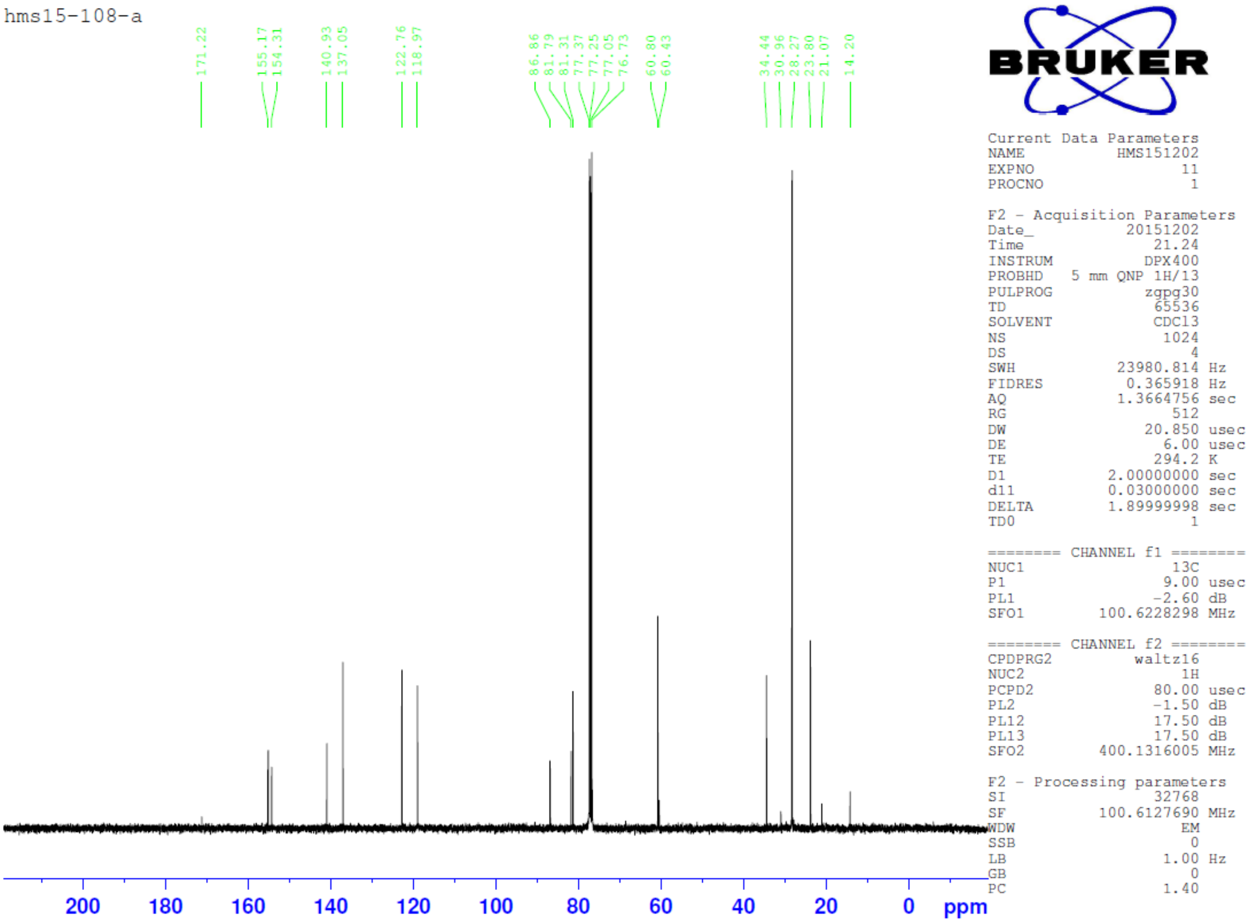

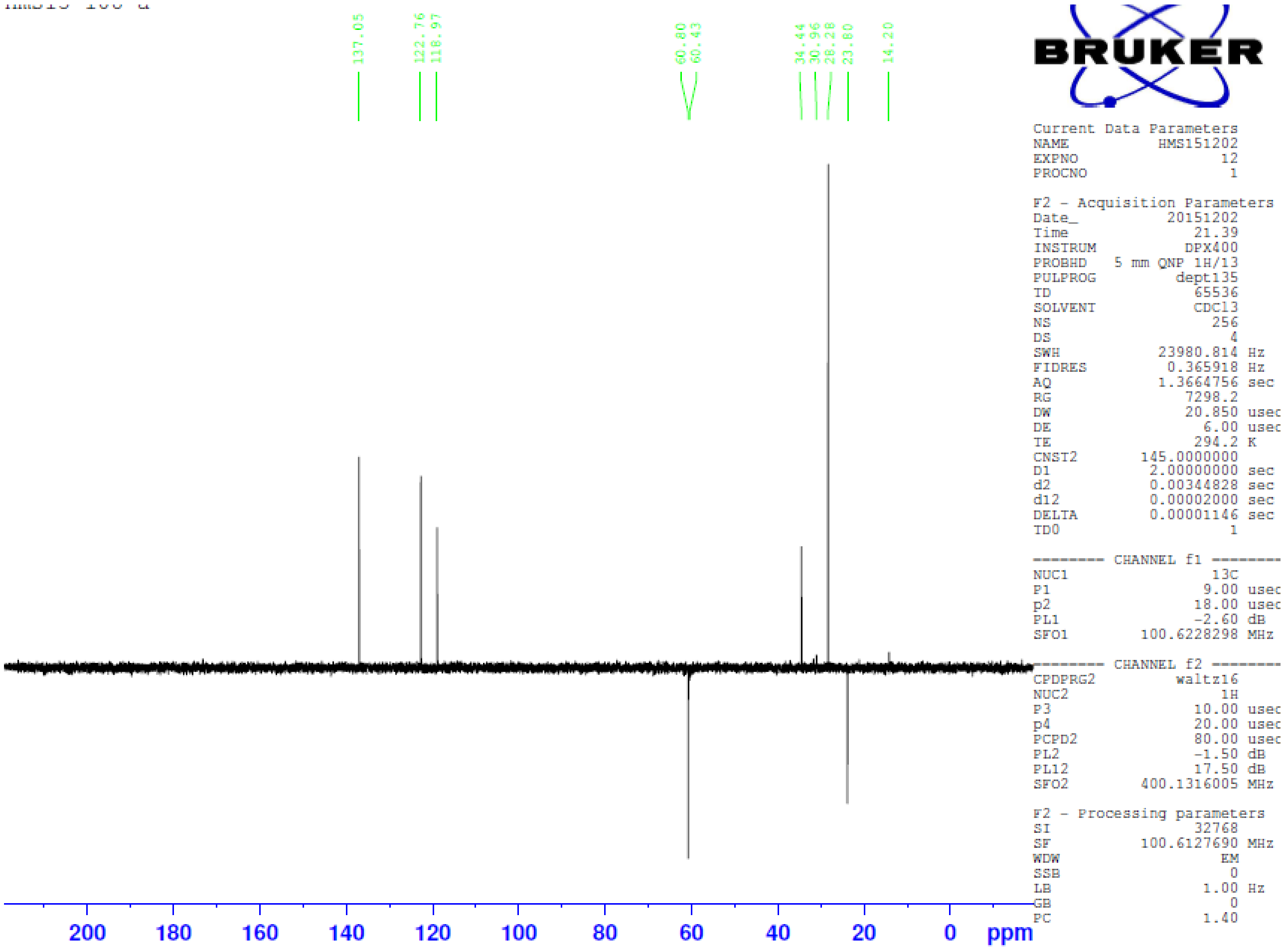
137.05
122.76
118.97
60.80
60.43
34.44
30.96
28.28
23.80
14.20
200 180 160 140 120 100 80 60 40 20 0 ppm
BRUKER
Current Data Parameters
NAME HMS151202
EXPNO 12
PROCNO 1
F2 - Acquisition Parameters
Date_ 20151202
Time 21.39
INSTRUM DPX400
PROBHD 5 mm QNP 1H/13
PULPROG dept135
TD 65536
SOLVENT CDCl3
NS 256
DS 4
SWH 23980.814 Hz
FIDRES 0.365918 Hz
AQ 1.3664756 sec
RG 7298.2
DW 20.850 usec
DE 6.00 usec
TE 294.2 K
CNST2 145.0000000
D1 2.00000000 sec
d2 0.00344828 sec
d12 0.00002000 sec
DELTA 0.00001146 sec
TD0 1
======== CHANNEL f1 ========
NUC1 13C
P1 9.00 usec
p2 18.00 usec
PL1 -2.60 dB
SFO1 100.6228298 MHz
======== CHANNEL f2 ========
CPDPRG2 waltz16
NUC2 1H
P3 10.00 usec
p4 20.00 usec
PCPD2 80.00 usec
PL2 -1.50 dB
PL12 17.50 dB
SFO2 400.1316005 MHz
F2 - Processing parameters
SI 32768
SF 100.6127690 MHz
WDW EM
SSB 0
LB 1.00 Hz
GB 0
PC 1.40

***tert*-butyl methyl(6-(4-oxobutyl)pyridin-2-yl)carbamate [Compound 43]**

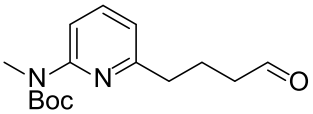

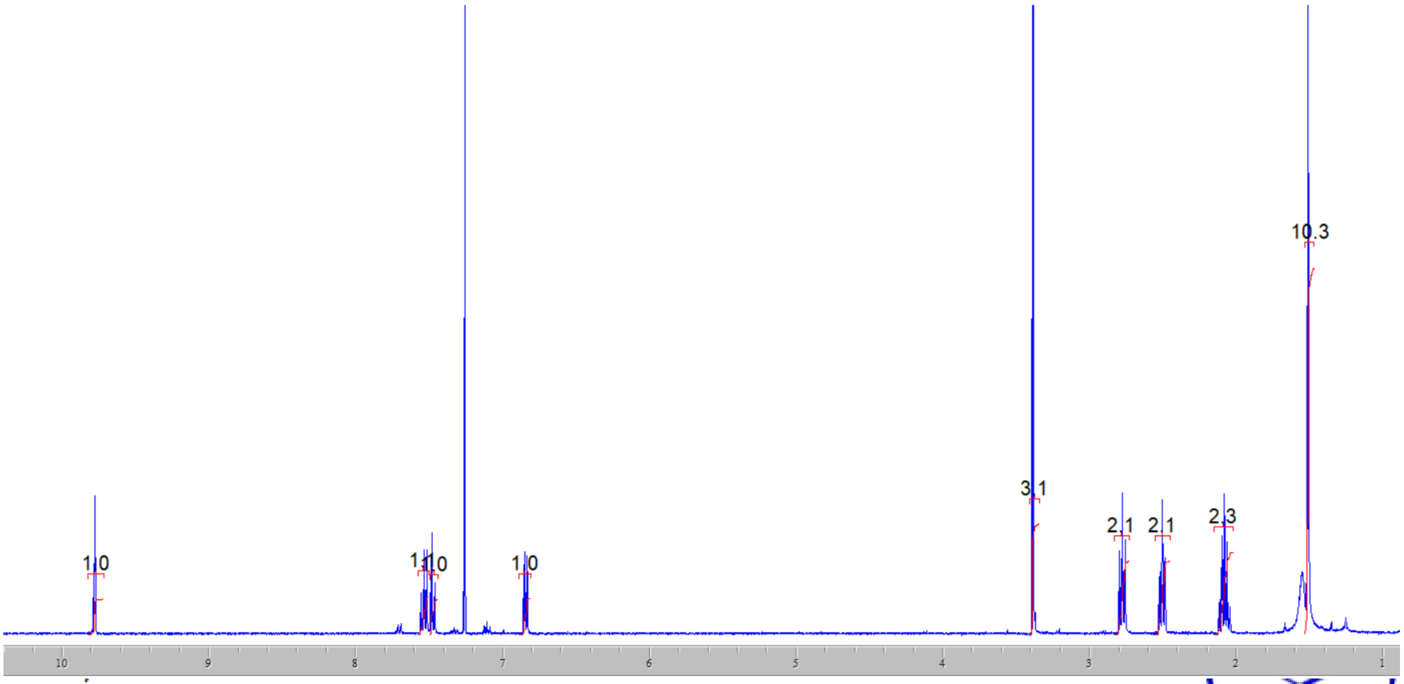

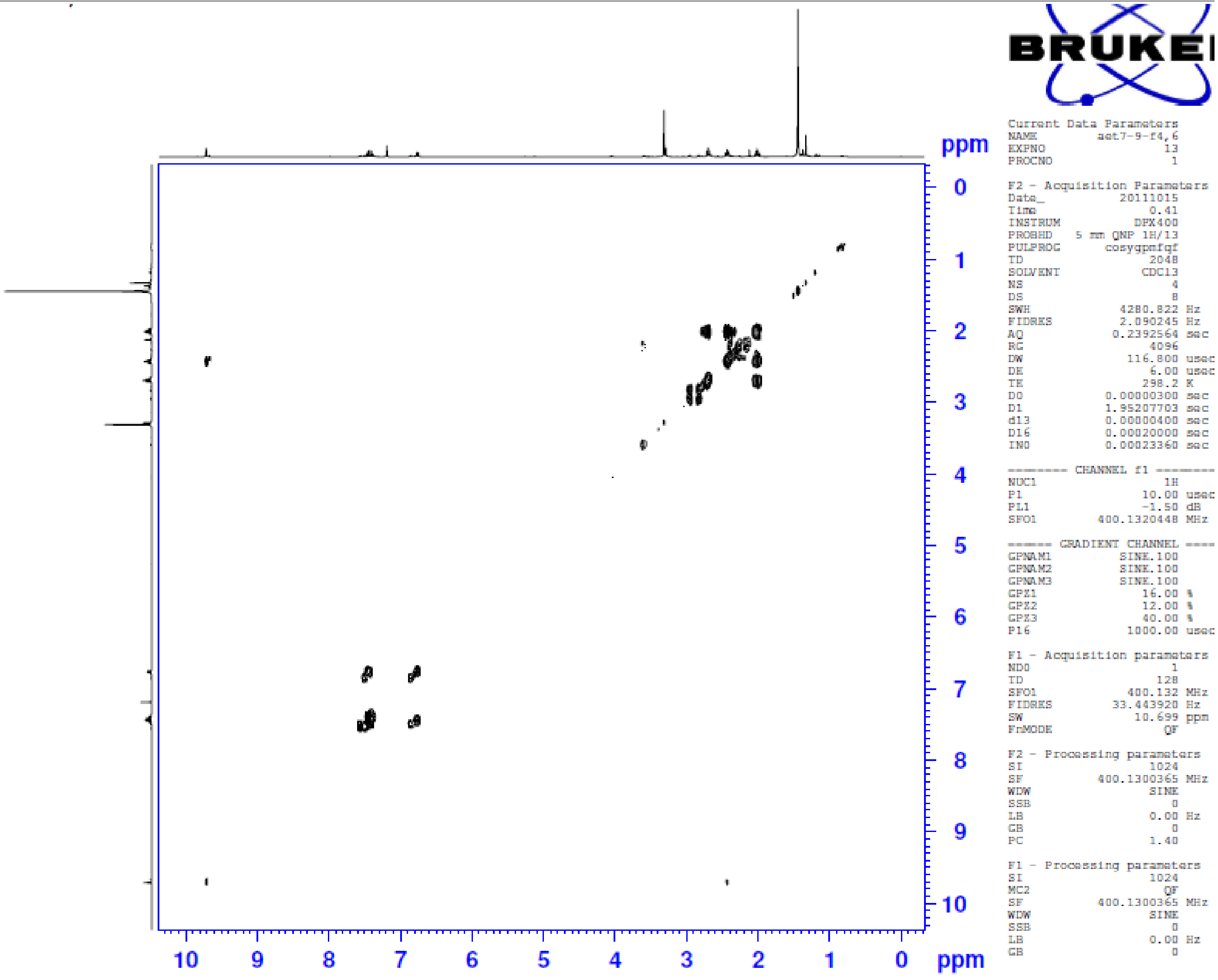

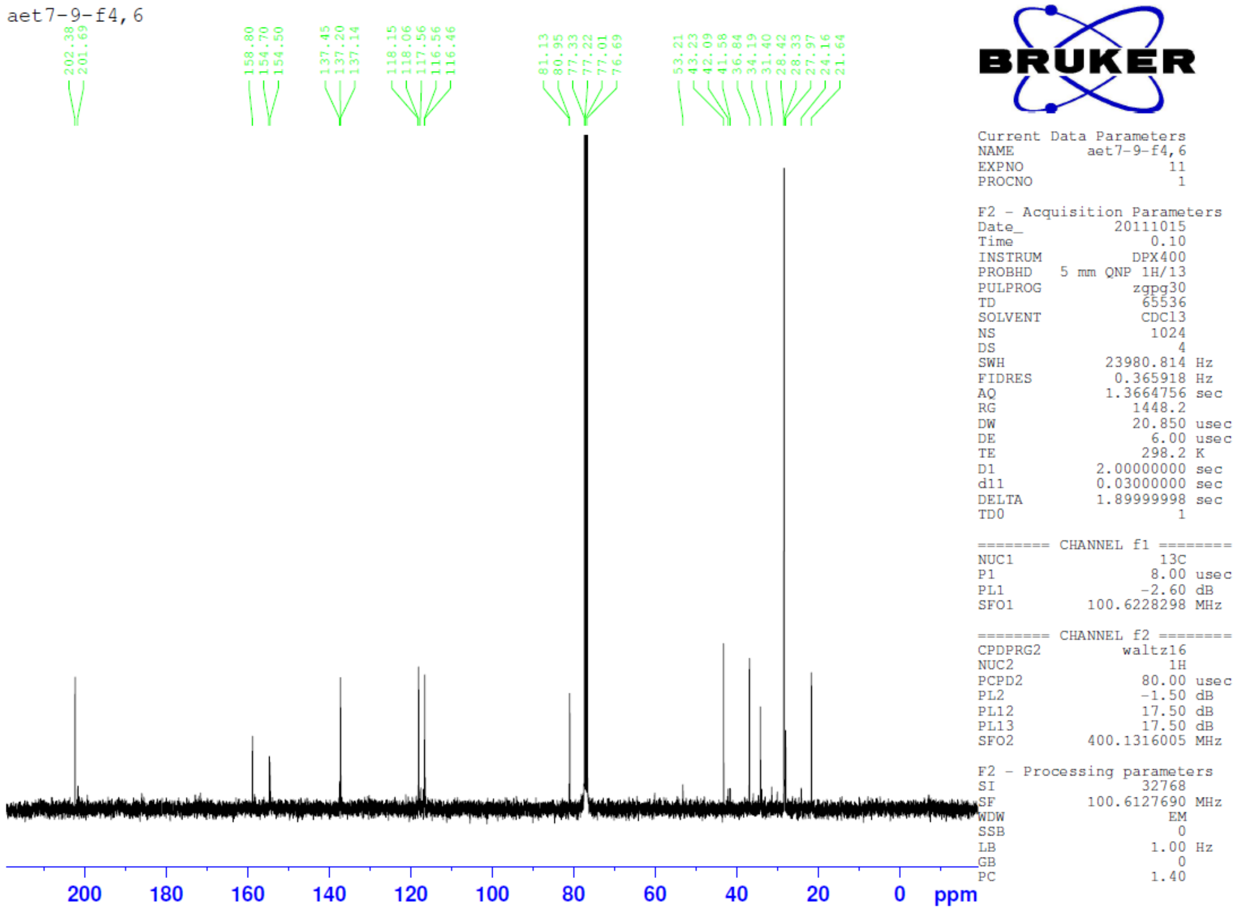
aet7-9-f4,6
202.38
201.69
158.80
154.70
154.50
137.45
137.20
137.14
118.15
118.06
117.56
116.56
116.46
81.13
80.95
77.33
77.22
77.01
76.69
53.21
43.23
42.09
41.58
36.84
34.19
31.40
28.42
28.33
27.97
24.16
21.64
BRUKER
Current Data Parameters
NAME aet7-9-f4,6
EXPNO 11
PROCNO 1
F2 - Acquisition Parameters
Date_ 20111015
Time 0.10
INSTRUM DPX400
PROBHD 5 mm QNP 1H/13
PULPROG zgpg30
TD 65536
SOLVENT CDCl3
NS 1024
DS 4
SWH 23980.814 Hz
FIDRES 0.365918 Hz
AQ 1.3664756 sec
RG 1448.2
DW 20.850 usec
DE 6.00 usec
TE 298.2 K
D1 2.00000000 sec
d11 0.03000000 sec
DELTA 1.89999998 sec
TD0 1
======== CHANNEL f1 ========
NUC1 13C
P1 8.00 usec
PL1 -2.60 dB
SFO1 100.6228298 MHz
======== CHANNEL f2 ========
CPDPRG2 waltz16
NUC2 1H
PCPD2 80.00 usec
PL2 -1.50 dB
PL12 17.50 dB
PL13 17.50 dB
SFO2 400.1316005 MHz
F2 - Processing parameters
SI 32768
SF 100.6127690 MHz
WDW EM
SSB 0
LB 1.00 Hz
GB 0
PC 1.40
200 180 160 140 120 100 80 60 40 20 0 ppm

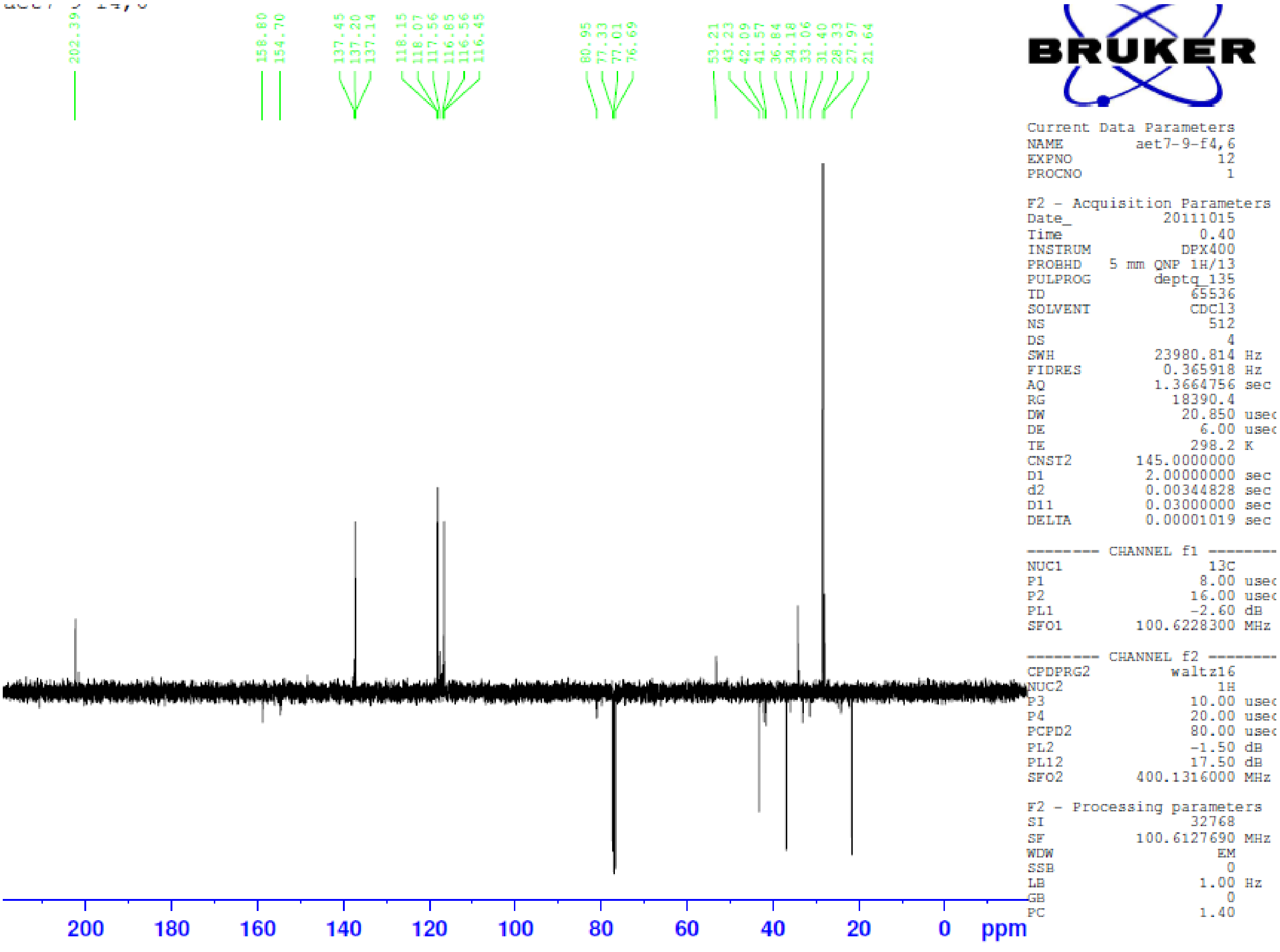
202.39
158.80
154.70
137.45
137.20
137.14
118.15
118.07
117.56
116.85
116.56
116.45
80.95
77.33
77.01
76.69
53.21
43.23
42.09
41.57
36.84
34.18
33.06
31.40
28.33
27.97
21.64
BRUKER
Current Data Parameters
NAME aet7-9-f4,6
EXPNO 12
PROCNO 1
F2 - Acquisition Parameters
Date_ 20111015
Time 0.40
INSTRUM DPX400
PROBHD 5 mm QNP 1H/13
PULPROG deptq_135
TD 65536
SOLVENT CDCl3
NS 512
DS 4
SWH 23980.814 Hz
FIDRES 0.365918 Hz
AQ 1.3664756 sec
RG 18390.4
DW 20.850 usec
DE 6.00 usec
TE 298.2 K
CNST2 145.0000000
D1 2.00000000 sec
d2 0.00344828 sec
D11 0.03000000 sec
DELTA 0.00001019 sec
======== CHANNEL f1 ========
NUC1 13C
P1 8.00 usec
P2 16.00 usec
PL1 -2.60 dB
SFO1 100.6228300 MHz
======== CHANNEL f2 ========
CPDPRG2 waltz16
NUC2 1H
P3 10.00 usec
P4 20.00 usec
PCPD2 80.00 usec
PL2 -1.50 dB
PL12 17.50 dB
SFO2 400.1316000 MHz
F2 - Processing parameters
SI 32768
SF 100.6127690 MHz
WDW EM
SSB 0
LB 1.00 Hz
GB 0
PC 1.40
200 180 160 140 120 100 80 60 40 20 0 ppm

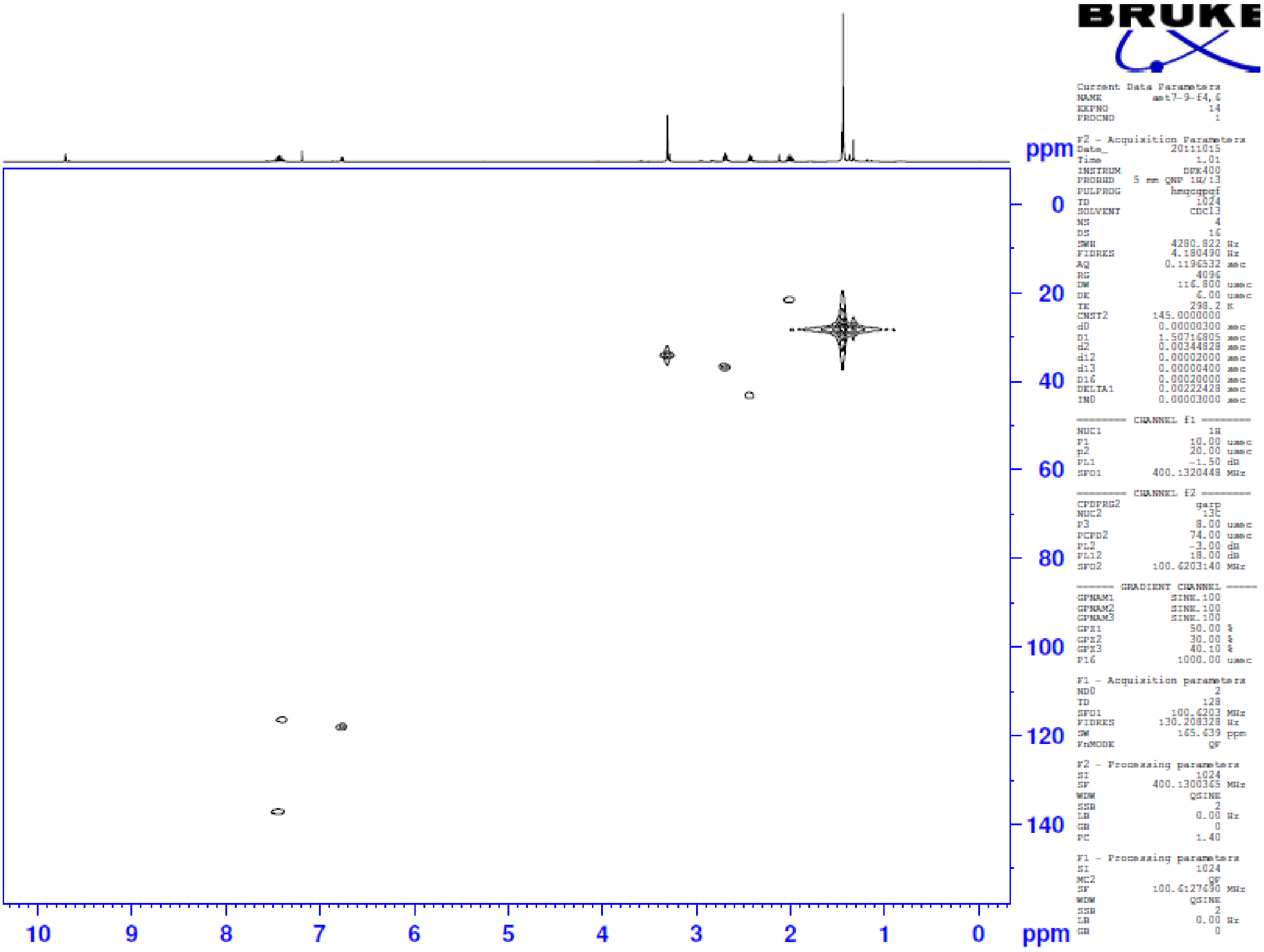
ppm
0
20
40
60
80
100
120
140
10
9
8
7
6
5
4
3
2
1
0
ppm
Current Data Parameters
NAME amt7-9-f4,6
EXPNO 14
PROCNO 1
F2 - Acquisition Parameters
Date_ 20111015
Time 1.01
INSTRUM DPX400
PROBHD 5 mm QNP 1H/13
PULPROG hmqcgpqf
TD 1024
SOLVENT CDCl3
NS 4
DS 16
SWH 4280.822 Hz
FIDRES 4.180490 Hz
AQ 0.1196532 sec
RG 4096
DW 116.800 usec
DE 6.00 usec
TE 298.2 K
CNST2 145.0000000
d0 0.00000300 sec
D1 1.50716805 sec
d2 0.00344828 sec
d12 0.00002000 sec
d13 0.00000400 sec
D16 0.00020000 sec
DELTA1 0.00222428 sec
IN0 0.00003000 sec
======== CHANNEL f1 ========
NUC1 1H
P1 10.00 usec
p2 20.00 usec
PL1 -1.50 dB
SFO1 400.1320448 MHz
======== CHANNEL f2 ========
CPDPRG2 garp
NUC2 13C
P3 8.00 usec
PCPD2 74.00 usec
PL2 -3.00 dB
PL12 18.00 dB
SFO2 100.6203140 MHz
====== GRADIENT CHANNEL ======
GPNAM1 SINE.100
GPNAM2 SINE.100
GPNAM3 SINE.100
GPZ1 50.00 %
GPZ2 30.00 %
GPZ3 40.10 %
P16 1000.00 usec
F1 - Acquisition parameters
ND0 2
TD 128
SFO1 100.6203 MHz
FIDRES 130.208328 Hz
SW 165.639 ppm
FnMODE QF
F2 - Processing parameters
SI 1024
SF 400.1300365 MHz
WDW QSINE
SSB 2
LB 0.00 Hz
GB 0
PC 1.40
F1 - Processing parameters
SI 1024
MC2 QF
SF 100.6127690 MHz
WDW QSINE
SSB 2
LB 0.00 Hz
GB 0

Methyl 3-(2-(6-((*tert*-butoxycarbonyl)(methyl)amino)pyridin-2-yl)ethyl)cyclobut-1-ene-1-carboxylate

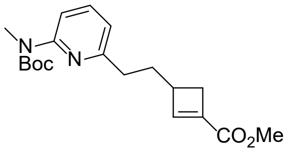

[Compound 47]

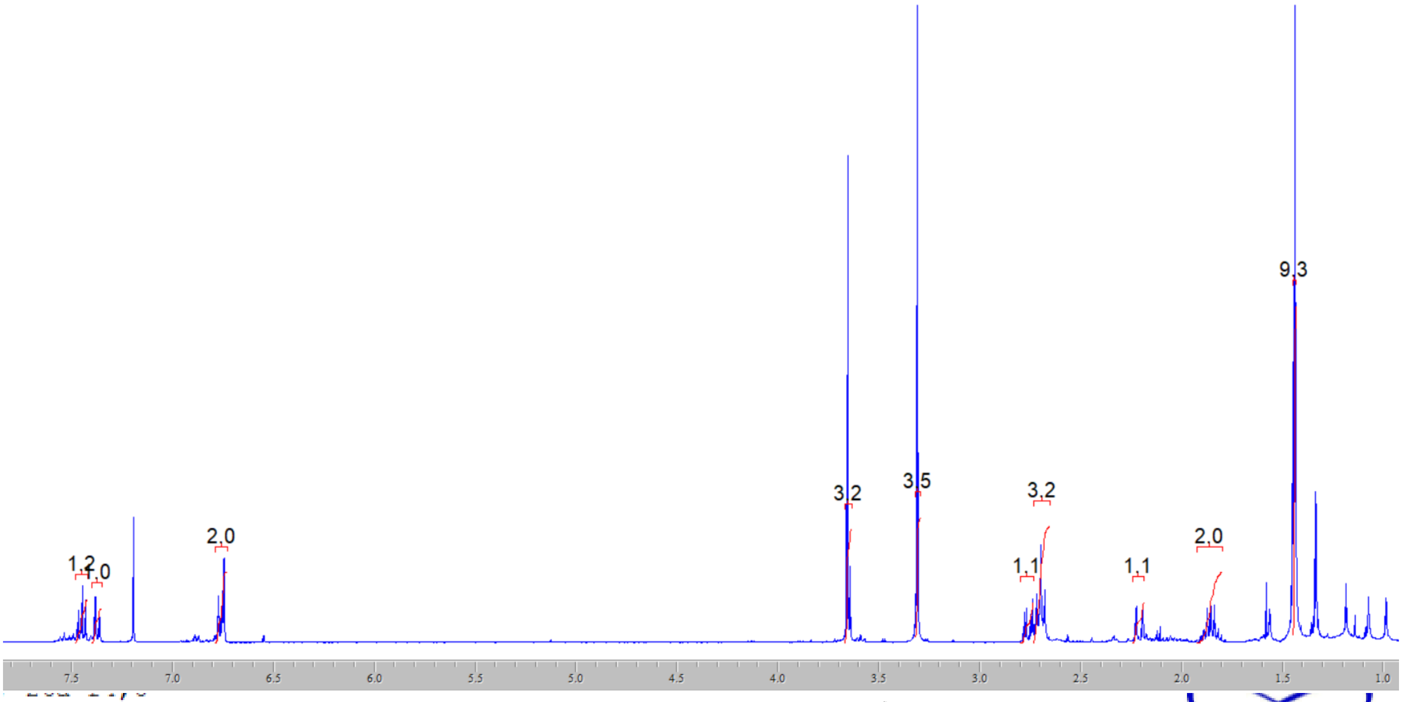

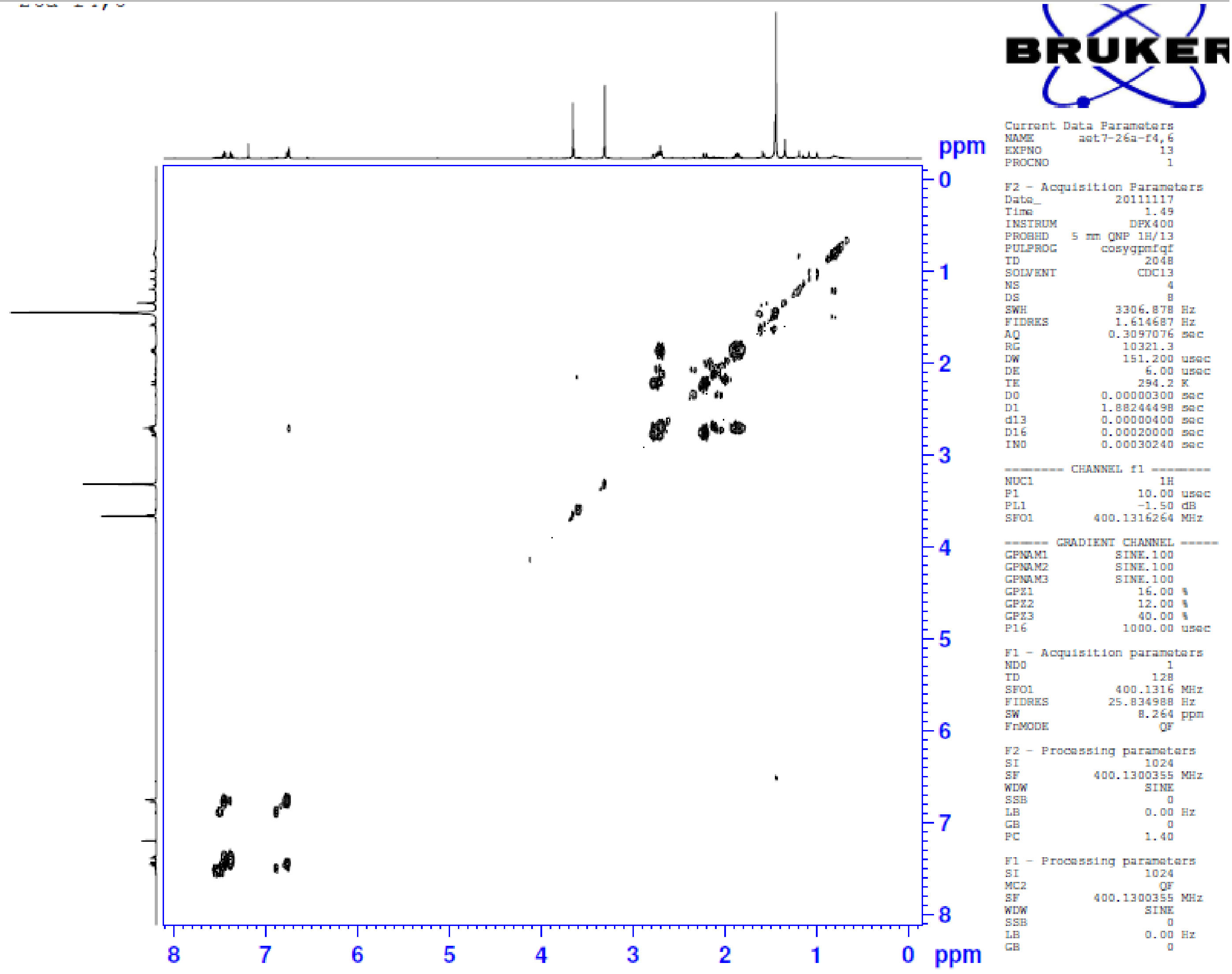

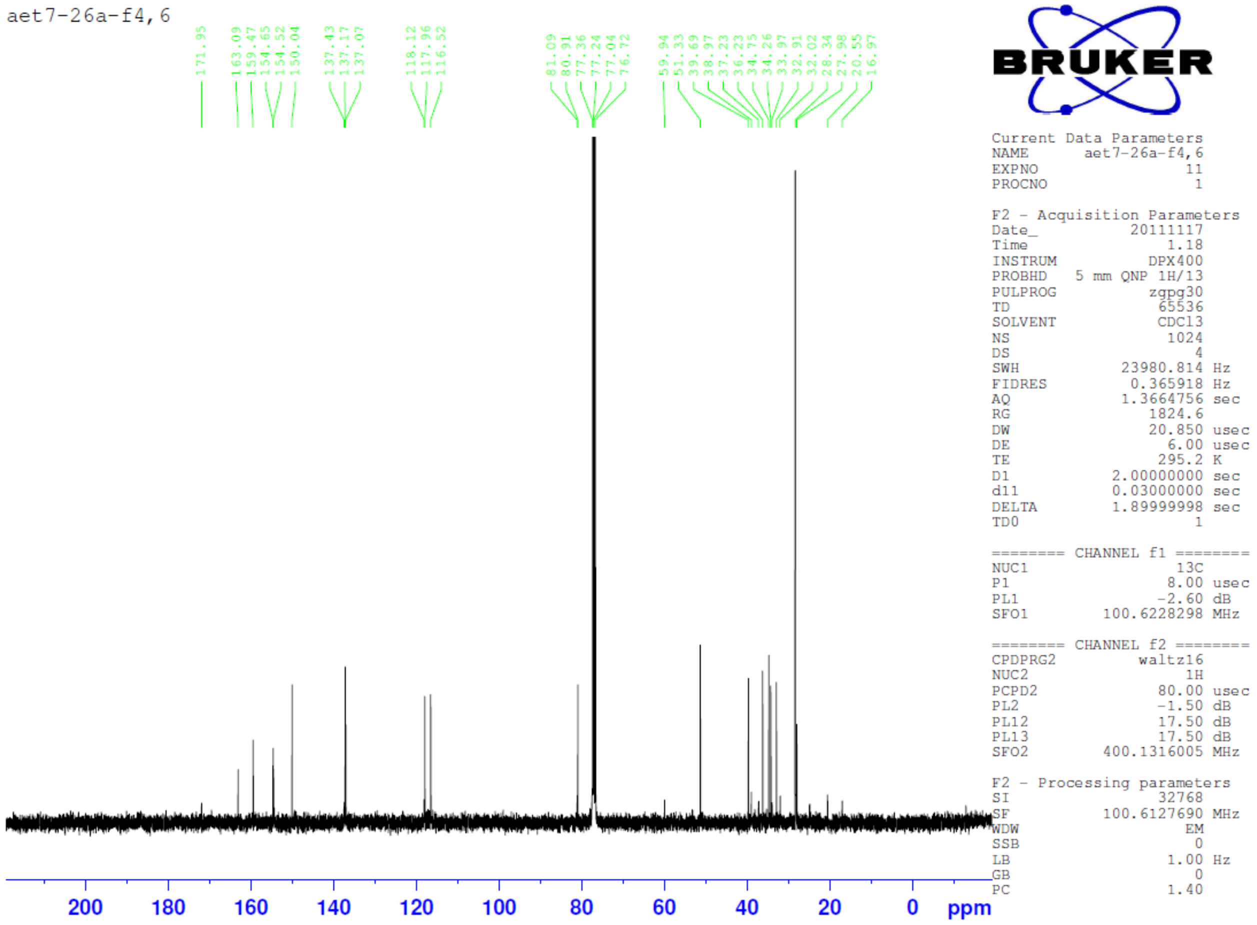
aet7-26a-f4,6
171.95
163.09
159.47
154.65
154.52
150.04
137.43
137.17
137.07
118.12
117.96
116.52
81.09
80.91
77.36
77.24
77.04
76.72
59.94
51.33
39.69
38.97
37.23
36.23
34.75
34.26
33.97
32.91
32.02
28.34
27.98
20.55
16.97
BRUKER
Current Data Parameters
NAME aet7-26a-f4,6
EXPNO 11
PROCNO 1
F2 - Acquisition Parameters
Date_ 20111117
Time 1.18
INSTRUM DPX400
PROBHD 5 mm QNP 1H/13
PULPROG zgpg30
TD 65536
SOLVENT CDCl3
NS 1024
DS 4
SWH 23980.814 Hz
FIDRES 0.365918 Hz
AQ 1.3664756 sec
RG 1824.6
DW 20.850 usec
DE 6.00 usec
TE 295.2 K
D1 2.00000000 sec
d11 0.03000000 sec
DELTA 1.89999998 sec
TD0 1
======== CHANNEL f1 ========
NUC1 13C
P1 8.00 usec
PL1 -2.60 dB
SFO1 100.6228298 MHz
======== CHANNEL f2 ========
CPDPRG2 waltz16
NUC2 1H
PCPD2 80.00 usec
PL2 -1.50 dB
PL12 17.50 dB
PL13 17.50 dB
SFO2 400.1316005 MHz
F2 - Processing parameters
SI 32768
SF 100.6127690 MHz
WDW EM
SSB 0
LB 1.00 Hz
GB 0
PC 1.40
200 180 160 140 120 100 80 60 40 20 0 ppm

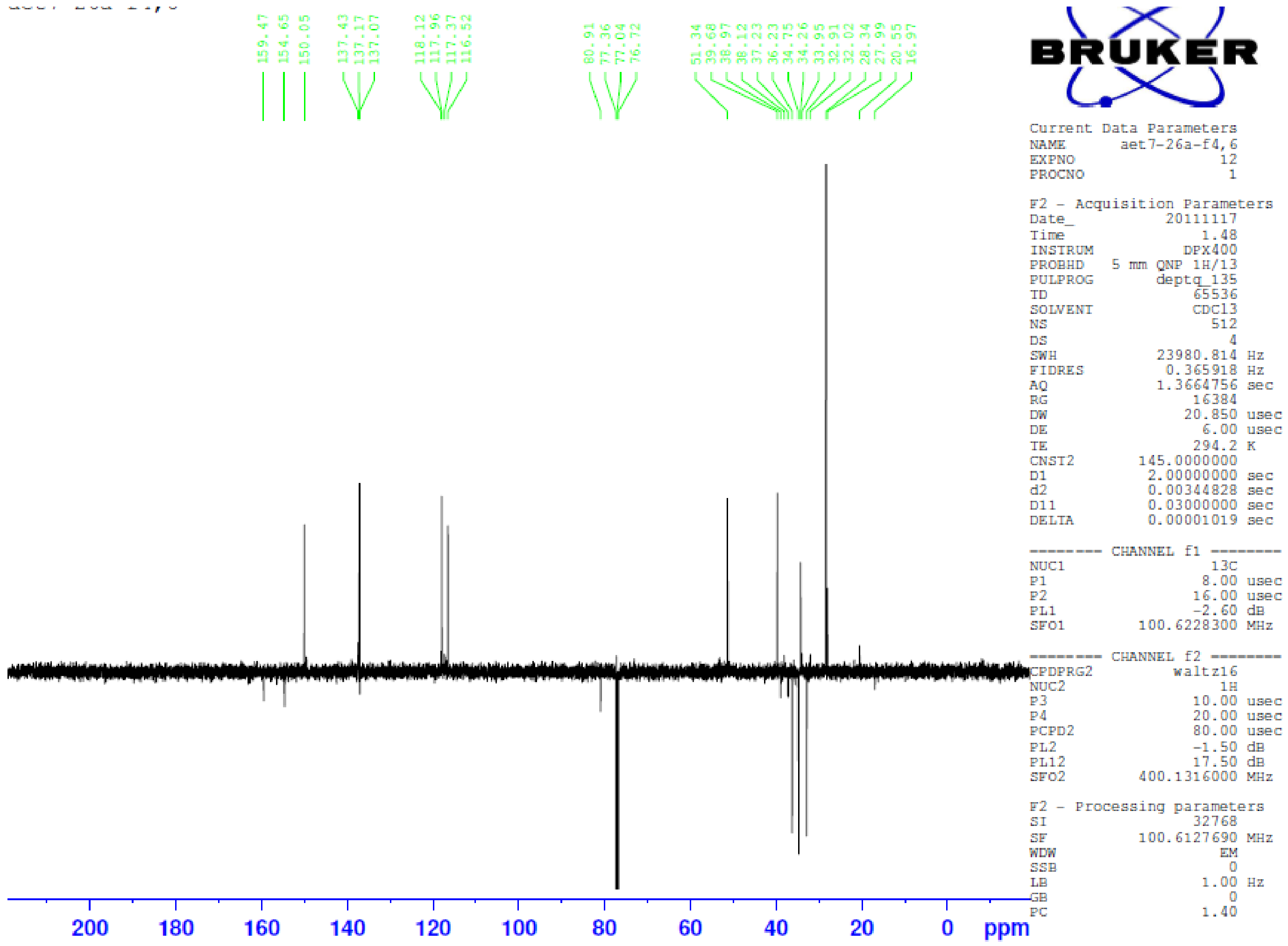
159.47
154.65
150.05
137.43
137.17
137.07
118.12
117.96
117.37
116.52
80.91
77.36
77.04
76.72
51.34
39.68
38.97
38.12
37.23
36.23
34.75
34.26
33.95
32.91
32.02
28.34
27.99
20.55
16.97
BRUKER
Current Data Parameters
NAME aet7-26a-f4,6
EXPNO 12
PROCNO 1
F2 - Acquisition Parameters
Date_ 20111117
Time 1.48
INSTRUM DPX400
PROBHD 5 mm QNP 1H/13
PULPROG deptq_135
TD 65536
SOLVENT CDCl3
NS 512
DS 4
SWH 23980.814 Hz
FIDRES 0.365918 Hz
AQ 1.3664756 sec
RG 16384
DW 20.850 usec
DE 6.00 usec
TE 294.2 K
CNST2 145.0000000
D1 2.00000000 sec
d2 0.00344828 sec
D11 0.03000000 sec
DELTA 0.00001019 sec
======== CHANNEL f1 ========
NUC1 13C
P1 8.00 usec
P2 16.00 usec
PL1 -2.60 dB
SFO1 100.6228300 MHz
======== CHANNEL f2 ========
CPDPRG2 waltz16
NUC2 1H
P3 10.00 usec
P4 20.00 usec
PCPD2 80.00 usec
PL2 -1.50 dB
PL12 17.50 dB
SFO2 400.1316000 MHz
F2 - Processing parameters
SI 32768
SF 100.6127690 MHz
WDW EM
SSB 0
LB 1.00 Hz
GB 0
PC 1.40
200 180 160 140 120 100 80 60 40 20 0 ppm

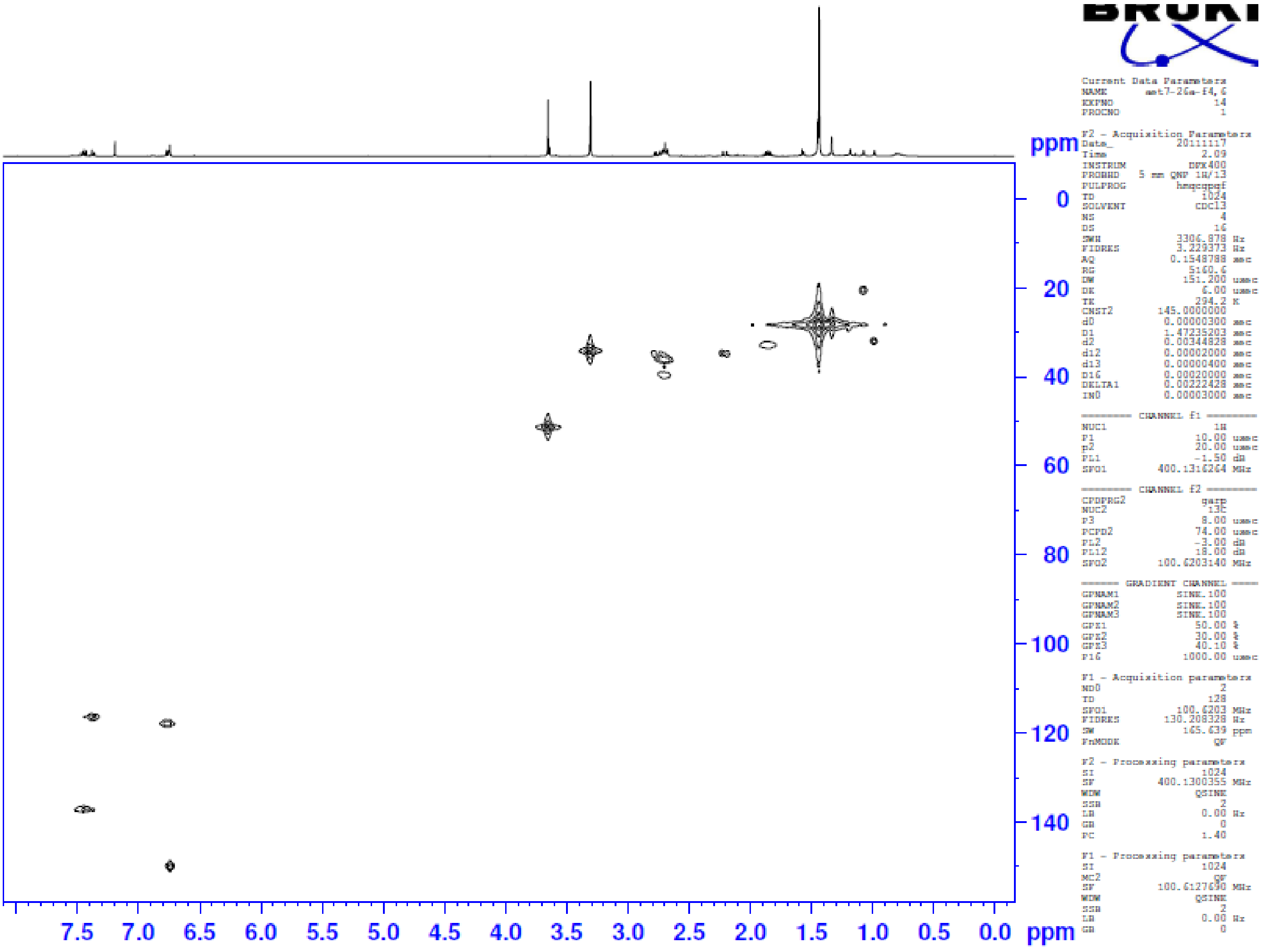
Current Data Parameters
NAME amt7-26a-f4,6
EXPNO 14
PROCNO 1
F2 - Acquisition Parameters
Date_ 20111117
Time 2.09
INSTRUM DPX400
PROBHD 5 mm QNP 1H/13
PULPROG hmqcgpqf
TD 1024
SOLVENT CDCl3
NS 4
DS 16
SWH 3306.878 Hz
FIDRES 3.229373 Hz
AQ 0.1548788 sec
RG 5160.6
DW 151.200 usec
DE 6.00 usec
TE 294.2 K
CNST2 145.0000000
d0 0.00000300 sec
D1 1.47235203 sec
d2 0.00344828 sec
d12 0.00002000 sec
d13 0.00000400 sec
D16 0.00020000 sec
DELTA1 0.00222428 sec
IN0 0.00003000 sec
CHANNEL f1
NUC1 1H
P1 10.00 usec
p2 20.00 usec
PL1 -1.50 dB
SFO1 400.1316264 MHz
CHANNEL f2
CPDPRG2 garp
NUC2 13C
P3 8.00 usec
PCPD2 74.00 usec
PL2 -3.00 dB
PL12 18.00 dB
SFO2 100.6203140 MHz
GRADIENT CHANNEL
GPNAM1 SINE.100
GPNAM2 SINE.100
GPNAM3 SINE.100
GPZ1 50.00 %
GPZ2 30.00 %
GPZ3 40.10 %
P16 1000.00 usec
F1 - Acquisition parameters
ND0 2
TD 128
SFO1 100.6203 MHz
FIDRES 130.208328 Hz
SW 165.639 ppm
FnMODE QF
F2 - Processing parameters
SI 1024
SF 400.1300355 MHz
WDW QSINE
SSB 2
LB 0.00 Hz
GB 0
PC 1.40
F1 - Processing parameters
SI 1024
MC2 QF
SF 100.6127690 MHz
WDW QSINE
SSB 2
LB 0.00 Hz
GB 0
ppm
0
20
40
60
80
100
120
140
7.5 7.0 6.5 6.0 5.5 5.0 4.5 4.0 3.5 3.0 2.5 2.0 1.5 1.0 0.5 0.0 ppm

Methyl (1*r,3*s**)-3-(2-(6-((tert-butoxycarbonyl)(methyl)amino)pyridin-2-yl)ethyl) cyclobutane-1-carboxylate [Compound 48]**

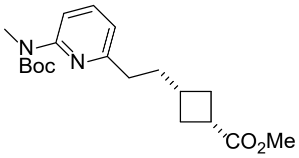

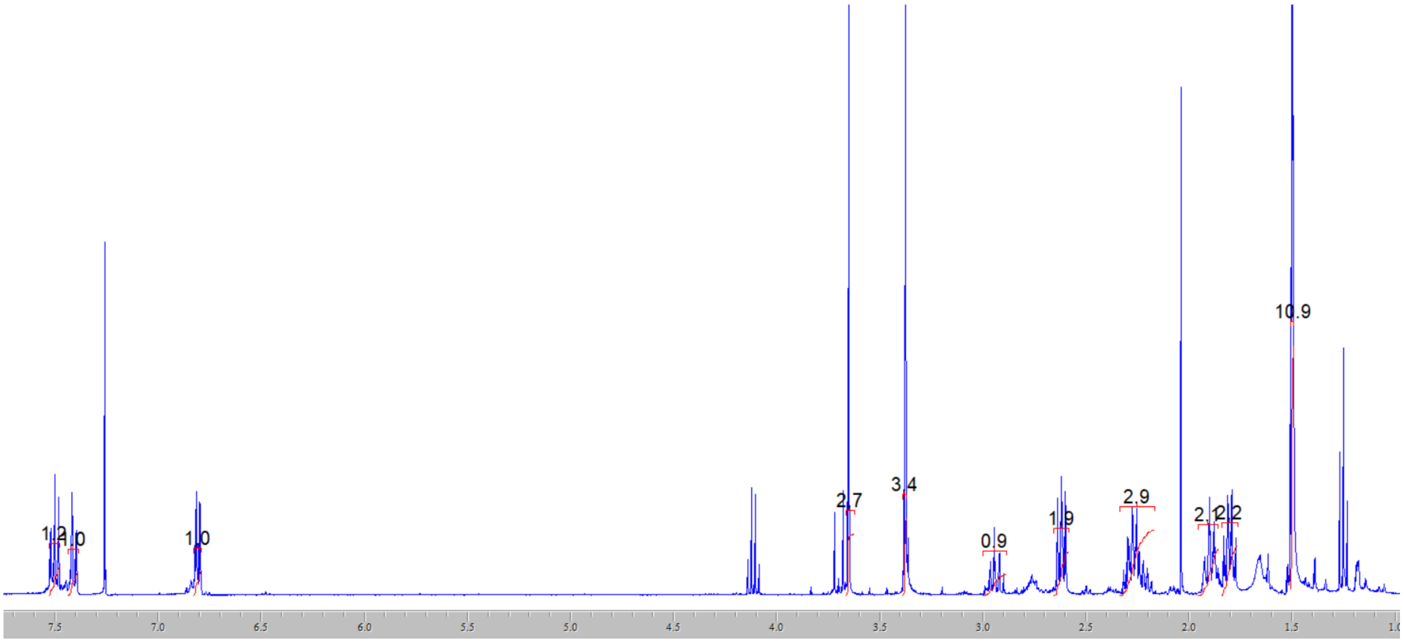

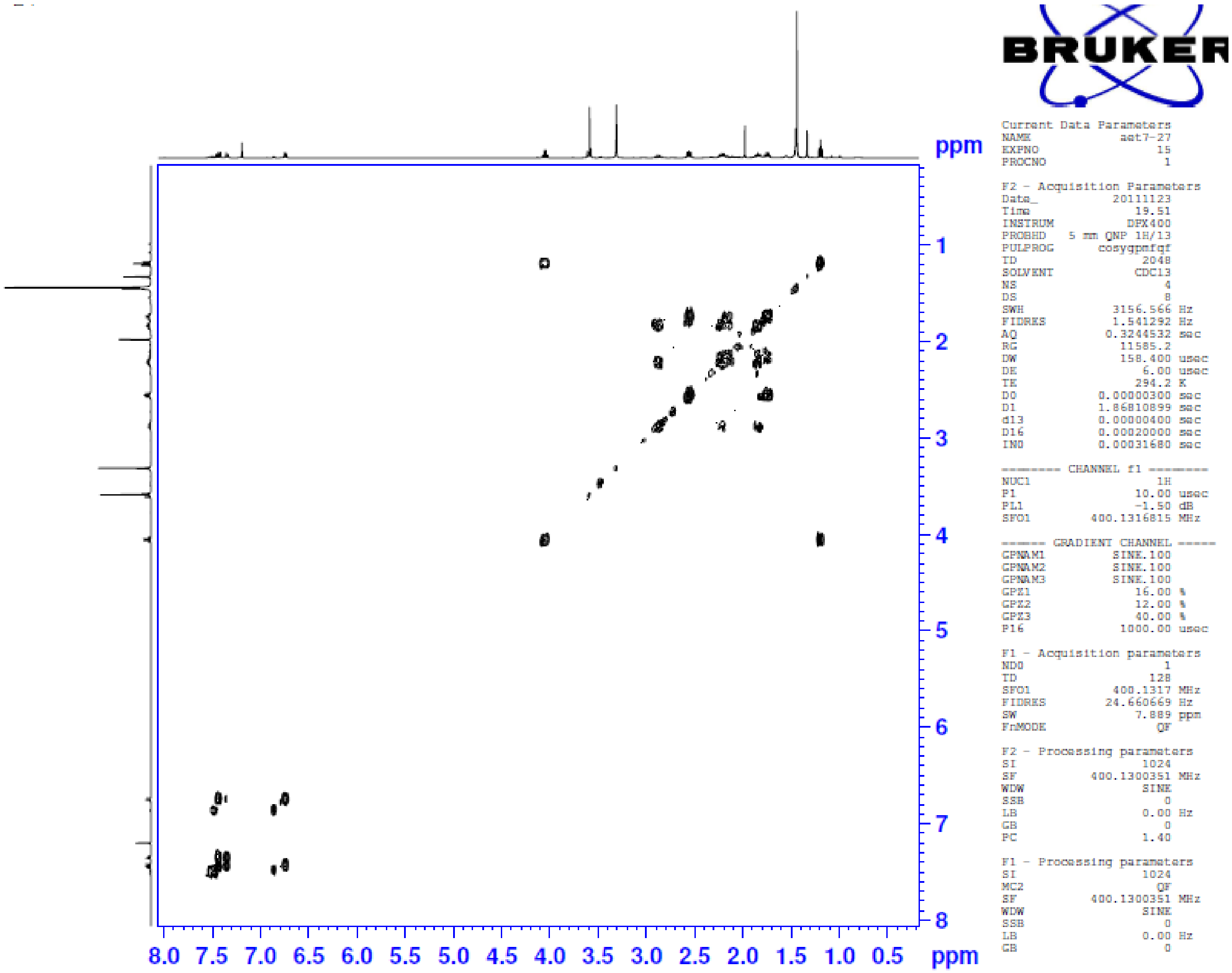

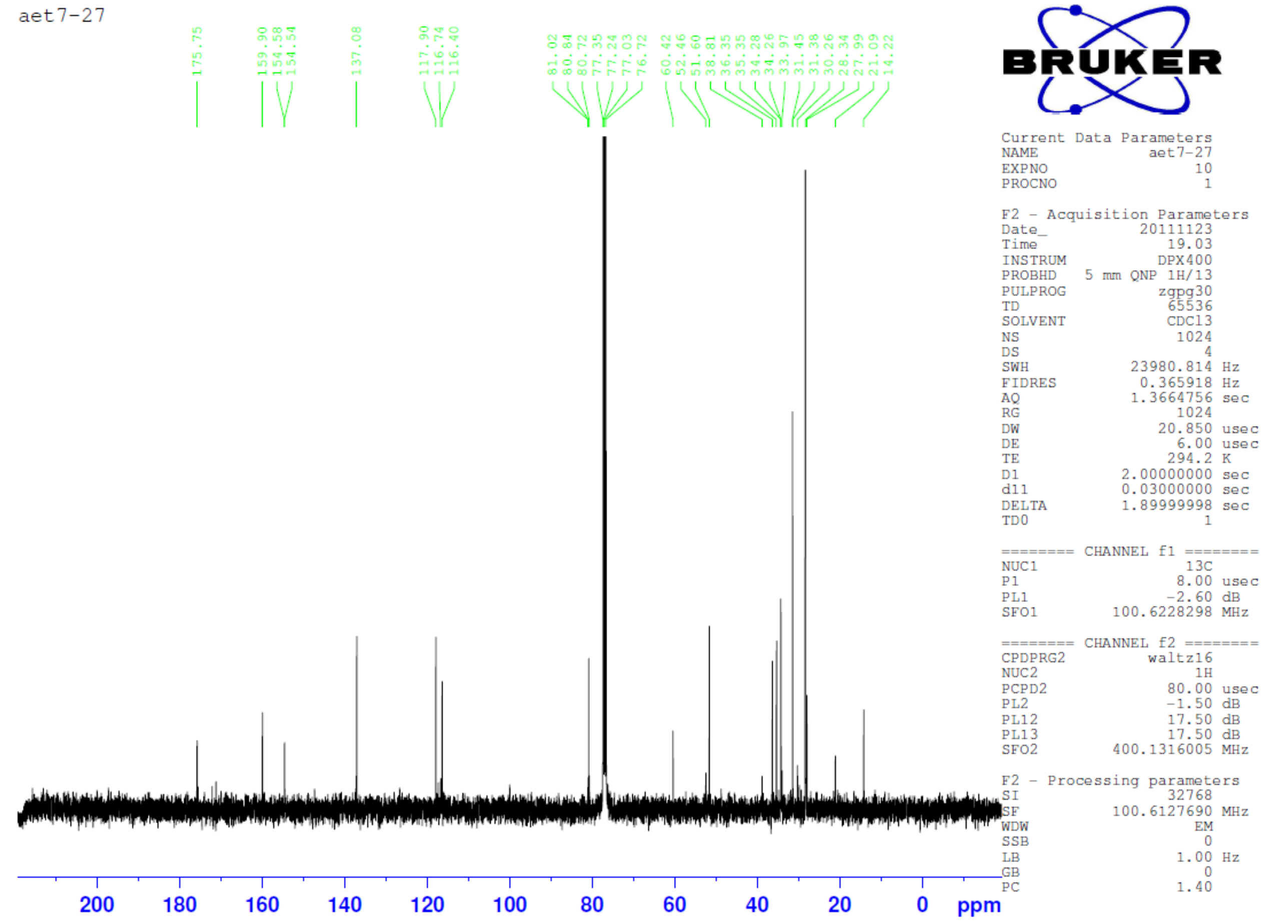
aet7-27
175.75
159.90
154.58
154.54
137.08
117.90
116.74
116.40
81.02
80.84
80.72
77.35
77.24
77.03
76.72
60.42
52.46
51.60
38.81
36.35
35.35
34.28
34.26
33.97
31.45
31.38
30.26
28.34
27.99
21.09
14.22
BRUKER
Current Data Parameters
NAME aet7-27
EXPNO 10
PROCNO 1
F2 - Acquisition Parameters
Date_ 20111123
Time 19.03
INSTRUM DPX400
PROBHD 5 mm QNP 1H/13
PULPROG zgpg30
TD 65536
SOLVENT CDCl3
NS 1024
DS 4
SWH 23980.814 Hz
FIDRES 0.365918 Hz
AQ 1.3664756 sec
RG 1024
DW 20.850 usec
DE 6.00 usec
TE 294.2 K
D1 2.00000000 sec
d11 0.03000000 sec
DELTA 1.89999998 sec
TD0 1
======== CHANNEL f1 ========
NUC1 13C
P1 8.00 usec
PL1 -2.60 dB
SFO1 100.6228298 MHz
======== CHANNEL f2 ========
CPDPRG2 waltz16
NUC2 1H
PCPD2 80.00 usec
PL2 -1.50 dB
PL12 17.50 dB
PL13 17.50 dB
SFO2 400.1316005 MHz
F2 - Processing parameters
SI 32768
SF 100.6127690 MHz
WDW EM
SSB 0
LB 1.00 Hz
GB 0
PC 1.40
200
180
160
140
120
100
80
60
40
20
0
ppm

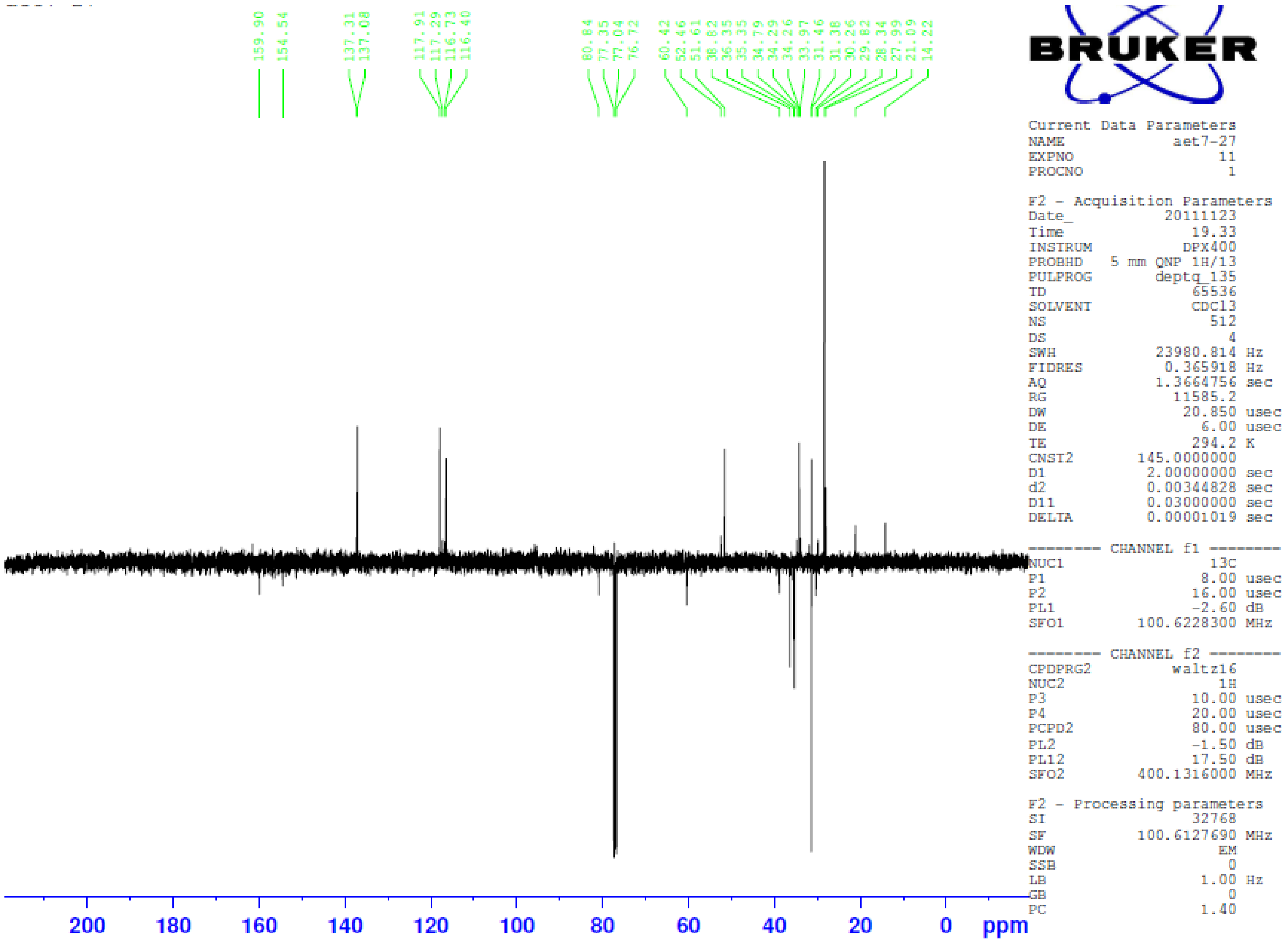
159.90
154.54
137.31
137.08
117.91
117.29
116.73
116.40
80.84
77.35
77.04
76.72
60.42
52.46
51.61
38.82
36.35
35.35
34.79
34.29
34.26
33.97
31.46
31.38
30.26
29.82
28.34
27.99
21.09
14.22
BRUKER
Current Data Parameters
NAME aet7-27
EXPNO 11
PROCNO 1
F2 - Acquisition Parameters
Date_ 20111123
Time 19.33
INSTRUM DPX400
PROBHD 5 mm QNP 1H/13
PULPROG deptq_135
TD 65536
SOLVENT CDCl3
NS 512
DS 4
SWH 23980.814 Hz
FIDRES 0.365918 Hz
AQ 1.3664756 sec
RG 11585.2
DW 20.850 usec
DE 6.00 usec
TE 294.2 K
CNST2 145.0000000
D1 2.00000000 sec
d2 0.00344828 sec
D11 0.03000000 sec
DELTA 0.00001019 sec
======== CHANNEL f1 ========
NUC1 13C
P1 8.00 usec
P2 16.00 usec
PL1 -2.60 dB
SFO1 100.6228300 MHz
======== CHANNEL f2 ========
CPDPRG2 waltz16
NUC2 1H
P3 10.00 usec
P4 20.00 usec
PCPD2 80.00 usec
PL2 -1.50 dB
PL12 17.50 dB
SFO2 400.1316000 MHz
F2 - Processing parameters
SI 32768
SF 100.6127690 MHz
WDW EM
SSB 0
LB 1.00 Hz
GB 0
PC 1.40
200
180
160
140
120
100
80
60
40
20
0
ppm

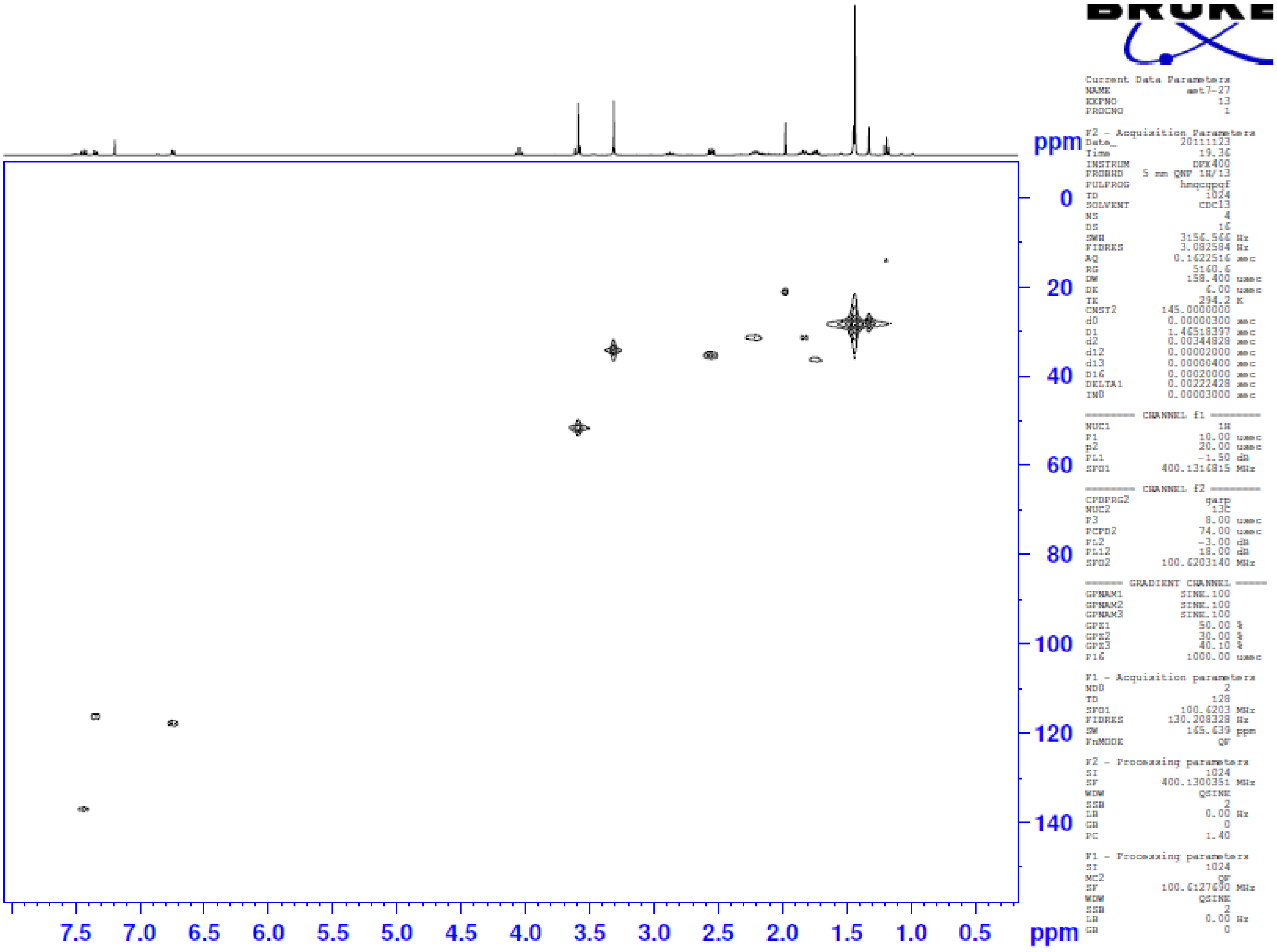
ppm
0
20
40
60
80
100
120
140
7.5
7.0
6.5
6.0
5.5
5.0
4.5
4.0
3.5
3.0
2.5
2.0
1.5
1.0
0.5
ppm
Current Data Parameters
NAME met7-27
EXPNO 13
PROCNO 1
F2 - Acquisition Parameters
Date_ 20111123
Time 19.36
INSTRUM DPX400
PROBHD 5 mm QNP 1H/13
PULPROG hmqcgpqf
TD 1024
SOLVENT CDCl3
NS 4
DS 16
SWH 3156.566 Hz
FIDRES 3.082584 Hz
AQ 0.1622516 sec
RG 5160.6
DW 158.400 usec
DE 6.00 usec
TE 294.2 K
CNST2 145.0000000
d0 0.00000300 sec
D1 1.46518397 sec
d2 0.00344828 sec
d12 0.00002000 sec
d13 0.00000400 sec
D16 0.00020000 sec
DELTA1 0.00222428 sec
IN0 0.00003000 sec
======== CHANNEL f1 ========
NUC1 1H
P1 10.00 usec
p2 20.00 usec
PL1 -1.50 dB
SFO1 400.1316815 MHz
======== CHANNEL f2 ========
CPDPRG2 garp
NUC2 13C
P3 8.00 usec
PCPD2 74.00 usec
PL2 -3.00 dB
PL12 18.00 dB
SFO2 100.6203140 MHz
====== GRADIENT CHANNEL =====
GPNAM1 SINE.100
GPNAM2 SINE.100
GPNAM3 SINE.100
GPZ1 50.00 %
GPZ2 30.00 %
GPZ3 40.10 %
P16 1000.00 usec
F1 - Acquisition parameters
ND0 2
TD 128
SFO1 100.6203 MHz
FIDRES 130.208328 Hz
SW 165.639 ppm
FnMODE QF
F2 - Processing parameters
SI 1024
SF 400.1300351 MHz
WDW QSINE
SSB 2
LB 0.00 Hz
GB 0
PC 1.40
F1 - Processing parameters
SI 1024
MC2 QF
SF 100.6127690 MHz
WDW QSINE
SSB 2
LB 0.00 Hz
GB 0

Methyl (*S*)-3-((1*r,3*R**)-3-(2-(6-((tert-butoxycarbonyl)(methyl)amino)pyridin-2-yl)ethyl)cyclobutane-1-carboxamido)-2-(phenylsulfonamido)propanoate [Compound 51]**

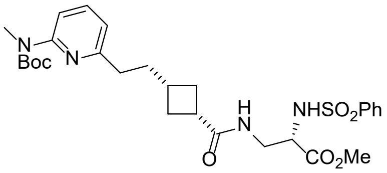

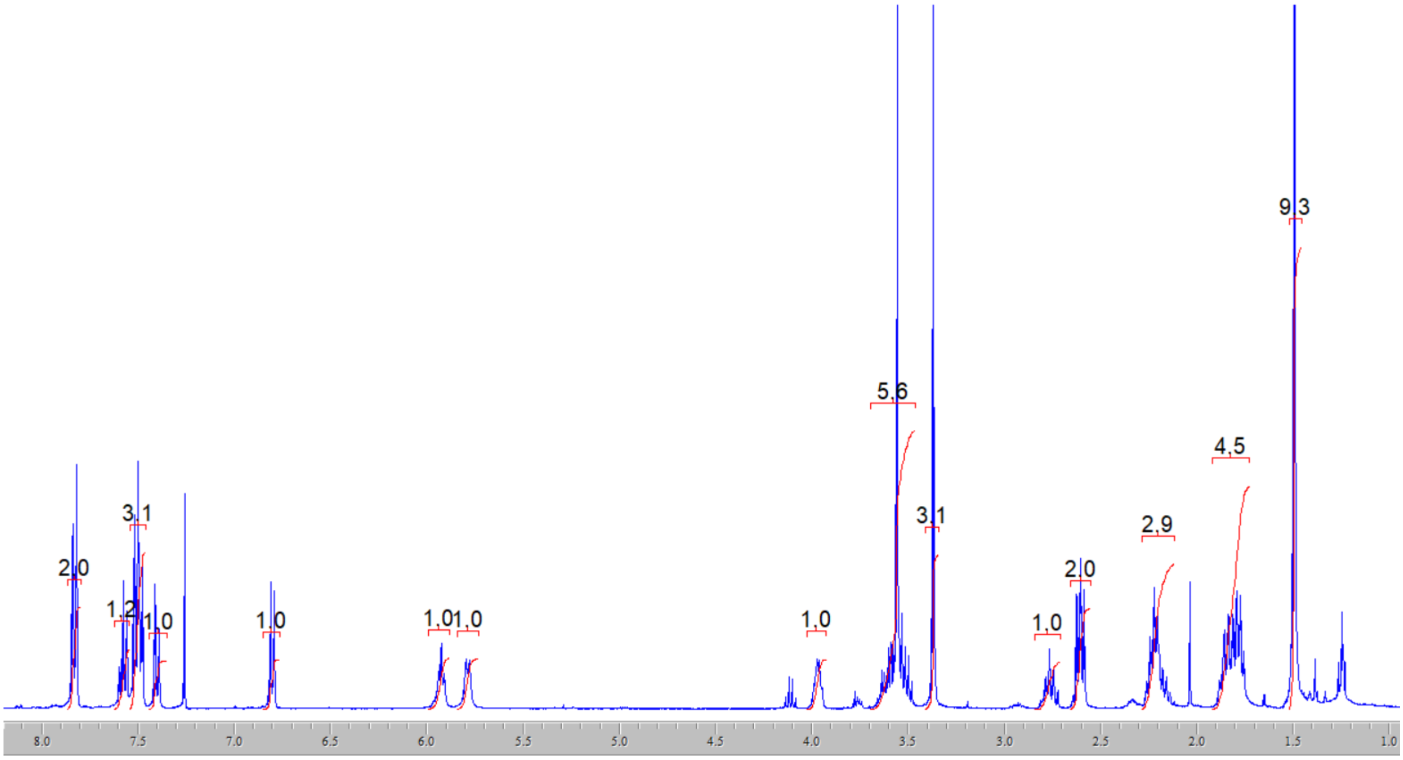

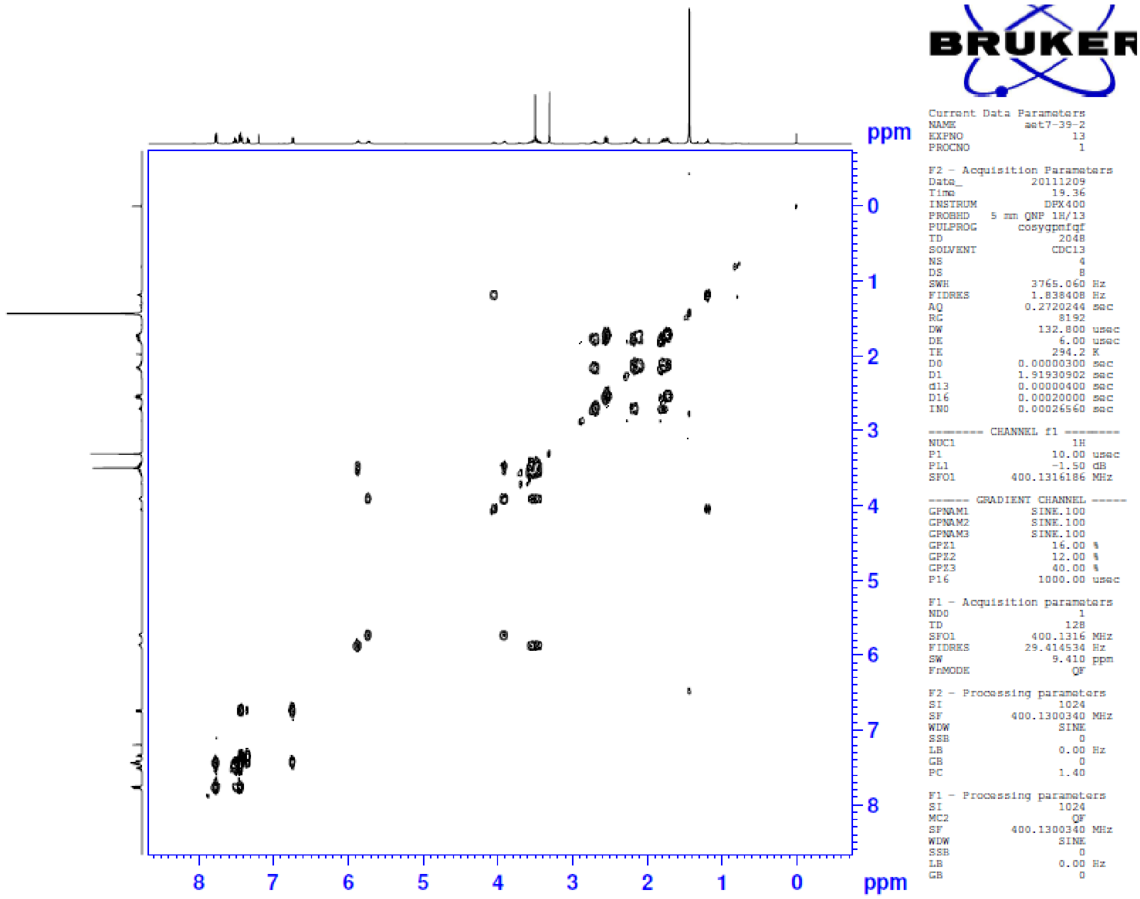

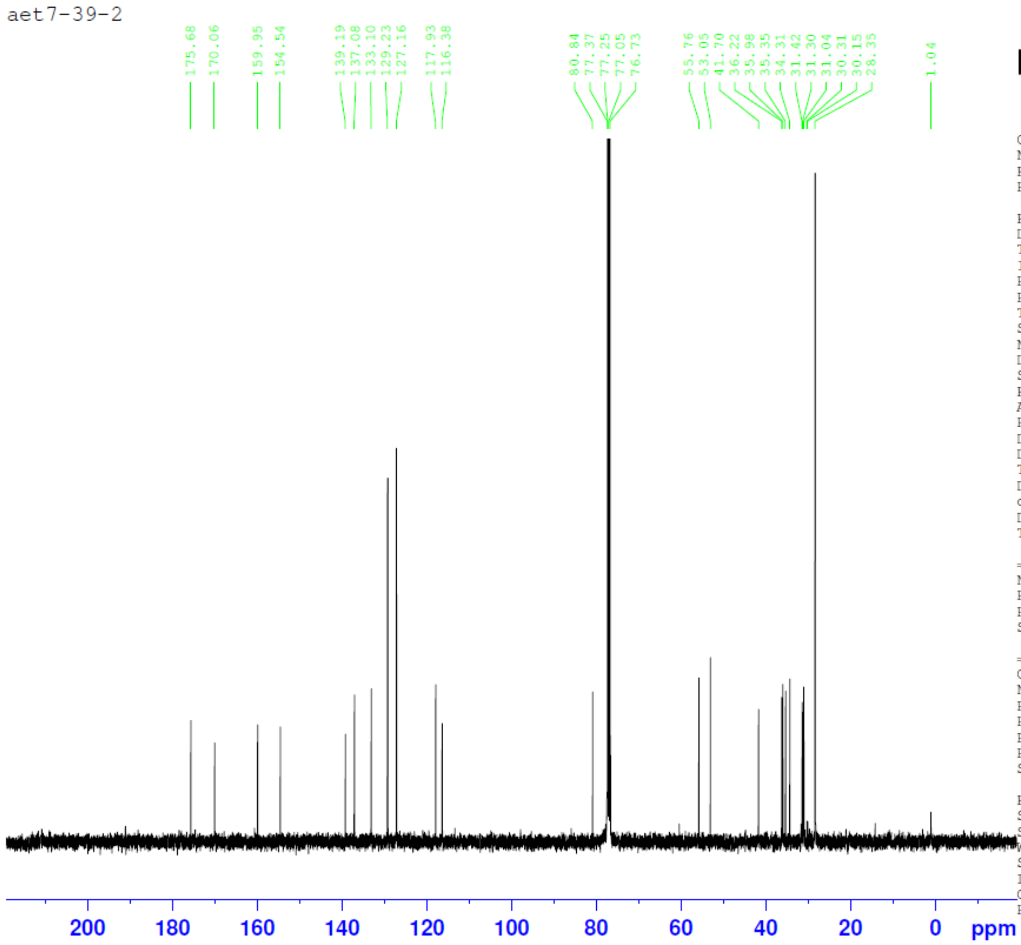


```
Current Data Parameters
NAME            aet7-39-2
EXPNO                  11
PROCNO                  1

F2 - Acquisition Parameters
Date_            20111209
Time                19.04
INSTRUM            DPX400
PROBHD   5 mm QNP 1H/13
PULPROG            zgpg30
TD                  65536
SOLVENT             CDCl3
NS                   1024
DS                      4
SWH             23980.814 Hz
FIDRES           0.365918 Hz
AQ              1.3664756 sec
RG                 1290.2
DW                 20.850 usec
DE                   6.00 usec
TE                  294.2 K
D1             2.00000000 sec
d11            0.03000000 sec
DELTA          1.89999998 sec
TD0                     1

======== CHANNEL f1 ========
NUC1                  13C
P1                   8.00 usec
PL1                 -2.60 dB
SFO1          100.6228298 MHz

======== CHANNEL f2 ========
CPDPRG2           waltz16
NUC2                   1H
PCPD2               80.00 usec
PL2                 -1.50 dB
PL12                17.50 dB
PL13                17.50 dB
SFO2          400.1316005 MHz

F2 - Processing parameters
SI                  32768
SF            100.6127690 MHz
WDW                    EM
SSB                     0
LB                   1.00 Hz
GB                      0
PC                   1.40
```

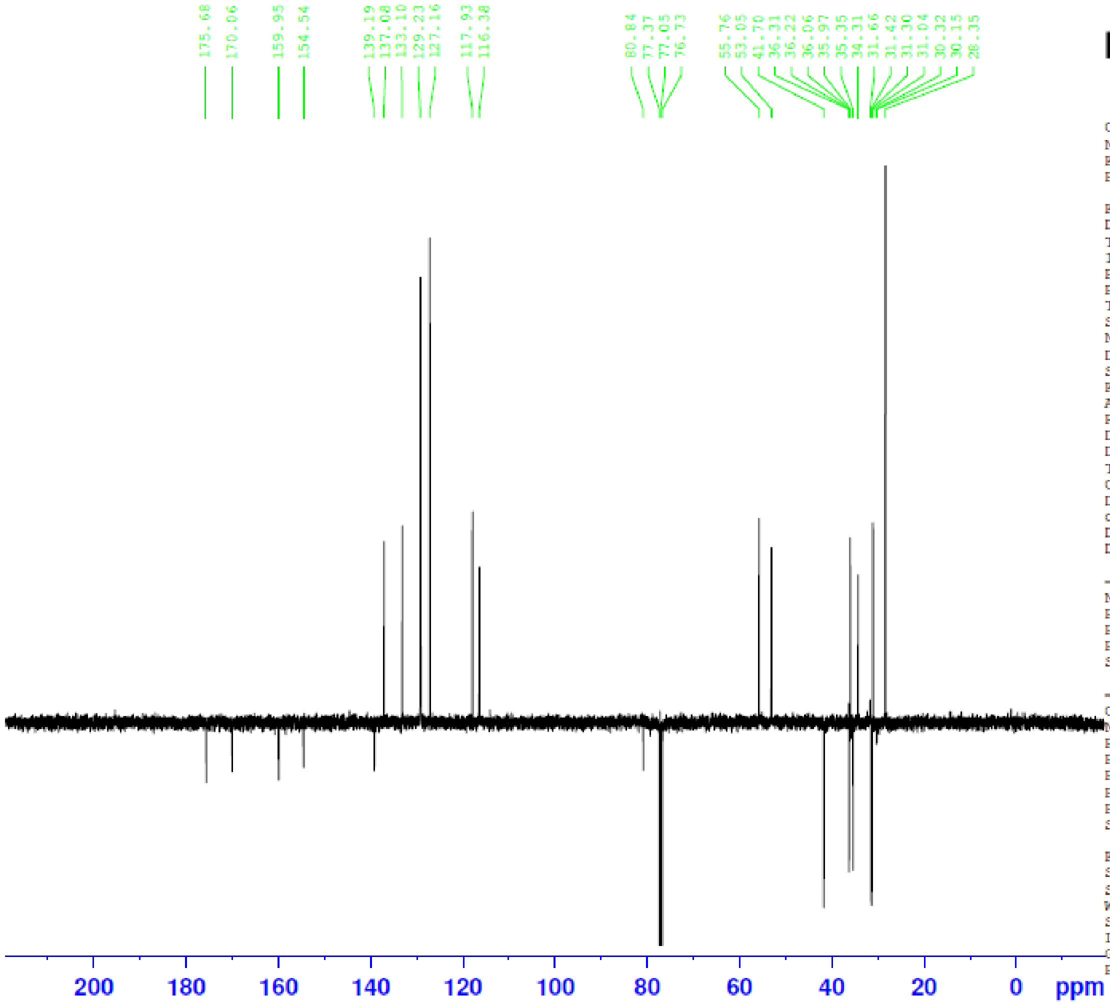


```
Current Data Parameters
NAME            aet7-39-2
EXPNO                  12
PROCNO                  1

F2 - Acquisition Parameters
Date_            20111209
Time                19.35
INSTRUM            DPX400
PROBHD   5 mm QNP 1H/13
PULPROG          deptq_135
TD                  65536
SOLVENT             CDCl3
NS                    512
DS                      4
SWH             23980.814 Hz
FIDRES           0.365918 Hz
AQ              1.3664756 sec
RG                18390.4
DW                 20.850 usec
DE                   6.00 usec
TE                  294.2 K
CNST2         145.0000000
D1             2.00000000 sec
d2             0.00344828 sec
D11            0.03000000 sec
DELTA          0.00001019 sec

======== CHANNEL f1 ========
NUC1                  13C
P1                   8.00 usec
P2                  16.00 usec
PL1                 -2.60 dB
SFO1          100.6228300 MHz

======== CHANNEL f2 ========
CPDPRG2           waltz16
NUC2                   1H
P3                  10.00 usec
P4                  20.00 usec
PCPD2               80.00 usec
PL2                 -1.50 dB
PL12                17.50 dB
SFO2          400.1316000 MHz

F2 - Processing parameters
SI                  32768
SF            100.6127690 MHz
WDW                    EM
SSB                     0
LB                   1.00 Hz
GB                      0
PC                   1.40
```

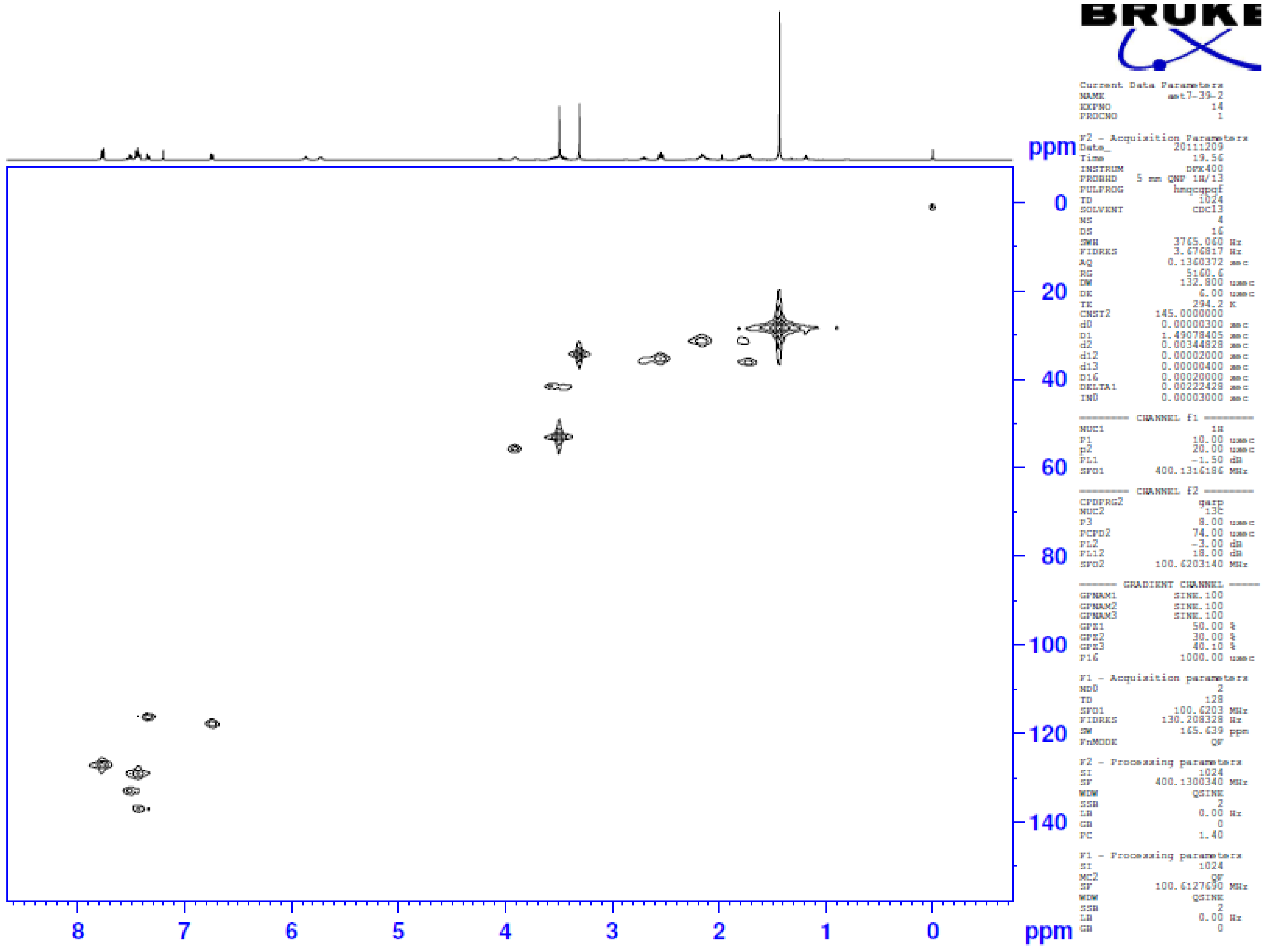
BRUKER
Current Data Parameters
NAME amt7-39-2
EXPNO 14
PROCNO 1
F2 - Acquisition Parameters
Date_ 20111209
Time 19.56
INSTRUM DPX400
PROBHD 5 mm QNP 1H/13
PULPROG hmqcgpqf
TD 1024
SOLVENT CDCl3
NS 4
DS 16
SWH 3765.060 Hz
FIDRES 3.676817 Hz
AQ 0.1360372 sec
RG 5160.6
DW 132.800 usec
DE 6.00 usec
TE 294.2 K
CNST2 145.0000000
d0 0.00000300 sec
D1 1.49078405 sec
d2 0.00344828 sec
d12 0.00002000 sec
d13 0.00000400 sec
D16 0.00020000 sec
DELTA1 0.00222428 sec
IN0 0.00003000 sec
======== CHANNEL f1 ========
NUC1 1H
P1 10.00 usec
p2 20.00 usec
PL1 -1.50 dB
SFO1 400.1316186 MHz
======== CHANNEL f2 ========
CPDPRG2 garp
NUC2 13C
P3 8.00 usec
PCPD2 74.00 usec
PL2 -3.00 dB
PL12 18.00 dB
SFO2 100.6203140 MHz
====== GRADIENT CHANNEL =====
GPNAM1 SINE.100
GPNAM2 SINE.100
GPNAM3 SINE.100
GPZ1 50.00 %
GPZ2 30.00 %
GPZ3 40.10 %
P16 1000.00 usec
F1 - Acquisition parameters
ND0 2
TD 128
SFO1 100.6203 MHz
FIDRES 130.208328 Hz
SW 165.639 ppm
FnMODE QF
F2 - Processing parameters
SI 1024
SF 400.1300340 MHz
WDW QSINE
SSB 2
LB 0.00 Hz
GB 0
PC 1.40
F1 - Processing parameters
SI 1024
MC2 QF
SF 100.6127690 MHz
WDW QSINE
SSB 2
LB 0.00 Hz
GB 0
ppm
0
20
40
60
80
100
120
140
8
7
6
5
4
3
2
1
0
ppm

Methyl (*S*)-3-((1*r,3*R**)-3-(2-(6-((tert-butoxycarbonyl)(methyl)amino)pyridin-2-yl)ethyl)cyclobutane-1-carboxamido)-2-(2,4,6-trimethylphenylsulfonamido)propanoate [Compound 52]**

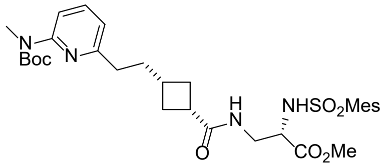

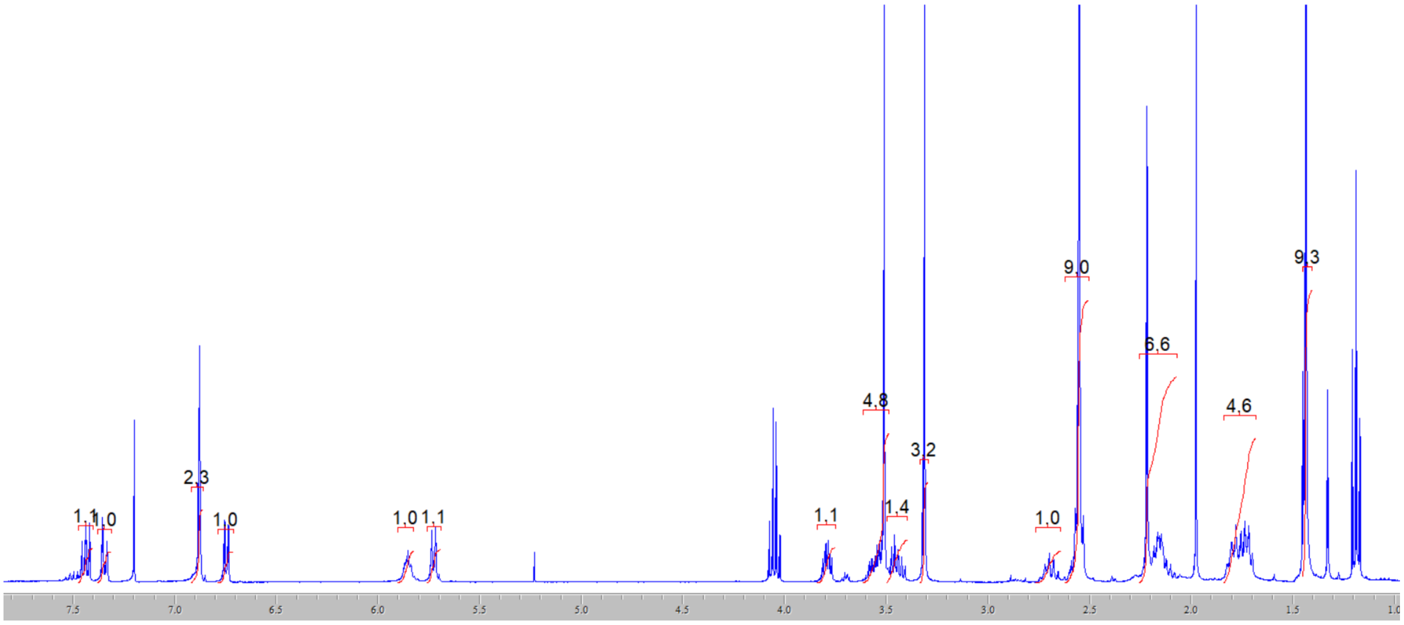

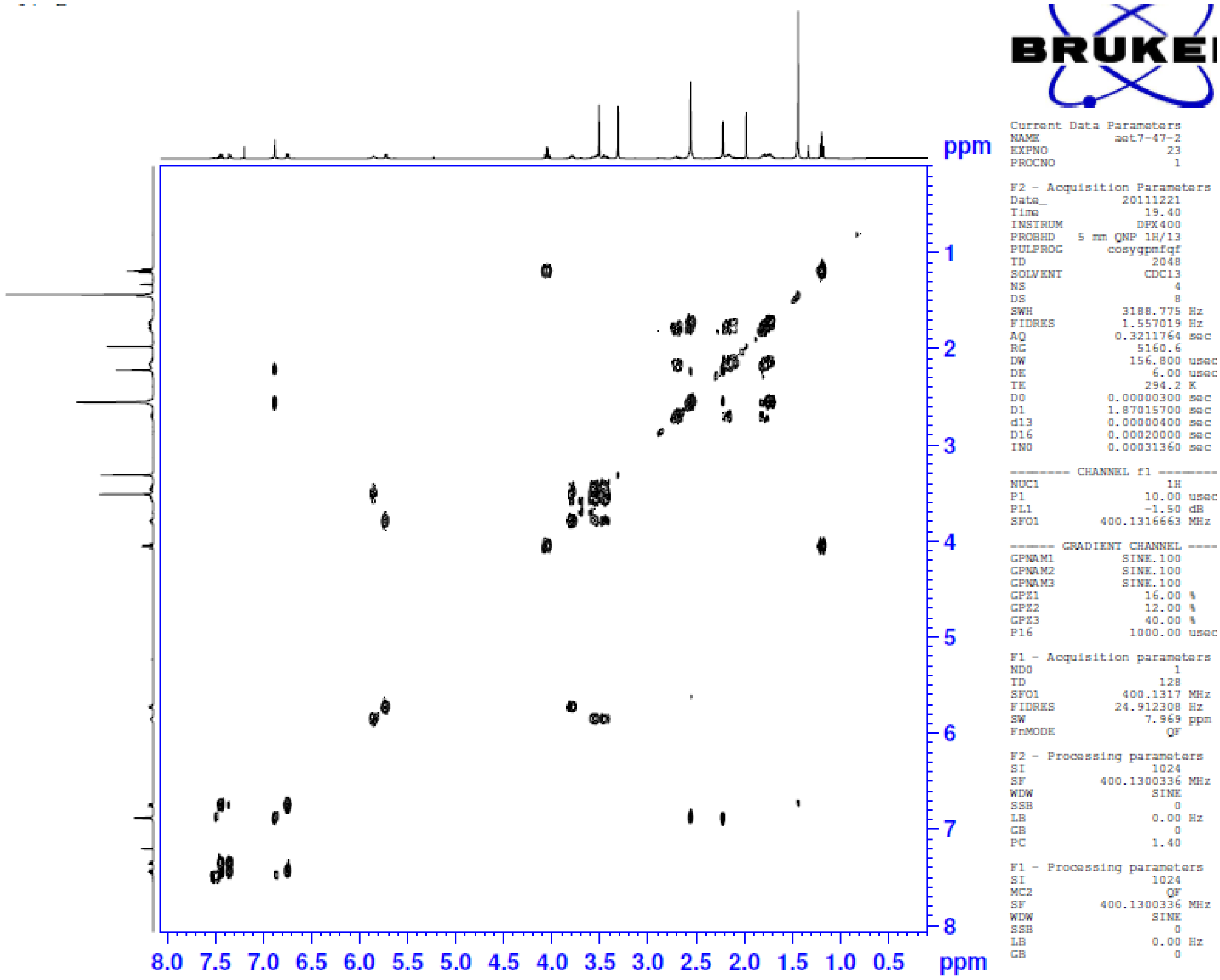

aet7-47-2

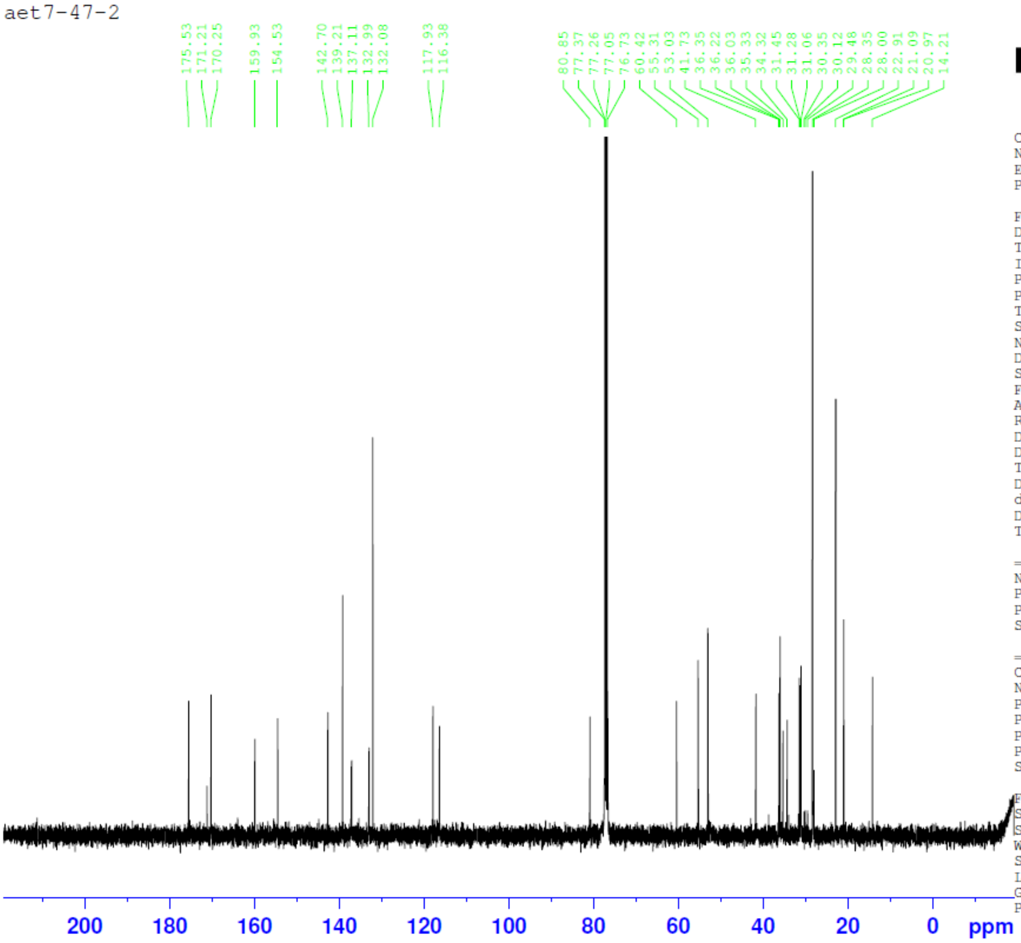

BRUKER

Current Data Parameters	
NAME	aet7-47-2
EXPNO	21
PROCNO	1
F2 - Acquisition Parameters	
Date_	20111221
Time	19.09
INSTRUM	DPX400
PROBHD	5 mm QNP 1H/13
PULPROG	zgpg30
TD	65536
SOLVENT	CDCl3
NS	1024
DS	4
SWH	23980.814 Hz
FIDRES	0.365918 Hz
AQ	1.3664756 sec
RG	1024
DW	20.850 usec
DE	6.00 usec
TE	294.2 K
D1	2.00000000 sec
d11	0.03000000 sec
DELTA	1.89999998 sec
TD0	1
======== CHANNEL f1 ========	
NUC1	13C
P1	8.00 usec
PL1	-2.60 dB
SFO1	100.6228298 MHz
======== CHANNEL f2 ========	
CPDPRG2	waltz16
NUC2	1H
PCPD2	80.00 usec
PL2	-1.50 dB
PL12	17.50 dB
PL13	17.50 dB
SFO2	400.1316005 MHz
F2 - Processing parameters	
SI	32768
SF	100.6127690 MHz
WDW	EM
SSB	0
LB	1.00 Hz
GB	0
PC	1.40

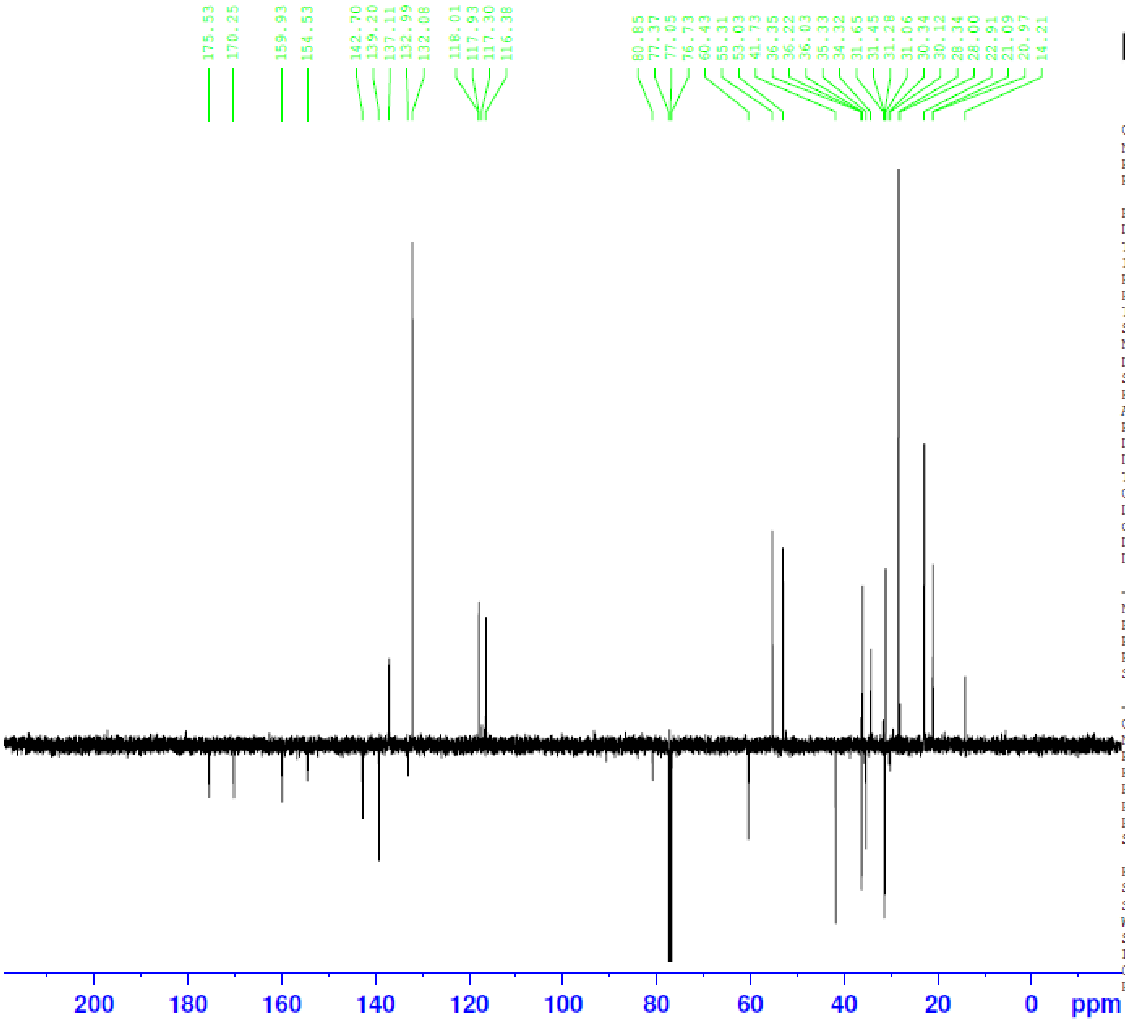

BRUKER

Current Data Parameters	
NAME	aet7-47-2
EXPNO	22
PROCNO	1
F2 - Acquisition Parameters	
Date_	20111221
Time	19.39
INSTRUM	DPX400
PROBHD	5 mm QNP 1H/13
PULPROG	deptq_135
TD	65536
SOLVENT	CDCl3
NS	512
DS	4
SWH	23980.814 Hz
FIDRES	0.365918 Hz
AQ	1.3664756 sec
RG	16384
DW	20.850 use
DE	6.00 use
TE	294.2 K
CNST2	145.0000000
D1	2.00000000 sec
d2	0.00344828 sec
D11	0.03000000 sec
DELTA	0.00001019 sec
======== CHANNEL f1 ========	
NUC1	13C
P1	8.00 use
P2	16.00 use
PL1	-2.60 dB
SFO1	100.6228300 MHz
======== CHANNEL f2 ========	
CPDPRG2	waltz16
NUC2	1H
P3	10.00 use
P4	20.00 use
PCPD2	80.00 use
PL2	-1.50 dB
PL12	17.50 dB
SFO2	400.1316000 MHz
F2 - Processing parameters	
SI	32768
SF	100.6127690 MHz
WDW	EM
SSB	0
LB	1.00 Hz
GB	0
PC	1.40

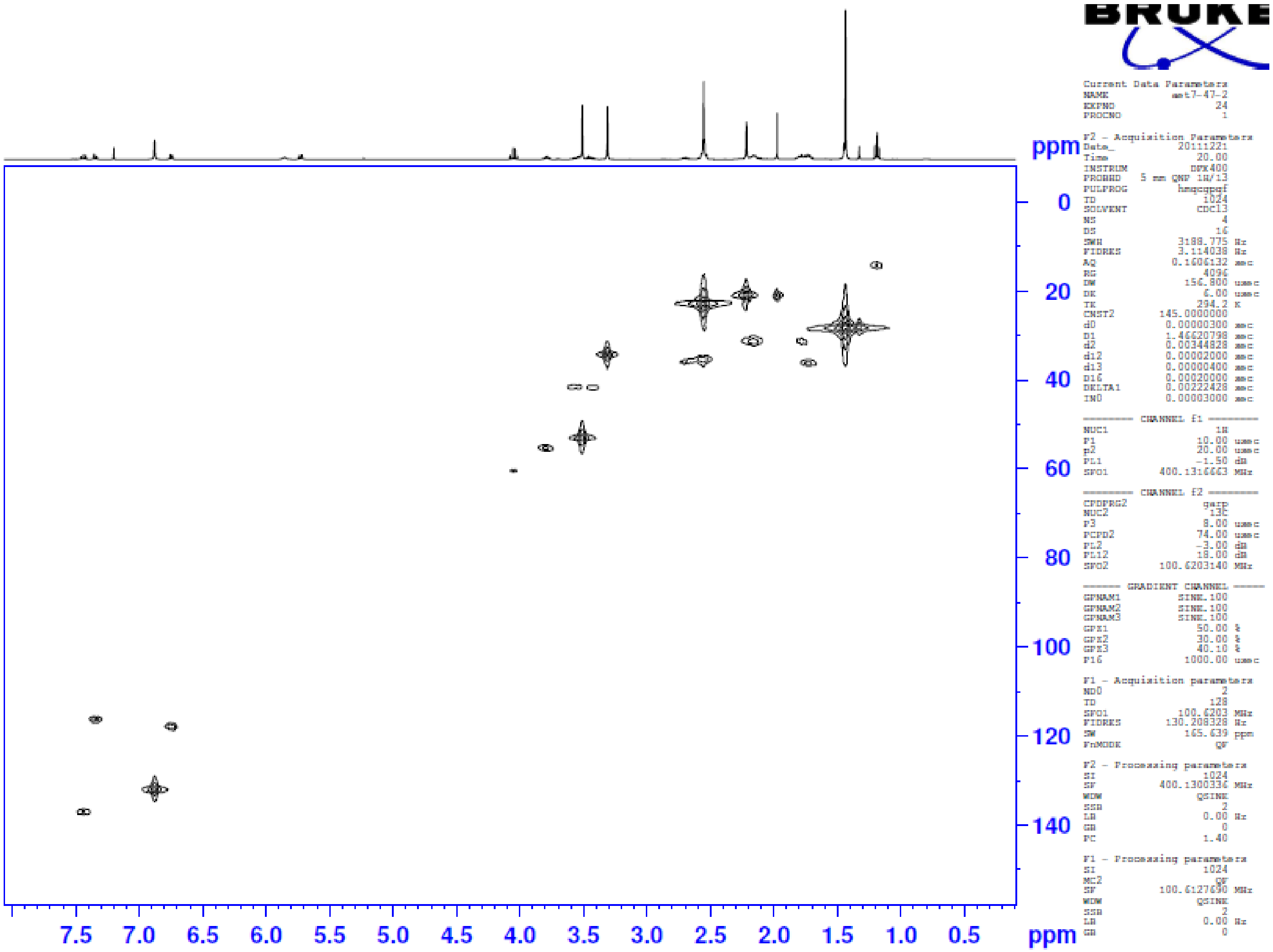
ppm
0
20
40
60
80
100
120
140
7.5 7.0 6.5 6.0 5.5 5.0 4.5 4.0 3.5 3.0 2.5 2.0 1.5 1.0 0.5 ppm
Current Data Parameters
NAME met7-47-2
EXPNO 24
PROCNO 1
F2 - Acquisition Parameters
Date_ 20111221
Time 20.00
INSTRUM DPX400
PROBHD 5 mm QNP 1H/13
PULPROG hsqcgpqf
TD 1024
SOLVENT CDCl3
NS 4
DS 16
SWH 3188.775 Hz
FIDRES 3.114038 Hz
AQ 0.1606132 sec
RG 4096
DW 156.800 usec
DE 6.00 usec
TE 294.2 K
CNST2 145.0000000
d0 0.00000300 sec
D1 1.46620798 sec
d2 0.00344828 sec
d12 0.00002000 sec
d13 0.00000400 sec
D16 0.00020000 sec
DELTA1 0.00222428 sec
IN0 0.00003000 sec
======== CHANNEL f1 ========
NUC1 1H
P1 10.00 usec
p2 20.00 usec
PL1 -1.50 dB
SFO1 400.1316663 MHz
======== CHANNEL f2 ========
CPDPRG2 garp
NUC2 13C
P3 8.00 usec
PCPD2 74.00 usec
PL2 -3.00 dB
PL12 18.00 dB
SFO2 100.6203140 MHz
====== GRADIENT CHANNEL =====
GPNAM1 SINE.100
GPNAM2 SINE.100
GPNAM3 SINE.100
GPZ1 50.00 %
GPZ2 30.00 %
GPZ3 40.10 %
P16 1000.00 usec
F1 - Acquisition parameters
ND0 2
TD 128
SFO1 100.6203 MHz
FIDRES 130.208328 Hz
SW 165.639 ppm
FnMODE QF
F2 - Processing parameters
SI 1024
SF 400.1300336 MHz
WDW QSINE
SSB 2
LB 0.00 Hz
GB 0
PC 1.40
F1 - Processing parameters
SI 1024
MC2 QF
SF 100.6127690 MHz
WDW QSINE
SSB 2
LB 0.00 Hz
GB 0

Methyl (*S*)-3-((1*r,3*R**)-3-(2-(6-(methylamino)pyridin-2-yl)ethyl)cyclobutane-1-carboxamido)-2-(phenylsulfonamido)propanoate [Compound 53]**

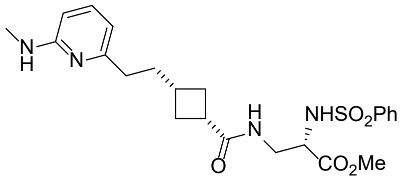

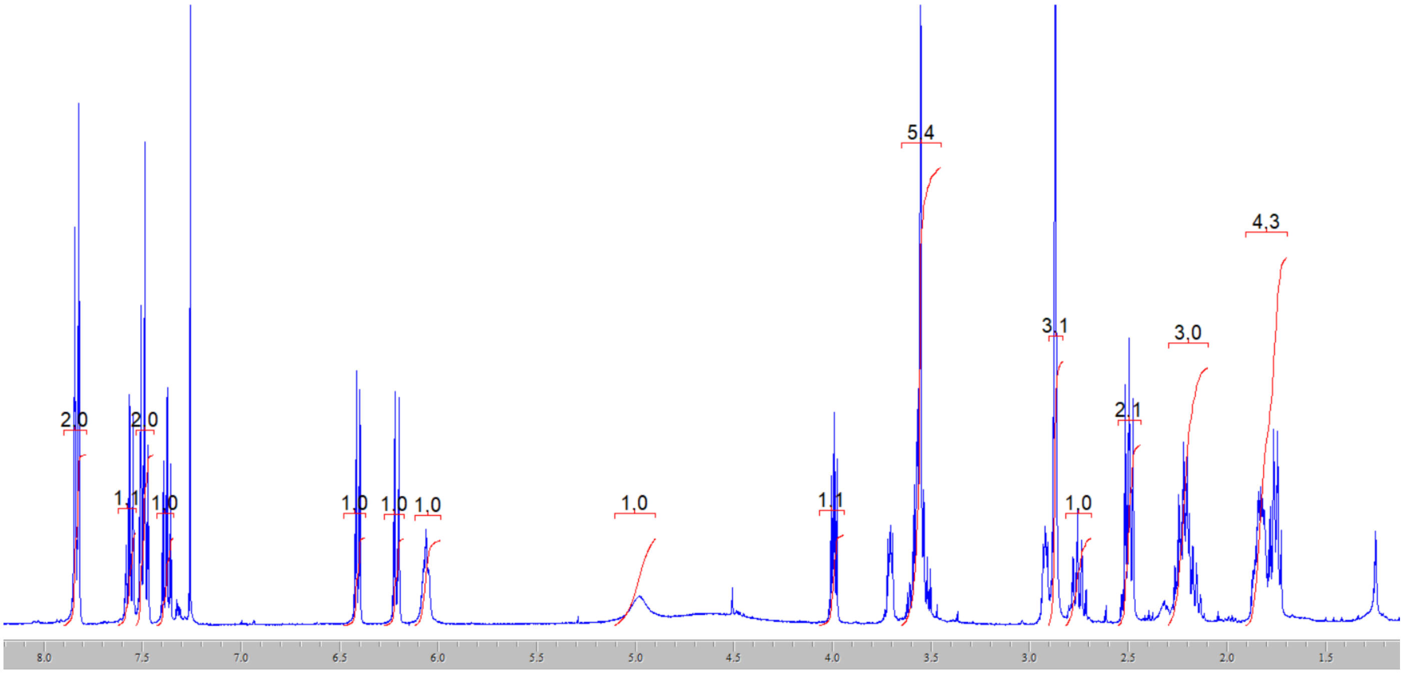

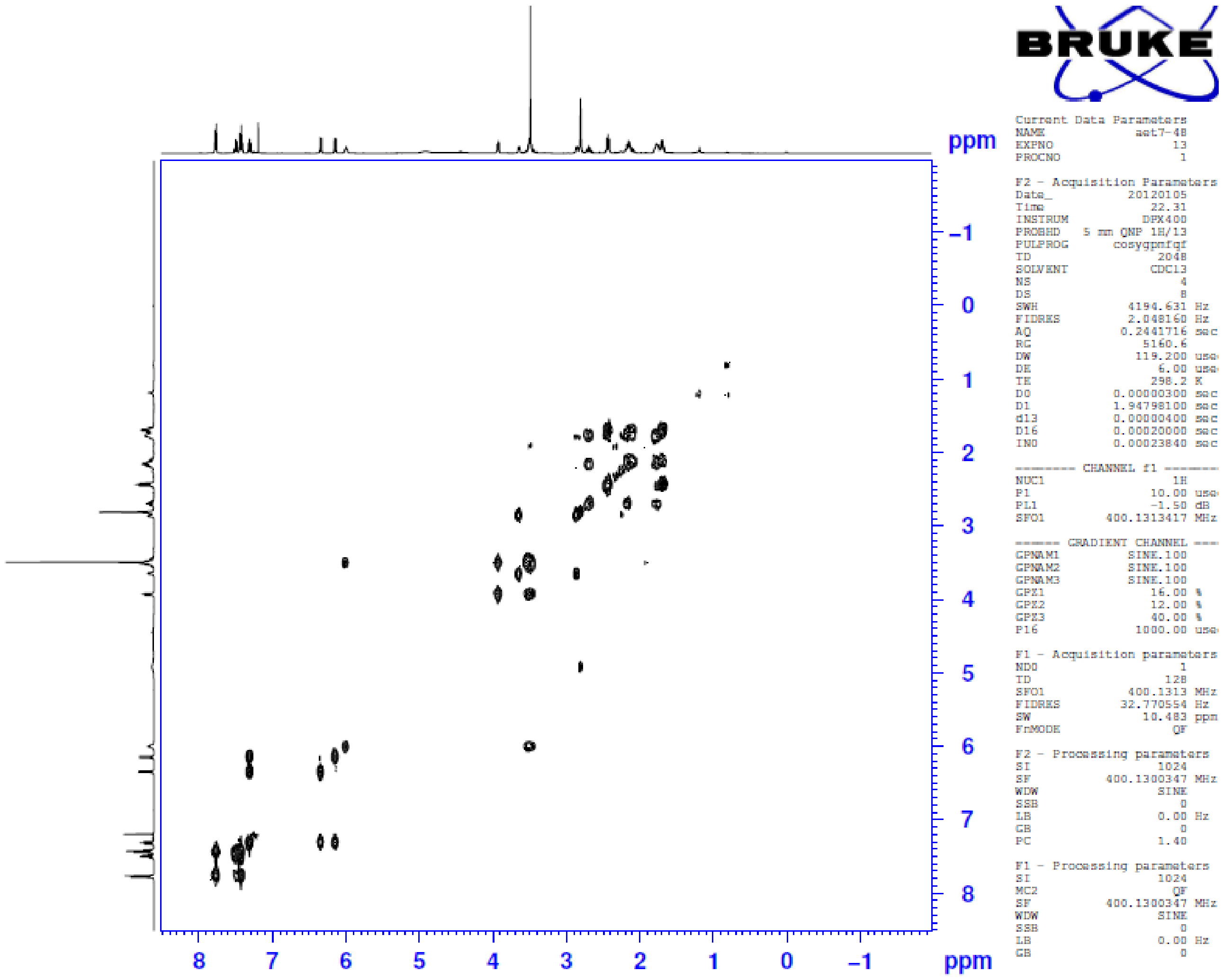

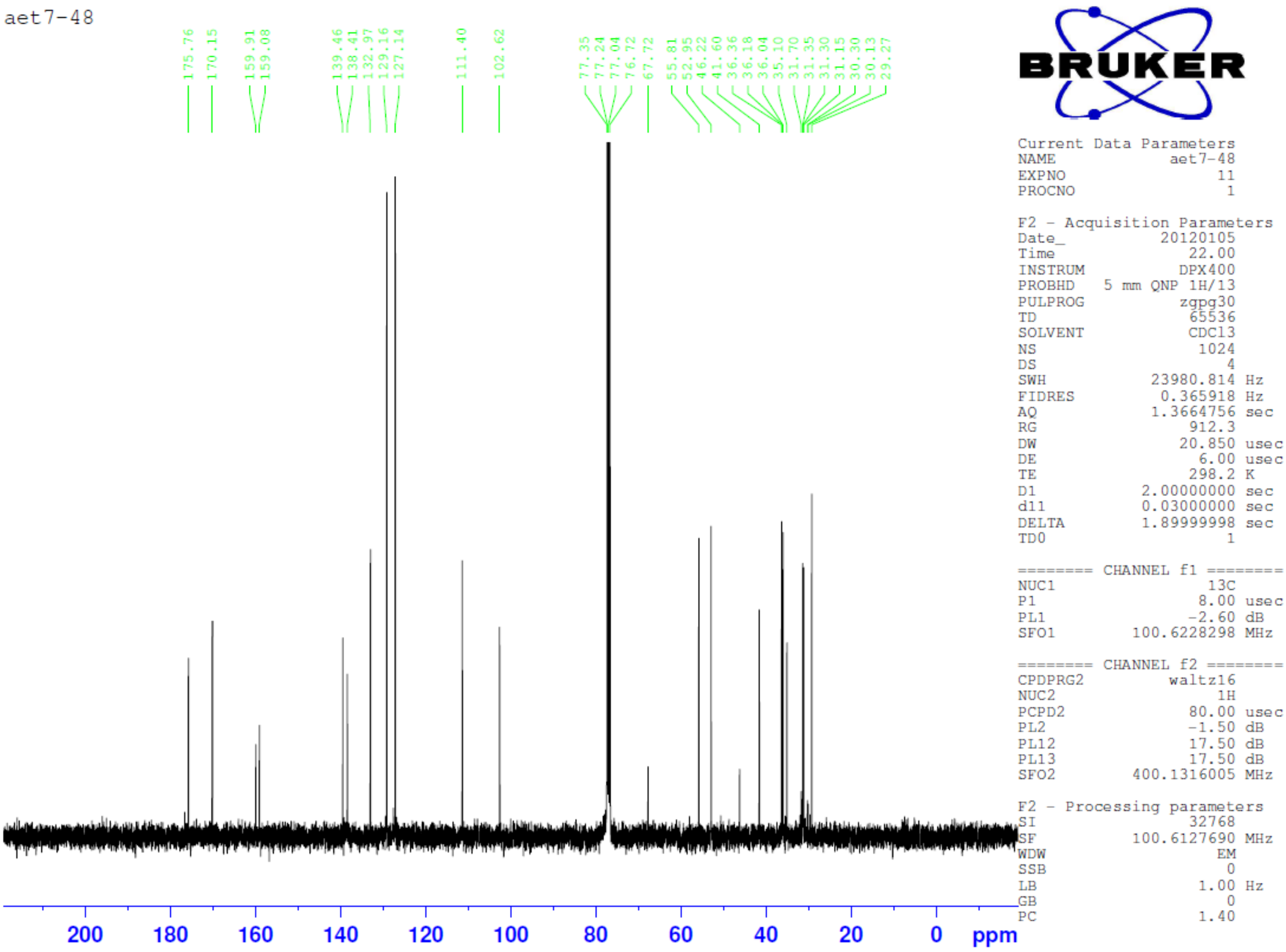
aet7-48
175.76
170.15
159.91
159.08
139.46
138.41
132.97
129.16
127.14
111.40
102.62
77.35
77.24
77.04
76.72
67.72
55.81
52.95
46.22
41.60
36.36
36.18
36.04
35.10
31.70
31.35
31.30
31.15
30.30
30.13
29.27
BRUKER
Current Data Parameters
NAME aet7-48
EXPNO 11
PROCNO 1
F2 - Acquisition Parameters
Date_ 20120105
Time 22.00
INSTRUM DPX400
PROBHD 5 mm QNP 1H/13
PULPROG zgpg30
TD 65536
SOLVENT CDCl3
NS 1024
DS 4
SWH 23980.814 Hz
FIDRES 0.365918 Hz
AQ 1.3664756 sec
RG 912.3
DW 20.850 usec
DE 6.00 usec
TE 298.2 K
D1 2.00000000 sec
d11 0.03000000 sec
DELTA 1.89999998 sec
TD0 1
======== CHANNEL f1 ========
NUC1 13C
P1 8.00 usec
PL1 -2.60 dB
SFO1 100.6228298 MHz
======== CHANNEL f2 ========
CPDPRG2 waltz16
NUC2 1H
PCPD2 80.00 usec
PL2 -1.50 dB
PL12 17.50 dB
PL13 17.50 dB
SFO2 400.1316005 MHz
F2 - Processing parameters
SI 32768
SF 100.6127690 MHz
WDW EM
SSB 0
LB 1.00 Hz
GB 0
PC 1.40
200 180 160 140 120 100 80 60 40 20 0 ppm

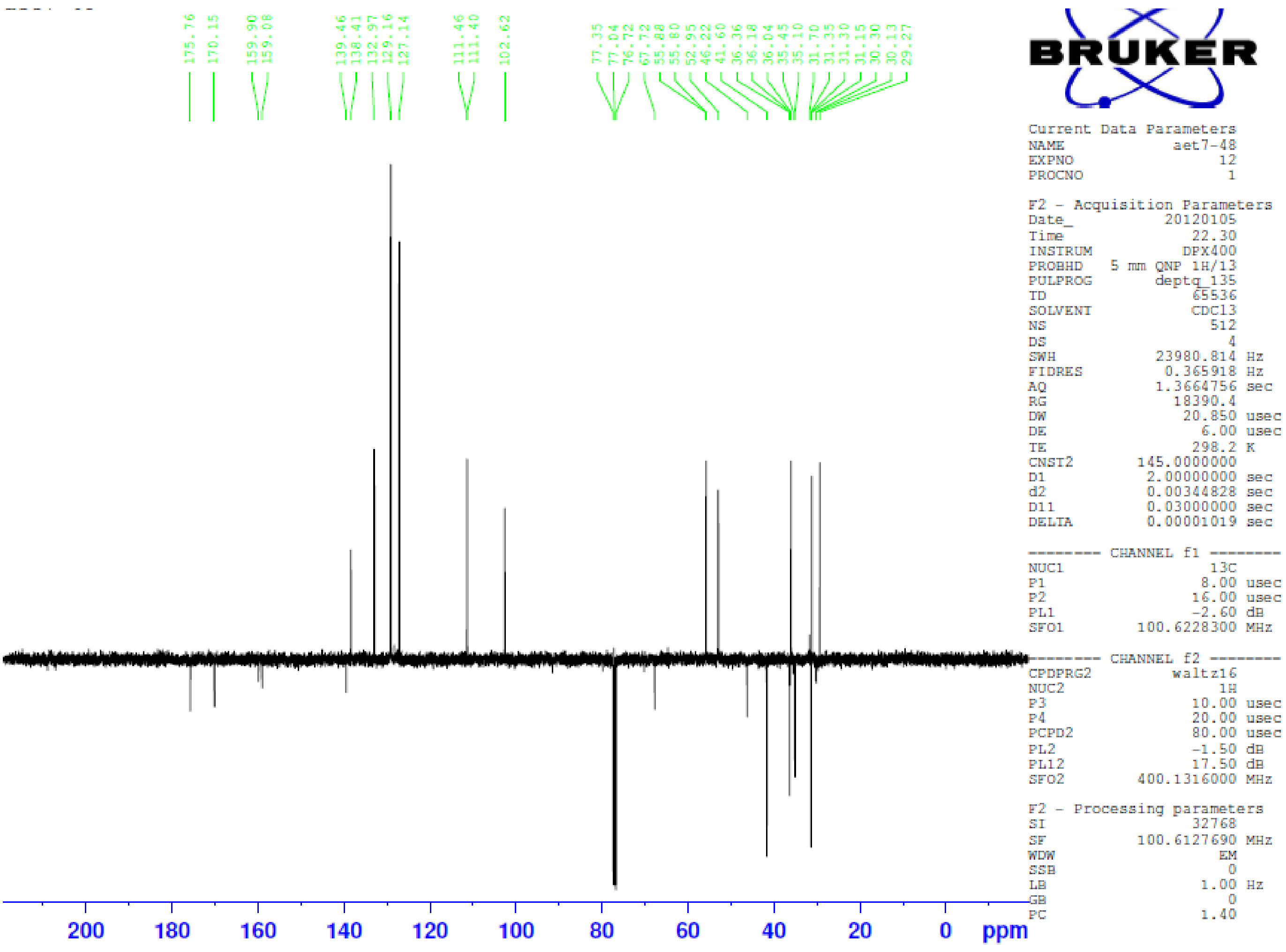
175.76
170.15
159.90
159.08
139.46
138.41
132.97
129.16
127.14
111.46
111.40
102.62
77.35
77.04
76.72
67.72
55.88
55.80
52.95
46.22
41.60
36.36
36.18
36.04
35.45
35.10
31.70
31.35
31.30
31.15
30.30
30.13
29.27
BRUKER
Current Data Parameters
NAME aet7-48
EXPNO 12
PROCNO 1
F2 - Acquisition Parameters
Date_ 20120105
Time 22.30
INSTRUM DPX400
PROBHD 5 mm QNP 1H/13
PULPROG deptq_135
TD 65536
SOLVENT CDCl3
NS 512
DS 4
SWH 23980.814 Hz
FIDRES 0.365918 Hz
AQ 1.3664756 sec
RG 18390.4
DW 20.850 usec
DE 6.00 usec
TE 298.2 K
CNST2 145.0000000
D1 2.00000000 sec
d2 0.00344828 sec
D11 0.03000000 sec
DELTA 0.00001019 sec
======== CHANNEL f1 ========
NUC1 13C
P1 8.00 usec
P2 16.00 usec
PL1 -2.60 dB
SFO1 100.6228300 MHz
======== CHANNEL f2 ========
CPDPRG2 waltz16
NUC2 1H
P3 10.00 usec
P4 20.00 usec
PCPD2 80.00 usec
PL2 -1.50 dB
PL12 17.50 dB
SFO2 400.1316000 MHz
F2 - Processing parameters
SI 32768
SF 100.6127690 MHz
WDW EM
SSB 0
LB 1.00 Hz
GB 0
PC 1.40
200 180 160 140 120 100 80 60 40 20 0 ppm

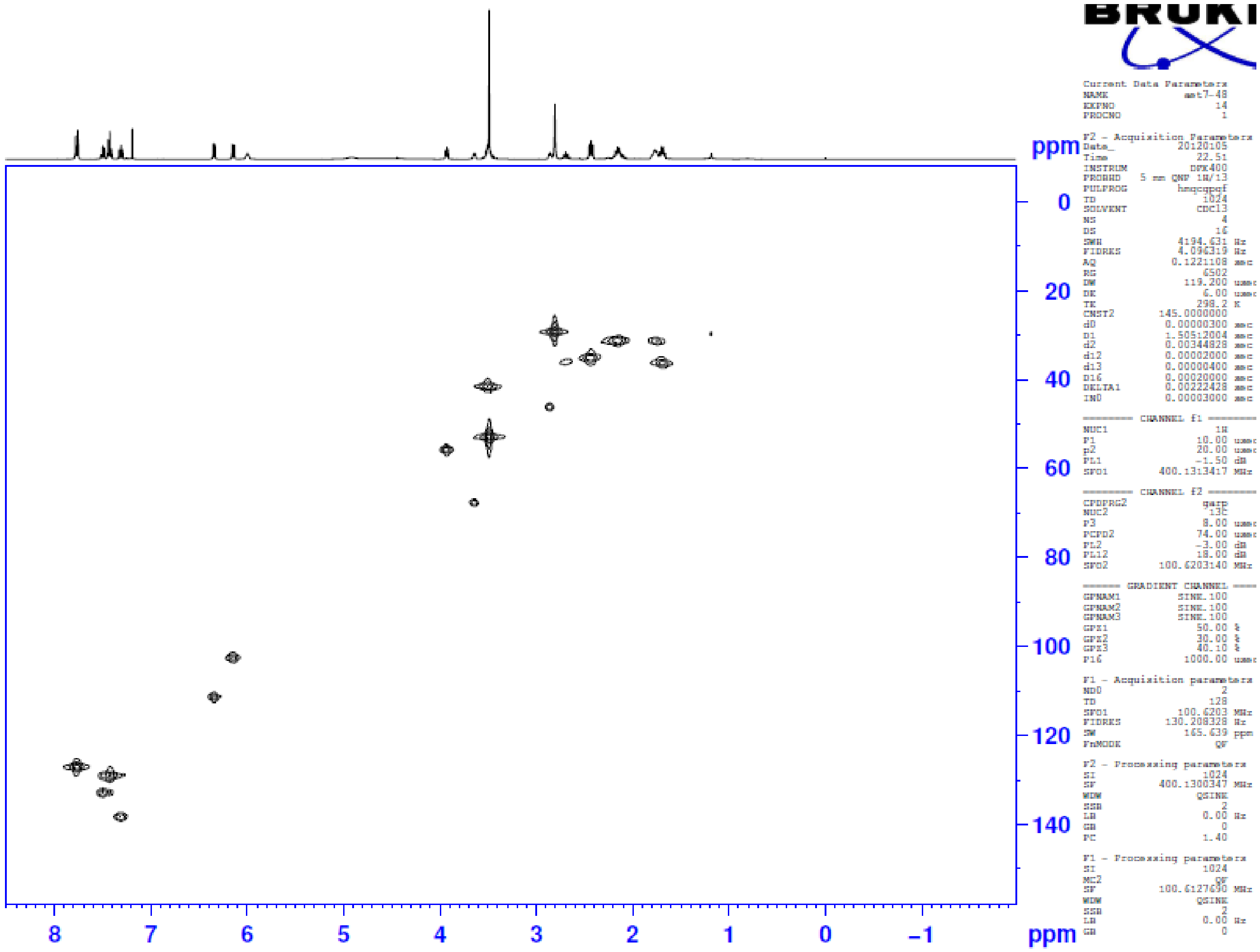
ppm
0
20
40
60
80
100
120
140
8
7
6
5
4
3
2
1
0
−1
ppm
Current Data Parameters
NAME ant7-48
EXPNO 14
PROCNO 1
F2 - Acquisition Parameters
Date_ 20120105
Time 22.51
INSTRUM DPX400
PROBHD 5 mm QNP 1H/13
PULPROG hmqcgpqf
TD 1024
SOLVENT CDCl3
NS 4
DS 16
SWH 4194.631 Hz
FIDRES 4.096319 Hz
AQ 0.1221108 sec
RG 6502
DW 119.200 usec
DE 6.00 usec
TE 298.2 K
CNST2 145.0000000
d0 0.00000300 sec
D1 1.50512004 sec
d2 0.00344828 sec
d12 0.00002000 sec
d13 0.00000400 sec
D16 0.00020000 sec
DELTA1 0.00222428 sec
IN0 0.00003000 sec
======== CHANNEL f1 ========
NUC1 1H
P1 10.00 usec
p2 20.00 usec
PL1 -1.50 dB
SFO1 400.1313417 MHz
======== CHANNEL f2 ========
CPDPRG2 garp
NUC2 13C
P3 8.00 usec
PCPD2 74.00 usec
PL2 -3.00 dB
PL12 18.00 dB
SFO2 100.6203140 MHz
====== GRADIENT CHANNEL =====
GPNAM1 SINE.100
GPNAM2 SINE.100
GPNAM3 SINE.100
GPZ1 50.00 %
GPZ2 30.00 %
GPZ3 40.10 %
P16 1000.00 usec
F1 - Acquisition parameters
ND0 2
TD 128
SFO1 100.6203 MHz
FIDRES 130.208328 Hz
SW 165.639 ppm
FnMODE QF
F2 - Processing parameters
SI 1024
SF 400.1300347 MHz
WDW QSINE
SSB 2
LB 0.00 Hz
GB 0
PC 1.40
F1 - Processing parameters
SI 1024
MC2 QF
SF 100.6127690 MHz
WDW QSINE
SSB 2
LB 0.00 Hz
GB 0

Methyl (*S*)-3-((1r*,3R*)-3-(2-(6-(methylamino)pyridin-2-yl)ethyl)cyclobutane-1-carboxamido)-2-(2,4,6-trimethylphenylsulfonamido)propanoate [Compound 54]

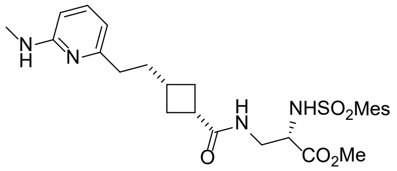

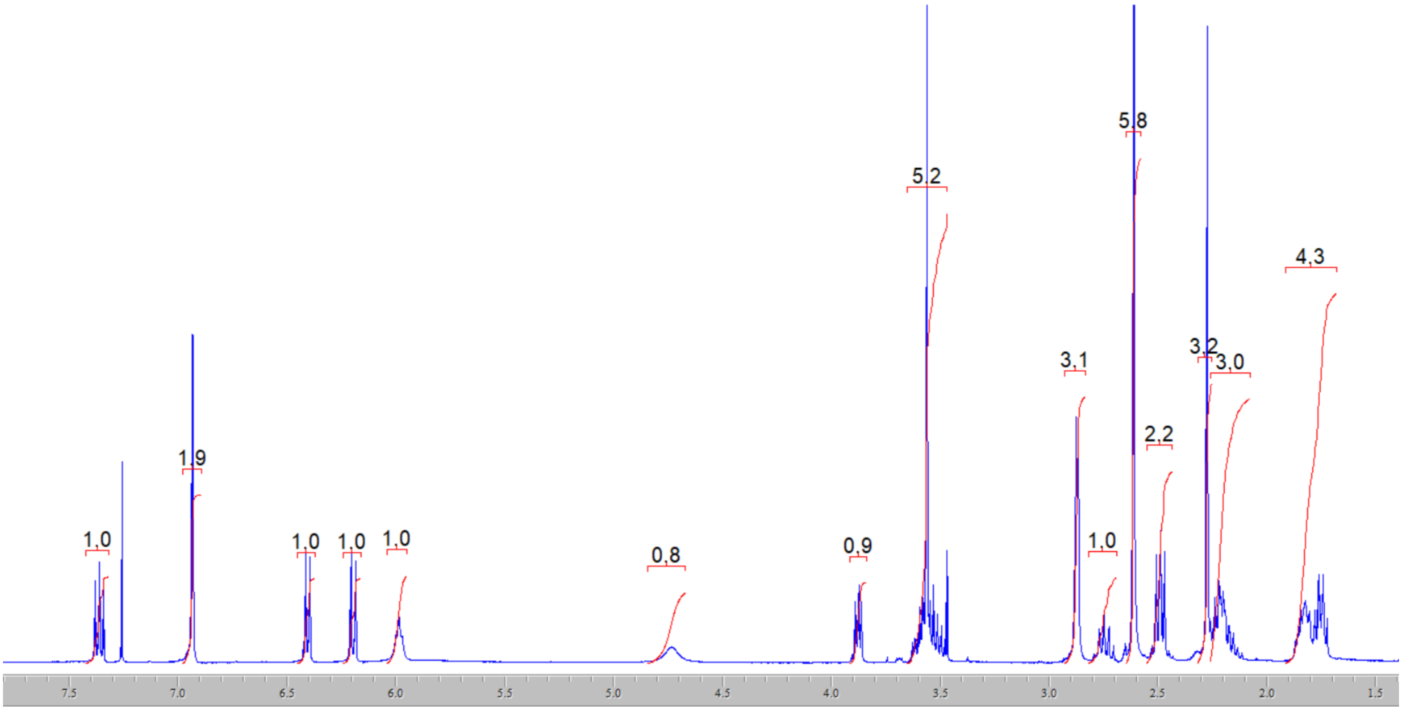

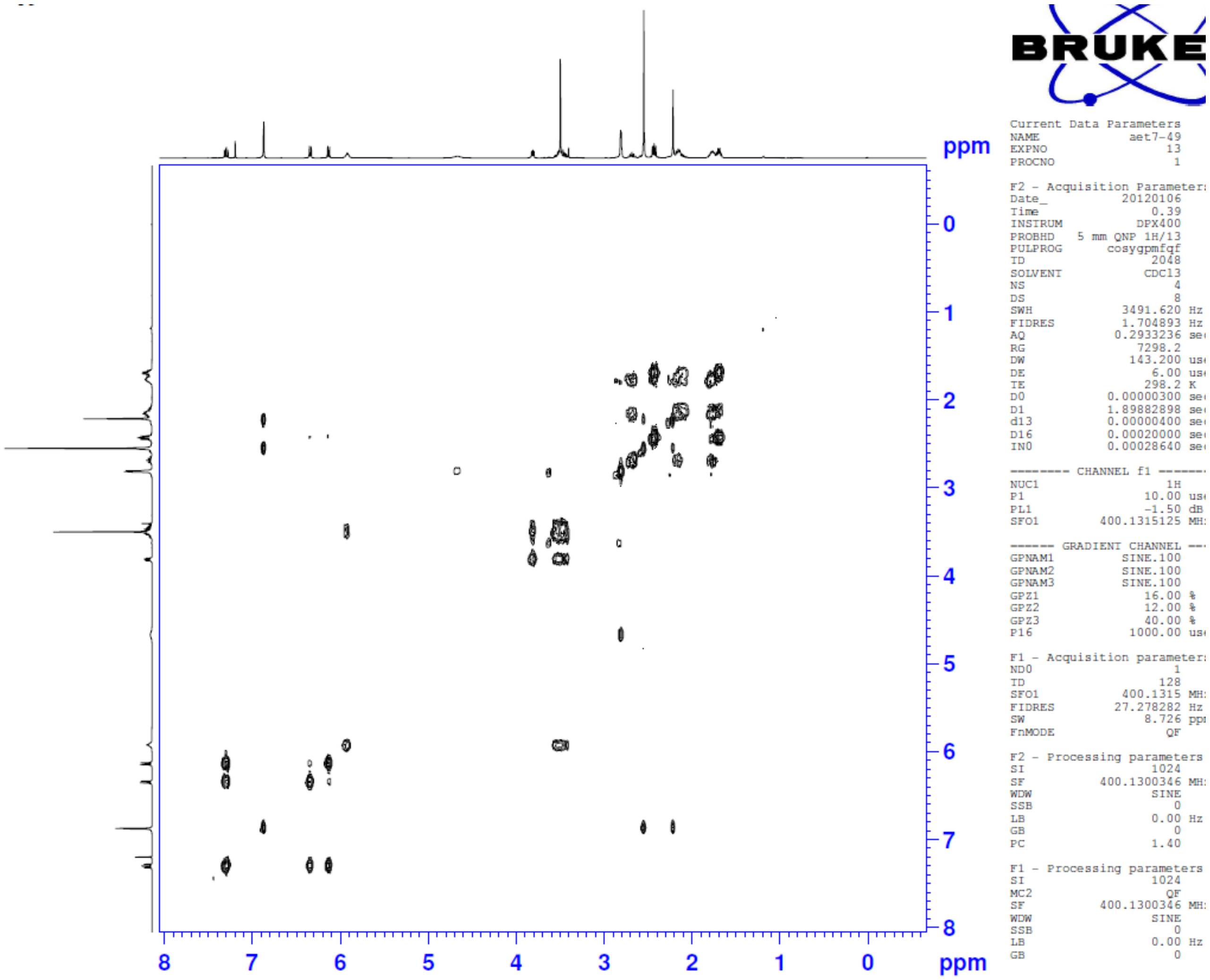

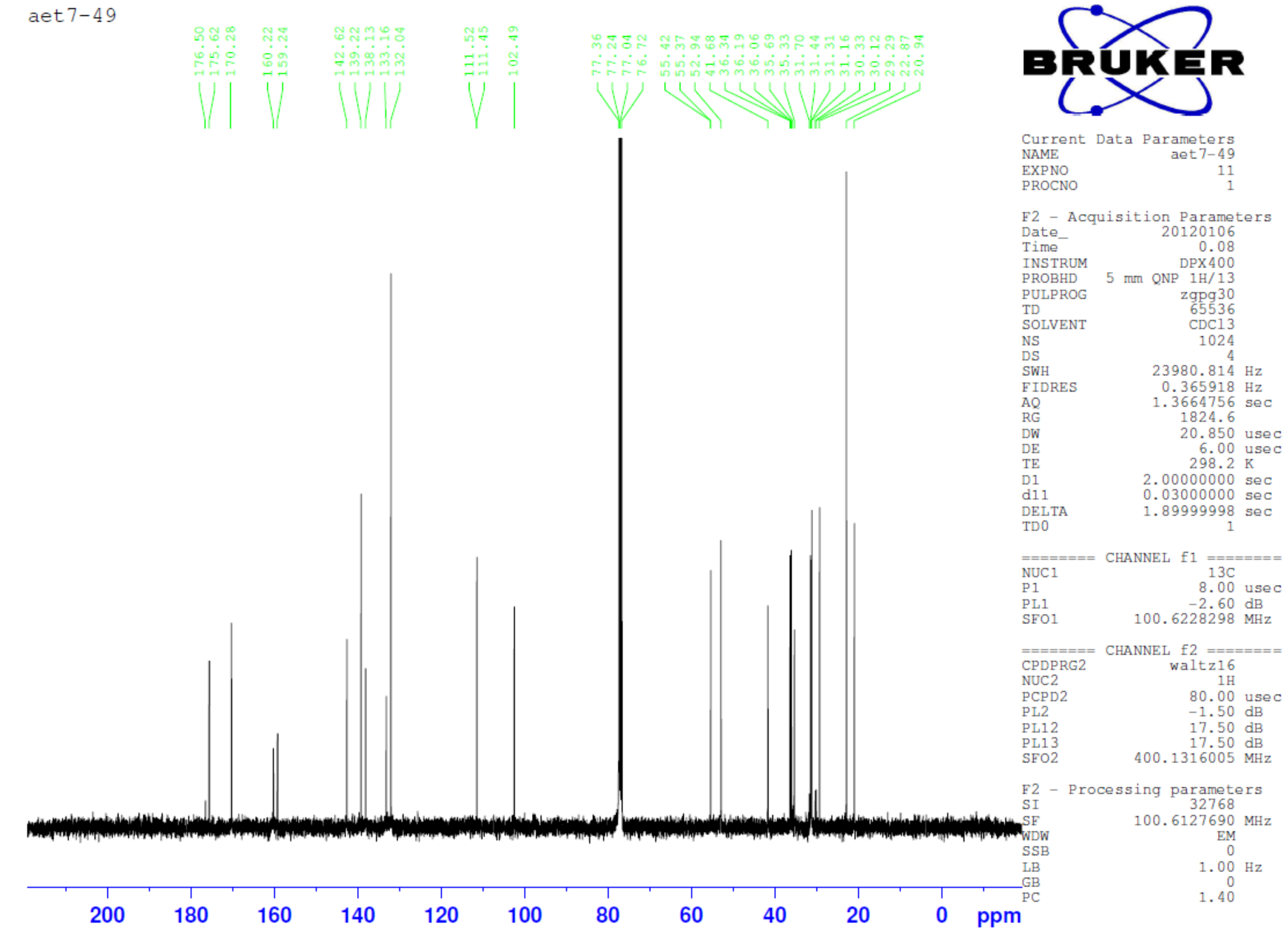
aet7-49
176.50
175.62
170.28
160.22
159.24
142.62
139.22
138.13
133.16
132.04
111.52
111.45
102.49
77.36
77.24
77.04
76.72
55.42
55.37
52.94
41.68
36.34
36.19
36.06
35.69
35.33
31.70
31.44
31.31
31.16
30.33
30.12
29.29
22.87
20.94
BRUKER
Current Data Parameters
NAME aet7-49
EXPNO 11
PROCNO 1
F2 - Acquisition Parameters
Date_ 20120106
Time 0.08
INSTRUM DPX400
PROBHD 5 mm QNP 1H/13
PULPROG zgpg30
TD 65536
SOLVENT CDCl3
NS 1024
DS 4
SWH 23980.814 Hz
FIDRES 0.365918 Hz
AQ 1.3664756 sec
RG 1824.6
DW 20.850 usec
DE 6.00 usec
TE 298.2 K
D1 2.00000000 sec
d11 0.03000000 sec
DELTA 1.89999998 sec
TD0 1
======== CHANNEL f1 ========
NUC1 13C
P1 8.00 usec
PL1 -2.60 dB
SFO1 100.6228298 MHz
======== CHANNEL f2 ========
CPDPRG2 waltz16
NUC2 1H
PCPD2 80.00 usec
PL2 -1.50 dB
PL12 17.50 dB
PL13 17.50 dB
SFO2 400.1316005 MHz
F2 - Processing parameters
SI 32768
SF 100.6127690 MHz
WDW EM
SSB 0
LB 1.00 Hz
GB 0
PC 1.40
200 180 160 140 120 100 80 60 40 20 0 ppm

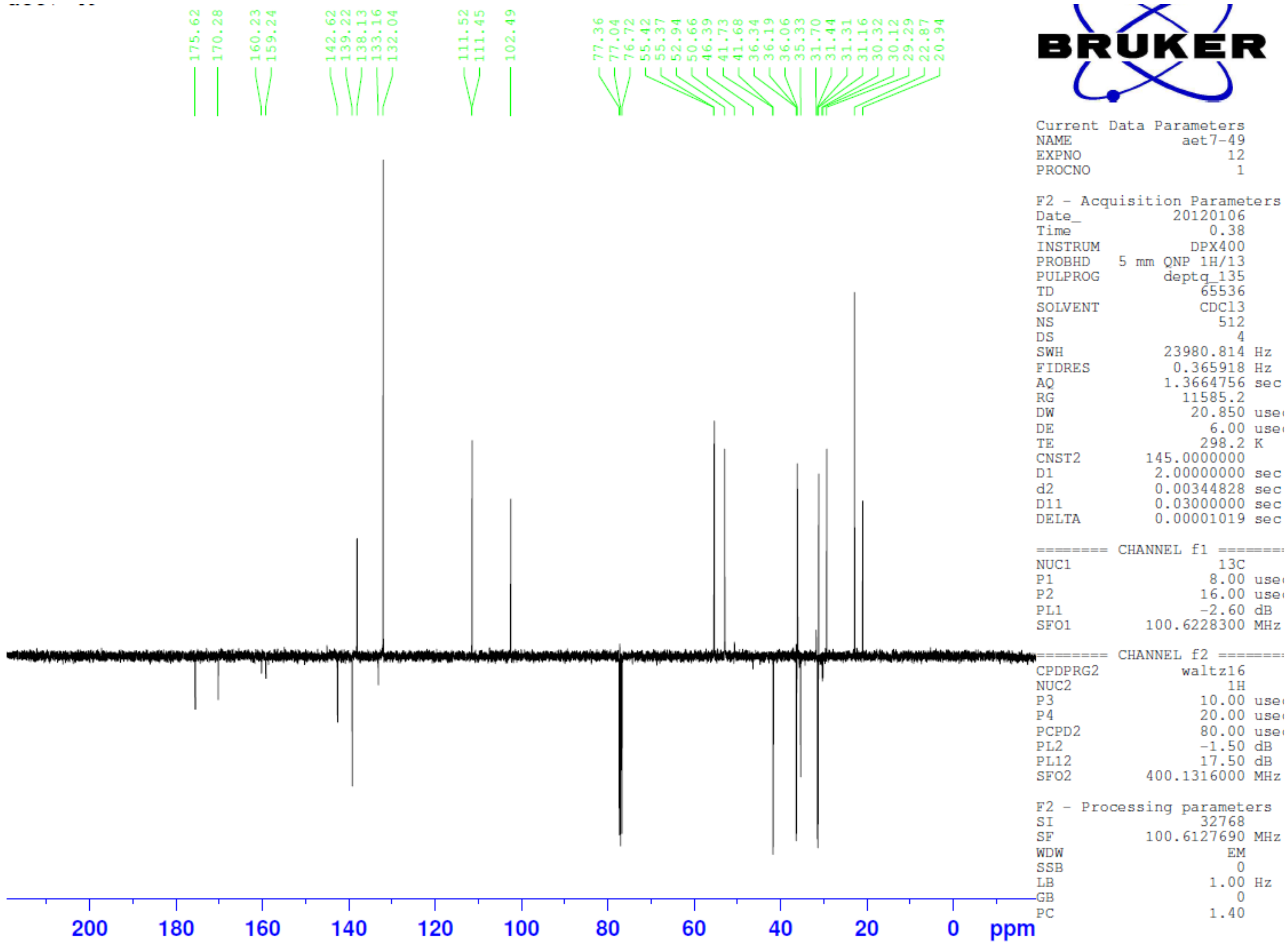
175.62
170.28
160.23
159.24
142.62
139.22
138.13
133.16
132.04
111.52
111.45
102.49
77.36
77.04
76.72
55.42
55.37
52.94
50.66
46.39
41.73
41.68
36.34
36.19
36.06
35.33
31.70
31.44
31.31
31.16
30.32
30.12
29.29
22.87
20.94
BRUKER
Current Data Parameters
NAME aet7-49
EXPNO 12
PROCNO 1
F2 - Acquisition Parameters
Date_ 20120106
Time 0.38
INSTRUM DPX400
PROBHD 5 mm QNP 1H/13
PULPROG deptq_135
TD 65536
SOLVENT CDCl3
NS 512
DS 4
SWH 23980.814 Hz
FIDRES 0.365918 Hz
AQ 1.3664756 sec
RG 11585.2
DW 20.850 usec
DE 6.00 usec
TE 298.2 K
CNST2 145.0000000
D1 2.00000000 sec
d2 0.00344828 sec
D11 0.03000000 sec
DELTA 0.00001019 sec
======== CHANNEL f1 ========
NUC1 13C
P1 8.00 usec
P2 16.00 usec
PL1 -2.60 dB
SFO1 100.6228300 MHz
======== CHANNEL f2 ========
CPDPRG2 waltz16
NUC2 1H
P3 10.00 usec
P4 20.00 usec
PCPD2 80.00 usec
PL2 -1.50 dB
PL12 17.50 dB
SFO2 400.1316000 MHz
F2 - Processing parameters
SI 32768
SF 100.6127690 MHz
WDW EM
SSB 0
LB 1.00 Hz
GB 0
PC 1.40
200 180 160 140 120 100 80 60 40 20 0 ppm

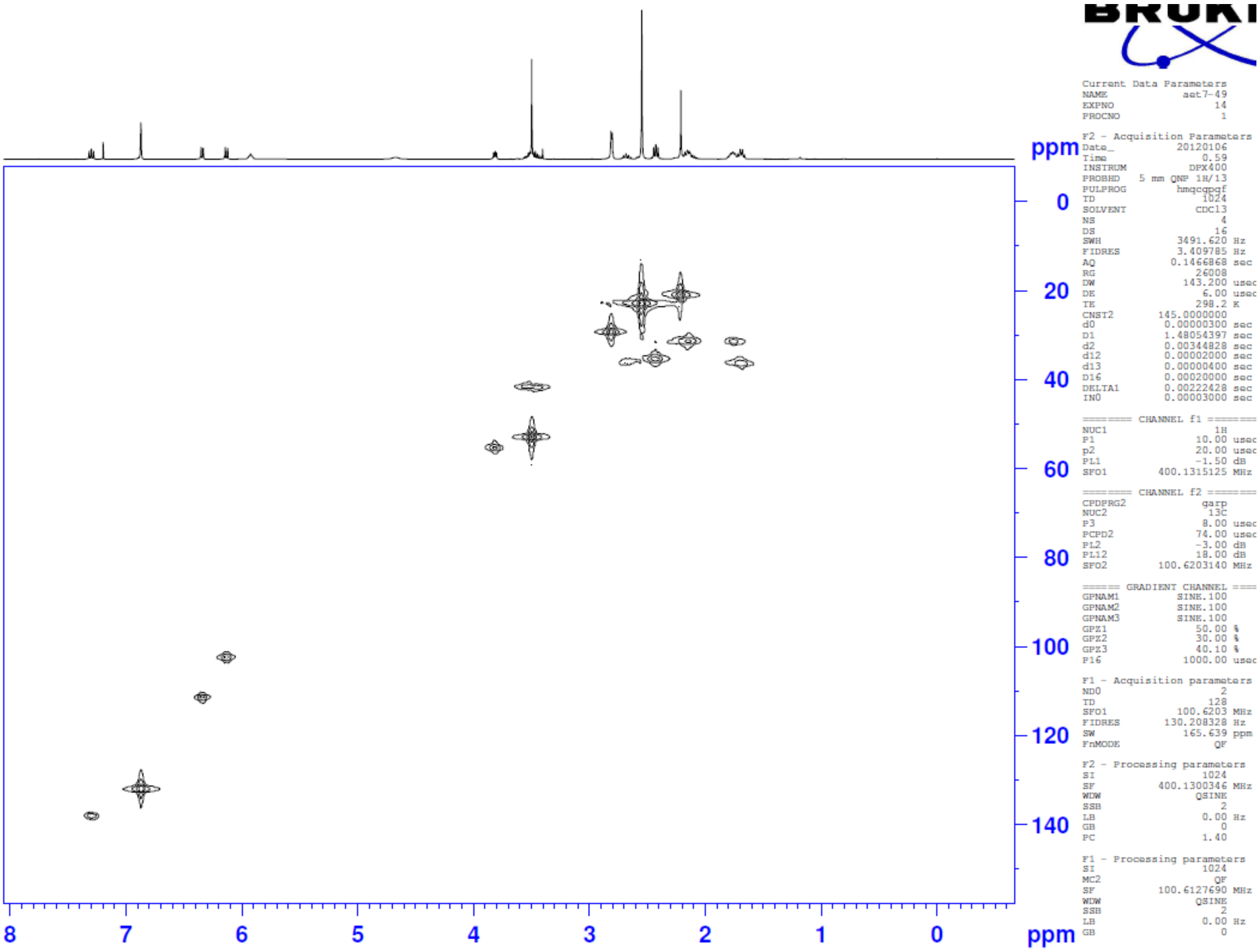

ppm
0
20
40
60
80
100
120
140
8
7
6
5
4
3
2
1
0
ppm
Current Data Parameters
NAME aet7-49
EXPNO 14
PROCNO 1
F2 - Acquisition Parameters
Date_ 20120106
Time 0.59
INSTRUM DPX400
PROBHD 5 mm QNP 1H/13
PULPROG hmqcgpqf
TD 1024
SOLVENT CDCl3
NS 4
DS 16
SWH 3491.620 Hz
FIDRES 3.409785 Hz
AQ 0.1466868 sec
RG 26008
DW 143.200 usec
DE 6.00 usec
TE 298.2 K
CNST2 145.0000000
d0 0.00000300 sec
D1 1.48054397 sec
d2 0.00344828 sec
d12 0.00002000 sec
d13 0.00000400 sec
D16 0.00020000 sec
DELTA1 0.00222428 sec
IN0 0.00003000 sec
======== CHANNEL f1 ========
NUC1 1H
P1 10.00 usec
p2 20.00 usec
PL1 -1.50 dB
SFO1 400.1315125 MHz
======== CHANNEL f2 ========
CPDPRG2 garp
NUC2 13C
P3 8.00 usec
PCPD2 74.00 usec
PL2 -3.00 dB
PL12 18.00 dB
SFO2 100.6203140 MHz
====== GRADIENT CHANNEL =====
GPNAM1 SINE.100
GPNAM2 SINE.100
GPNAM3 SINE.100
GPZ1 50.00 %
GPZ2 30.00 %
GPZ3 40.10 %
P16 1000.00 usec
F1 - Acquisition parameters
ND0 2
TD 128
SFO1 100.6203 MHz
FIDRES 130.208328 Hz
SW 165.639 ppm
FnMODE QF
F2 - Processing parameters
SI 1024
SF 400.1300346 MHz
WDW QSINE
SSB 2
LB 0.00 Hz
GB 0
PC 1.40
F1 - Processing parameters
SI 1024
MC2 QF
SF 100.6127690 MHz
WDW QSINE
SSB 2
LB 0.00 Hz
GB 0

(*S*)-3-((1*r,3*R**)-3-(2-(6-(methylamino)pyridin-2-yl)ethyl)cyclobutane-1-carboxamido)-2-(2,4,6-trimethylphenylsulfonamido)propanoic acid [Compound 55]**

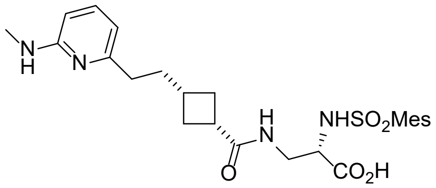

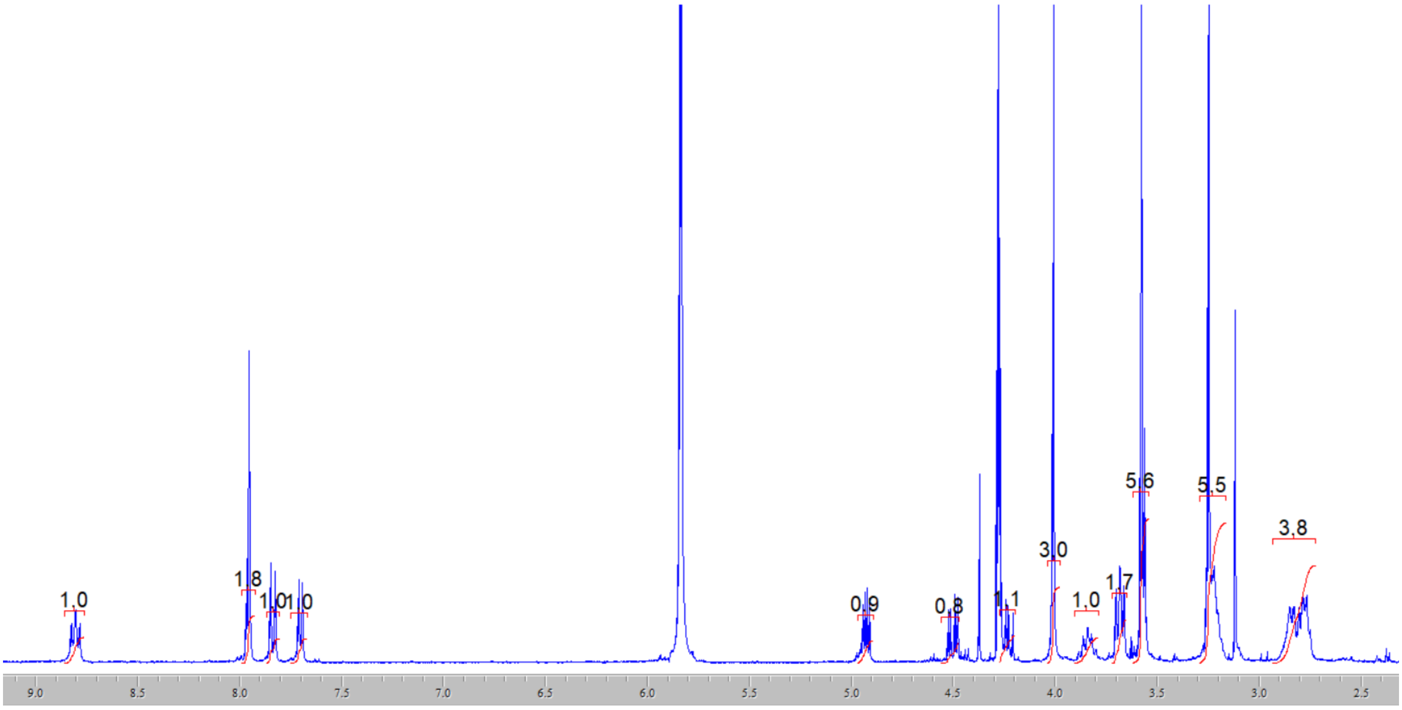

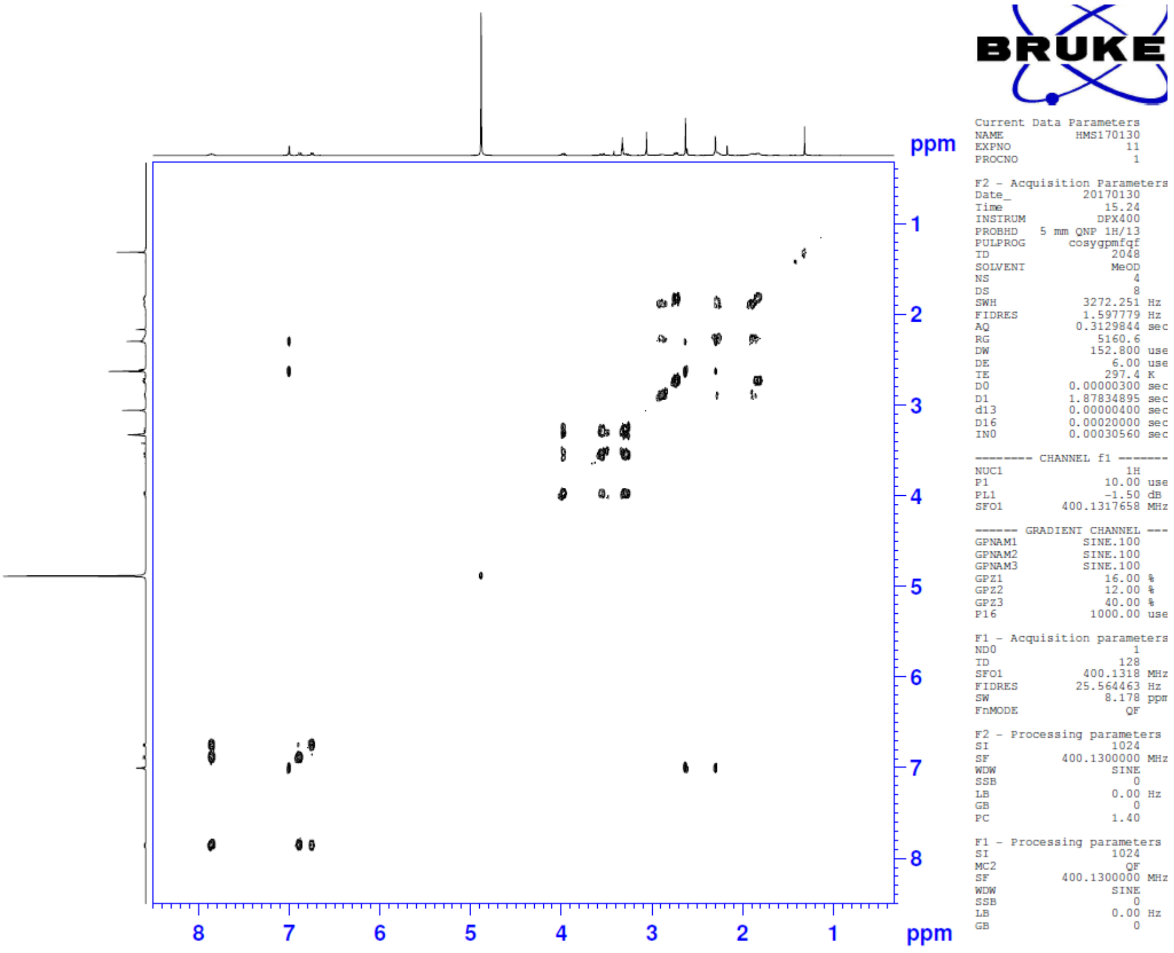

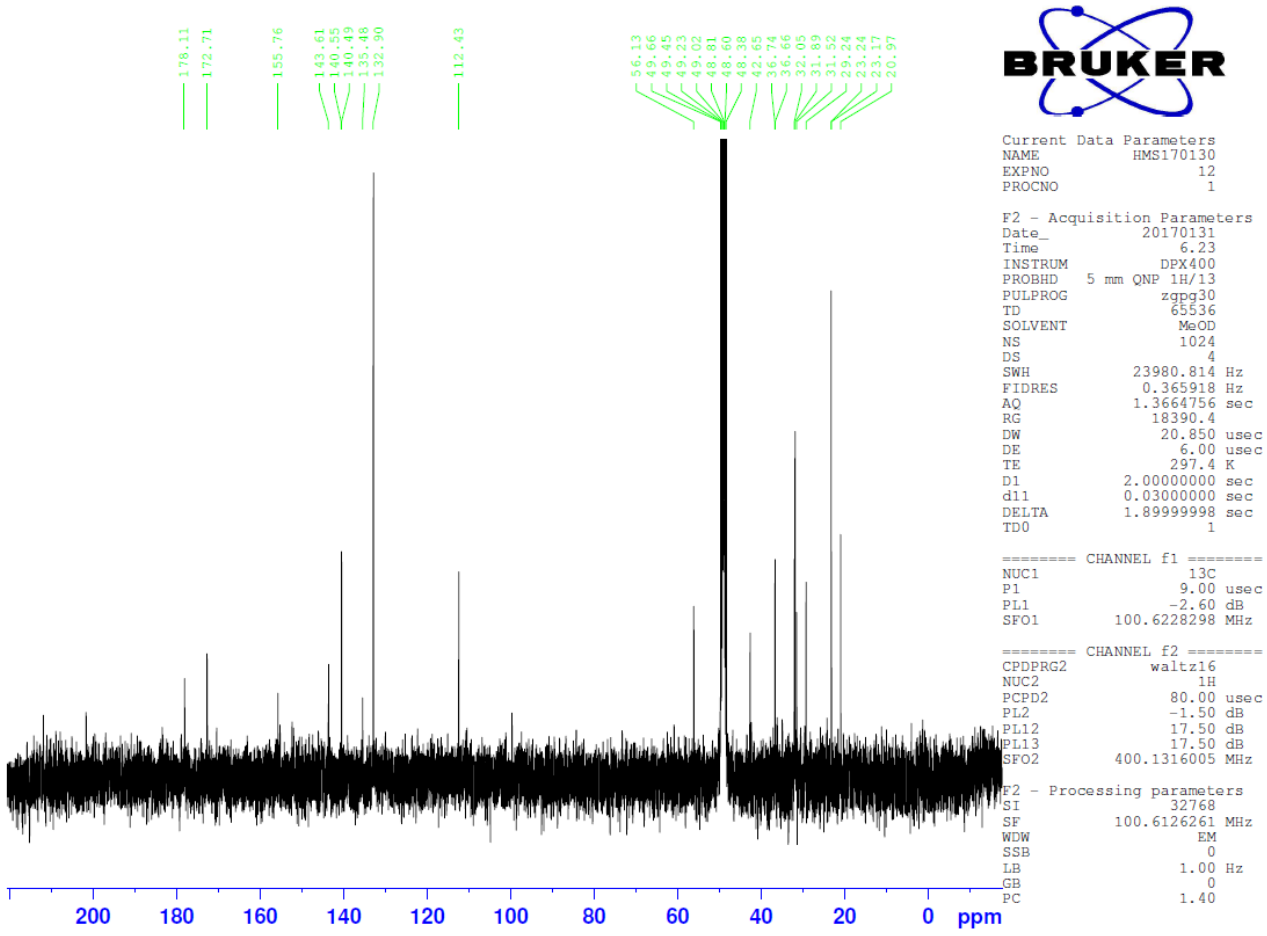
178.11
172.71
155.76
143.61
140.55
140.49
135.48
132.90
112.43
56.13
49.66
49.45
49.23
49.02
48.81
48.60
48.38
42.65
36.74
36.66
32.05
31.89
31.52
29.24
23.24
23.17
20.97
200
180
160
140
120
100
80
60
40
20
0
ppm
Current Data Parameters
NAME HMS170130
EXPNO 12
PROCNO 1
F2 - Acquisition Parameters
Date_ 20170131
Time 6.23
INSTRUM DPX400
PROBHD 5 mm QNP 1H/13
PULPROG zgpg30
TD 65536
SOLVENT MeOD
NS 1024
DS 4
SWH 23980.814 Hz
FIDRES 0.365918 Hz
AQ 1.3664756 sec
RG 18390.4
DW 20.850 usec
DE 6.00 usec
TE 297.4 K
D1 2.00000000 sec
d11 0.03000000 sec
DELTA 1.89999998 sec
TD0 1
======== CHANNEL f1 ========
NUC1 13C
P1 9.00 usec
PL1 -2.60 dB
SFO1 100.6228298 MHz
======== CHANNEL f2 ========
CPDPRG2 waltz16
NUC2 1H
PCPD2 80.00 usec
PL2 -1.50 dB
PL12 17.50 dB
PL13 17.50 dB
SFO2 400.1316005 MHz
F2 - Processing parameters
SI 32768
SF 100.6126261 MHz
WDW EM
SSB 0
LB 1.00 Hz
GB 0
PC 1.40

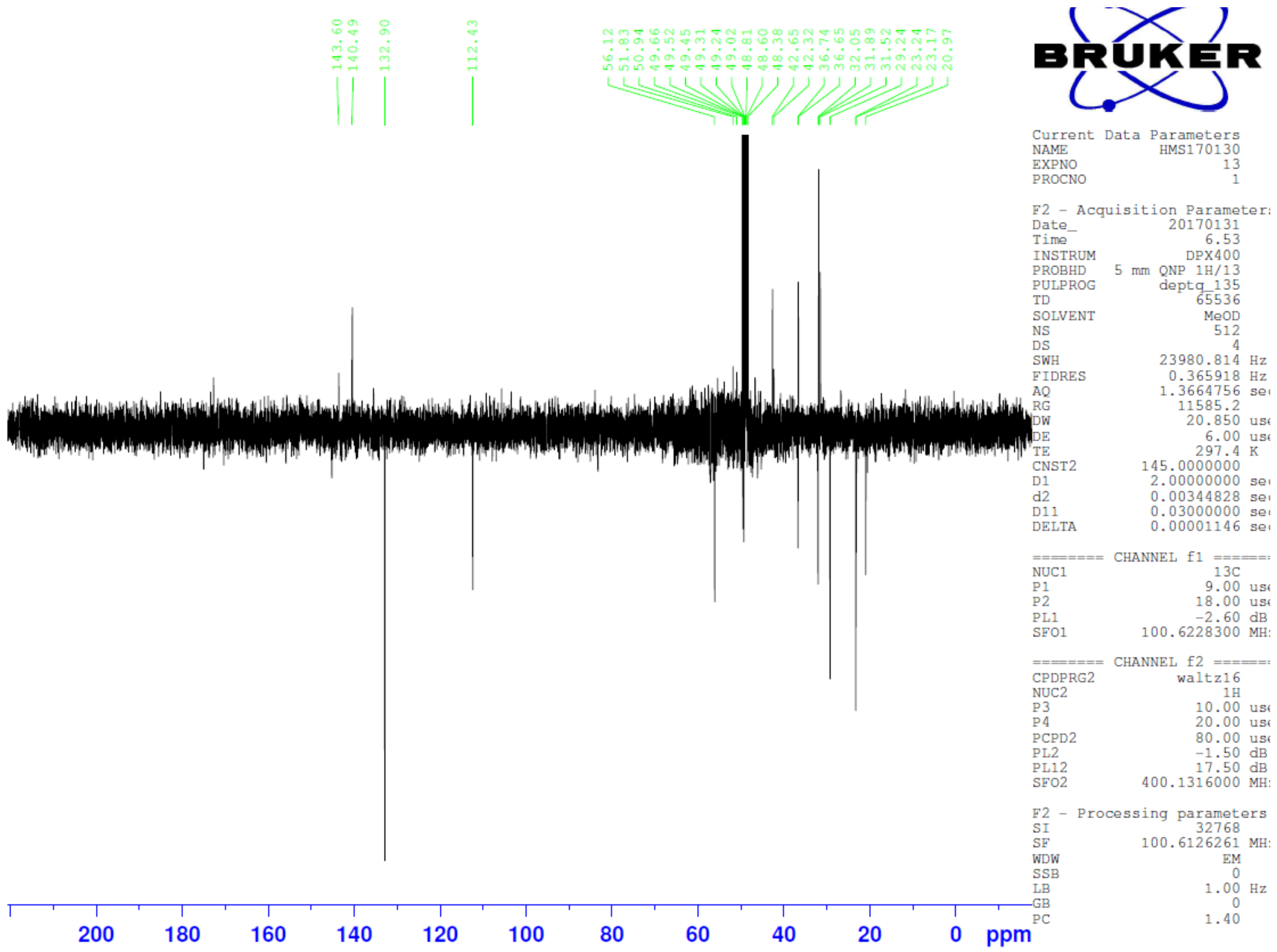
143.60
140.49
132.90
112.43
56.12
51.83
50.94
49.66
49.52
49.45
49.31
49.24
49.02
48.81
48.60
48.38
42.65
42.32
36.74
36.65
32.05
31.89
31.52
29.24
23.24
23.17
20.97
200
180
160
140
120
100
80
60
40
20
0
ppm
Current Data Parameters
NAME HMS170130
EXPNO 13
PROCNO 1
F2 - Acquisition Parameter
Date_ 20170131
Time 6.53
INSTRUM DPX400
PROBHD 5 mm QNP 1H/13
PULPROG deptq_135
TD 65536
SOLVENT MeOD
NS 512
DS 4
SWH 23980.814 Hz
FIDRES 0.365918 Hz
AQ 1.3664756 se
RG 11585.2
DW 20.850 us
DE 6.00 us
TE 297.4 K
CNST2 145.0000000
D1 2.00000000 se
d2 0.00344828 se
D11 0.03000000 se
DELTA 0.00001146 se
======== CHANNEL f1 ======
NUC1 13C
P1 9.00 us
P2 18.00 us
PL1 -2.60 dB
SFO1 100.6228300 MH
======== CHANNEL f2 ======
CPDPRG2 waltz16
NUC2 1H
P3 10.00 us
P4 20.00 us
PCPD2 80.00 us
PL2 -1.50 dB
PL12 17.50 dB
SFO2 400.1316000 MH
F2 - Processing parameters
SI 32768
SF 100.6126261 MH
WDW EM
SSB 0
LB 1.00 Hz
GB 0
PC 1.40

Compound 4

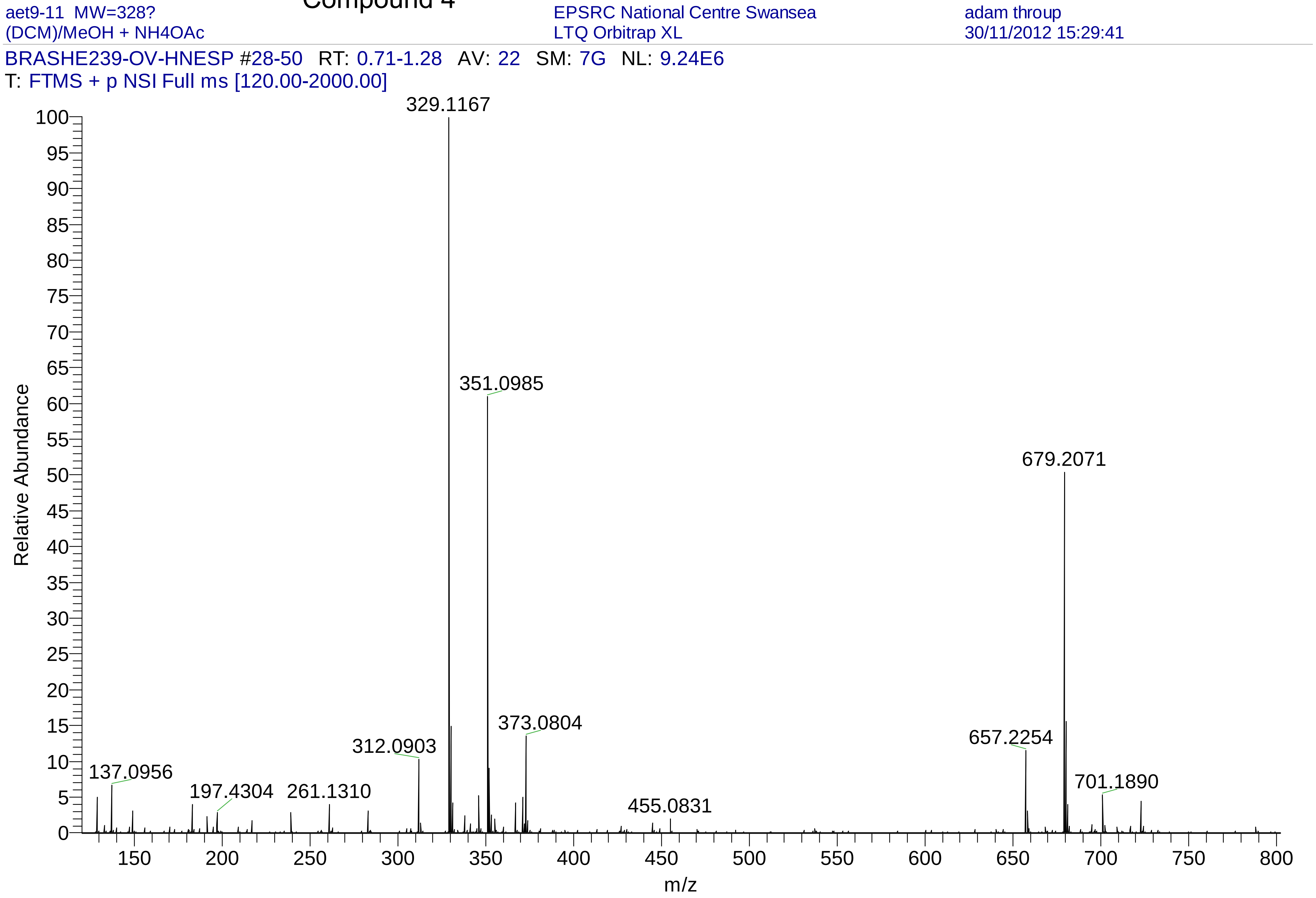
aet9-11 MW=328?
(DCM)/MeOH + NH4OAc
EPSRC National Centre Swansea
LTQ Orbitrap XL
adam throup
30/11/2012 15:29:41
BRASHE239-OV-HNESP #28-50 RT: 0.71-1.28 AV: 22 SM: 7G NL: 9.24E6
T: FTMS + p NSI Full ms [120.00-2000.00]
329.1167
351.0985
679.2071
373.0804
657.2254
312.0903
137.0956
197.4304
261.1310
455.0831
701.1890
Relative Abundance
m/z

aet9-11 MW=328?
(DCM)/MeOH + NH4OAc

EPSRC National Centre Swansea
LTQ Orbitrap XL

adam throup
30/11/2012 15:29:41

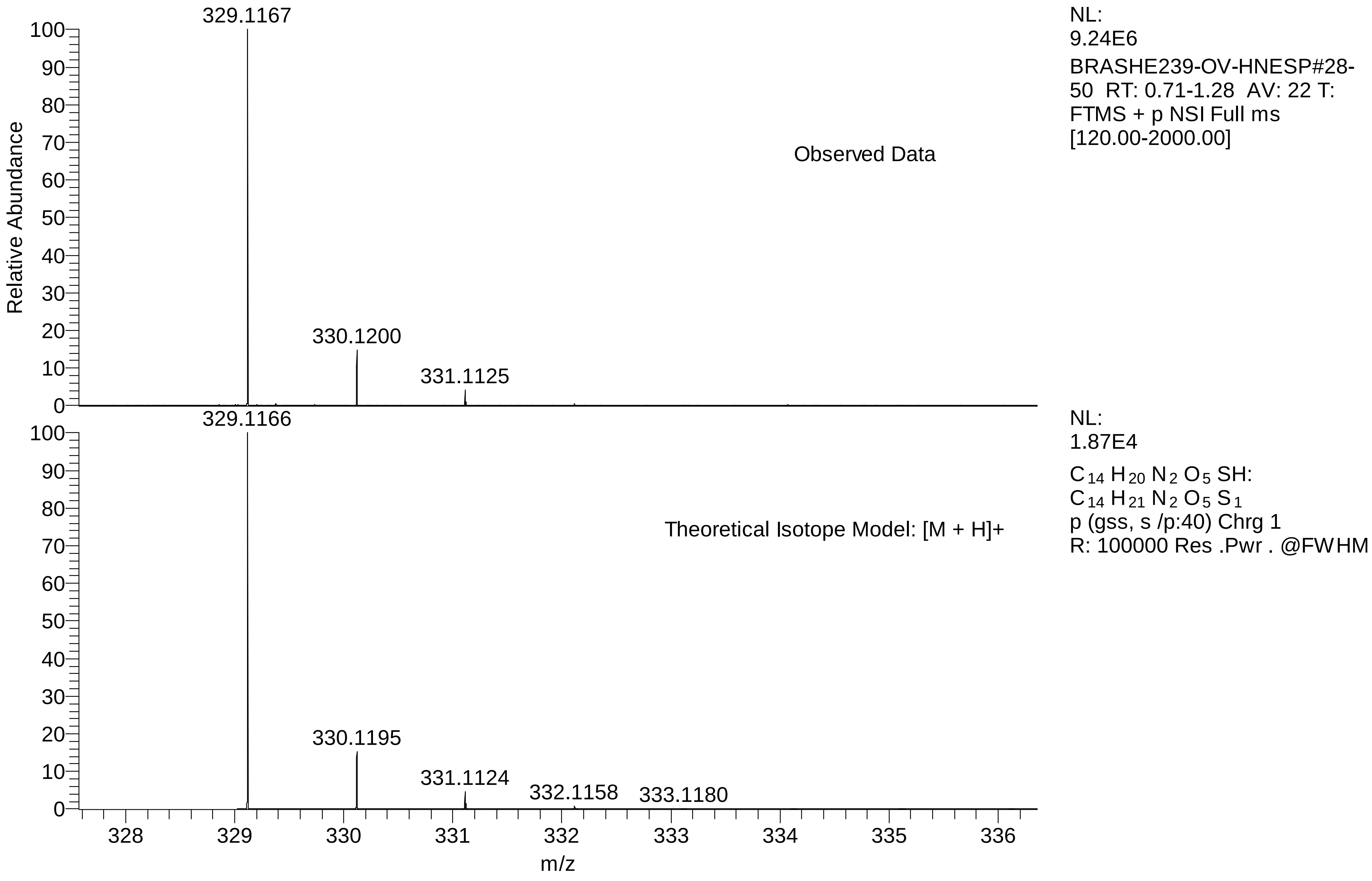

Isotope: **Min. .. Max.**
14 N **0....12**
16 O **0....15**
12 C **0....50**
1 H **0....65**
23 Na **0....0**
32 S **0....4**
Tolerance Window: **+- 5.00 ppm**
Db/Ring Equiv: **-3.. 100** **N-Rule:** **Do not use**
Fits: **100** **Charge:** **1**

Mass	Theoretical Mass	Delta [ppm]	RDB	Composition
329.1167	329.1166	0.4	5.5	$C_{14} H_{21} O_5 N_2 S_1$
	329.1166	0.4	11.0	$C_{13} H_{15} N_9 S_1$
	329.1164	0.9	-3.0	$C_7 H_{23} O_{13} N_1$
	329.1164	1.0	2.5	$C_6 H_{17} O_8 N_8$
	329.1172	-1.6	14.5	$C_{22} H_{17} O_3$
	329.1173	-1.7	1.5	$C_7 H_{21} O_3 N_8 S_2$
	329.1174	-2.2	4.5	$C_{15} H_{25} N_2 S_3$
	329.1159	2.4	2.0	$C_5 H_{19} O_2 N_{11} S_2$
	329.1159	2.5	15.0	$C_{20} H_{15} O_2 N_3$
	329.1177	-3.1	7.5	$C_7 H_{13} O_4 N_{12}$
	329.1177	-3.1	2.0	$C_8 H_{19} O_9 N_5$
	329.1179	-3.7	10.5	$C_{15} H_{17} O_1 N_6 S_1$
	329.1152	4.5	6.0	$C_{12} H_{19} O_4 N_5 S_1$

Compound 5

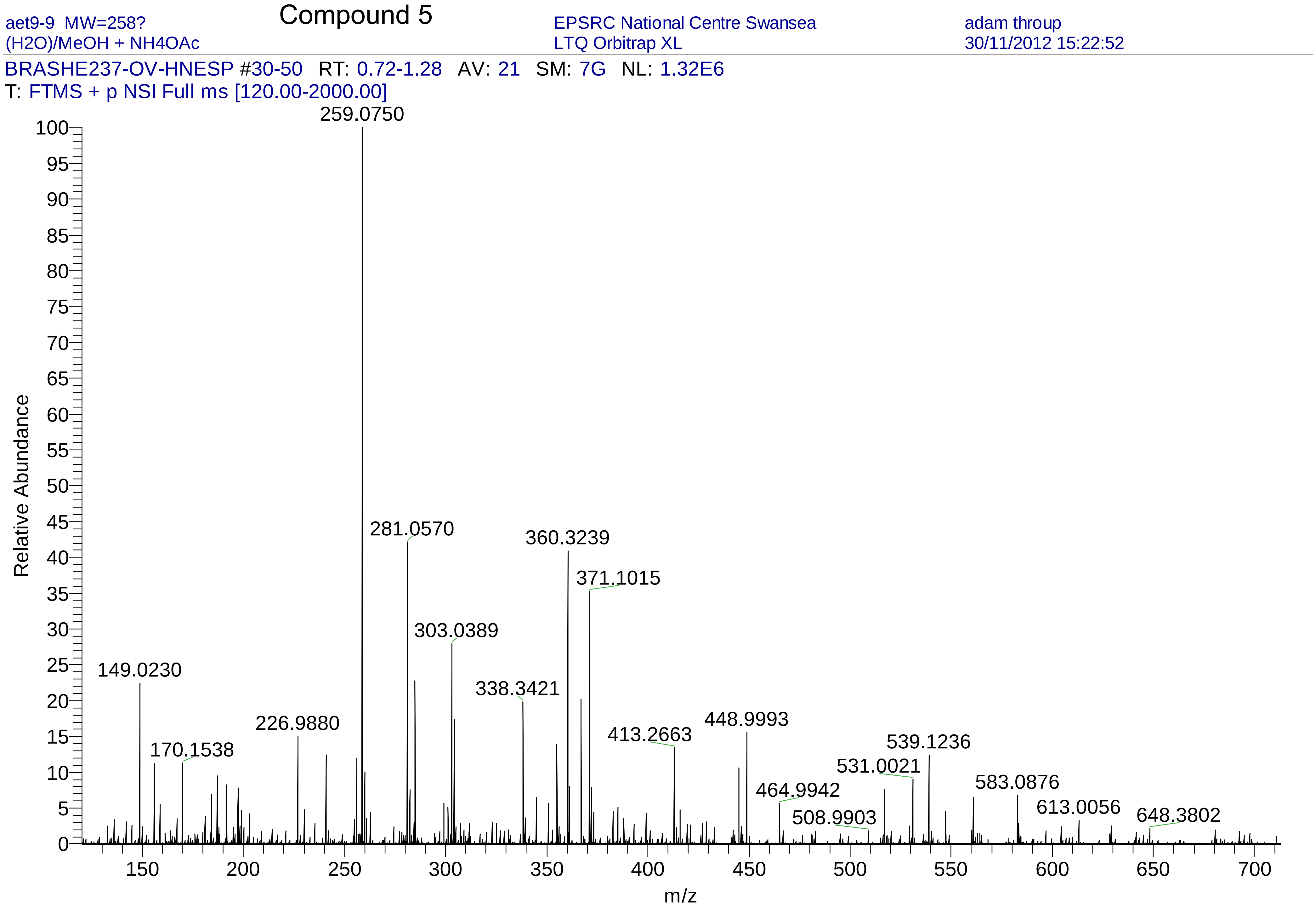
aet9-9 MW=258?
(H2O)/MeOH + NH4OAc
EPSRC National Centre Swansea
LTQ Orbitrap XL
adam throup
30/11/2012 15:22:52
BRASHE237-OV-HNESP #30-50 RT: 0.72-1.28 AV: 21 SM: 7G NL: 1.32E6
T: FTMS + p NSI Full ms [120.00-2000.00]
259.0750
281.0570
360.3239
371.1015
303.0389
149.0230
338.3421
226.9880
170.1538
413.2663
448.9993
539.1236
531.0021
464.9942
583.0876
613.0056
648.3802
508.9903
Relative Abundance
m/z

aet9-9 MW=258?
(H2O)/MeOH + NH4OAc

EPSRC National Centre Swansea
LTQ Orbitrap XL

adam throup
30/11/2012 15:22:52

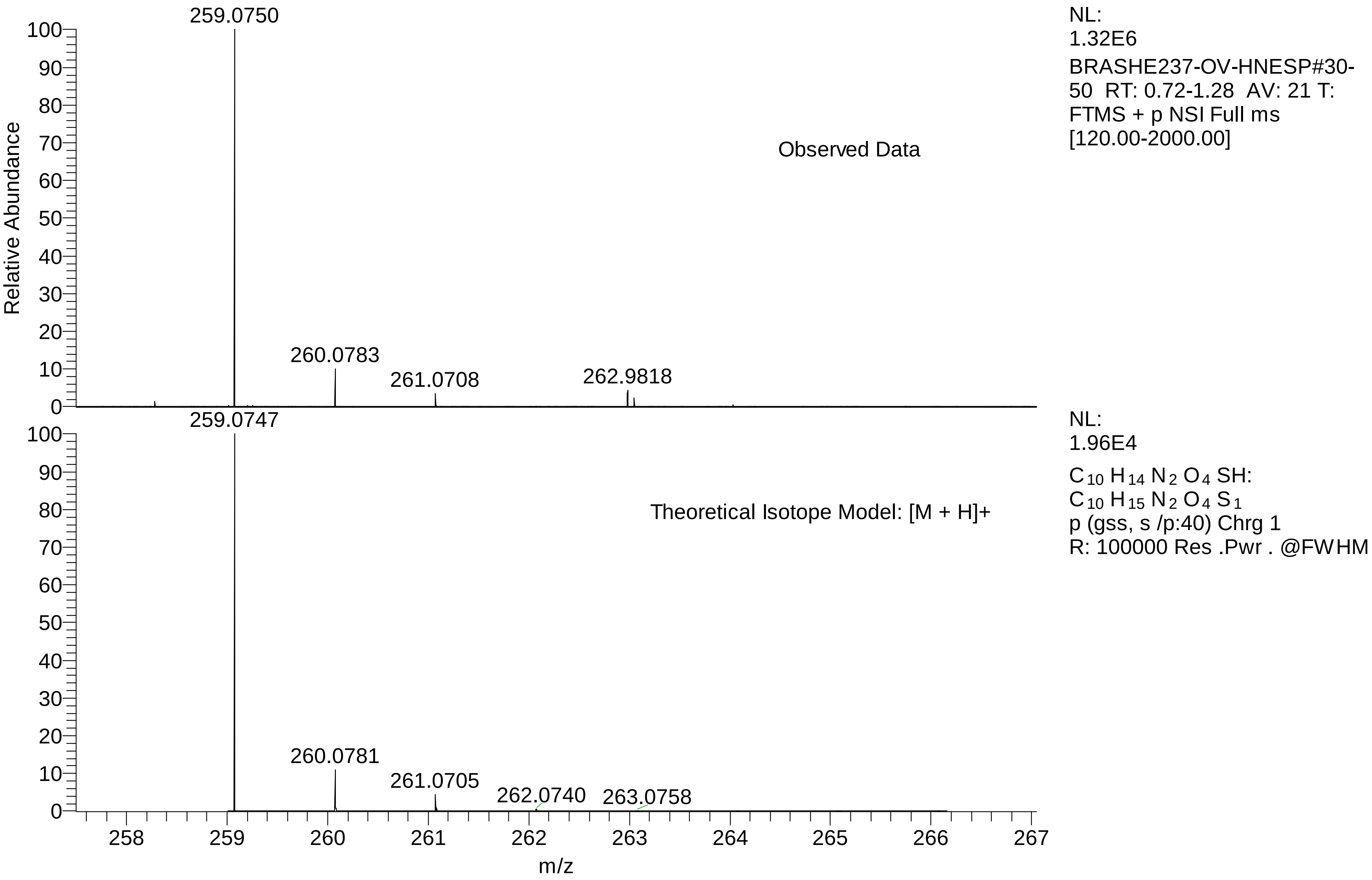

Isotope: **Min. .. Max.**
14 N **0....12**
16 O **0....14**
12 C **0....50**
1 H **0....65**
23 Na **0....0**
32 S **0....4**
Tolerance Window: **+- 5.00 ppm**
Db/Ring Equiv: **-3.. 100** **N-Rule:** **Do not use**
Fits: **100** **Charge:** **1**

**Mass**	**Theoretical Mass**	**Delta [ppm]**	**RDB**	**Composition**
259.0750	259.0747	1.1	4.5	$C_{10} H_{15} O_4 N_2 S_1$
	259.0754	-1.4	13.5	$C_{18} H_{11} O_2$
	259.0754	-1.5	0.5	$C_3 H_{15} O_2 N_8 S_2$
	259.0745	1.8	1.5	$C_2 H_{11} O_7 N_8$
	259.0759	-3.3	6.5	$C_3 H_7 O_3 N_{12}$
	259.0759	-3.3	1.0	$C_4 H_{13} O_8 N_5$
	259.0740	3.7	1.0	$C_1 H_{13} O_1 N_{11} S_2$
	259.0740	3.8	14.0	$C_{16} H_9 O_1 N_3$
	259.0760	-4.0	9.5	$C_{11} H_{11} N_6 S_1$

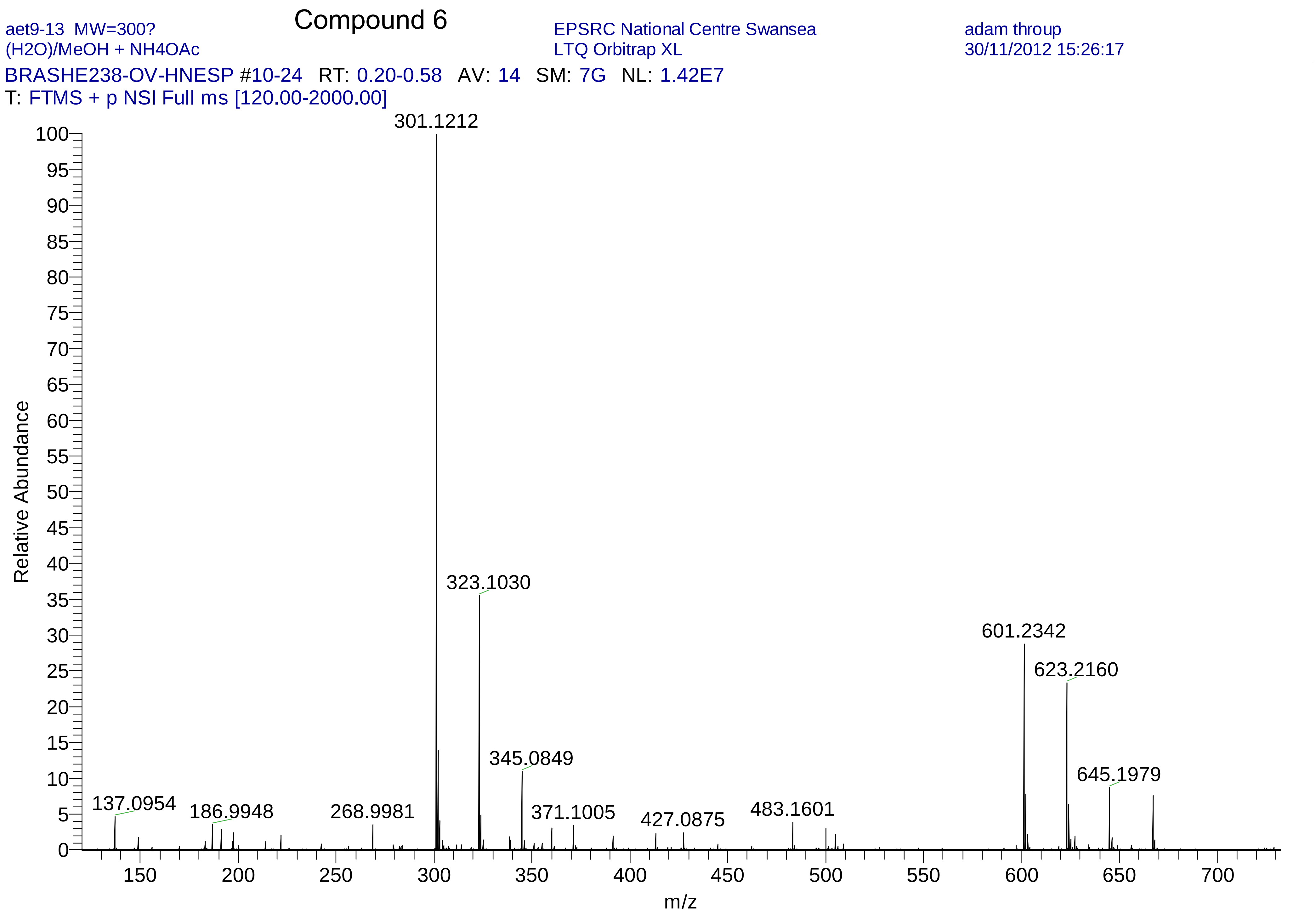
aet9-13 MW=300?
(H2O)/MeOH + NH4OAc
Compound 6
EPSRC National Centre Swansea
LTQ Orbitrap XL
adam throup
30/11/2012 15:26:17
BRASHE238-OV-HNESP #10-24 RT: 0.20-0.58 AV: 14 SM: 7G NL: 1.42E7
T: FTMS + p NSI Full ms [120.00-2000.00]
301.1212
323.1030
601.2342
623.2160
345.0849
645.1979
137.0954
186.9948
268.9981
371.1005
427.0875
483.1601
Relative Abundance
m/z

aet9-13 MW=300?
(H2O)/MeOH + NH4OAc

EPSRC National Centre Swansea
LTQ Orbitrap XL

adam throup
30/11/2012 15:26:17

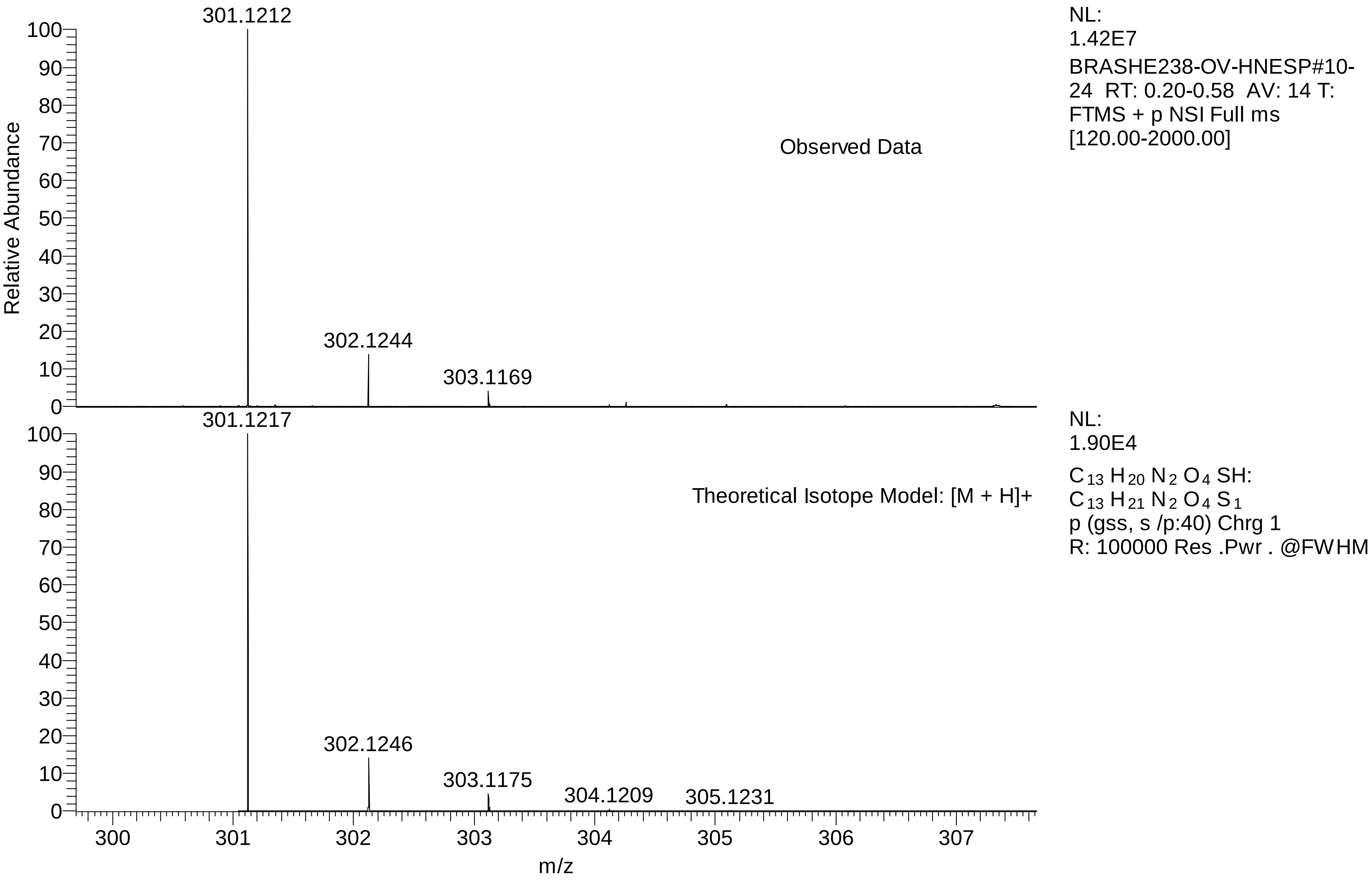

**Isotope:**	**Min. .. Max.**
**14 N**	**0....12**
**16 O**	**0....14**
**12 C**	**0....50**
**1 H**	**0....65**
**23 Na**	**0....0**
**32 S**	**0....4**

**Tolerance Window:**		**+- 5.00 ppm**	
**Db/Ring Equiv:**	**-3.. 100**	**N-Rule:**	**Do not use**
**Fits:**	**100**	**Charge:**	**1**

**Mass**	**Theoretical Mass**	**Delta [ppm]**	**RDB**	**Composition**
301.1212	301.1210	0.7	1.0	$C_4 H_{19} O_1 N_{11} S_2$
	301.1210	0.8	14.0	$C_{19} H_{15} O_1 N_3$
	301.1215	-0.9	1.5	$C_5 H_{17} O_7 N_8$
	301.1217	-1.5	4.5	$C_{13} H_{21} O_4 N_2 S_1$
	301.1203	3.0	5.0	$C_{11} H_{19} O_3 N_5 S_1$
	301.1201	3.6	2.0	$C_3 H_{15} O_6 N_{11}$
	301.1223	-3.7	13.5	$C_{21} H_{17} O_2$
	301.1223	-3.8	0.5	$C_6 H_{21} O_2 N_8 S_2$
	301.1198	4.5	-1.0	$C_{11} H_{27} O_2 N_1 S_3$

Compound 8

aet9-16 MW=314?
(MeOH)/MeOH + NH4OAc

EPSRC National Centre Swansea
LTQ Orbitrap XL

adam throup
30/11/2012 15:19:26

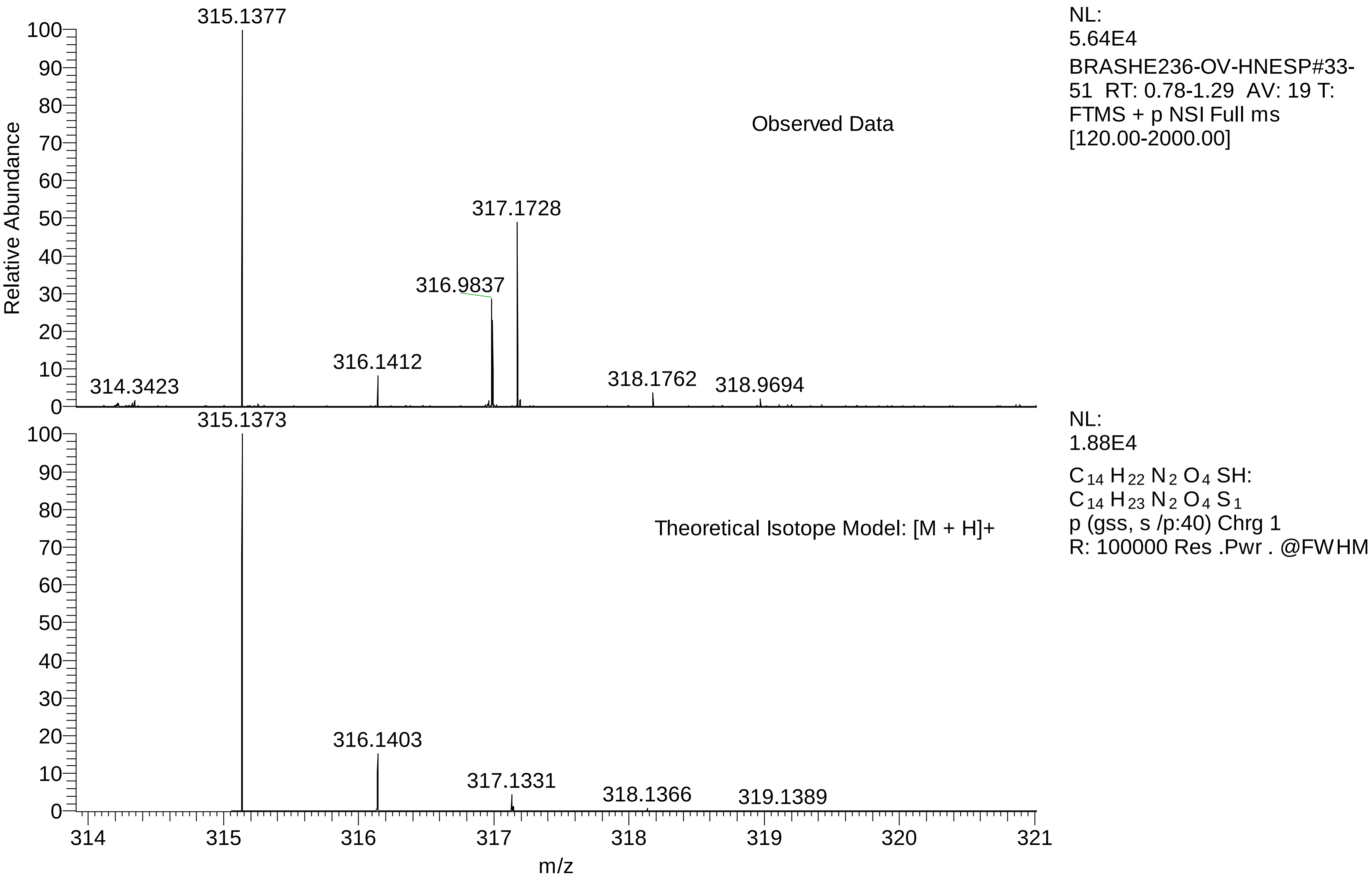

Isotope:	Min. .. Max.
14 N	0....12
16 O	0....14
12 C	0....50
1 H	0....65
23 Na	0....0
32 S	0....4

Tolerance Window: **+- 5.00 ppm**

Db/Ring Equiv: -3.. 100 **N-Rule: Do not use**

Fits: 100 **Charge: 1**

Mass	Theoretical Mass	Delta [ppm]	RDB	Composition
315.1377	315.1380	-0.8	13.5	$C_{22} H_{19} O_2$
	315.1380	-0.9	0.5	$C_7 H_{23} O_2 N_8 S_2$
	315.1373	1.3	4.5	$C_{14} H_{23} O_4 N_2 S_1$
	315.1371	1.8	1.5	$C_6 H_{19} O_7 N_8$
	315.1385	-2.4	6.5	$C_7 H_{15} O_3 N_{12}$
	315.1385	-2.4	1.0	$C_8 H_{21} O_8 N_5$
	315.1386	-3.0	9.5	$C_{15} H_{19} N_6 S_1$
	315.1366	3.3	1.0	$C_5 H_{21} O_1 N_{11} S_2$
	315.1366	3.4	14.0	$C_{20} H_{17} O_1 N_3$
	315.1391	-4.6	-3.0	$C_1 H_{21} O_6 N_{11} S_1$

Compound 17

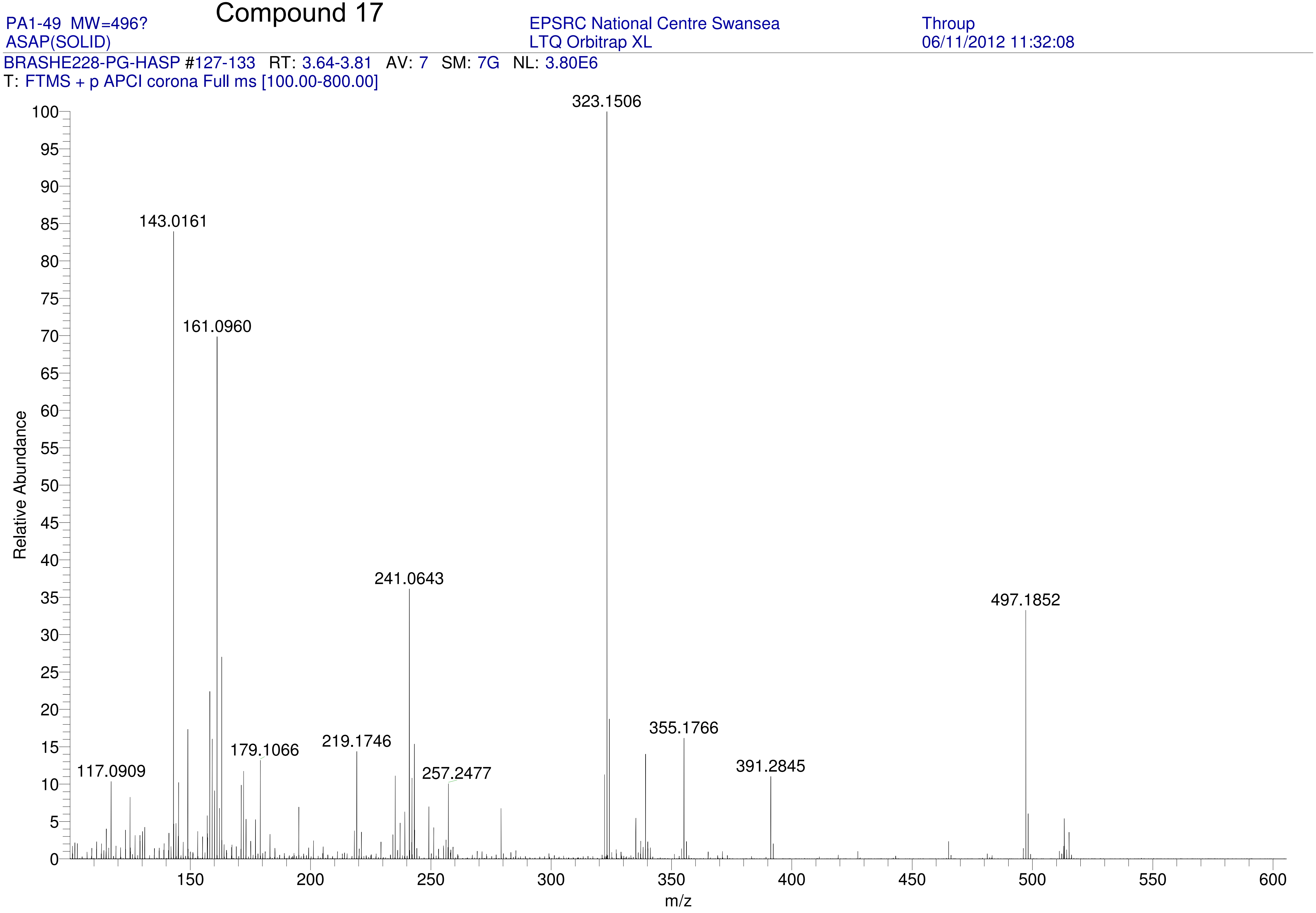

PA1-49 MW=496?
ASAP(SOLID)
EPSRC National Centre Swansea
LTQ Orbitrap XL
Throup
06/11/2012 11:32:08
BRASHE228-PG-HASP #127-133 RT: 3.64-3.81 AV: 7 SM: 7G NL: 3.80E6
T: FTMS + p APCI corona Full ms [100.00-800.00]
Relative Abundance
m/z
323.1506
143.0161
161.0960
241.0643
497.1852
355.1766
179.1066
219.1746
117.0909
257.2477
391.2845

PA1-49 MW=496? EPSRC National Centre Swansea Throup
ASAP(SOLID) LTQ Orbitrap XL 06/11/2012 11:32:08

BRASHE228-PG-HASP #127-133 RT: 3.64-3.81 AV: 7 SM: 7G NL: 3.80E6
T: FTMS + p APCI corona Full ms [100.00-800.00]

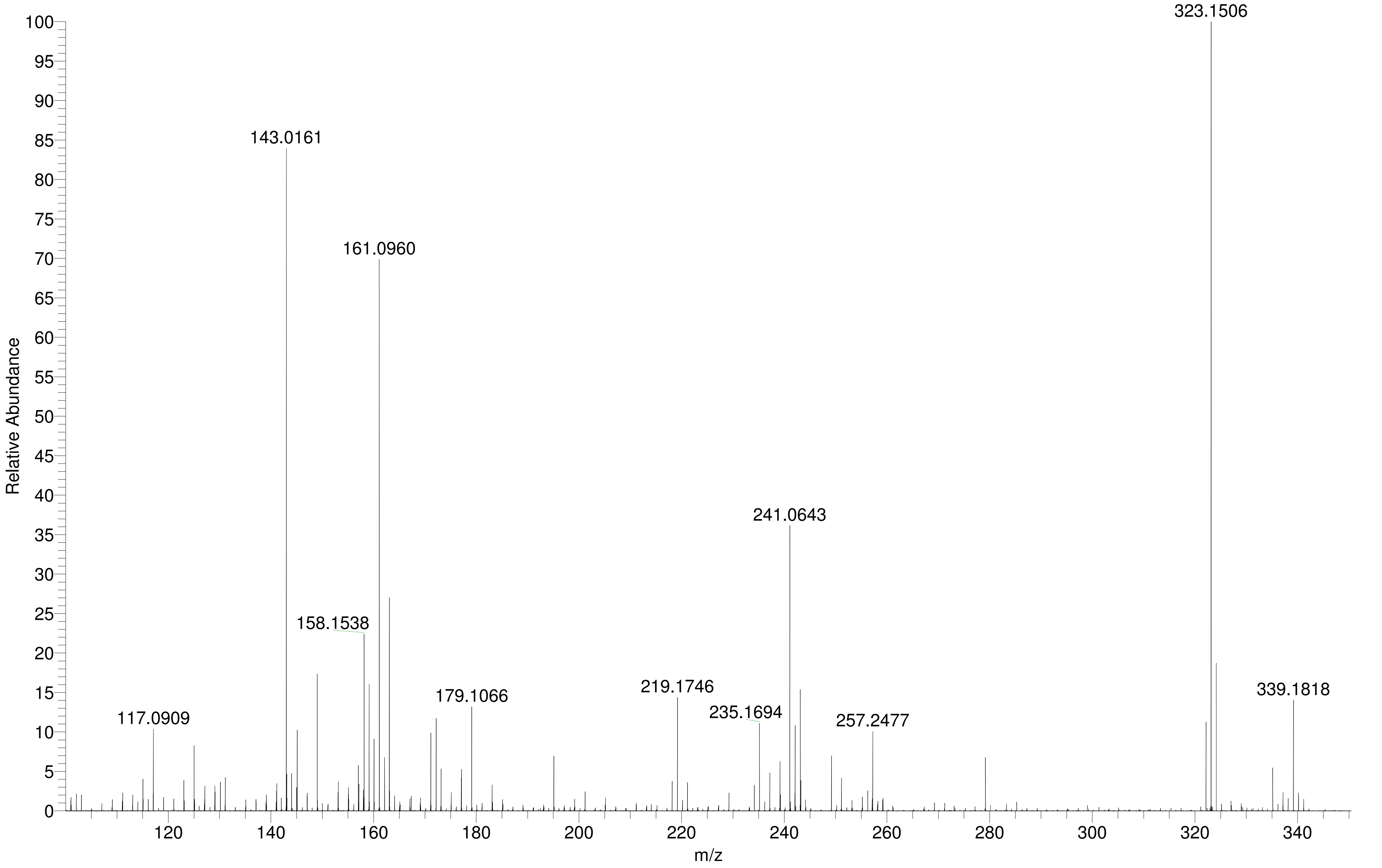

PA1-49 MW=496?
ASAP(SOLID)

EPSRC National Centre Swansea
LTQ Orbitrap XL

Throup
06/11/2012 11:32:08

BRASHE228-PG-HASP #127-133 RT: 3.64-3.81 AV: 7 SM: 7G NL: 1.26E6
T: FTMS + p APCI corona Full ms [100.00-800.00]

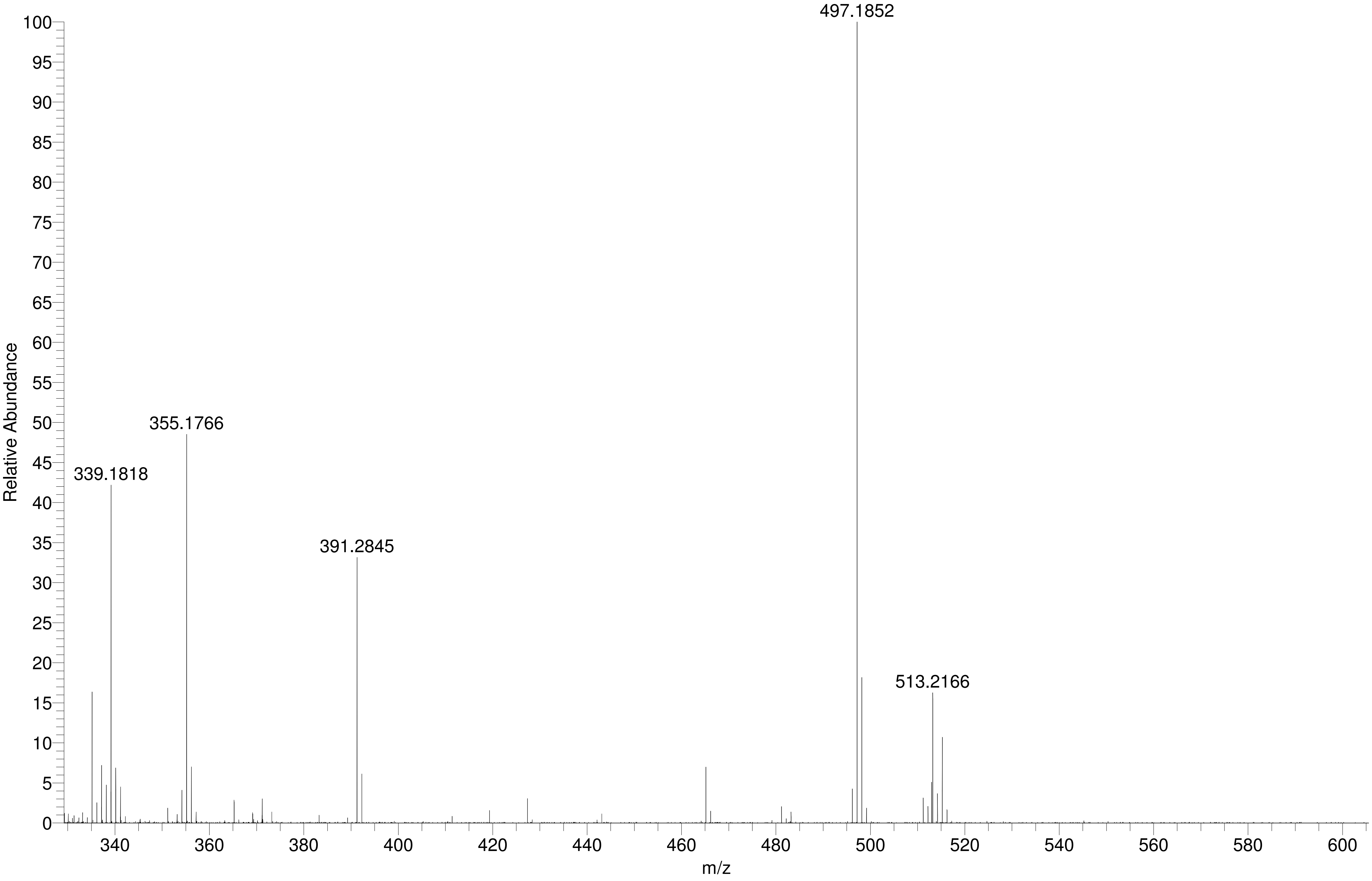

PA1-49 MW=496? EPSRC National Centre Swansea Throup
ASAP(SOLID) LTQ Orbitrap XL 06/11/2012 11:32:08
SM: 7G

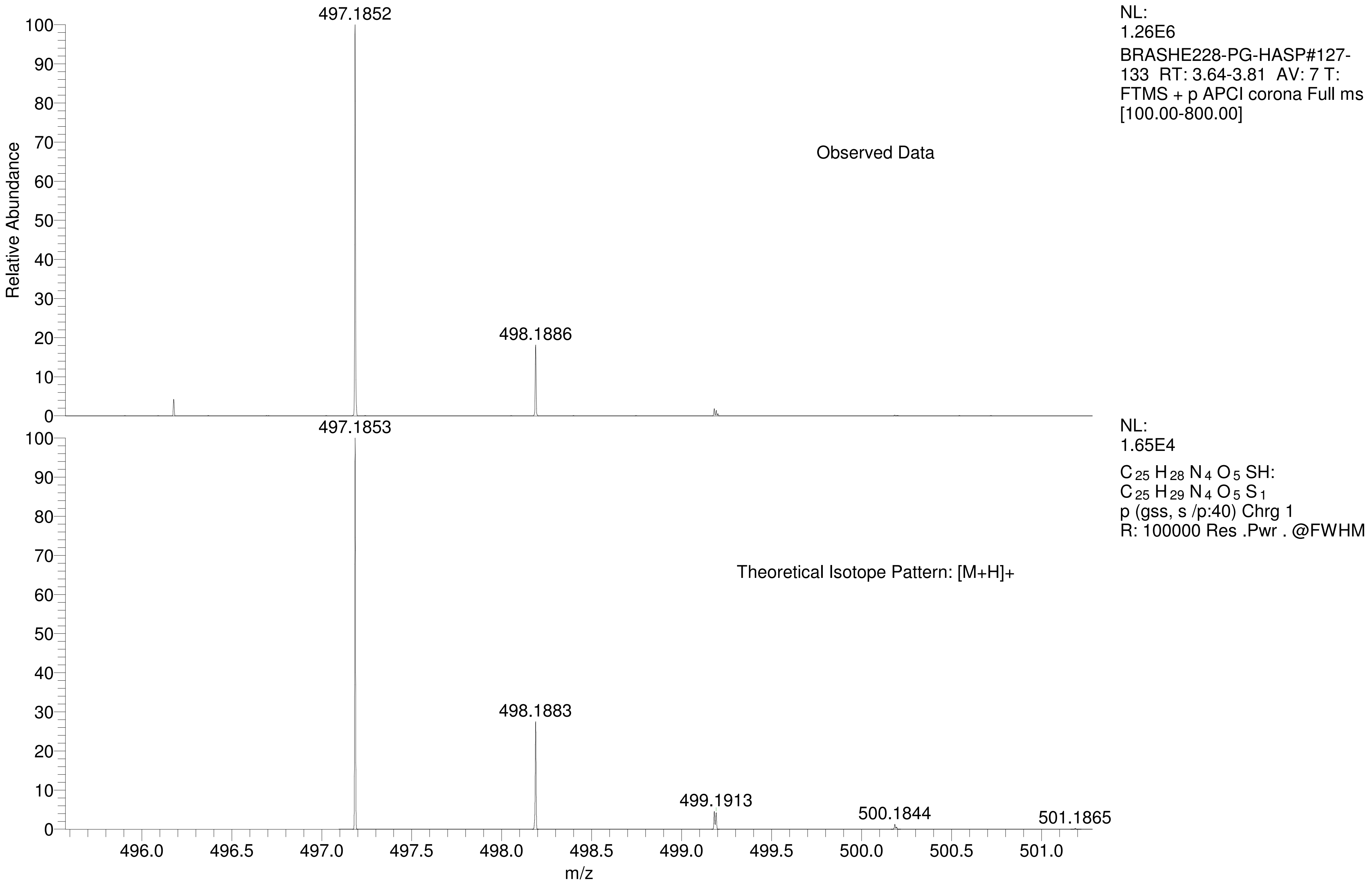

**Isotope:**	**Min. .. Max.**		
**14 N**	**0....16**		
**16 O**	**0....15**		
**12 C**	**0....80**		
**1 H**	**0....100**		
**23 Na**	**0....0**		
**32 S**	**0....3**		
**Tolerance Window:**	**+- 5.00 ppm**		
**Db/Ring Equiv:**	**-3.. 100**	**N-Rule:**	**Do not use**
**Fits:**	**100**	**Charge:**	**1**

**Mass**	**Theoretical Mass**	**Delta [ppm]**	**RDB**	**Composition**
497.1852	497.1851	0.1	5.0	$C_{18} H_{31} O_{13} N_{3}$
	497.1851	0.1	10.5	$C_{17} H_{25} O_{8} N_{10}$
	497.1853	-0.2	19.0	$C_{24} H_{23} N_{11} S_{1}$
	497.1853	-0.2	13.5	$C_{25} H_{29} O_{5} N_{4} S_{1}$
	497.1854	-0.3	0.5	$C_{10} H_{33} O_{5} N_{12} S_{3}$
	497.1848	0.7	7.5	$C_{25} H_{37} O_{4} S_{3}$
	497.1856	-0.9	-2.0	$C_{3} H_{27} O_{14} N_{15}$
	497.1847	1.1	4.5	$C_{17} H_{33} O_{7} N_{6} S_{2}$
	497.1847	1.1	10.0	$C_{16} H_{27} O_{2} N_{13} S_{2}$
	497.1846	1.2	23.0	$C_{31} H_{23} O_{2} N_{5}$
	497.1858	-1.2	6.5	$C_{10} H_{25} O_{6} N_{16} S_{1}$
	497.1858	-1.3	1.0	$C_{11} H_{31} O_{11} N_{9} S_{1}$
	497.1845	1.4	1.5	$C_{9} H_{29} O_{10} N_{12} S_{1}$
	497.1860	-1.5	22.5	$C_{33} H_{25} O_{3} N_{2}$
	497.1860	-1.6	9.5	$C_{18} H_{29} O_{3} N_{10} S_{2}$
	497.1860	-1.6	4.0	$C_{19} H_{35} O_{8} N_{3} S_{2}$
	497.1862	-2.0	12.5	$C_{26} H_{33} N_{4} S_{3}$
	497.1842	2.1	17.0	$C_{31} H_{31} O_{1} N_{1} S_{2}$
	497.1840	2.4	1.0	$C_{8} H_{31} O_{4} N_{15} S_{3}$
	497.1840	2.5	8.5	$C_{24} H_{33} O_{9} S_{1}$
	497.1840	2.5	14.0	$C_{23} H_{27} O_{4} N_{7} S_{1}$
	497.1865	-2.6	15.5	$C_{18} H_{21} O_{4} N_{14}$
	497.1865	-2.6	10.0	$C_{19} H_{27} O_{9} N_{7}$
	497.1865	-2.6	4.5	$C_{20} H_{33} O_{14}$
	497.1865	-2.6	-3.0	$C_{4} H_{31} O_{9} N_{15} S_{2}$
	497.1838	2.8	5.5	$C_{16} H_{29} O_{12} N_{6}$
	497.1838	2.8	11.0	$C_{15} H_{23} O_{7} N_{13}$
	497.1867	-2.9	18.5	$C_{26} H_{25} O_{1} N_{8} S_{1}$
	497.1867	-2.9	13.0	$C_{27} H_{31} O_{6} N_{1} S_{1}$
	497.1867	-3.0	5.5	$C_{11} H_{29} O_{1} N_{16} S_{3}$
	497.1867	-3.0	0.0	$C_{12} H_{35} O_{6} N_{9} S_{3}$

Mass	Theoretical Mass	Delta [ppm]	RDB	Composition
	497.1835	3.4	8.0	$C_{23} H_{35} O_3 N_3 S_3$
	497.1870	-3.6	-2.5	$C_5 H_{29} O_{15} N_{12}$
	497.1833	3.8	-0.5	$C_{16} H_{37} O_{11} N_2 S_2$
	497.1833	3.8	5.0	$C_{15} H_{31} O_6 N_9 S_2$
	497.1833	3.8	10.5	$C_{14} H_{25} O_1 N_{16} S_2$
	497.1833	3.8	18.0	$C_{30} H_{27} O_6 N_1$
	497.1833	3.9	23.5	$C_{29} H_{21} O_1 N_8$
	497.1872	-3.9	6.0	$C_{12} H_{27} O_7 N_{13} S_1$
	497.1872	-4.0	0.5	$C_{13} H_{33} O_{12} N_6 S_1$
	497.1831	4.1	2.0	$C_7 H_{27} O_9 N_{15} S_1$
	497.1873	-4.3	9.0	$C_{20} H_{31} O_4 N_7 S_2$
	497.1874	-4.3	3.5	$C_{21} H_{37} O_9 S_2$
	497.1875	-4.7	12.0	$C_{28} H_{35} O_1 N_1 S_3$
	497.1828	4.8	17.5	$C_{29} H_{29} N_4 S_2$

Compound 18

PA1-47 MW=538?
ASAP(DCM)

EPSRC National Centre Swansea
LTQ Orbitrap XL

Throup
06/11/2012 11:41:24

BRASHE229-PG-HASP #34-36 RT: 0.95-1.01 AV: 3 SM: 7G NL: 1.17E7
T: FTMS + p APCI corona Full ms [100.00-800.00]

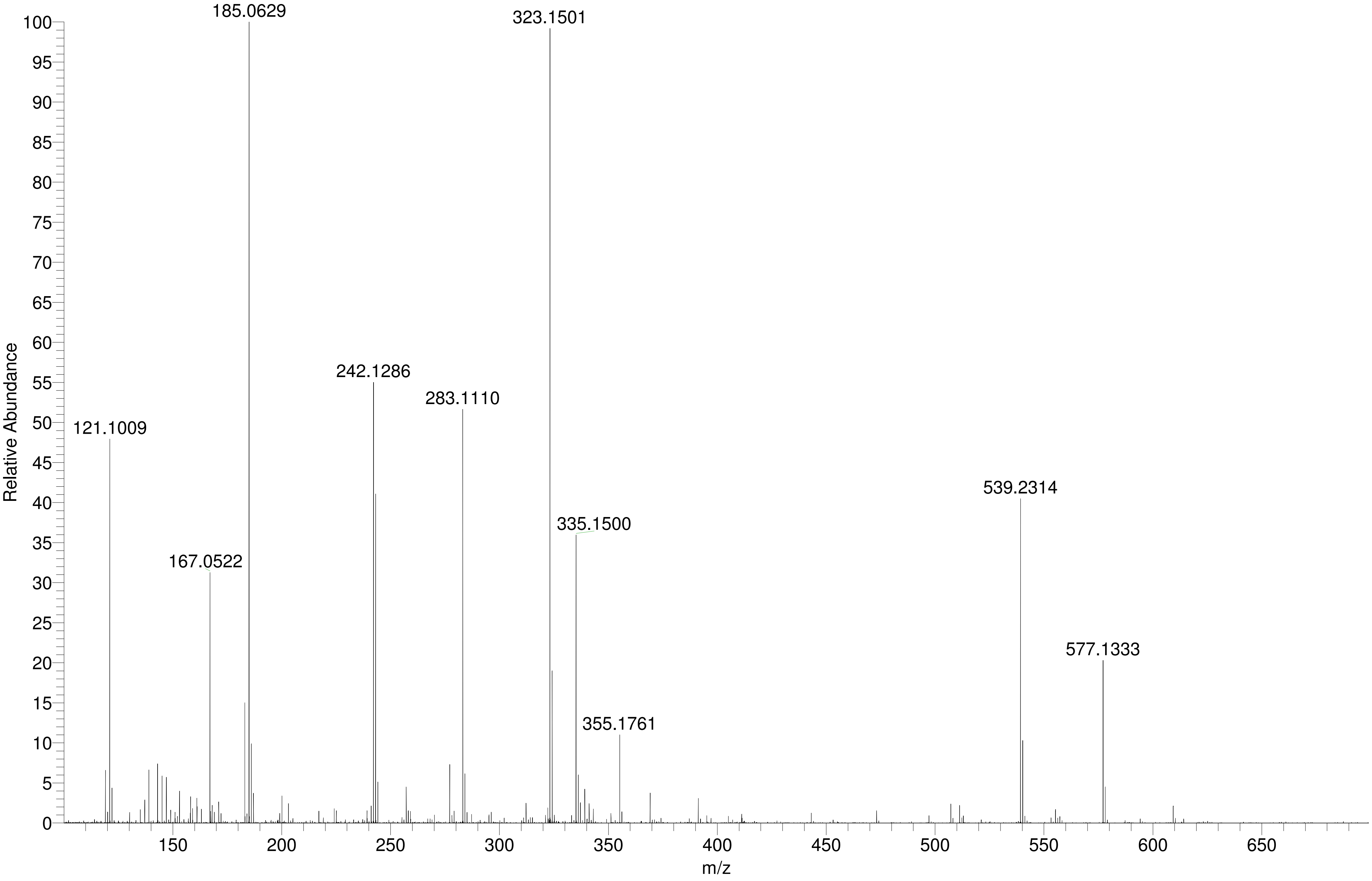

PA1-47 MW=538?
ASAP(DCM)

EPSRC National Centre Swansea
LTQ Orbitrap XL

Throup
06/11/2012 11:41:24

BRASHE229-PG-HASP #34-36 RT: 0.95-1.01 AV: 3 SM: 7G NL: 1.17E7
T: FTMS + p APCI corona Full ms [100.00-800.00]

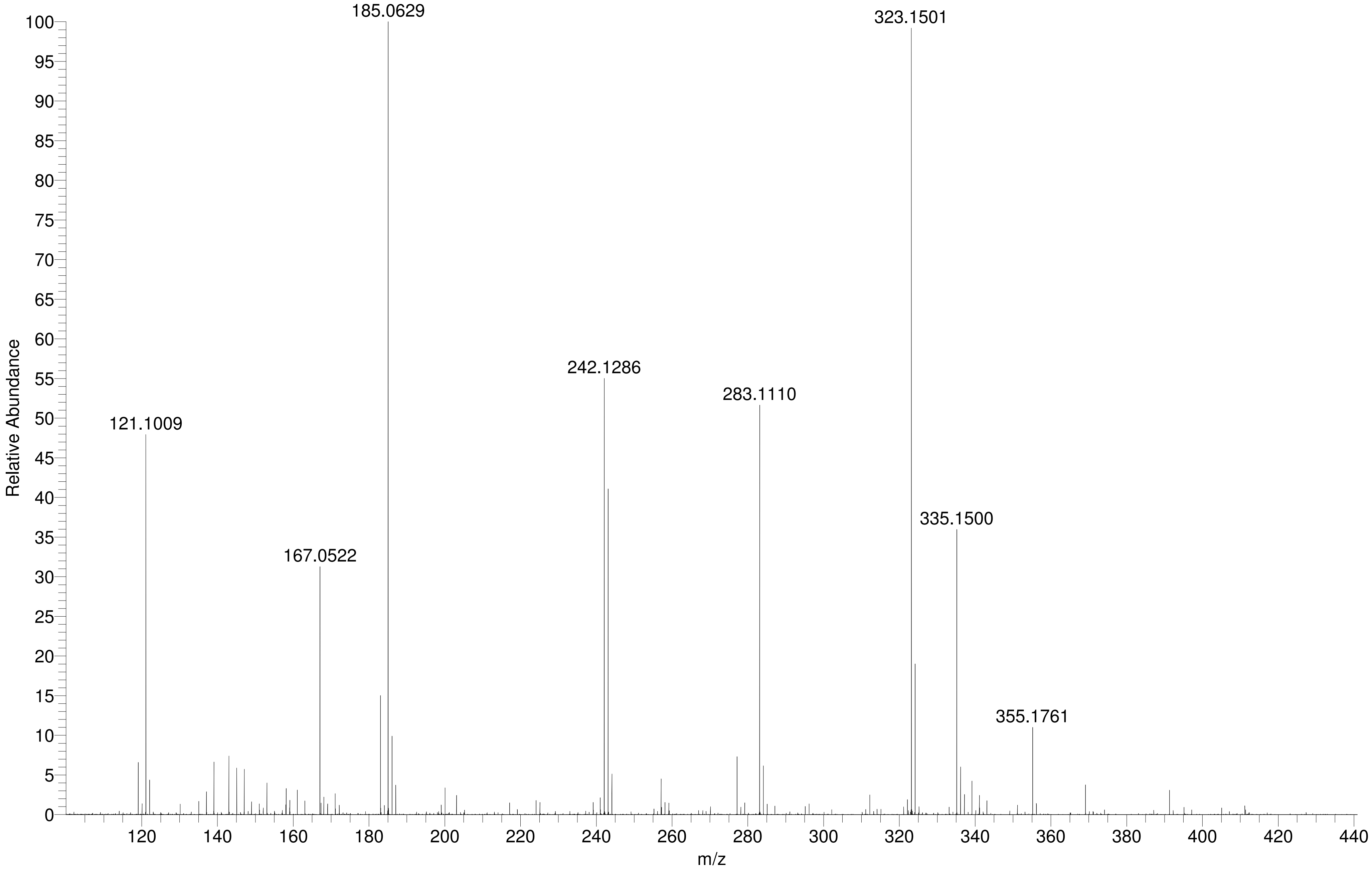

PA1-47 MW=538?
ASAP(DCM)

EPSRC National Centre Swansea
LTQ Orbitrap XL

Throup
06/11/2012 11:41:24

BRASHE229-PG-HASP #34-36 RT: 0.95-1.01 AV: 3 SM: 7G NL: 4.74E6
T: FTMS + p APCI corona Full ms [100.00-800.00]

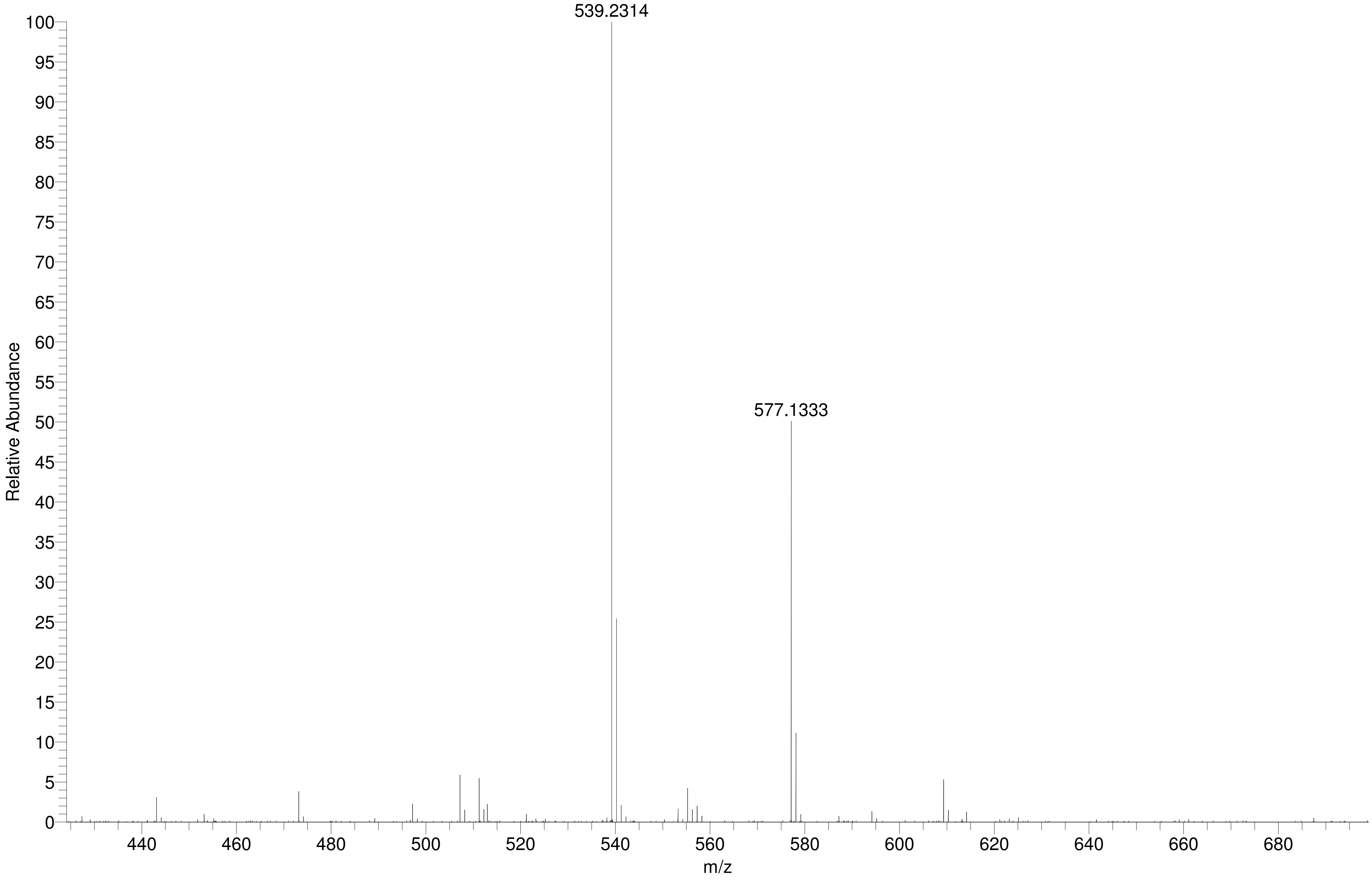

PA1-47 MW=538?
ASAP(DCM)

EPSRC National Centre Swansea
LTQ Orbitrap XL

Throup
06/11/2012 11:41:24

SM: 7G

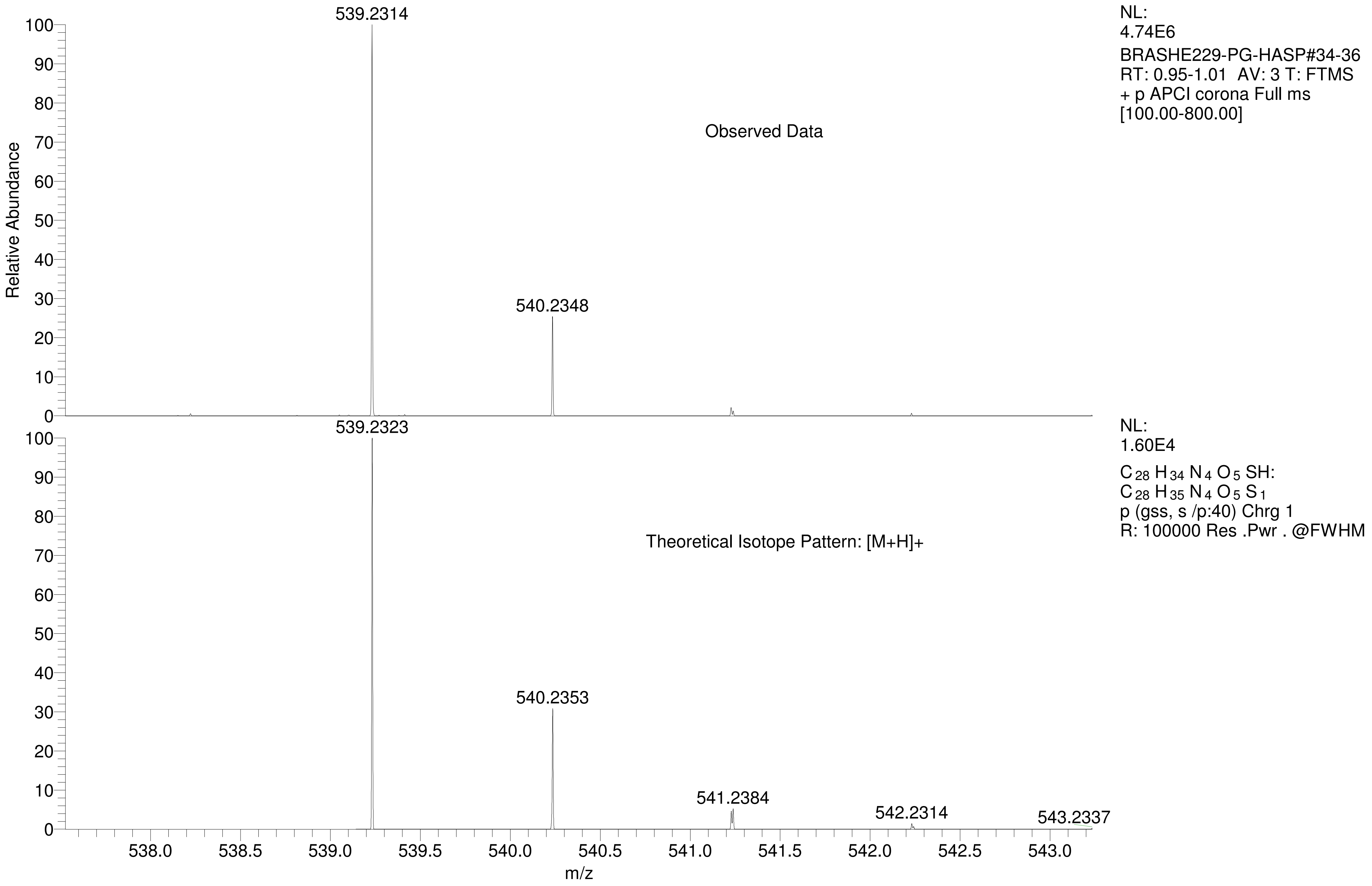

Isotope:	Min. .. Max.		
14 N	0....16		
16 O	0....15		
12 C	0....80		
1 H	0....100		
23 Na	0....0		
32 S	0....3		
Tolerance Window:	+- 5.00 ppm		
Db/Ring Equiv:	-3.. 100	N-Rule:	Do not use
Fits:	100	Charge:	1

Mass	Theoretical Mass	Delta [ppm]	RDB	Composition
539.2314	539.2314	-0.1	1.5	$C_{12} H_{35} O_{10} N_{12} S_{1}$
	539.2316	-0.3	23.0	$C_{34} H_{29} O_{2} N_{5}$
	539.2316	-0.4	10.0	$C_{19} H_{33} O_{2} N_{13} S_{2}$
	539.2316	-0.4	4.5	$C_{20} H_{39} O_{7} N_{6} S_{2}$
	539.2311	0.5	17.0	$C_{34} H_{37} O_{1} N_{1} S_{2}$
	539.2318	-0.7	7.5	$C_{28} H_{43} O_{4} S_{3}$
	539.2310	0.8	1.0	$C_{11} H_{37} O_{4} N_{15} S_{3}$
	539.2309	0.9	8.5	$C_{27} H_{39} O_{9} S_{1}$
	539.2309	0.9	14.0	$C_{26} H_{33} O_{4} N_{7} S_{1}$
	539.2307	1.2	5.5	$C_{19} H_{35} O_{12} N_{6}$
	539.2307	1.2	11.0	$C_{18} H_{29} O_{7} N_{13}$
	539.2321	-1.3	10.5	$C_{20} H_{31} O_{8} N_{10}$
	539.2321	-1.3	5.0	$C_{21} H_{37} O_{13} N_{3}$
	539.2323	-1.6	19.0	$C_{27} H_{29} N_{11} S_{1}$
	539.2323	-1.6	13.5	$C_{28} H_{35} O_{5} N_{4} S_{1}$
	539.2323	-1.7	0.5	$C_{13} H_{39} O_{5} N_{12} S_{3}$
	539.2305	1.8	8.0	$C_{26} H_{41} O_{3} N_{3} S_{3}$
	539.2303	2.1	-0.5	$C_{19} H_{43} O_{11} N_{2} S_{2}$
	539.2303	2.1	5.0	$C_{18} H_{37} O_{6} N_{9} S_{2}$
	539.2303	2.1	10.5	$C_{17} H_{31} O_{1} N_{16} S_{2}$
	539.2302	2.2	18.0	$C_{33} H_{33} O_{6} N_{1}$
	539.2302	2.2	23.5	$C_{32} H_{27} O_{1} N_{8}$
	539.2326	-2.2	-2.0	$C_{6} H_{33} O_{14} N_{15}$
	539.2301	2.4	2.0	$C_{10} H_{33} O_{9} N_{15} S_{1}$
	539.2328	-2.5	6.5	$C_{13} H_{31} O_{6} N_{16} S_{1}$
	539.2328	-2.5	1.0	$C_{14} H_{37} O_{11} N_{9} S_{1}$
	539.2329	-2.8	22.5	$C_{36} H_{31} O_{3} N_{2}$
	539.2330	-2.9	9.5	$C_{21} H_{35} O_{3} N_{10} S_{2}$
	539.2330	-2.9	4.0	$C_{22} H_{41} O_{8} N_{3} S_{2}$
	539.2298	3.0	17.5	$C_{32} H_{35} N_{4} S_{2}$
	539.2331	-3.2	12.5	$C_{29} H_{39} N_{4} S_{3}$

Mass	Theoretical Mass	Delta [ppm]	RDB	Composition
	539.2296	3.4	9.0	$C_{25} H_{37} O_{8} N_{3} S_{1}$
	539.2296	3.4	14.5	$C_{24} H_{31} O_{3} N_{10} S_{1}$
	539.2294	3.7	6.0	$C_{17} H_{33} O_{11} N_{9}$
	539.2294	3.7	11.5	$C_{16} H_{27} O_{6} N_{16}$
	539.2334	-3.7	15.5	$C_{21} H_{27} O_{4} N_{14}$
	539.2334	-3.8	10.0	$C_{22} H_{33} O_{9} N_{7}$
	539.2334	-3.8	4.5	$C_{23} H_{39} O_{14}$
	539.2335	-3.8	-3.0	$C_{7} H_{37} O_{9} N_{15} S_{2}$
	539.2336	-4.1	18.5	$C_{29} H_{31} O_{1} N_{8} S_{1}$
	539.2336	-4.1	13.0	$C_{30} H_{37} O_{6} N_{1} S_{1}$
	539.2336	-4.2	5.5	$C_{14} H_{35} O_{1} N_{16} S_{3}$
	539.2336	-4.2	0.0	$C_{15} H_{41} O_{6} N_{9} S_{3}$
	539.2291	4.2	8.5	$C_{24} H_{39} O_{2} N_{6} S_{3}$
	539.2289	4.6	0.0	$C_{17} H_{41} O_{10} N_{5} S_{2}$
	539.2289	4.6	5.5	$C_{16} H_{35} O_{5} N_{12} S_{2}$
	539.2289	4.6	18.5	$C_{31} H_{31} O_{5} N_{4}$
	539.2289	4.7	24.0	$C_{30} H_{25} N_{11}$
	539.2339	-4.7	-2.5	$C_{8} H_{35} O_{15} N_{12}$
	539.2288	4.9	-3.0	$C_{9} H_{37} O_{13} N_{11} S_{1}$

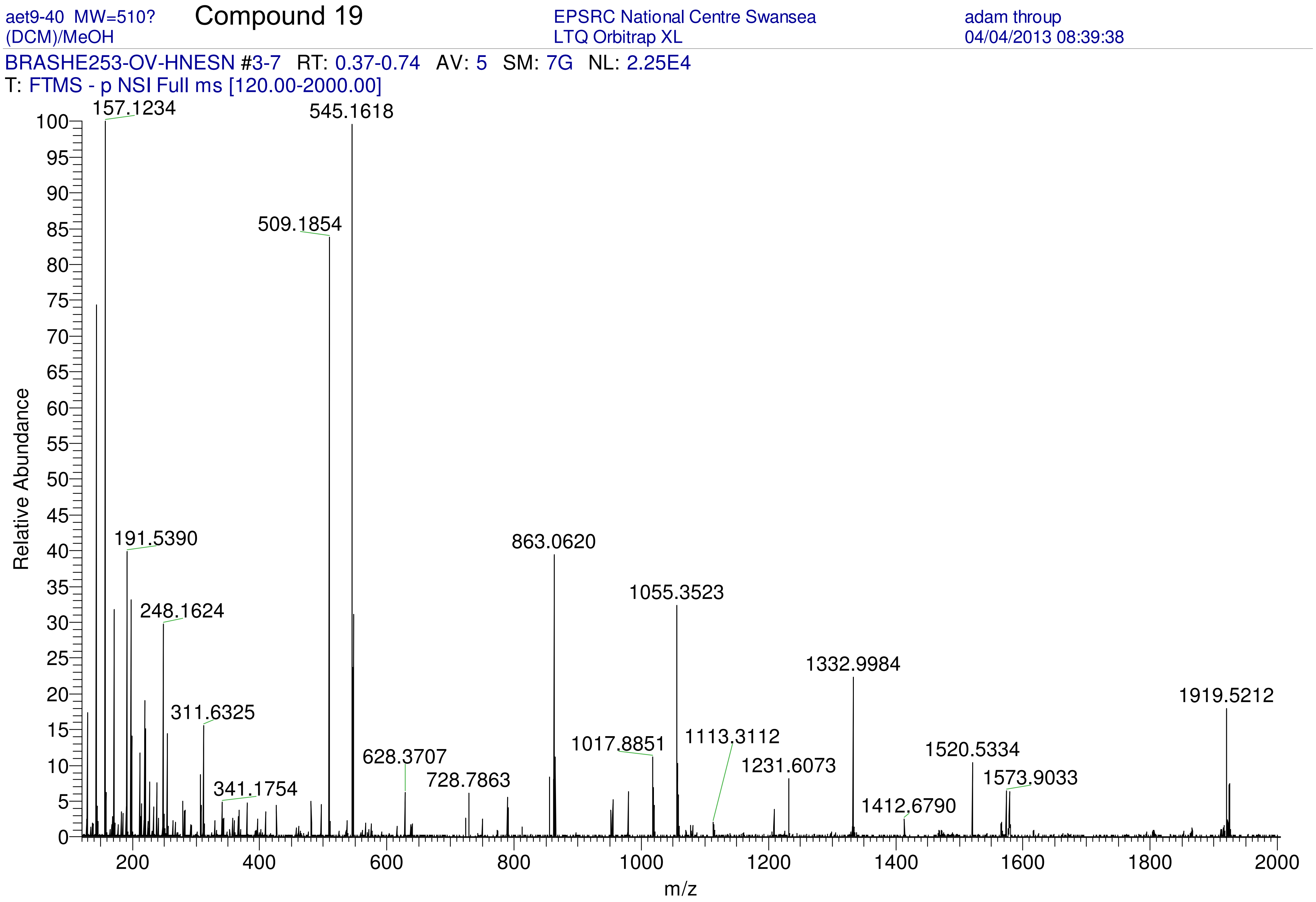
aet9-40 MW=510?
(DCM)/MeOH
Compound 19
EPSRC National Centre Swansea
LTQ Orbitrap XL
adam throup
04/04/2013 08:39:38
BRASHE253-OV-HNESN #3-7 RT: 0.37-0.74 AV: 5 SM: 7G NL: 2.25E4
T: FTMS - p NSI Full ms [120.00-2000.00]
157.1234
545.1618
509.1854
191.5390
248.1624
311.6325
341.1754
628.3707
728.7863
863.0620
1017.8851
1055.3523
1113.3112
1231.6073
1332.9984
1412.6790
1520.5334
1573.9033
1919.5212
Relative Abundance
m/z

aet9-40 MW=510?
(DCM)/MeOH

EPSRC National Centre Swansea
LTQ Orbitrap XL

adam throup
04/04/2013 08:39:38

BRASHE253-OV-HNESN #3-7   RT: 0.37-0.74   AV: 5   SM: 7G   NL: 1.89E4
T: FTMS - p NSI Full ms [120.00-2000.00]

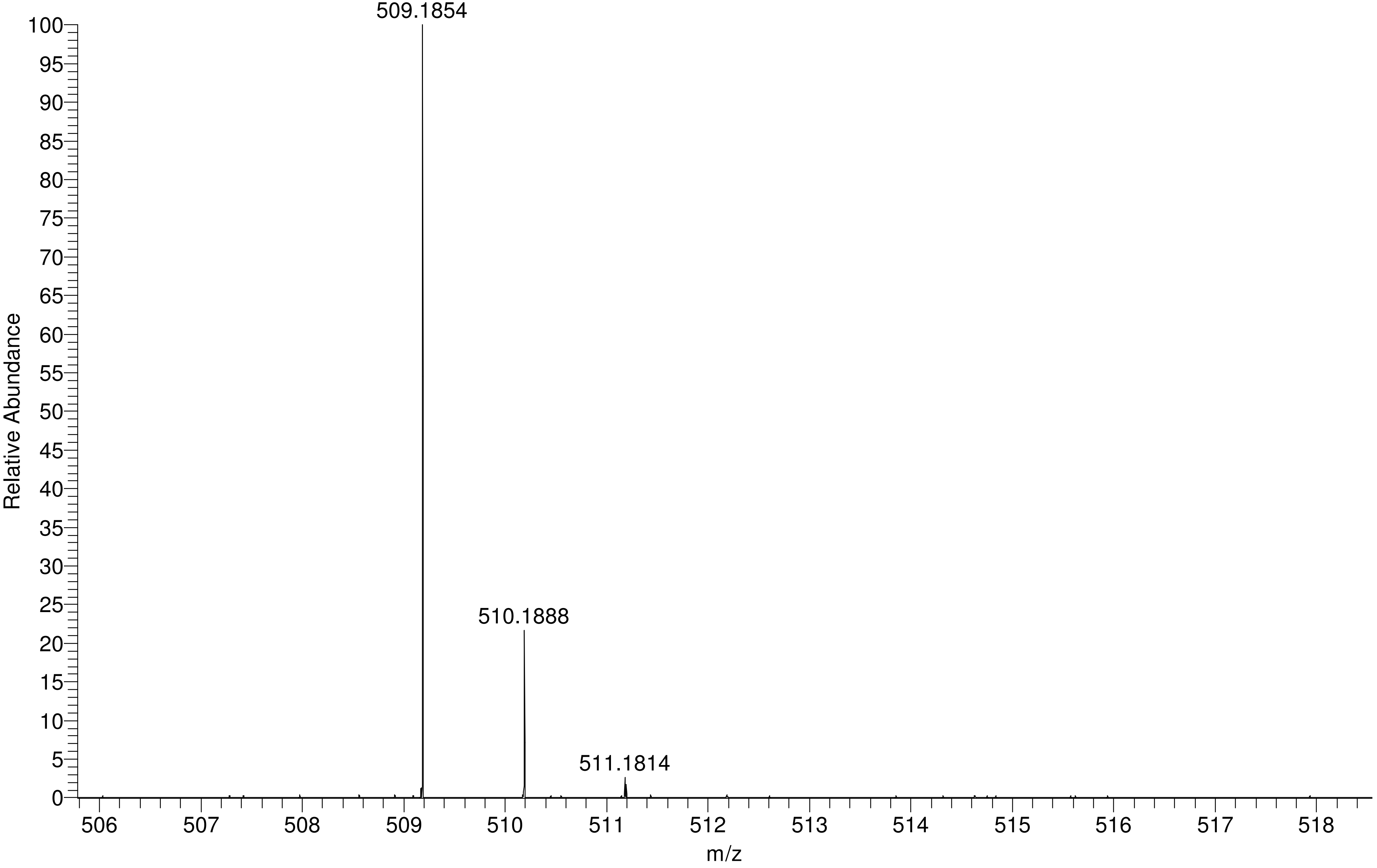

**Isotope:**	**Min. .. Max.**			
**12 C**	**0....60**			
**1 H**	**0....70**			
**14 N**	**0....15**			
**16 O**	**0....15**			
**23 Na**	**0....0**			
**32 S**	**0....3**			
**Tolerance Window:**	**+- 5.00 ppm**			
**Db/Ring Equiv:**	**-3.. 100**		**N-Rule:**	**Do not use**
**Fits:**	**100**		**Charge:**	**-1**

**Mass**	**Theoretical Mass**	**Delta [ppm]**	**RDB**	**Composition**
509.1854	509.1853	0.3	18.0	$C_{32} H_{31} O_{1} N_{1} S_{2}$
	509.1856	-0.4	2.5	$C_{10} H_{29} O_{10} N_{12} S_{1}$
	509.1856	-0.4	-3.0	$C_{11} H_{35} O_{15} N_{5} S_{1}$
	509.1851	0.6	2.0	$C_{9} H_{31} O_{4} N_{15} S_{3}$
	509.1857	-0.6	24.0	$C_{32} H_{23} O_{2} N_{5}$
	509.1851	0.6	9.5	$C_{25} H_{33} O_{9} S_{1}$
	509.1851	0.6	15.0	$C_{24} H_{27} O_{4} N_{7} S_{1}$
	509.1858	-0.7	11.0	$C_{17} H_{27} O_{2} N_{13} S_{2}$
	509.1858	-0.7	5.5	$C_{18} H_{33} O_{7} N_{6} S_{2}$
	509.1849	1.0	6.5	$C_{17} H_{29} O_{12} N_{6}$
	509.1849	1.0	12.0	$C_{16} H_{23} O_{7} N_{13}$
	509.1859	-1.1	8.5	$C_{26} H_{37} O_{4} S_{3}$
	509.1846	1.6	9.0	$C_{24} H_{35} O_{3} N_{3} S_{3}$
	509.1862	-1.6	11.5	$C_{18} H_{25} O_{8} N_{10}$
	509.1862	-1.6	6.0	$C_{19} H_{31} O_{13} N_{3}$
	509.1844	1.9	0.5	$C_{17} H_{37} O_{11} N_{2} S_{2}$
	509.1844	1.9	6.0	$C_{16} H_{31} O_{6} N_{9} S_{2}$
	509.1864	-2.0	20.0	$C_{25} H_{23} N_{11} S_{1}$
	509.1864	-2.0	14.5	$C_{26} H_{29} O_{5} N_{4} S_{1}$
	509.1844	2.0	19.0	$C_{31} H_{27} O_{6} N_{1}$
	509.1844	2.0	24.5	$C_{30} H_{21} O_{1} N_{8}$
	509.1864	-2.1	1.5	$C_{11} H_{33} O_{5} N_{12} S_{3}$
	509.1842	2.3	-2.5	$C_{9} H_{33} O_{14} N_{8} S_{1}$
	509.1842	2.3	3.0	$C_{8} H_{27} O_{9} N_{15} S_{1}$
	509.1867	-2.6	-1.0	$C_{4} H_{27} O_{14} N_{15}$
	509.1839	2.9	18.5	$C_{30} H_{29} N_{4} S_{2}$
	509.1869	-3.0	2.0	$C_{12} H_{31} O_{11} N_{9} S_{1}$
	509.1838	3.2	-3.0	$C_{8} H_{35} O_{8} N_{11} S_{3}$
	509.1871	-3.3	23.5	$C_{34} H_{25} O_{3} N_{2}$
	509.1837	3.3	10.0	$C_{23} H_{31} O_{8} N_{3} S_{1}$
	509.1837	3.3	15.5	$C_{22} H_{25} O_{3} N_{10} S_{1}$

Mass	Theoretical Mass	Delta [ppm]	RDB	Composition
	509.1871	-3.3	10.5	C_{19} H_{29} O_{3} N_{10} S_{2}
	509.1871	-3.3	5.0	C_{20} H_{35} O_{8} N_{3} S_{2}
	509.1836	3.6	7.0	C_{15} H_{27} O_{11} N_{9}
	509.1873	-3.7	13.5	C_{27} H_{33} N_{4} S_{3}
	509.1833	4.2	9.5	C_{22} H_{33} O_{2} N_{6} S_{3}
	509.1876	-4.3	16.5	C_{19} H_{21} O_{4} N_{14}
	509.1876	-4.3	11.0	C_{20} H_{27} O_{9} N_{7}
	509.1876	-4.3	5.5	C_{21} H_{33} O_{14}
	509.1876	-4.3	-2.0	C_{5} H_{31} O_{9} N_{15} S_{2}
	509.1831	4.6	1.0	C_{15} H_{35} O_{10} N_{5} S_{2}
	509.1831	4.6	6.5	C_{14} H_{29} O_{5} N_{12} S_{2}
	509.1878	-4.6	19.5	C_{27} H_{25} O_{1} N_{8} S_{1}
	509.1878	-4.6	14.0	C_{28} H_{31} O_{6} N_{1} S_{1}
	509.1830	4.6	19.5	C_{29} H_{25} O_{5} N_{4}
	509.1830	4.6	25.0	C_{28} H_{19} N_{11}
	509.1878	-4.7	1.0	C_{13} H_{35} O_{6} N_{9} S_{3}
	509.1829	4.9	-2.0	C_{7} H_{31} O_{13} N_{11} S_{1}

aet9-19 MW=552?
(DCM)/MeOH + NH4OAc

Compound 20

EPSRC National Centre Swansea
LTQ Orbitrap XL

adam throup
14/01/2013 16:40:17

BRASHE246-OJ-HNESP #37-50 RT: 0.95-1.29 AV: 13 SM: 7G NL: 2.62E7
T: FTMS + p NSI Full ms [120.00-2000.00]

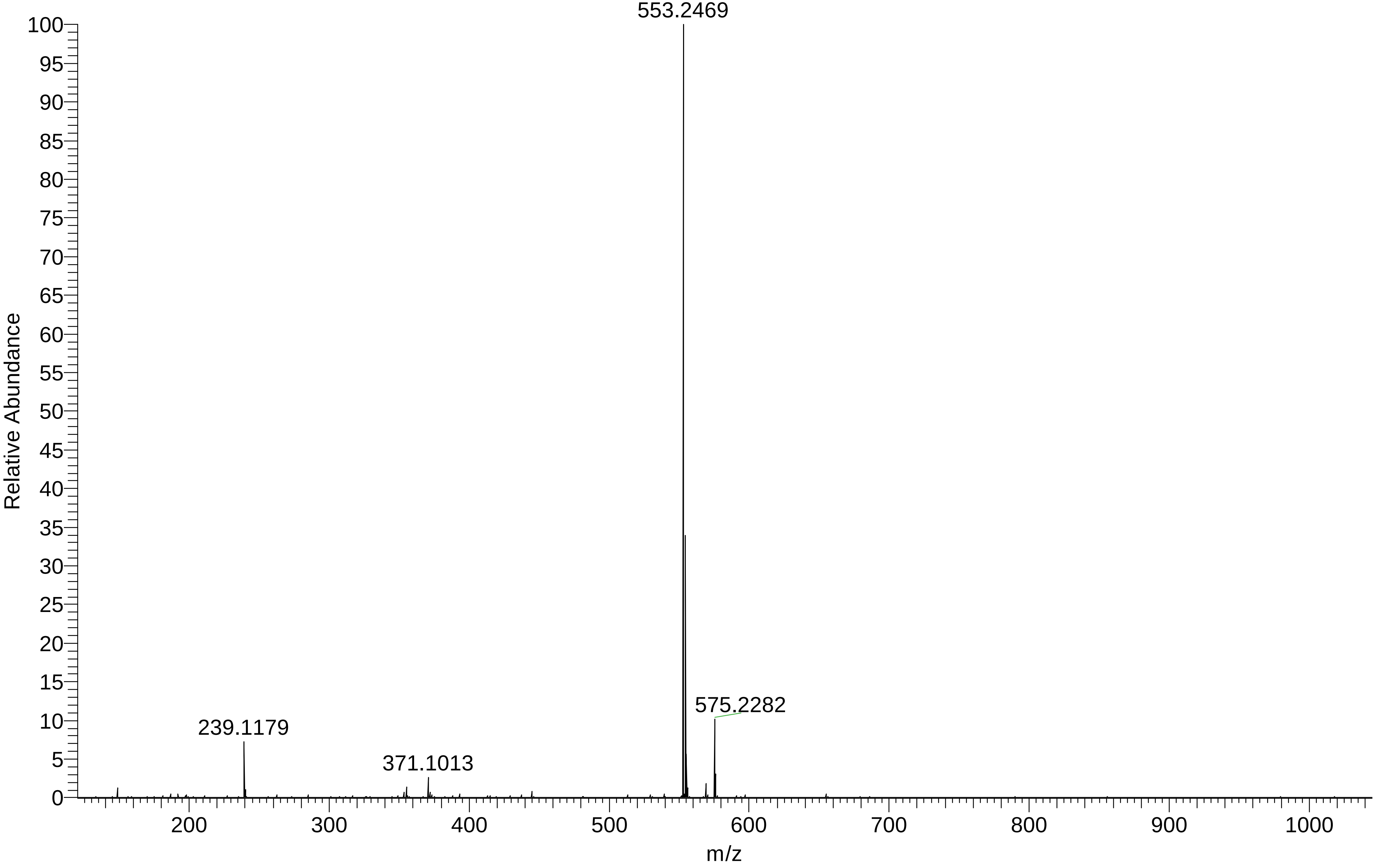

aet9-19 MW=552?
(DCM)/MeOH + NH4OAc

EPSRC National Centre Swansea
LTQ Orbitrap XL

adam throup
14/01/2013 16:40:17

BRASHE246-OJ-HNESP #37-50 RT: 0.95-1.29 AV: 13 SM: 7G NL: 2.62E7
T: FTMS + p NSI Full ms [120.00-2000.00]

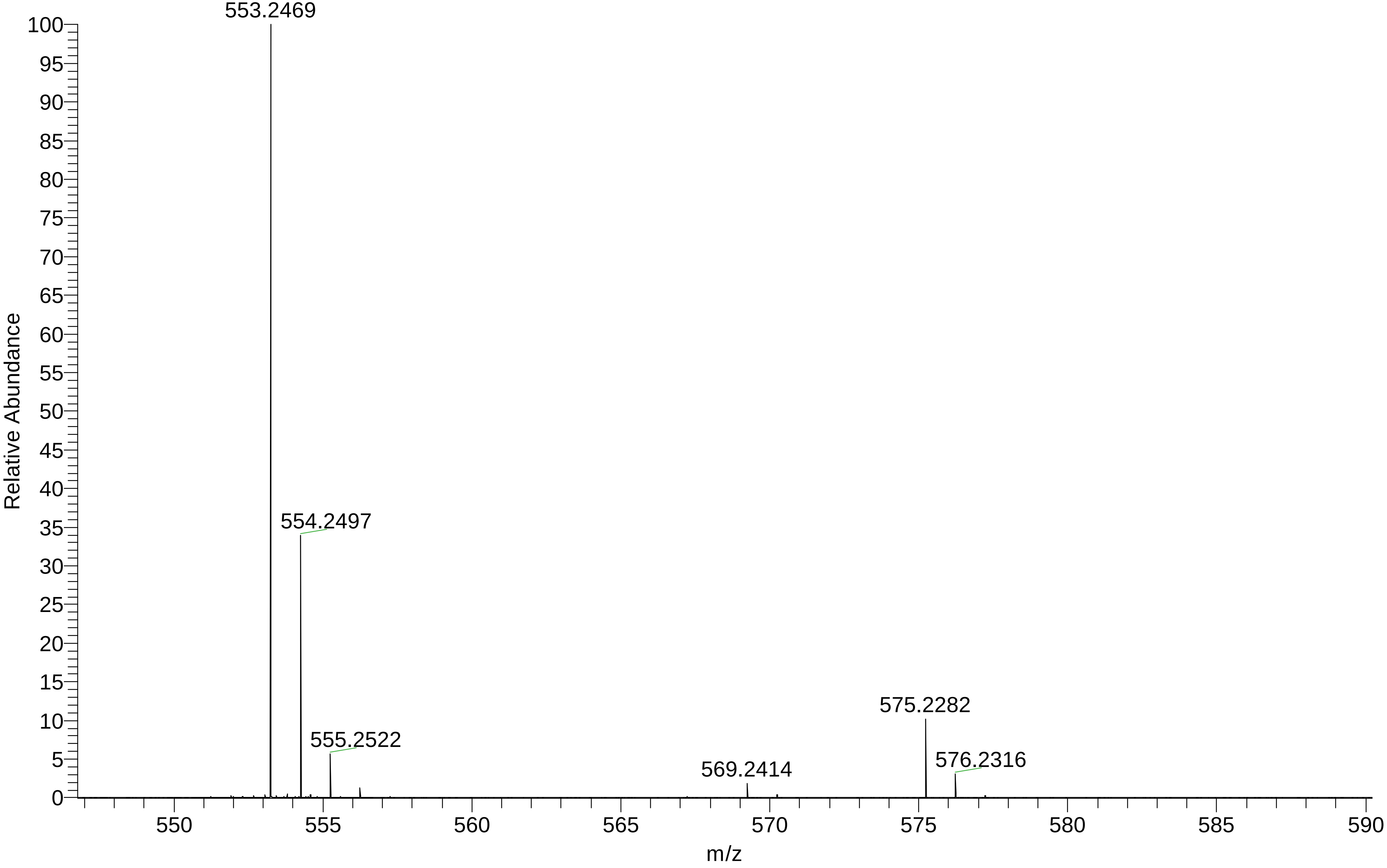

aet9-19 MW=552?
(DCM)/MeOH + NH4OAc

EPSRC National Centre Swansea
LTQ Orbitrap XL

adam throup
14/01/2013 16:40:17

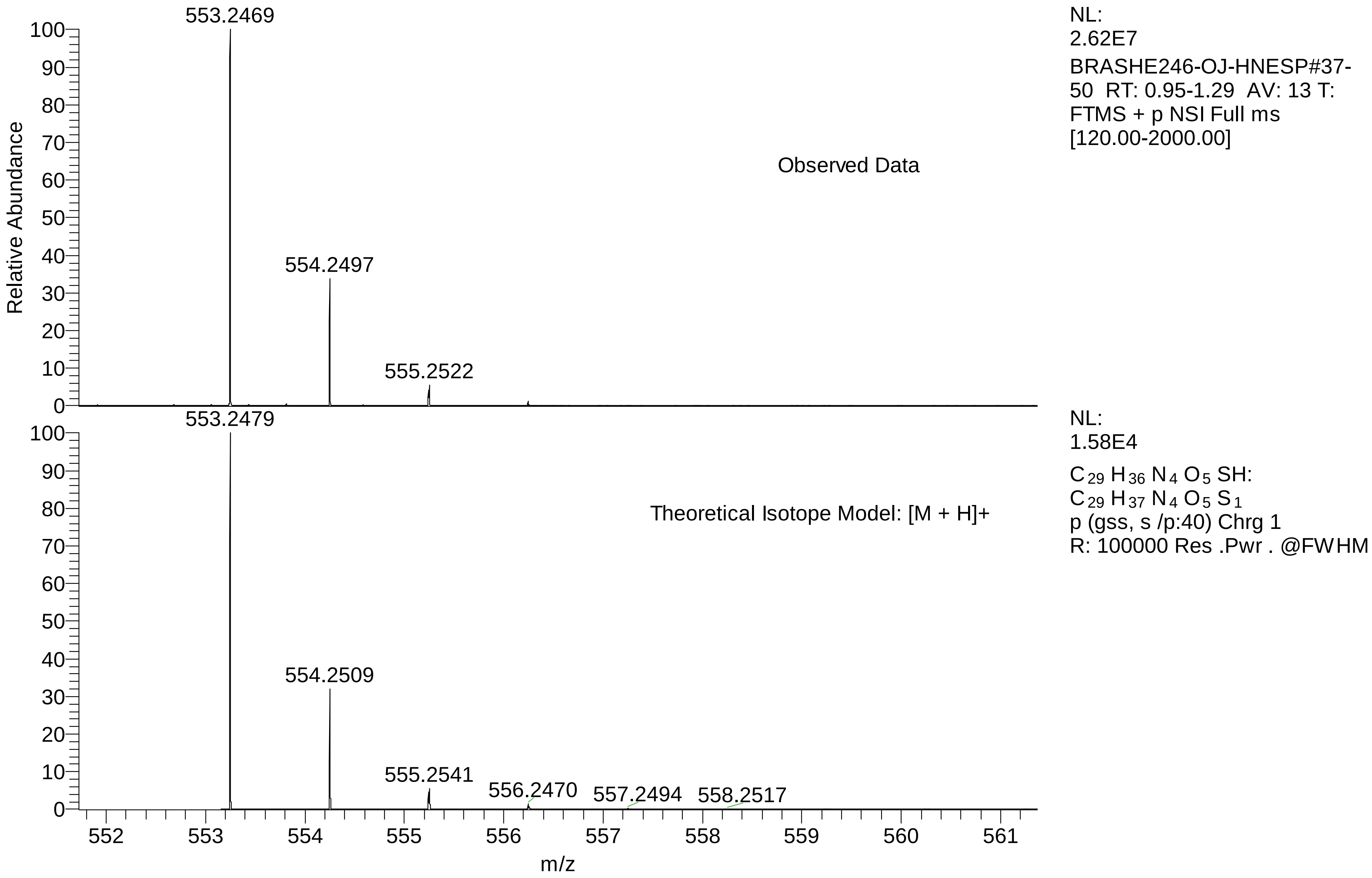

Isotope: **Min. .. Max.**
14 N **0....14**
16 O **0....15**
12 C **0....60**
1 H **0....80**
23 Na **0....0**
32 S **0....5**
Tolerance Window: **+- 5.00 ppm**
Db/Ring Equiv: -3.. 100 **N-Rule: Do not use**
Fits: 200 **Charge: 1**

**Mass**	**Theoretical Mass**	**Delta [ppm]**	**RDB**	**Composition**
553.2469	553.2468	0.2	-1.5	$C_{21} H_{49} O_6 N_2 S_4$
	553.2468	0.2	4.0	$C_{20} H_{43} O_1 N_9 S_4$
	553.2468	0.3	17.0	$C_{35} H_{39} O_1 N_1 S_2$
	553.2471	-0.3	1.5	$C_{13} H_{37} O_{10} N_{12} S_1$
	553.2466	0.6	8.5	$C_{28} H_{41} O_9 S_1$
	553.2466	0.6	14.0	$C_{27} H_{35} O_4 N_7 S_1$
	553.2472	-0.6	23.0	$C_{35} H_{31} O_2 N_5$
	553.2473	-0.7	10.0	$C_{20} H_{35} O_2 N_{13} S_2$
	553.2473	-0.7	4.5	$C_{21} H_{41} O_7 N_6 S_2$
	553.2464	0.9	5.5	$C_{20} H_{37} O_{12} N_6$
	553.2464	0.9	11.0	$C_{19} H_{31} O_7 N_{13}$
	553.2474	-1.0	7.5	$C_{29} H_{45} O_4 S_3$
	553.2461	1.4	8.0	$C_{27} H_{43} O_3 N_3 S_3$
	553.2477	-1.5	10.5	$C_{21} H_{33} O_8 N_{10}$
	553.2477	-1.5	5.0	$C_{22} H_{39} O_{13} N_3$
	553.2459	1.8	-0.5	$C_{20} H_{45} O_{11} N_2 S_2$
	553.2459	1.8	5.0	$C_{19} H_{39} O_6 N_9 S_2$
	553.2459	1.8	18.0	$C_{34} H_{35} O_6 N_1$
	553.2479	-1.8	19.0	$C_{28} H_{31} N_{11} S_1$
	553.2459	1.8	23.5	$C_{33} H_{29} O_1 N_8$
	553.2479	-1.8	13.5	$C_{29} H_{37} O_5 N_4 S_1$
	553.2480	-1.9	0.5	$C_{14} H_{41} O_5 N_{12} S_3$
	553.2481	-2.2	3.5	$C_{22} H_{45} O_2 N_6 S_4$
	553.2455	2.6	-1.0	$C_{19} H_{47} O_5 N_5 S_4$
	553.2454	2.6	4.5	$C_{18} H_{41} N_{12} S_4$
	553.2454	2.7	17.5	$C_{33} H_{37} N_4 S_2$
	553.2484	-2.8	1.0	$C_{15} H_{39} O_{11} N_9 S_1$
	553.2452	3.0	9.0	$C_{26} H_{39} O_8 N_3 S_1$
	553.2452	3.0	14.5	$C_{25} H_{33} O_3 N_{10} S_1$
	553.2486	-3.0	22.5	$C_{37} H_{33} O_3 N_2$
	553.2486	-3.1	9.5	$C_{22} H_{37} O_3 N_{10} S_2$

Mass	Theoretical Mass	Delta [ppm]	RDB	Composition
	553.2486	-3.1	4.0	$C_{23} H_{43} O_{8} N_{3} S_{2}$
	553.2451	3.3	6.0	$C_{18} H_{35} O_{11} N_{9}$
	553.2488	-3.4	12.5	$C_{30} H_{41} N_{4} S_{3}$
	553.2488	-3.5	-0.5	$C_{15} H_{45} N_{12} S_{5}$
	553.2448	3.9	8.5	$C_{25} H_{41} O_{2} N_{6} S_{3}$
	553.2491	-3.9	15.5	$C_{22} H_{29} O_{4} N_{14}$
	553.2491	-3.9	10.0	$C_{23} H_{35} O_{9} N_{7}$
	553.2491	-3.9	4.5	$C_{24} H_{41} O_{14}$
	553.2446	4.2	0.0	$C_{18} H_{43} O_{10} N_{5} S_{2}$
	553.2446	4.2	5.5	$C_{17} H_{37} O_{5} N_{12} S_{2}$
	553.2445	4.3	18.5	$C_{32} H_{33} O_{5} N_{4}$
	553.2493	-4.3	18.5	$C_{30} H_{33} O_{1} N_{8} S_{1}$
	553.2445	4.3	24.0	$C_{31} H_{27} N_{11}$
	553.2493	-4.3	13.0	$C_{31} H_{39} O_{6} N_{1} S_{1}$
	553.2493	-4.3	0.0	$C_{16} H_{43} O_{6} N_{9} S_{3}$
	553.2444	4.5	-3.0	$C_{10} H_{39} O_{13} N_{11} S_{1}$
	553.2495	-4.7	3.0	$C_{24} H_{47} O_{3} N_{3} S_{4}$
	553.2443	4.7	2.5	$C_{25} H_{49} O_{1} N_{2} S_{5}$
	553.2496	-4.9	-2.5	$C_{9} H_{37} O_{15} N_{12}$

Compound 21

AET9-3 MW=500?
ASAP(SOLID)

EPSRC National Centre Swansea
LTQ Orbitrap XL

Throup
06/11/2012 12:24:50

BRASHE235-PG-HASP #33-40 RT: 0.92-1.12 AV: 8 SM: 7G NL: 5.61E6
T: FTMS + p APCI corona Full ms [100.00-800.00]

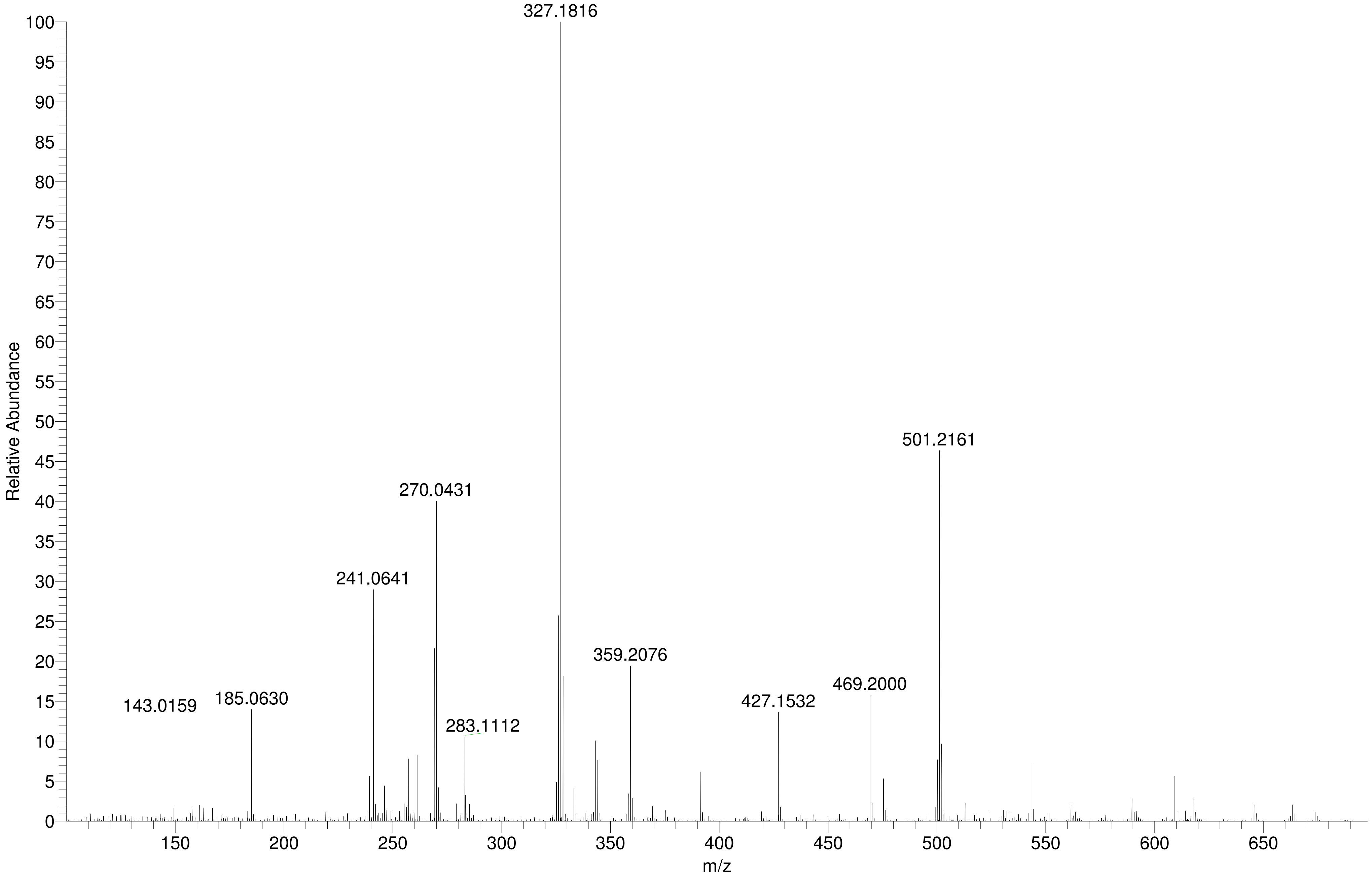

AET9-3 MW=500?
ASAP(SOLID)

EPSRC National Centre Swansea
LTQ Orbitrap XL

Throup
06/11/2012 12:24:50

BRASHE235-PG-HASP #33-40 RT: 0.92-1.12 AV: 8 SM: 7G NL: 5.61E6
T: FTMS + p APCI corona Full ms [100.00-800.00]

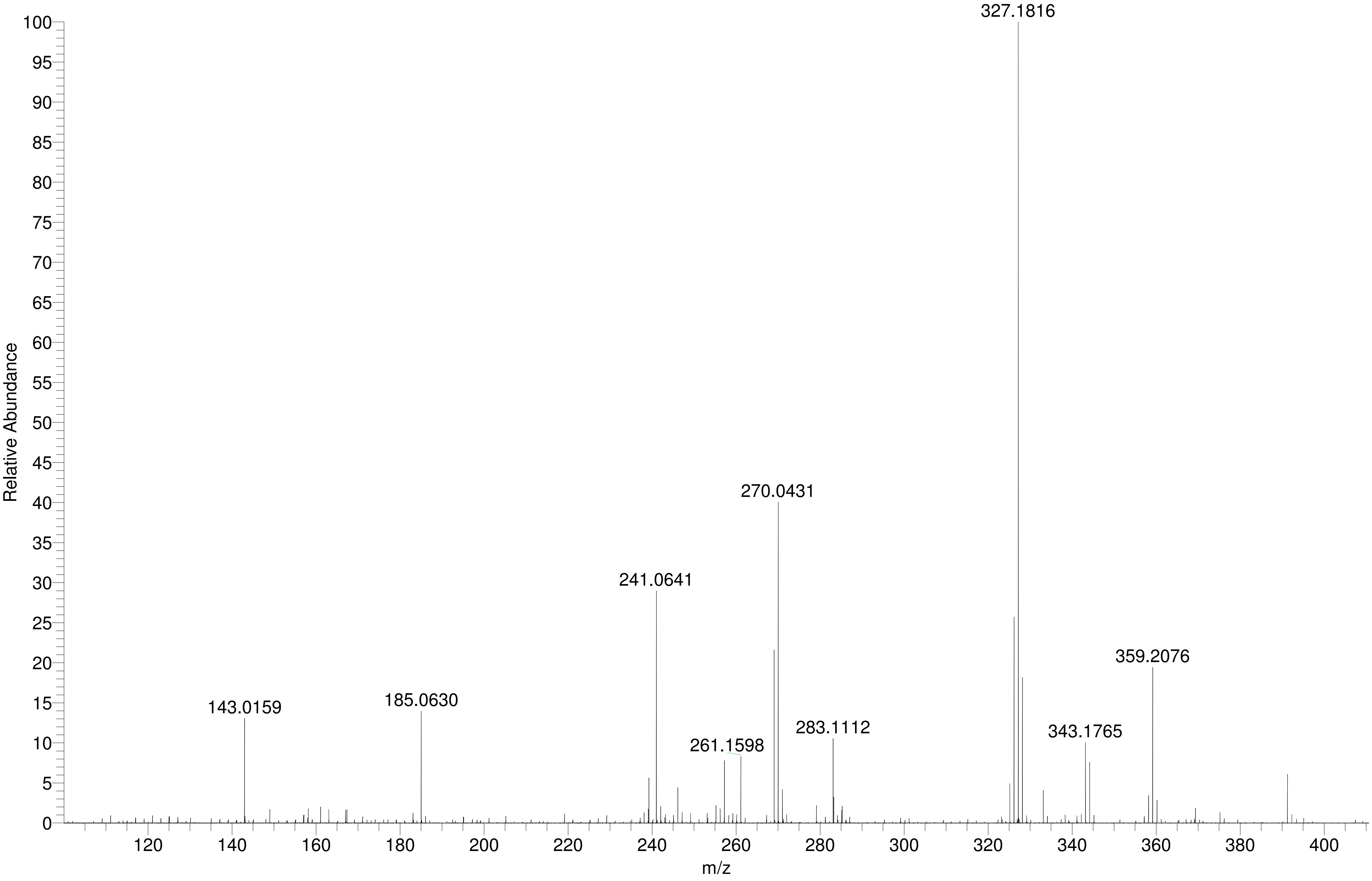

AET9-3 MW=500?
ASAP(SOLID)

EPSRC National Centre Swansea
LTQ Orbitrap XL

Throup
06/11/2012 12:24:50

BRASHE235-PG-HASP #33-40 RT: 0.92-1.12 AV: 8 SM: 7G NL: 2.60E6
T: FTMS + p APCI corona Full ms [100.00-800.00]

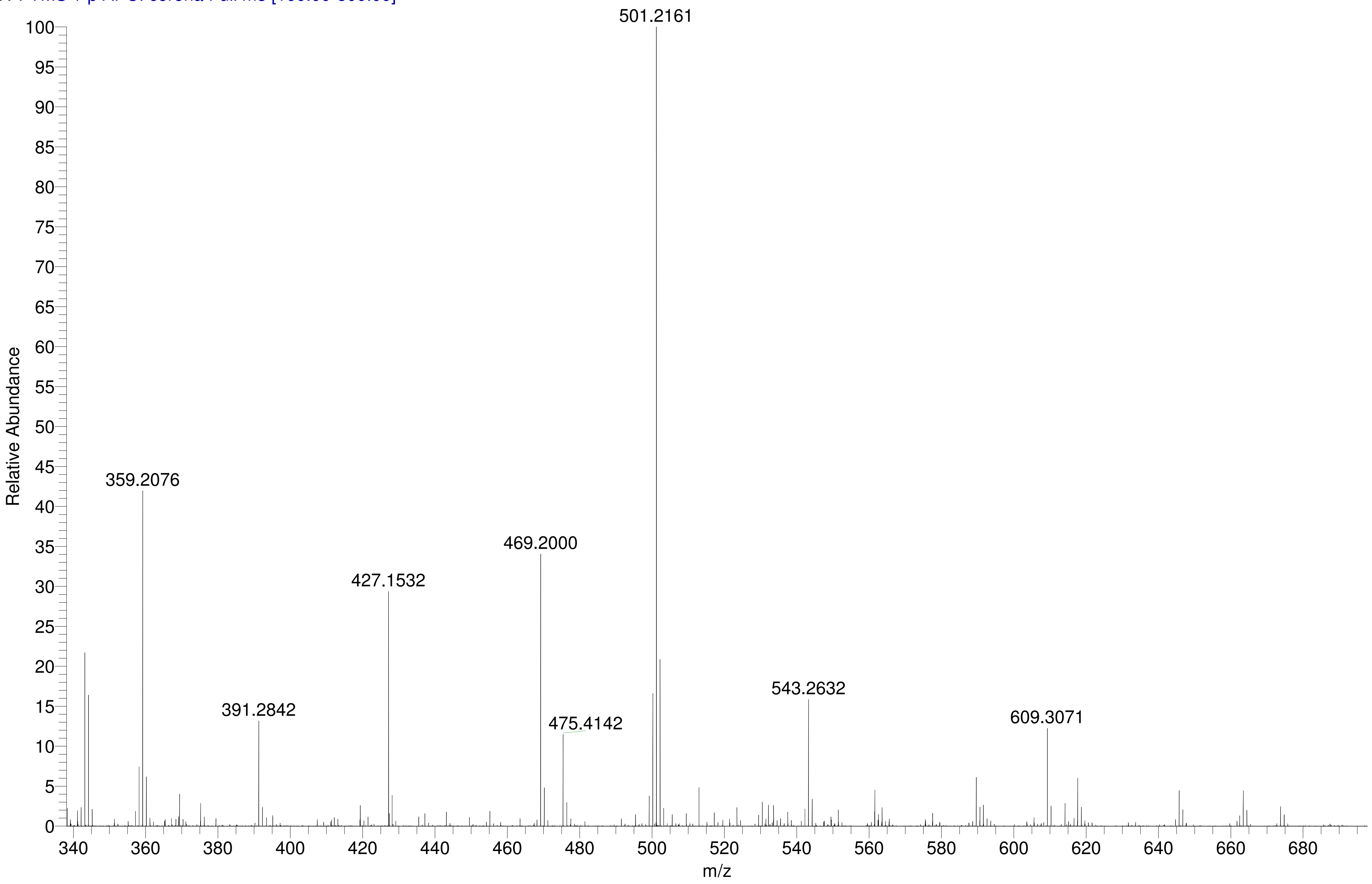

AET9-3 MW=500?
ASAP(SOLID)

EPSRC National Centre Swansea
LTQ Orbitrap XL

Throup
06/11/2012 12:24:50

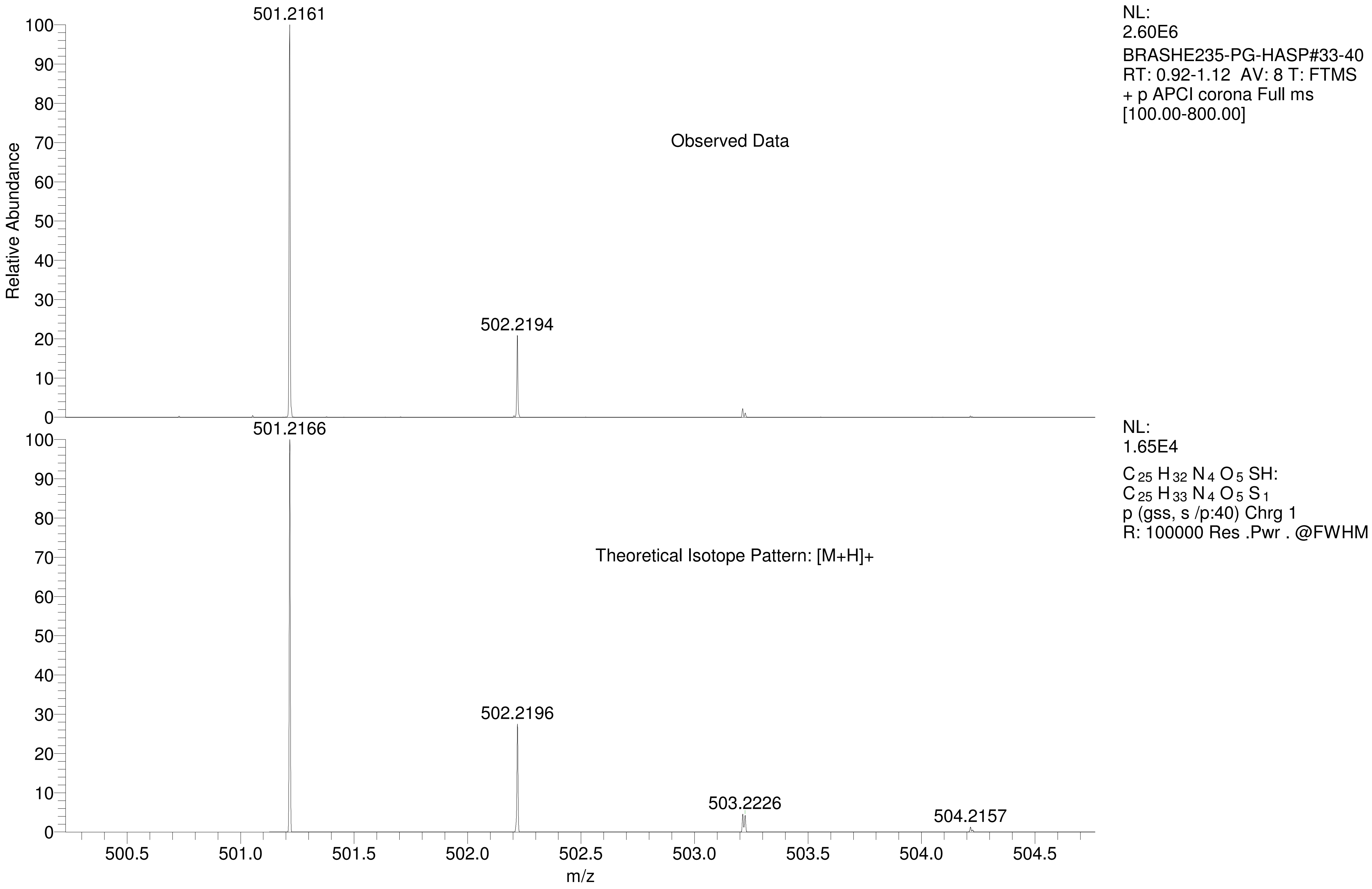

Isotope: **Min. .. Max.**
14 N **0....16**
16 O **0....15**
12 C **0....80**
1 H **0....100**
23 Na **0....0**
32 S **0....3**
Tolerance Window: **+- 5.00 ppm**
Db/Ring Equiv: **-3.. 100** **N-Rule:** **Do not use**
Fits: **100** **Charge:** **1**

**Mass**	**Theoretical Mass**	**Delta [ppm]**	**RDB**	**Composition**
501.2161	501.2161	-0.1	5.5	$C_{25} H_{41} O_4 S_3$
	501.2160	0.3	2.5	$C_{17} H_{37} O_7 N_6 S_2$
	501.2160	0.3	8.0	$C_{16} H_{31} O_2 N_{13} S_2$
	501.2159	0.3	21.0	$C_{31} H_{27} O_2 N_5$
	501.2158	0.6	-0.5	$C_9 H_{33} O_{10} N_{12} S_1$
	501.2164	-0.7	8.5	$C_{17} H_{29} O_8 N_{10}$
	501.2164	-0.7	3.0	$C_{18} H_{35} O_{13} N_3$
	501.2166	-1.0	17.0	$C_{24} H_{27} N_{11} S_1$
	501.2166	-1.0	11.5	$C_{25} H_{33} O_5 N_4 S_1$
	501.2167	-1.1	-1.5	$C_{10} H_{37} O_5 N_{12} S_3$
	501.2155	1.3	15.0	$C_{31} H_{35} O_1 N_1 S_2$
	501.2153	1.6	-1.0	$C_8 H_{35} O_4 N_{15} S_3$
	501.2153	1.6	6.5	$C_{24} H_{37} O_9 S_1$
	501.2153	1.6	12.0	$C_{23} H_{31} O_4 N_7 S_1$
	501.2151	2.0	3.5	$C_{16} H_{33} O_{12} N_6$
	501.2151	2.0	9.0	$C_{15} H_{27} O_7 N_{13}$
	501.2171	-2.0	4.5	$C_{10} H_{29} O_6 N_{16} S_1$
	501.2171	-2.0	-1.0	$C_{11} H_{35} O_{11} N_9 S_1$
	501.2173	-2.3	20.5	$C_{33} H_{29} O_3 N_2$
	501.2173	-2.4	7.5	$C_{18} H_{33} O_3 N_{10} S_2$
	501.2173	-2.4	2.0	$C_{19} H_{39} O_8 N_3 S_2$
	501.2148	2.6	6.0	$C_{23} H_{39} O_3 N_3 S_3$
	501.2175	-2.8	10.5	$C_{26} H_{37} N_4 S_3$
	501.2146	2.9	-2.5	$C_{16} H_{41} O_{11} N_2 S_2$
	501.2146	2.9	3.0	$C_{15} H_{35} O_6 N_9 S_2$
	501.2146	3.0	8.5	$C_{14} H_{29} O_1 N_{16} S_2$
	501.2146	3.0	16.0	$C_{30} H_{31} O_6 N_1$
	501.2146	3.0	21.5	$C_{29} H_{25} O_1 N_8$
	501.2144	3.3	0.0	$C_7 H_{31} O_9 N_{15} S_1$
	501.2178	-3.3	13.5	$C_{18} H_{25} O_4 N_{14}$
	501.2178	-3.3	8.0	$C_{19} H_{31} O_9 N_7$

Mass	Theoretical Mass	Delta [ppm]	RDB	Composition
	501.2178	-3.4	2.5	C_{20} H_{37} O_{14}
	501.2180	-3.7	16.5	C_{26} H_{29} O_{1} N_{8} S_{1}
	501.2180	-3.7	11.0	C_{27} H_{35} O_{6} N_{1} S_{1}
	501.2180	-3.8	3.5	C_{11} H_{33} O_{1} N_{16} S_{3}
	501.2180	-3.8	-2.0	C_{12} H_{39} O_{6} N_{9} S_{3}
	501.2141	4.0	15.5	C_{29} H_{33} N_{4} S_{2}
	501.2139	4.3	7.0	C_{22} H_{35} O_{8} N_{3} S_{1}
	501.2139	4.3	12.5	C_{21} H_{29} O_{3} N_{10} S_{1}
	501.2138	4.7	4.0	C_{14} H_{31} O_{11} N_{9}
	501.2137	4.7	9.5	C_{13} H_{25} O_{6} N_{16}
	501.2185	-4.7	4.0	C_{12} H_{31} O_{7} N_{13} S_{1}
	501.2185	-4.7	-1.5	C_{13} H_{37} O_{12} N_{6} S_{1}

AET9-4 MW=542?
ASAP(SOLID)

Compound 22

EPSRC National Centre Swansea
LTQ Orbitrap XL

Throup
06/11/2012 12:17:45

BRASHE234-PG-HASP #45-50 RT: 1.26-1.41 AV: 6 SM: 7G NL: 1.58E7
T: FTMS + p APCI corona Full ms [100.00-800.00]

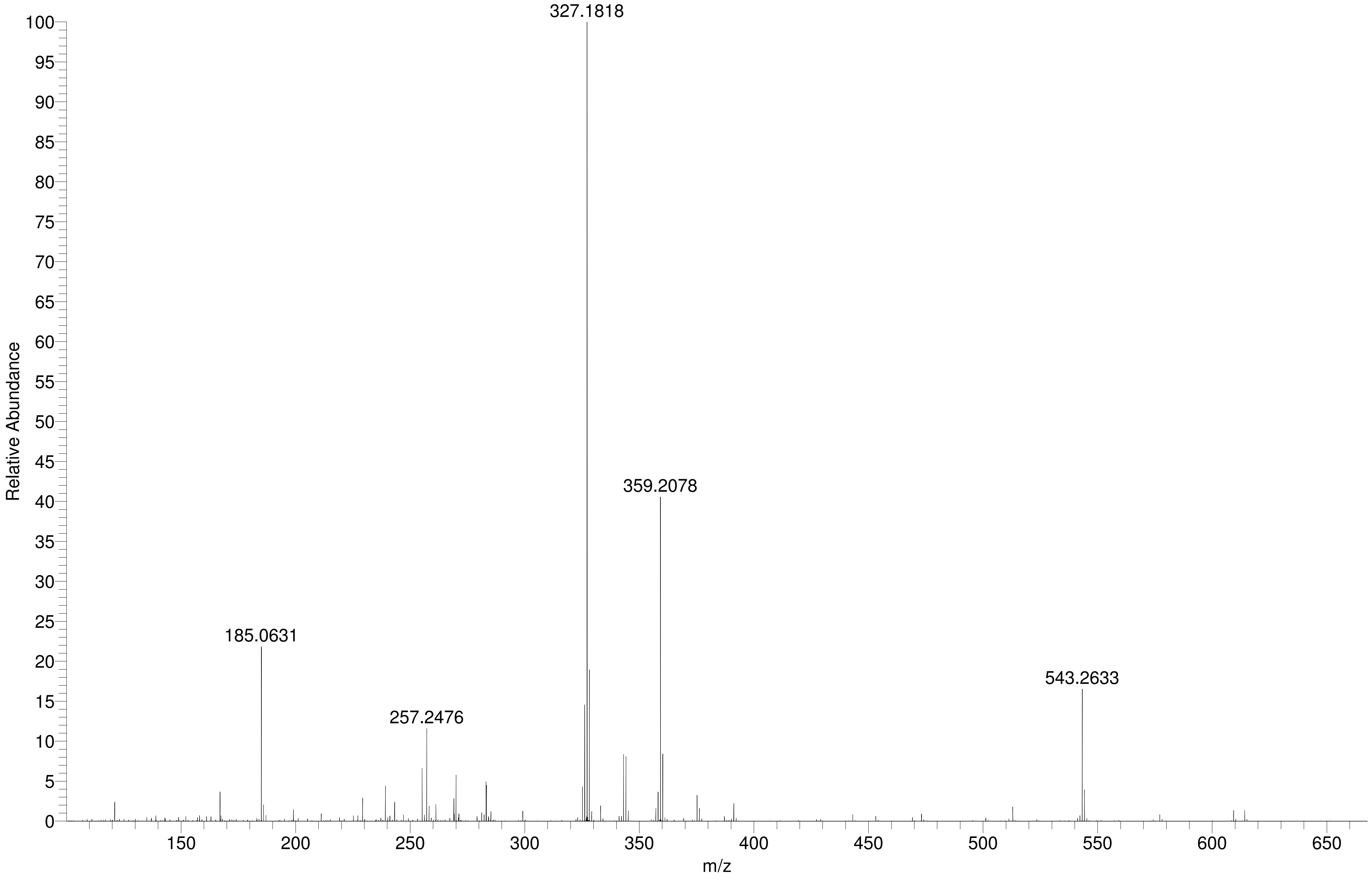

AET9-4 MW=542?
ASAP(SOLID)

EPSRC National Centre Swansea
LTQ Orbitrap XL

Throup
06/11/2012 12:17:45

BRASHE234-PG-HASP #45-50 RT: 1.26-1.41 AV: 6 SM: 7G NL: 1.58E7
T: FTMS + p APCI corona Full ms [100.00-800.00]

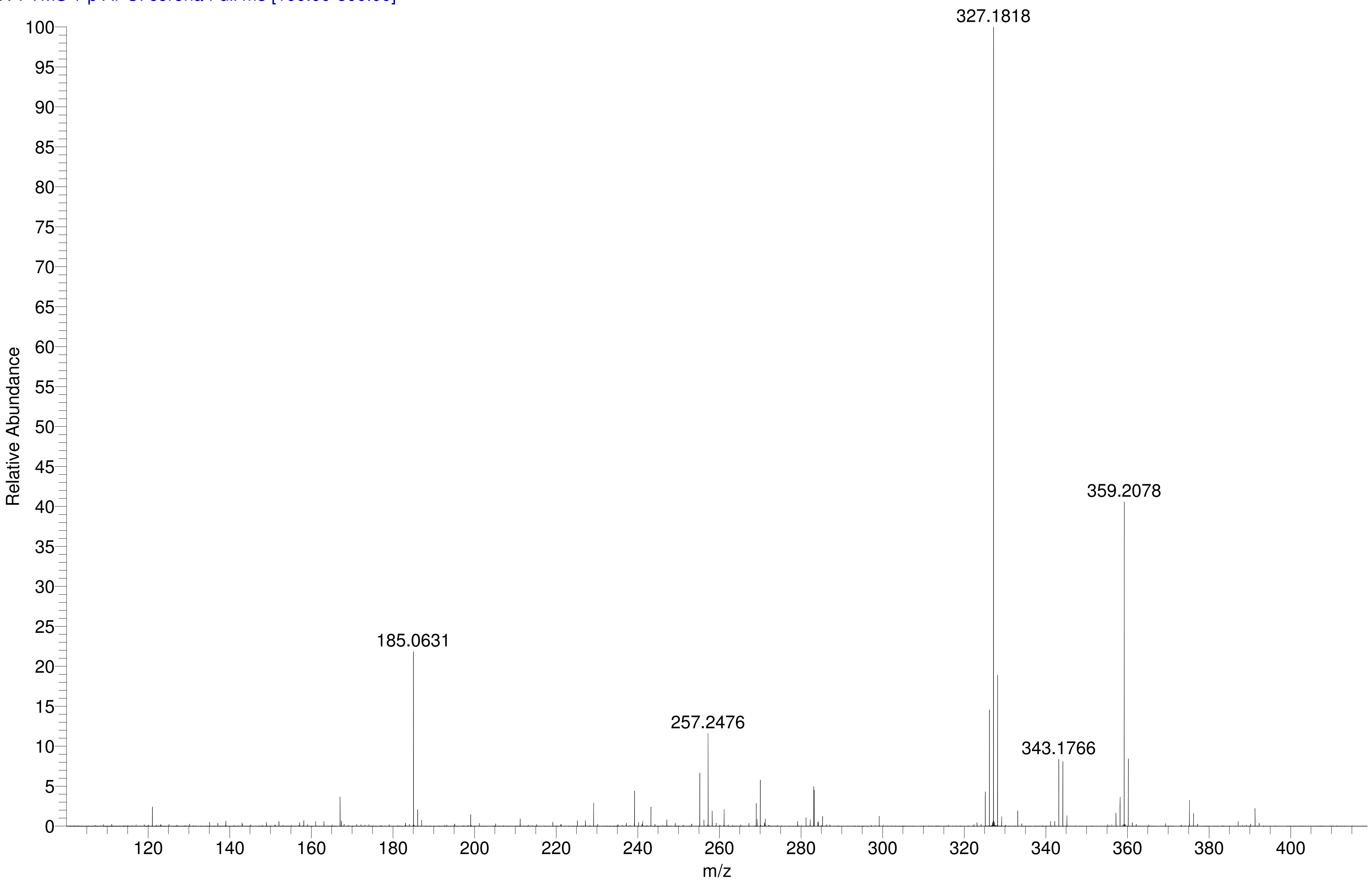

AET9-4 MW=542?
ASAP(SOLID)

EPSRC National Centre Swansea
LTQ Orbitrap XL

Throup
06/11/2012 12:17:45

BRASHE234-PG-HASP #45-50 RT: 1.26-1.41 AV: 6 SM: 7G NL: 2.61E6
T: FTMS + p APCI corona Full ms [100.00-800.00]

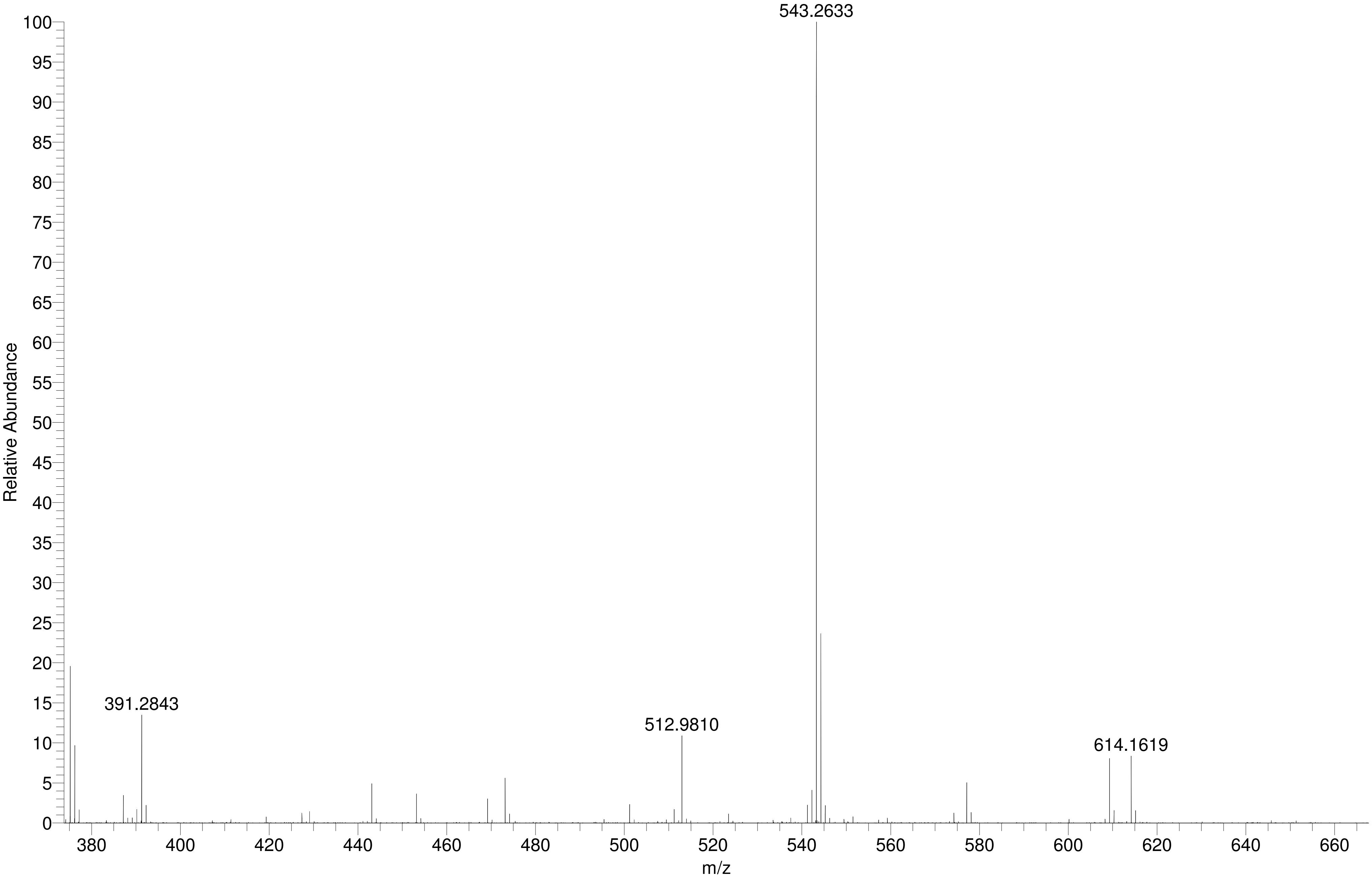

AET9-4 MW=542?
ASAP(SOLID)

EPSRC National Centre Swansea
LTQ Orbitrap XL

Throup
06/11/2012 12:17:45

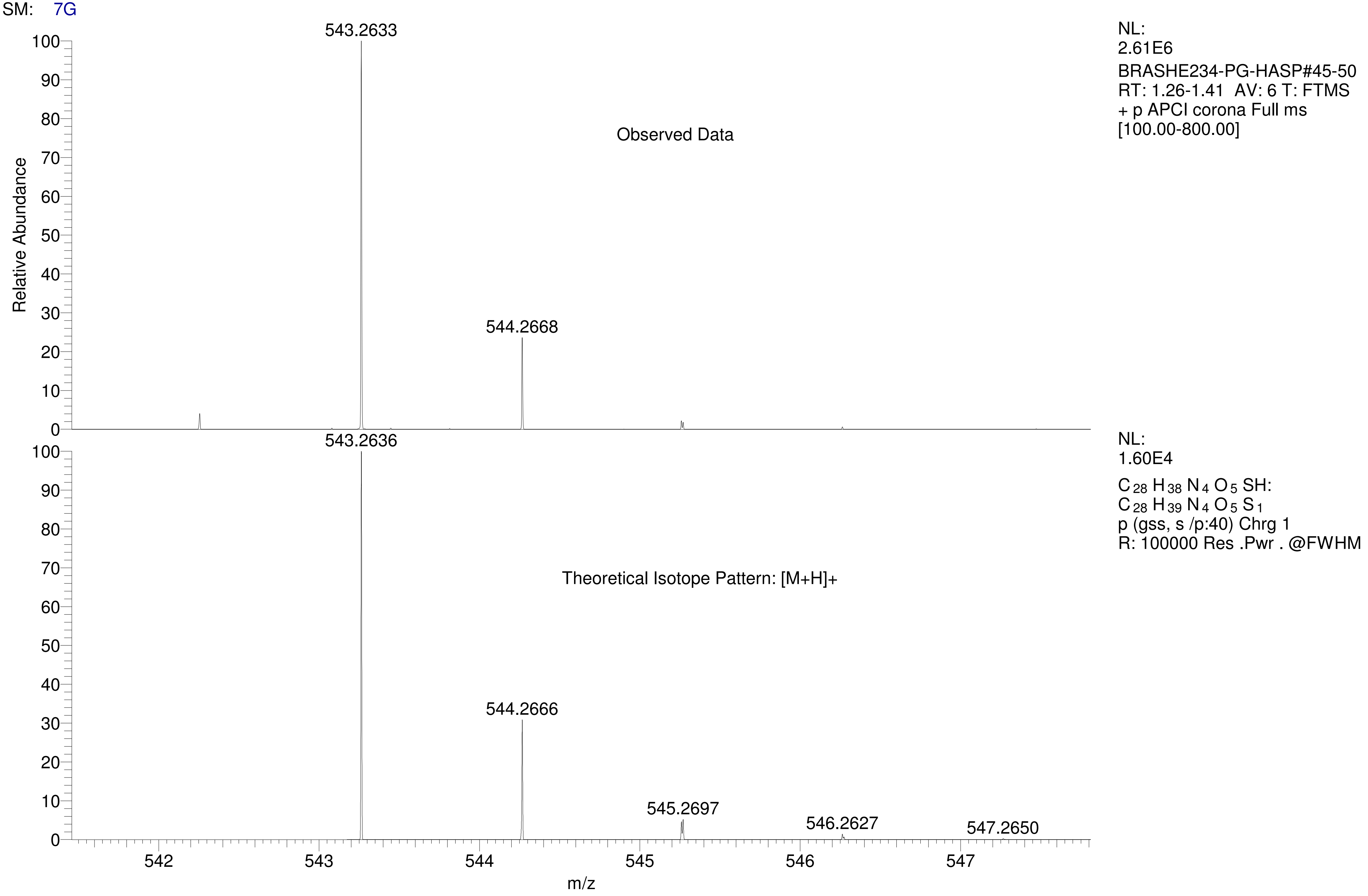

**Isotope:**	**Min. .. Max.**			
**14 N**	**0....16**			
**16 O**	**0....15**			
**12 C**	**0....80**			
**1 H**	**0....100**			
**23 Na**	**0....0**			
**32 S**	**0....3**			
**Tolerance Window:**	**+- 5.00 ppm**			
**Db/Ring Equiv:**	**-3.. 100**		**N-Rule:**	**Do not use**
**Fits:**	**100**		**Charge:**	**1**

**Mass**	**Theoretical Mass**	**Delta [ppm]**	**RDB**	**Composition**
543.2633	543.2634	-0.2	8.5	$C_{20} H_{35} O_{8} N_{10}$
	543.2634	-0.2	3.0	$C_{21} H_{41} O_{13} N_{3}$
	543.2631	0.4	5.5	$C_{28} H_{47} O_{4} S_{3}$
	543.2636	-0.5	17.0	$C_{27} H_{33} N_{11} S_{1}$
	543.2636	-0.5	11.5	$C_{28} H_{39} O_{5} N_{4} S_{1}$
	543.2636	-0.6	-1.5	$C_{13} H_{43} O_{5} N_{12} S_{3}$
	543.2629	0.7	2.5	$C_{20} H_{43} O_{7} N_{6} S_{2}$
	543.2629	0.7	8.0	$C_{19} H_{37} O_{2} N_{13} S_{2}$
	543.2629	0.8	21.0	$C_{34} H_{33} O_{2} N_{5}$
	543.2627	1.0	-0.5	$C_{12} H_{39} O_{10} N_{12} S_{1}$
	543.2641	-1.4	4.5	$C_{13} H_{35} O_{6} N_{16} S_{1}$
	543.2641	-1.4	-1.0	$C_{14} H_{41} O_{11} N_{9} S_{1}$
	543.2624	1.6	15.0	$C_{34} H_{41} O_{1} N_{1} S_{2}$
	543.2642	-1.7	20.5	$C_{36} H_{35} O_{3} N_{2}$
	543.2643	-1.8	7.5	$C_{21} H_{39} O_{3} N_{10} S_{2}$
	543.2643	-1.8	2.0	$C_{22} H_{45} O_{8} N_{3} S_{2}$
	543.2623	1.9	-1.0	$C_{11} H_{41} O_{4} N_{15} S_{3}$
	543.2622	2.0	6.5	$C_{27} H_{43} O_{9} S_{1}$
	543.2622	2.0	12.0	$C_{26} H_{37} O_{4} N_{7} S_{1}$
	543.2644	-2.1	10.5	$C_{29} H_{43} N_{4} S_{3}$
	543.2620	2.3	3.5	$C_{19} H_{39} O_{12} N_{6}$
	543.2620	2.3	9.0	$C_{18} H_{33} O_{7} N_{13}$
	543.2647	-2.6	13.5	$C_{21} H_{31} O_{4} N_{14}$
	543.2647	-2.6	8.0	$C_{22} H_{37} O_{9} N_{7}$
	543.2647	-2.6	2.5	$C_{23} H_{43} O_{14}$
	543.2618	2.8	6.0	$C_{26} H_{45} O_{3} N_{3} S_{3}$
	543.2649	-3.0	16.5	$C_{29} H_{35} O_{1} N_{8} S_{1}$
	543.2649	-3.0	11.0	$C_{30} H_{41} O_{6} N_{1} S_{1}$
	543.2649	-3.0	3.5	$C_{14} H_{39} O_{1} N_{16} S_{3}$
	543.2649	-3.0	-2.0	$C_{15} H_{45} O_{6} N_{9} S_{3}$
	543.2616	3.2	-2.5	$C_{19} H_{47} O_{11} N_{2} S_{2}$

Mass	Theoretical Mass	Delta [ppm]	RDB	Composition
	543.2616	3.2	3.0	$C_{18} H_{41} O_{6} N_{9} S_{2}$
	543.2616	3.2	8.5	$C_{17} H_{35} O_{1} N_{16} S_{2}$
	543.2615	3.2	16.0	$C_{33} H_{37} O_{6} N_{1}$
	543.2615	3.3	21.5	$C_{32} H_{31} O_{1} N_{8}$
	543.2614	3.5	0.0	$C_{10} H_{37} O_{9} N_{15} S_{1}$
	543.2654	-3.9	4.0	$C_{15} H_{37} O_{7} N_{13} S_{1}$
	543.2654	-3.9	-1.5	$C_{16} H_{43} O_{12} N_{6} S_{1}$
	543.2611	4.1	15.5	$C_{32} H_{39} N_{4} S_{2}$
	543.2656	-4.2	7.0	$C_{23} H_{41} O_{4} N_{7} S_{2}$
	543.2656	-4.2	1.5	$C_{24} H_{47} O_{9} S_{2}$
	543.2609	4.4	7.0	$C_{25} H_{41} O_{8} N_{3} S_{1}$
	543.2609	4.5	12.5	$C_{24} H_{35} O_{3} N_{10} S_{1}$
	543.2658	-4.6	10.0	$C_{31} H_{45} O_{1} N_{1} S_{3}$
	543.2607	4.8	4.0	$C_{17} H_{37} O_{11} N_{9}$
	543.2607	4.8	9.5	$C_{16} H_{31} O_{6} N_{16}$

aet9-20-1 MW=556?
(DCM)/MeOH + NH4OAc

Compound 24

EPSRC National Centre Swansea
LTQ Orbitrap XL

adam throup
14/01/2013 16:43:46

BRASHE247-OJ-HNESP #27-50 RT: 0.72-1.28 AV: 22 SM: 7G NL: 7.74E7
T: FTMS + p NSI Full ms [120.00-2000.00]

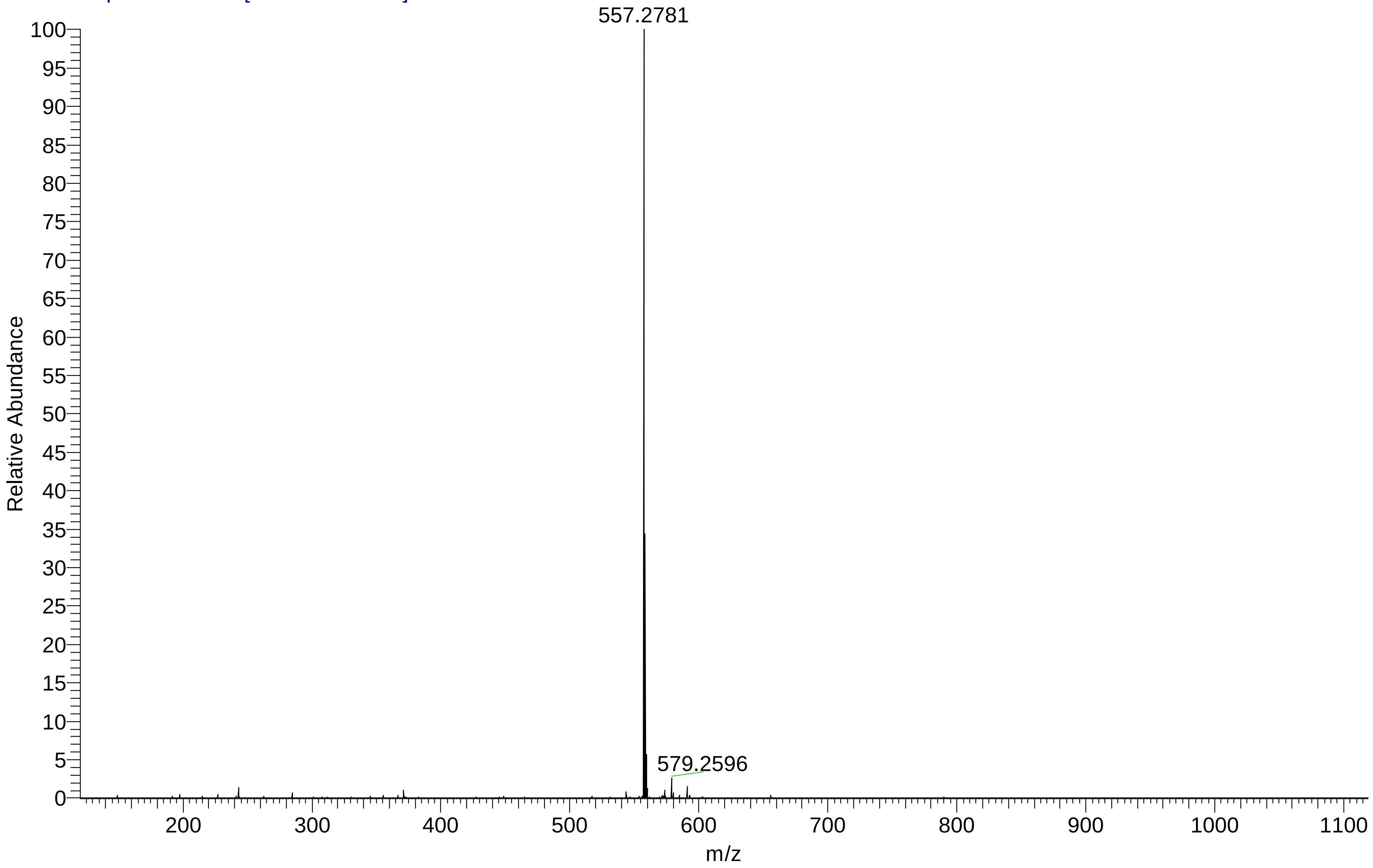

aet9-20-1 MW=556?
(DCM)/MeOH + NH4OAc

EPSRC National Centre Swansea
LTQ Orbitrap XL

adam throup
14/01/2013 16:43:46

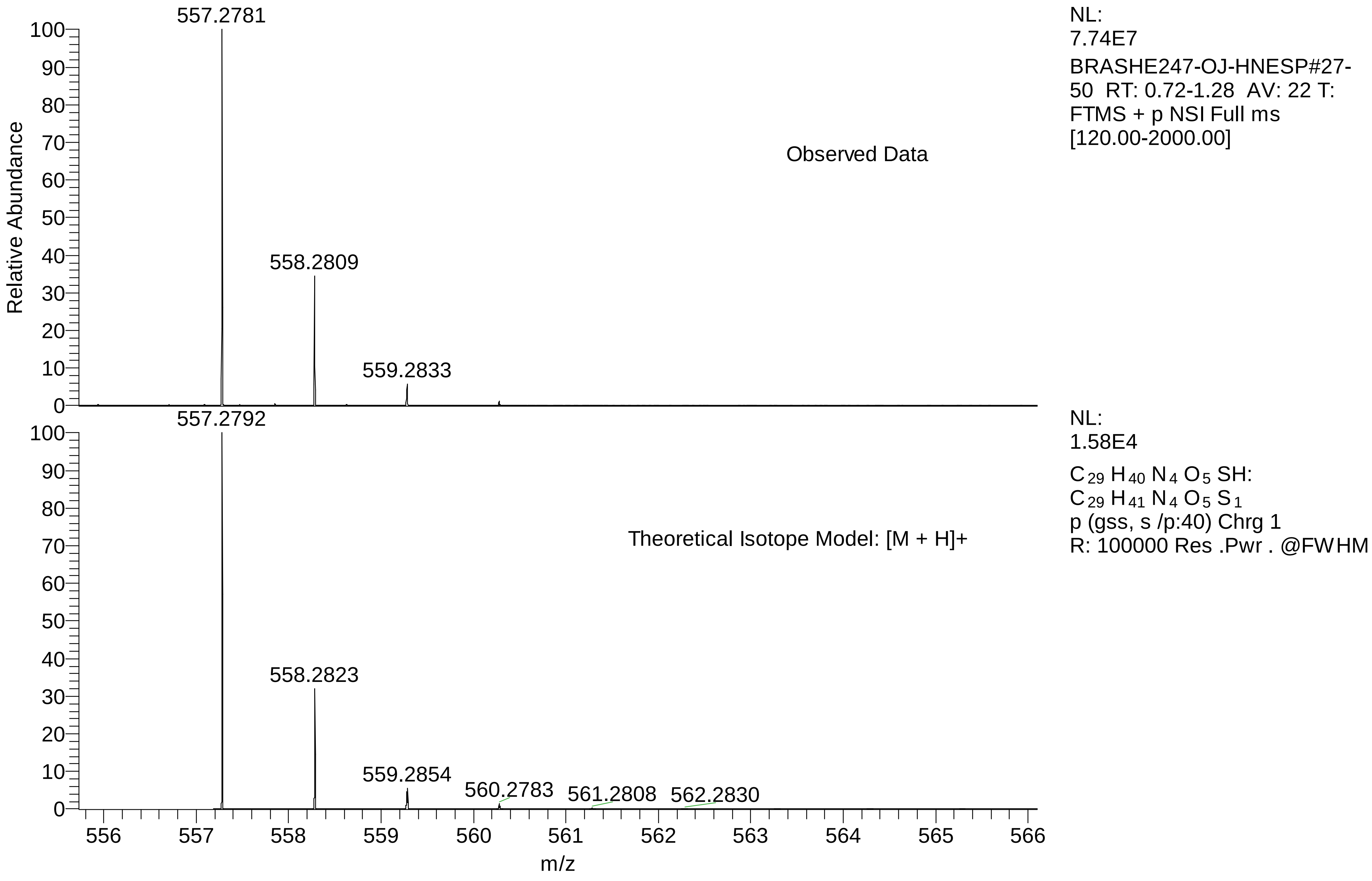

Isotope: **Min. .. Max.**
14 N **0....14**
16 O **0....15**
12 C **0....60**
1 H **0....80**
23 Na **0....0**
32 S **0....5**
Tolerance Window: **+- 5.00 ppm**
Db/Ring Equiv: **-3.. 100** **N-Rule:** **Do not use**
Fits: **200** **Charge:** **1**

**Mass**	**Theoretical Mass**	**Delta [ppm]**	**RDB**	**Composition**
557.2781	557.2781	0.0	2.0	$C_{20} H_{47} O_1 N_9 S_4$
	557.2781	0.1	15.0	$C_{35} H_{43} O_1 N_1 S_2$
	557.2779	0.4	6.5	$C_{28} H_{45} O_9 S_1$
	557.2779	0.4	12.0	$C_{27} H_{39} O_4 N_7 S_1$
	557.2784	-0.5	-0.5	$C_{13} H_{41} O_{10} N_{12} S_1$
	557.2777	0.7	3.5	$C_{20} H_{41} O_{12} N_6$
	557.2777	0.7	9.0	$C_{19} H_{35} O_7 N_{13}$
	557.2785	-0.8	21.0	$C_{35} H_{35} O_2 N_5$
	557.2786	-0.8	8.0	$C_{20} H_{39} O_2 N_{13} S_2$
	557.2786	-0.8	2.5	$C_{21} H_{45} O_7 N_6 S_2$
	557.2787	-1.2	5.5	$C_{29} H_{49} O_4 S_3$
	557.2774	1.2	6.0	$C_{27} H_{47} O_3 N_3 S_3$
	557.2772	1.6	-2.5	$C_{20} H_{49} O_{11} N_2 S_2$
	557.2772	1.6	3.0	$C_{19} H_{43} O_6 N_9 S_2$
	557.2772	1.6	16.0	$C_{34} H_{39} O_6 N_1$
	557.2772	1.6	21.5	$C_{33} H_{33} O_1 N_8$
	557.2790	-1.7	8.5	$C_{21} H_{37} O_8 N_{10}$
	557.2790	-1.7	3.0	$C_{22} H_{43} O_{13} N_3$
	557.2792	-2.0	17.0	$C_{28} H_{35} N_{11} S_1$
	557.2792	-2.0	11.5	$C_{29} H_{41} O_5 N_4 S_1$
	557.2793	-2.1	-1.5	$C_{14} H_{45} O_5 N_{12} S_3$
	557.2794	-2.4	1.5	$C_{22} H_{49} O_2 N_6 S_4$
	557.2768	2.4	-3.0	$C_{19} H_{51} O_5 N_5 S_4$
	557.2767	2.4	2.5	$C_{18} H_{45} N_{12} S_4$
	557.2767	2.5	15.5	$C_{33} H_{41} N_4 S_2$
	557.2765	2.8	7.0	$C_{26} H_{43} O_8 N_3 S_1$
	557.2765	2.8	12.5	$C_{25} H_{37} O_3 N_{10} S_1$
	557.2797	-2.9	-1.0	$C_{15} H_{43} O_{11} N_9 S_1$
	557.2764	3.1	4.0	$C_{18} H_{39} O_{11} N_9$
	557.2799	-3.2	20.5	$C_{37} H_{37} O_3 N_2$
	557.2799	-3.2	7.5	$C_{22} H_{41} O_3 N_{10} S_2$

Mass	Theoretical Mass	Delta [ppm]	RDB	Composition
	557.2799	-3.2	2.0	$C_{23} H_{47} O_8 N_3 S_2$
	557.2801	-3.6	10.5	$C_{30} H_{45} N_4 S_3$
	557.2801	-3.6	-2.5	$C_{15} H_{49} N_{12} S_5$
	557.2761	3.7	6.5	$C_{25} H_{45} O_2 N_6 S_3$
	557.2759	4.0	-2.0	$C_{18} H_{47} O_{10} N_5 S_2$
	557.2759	4.0	3.5	$C_{17} H_{41} O_5 N_{12} S_2$
	557.2758	4.0	16.5	$C_{32} H_{37} O_5 N_4$
	557.2758	4.1	22.0	$C_{31} H_{31} N_{11}$
	557.2804	-4.1	13.5	$C_{22} H_{33} O_4 N_{14}$
	557.2804	-4.1	8.0	$C_{23} H_{39} O_9 N_7$
	557.2804	-4.1	2.5	$C_{24} H_{45} O_{14}$
	557.2806	-4.4	16.5	$C_{30} H_{37} O_1 N_8 S_1$
	557.2806	-4.4	11.0	$C_{31} H_{43} O_6 N_1 S_1$
	557.2806	-4.5	-2.0	$C_{16} H_{47} O_6 N_9 S_3$
	557.2756	4.5	0.5	$C_{25} H_{53} O_1 N_2 S_5$
	557.2808	-4.8	1.0	$C_{24} H_{51} O_3 N_3 S_4$
	557.2754	4.8	-2.5	$C_{17} H_{49} O_4 N_8 S_4$
	557.2754	4.9	10.5	$C_{32} H_{45} O_4 S_2$

Compound 25

aet7-16 MW=227?
(DCM)/MeOH + NH4OAc

EPSRC National Centre Swansea
LTQ Orbitrap XL

adam throup
05/12/2011 13:10:59

BRASHE162-PV-HNESP #21-32 RT: 0.86-1.18 AV: 12 SM: 7G NL: 1.50E7
T: FTMS + p NSI Full ms [120.00-2000.00]

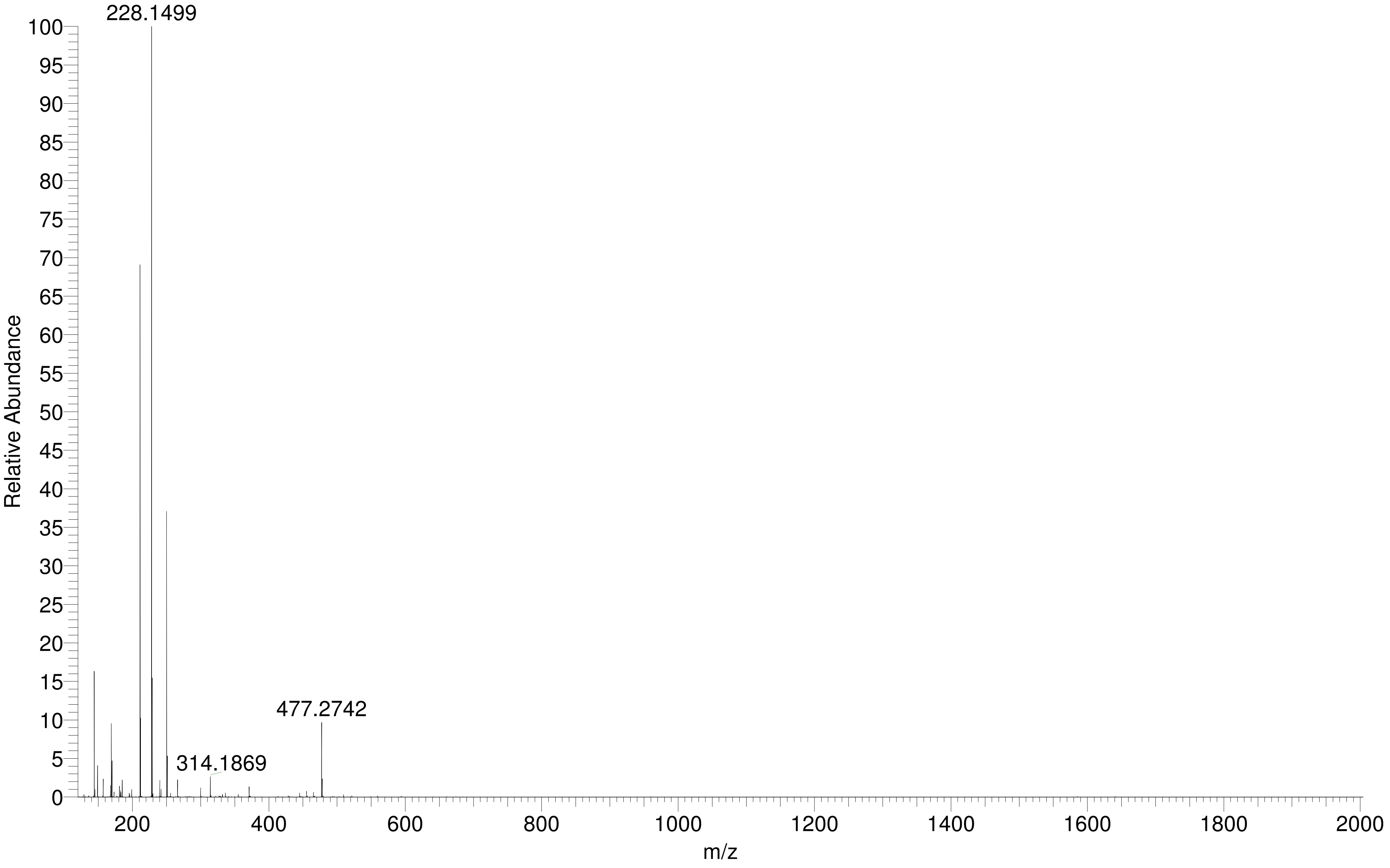

aet7-16 MW=227?
(DCM)/MeOH + NH4OAc

EPSRC National Centre Swansea
LTQ Orbitrap XL

adam throup
05/12/2011 13:10:59

BRASHE162-PV-HNESP #21-32 RT: 0.86-1.18 AV: 12 SM: 7G NL: 1.50E7
T: FTMS + p NSI Full ms [120.00-2000.00]

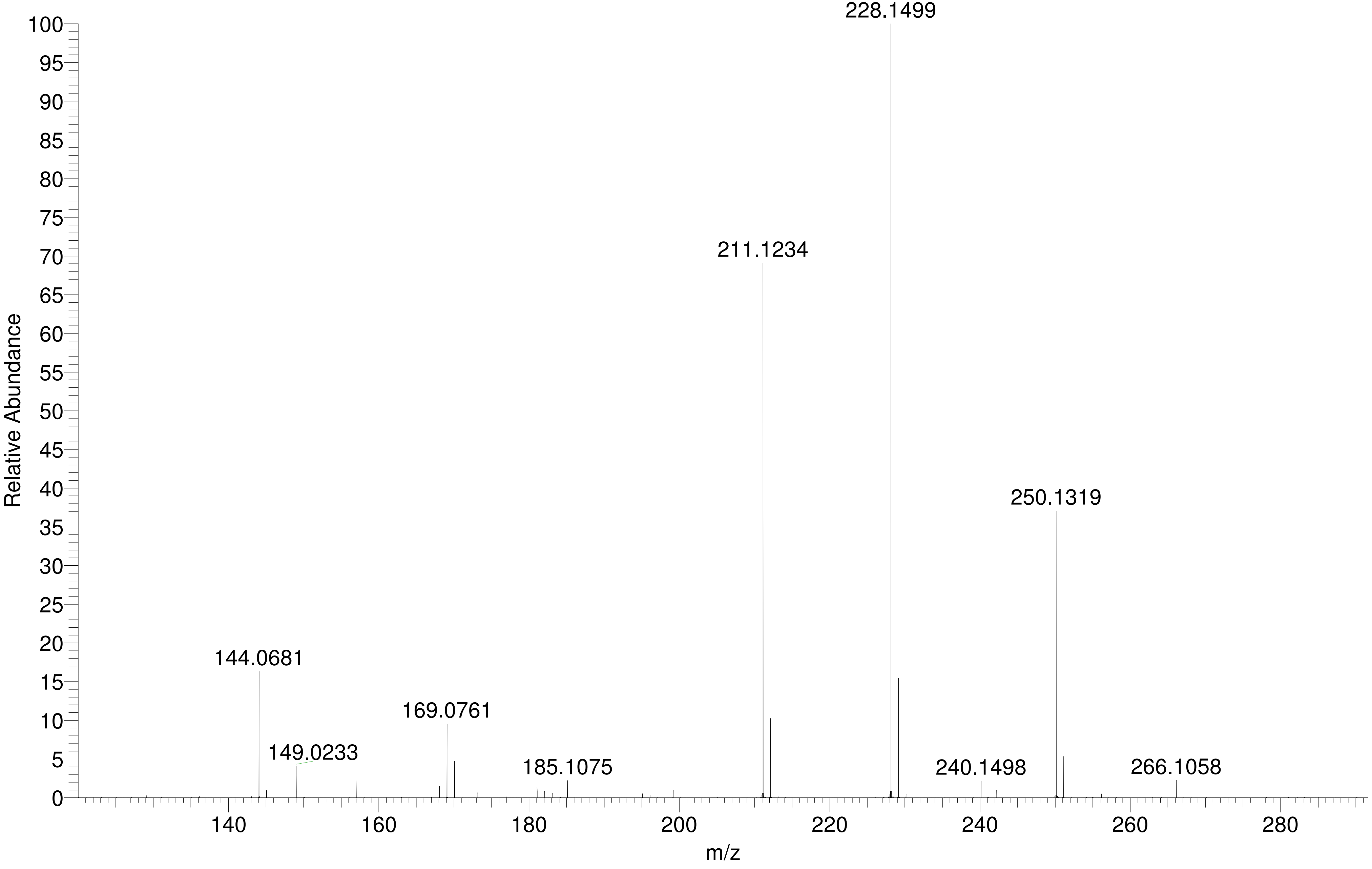

aet7-16 MW=227?
(DCM)/MeOH + NH4OAc

EPSRC National Centre Swansea
LTQ Orbitrap XL

adam throup
05/12/2011 13:10:59

BRASHE162-PV-HNESP #21-32 RT: 0.86-1.18 AV: 12 SM: 7G NL: 1.50E7
T: FTMS + p NSI Full ms [120.00-2000.00]

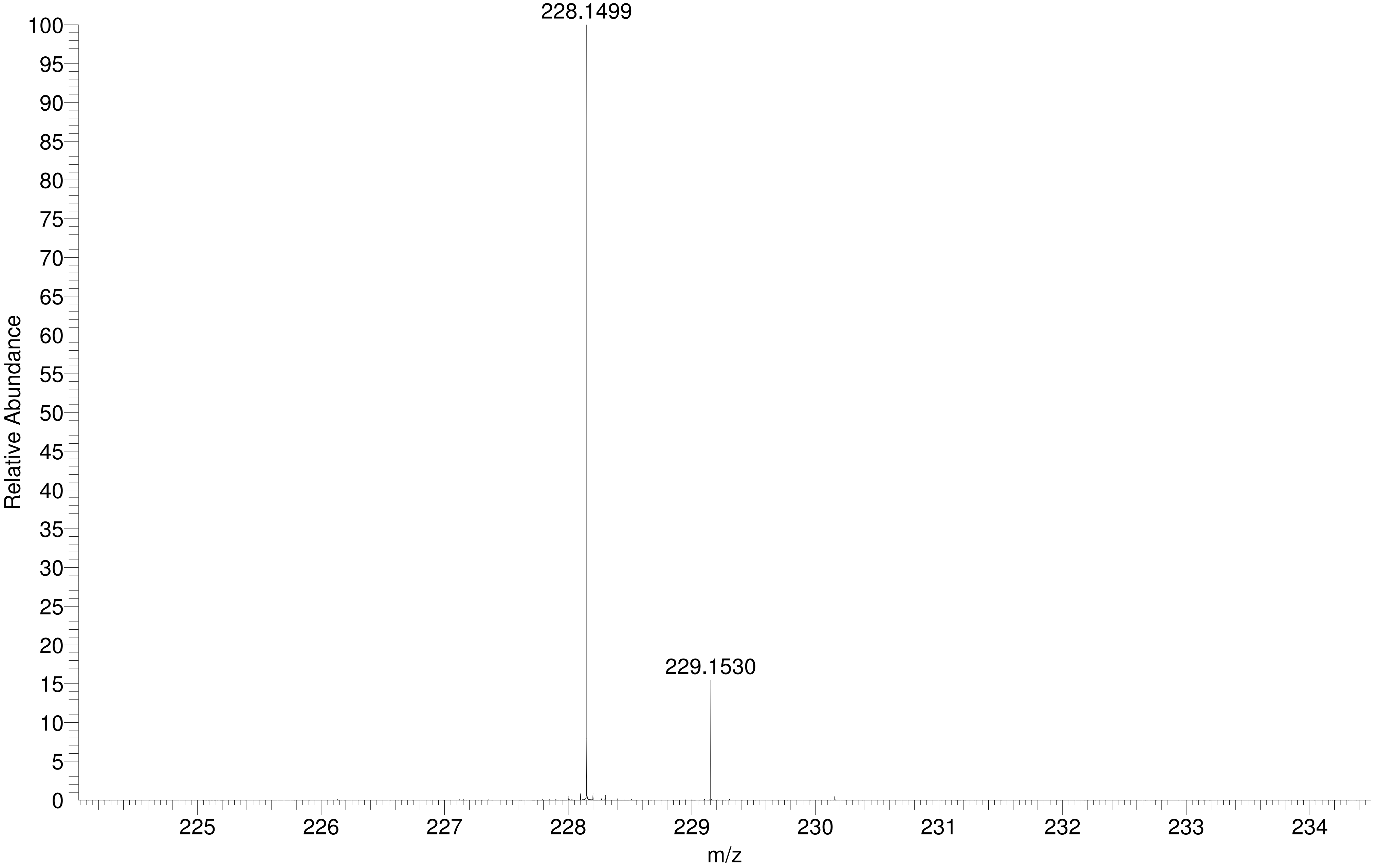

Mass	Theoretical Mass	Delta [ppm]	RDB	Composition
228.1499	228.1495	1.6	7.5	C_{14} H_{18} N_{3}
	228.1509	-4.2	7.0	C_{16} H_{20} O_{1}

Compound 26

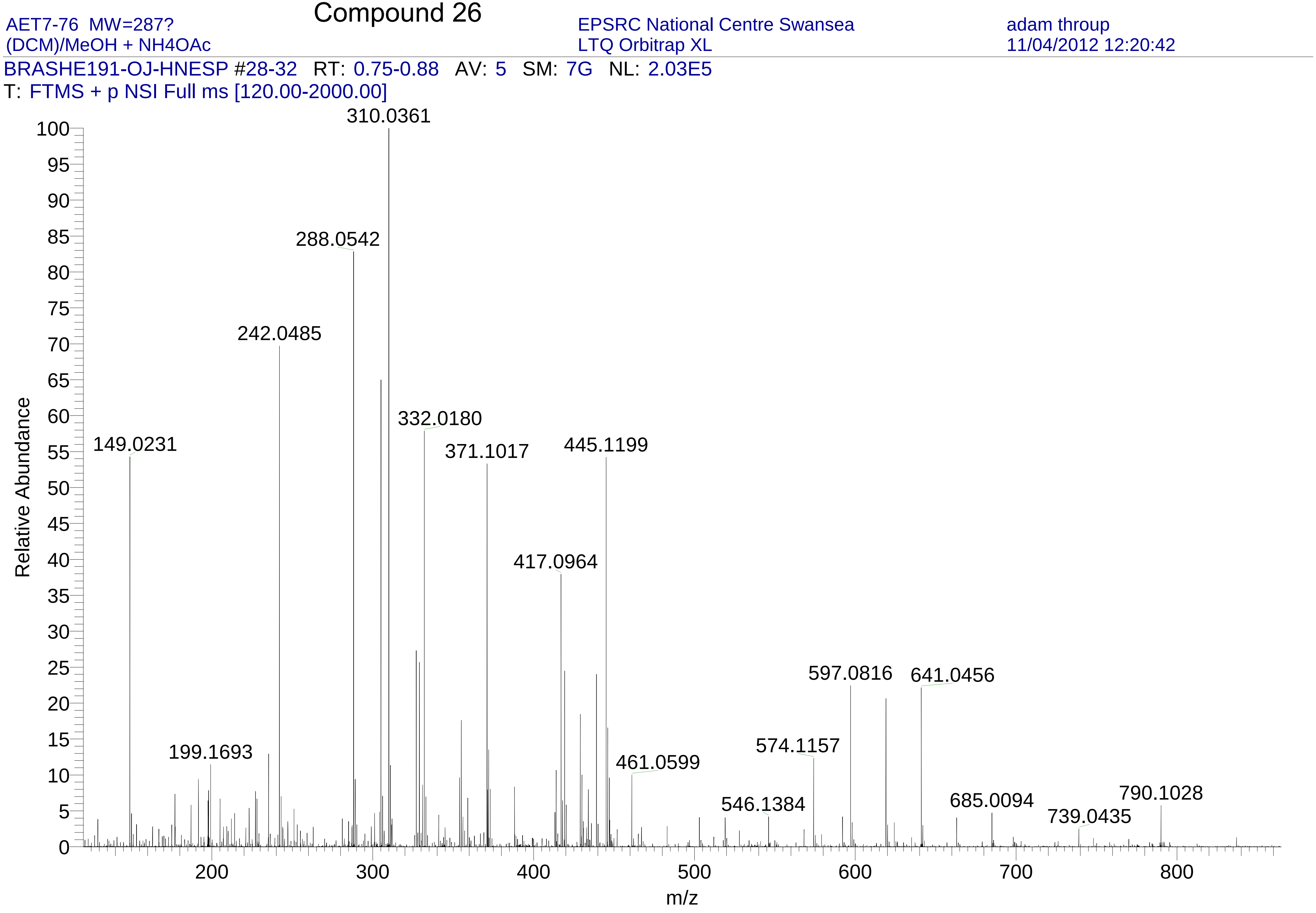

AET7-76 MW=287?
(DCM)/MeOH + NH4OAc
EPSRC National Centre Swansea
LTQ Orbitrap XL
adam throup
11/04/2012 12:20:42
BRASHE191-OJ-HNESP #28-32 RT: 0.75-0.88 AV: 5 SM: 7G NL: 2.03E5
T: FTMS + p NSI Full ms [120.00-2000.00]
310.0361
288.0542
242.0485
332.0180
149.0231
371.1017
445.1199
417.0964
597.0816
641.0456
574.1157
199.1693
461.0599
546.1384
685.0094
790.1028
739.0435
Relative Abundance
m/z

AET7-76 MW=287?
(DCM)/MeOH + NH4OAc

EPSRC National Centre Swansea
LTQ Orbitrap XL

adam throup
11/04/2012 12:20:42

SM: 7G

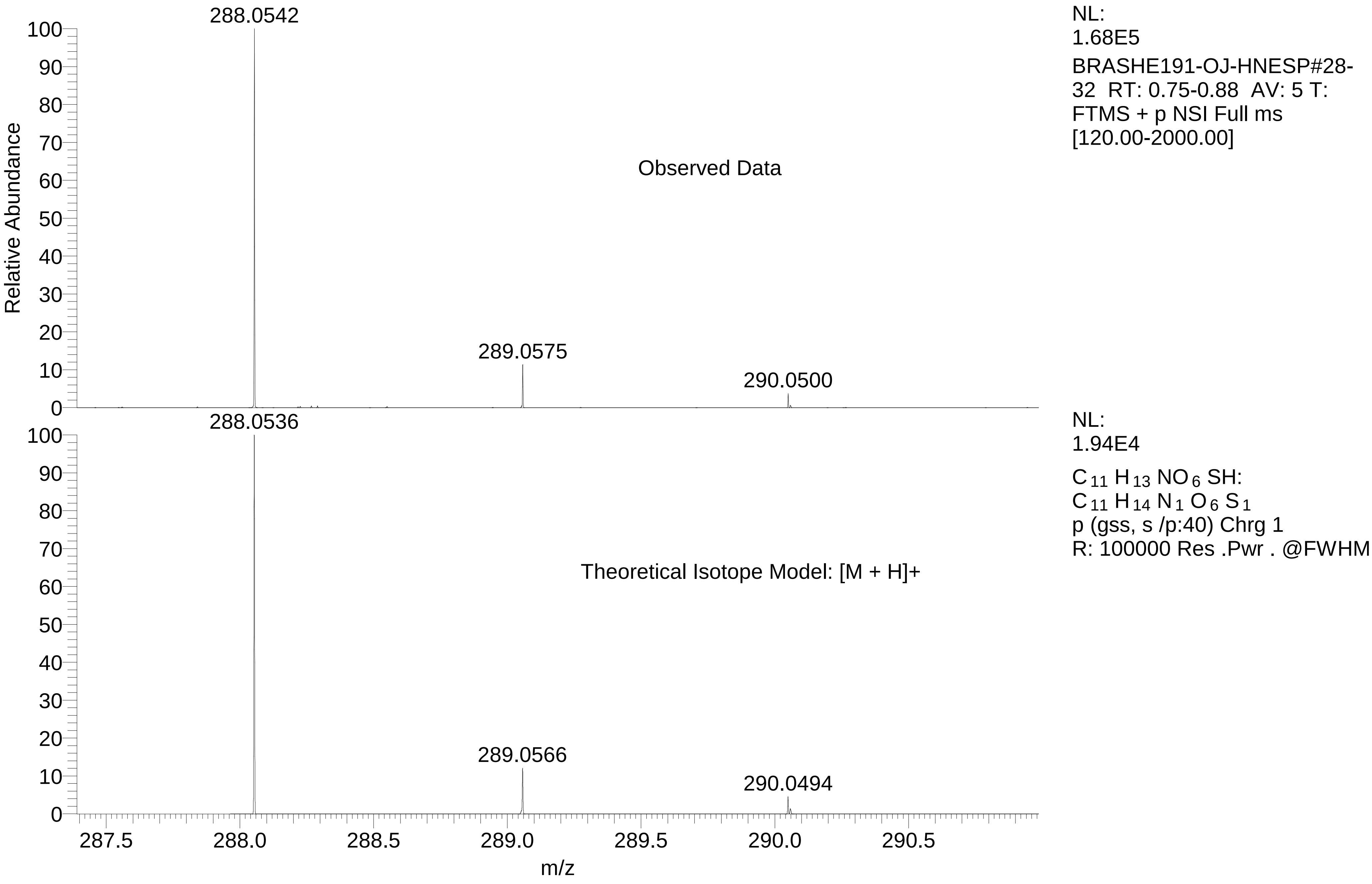

**Isotope:**	**Min. .. Max.**		
**14 N**	**0....10**		
**16 O**	**0....15**		
**12 C**	**0....65**		
**1 H**	**0....75**		
**23 Na**	**0....0**		
**32 S**	**0....2**		
**Tolerance Window:**	**+- 5.00 ppm**		
**Db/Ring Equiv:**	**-3.. 100**	**N-Rule:**	**Do not use**
**Fits:**	**500**	**Charge:**	**1**

**Mass**	**Theoretical Mass**	**Delta [ppm]**	**RDB**	**Composition**
288.0542	288.0543	-0.4	1.5	$C_4 H_{14} O_4 N_7 S_2$
	288.0536	2.0	5.5	$C_{11} H_{14} O_6 N_1 S_1$
	288.0536	2.0	11.0	$C_{10} H_8 O_1 N_8 S_1$
	288.0548	-2.1	2.0	$C_5 H_{12} O_{10} N_4$
	288.0535	2.6	-3.0	$C_4 H_{16} O_{14}$
	288.0535	2.6	2.5	$C_3 H_{10} O_9 N_7$
	288.0550	-2.7	10.5	$C_{12} H_{10} O_2 N_5 S_1$
	288.0530	4.2	2.0	$C_2 H_{12} O_3 N_{10} S_2$
	288.0529	4.4	15.0	$C_{17} H_8 O_3 N_2$
	288.0556	-4.9	19.5	$C_{20} H_6 N_3$

Compound 28

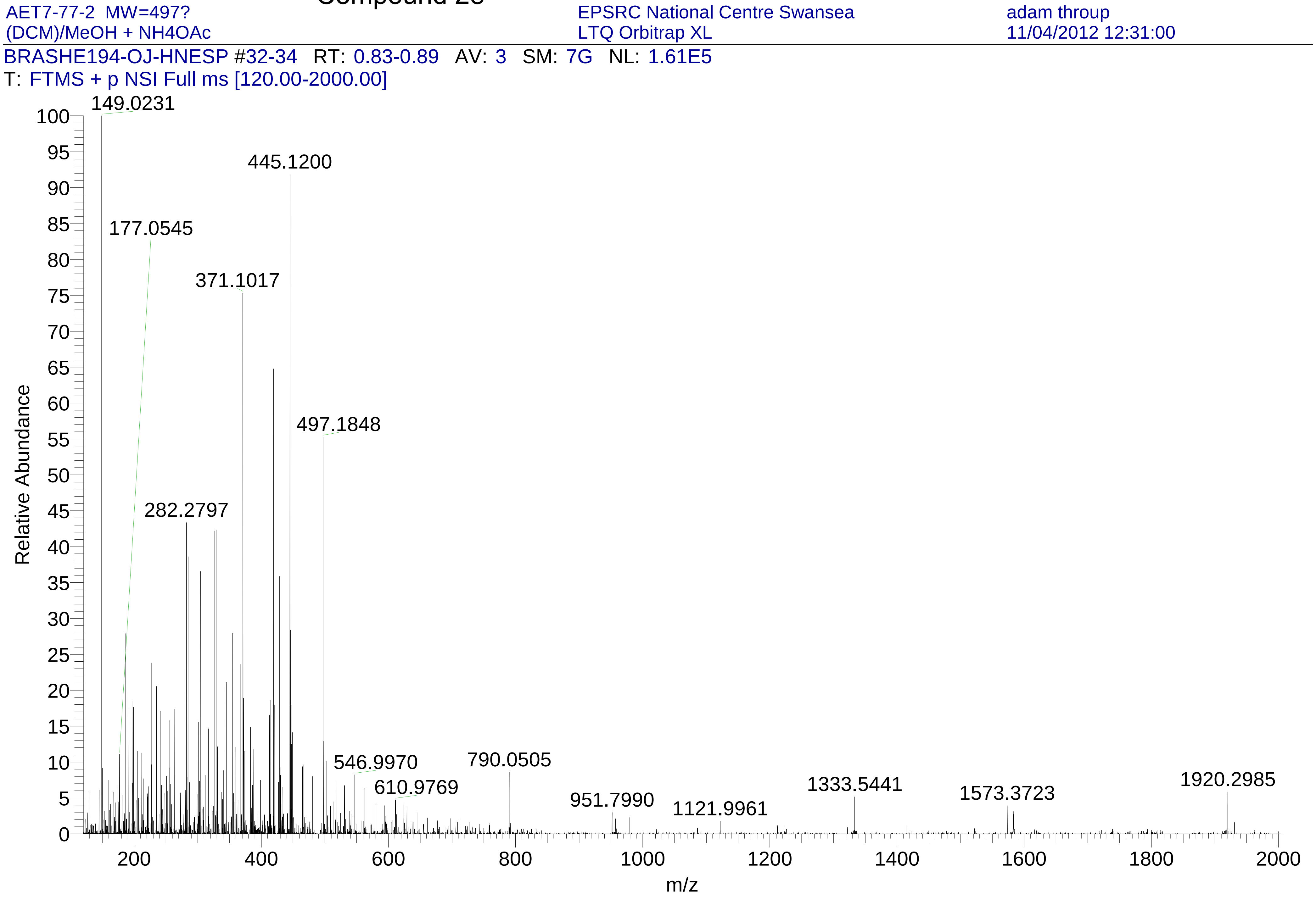
AET7-77-2 MW=497?
(DCM)/MeOH + NH4OAc
EPSRC National Centre Swansea
LTQ Orbitrap XL
adam throup
11/04/2012 12:31:00
BRASHE194-OJ-HNESP #32-34 RT: 0.83-0.89 AV: 3 SM: 7G NL: 1.61E5
T: FTMS + p NSI Full ms [120.00-2000.00]
149.0231
445.1200
177.0545
371.1017
497.1848
282.2797
546.9970
790.0505
610.9769
951.7990
1121.9961
1333.5441
1573.3723
1920.2985
Relative Abundance
m/z

AET7-77-2 MW=497?
(DCM)/MeOH + NH4OAc

EPSRC National Centre Swansea
LTQ Orbitrap XL

adam throup
11/04/2012 12:31:00

SM: 7G

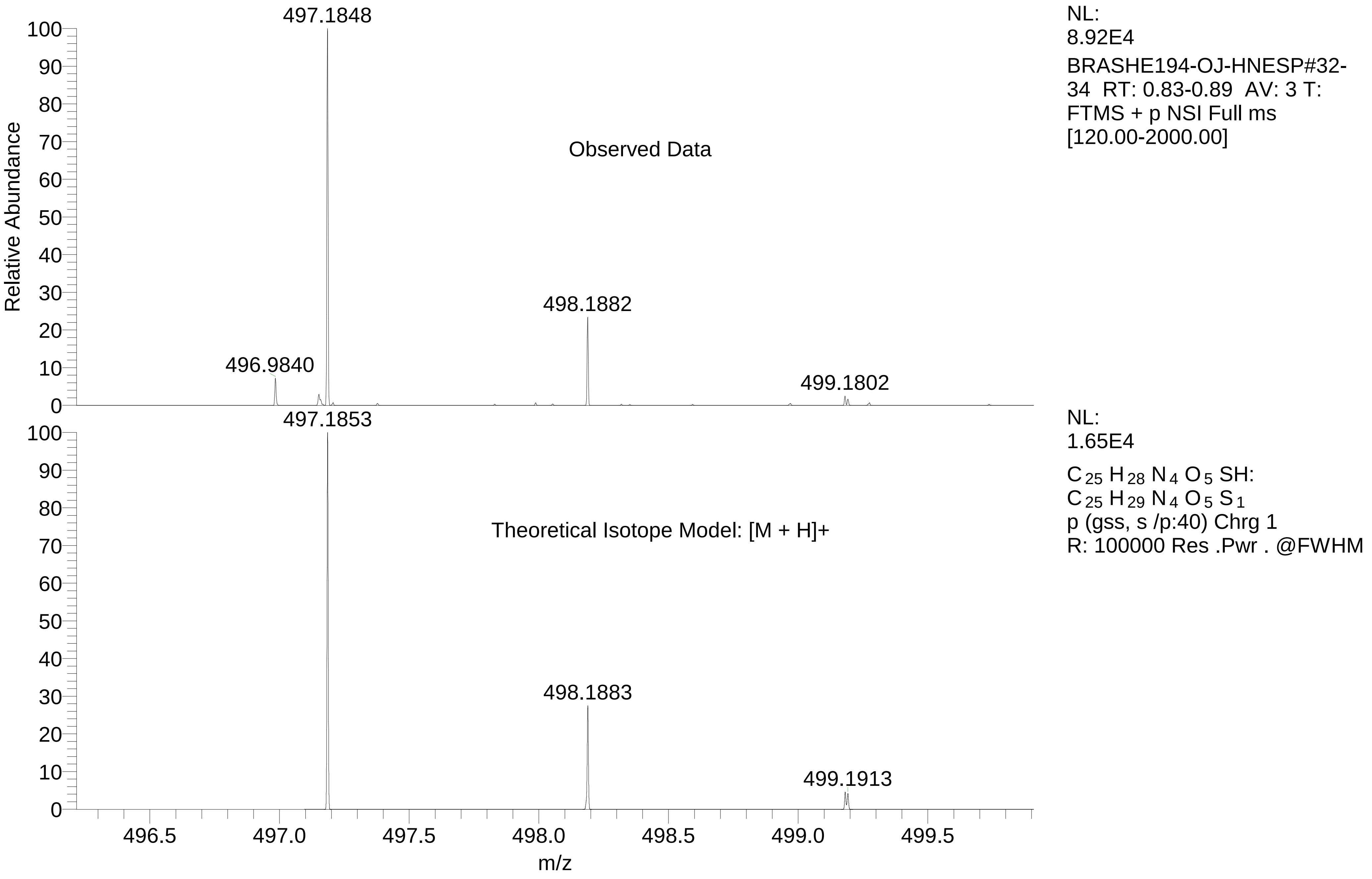

Isotope: **Min. .. Max.**
14 N **0....10**
16 O **0....15**
12 C **0....65**
1 H **0....75**
23 Na **0....0**
32 S **0....2**
Tolerance Window: **+- 5.00 ppm**
Db/Ring Equiv: **-3.. 100** **N-Rule:** **Do not use**
Fits: **500** **Charge:** **1**

**Mass**	**Theoretical Mass**	**Delta [ppm]**	**RDB**	**Composition**
497.1848	497.1847	0.3	4.5	$C_{17} H_{33} O_{7} N_{6} S_{2}$
	497.1846	0.3	23.0	$C_{31} H_{23} O_{2} N_{5}$
	497.1851	-0.7	10.5	$C_{17} H_{25} O_{8} N_{10}$
	497.1851	-0.7	5.0	$C_{18} H_{31} O_{13} N_{3}$
	497.1853	-1.0	13.5	$C_{25} H_{29} O_{5} N_{4} S_{1}$
	497.1842	1.3	17.0	$C_{31} H_{31} O_{1} N_{1} S_{2}$
	497.1840	1.6	8.5	$C_{24} H_{33} O_{9} S_{1}$
	497.1840	1.7	14.0	$C_{23} H_{27} O_{4} N_{7} S_{1}$
	497.1838	2.0	5.5	$C_{16} H_{29} O_{12} N_{6}$
	497.1858	-2.1	1.0	$C_{11} H_{31} O_{11} N_{9} S_{1}$
	497.1860	-2.4	22.5	$C_{33} H_{25} O_{3} N_{2}$
	497.1860	-2.4	9.5	$C_{18} H_{29} O_{3} N_{10} S_{2}$
	497.1860	-2.4	4.0	$C_{19} H_{35} O_{8} N_{3} S_{2}$
	497.1833	3.0	-0.5	$C_{16} H_{37} O_{11} N_{2} S_{2}$
	497.1833	3.0	5.0	$C_{15} H_{31} O_{6} N_{9} S_{2}$
	497.1833	3.0	18.0	$C_{30} H_{27} O_{6} N_{1}$
	497.1833	3.0	23.5	$C_{29} H_{21} O_{1} N_{8}$
	497.1865	-3.4	10.0	$C_{19} H_{27} O_{9} N_{7}$
	497.1865	-3.4	4.5	$C_{20} H_{33} O_{14}$
	497.1867	-3.7	18.5	$C_{26} H_{25} O_{1} N_{8} S_{1}$
	497.1867	-3.7	13.0	$C_{27} H_{31} O_{6} N_{1} S_{1}$
	497.1828	4.0	17.5	$C_{29} H_{29} N_{4} S_{2}$
	497.1826	4.4	9.0	$C_{22} H_{31} O_{8} N_{3} S_{1}$
	497.1826	4.4	14.5	$C_{21} H_{25} O_{3} N_{10} S_{1}$
	497.1825	4.7	6.0	$C_{14} H_{27} O_{11} N_{9}$
	497.1872	-4.8	0.5	$C_{13} H_{33} O_{12} N_{6} S_{1}$

Compound 29

AET7-78-2 MW=538?
(DCM)/MeOH + NH4OAc

EPSRC National Centre Swansea
LTQ Orbitrap XL

adam throup
11/04/2012 12:27:35

BRASHE193-OJ-HNESP #31-38 RT: 0.81-1.02 AV: 8 SM: 7G NL: 1.33E6
T: FTMS + p NSI Full ms [120.00-2000.00]

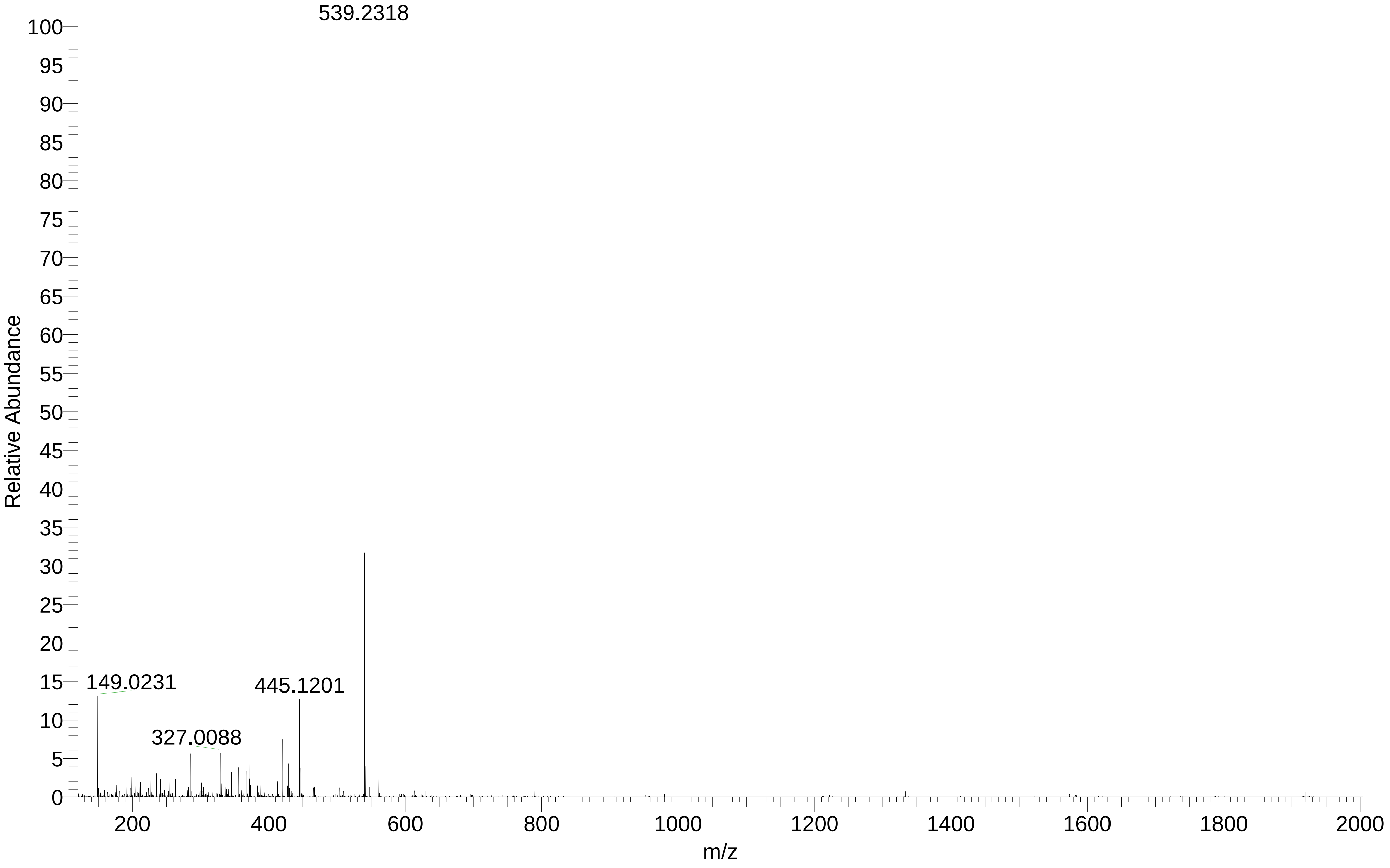

AET7-78-2 MW=538?
(DCM)/MeOH + NH4OAc

EPSRC National Centre Swansea
LTQ Orbitrap XL

adam throup
11/04/2012 12:27:35

SM: 7G

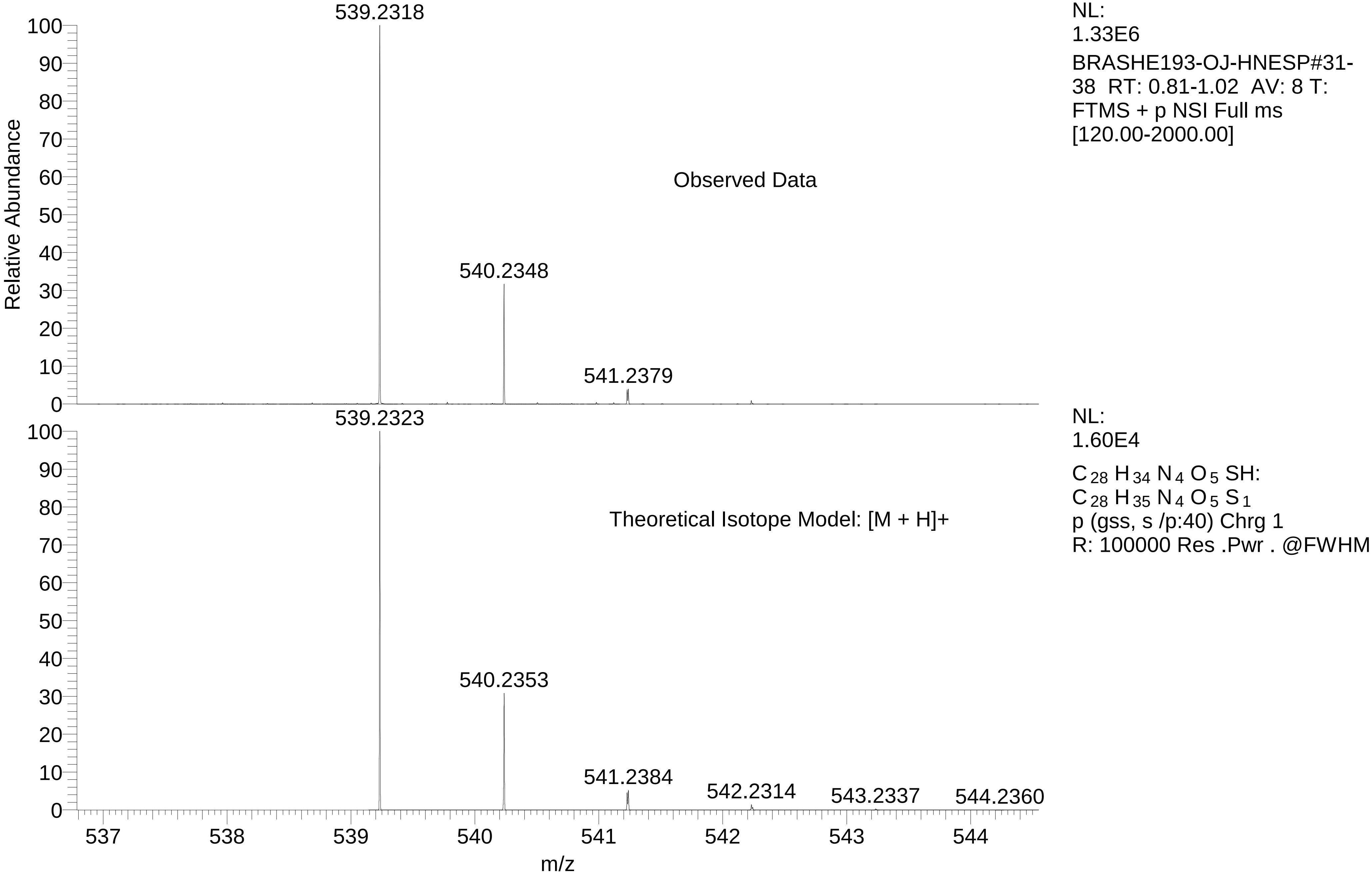

Isotope: **Min. .. Max.**
14 N **0....10**
16 O **0....15**
12 C **0....65**
1 H **0....75**
23 Na **0....0**
32 S **0....2**
Tolerance Window: **+- 5.00 ppm**
Db/Ring Equiv: **-3.. 100** **N-Rule:** **Do not use**
Fits: **500** **Charge:** **1**

**Mass**	**Theoretical Mass**	**Delta [ppm]**	**RDB**	**Composition**
539.2318	539.2316	0.3	4.5	$C_{20}H_{39}O_7N_6S_2$
	539.2316	0.4	23.0	$C_{34}H_{29}O_2N_5$
	539.2321	-0.5	10.5	$C_{20}H_{31}O_8N_{10}$
	539.2321	-0.5	5.0	$C_{21}H_{37}O_{13}N_3$
	539.2323	-0.9	13.5	$C_{28}H_{35}O_5N_4S_1$
	539.2311	1.3	17.0	$C_{34}H_{37}O_1N_1S_2$
	539.2309	1.6	8.5	$C_{27}H_{39}O_9S_1$
	539.2309	1.6	14.0	$C_{26}H_{33}O_4N_7S_1$
	539.2328	-1.8	1.0	$C_{14}H_{37}O_{11}N_9S_1$
	539.2307	2.0	5.5	$C_{19}H_{35}O_{12}N_6$
	539.2329	-2.1	22.5	$C_{36}H_{31}O_3N_2$
	539.2330	-2.1	9.5	$C_{21}H_{35}O_3N_{10}S_2$
	539.2330	-2.1	4.0	$C_{22}H_{41}O_8N_3S_2$
	539.2303	2.8	-0.5	$C_{19}H_{43}O_{11}N_2S_2$
	539.2303	2.8	5.0	$C_{18}H_{37}O_6N_9S_2$
	539.2302	2.9	18.0	$C_{33}H_{33}O_6N_1$
	539.2302	2.9	23.5	$C_{32}H_{27}O_1N_8$
	539.2334	-3.0	10.0	$C_{22}H_{33}O_9N_7$
	539.2334	-3.0	4.5	$C_{23}H_{39}O_{14}$
	539.2336	-3.3	18.5	$C_{29}H_{31}O_1N_8S_1$
	539.2336	-3.4	13.0	$C_{30}H_{37}O_6N_1S_1$
	539.2298	3.8	17.5	$C_{32}H_{35}N_4S_2$
	539.2296	4.1	9.0	$C_{25}H_{37}O_8N_3S_1$
	539.2296	4.1	14.5	$C_{24}H_{31}O_3N_{10}S_1$
	539.2341	-4.3	0.5	$C_{16}H_{39}O_{12}N_6S_1$
	539.2294	4.4	6.0	$C_{17}H_{33}O_{11}N_9$
	539.2343	-4.6	9.0	$C_{23}H_{37}O_4N_7S_2$
	539.2343	-4.6	3.5	$C_{24}H_{43}O_9S_2$

Compound 30

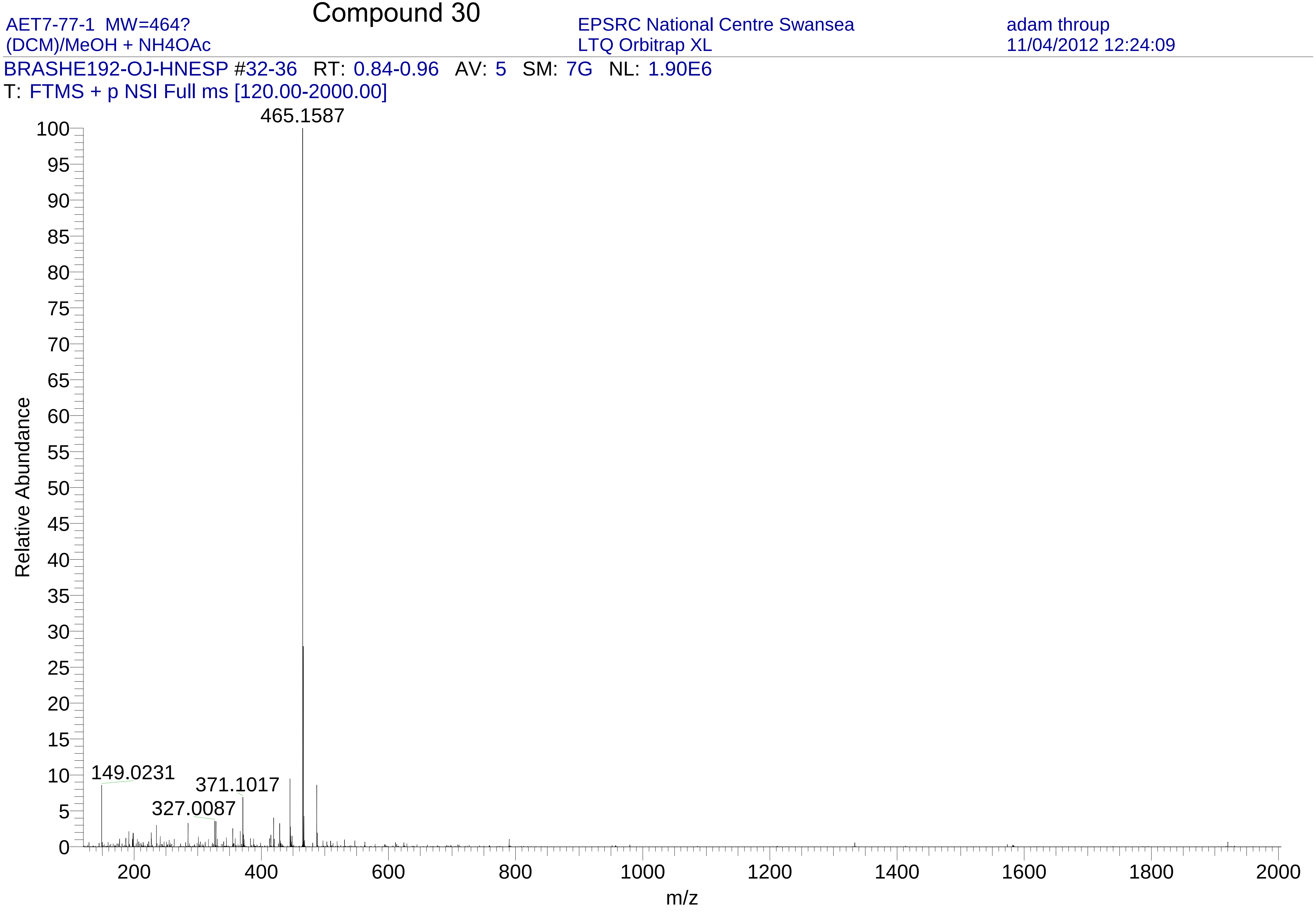

AET7-77-1 MW=464?
(DCM)/MeOH + NH4OAc
EPSRC National Centre Swansea
LTQ Orbitrap XL
adam throup
11/04/2012 12:24:09
BRASHE192-OJ-HNESP #32-36 RT: 0.84-0.96 AV: 5 SM: 7G NL: 1.90E6
T: FTMS + p NSI Full ms [120.00-2000.00]
465.1587
149.0231
371.1017
327.0087
Relative Abundance
m/z

AET7-77-1 MW=464?
(DCM)/MeOH + NH4OAc

EPSRC National Centre Swansea
LTQ Orbitrap XL

adam throup
11/04/2012 12:24:09

SM: 7G

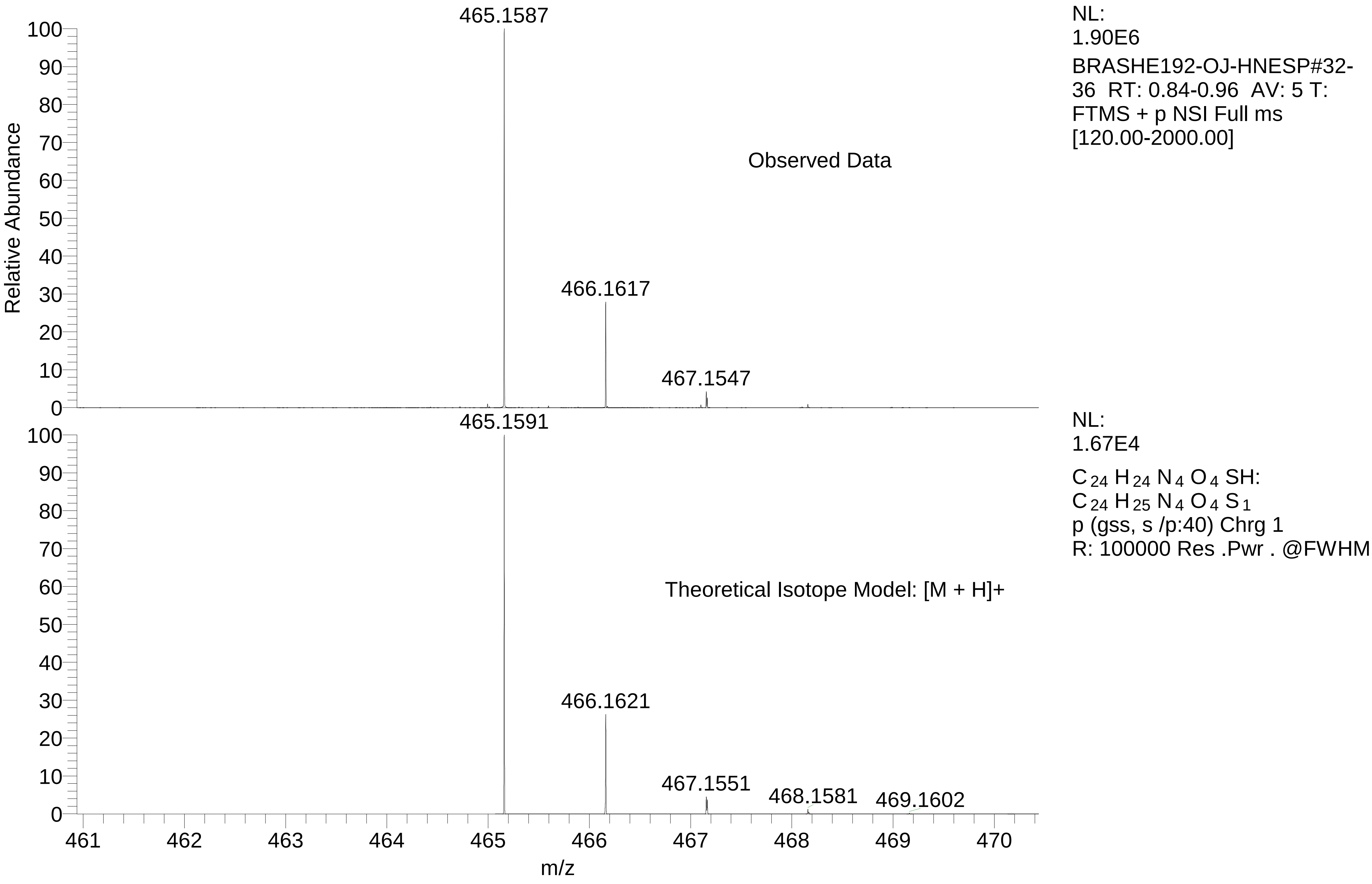

**Isotope:**	**Min. .. Max.**			
**14 N**	**0....10**			
**16 O**	**0....15**			
**12 C**	**0....65**			
**1 H**	**0....75**			
**23 Na**	**0....0**			
**32 S**	**0....2**			
**Tolerance Window:**	**+- 5.00 ppm**			
**Db/Ring Equiv:**	**-3.. 100**	**N-Rule:**	**Do not use**	
**Fits:**	**500**	**Charge:**	**1**	

**Mass**	**Theoretical Mass**	**Delta [ppm]**	**RDB**	**Composition**
465.1587	465.1589	-0.5	11.5	$C_{16} H_{21} O_{7} N_{10}$
	465.1589	-0.5	6.0	$C_{17} H_{27} O_{12} N_{3}$
	465.1585	0.5	5.5	$C_{16} H_{29} O_{6} N_{6} S_{2}$
	465.1584	0.6	24.0	$C_{30} H_{19} O_{1} N_{5}$
	465.1591	-0.9	14.5	$C_{24} H_{25} O_{4} N_{4} S_{1}$
	465.1583	0.9	-3.0	$C_{9} H_{31} O_{14} N_{5} S_{1}$
	465.1579	1.6	18.0	$C_{30} H_{27} N_{1} S_{2}$
	465.1596	-2.0	2.0	$C_{10} H_{27} O_{10} N_{9} S_{1}$
	465.1578	2.0	9.5	$C_{23} H_{29} O_{8} S_{1}$
	465.1578	2.0	15.0	$C_{22} H_{23} O_{3} N_{7} S_{1}$
	465.1598	-2.3	23.5	$C_{32} H_{21} O_{2} N_{2}$
	465.1598	-2.3	10.5	$C_{17} H_{25} O_{2} N_{10} S_{2}$
	465.1598	-2.4	5.0	$C_{18} H_{31} O_{7} N_{3} S_{2}$
	465.1576	2.4	6.5	$C_{15} H_{25} O_{11} N_{6}$
	465.1603	-3.4	11.0	$C_{18} H_{23} O_{8} N_{7}$
	465.1603	-3.4	5.5	$C_{19} H_{29} O_{13}$
	465.1571	3.4	0.5	$C_{15} H_{33} O_{10} N_{2} S_{2}$
	465.1571	3.4	6.0	$C_{14} H_{27} O_{5} N_{9} S_{2}$
	465.1571	3.5	19.0	$C_{29} H_{23} O_{5} N_{1}$
	465.1571	3.5	24.5	$C_{28} H_{17} N_{8}$
	465.1604	-3.7	19.5	$C_{25} H_{21} N_{8} S_{1}$
	465.1604	-3.8	14.0	$C_{26} H_{27} O_{5} N_{1} S_{1}$
	465.1569	3.8	-2.5	$C_{7} H_{29} O_{13} N_{8} S_{1}$
	465.1610	-4.8	1.5	$C_{12} H_{29} O_{11} N_{6} S_{1}$
	465.1564	4.9	10.0	$C_{21} H_{27} O_{7} N_{3} S_{1}$
	465.1564	4.9	15.5	$C_{20} H_{21} O_{2} N_{10} S_{1}$

Compound 31

AET7-78-1 MW=507?
(DCM)/MeOH + NH4OAc

EPSRC National Centre Swansea
LTQ Orbitrap XL

adam throup
11/04/2012 12:34:26

BRASHE195-OJ-HNESP #28-34 RT: 0.72-0.86 AV: 7 SM: 7G NL: 9.10E5
T: FTMS + p NSI Full ms [120.00-2000.00]

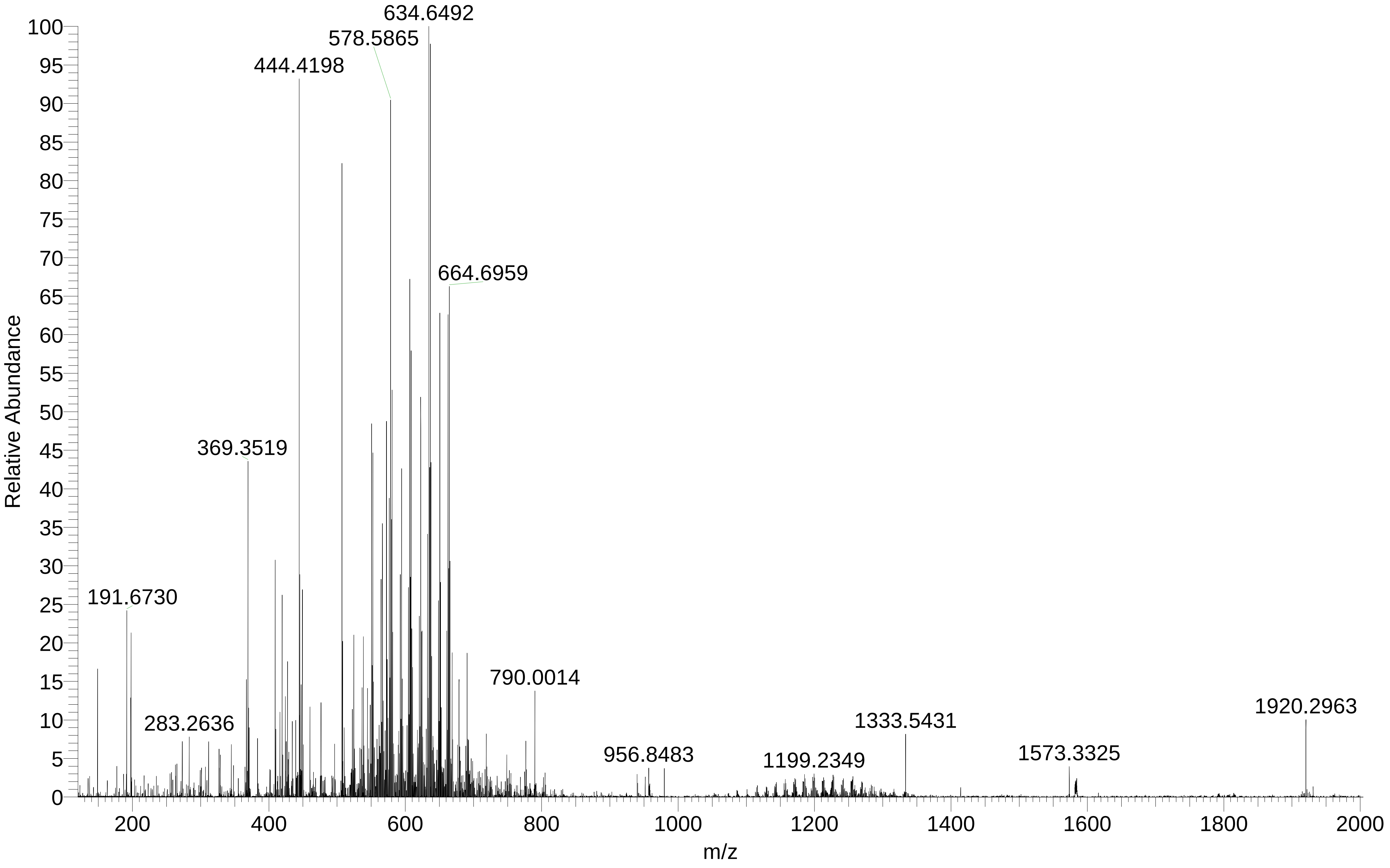

AET7-78-1 MW=507?
(DCM)/MeOH + NH4OAc

EPSRC National Centre Swansea
LTQ Orbitrap XL

adam throup
11/04/2012 12:34:26

BRASHE195-OJ-HNESP #28-34 RT: 0.72-0.86 AV: 7 SM: 7G NL: 9.10E5
T: FTMS + p NSI Full ms [120.00-2000.00]

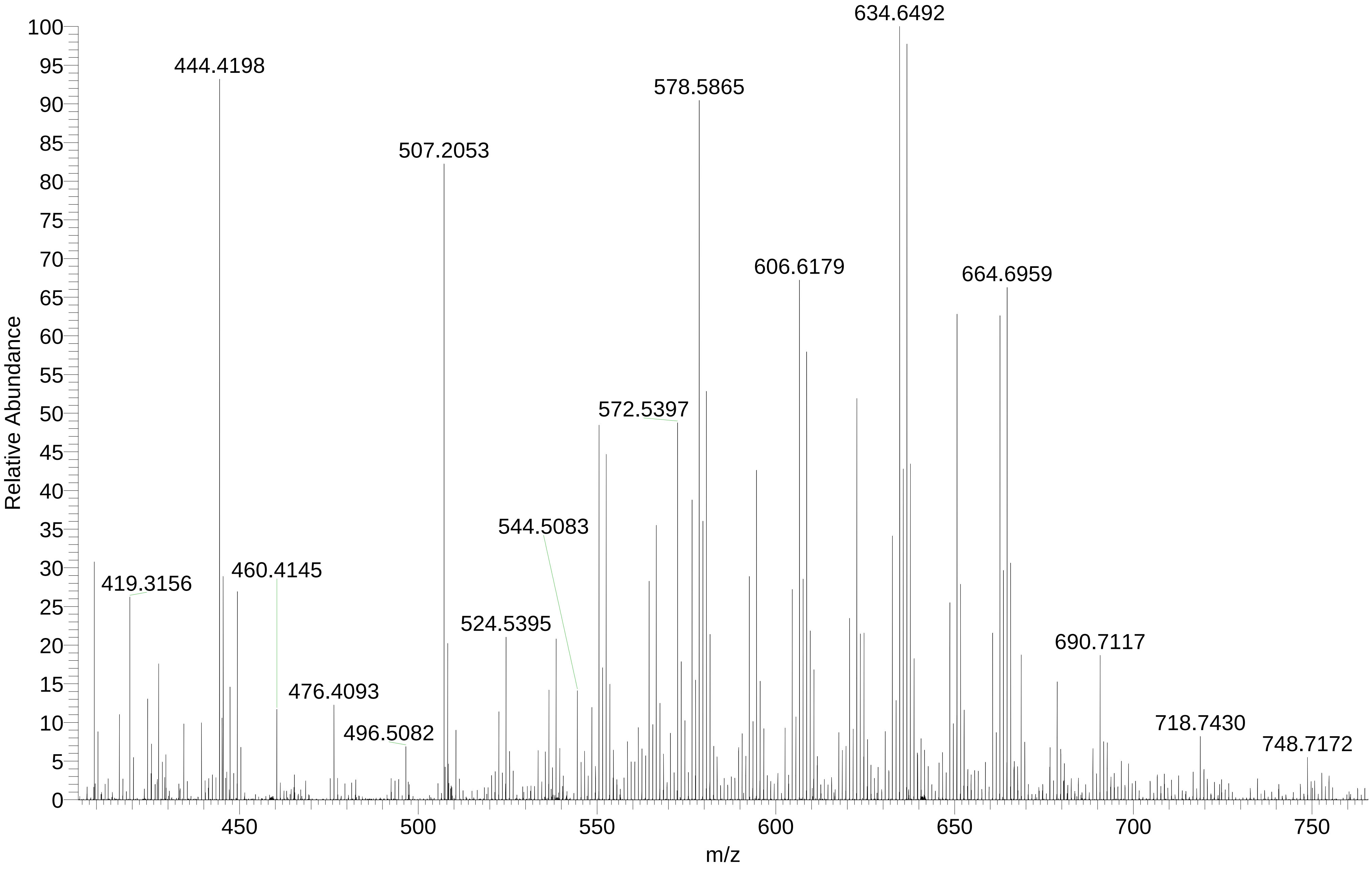

AET7-78-1 MW=507?
(DCM)/MeOH + NH4OAc

EPSRC National Centre Swansea
LTQ Orbitrap XL

adam throup
11/04/2012 12:34:26

SM: 7G

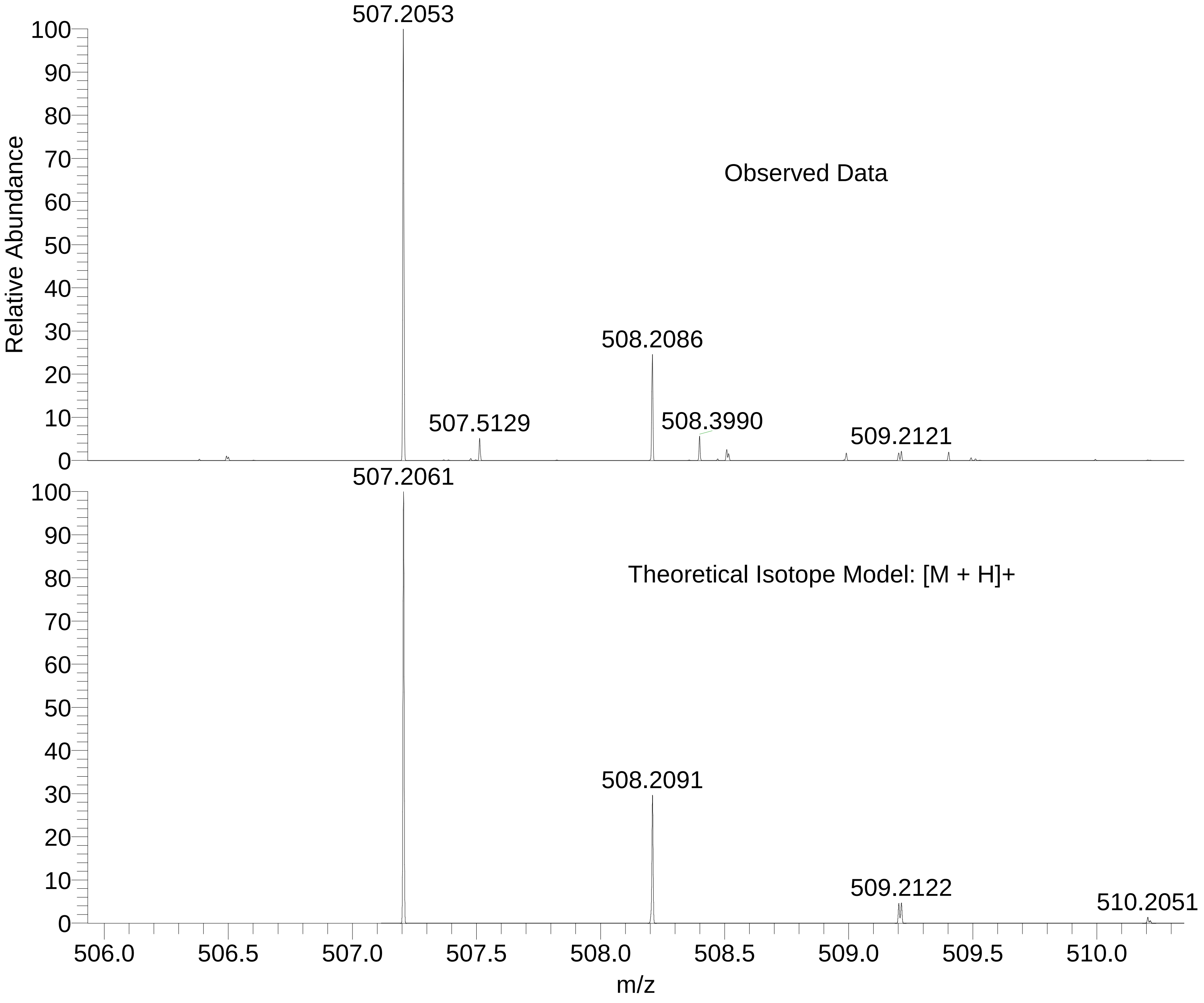

NL:
7.48E5
BRASHE195-OJ-HNESP#28-34 RT: 0.72-0.86 AV: 7 T: FTMS + p NSI Full ms [120.00-2000.00]

NL:
1.62E4
$C_{27}H_{30}N_4O_4SH$:
$C_{27}H_{31}N_4O_4S_1$
p (gss, s /p:40) Chrg 1
R: 100000 Res .Pwr . @FWHM

**Isotope:**	**Min. .. Max.**		
**14 N**	**0....10**		
**16 O**	**0....15**		
**12 C**	**0....65**		
**1 H**	**0....75**		
**23 Na**	**0....0**		
**32 S**	**0....2**		
**Tolerance Window:**	**+- 5.00 ppm**		
**Db/Ring Equiv:**	**-3.. 100**	**N-Rule:**	**Do not use**
**Fits:**	**500**	**Charge:**	**1**

**Mass**	**Theoretical Mass**	**Delta [ppm]**	**RDB**	**Composition**
507.2053	507.2054	-0.1	24.0	$C_{33} H_{25} O_{1} N_{5}$
	507.2052	0.2	-3.0	$C_{12} H_{37} O_{14} N_{5} S_{1}$
	507.2054	-0.2	5.5	$C_{19} H_{35} O_{6} N_{6} S_{2}$
	507.2049	0.8	18.0	$C_{33} H_{33} N_{1} S_{2}$
	507.2059	-1.1	11.5	$C_{19} H_{27} O_{7} N_{10}$
	507.2059	-1.1	6.0	$C_{20} H_{33} O_{12} N_{3}$
	507.2047	1.2	9.5	$C_{26} H_{35} O_{8} S_{1}$
	507.2047	1.2	15.0	$C_{25} H_{29} O_{3} N_{7} S_{1}$
	507.2061	-1.5	14.5	$C_{27} H_{31} O_{4} N_{4} S_{1}$
	507.2045	1.5	6.5	$C_{18} H_{31} O_{11} N_{6}$
	507.2041	2.4	0.5	$C_{18} H_{39} O_{10} N_{2} S_{2}$
	507.2041	2.4	6.0	$C_{17} H_{33} O_{5} N_{9} S_{2}$
	507.2066	-2.5	2.0	$C_{13} H_{33} O_{10} N_{9} S_{1}$
	507.2040	2.5	19.0	$C_{32} H_{29} O_{5} N_{1}$
	507.2040	2.5	24.5	$C_{31} H_{23} N_{8}$
	507.2067	-2.8	23.5	$C_{35} H_{27} O_{2} N_{2}$
	507.2039	2.8	-2.5	$C_{10} H_{35} O_{13} N_{8} S_{1}$
	507.2067	-2.8	10.5	$C_{20} H_{31} O_{2} N_{10} S_{2}$
	507.2067	-2.8	5.0	$C_{21} H_{37} O_{7} N_{3} S_{2}$
	507.2072	-3.8	11.0	$C_{21} H_{29} O_{8} N_{7}$
	507.2072	-3.8	5.5	$C_{22} H_{35} O_{13}$
	507.2034	3.8	10.0	$C_{24} H_{33} O_{7} N_{3} S_{1}$
	507.2034	3.8	15.5	$C_{23} H_{27} O_{2} N_{10} S_{1}$
	507.2074	-4.1	19.5	$C_{28} H_{27} N_{8} S_{1}$
	507.2074	-4.1	14.0	$C_{29} H_{33} O_{5} N_{1} S_{1}$
	507.2032	4.2	1.5	$C_{17} H_{35} O_{15} N_{2}$
	507.2032	4.2	7.0	$C_{16} H_{29} O_{10} N_{9}$

Compound 32

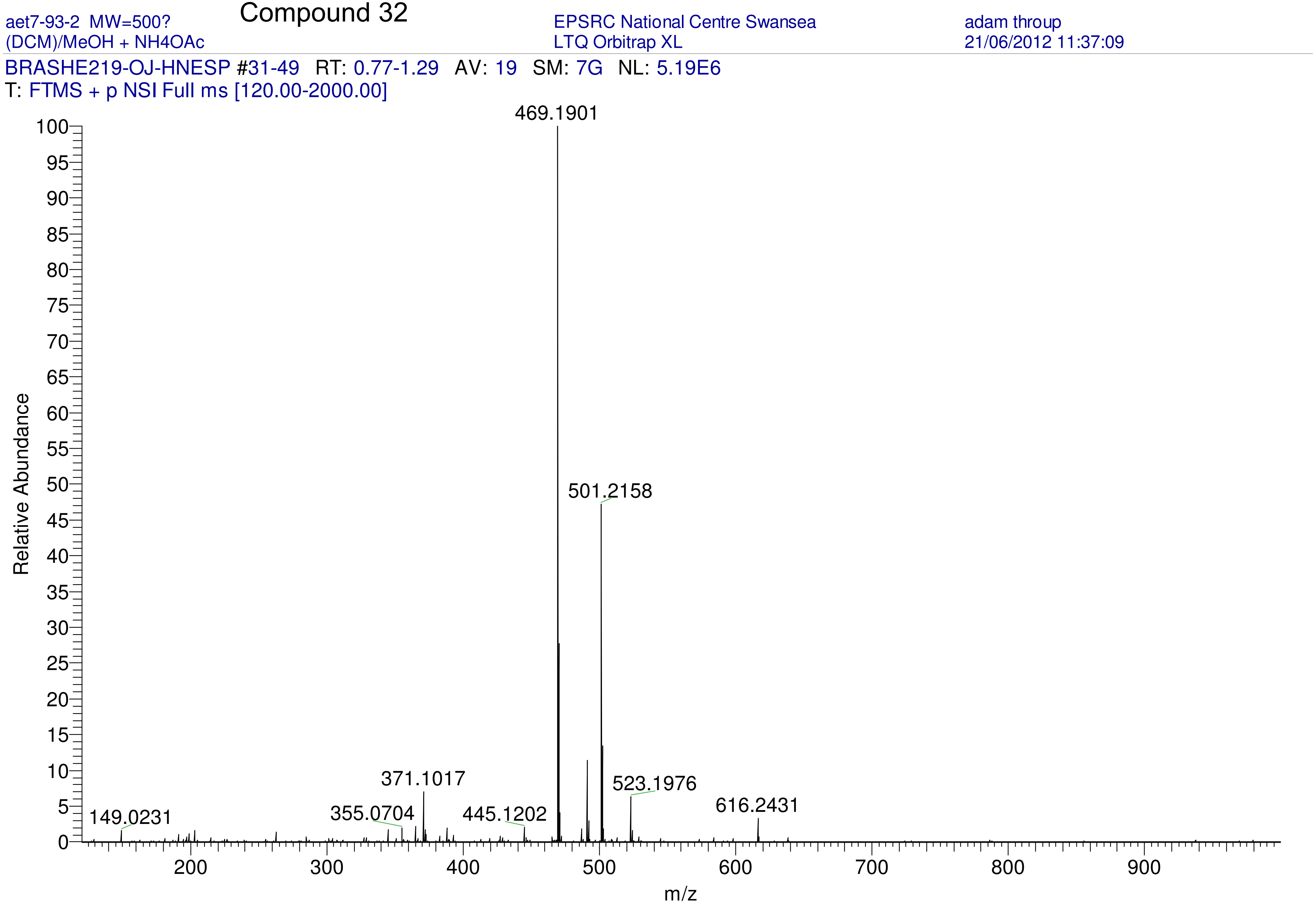

aet7-93-2 MW=500?
(DCM)/MeOH + NH4OAc
EPSRC National Centre Swansea
LTQ Orbitrap XL
adam throup
21/06/2012 11:37:09
BRASHE219-OJ-HNESP #31-49 RT: 0.77-1.29 AV: 19 SM: 7G NL: 5.19E6
T: FTMS + p NSI Full ms [120.00-2000.00]
469.1901
501.2158
371.1017
523.1976
616.2431
355.0704
445.1202
149.0231
Relative Abundance
m/z

aet7-93-2 MW=500?
(DCM)/MeOH + NH4OAc

EPSRC National Centre Swansea
LTQ Orbitrap XL

adam throup
21/06/2012 11:37:09

BRASHE219-OJ-HNESP #31-49 RT: 0.77-1.29 AV: 19 SM: 7G NL: 2.45E6
T: FTMS + p NSI Full ms [120.00-2000.00]

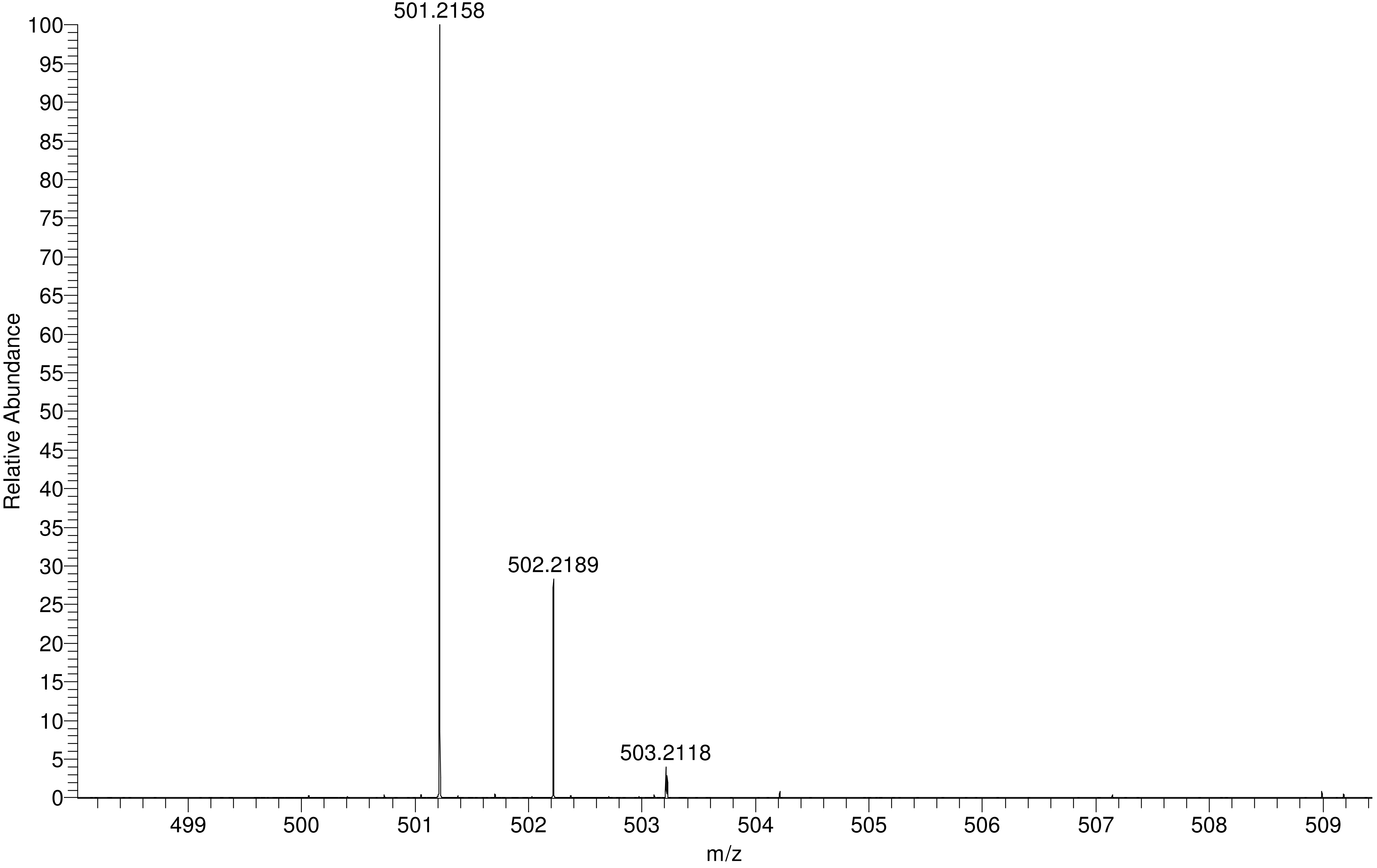

**Isotope:**	**Min. .. Max.**			
**12 C**	**0....60**			
**1 H**	**0....70**			
**14 N**	**0....15**			
**16 O**	**0....15**			
**23 Na**	**0....0**			
**32 S**	**0....3**			
**Tolerance Window:**	**+- 5.00 ppm**			
**Db/Ring Equiv:**	**-3.. 100**		**N-Rule:**	**Do not use**
**Fits:**	**100**		**Charge:**	**1**

**Mass**	**Theoretical Mass**	**Delta [ppm]**	**RDB**	**Composition**
501.2158	501.2158	0.0	-0.5	$C_{9} H_{33} O_{10} N_{12} S_{1}$
	501.2159	-0.3	21.0	$C_{31} H_{27} O_{2} N_{5}$
	501.2160	-0.3	8.0	$C_{16} H_{31} O_{2} N_{13} S_{2}$
	501.2160	-0.3	2.5	$C_{17} H_{37} O_{7} N_{6} S_{2}$
	501.2155	0.7	15.0	$C_{31} H_{35} O_{1} N_{1} S_{2}$
	501.2161	-0.7	5.5	$C_{25} H_{41} O_{4} S_{3}$
	501.2153	1.0	-1.0	$C_{8} H_{35} O_{4} N_{15} S_{3}$
	501.2153	1.0	6.5	$C_{24} H_{37} O_{9} S_{1}$
	501.2153	1.0	12.0	$C_{23} H_{31} O_{4} N_{7} S_{1}$
	501.2164	-1.3	8.5	$C_{17} H_{29} O_{8} N_{10}$
	501.2164	-1.3	3.0	$C_{18} H_{35} O_{13} N_{3}$
	501.2151	1.4	3.5	$C_{16} H_{33} O_{12} N_{6}$
	501.2151	1.4	9.0	$C_{15} H_{27} O_{7} N_{13}$
	501.2166	-1.6	17.0	$C_{24} H_{27} N_{11} S_{1}$
	501.2166	-1.6	11.5	$C_{25} H_{33} O_{5} N_{4} S_{1}$
	501.2167	-1.7	-1.5	$C_{10} H_{37} O_{5} N_{12} S_{3}$
	501.2148	2.0	6.0	$C_{23} H_{39} O_{3} N_{3} S_{3}$
	501.2146	2.3	-2.5	$C_{16} H_{41} O_{11} N_{2} S_{2}$
	501.2146	2.3	3.0	$C_{15} H_{35} O_{6} N_{9} S_{2}$
	501.2146	2.4	16.0	$C_{30} H_{31} O_{6} N_{1}$
	501.2146	2.4	21.5	$C_{29} H_{25} O_{1} N_{8}$
	501.2171	-2.6	-1.0	$C_{11} H_{35} O_{11} N_{9} S_{1}$
	501.2144	2.7	0.0	$C_{7} H_{31} O_{9} N_{15} S_{1}$
	501.2173	-2.9	20.5	$C_{33} H_{29} O_{3} N_{2}$
	501.2173	-3.0	7.5	$C_{18} H_{33} O_{3} N_{10} S_{2}$
	501.2173	-3.0	2.0	$C_{19} H_{39} O_{8} N_{3} S_{2}$
	501.2141	3.4	15.5	$C_{29} H_{33} N_{4} S_{2}$
	501.2175	-3.4	10.5	$C_{26} H_{37} N_{4} S_{3}$
	501.2139	3.7	7.0	$C_{22} H_{35} O_{8} N_{3} S_{1}$
	501.2139	3.7	12.5	$C_{21} H_{29} O_{3} N_{10} S_{1}$
	501.2178	-3.9	13.5	$C_{18} H_{25} O_{4} N_{14}$

aet7-96 MW=542?
(DCM)/MeOH + NH4OAc

Compound 33

EPSRC National Centre Swansea
LTQ Orbitrap XL

adam throup
21/06/2012 11:33:42

BRASHE218-OJ-HNESP #34-46 RT: 0.86-1.21 AV: 13 SM: 7G NL: 3.26E6
T: FTMS + p NSI Full ms [120.00-2000.00]

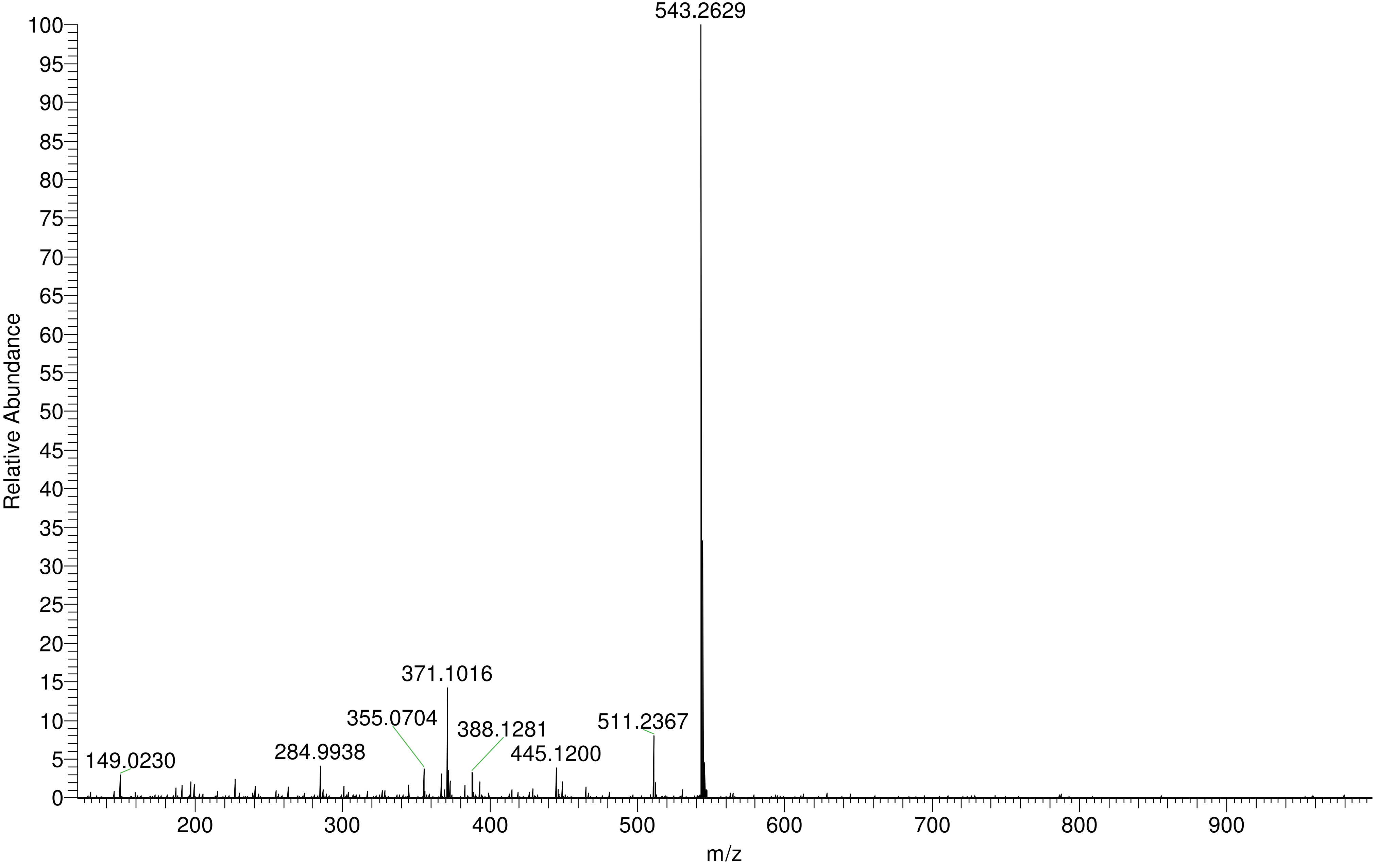

aet7-96 MW=542?
(DCM)/MeOH + NH4OAc

EPSRC National Centre Swansea
LTQ Orbitrap XL

adam throup
21/06/2012 11:33:42

BRASHE218-OJ-HNESP #34-46 RT: 0.86-1.21 AV: 13 SM: 7G NL: 3.26E6
T: FTMS + p NSI Full ms [120.00-2000.00]

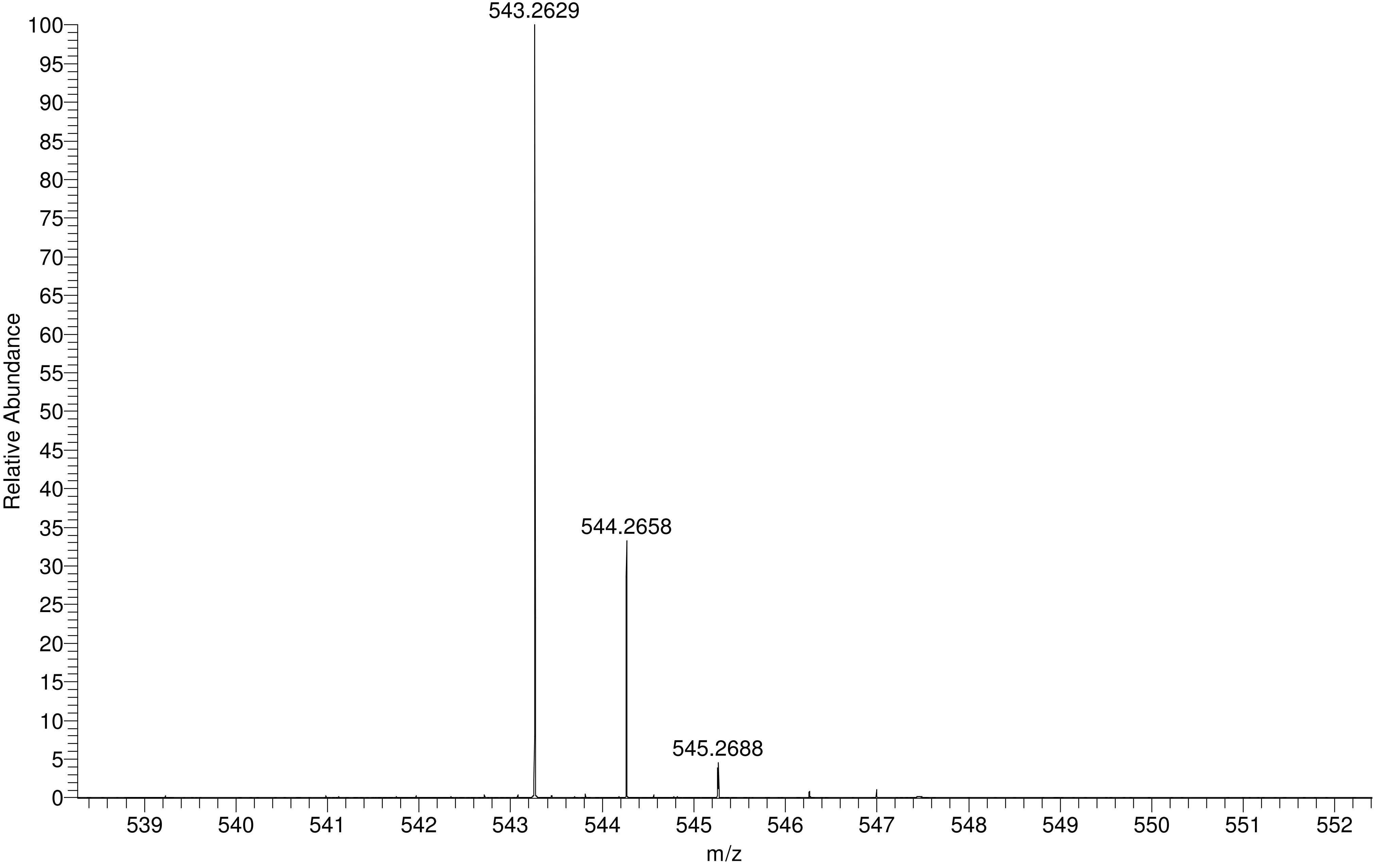

Isotope:	Min. .. Max.		
12 C	0....60		
1 H	0....70		
14 N	0....15		
16 O	0....15		
23 Na	0....0		
32 S	0....3		
Tolerance Window:	+- 5.00 ppm		
Db/Ring Equiv:	-3.. 100	N-Rule:	Do not use
Fits:	100	Charge:	1

Mass	Theoretical Mass	Delta [ppm]	RDB	Composition
543.2629	543.2629	-0.0	8.0	$C_{19} H_{37} O_{2} N_{13} S_{2}$
	543.2629	-0.0	2.5	$C_{20} H_{43} O_{7} N_{6} S_{2}$
	543.2629	0.0	21.0	$C_{34} H_{33} O_{2} N_{5}$
	543.2627	0.3	-0.5	$C_{12} H_{39} O_{10} N_{12} S_{1}$
	543.2631	-0.4	5.5	$C_{28} H_{47} O_{4} S_{3}$
	543.2634	-0.9	8.5	$C_{20} H_{35} O_{8} N_{10}$
	543.2634	-0.9	3.0	$C_{21} H_{41} O_{13} N_{3}$
	543.2624	0.9	15.0	$C_{34} H_{41} O_{1} N_{1} S_{2}$
	543.2623	1.2	-1.0	$C_{11} H_{41} O_{4} N_{15} S_{3}$
	543.2636	-1.2	17.0	$C_{27} H_{33} N_{11} S_{1}$
	543.2636	-1.2	11.5	$C_{28} H_{39} O_{5} N_{4} S_{1}$
	543.2622	1.2	6.5	$C_{27} H_{43} O_{9} S_{1}$
	543.2622	1.2	12.0	$C_{26} H_{37} O_{4} N_{7} S_{1}$
	543.2636	-1.3	-1.5	$C_{13} H_{43} O_{5} N_{12} S_{3}$
	543.2620	1.6	3.5	$C_{19} H_{39} O_{12} N_{6}$
	543.2620	1.6	9.0	$C_{18} H_{33} O_{7} N_{13}$
	543.2618	2.1	6.0	$C_{26} H_{45} O_{3} N_{3} S_{3}$
	543.2641	-2.2	-1.0	$C_{14} H_{41} O_{11} N_{9} S_{1}$
	543.2642	-2.4	20.5	$C_{36} H_{35} O_{3} N_{2}$
	543.2616	2.4	-2.5	$C_{19} H_{47} O_{11} N_{2} S_{2}$
	543.2616	2.4	3.0	$C_{18} H_{41} O_{6} N_{9} S_{2}$
	543.2643	-2.5	7.5	$C_{21} H_{39} O_{3} N_{10} S_{2}$
	543.2643	-2.5	2.0	$C_{22} H_{45} O_{8} N_{3} S_{2}$
	543.2615	2.5	16.0	$C_{33} H_{37} O_{6} N_{1}$
	543.2615	2.5	21.5	$C_{32} H_{31} O_{1} N_{8}$
	543.2614	2.8	0.0	$C_{10} H_{37} O_{9} N_{15} S_{1}$
	543.2644	-2.8	10.5	$C_{29} H_{43} N_{4} S_{3}$
	543.2647	-3.4	13.5	$C_{21} H_{31} O_{4} N_{14}$
	543.2647	-3.4	8.0	$C_{22} H_{37} O_{9} N_{7}$
	543.2647	-3.4	2.5	$C_{23} H_{43} O_{14}$
	543.2611	3.4	15.5	$C_{32} H_{39} N_{4} S_{2}$

Mass	Theoretical Mass	Delta [ppm]	RDB	Composition
	543.2649	-3.7	16.5	$C_{29} H_{35} O_{1} N_{8} S_{1}$
	543.2649	-3.7	11.0	$C_{30} H_{41} O_{6} N_{1} S_{1}$
	543.2609	3.7	7.0	$C_{25} H_{41} O_{8} N_{3} S_{1}$
	543.2609	3.7	12.5	$C_{24} H_{35} O_{3} N_{10} S_{1}$
	543.2649	-3.8	-2.0	$C_{15} H_{45} O_{6} N_{9} S_{3}$
	543.2607	4.0	4.0	$C_{17} H_{37} O_{11} N_{9}$
	543.2604	4.6	6.5	$C_{24} H_{43} O_{2} N_{6} S_{3}$
	543.2654	-4.6	4.0	$C_{15} H_{37} O_{7} N_{13} S_{1}$
	543.2654	-4.6	-1.5	$C_{16} H_{43} O_{12} N_{6} S_{1}$
	543.2602	4.9	-2.0	$C_{17} H_{45} O_{10} N_{5} S_{2}$
	543.2602	4.9	3.5	$C_{16} H_{39} O_{5} N_{12} S_{2}$
	543.2656	-5.0	7.0	$C_{23} H_{41} O_{4} N_{7} S_{2}$
	543.2656	-5.0	1.5	$C_{24} H_{47} O_{9} S_{2}$
	543.2602	5.0	16.5	$C_{31} H_{35} O_{5} N_{4}$
	543.2602	5.0	22.0	$C_{30} H_{29} N_{11}$

aet9-1-2 MW=468?
(DCM)/MeOH + NH4OAc

Compound 34

EPSRC National Centre Swansea
LTQ Orbitrap XL

adam throup
01/08/2012 10:21:47

BRASHE223-OJ-HNESP #31-49 RT: 0.75-1.28 AV: 19 SM: 7G NL: 3.30E6
T: FTMS + p NSI Full ms [120.00-2000.00]

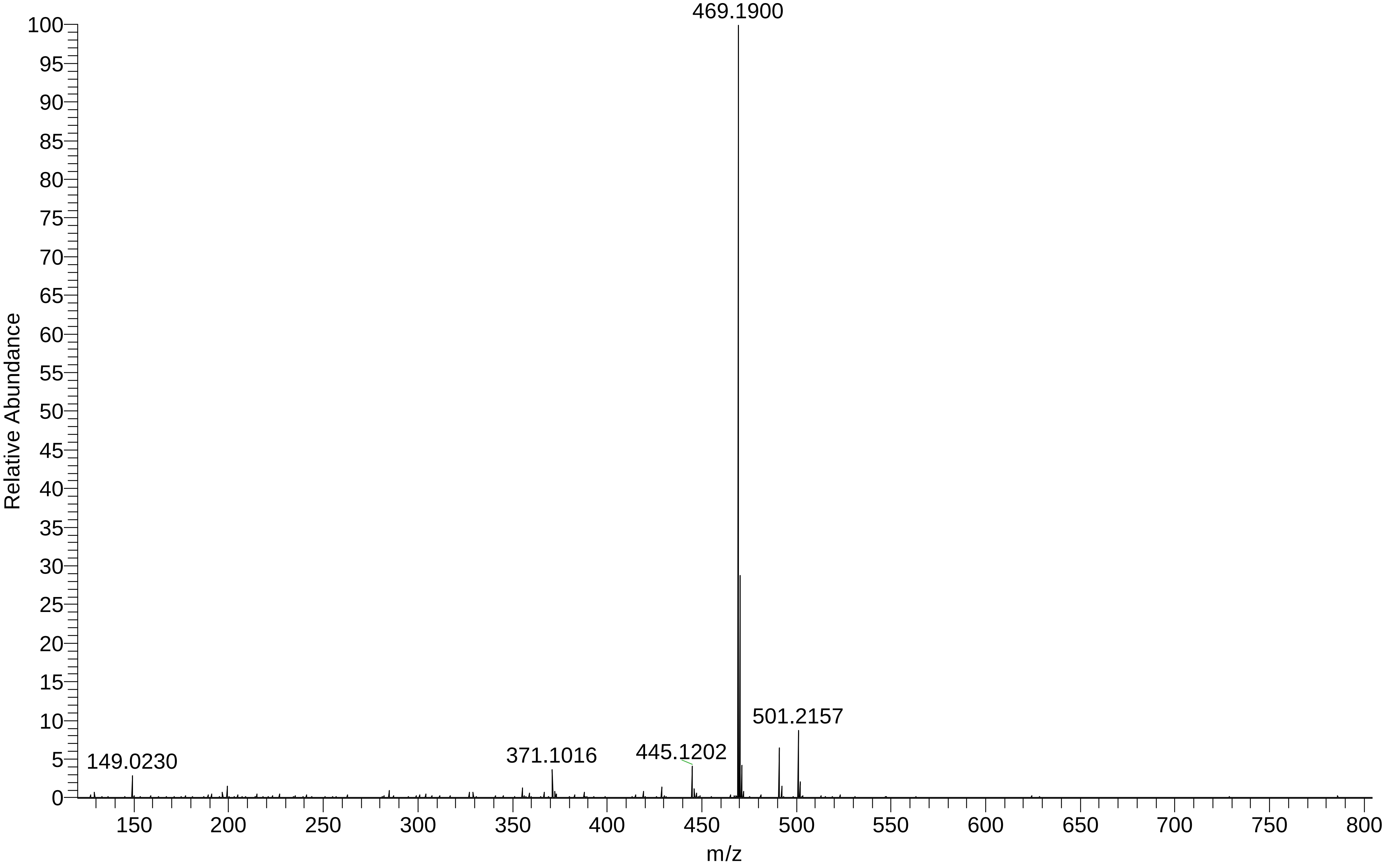

aet9-1-2 MW=468?
(DCM)/MeOH + NH4OAc

EPSRC National Centre Swansea
LTQ Orbitrap XL

adam throup
01/08/2012 10:21:47

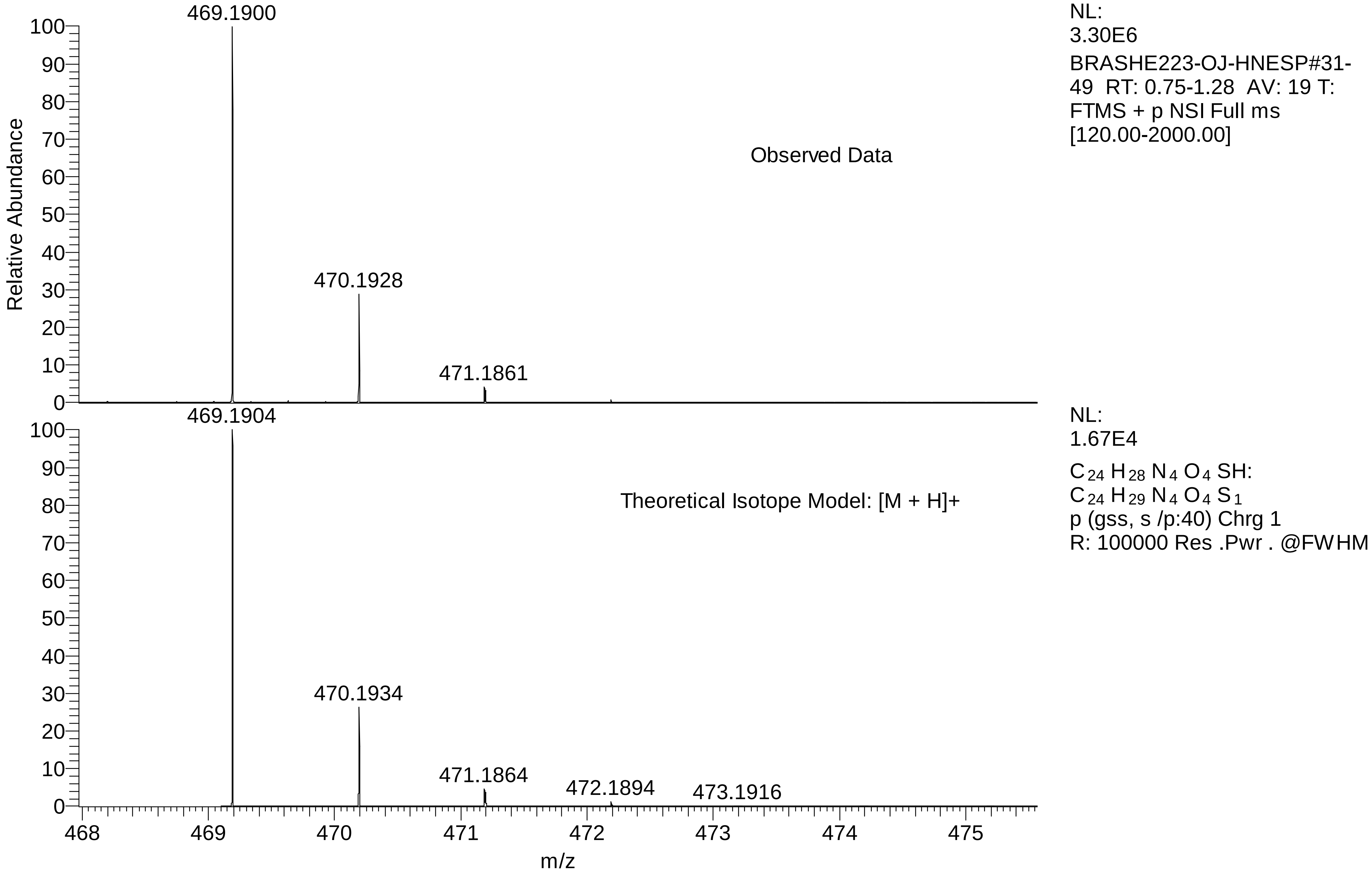

Isotope: **Min. .. Max.**
14 N **0....14**
16 O **0....14**
12 C **0....60**
1 H **0....70**
23 Na **0....0**
32 S **0....4**
Tolerance Window: **+- 5.00 ppm**
Db/Ring Equiv: -3.. 100 **N-Rule: Do not use**
Fits: 100 **Charge: 1**

**Mass**	**Theoretical Mass**	**Delta [ppm]**	**RDB**	**Composition**
469.1900	469.1899	0.1	6.5	$C_{24} H_{37} O_3 S_3$
	469.1902	-0.5	9.5	$C_{16} H_{25} O_7 N_{10}$
	469.1902	-0.5	4.0	$C_{17} H_{31} O_{12} N_3$
	469.1898	0.5	3.5	$C_{16} H_{33} O_6 N_6 S_2$
	469.1897	0.5	9.0	$C_{15} H_{27} O_1 N_{13} S_2$
	469.1897	0.6	22.0	$C_{30} H_{23} O_1 N_5$
	469.1904	-0.9	12.5	$C_{24} H_{29} O_4 N_4 S_1$
	469.1896	0.9	0.5	$C_8 H_{29} O_9 N_{12} S_1$
	469.1904	-0.9	-0.5	$C_9 H_{33} O_4 N_{12} S_3$
	469.1906	-1.3	2.5	$C_{17} H_{37} O_1 N_6 S_4$
	469.1893	1.5	-2.5	$C_{16} H_{41} O_5 N_2 S_4$
	469.1893	1.5	3.0	$C_{15} H_{35} N_9 S_4$
	469.1892	1.6	16.0	$C_{30} H_{31} N_1 S_2$
	469.1909	-1.9	0.0	$C_{10} H_{31} O_{10} N_9 S_1$
	469.1891	2.0	7.5	$C_{23} H_{33} O_8 S_1$
	469.1891	2.0	13.0	$C_{22} H_{27} O_3 N_7 S_1$
	469.1911	-2.2	21.5	$C_{32} H_{25} O_2 N_2$
	469.1911	-2.3	8.5	$C_{17} H_{29} O_2 N_{10} S_2$
	469.1911	-2.3	3.0	$C_{18} H_{35} O_7 N_3 S_2$
	469.1889	2.4	4.5	$C_{15} H_{29} O_{11} N_6$
	469.1889	2.4	10.0	$C_{14} H_{23} O_6 N_{13}$
	469.1886	3.0	7.0	$C_{22} H_{35} O_2 N_3 S_3$
	469.1916	-3.3	14.5	$C_{17} H_{21} O_3 N_{14}$
	469.1916	-3.3	9.0	$C_{18} H_{27} O_8 N_7$
	469.1916	-3.3	3.5	$C_{19} H_{33} O_{13}$
	469.1884	3.4	-1.5	$C_{15} H_{37} O_{10} N_2 S_2$
	469.1884	3.4	4.0	$C_{14} H_{31} O_5 N_9 S_2$
	469.1884	3.5	17.0	$C_{29} H_{27} O_5 N_1$
	469.1884	3.5	22.5	$C_{28} H_{21} N_8$
	469.1917	-3.7	17.5	$C_{25} H_{25} N_8 S_1$
	469.1917	-3.7	12.0	$C_{26} H_{31} O_5 N_1 S_1$

aet9-2-2 MW=510?
(DCM)/MeOH + NH4OAc

Compound 35

EPSRC National Centre Swansea
LTQ Orbitrap XL

adam throup
01/08/2012 10:25:14

BRASHE224-OJ-HNESP #29-50 RT: 0.71-1.29 AV: 21 SM: 7G NL: 4.81E5
T: FTMS + p NSI Full ms [120.00-2000.00]

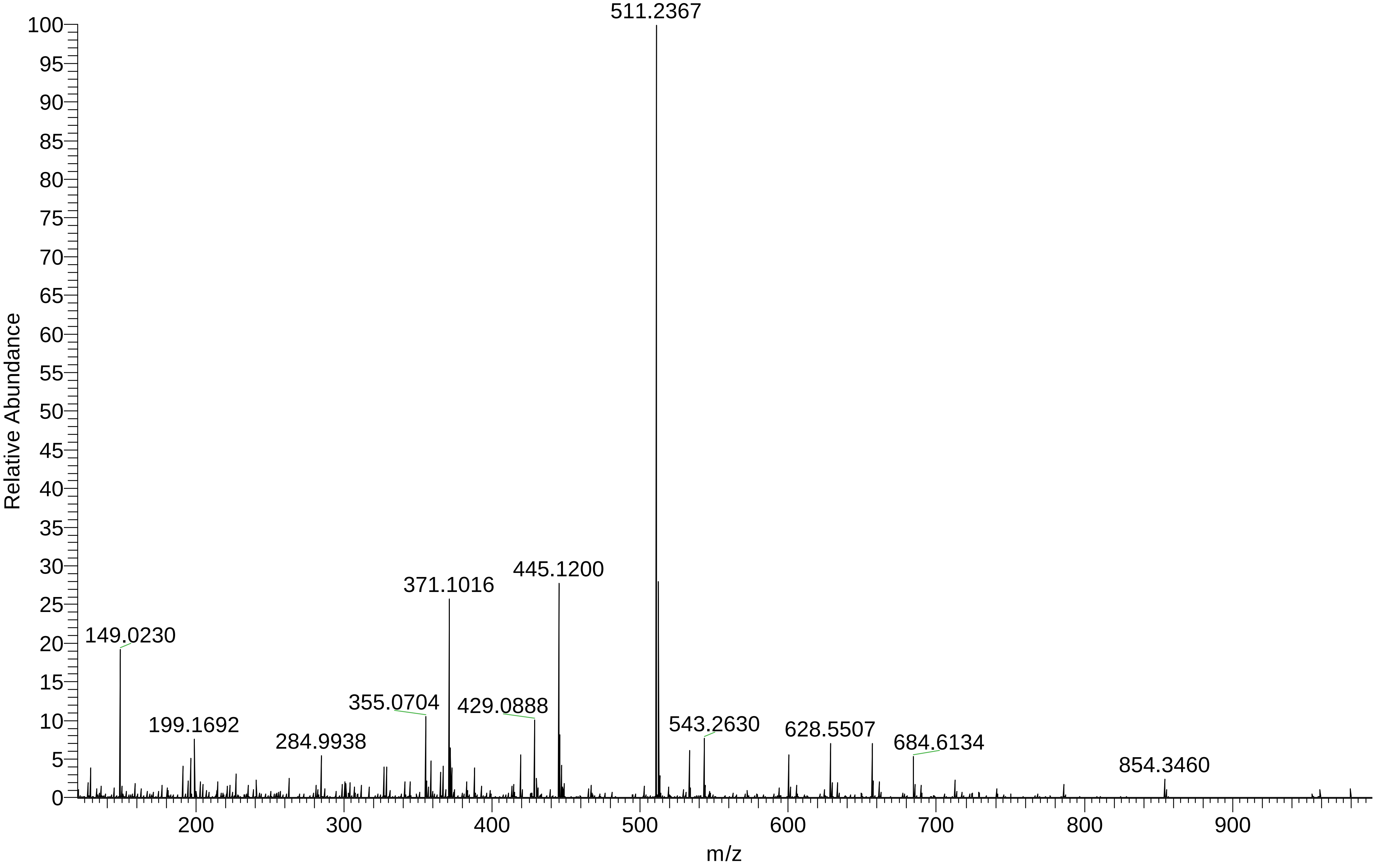

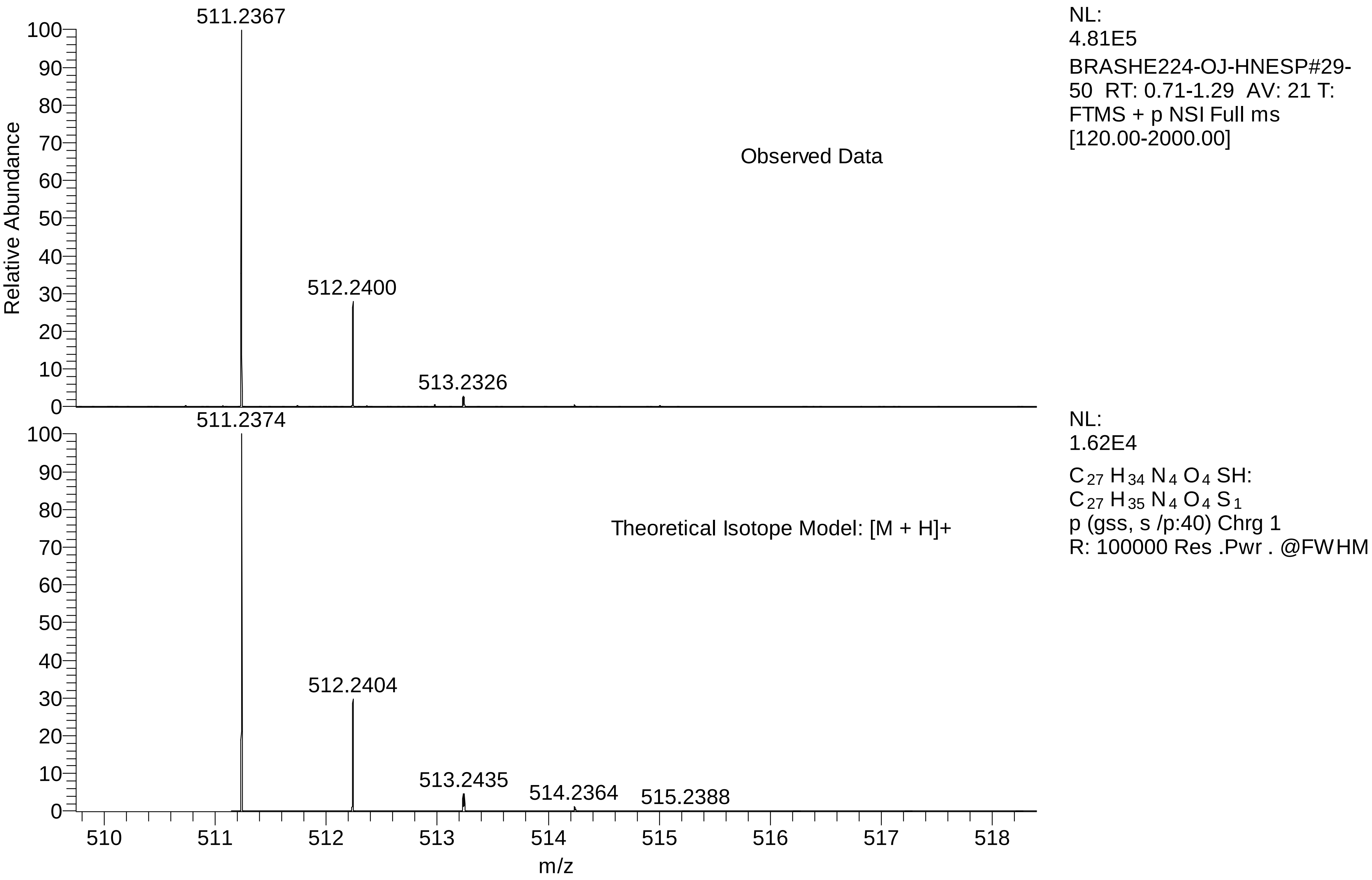
511.2367
512.2400
513.2326
Observed Data
NL:
4.81E5
BRASHE224-OJ-HNESP#29-50 RT: 0.71-1.29 AV: 21 T: FTMS + p NSI Full ms [120.00-2000.00]
511.2374
512.2404
513.2435
514.2364
515.2388
Theoretical Isotope Model: [M + H]+
NL:
1.62E4
C 27 H 34 N 4 O 4 SH:
C 27 H 35 N 4 O 4 S 1
p (gss, s /p:40) Chrg 1
R: 100000 Res .Pwr . @FWHM
Relative Abundance
m/z
510
511
512
513
514
515
516
517
518

Isotope: **Min. .. Max.**
14 N **0....14**
16 O **0....14**
12 C **0....60**
1 H **0....75**
23 Na **0....0**
32 S **0....4**
Tolerance Window: **+- 5.00 ppm**
Db/Ring Equiv: -3.. 100 **N-Rule: Do not use**
Fits: 100 **Charge: 1**

**Mass**	**Theoretical Mass**	**Delta [ppm]**	**RDB**	**Composition**
511.2367	511.2367	-0.0	3.5	$C_{19} H_{39} O_{6} N_{6} S_{2}$
	511.2367	0.0	9.0	$C_{18} H_{33} O_{1} N_{13} S_{2}$
	511.2367	0.1	22.0	$C_{33} H_{29} O_{1} N_{5}$
	511.2365	0.4	0.5	$C_{11} H_{35} O_{9} N_{12} S_{1}$
	511.2369	-0.4	6.5	$C_{27} H_{43} O_{3} S_{3}$
	511.2362	0.9	-2.5	$C_{19} H_{47} O_{5} N_{2} S_{4}$
	511.2372	-0.9	9.5	$C_{19} H_{31} O_{7} N_{10}$
	511.2362	0.9	3.0	$C_{18} H_{41} N_{9} S_{4}$
	511.2372	-0.9	4.0	$C_{20} H_{37} O_{12} N_{3}$
	511.2362	1.0	16.0	$C_{33} H_{37} N_{1} S_{2}$
	511.2374	-1.3	12.5	$C_{27} H_{35} O_{4} N_{4} S_{1}$
	511.2360	1.3	7.5	$C_{26} H_{39} O_{8} S_{1}$
	511.2374	-1.3	-0.5	$C_{12} H_{39} O_{4} N_{12} S_{3}$
	511.2360	1.3	13.0	$C_{25} H_{33} O_{3} N_{7} S_{1}$
	511.2358	1.7	4.5	$C_{18} H_{35} O_{11} N_{6}$
	511.2376	-1.7	2.5	$C_{20} H_{43} O_{1} N_{6} S_{4}$
	511.2358	1.7	10.0	$C_{17} H_{29} O_{6} N_{13}$
	511.2355	2.3	7.0	$C_{25} H_{41} O_{2} N_{3} S_{3}$
	511.2379	-2.3	0.0	$C_{13} H_{37} O_{10} N_{9} S_{1}$
	511.2380	-2.6	21.5	$C_{35} H_{31} O_{2} N_{2}$
	511.2354	2.6	-1.5	$C_{18} H_{43} O_{10} N_{2} S_{2}$
	511.2380	-2.6	8.5	$C_{20} H_{35} O_{2} N_{10} S_{2}$
	511.2354	2.6	4.0	$C_{17} H_{37} O_{5} N_{9} S_{2}$
	511.2380	-2.6	3.0	$C_{21} H_{41} O_{7} N_{3} S_{2}$
	511.2353	2.7	17.0	$C_{32} H_{33} O_{5} N_{1}$
	511.2353	2.7	22.5	$C_{31} H_{27} N_{8}$
	511.2385	-3.5	14.5	$C_{20} H_{27} O_{3} N_{14}$
	511.2349	3.5	-2.0	$C_{17} H_{45} O_{4} N_{5} S_{4}$
	511.2385	-3.5	9.0	$C_{21} H_{33} O_{8} N_{7}$
	511.2385	-3.6	3.5	$C_{22} H_{39} O_{13}$
	511.2387	-3.9	17.5	$C_{28} H_{31} N_{8} S_{1}$

AET9-6 MW=329?
ASAP(SOLID + NH4OAc)

Compound 36

EPSRC National Centre Swansea
LTQ Orbitrap XL

Throup
06/11/2012 12:06:57

BRASHE233-PG-HASP #135-137 RT: 3.87-3.92 AV: 3 SM: 7G NL: 2.04E7
T: FTMS + p APCI corona Full ms [100.00-800.00]

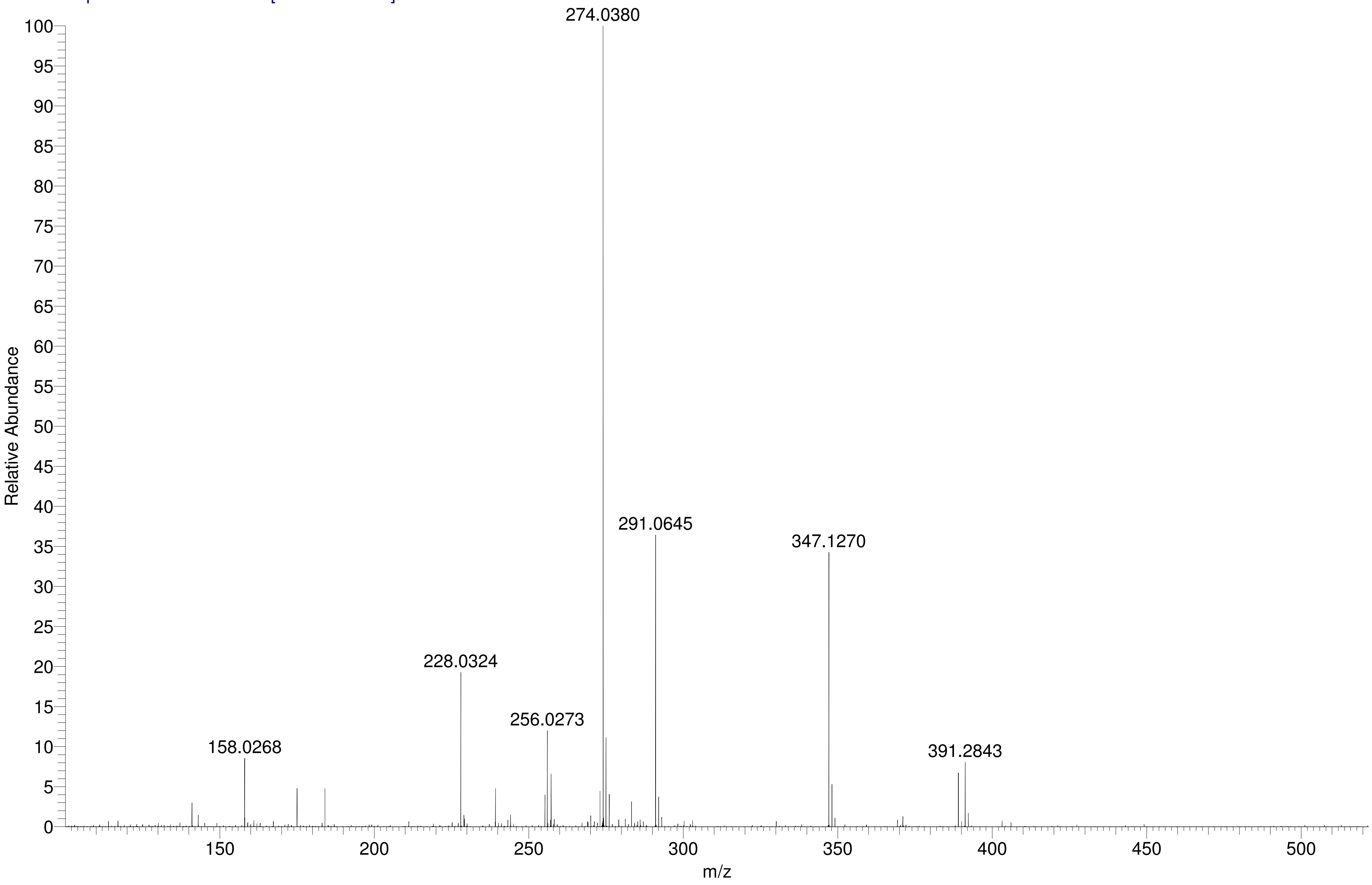

AET9-6 MW=329?
ASAP(SOLID + NH4OAc)

EPSRC National Centre Swansea
LTQ Orbitrap XL

Throup
06/11/2012 12:06:57

BRASHE233-PG-HASP #135-137 RT: 3.87-3.92 AV: 3 SM: 7G NL: 2.04E7
T: FTMS + p APCI corona Full ms [100.00-800.00]

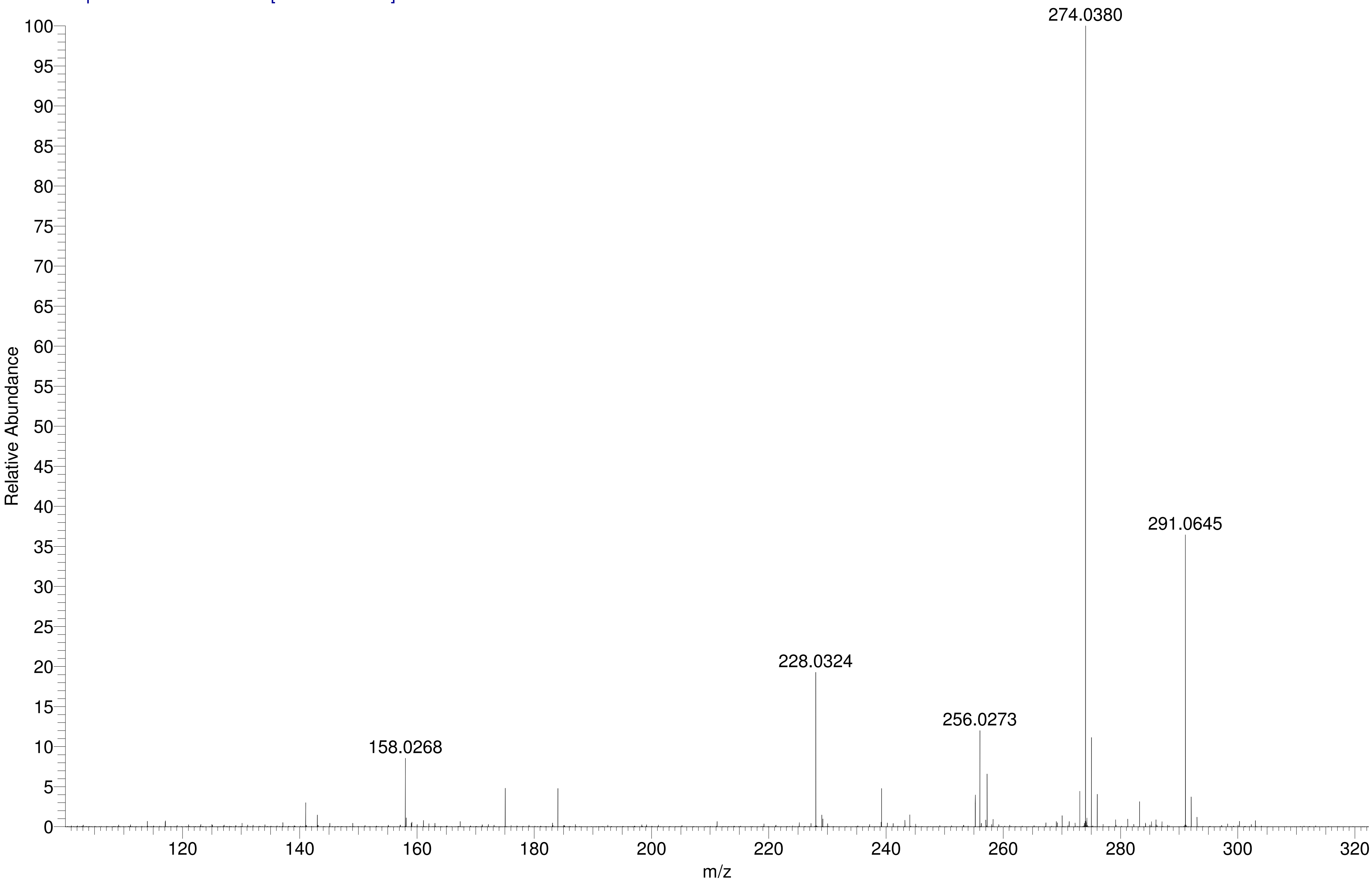

AET9-6 MW=329?
ASAP(SOLID + NH4OAc)

EPSRC National Centre Swansea
LTQ Orbitrap XL

Throup
06/11/2012 12:06:57

BRASHE233-PG-HASP #135-137 RT: 3.87-3.92 AV: 3 SM: 7G NL: 7.44E6
T: FTMS + p APCI corona Full ms [100.00-800.00]

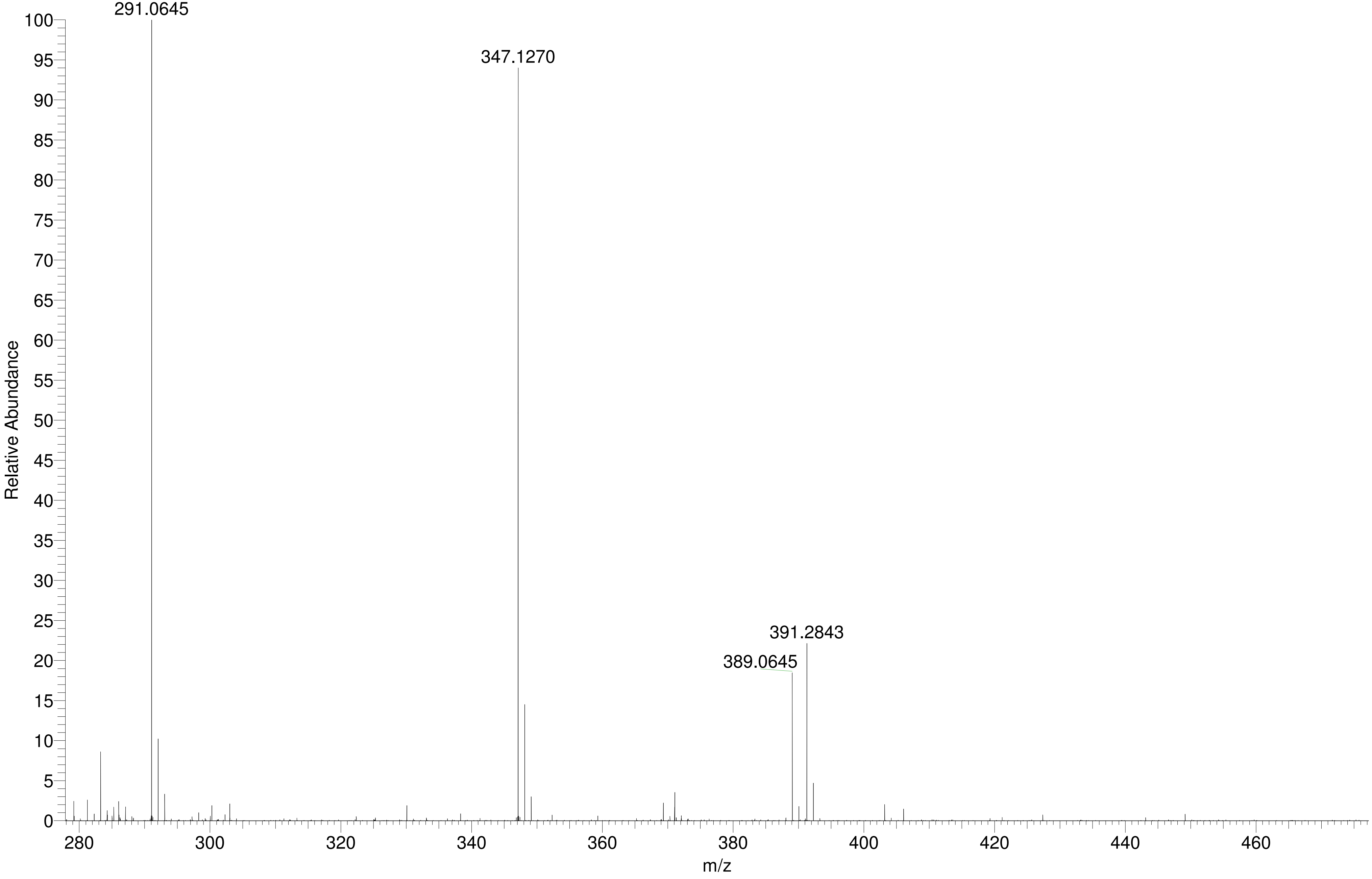

AET9-6 MW=329?
ASAP(SOLID + NH4OAc)

EPSRC National Centre Swansea
LTQ Orbitrap XL

Throup
06/11/2012 12:06:57

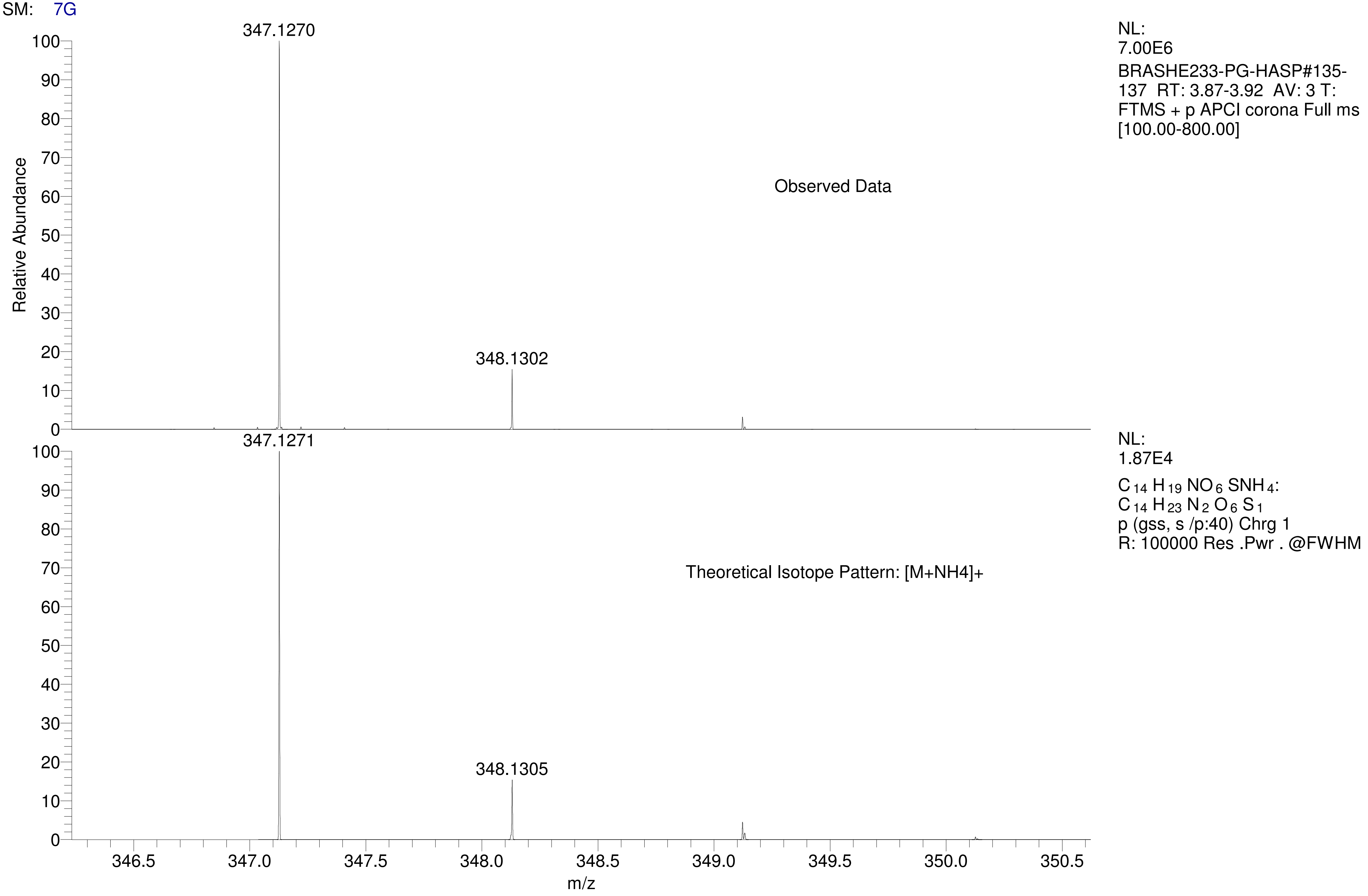

Isotope:	Min. .. Max.		
14 N	0....16		
16 O	0....15		
12 C	0....80		
1 H	0....100		
23 Na	0....0		
32 S	0....3		
Tolerance Window:	+- 5.00 ppm		
Db/Ring Equiv:	-3.. 100	N-Rule:	Do not use
Fits:	100	Charge:	1

Mass	Theoretical Mass	Delta [ppm]	RDB	Composition
347.1270	347.1270	0.1	1.5	$C_{6} H_{19} O_{9} N_{8}$
	347.1269	0.2	7.0	$C_{5} H_{13} O_{4} N_{15}$
	347.1271	-0.4	10.0	$C_{13} H_{17} O_{1} N_{9} S_{1}$
	347.1271	-0.4	4.5	$C_{14} H_{23} O_{6} N_{2} S_{1}$
	347.1267	1.0	4.0	$C_{13} H_{25} N_{5} S_{3}$
	347.1265	1.5	1.0	$C_{5} H_{21} O_{3} N_{11} S_{2}$
	347.1264	1.6	14.0	$C_{20} H_{17} O_{3} N_{3}$
	347.1278	-2.3	13.5	$C_{22} H_{19} O_{4}$
	347.1278	-2.4	0.5	$C_{7} H_{23} O_{4} N_{8} S_{2}$
	347.1280	-2.9	3.5	$C_{15} H_{27} O_{1} N_{2} S_{3}$
	347.1258	3.5	5.0	$C_{12} H_{21} O_{5} N_{5} S_{1}$
	347.1258	3.5	10.5	$C_{11} H_{15} N_{12} S_{1}$
	347.1283	-3.7	6.5	$C_{7} H_{15} O_{5} N_{12}$
	347.1283	-3.7	1.0	$C_{8} H_{21} O_{10} N_{5}$
	347.1256	4.0	2.0	$C_{4} H_{17} O_{8} N_{11}$
	347.1285	-4.2	9.5	$C_{15} H_{19} O_{2} N_{6} S_{1}$
	347.1253	4.8	-1.0	$C_{12} H_{29} O_{4} N_{1} S_{3}$

Compound 37

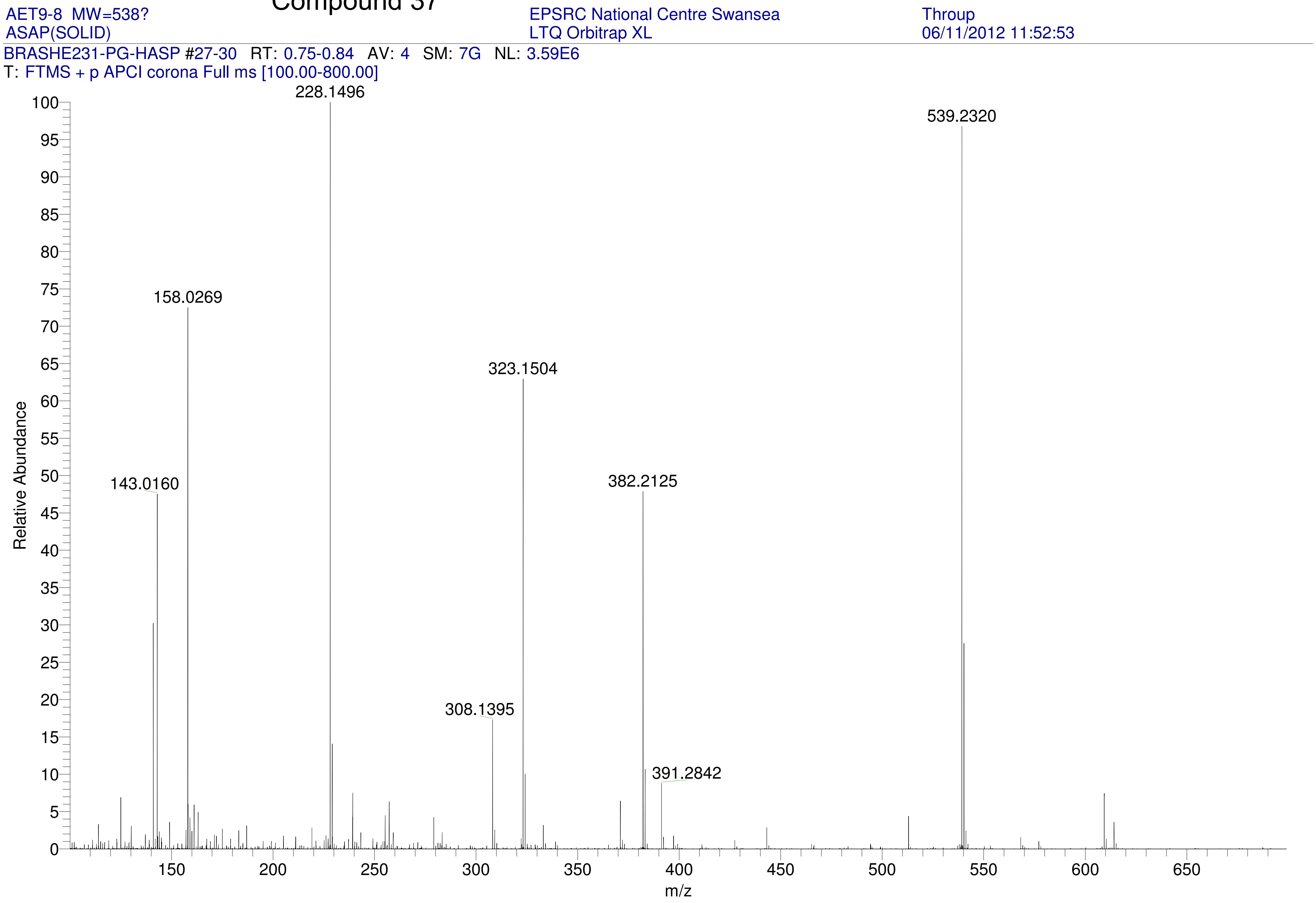
AET9-8 MW=538?
ASAP(SOLID)
EPSRC National Centre Swansea
LTQ Orbitrap XL
Throup
06/11/2012 11:52:53
BRASHE231-PG-HASP #27-30 RT: 0.75-0.84 AV: 4 SM: 7G NL: 3.59E6
T: FTMS + p APCI corona Full ms [100.00-800.00]
228.1496
539.2320
158.0269
323.1504
143.0160
382.2125
308.1395
391.2842
Relative Abundance
m/z

AET9-8 MW=538?
ASAP(SOLID)

EPSRC National Centre Swansea
LTQ Orbitrap XL

Throup
06/11/2012 11:52:53

BRASHE231-PG-HASP #27-30 RT: 0.75-0.84 AV: 4 SM: 7G NL: 3.59E6
T: FTMS + p APCI corona Full ms [100.00-800.00]

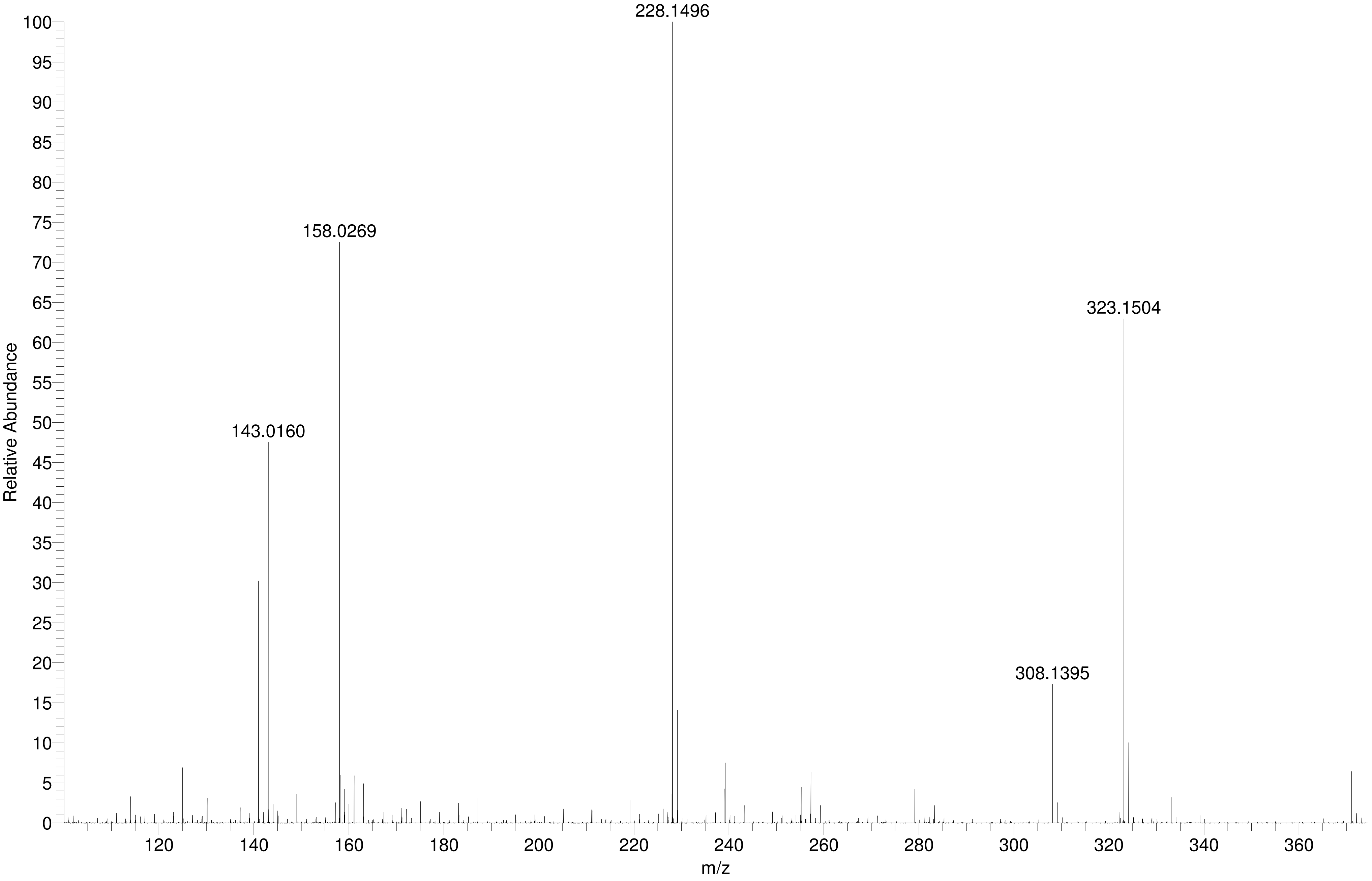

AET9-8 MW=538?
ASAP(SOLID)

EPSRC National Centre Swansea
LTQ Orbitrap XL

Throup
06/11/2012 11:52:53

BRASHE231-PG-HASP #27-30 RT: 0.75-0.84 AV: 4 SM: 7G NL: 3.47E6
T: FTMS + p APCI corona Full ms [100.00-800.00]

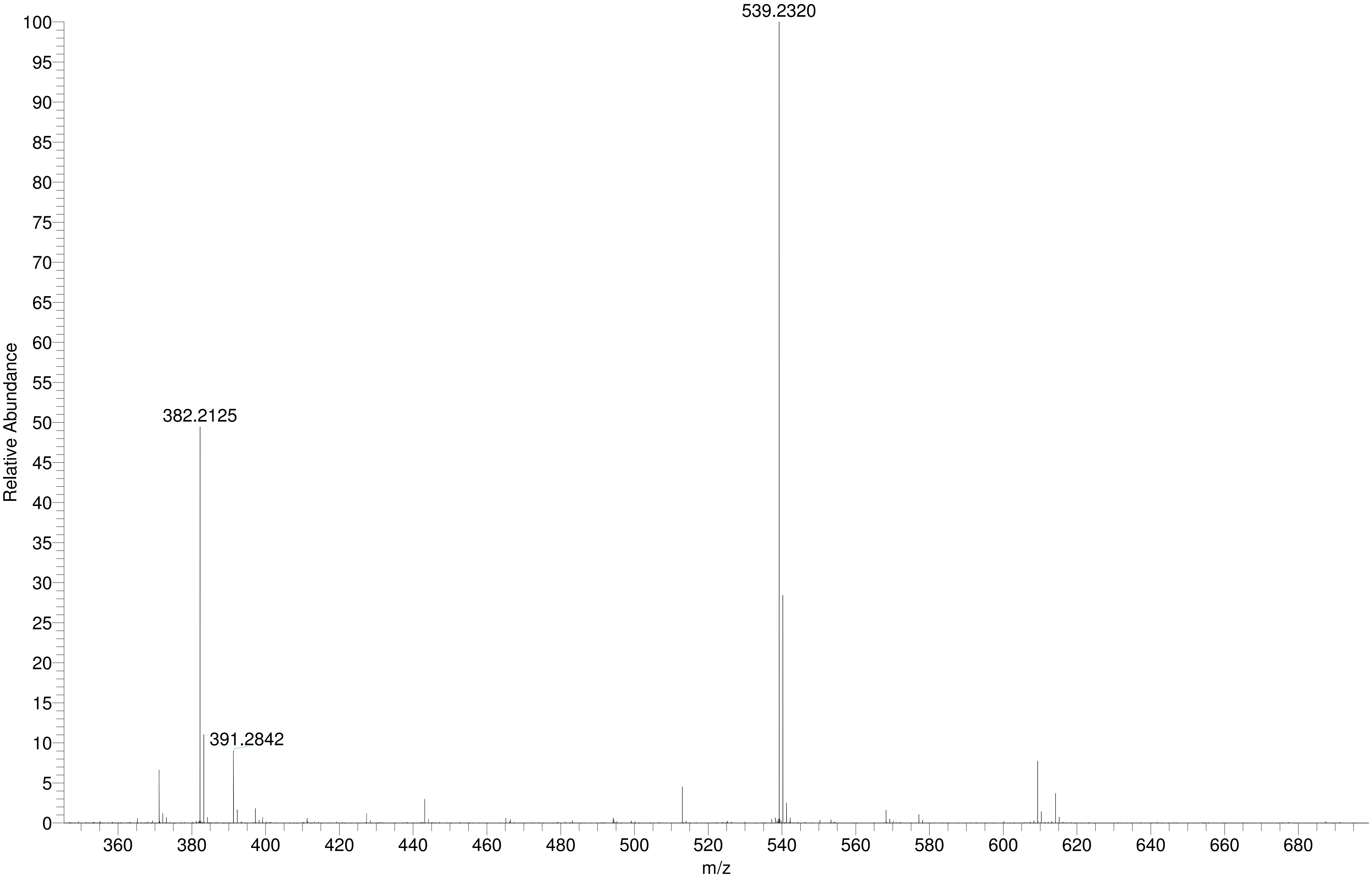

AET9-8 MW=538?
ASAP(SOLID)

EPSRC National Centre Swansea
LTQ Orbitrap XL

Throup
06/11/2012 11:52:53

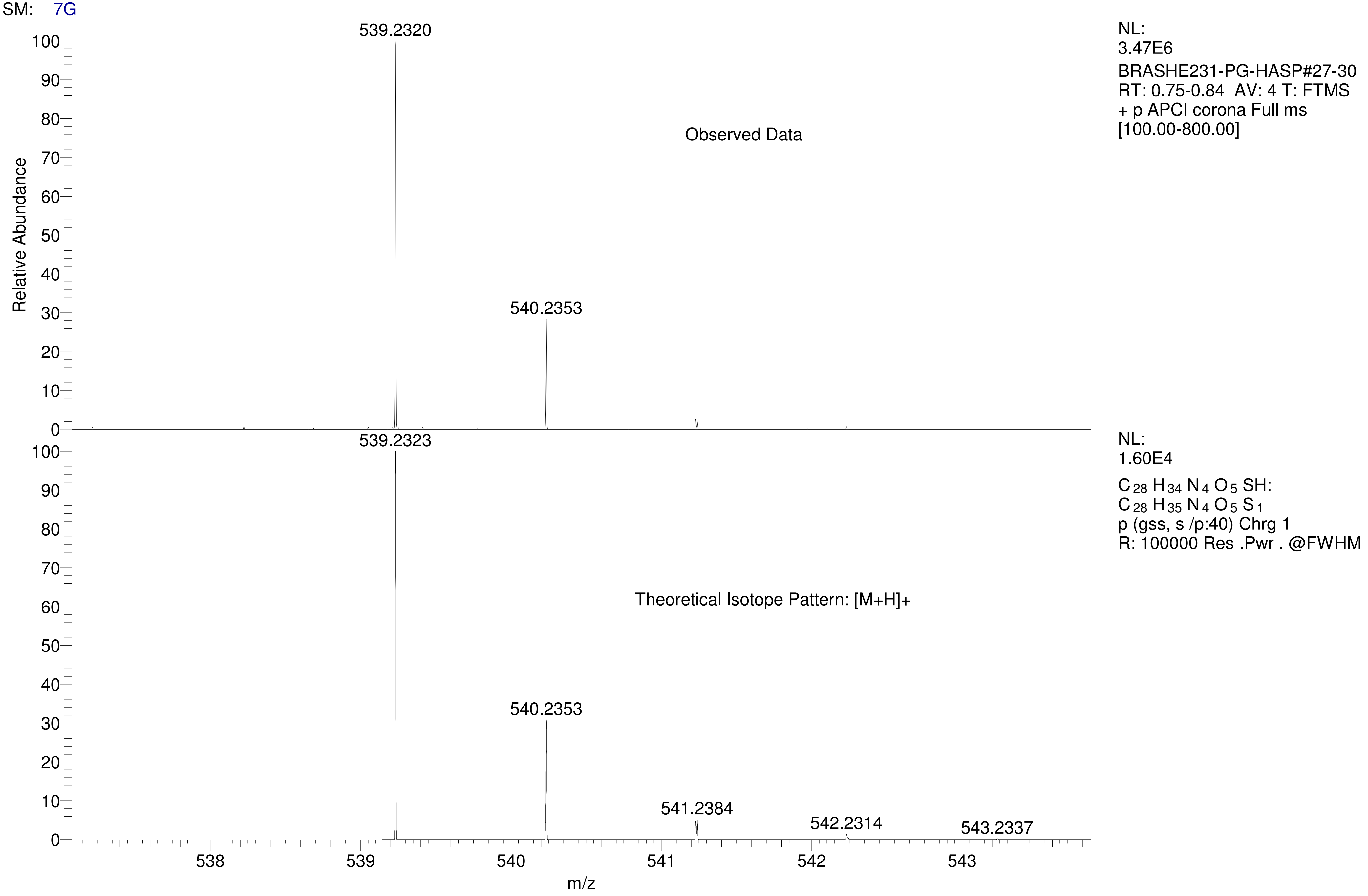

Isotope:	Min. .. Max.		
14 N	0....16		
16 O	0....15		
12 C	0....80		
1 H	0....100		
23 Na	0....0		
32 S	0....3		
Tolerance Window:	+- 5.00 ppm		
Db/Ring Equiv:	-3.. 100	N-Rule:	Do not use
Fits:	100	Charge:	1

Mass	Theoretical Mass	Delta [ppm]	RDB	Composition
539.2320	539.2321	-0.2	10.5	$C_{20} H_{31} O_{8} N_{10}$
	539.2321	-0.2	5.0	$C_{21} H_{37} O_{13} N_{3}$
	539.2318	0.4	7.5	$C_{28} H_{43} O_{4} S_{3}$
	539.2323	-0.5	19.0	$C_{27} H_{29} N_{11} S_{1}$
	539.2323	-0.5	13.5	$C_{28} H_{35} O_{5} N_{4} S_{1}$
	539.2323	-0.6	0.5	$C_{13} H_{39} O_{5} N_{12} S_{3}$
	539.2316	0.7	4.5	$C_{20} H_{39} O_{7} N_{6} S_{2}$
	539.2316	0.7	10.0	$C_{19} H_{33} O_{2} N_{13} S_{2}$
	539.2316	0.8	23.0	$C_{34} H_{29} O_{2} N_{5}$
	539.2314	1.1	1.5	$C_{12} H_{35} O_{10} N_{12} S_{1}$
	539.2326	-1.1	-2.0	$C_{6} H_{33} O_{14} N_{15}$
	539.2328	-1.4	6.5	$C_{13} H_{31} O_{6} N_{16} S_{1}$
	539.2328	-1.4	1.0	$C_{14} H_{37} O_{11} N_{9} S_{1}$
	539.2311	1.7	17.0	$C_{34} H_{37} O_{1} N_{1} S_{2}$
	539.2329	-1.7	22.5	$C_{36} H_{31} O_{3} N_{2}$
	539.2330	-1.8	9.5	$C_{21} H_{35} O_{3} N_{10} S_{2}$
	539.2330	-1.8	4.0	$C_{22} H_{41} O_{8} N_{3} S_{2}$
	539.2310	1.9	1.0	$C_{11} H_{37} O_{4} N_{15} S_{3}$
	539.2309	2.0	8.5	$C_{27} H_{39} O_{9} S_{1}$
	539.2309	2.0	14.0	$C_{26} H_{33} O_{4} N_{7} S_{1}$
	539.2331	-2.1	12.5	$C_{29} H_{39} N_{4} S_{3}$
	539.2307	2.3	5.5	$C_{19} H_{35} O_{12} N_{6}$
	539.2307	2.3	11.0	$C_{18} H_{29} O_{7} N_{13}$
	539.2334	-2.6	15.5	$C_{21} H_{27} O_{4} N_{14}$
	539.2334	-2.6	10.0	$C_{22} H_{33} O_{9} N_{7}$
	539.2334	-2.7	4.5	$C_{23} H_{39} O_{14}$
	539.2335	-2.7	-3.0	$C_{7} H_{37} O_{9} N_{15} S_{2}$
	539.2305	2.9	8.0	$C_{26} H_{41} O_{3} N_{3} S_{3}$
	539.2336	-3.0	18.5	$C_{29} H_{31} O_{1} N_{8} S_{1}$
	539.2336	-3.0	13.0	$C_{30} H_{37} O_{6} N_{1} S_{1}$
	539.2336	-3.0	5.5	$C_{14} H_{35} O_{1} N_{16} S_{3}$

Mass	Theoretical Mass	Delta [ppm]	RDB	Composition
	539.2336	-3.0	0.0	$C_{15} H_{41} O_{6} N_{9} S_{3}$
	539.2303	3.2	-0.5	$C_{19} H_{43} O_{11} N_{2} S_{2}$
	539.2303	3.2	5.0	$C_{18} H_{37} O_{6} N_{9} S_{2}$
	539.2303	3.2	10.5	$C_{17} H_{31} O_{1} N_{16} S_{2}$
	539.2302	3.3	18.0	$C_{33} H_{33} O_{6} N_{1}$
	539.2302	3.3	23.5	$C_{32} H_{27} O_{1} N_{8}$
	539.2301	3.5	2.0	$C_{10} H_{33} O_{9} N_{15} S_{1}$
	539.2339	-3.6	-2.5	$C_{8} H_{35} O_{15} N_{12}$
	539.2341	-3.9	6.0	$C_{15} H_{33} O_{7} N_{13} S_{1}$
	539.2341	-3.9	0.5	$C_{16} H_{39} O_{12} N_{6} S_{1}$
	539.2298	4.1	17.5	$C_{32} H_{35} N_{4} S_{2}$
	539.2343	-4.3	9.0	$C_{23} H_{37} O_{4} N_{7} S_{2}$
	539.2343	-4.3	3.5	$C_{24} H_{43} O_{9} S_{2}$
	539.2296	4.5	9.0	$C_{25} H_{37} O_{8} N_{3} S_{1}$
	539.2296	4.5	14.5	$C_{24} H_{31} O_{3} N_{10} S_{1}$
	539.2345	-4.6	12.0	$C_{31} H_{41} O_{1} N_{1} S_{3}$
	539.2294	4.8	6.0	$C_{17} H_{33} O_{11} N_{9}$
	539.2294	4.8	11.5	$C_{16} H_{27} O_{6} N_{16}$

AET9-12 MW=542?
(DCM)/MeOH + NH4OAc

Compound 39

EPSRC National Centre Swansea
LTQ Orbitrap XL

adam throup
06/11/2012 11:01:27

BRASHE230-OJ-HNESP #31-49 RT: 0.77-1.28 AV: 19 SM: 7G NL: 5.17E6
T: FTMS + p NSI Full ms [120.00-2000.00]

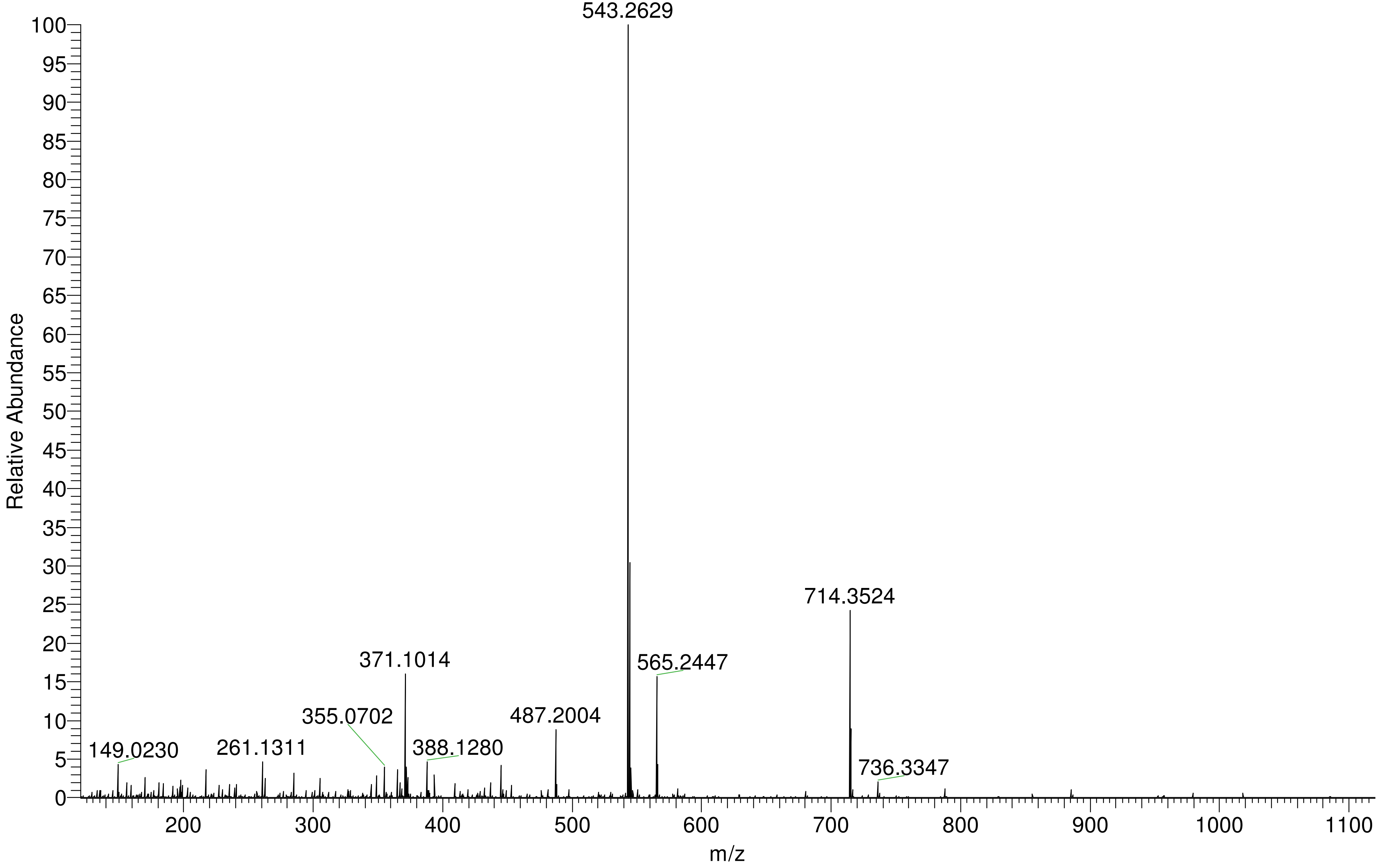

AET9-12 MW=542?
(DCM)/MeOH + NH4OAc

EPSRC National Centre Swansea
LTQ Orbitrap XL

adam throup
06/11/2012 11:01:27

BRASHE230-OJ-HNESP #31-49 RT: 0.77-1.28 AV: 19 SM: 7G NL: 5.17E6
T: FTMS + p NSI Full ms [120.00-2000.00]

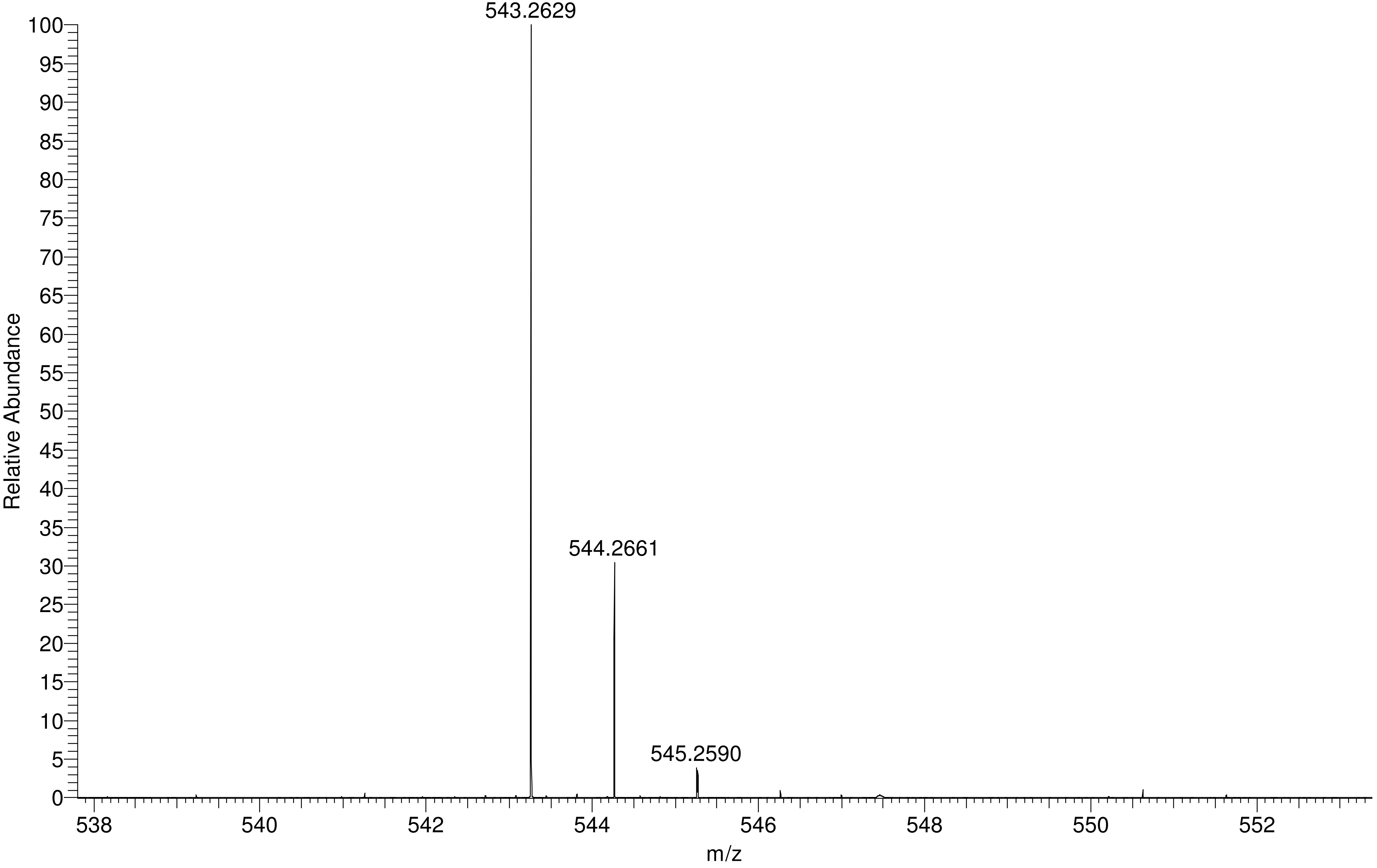

**Isotope:**	**Min. .. Max.**		
**12 C**	**0....60**		
**1 H**	**0....70**		
**14 N**	**0....15**		
**16 O**	**0....15**		
**23 Na**	**0....0**		
**32 S**	**0....3**		
**Tolerance Window:**	**+- 5.00 ppm**		
**Db/Ring Equiv:**	**-3.. 100**	**N-Rule:**	**Do not use**
**Fits:**	**100**	**Charge:**	**1**

**Mass**	**Theoretical Mass**	**Delta [ppm]**	**RDB**	**Composition**
543.2629	543.2629	-0.0	8.0	$C_{19} H_{37} O_{2} N_{13} S_{2}$
	543.2629	-0.0	2.5	$C_{20} H_{43} O_{7} N_{6} S_{2}$
	543.2629	0.0	21.0	$C_{34} H_{33} O_{2} N_{5}$
	543.2627	0.3	-0.5	$C_{12} H_{39} O_{10} N_{12} S_{1}$
	543.2631	-0.4	5.5	$C_{28} H_{47} O_{4} S_{3}$
	543.2634	-0.9	8.5	$C_{20} H_{35} O_{8} N_{10}$
	543.2634	-0.9	3.0	$C_{21} H_{41} O_{13} N_{3}$
	543.2624	0.9	15.0	$C_{34} H_{41} O_{1} N_{1} S_{2}$
	543.2623	1.2	-1.0	$C_{11} H_{41} O_{4} N_{15} S_{3}$
	543.2636	-1.2	17.0	$C_{27} H_{33} N_{11} S_{1}$
	543.2636	-1.2	11.5	$C_{28} H_{39} O_{5} N_{4} S_{1}$
	543.2622	1.2	6.5	$C_{27} H_{43} O_{9} S_{1}$
	543.2622	1.2	12.0	$C_{26} H_{37} O_{4} N_{7} S_{1}$
	543.2636	-1.3	-1.5	$C_{13} H_{43} O_{5} N_{12} S_{3}$
	543.2620	1.6	3.5	$C_{19} H_{39} O_{12} N_{6}$
	543.2620	1.6	9.0	$C_{18} H_{33} O_{7} N_{13}$
	543.2618	2.1	6.0	$C_{26} H_{45} O_{3} N_{3} S_{3}$
	543.2641	-2.2	-1.0	$C_{14} H_{41} O_{11} N_{9} S_{1}$
	543.2642	-2.4	20.5	$C_{36} H_{35} O_{3} N_{2}$
	543.2616	2.4	-2.5	$C_{19} H_{47} O_{11} N_{2} S_{2}$
	543.2616	2.4	3.0	$C_{18} H_{41} O_{6} N_{9} S_{2}$
	543.2643	-2.5	7.5	$C_{21} H_{39} O_{3} N_{10} S_{2}$
	543.2643	-2.5	2.0	$C_{22} H_{45} O_{8} N_{3} S_{2}$
	543.2615	2.5	16.0	$C_{33} H_{37} O_{6} N_{1}$
	543.2615	2.5	21.5	$C_{32} H_{31} O_{1} N_{8}$
	543.2614	2.8	0.0	$C_{10} H_{37} O_{9} N_{15} S_{1}$
	543.2644	-2.8	10.5	$C_{29} H_{43} N_{4} S_{3}$
	543.2647	-3.4	13.5	$C_{21} H_{31} O_{4} N_{14}$
	543.2647	-3.4	8.0	$C_{22} H_{37} O_{9} N_{7}$
	543.2647	-3.4	2.5	$C_{23} H_{43} O_{14}$
	543.2611	3.4	15.5	$C_{32} H_{39} N_{4} S_{2}$

Mass	Theoretical Mass	Delta [ppm]	RDB	Composition
	543.2649	-3.7	16.5	$C_{29} H_{35} O_{1} N_{8} S_{1}$
	543.2649	-3.7	11.0	$C_{30} H_{41} O_{6} N_{1} S_{1}$
	543.2609	3.7	7.0	$C_{25} H_{41} O_{8} N_{3} S_{1}$
	543.2609	3.7	12.5	$C_{24} H_{35} O_{3} N_{10} S_{1}$
	543.2649	-3.8	-2.0	$C_{15} H_{45} O_{6} N_{9} S_{3}$
	543.2607	4.0	4.0	$C_{17} H_{37} O_{11} N_{9}$
	543.2604	4.6	6.5	$C_{24} H_{43} O_{2} N_{6} S_{3}$
	543.2654	-4.6	4.0	$C_{15} H_{37} O_{7} N_{13} S_{1}$
	543.2654	-4.6	-1.5	$C_{16} H_{43} O_{12} N_{6} S_{1}$
	543.2602	4.9	-2.0	$C_{17} H_{45} O_{10} N_{5} S_{2}$
	543.2602	4.9	3.5	$C_{16} H_{39} O_{5} N_{12} S_{2}$
	543.2656	-5.0	7.0	$C_{23} H_{41} O_{4} N_{7} S_{2}$
	543.2656	-5.0	1.5	$C_{24} H_{47} O_{9} S_{2}$
	543.2602	5.0	16.5	$C_{31} H_{35} O_{5} N_{4}$
	543.2602	5.0	22.0	$C_{30} H_{29} N_{11}$

15-177-a
(DCM)/MeOH + NH4OAc
C15H20N2O3

Compound 46

EPSRC National Facility Swansea
LTQ Orbitrap XL

BRASHE
23/01/2017 11:34:10

BRASHE_0U5ZN_ #6-12 RT: 0.11-0.37 AV: 6 SM: 7G NL: 3.59E6
T: FTMS + p NSI Full ms [120.00-1935.00]

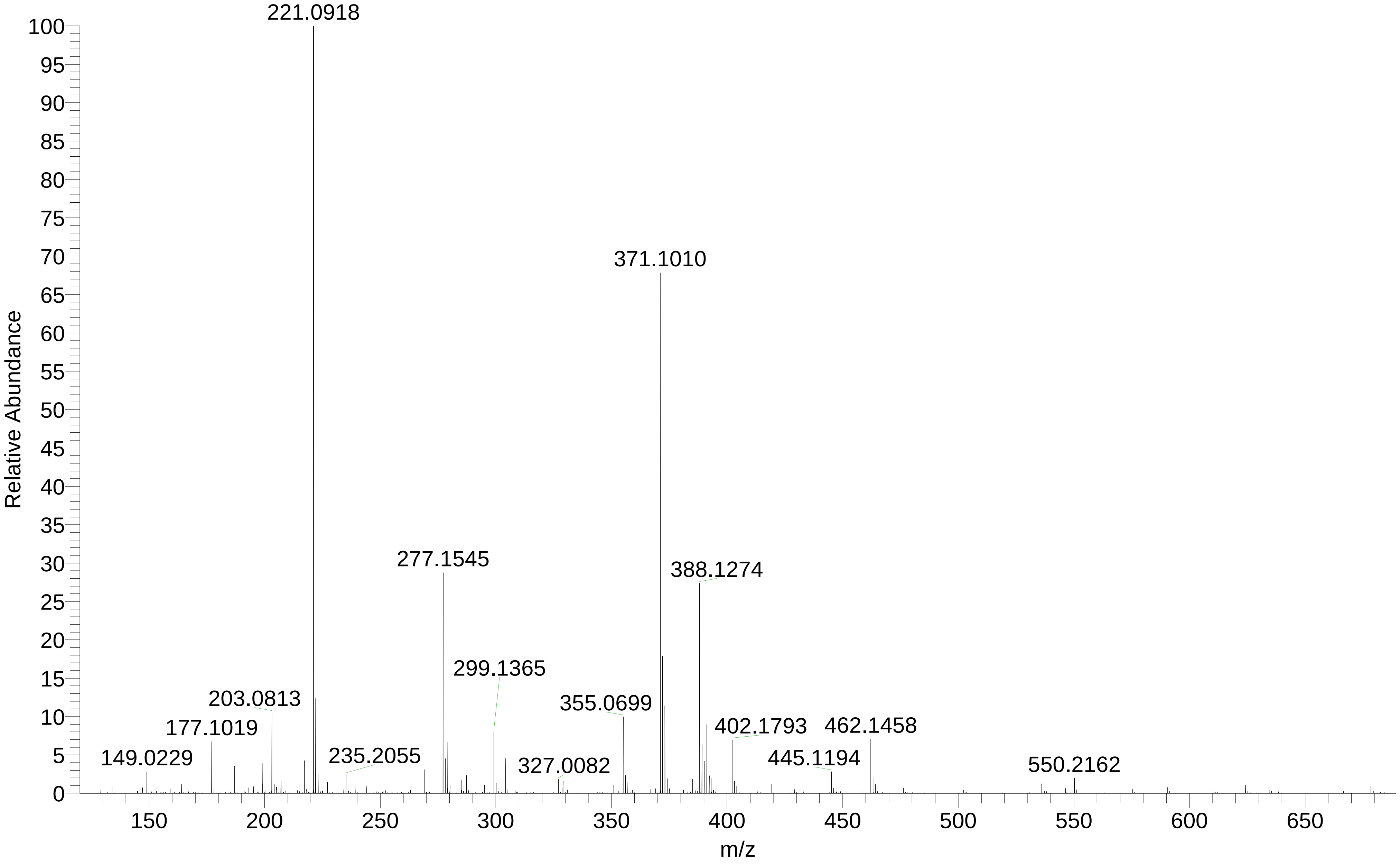

15-177-a
(DCM)/MeOH + NH4OAc
C15H20N2O3

EPSRC National Facility Swansea
LTQ Orbitrap XL

BRASHE
23/01/2017 11:34:10

BRASHE_0U5ZN_ #6-12 RT: 0.11-0.37 AV: 6 SM: 7G NL: 3.59E6
T: FTMS + p NSI Full ms [120.00-1935.00]

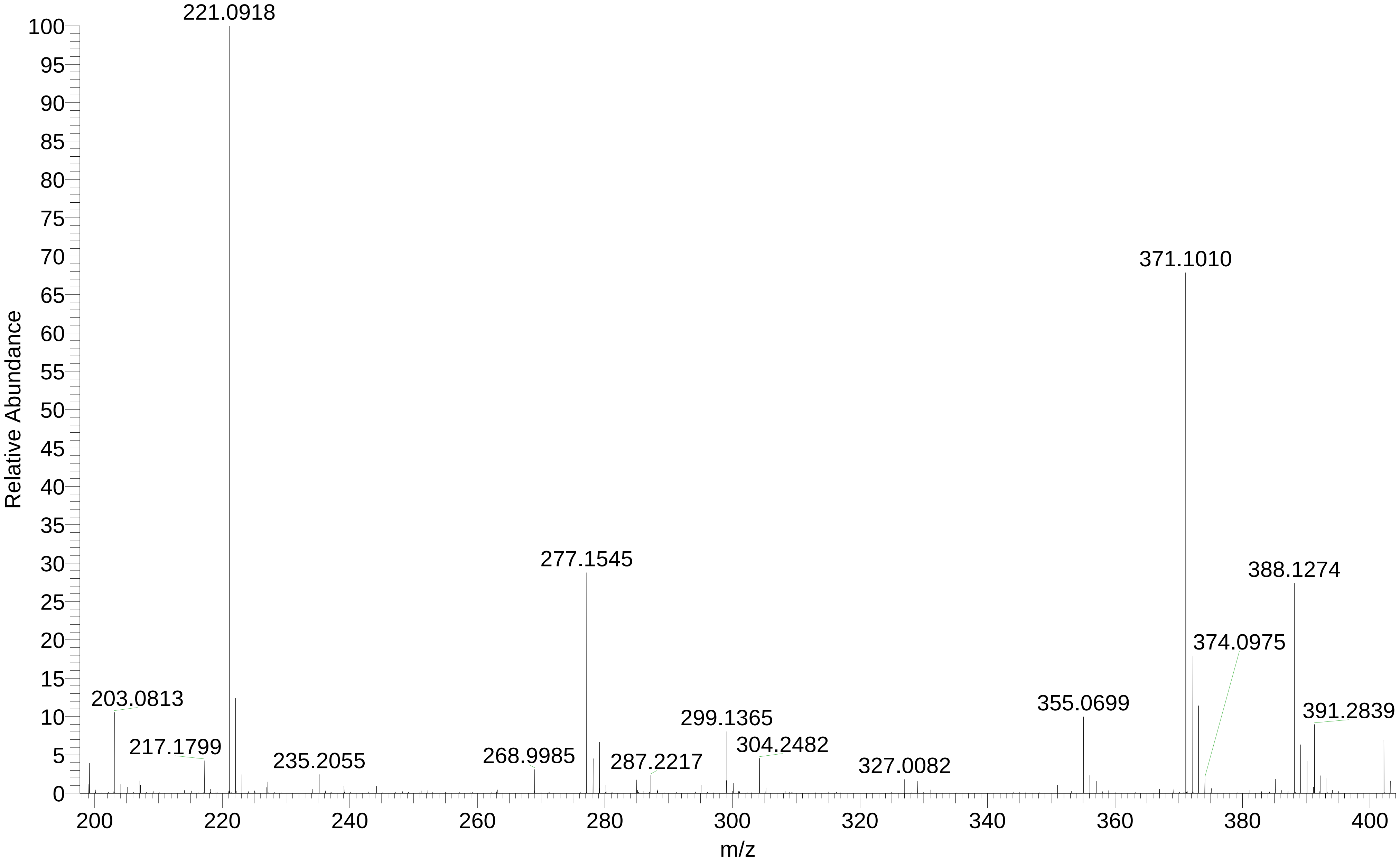

15-177-a
(DCM)/MeOH + NH4OAc
C15H20N2O3

EPSRC National Facility Swansea
LTQ Orbitrap XL

BRASHE
23/01/2017 11:34:10

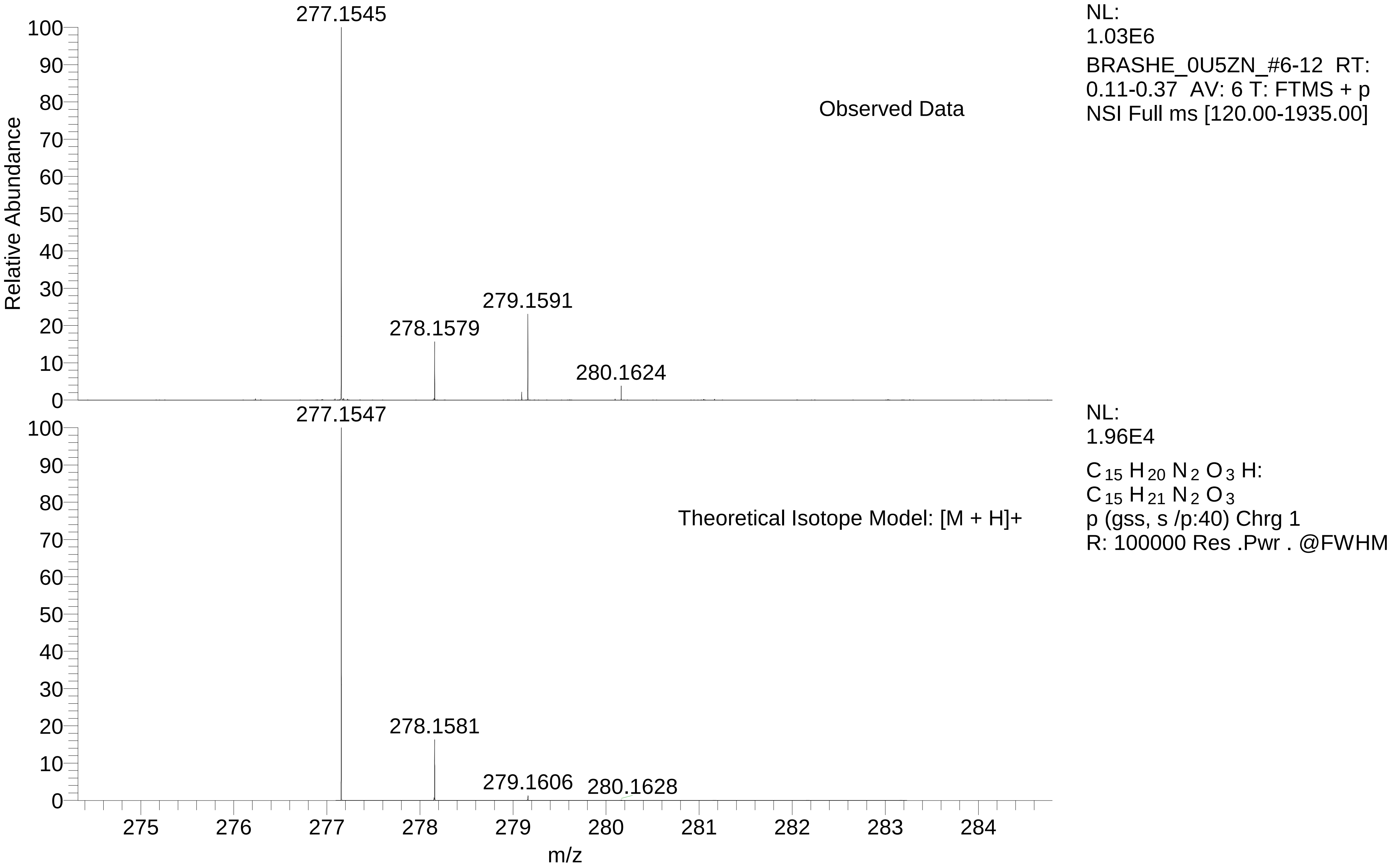

**Isotope:**	**Min. .. Max.**
**14 N**	**0....15**
**16 O**	**0....15**
**12 C**	**0....60**
**1 H**	**0....70**
**23 Na**	**0....0**
**Tolerance Window:**	**+- 5.00 ppm**
**Db/Ring Equiv:**	**-3.. 100**
**Fits:**	**100**

N-Rule: **Do not use**
Charge: **1**

**Mass**	**Theoretical Mass**	**Delta [ppm]**	**RDB**	**Composition**
277.1545	277.1547	-0.6	6.5	$C_{15} H_{21} O_3 N_2$
	277.1552	-2.4	-0.5	$H_{17} O_4 N_{14}$
	277.1533	4.2	7.0	$C_{13} H_{19} O_2 N_5$

aet7-23 MW=278?
(?)/MeOH + NH4OAc

Compound 43

EPSRC National Centre Swansea
LTQ Orbitrap XL

adam throup
05/12/2011 13:14:33

BRASHE163-PV-HNESP #20-35 RT: 0.82-1.25 AV: 16 SM: 7G NL: 2.40E7
T: FTMS + p NSI Full ms [120.00-2000.00]

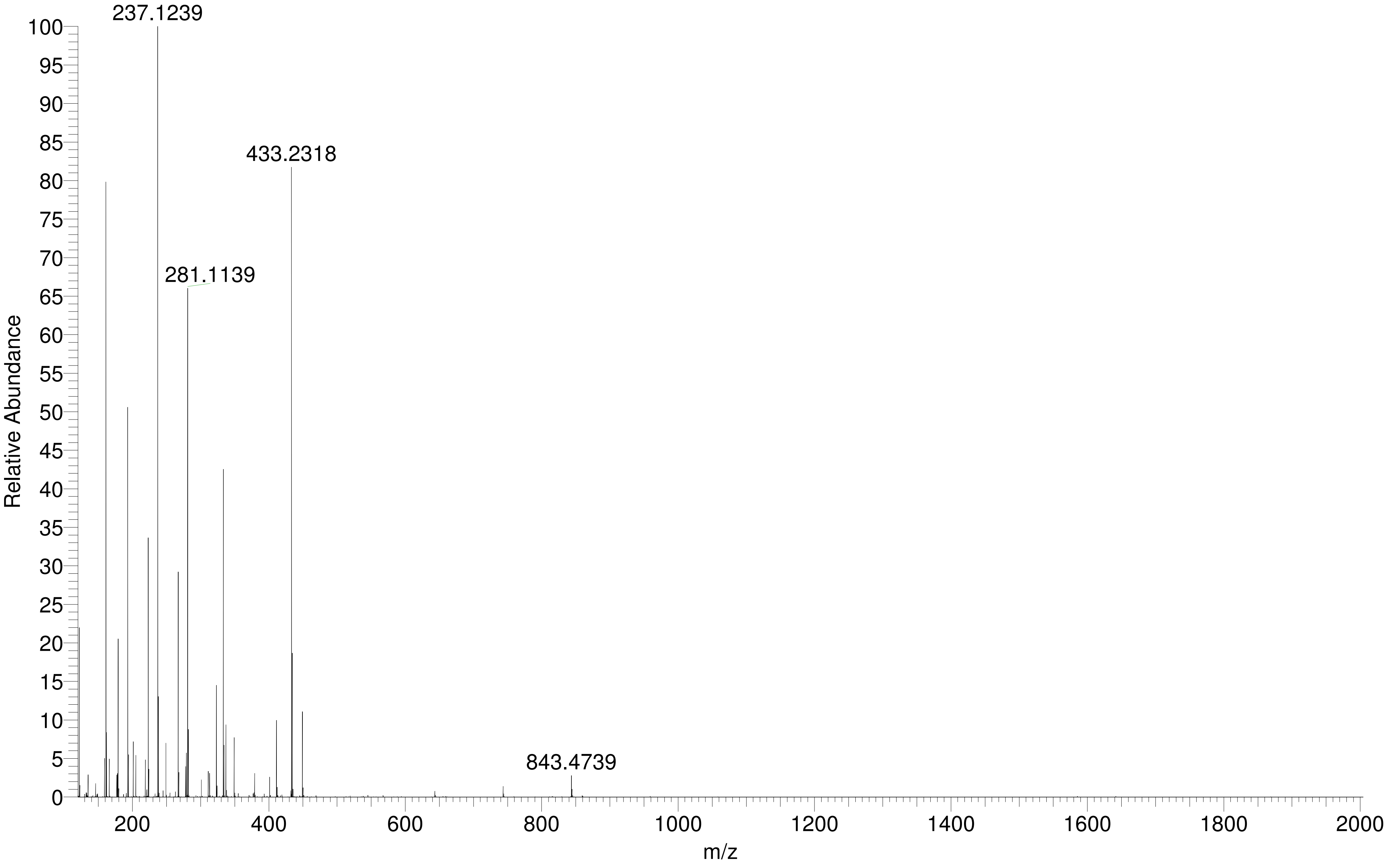

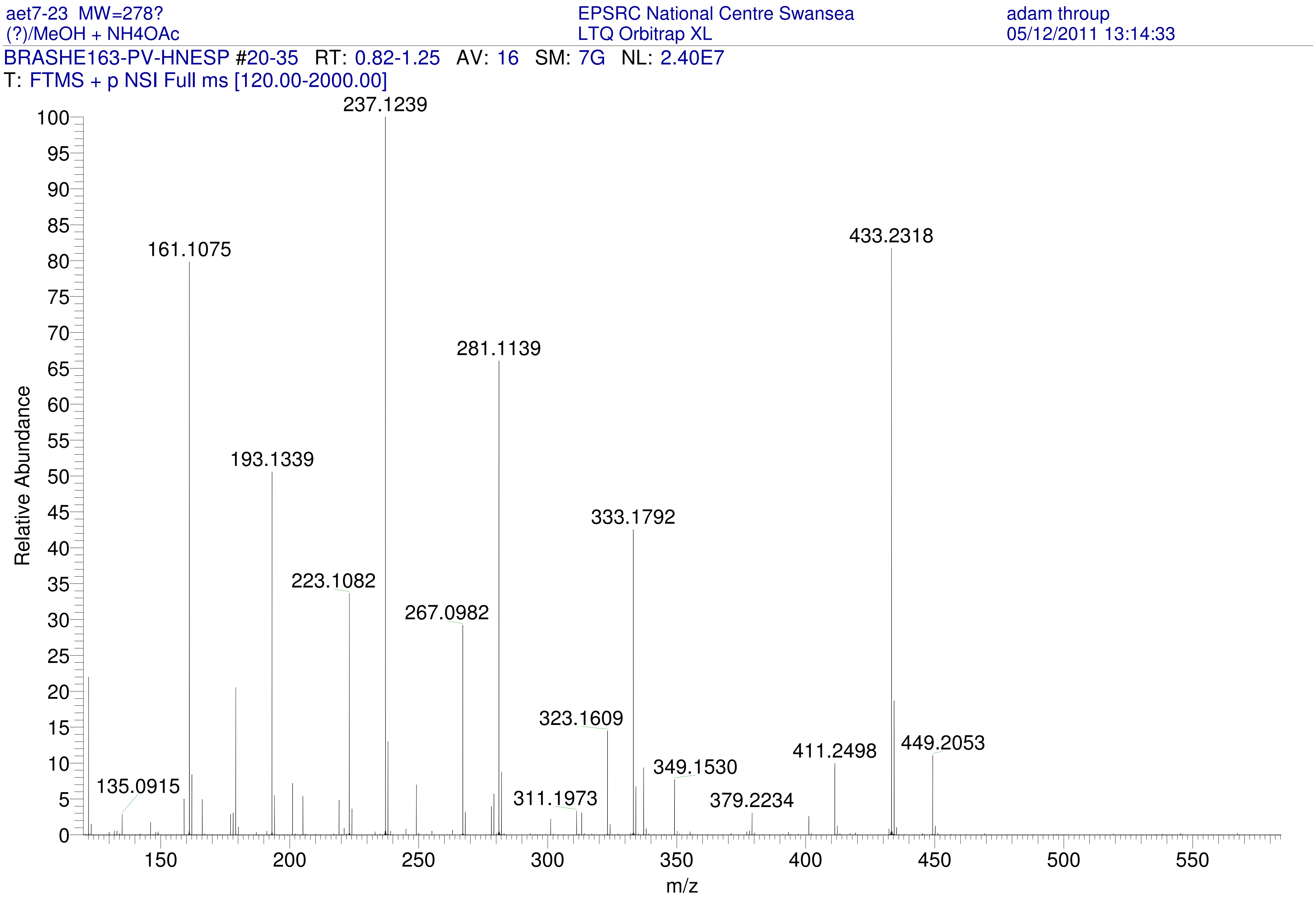
aet7-23 MW=278?
(?)/MeOH + NH4OAc
EPSRC National Centre Swansea
LTQ Orbitrap XL
adam throup
05/12/2011 13:14:33
BRASHE163-PV-HNESP #20-35 RT: 0.82-1.25 AV: 16 SM: 7G NL: 2.40E7
T: FTMS + p NSI Full ms [120.00-2000.00]
237.1239
433.2318
161.1075
281.1139
193.1339
333.1792
223.1082
267.0982
323.1609
349.1530
411.2498
449.2053
135.0915
311.1973
379.2234
Relative Abundance
m/z

aet7-23 MW=278?
(?)/MeOH + NH4OAc

EPSRC National Centre Swansea
LTQ Orbitrap XL

adam throup
05/12/2011 13:14:33

BRASHE163-PV-HNESP #20-35 RT: 0.82-1.25 AV: 16 SM: 7G NL: 1.38E6
T: FTMS + p NSI Full ms [120.00-2000.00]

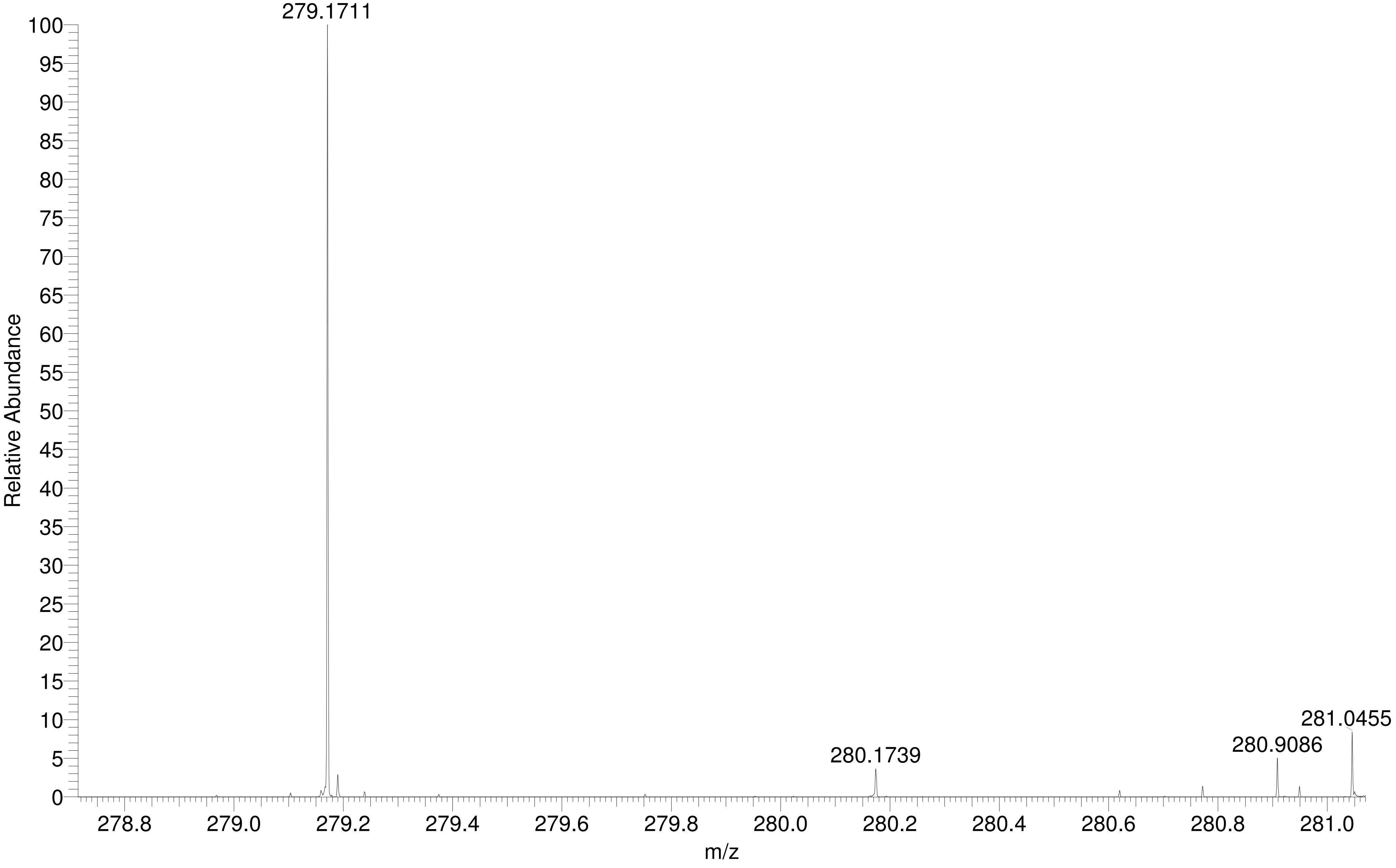

Isotope:	Min. .. Max.		
14 N	0....10		
16 O	0....15		
12 C	0....60		
1 H	0....90		
23 Na	0....0		
Tolerance Window:	+- 5.00 ppm		
Db/Ring Equiv:	-3.. 100	N-Rule:	Do not use
Fits:	100	Charge:	1

Mass	Theoretical Mass	Delta [ppm]	RDB	Composition
279.1711	279.1703	2.8	5.5	$C_{15} H_{23} O_3 N_2$

Compound 47

aet7-26 MW=345?
ASAP

EPSRC National Centre Swansea
LTQ Orbitrap XL

adam throup
13/12/2011 14:03:44

BRASHE168-OC-HASP #32-33 RT: 1.01-1.04 AV: 2 SM: 7G NL: 2.20E8
T: FTMS + p APCI corona Full ms [140.00-800.00]

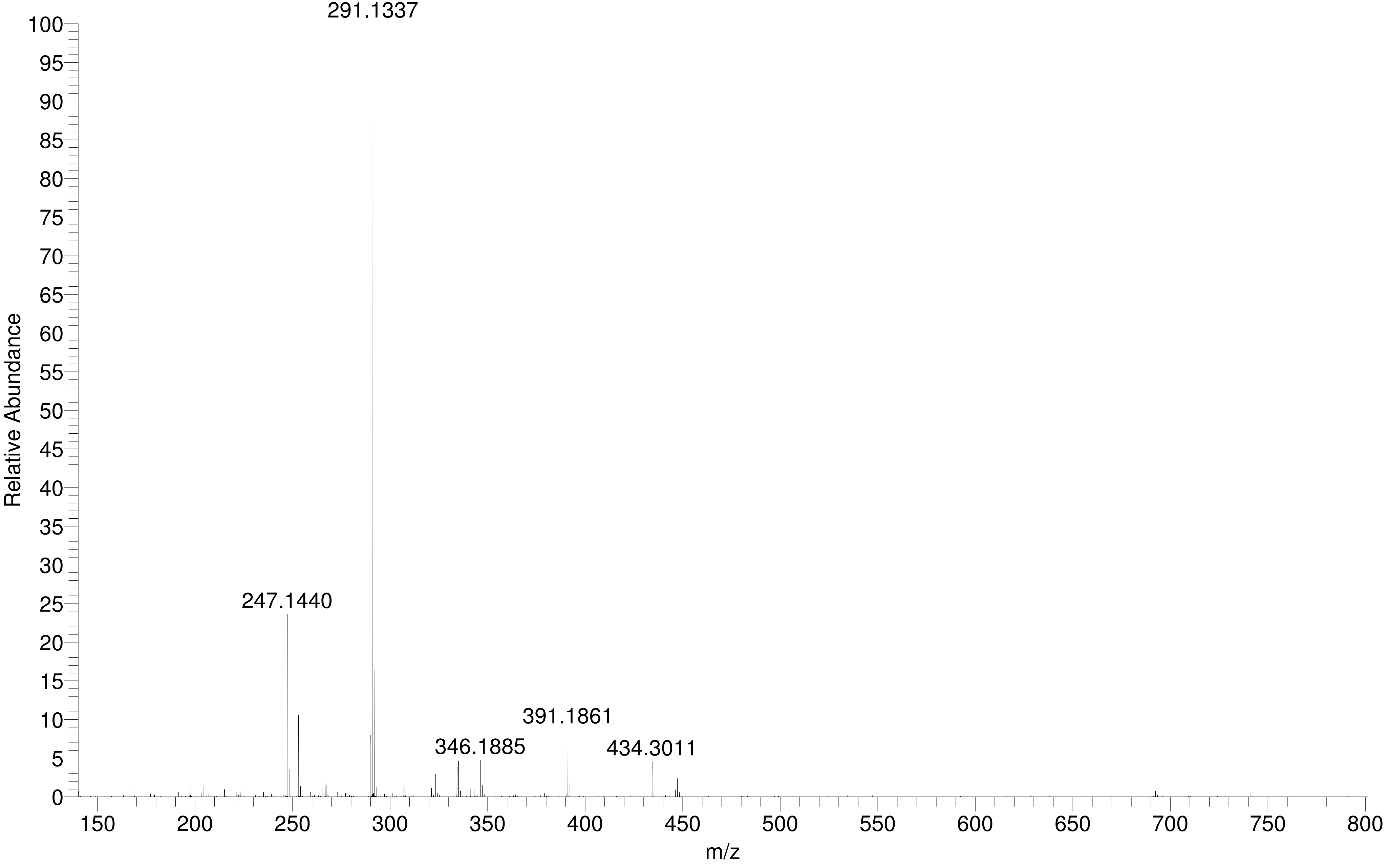

aet7-26 MW=345?
ASAP

EPSRC National Centre Swansea
LTQ Orbitrap XL

adam throup
13/12/2011 14:03:44

BRASHE168-OC-HASP #32-33 RT: 1.01-1.04 AV: 2 SM: 7G NL: 1.91E7
T: FTMS + p APCI corona Full ms [140.00-800.00]

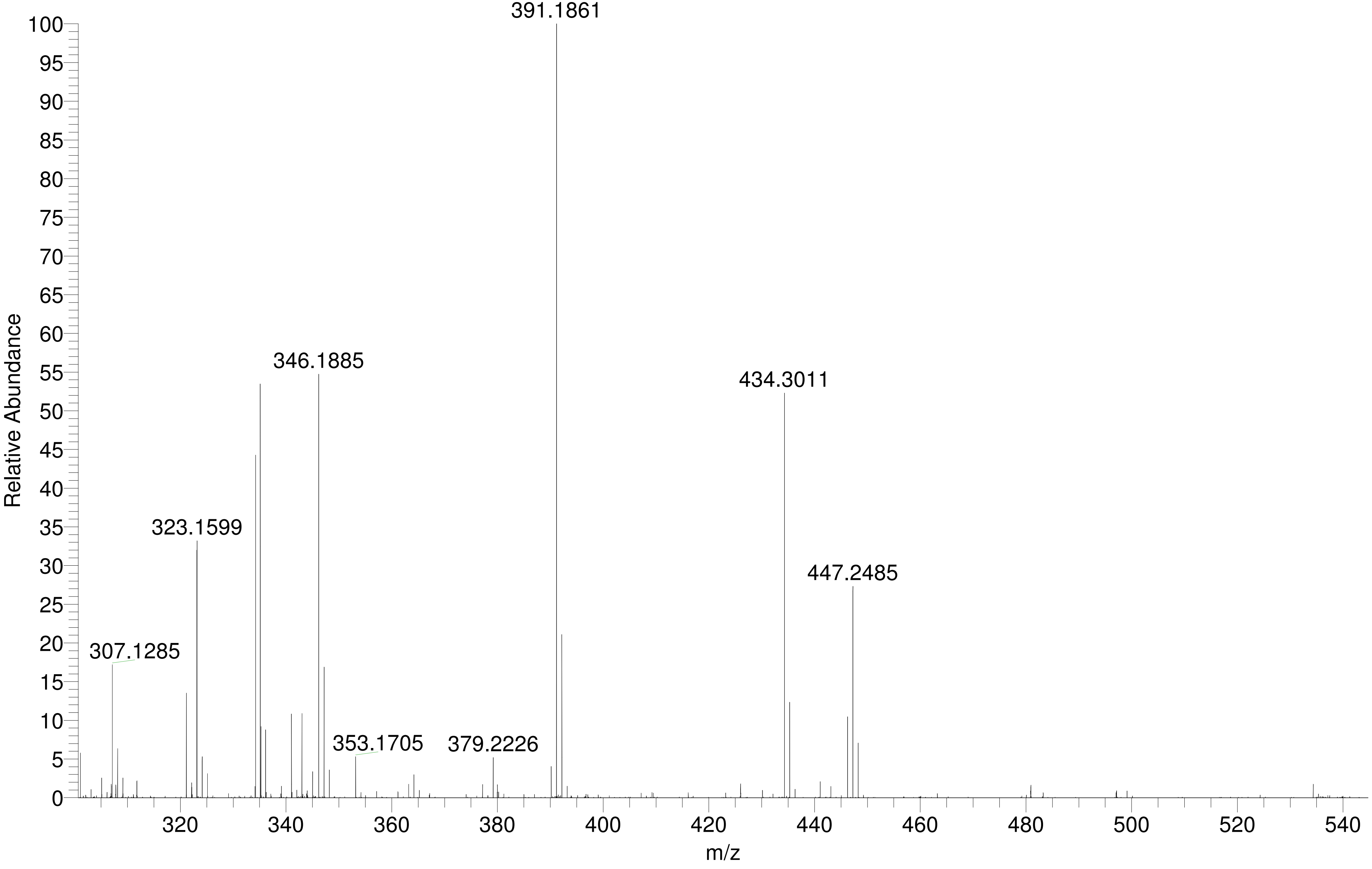

aet7-26 MW=345?
ASAP

EPSRC National Centre Swansea
LTQ Orbitrap XL

adam throup
13/12/2011 14:03:44

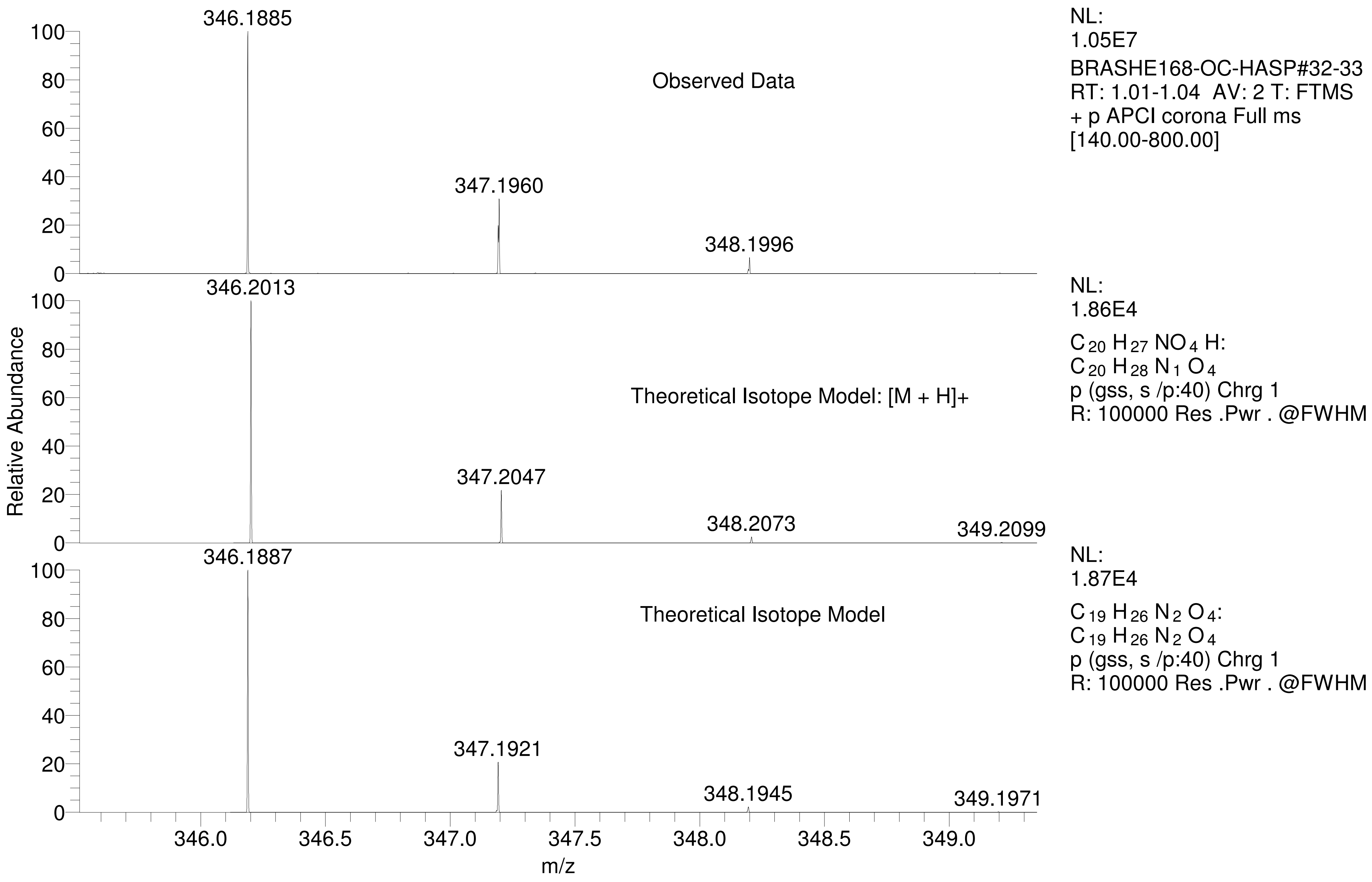

Isotope:	Min. .. Max.		
12 C	0....40		
1 H	0....60		
14 N	0....10		
16 O	0....10		
23 Na	0....0		
Tolerance Window:	+- 5.00 ppm		
Db/Ring Equiv:	-3.. 100	N-Rule:	Do not use
Fits:	200	Charge:	1

Mass	Theoretical Mass	Delta [ppm]	RDB	Composition
346.1885	346.1887	-0.6	8.0	$C_{19} H_{26} O_4 N_2$
	346.1874	3.3	8.5	$C_{17} H_{24} O_3 N_5$
	346.1900	-4.5	13.0	$C_{20} H_{22} N_6$

Compound 48

aet7-27 MW=347?
ASAP

EPSRC National Centre Swansea
LTQ Orbitrap XL

adam throup
13/12/2011 14:15:40

BRASHE169-OC-HASP #17 RT: 0.62 AV: 1 SM: 7G NL: 3.86E8
T: FTMS + p APCI corona Full ms [140.00-800.00]

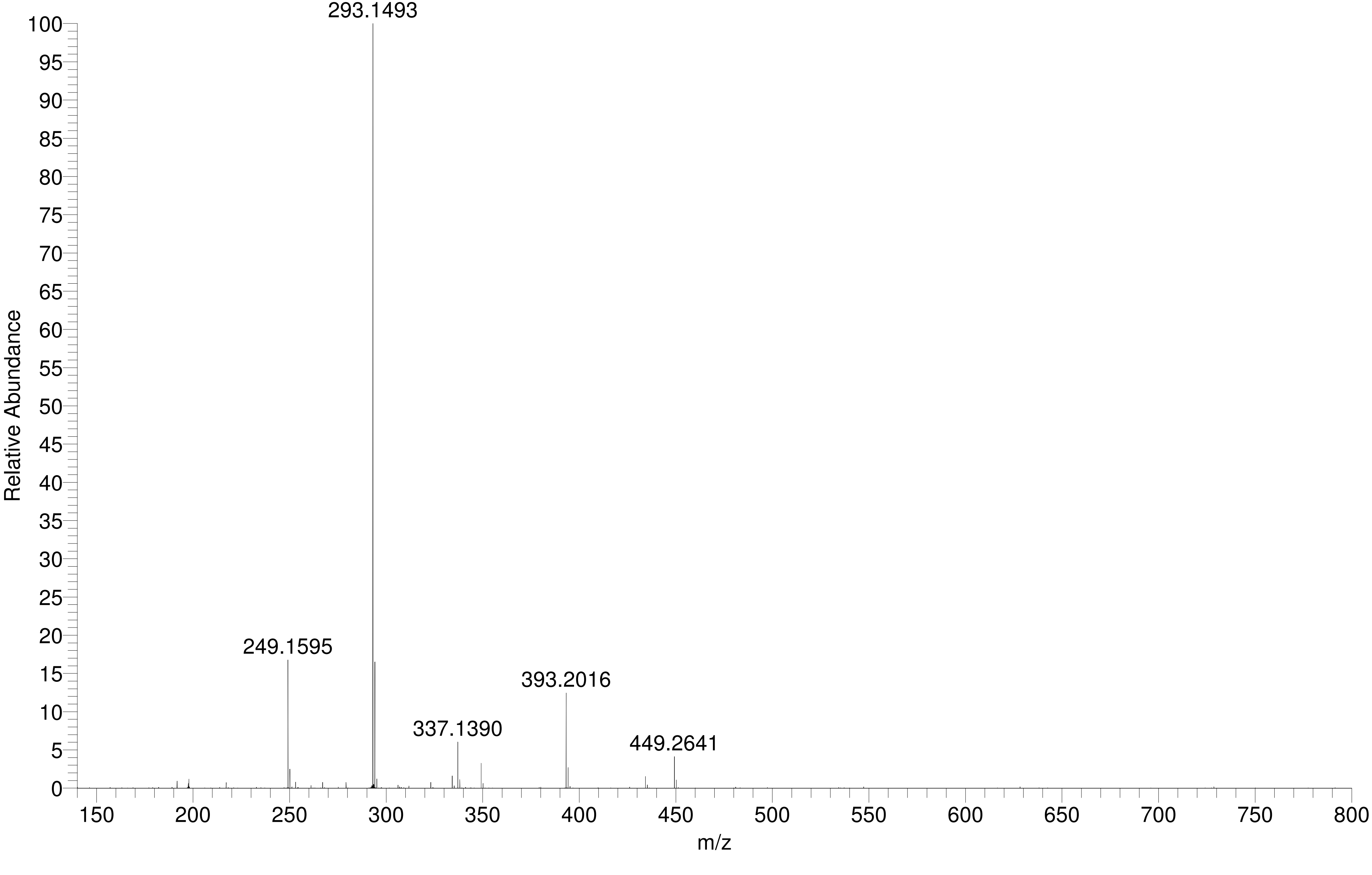

aet7-27 MW=347?
ASAP

EPSRC National Centre Swansea
LTQ Orbitrap XL

adam throup
13/12/2011 14:15:40

BRASHE169-OC-HASP #17 RT: 0.62 AV: 1 SM: 7G NL: 3.86E8
T: FTMS + p APCI corona Full ms [140.00-800.00]

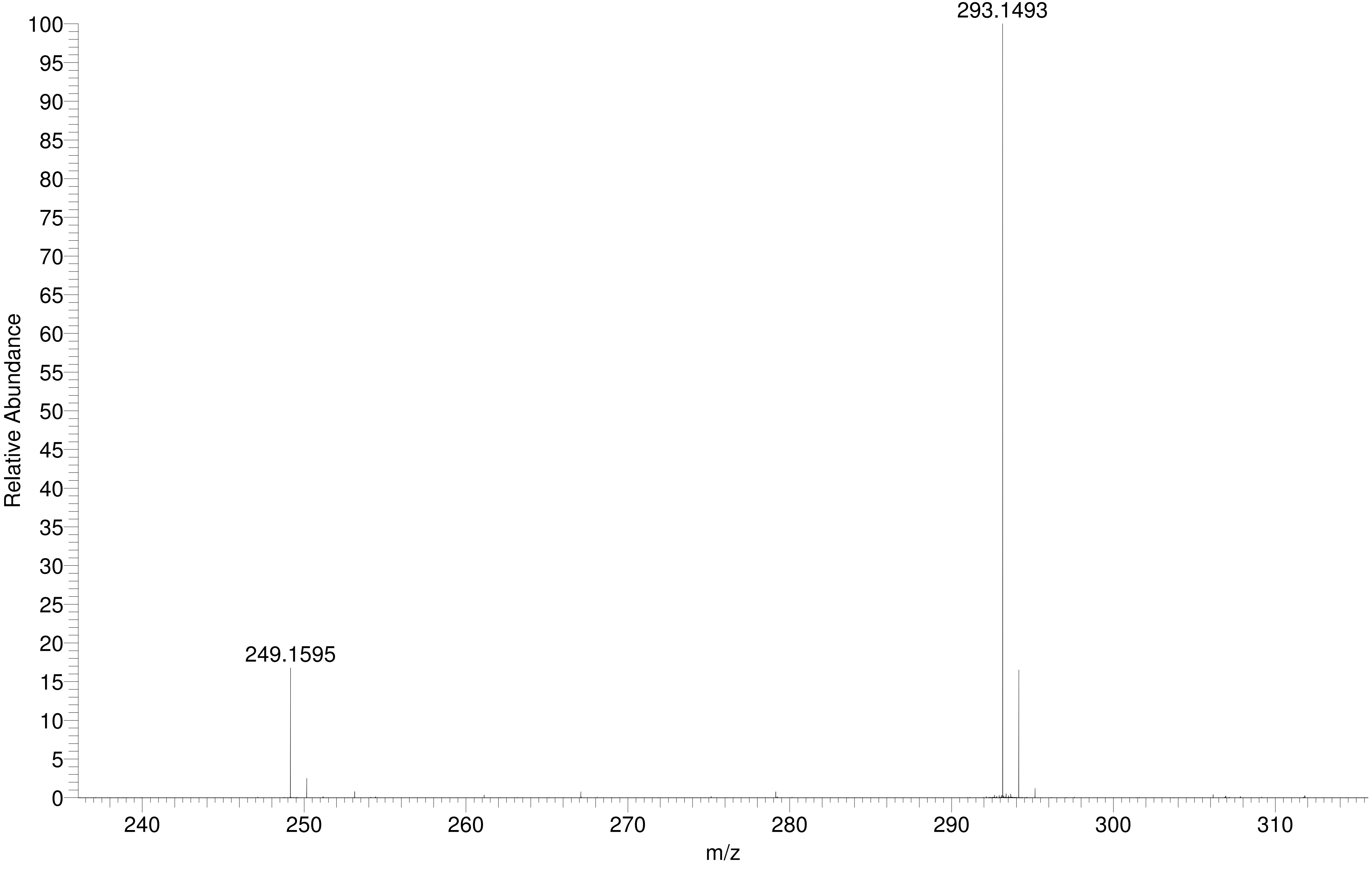

aet7-27 MW=347?
ASAP

EPSRC National Centre Swansea
LTQ Orbitrap XL

adam throup
13/12/2011 14:15:40

BRASHE169-OC-HASP #17 RT: 0.62 AV: 1 SM: 7G NL: 4.81E7
T: FTMS + p APCI corona Full ms [140.00-800.00]

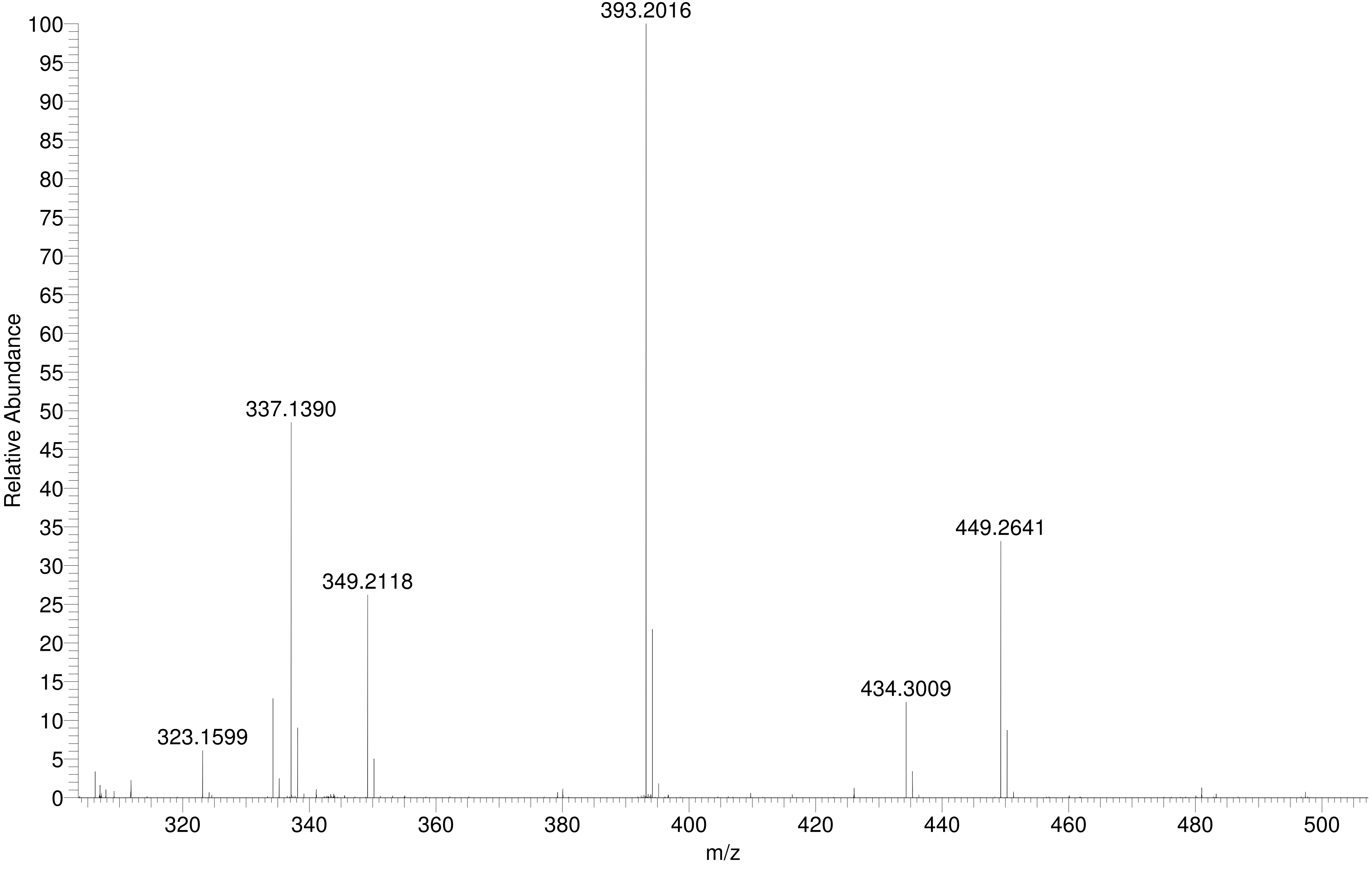

aet7-27 MW=347?
ASAP

EPSRC National Centre Swansea
LTQ Orbitrap XL

adam throup
13/12/2011 14:15:40

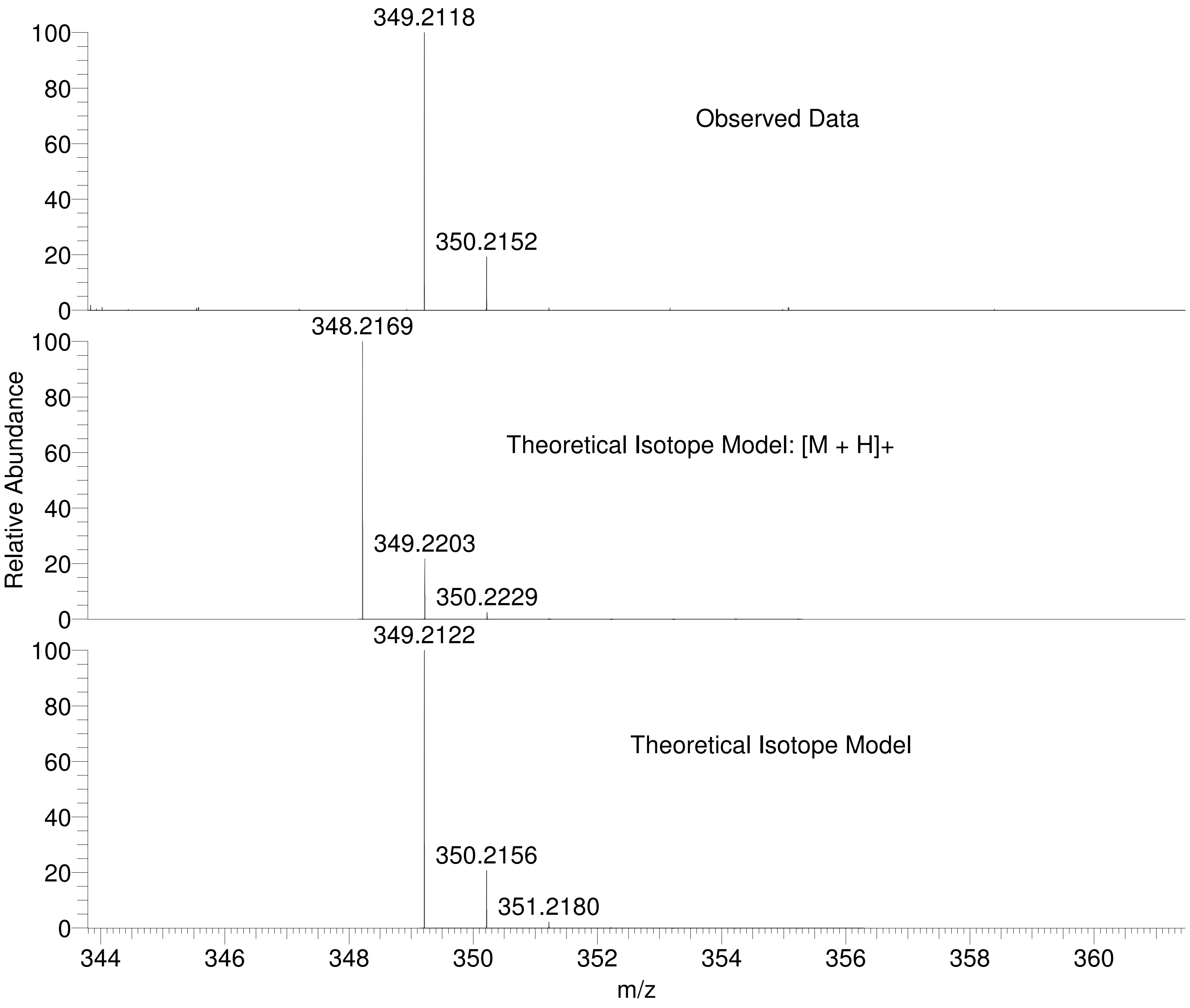

NL:
1.26E7
BRASHE169-OC-HASP#17
RT: 0.62 AV: 1 T: FTMS + p
APCI corona Full ms
[140.00-800.00]

NL:
1.86E4
$C_{20}H_{29}NO_4$ H:
$C_{20}H_{30}N_1O_4$
p (gss, s /p:40) Chrg 1
R: 100000 Res .Pwr . @FWHM

NL:
1.87E4
$C_{19}H_{29}N_2O_4$:
$C_{19}H_{29}N_2O_4$
p (gss, s /p:40) Chrg 1
R: 100000 Res .Pwr . @FWHM

Isotope:	Min. .. Max.		
12 C	0....40		
1 H	0....60		
14 N	0....10		
16 O	0....10		
23 Na	0....0		
Tolerance Window:	+- 5.00 ppm		
Db/Ring Equiv:	-3.. 100	N-Rule:	Do not use
Fits:	200	Charge:	1

Mass	Theoretical Mass	Delta [ppm]	RDB	Composition
349.2118	349.2122	-1.1	6.5	$C_{19} H_{29} O_4 N_2$
	349.2108	2.7	7.0	$C_{17} H_{27} O_3 N_5$
	349.2135	-4.9	11.5	$C_{20} H_{25} N_6$

aet7-39 MW=574?
(DCM)/MeOH + NH4OAc

Compound 51

EPSRC National Centre Swansea
LTQ Orbitrap XL

adam throup
13/01/2012 14:33:35

BRASHE178-OV-HNESP #21-34 RT: 0.88-1.26 AV: 14 NL: 5.08E6
T: FTMS + p NSI Full ms [120.00-2000.00]

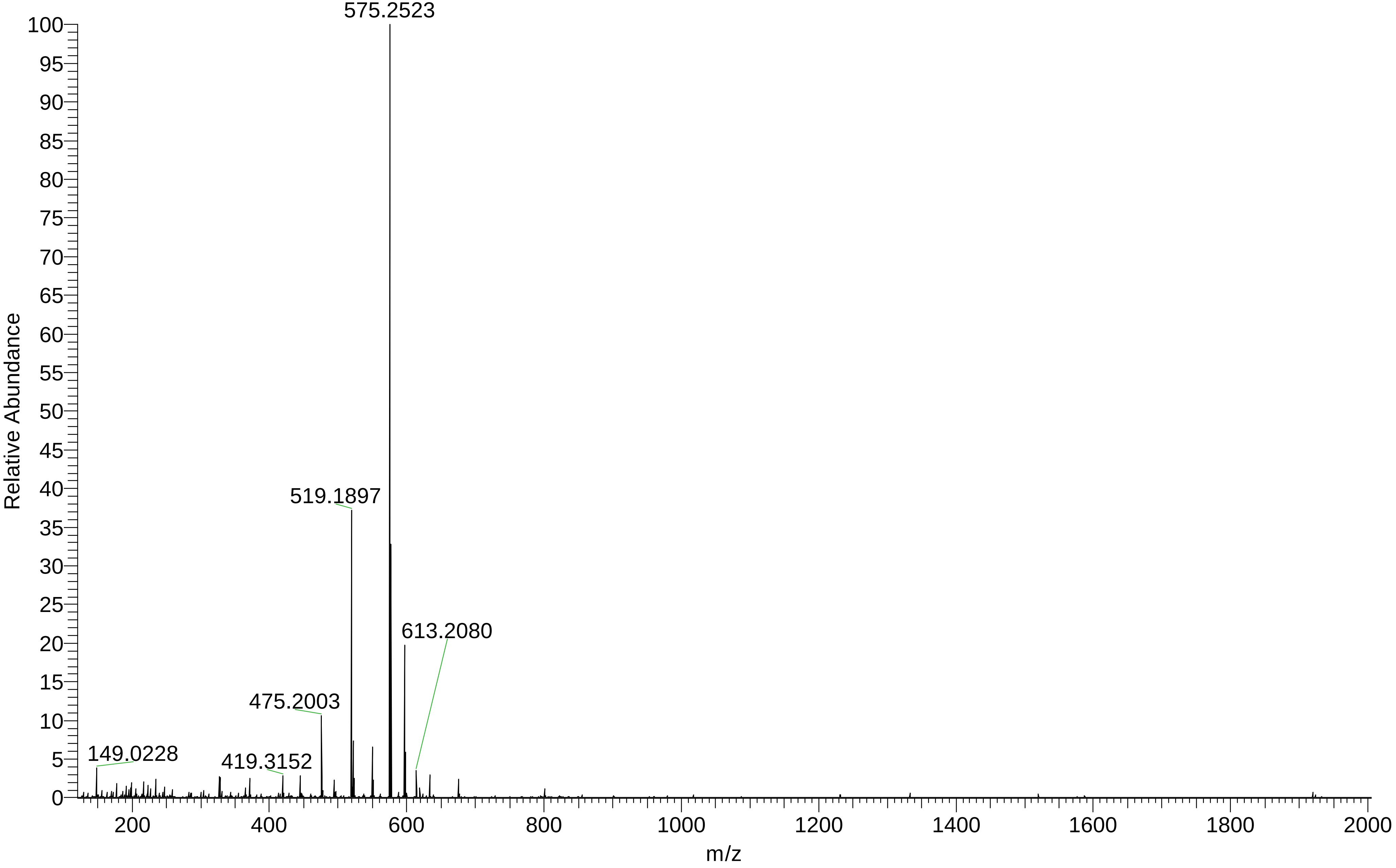

aet7-39 MW=574?
(DCM)/MeOH + NH4OAc

EPSRC National Centre Swansea
LTQ Orbitrap XL

adam throup
13/01/2012 14:33:35

BRASHE178-OV-HNESP #21-34 RT: 0.88-1.26 AV: 14 NL: 5.08E6
T: FTMS + p NSI Full ms [120.00-2000.00]

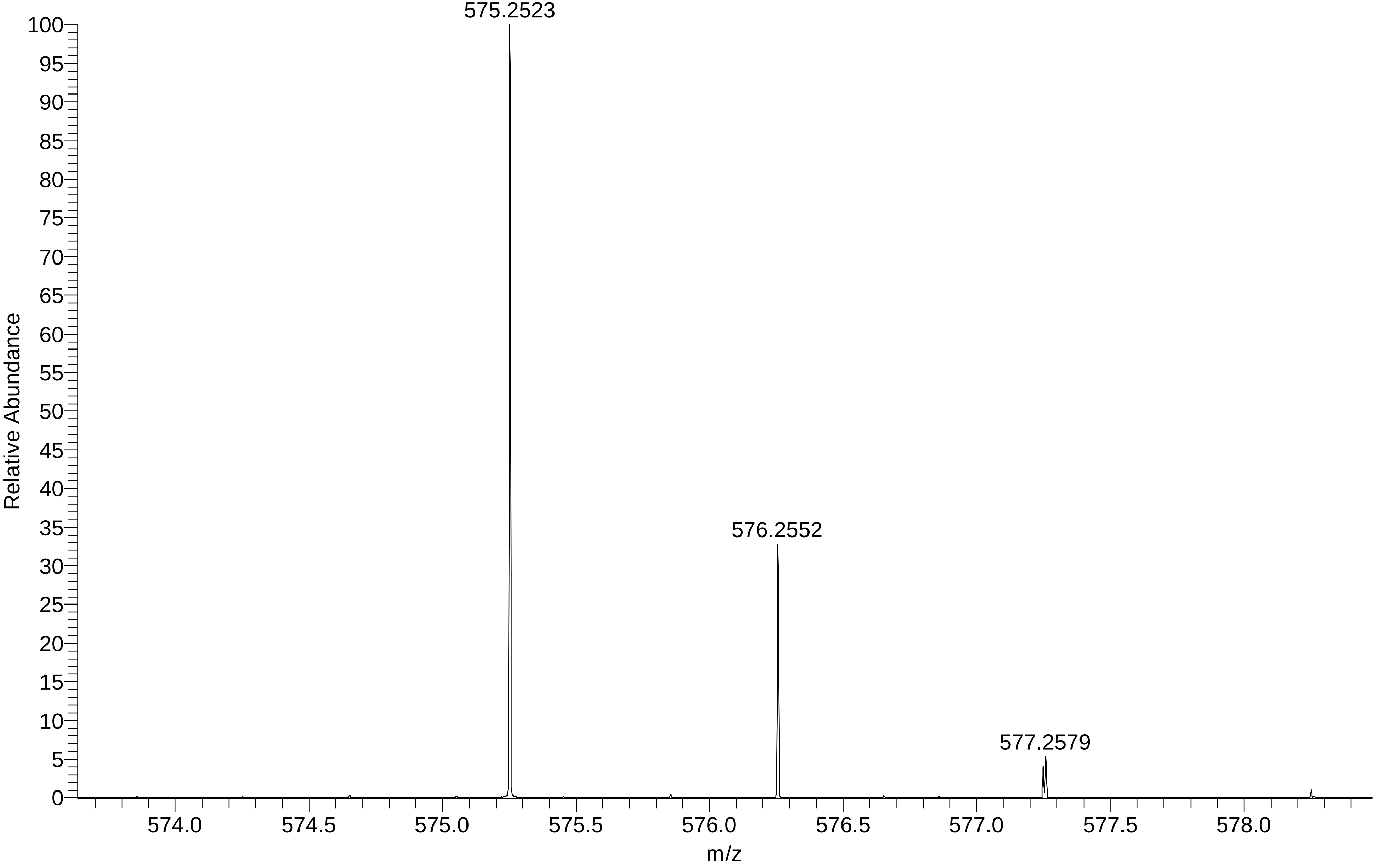

Isotope: **Min. .. Max.**
14 N **0....10**
16 O **0....15**
12 C **0....30**
1 H **0....60**
23 Na **0....0**
32 S **0....2**
Tolerance Window: **+- 5.00 ppm**
Db/Ring Equiv: **-3..100** **N-Rule:** **Do not use**
Fits: **100** **Charge:** **1**

Mass	Theoretical Mass	Delta [ppm]	RDB	Composition
575.2523	575.2521	0.4	6.5	$C_{27} H_{43} O_{11} S_1$
	575.2521	0.4	12.0	$C_{26} H_{37} O_6 N_7 S_1$
	575.2519	0.7	3.5	$C_{19} H_{39} O_{14} N_6$
	575.2527	-0.8	2.5	$C_{20} H_{43} O_9 N_6 S_2$
	575.2514	1.6	-2.5	$C_{19} H_{47} O_{13} N_2 S_2$
	575.2514	1.6	3.0	$C_{18} H_{41} O_8 N_9 S_2$
	575.2532	-1.6	8.5	$C_{20} H_{35} O_{10} N_{10}$
	575.2532	-1.6	3.0	$C_{21} H_{41} O_{15} N_3$
	575.2534	-1.9	11.5	$C_{28} H_{39} O_7 N_4 S_1$
	575.2507	2.8	7.0	$C_{25} H_{41} O_{10} N_3 S_1$
	575.2507	2.8	12.5	$C_{24} H_{35} O_5 N_{10} S_1$
	575.2539	-2.8	-1.0	$C_{14} H_{41} O_{13} N_9 S_1$
	575.2505	3.1	4.0	$C_{17} H_{37} O_{13} N_9$
	575.2541	-3.1	7.5	$C_{21} H_{39} O_5 N_{10} S_2$
	575.2541	-3.1	2.0	$C_{22} H_{45} O_{10} N_3 S_2$
	575.2501	3.9	-2.0	$C_{17} H_{45} O_{12} N_5 S_2$
	575.2546	-3.9	8.0	$C_{22} H_{37} O_{11} N_7$
	575.2547	-4.2	16.5	$C_{29} H_{35} O_3 N_8 S_1$
	575.2547	-4.2	11.0	$C_{30} H_{41} O_8 N_1 S_1$
	575.2496	4.8	16.0	$C_{30} H_{37} O_1 N_7 S_2$

aet7-47 MW=616?
(DCM)/MeOH + NH4OAc

Compound 52

EPSRC National Centre Swansea
LTQ Orbitrap XL

adam throup
13/01/2012 14:40:48

BRASHE180-OV-HNESP #20-33 RT: 0.87-1.26 AV: 14 NL: 2.51E6
T: FTMS + p NSI Full ms [120.00-2000.00]

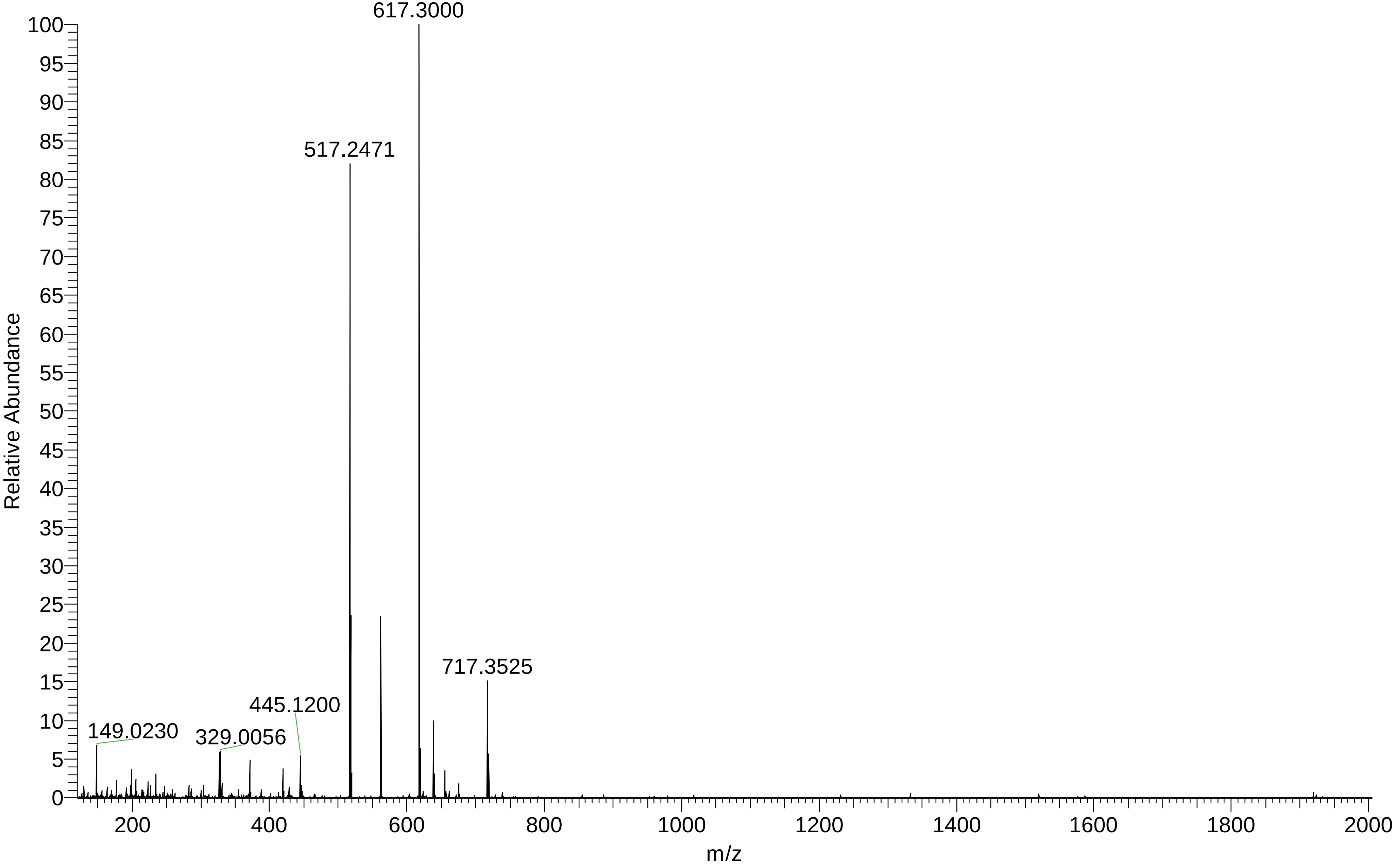

aet7-47 MW=616?
(DCM)/MeOH + NH4OAc

EPSRC National Centre Swansea
LTQ Orbitrap XL

adam throup
13/01/2012 14:40:48

BRASHE180-OV-HNESP #20-33 RT: 0.87-1.26 AV: 14 NL: 2.51E6
T: FTMS + p NSI Full ms [120.00-2000.00]

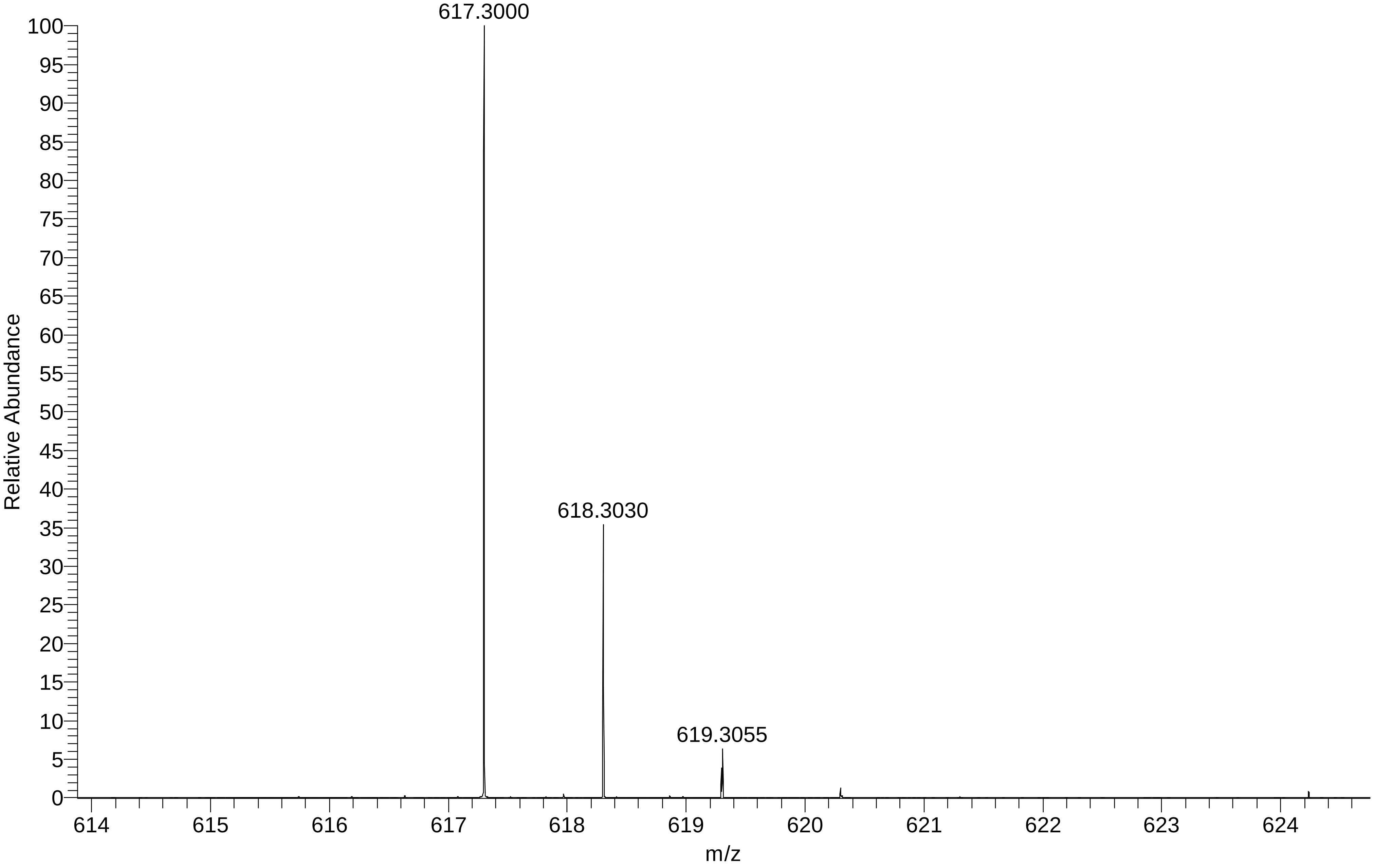

Isotope: **Min. .. Max.**
14 N **0....10**
16 O **0....15**
12 C **0....40**
1 H **0....60**
23 Na **0....0**
32 S **0....2**
Tolerance Window: **+- 5.00 ppm**
Db/Ring Equiv: **-3..100** **N-Rule:** **Do not use**
Fits: **100** **Charge:** **1**

**Mass**	**Theoretical Mass**	**Delta [ppm]**	**RDB**	**Composition**
617.3000	617.3002	-0.3	8.5	$C_{23} H_{41} O_{10} N_{10}$
	617.3002	-0.3	3.0	$C_{24} H_{47} O_{15} N_{3}$
	617.2997	0.5	2.5	$C_{23} H_{49} O_{9} N_{6} S_{2}$
	617.2997	0.6	21.0	$C_{37} H_{39} O_{4} N_{5}$
	617.3003	-0.6	11.5	$C_{31} H_{45} O_{7} N_{4} S_{1}$
	617.2992	1.3	15.0	$C_{37} H_{47} O_{3} N_{1} S_{2}$
	617.3009	-1.4	-1.0	$C_{17} H_{47} O_{13} N_{9} S_{1}$
	617.2990	1.6	6.5	$C_{30} H_{49} O_{11} S_{1}$
	617.3010	-1.6	26.0	$C_{38} H_{35} N_{9}$
	617.2990	1.6	12.0	$C_{29} H_{43} O_{6} N_{7} S_{1}$
	617.3010	-1.6	20.5	$C_{39} H_{41} O_{5} N_{2}$
	617.3010	-1.7	7.5	$C_{24} H_{45} O_{5} N_{10} S_{2}$
	617.3010	-1.7	2.0	$C_{25} H_{51} O_{10} N_{3} S_{2}$
	617.2988	1.9	3.5	$C_{22} H_{45} O_{14} N_{6}$
	617.3015	-2.4	8.0	$C_{25} H_{43} O_{11} N_{7}$
	617.2984	2.7	-2.5	$C_{22} H_{53} O_{13} N_{2} S_{2}$
	617.2984	2.7	3.0	$C_{21} H_{47} O_{8} N_{9} S_{2}$
	617.2983	2.7	16.0	$C_{36} H_{43} O_{8} N_{1}$
	617.3017	-2.7	16.5	$C_{32} H_{41} O_{3} N_{8} S_{1}$
	617.2983	2.7	21.5	$C_{35} H_{37} O_{3} N_{8}$
	617.3017	-2.7	11.0	$C_{33} H_{47} O_{8} N_{1} S_{1}$
	617.3019	-3.0	19.5	$C_{40} H_{45} N_{2} S_{2}$
	617.2978	3.5	15.5	$C_{35} H_{45} O_{2} N_{4} S_{2}$
	617.3022	-3.6	-1.5	$C_{19} H_{49} O_{14} N_{6} S_{1}$
	617.2977	3.8	7.0	$C_{28} H_{47} O_{10} N_{3} S_{1}$
	617.3023	-3.8	25.5	$C_{40} H_{37} O_{1} N_{6}$
	617.2977	3.8	12.5	$C_{27} H_{41} O_{5} N_{10} S_{1}$
	617.3024	-3.8	7.0	$C_{26} H_{47} O_{6} N_{7} S_{2}$
	617.3024	-3.9	1.5	$C_{27} H_{53} O_{11} S_{2}$
	617.2975	4.1	4.0	$C_{20} H_{43} O_{13} N_{9}$
	617.3028	-4.6	7.5	$C_{27} H_{45} O_{12} N_{4}$

aet7-42 MW=474?
(DCM)/MeOH + NH4OAc

Compound 53

EPSRC National Centre Swansea
LTQ Orbitrap XL

adam throup
13/01/2012 14:37:12

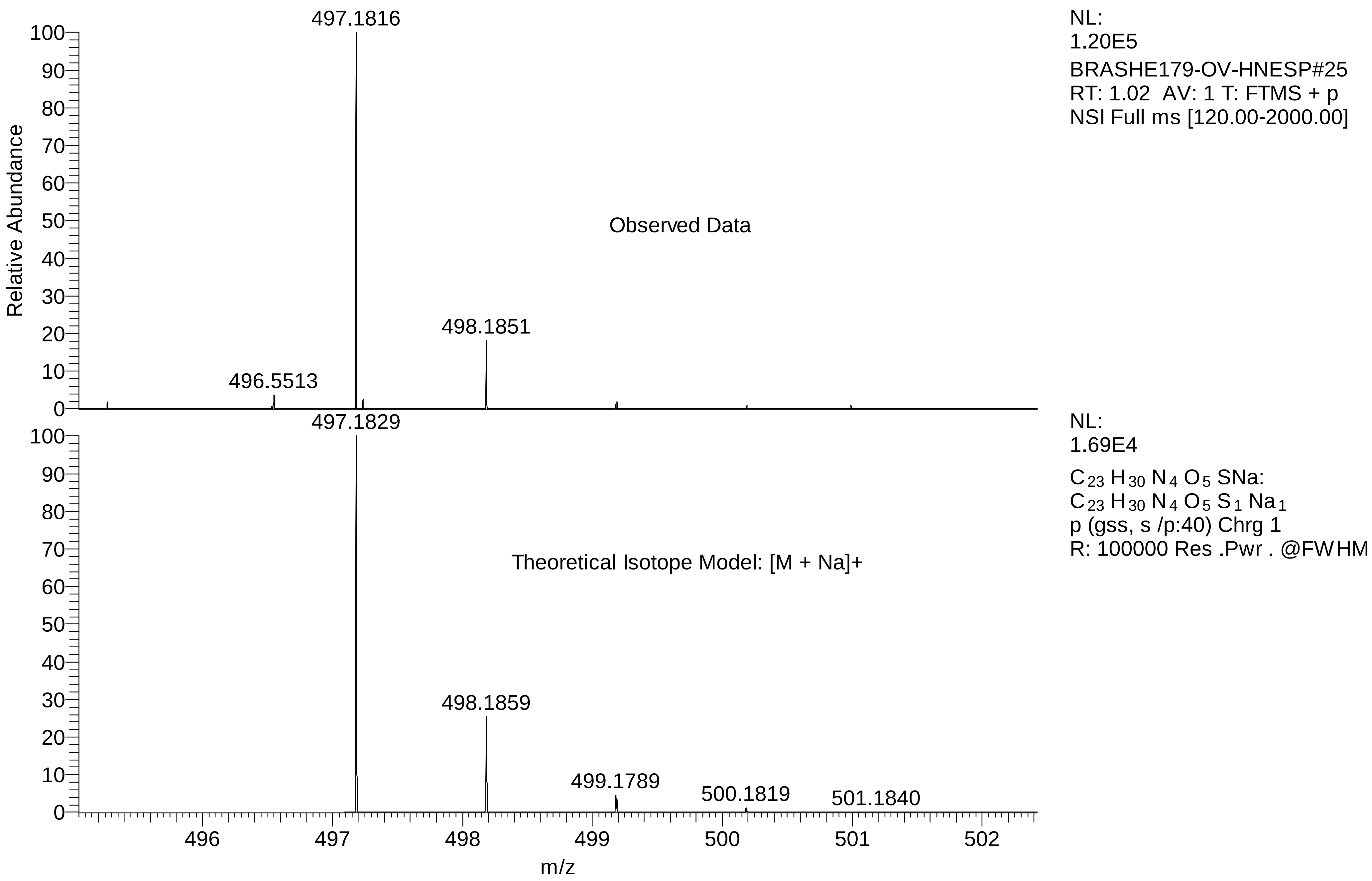

**Isotope:**	**Min. .. Max.**		
**14 N**	**0....10**		
**16 O**	**0....15**		
**12 C**	**0....30**		
**1 H**	**0....60**		
**23 Na**	**0....1**		
**32 S**	**0....2**		
**Tolerance Window:**	**+- 5.00 ppm**		
**Db/Ring Equiv:**	**-3..100**	**N-Rule:**	**Do not use**
**Fits:**	**100**	**Charge:**	**1**

**Mass**	**Theoretical Mass**	**Delta [ppm]**	**RDB**	**Composition**
497.1816	497.1816	0.1	5.5	$C_{22} H_{34} O_{9} Na_{1} S_{1}$
	497.1816	0.1	11.0	$C_{21} H_{28} O_{4} N_{7} Na_{1} S_{1}$
	497.1815	0.2	12.5	$C_{28} H_{33} O_{4} S_{2}$
	497.1818	-0.3	14.0	$C_{29} H_{32} O_{1} N_{1} Na_{1} S_{2}$
	497.1814	0.4	2.5	$C_{14} H_{30} O_{12} N_{6} Na_{1}$
	497.1813	0.6	9.5	$C_{20} H_{29} O_{7} N_{6} S_{1}$
	497.1819	-0.7	18.5	$C_{28} H_{25} O_{5} N_{4}$
	497.1820	-0.8	0.0	$C_{14} H_{35} O_{10} N_{5} S_{2}$
	497.1811	1.0	1.0	$C_{13} H_{31} O_{15} N_{5}$
	497.1822	-1.2	20.0	$C_{29} H_{24} O_{2} N_{5} Na_{1}$
	497.1823	-1.3	1.5	$C_{15} H_{34} O_{7} N_{6} Na_{1} S_{2}$
	497.1809	1.4	2.0	$C_{13} H_{32} O_{6} N_{9} Na_{1} S_{2}$
	497.1809	1.4	15.0	$C_{28} H_{28} O_{6} N_{1} Na_{1}$
	497.1809	1.5	20.5	$C_{27} H_{22} O_{1} N_{8} Na_{1}$
	497.1825	-1.7	6.0	$C_{14} H_{27} O_{11} N_{9}$
	497.1806	1.9	0.5	$C_{12} H_{33} O_{9} N_{8} S_{2}$
	497.1806	2.0	13.5	$C_{27} H_{29} O_{9}$
	497.1806	2.0	19.0	$C_{26} H_{23} O_{4} N_{7}$
	497.1826	-2.1	14.5	$C_{21} H_{25} O_{3} N_{10} S_{1}$
	497.1826	-2.1	9.0	$C_{22} H_{31} O_{8} N_{3} S_{1}$
	497.1827	-2.3	7.5	$C_{15} H_{26} O_{8} N_{10} Na_{1}$
	497.1827	-2.3	2.0	$C_{16} H_{32} O_{13} N_{3} Na_{1}$
	497.1804	2.4	14.5	$C_{27} H_{30} N_{4} Na_{1} S_{2}$
	497.1828	-2.4	17.5	$C_{29} H_{29} N_{4} S_{2}$
	497.1829	-2.6	10.5	$C_{23} H_{30} O_{5} N_{4} Na_{1} S_{1}$
	497.1802	2.8	6.0	$C_{20} H_{32} O_{8} N_{3} Na_{1} S_{1}$
	497.1802	2.8	11.5	$C_{19} H_{26} O_{3} N_{10} Na_{1} S_{1}$
	497.1801	2.9	13.0	$C_{26} H_{31} O_{3} N_{3} S_{2}$
	497.1800	3.1	3.0	$C_{12} H_{28} O_{11} N_{9} Na_{1}$
	497.1800	3.3	4.5	$C_{19} H_{33} O_{11} N_{2} S_{1}$
	497.1800	3.3	10.0	$C_{18} H_{27} O_{6} N_{9} S_{1}$

Mass	Theoretical Mass	Delta [ppm]	RDB	Composition
	497.1833	-3.4	23.5	$C_{29} H_{21} O_1 N_8$
	497.1833	-3.4	18.0	$C_{30} H_{27} O_6 N_1$
	497.1833	-3.5	5.0	$C_{15} H_{31} O_6 N_9 S_2$
	497.1833	-3.5	-0.5	$C_{16} H_{37} O_{11} N_2 S_2$
	497.1834	-3.7	-2.0	$C_9 H_{32} O_{11} N_9 Na_1 S_1$
	497.1798	3.7	1.5	$C_{11} H_{29} O_{14} N_8$
	497.1836	-4.0	6.5	$C_{16} H_{30} O_3 N_{10} Na_1 S_2$
	497.1836	-4.0	1.0	$C_{17} H_{36} O_8 N_3 Na_1 S_2$
	497.1796	4.1	-3.0	$C_{12} H_{36} O_{10} N_5 Na_1 S_2$
	497.1795	4.1	15.5	$C_{26} H_{26} O_5 N_4 Na_1$
	497.1838	-4.4	5.5	$C_{16} H_{29} O_{12} N_6$
	497.1793	4.7	14.0	$C_{25} H_{27} O_8 N_3$
	497.1793	4.7	19.5	$C_{24} H_{21} O_3 N_{10}$
	497.1840	-4.8	14.0	$C_{23} H_{27} O_4 N_7 S_1$
	497.1840	-4.8	8.5	$C_{24} H_{33} O_9 S_1$
	497.1841	-5.0	7.0	$C_{17} H_{28} O_9 N_7 Na_1$
	497.1841	-5.0	1.5	$C_{18} H_{34} O_{14} Na_1$

Mass	Theoretical Mass	Delta [ppm]	RDB	Composition

Mass	Theoretical Mass	Delta [ppm]	RDB	Composition

aet7-49 MW=516?
(DCM)/MeOH + NH4OAc

Compound 54

EPSRC National Centre Swansea
LTQ Orbitrap XL

adam throup
13/01/2012 14:44:24

BRASHE181-OV-HNESP #21-33 RT: 0.89-1.24 AV: 13 NL: 2.30E7
T: FTMS + p NSI Full ms [120.00-2000.00]

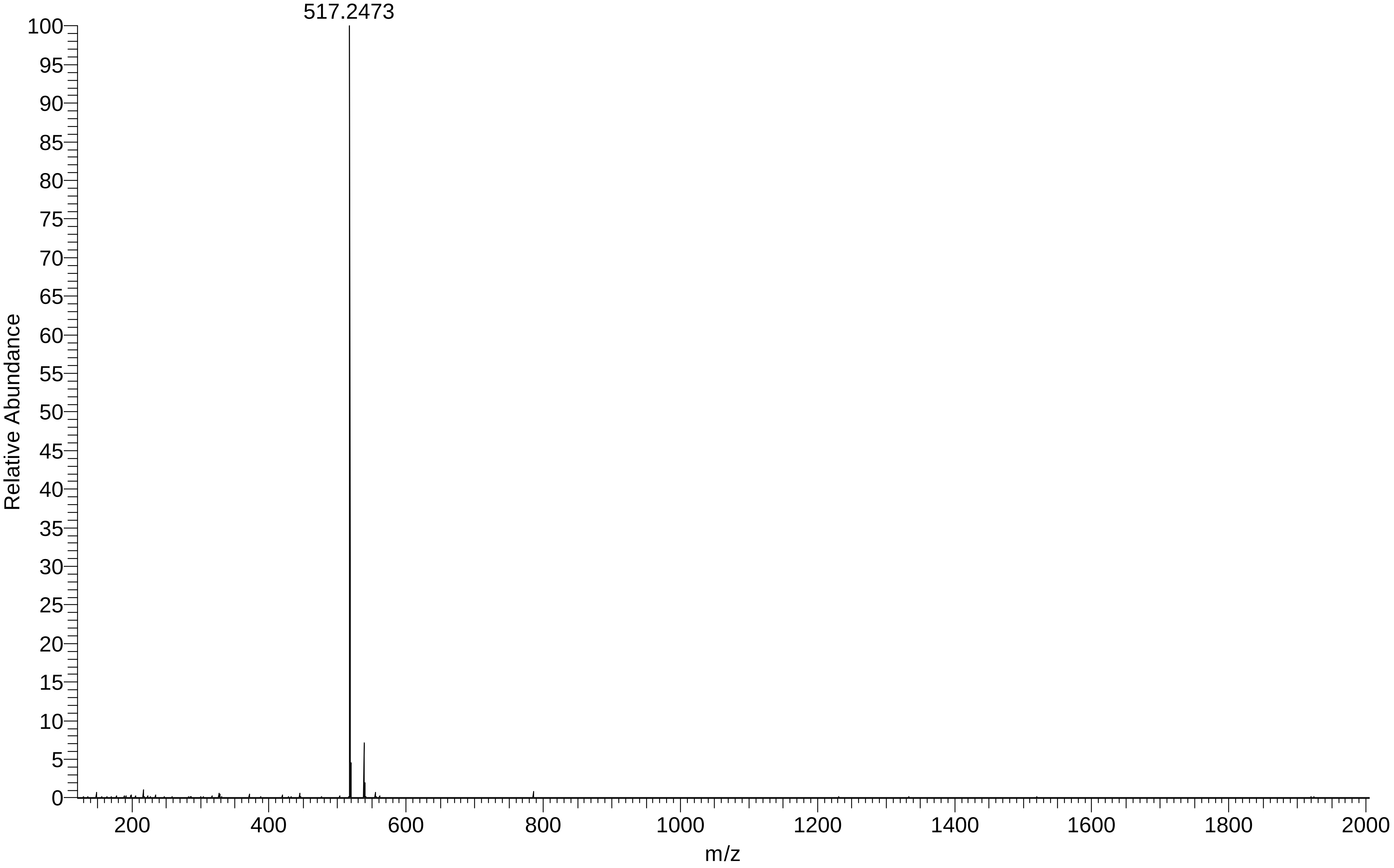

aet7-49 MW=516?
(DCM)/MeOH + NH4OAc

EPSRC National Centre Swansea
LTQ Orbitrap XL

adam throup
13/01/2012 14:44:24

BRASHE181-OV-HNESP #21-33 RT: 0.89-1.24 AV: 13 NL: 2.30E7
T: FTMS + p NSI Full ms [120.00-2000.00]

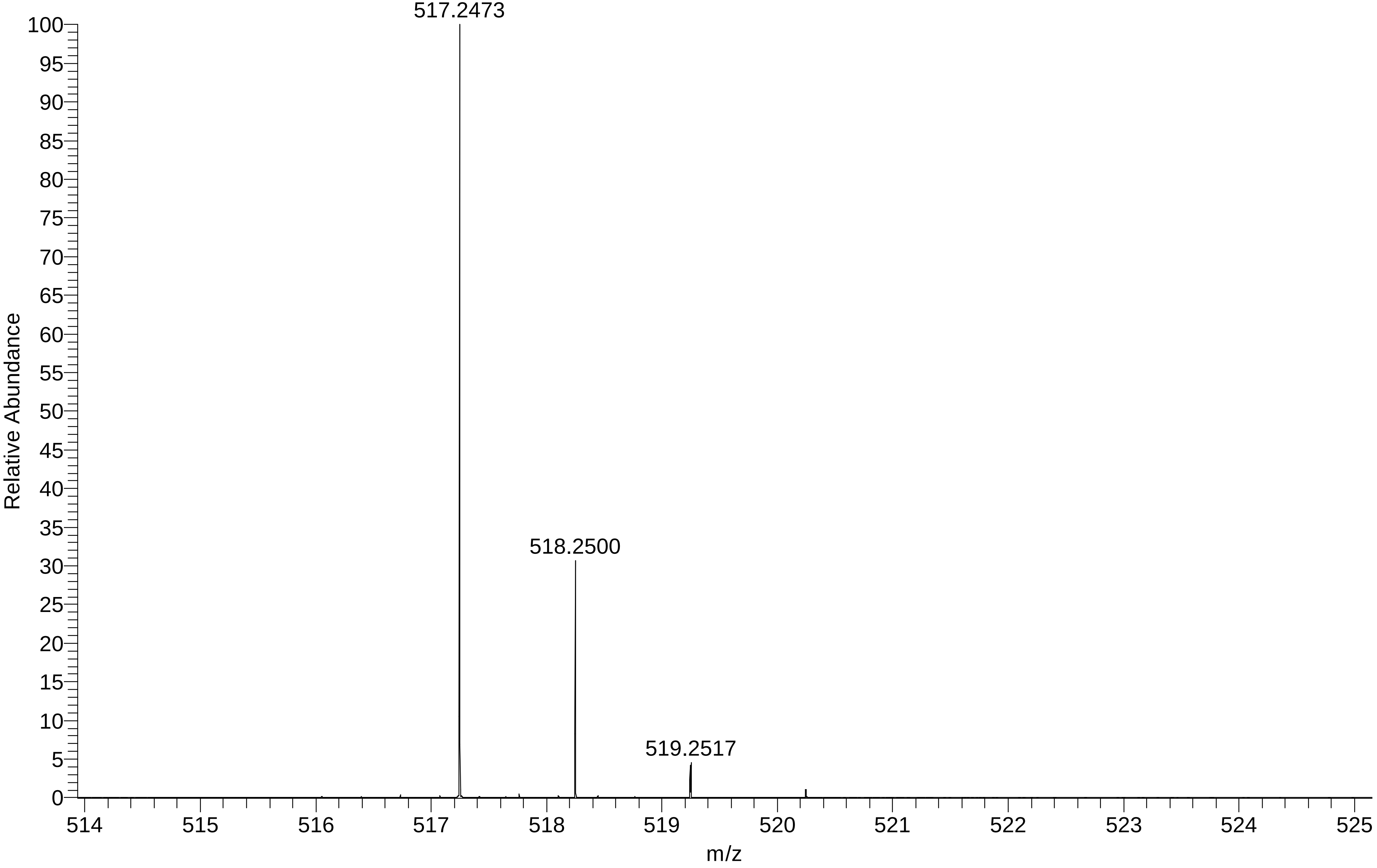

**Isotope:**	**Min. .. Max.**			
**14 N**	**0....10**			
**16 O**	**0....15**			
**12 C**	**0....40**			
**1 H**	**0....60**			
**23 Na**	**0....0**			
**32 S**	**0....2**			
**Tolerance Window:**	**+- 5.00 ppm**			
**Db/Ring Equiv:**	**-3..100**		**N-Rule:**	**Do not use**
**Fits:**	**100**		**Charge:**	**1**

Mass	Theoretical Mass	Delta [ppm]	RDB	Composition
517.2473	517.2473	0.1	1.5	$C_{18} H_{41} O_{7} N_{6} S_{2}$
	517.2472	0.1	20.0	$C_{32} H_{31} O_{2} N_{5}$
	517.2477	-0.8	7.5	$C_{18} H_{33} O_{8} N_{10}$
	517.2477	-0.8	2.0	$C_{19} H_{39} O_{13} N_{3}$
	517.2468	1.0	14.0	$C_{32} H_{39} O_{1} N_{1} S_{2}$
	517.2479	-1.2	10.5	$C_{26} H_{37} O_{5} N_{4} S_{1}$
	517.2466	1.4	5.5	$C_{25} H_{41} O_{9} S_{1}$
	517.2466	1.4	11.0	$C_{24} H_{35} O_{4} N_{7} S_{1}$
	517.2464	1.7	2.5	$C_{17} H_{37} O_{12} N_{6}$
	517.2484	-2.2	-2.0	$C_{12} H_{39} O_{11} N_{9} S_{1}$
	517.2486	-2.5	19.5	$C_{34} H_{33} O_{3} N_{2}$
	517.2486	-2.5	6.5	$C_{19} H_{37} O_{3} N_{10} S_{2}$
	517.2486	-2.5	1.0	$C_{20} H_{43} O_{8} N_{3} S_{2}$
	517.2459	2.7	2.0	$C_{16} H_{39} O_{6} N_{9} S_{2}$
	517.2459	2.7	15.0	$C_{31} H_{35} O_{6} N_{1}$
	517.2459	2.7	20.5	$C_{30} H_{29} O_{1} N_{8}$
	517.2491	-3.4	7.0	$C_{20} H_{35} O_{9} N_{7}$
	517.2491	-3.4	1.5	$C_{21} H_{41} O_{14}$
	517.2454	3.6	14.5	$C_{30} H_{37} N_{4} S_{2}$
	517.2493	-3.8	15.5	$C_{27} H_{33} O_{1} N_{8} S_{1}$
	517.2493	-3.8	10.0	$C_{28} H_{39} O_{6} N_{1} S_{1}$
	517.2452	4.0	6.0	$C_{23} H_{39} O_{8} N_{3} S_{1}$
	517.2452	4.0	11.5	$C_{22} H_{33} O_{3} N_{10} S_{1}$
	517.2451	4.3	3.0	$C_{15} H_{35} O_{11} N_{9}$
	517.2498	-4.8	-2.5	$C_{14} H_{41} O_{12} N_{6} S_{1}$

15-187
(MeOH)/MeOH+NH4OAc
C25H34N4O5S

Compound 55

EPSRC National Facility Swansea
LTQ Orbitrap XL

BRASHE
22/02/2017 15:50:57

BRASHE_O88TR_A #39-52 RT: 0.66-1.04 AV: 14 SM: 7G NL: 3.12E6
T: FTMS + p NSI Full ms [120.00-1935.00]

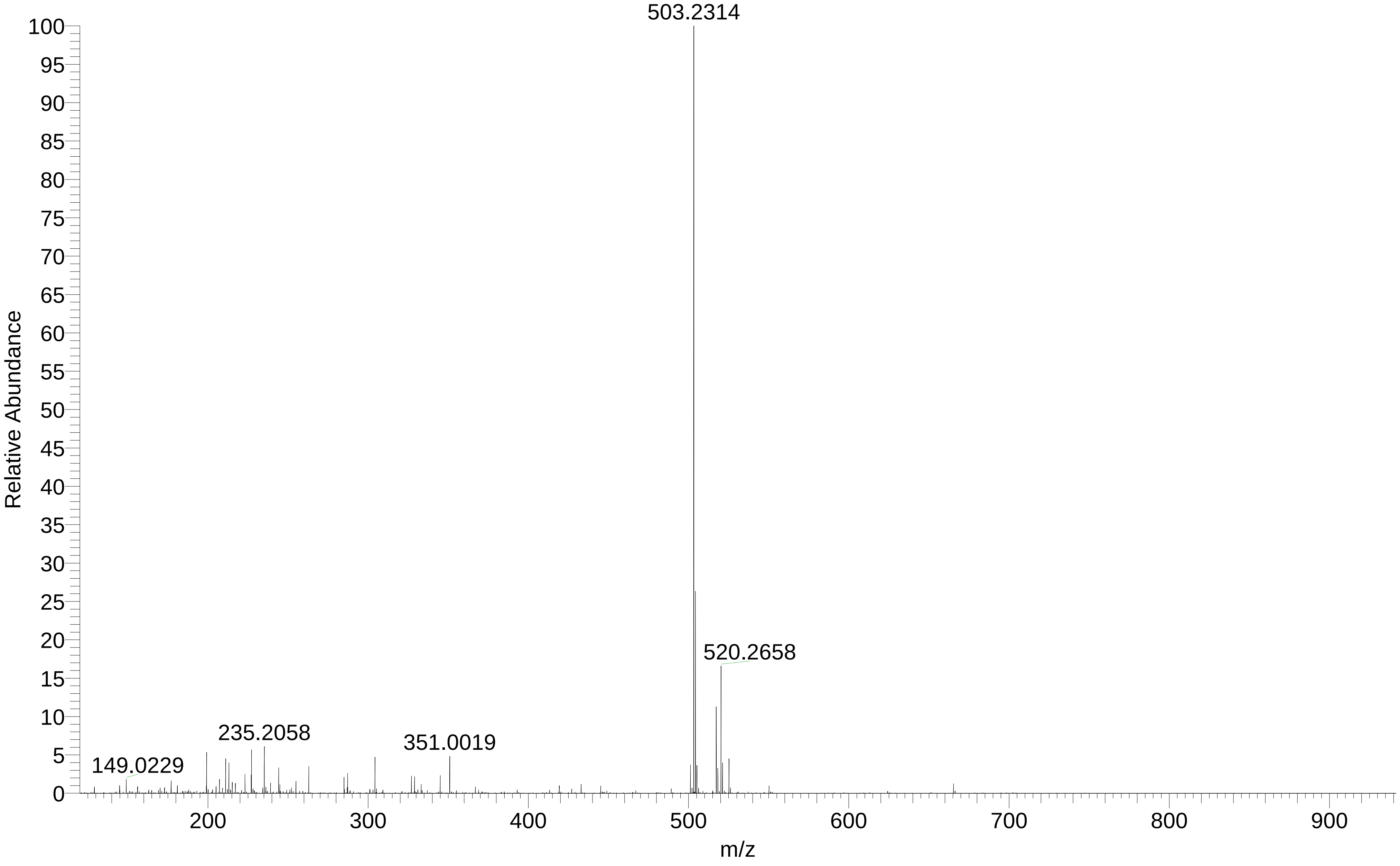

15-187
(MeOH)/MeOH+NH4OAc
C25H34N4O5S

EPSRC National Facility Swansea
LTQ Orbitrap XL

BRASHE
22/02/2017 15:50:57

BRASHE_O88TR_A #39-52 RT: 0.66-1.04 AV: 14 SM: 7G NL: 3.12E6
T: FTMS + p NSI Full ms [120.00-1935.00]

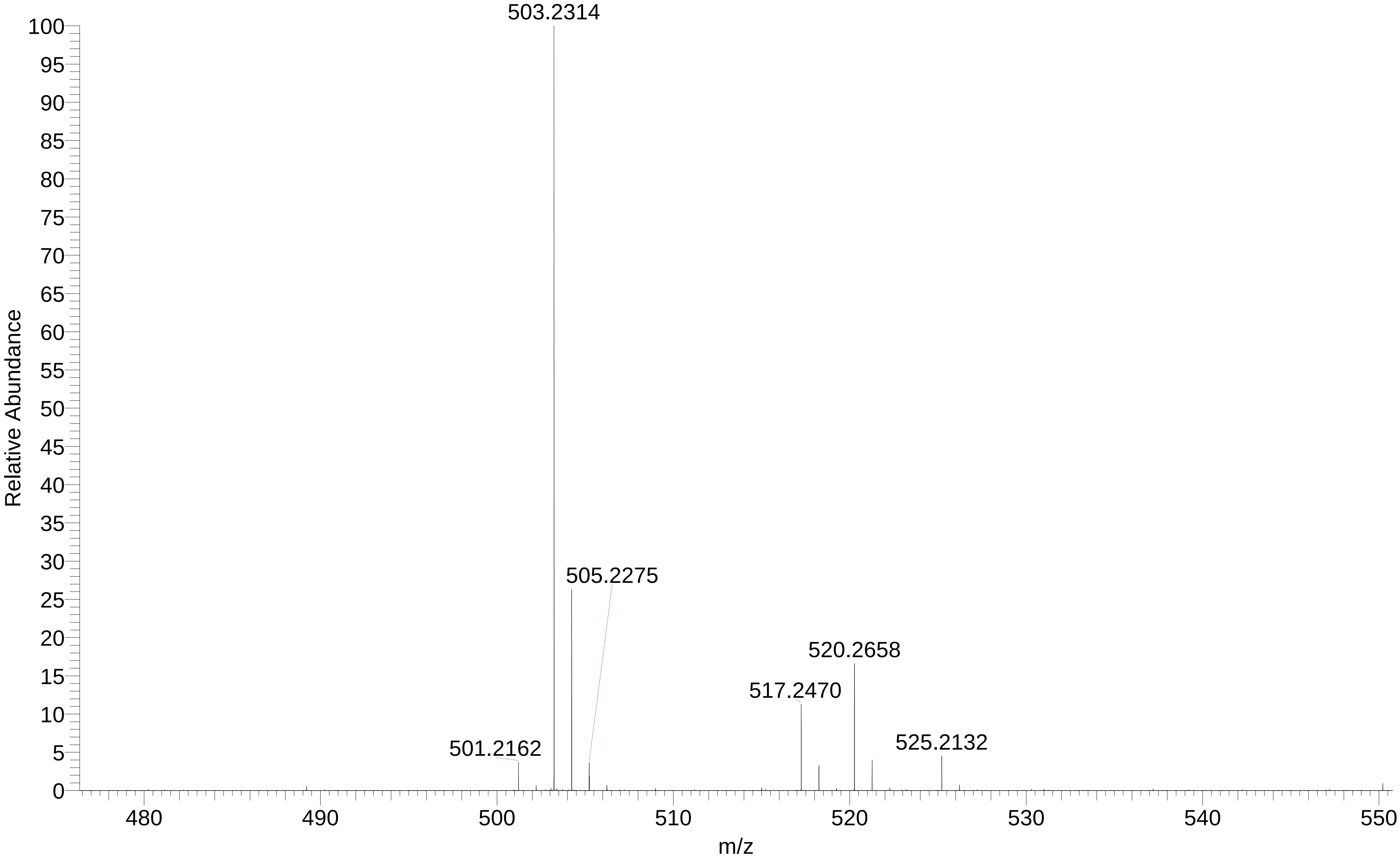

15-187
(MeOH)/MeOH+NH4OAc
C25H34N4O5S

EPSRC National Facility Swansea
LTQ Orbitrap XL

BRASHE
22/02/2017 15:50:57

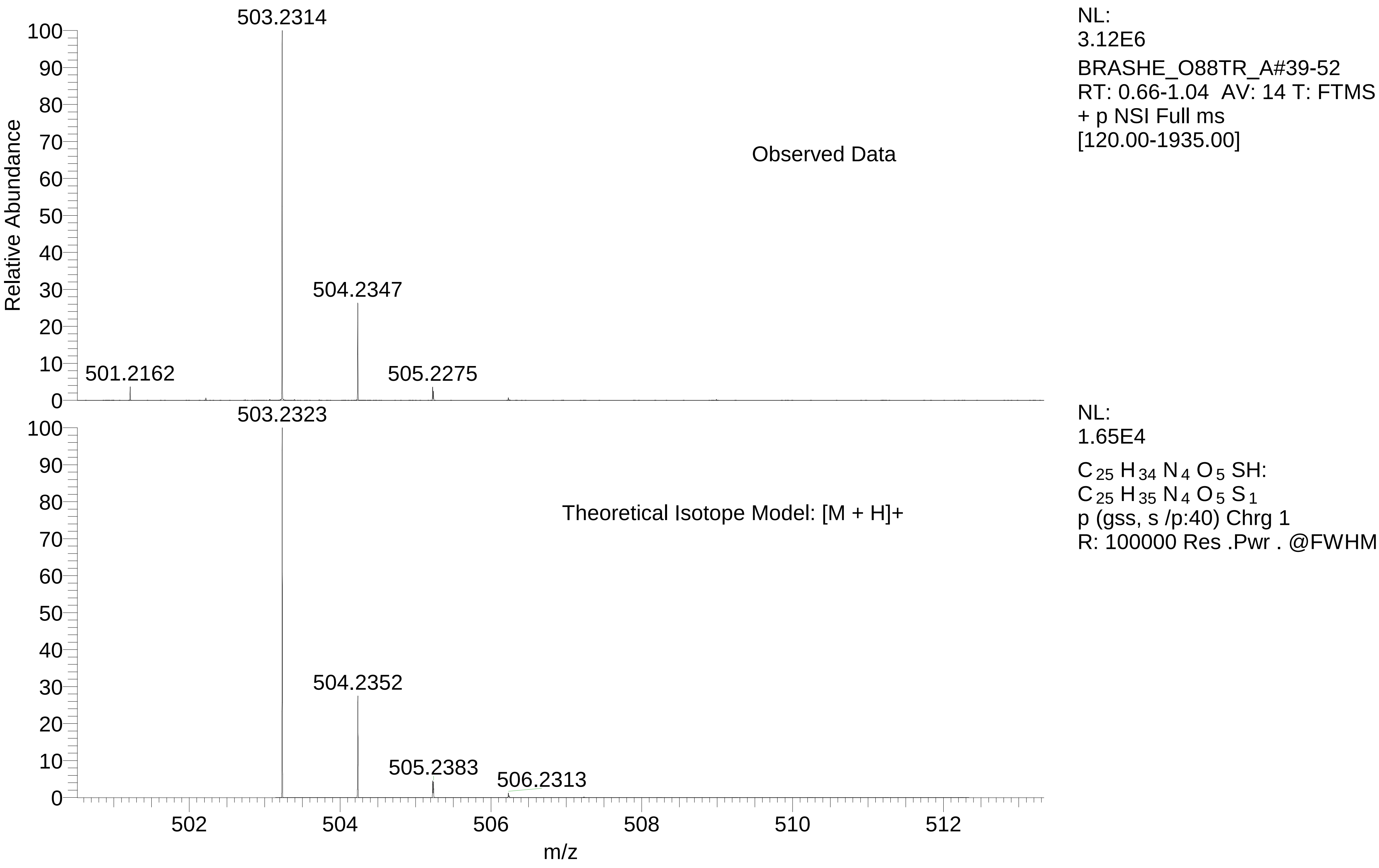

Isotope: **Min. .. Max.**
14 N **0....15**
16 O **0....15**
12 C **0....60**
1 H **0....70**
23 Na **0....0**
32 S **0....5**
Tolerance Window: **+- 5.00 ppm**
Db/Ring Equiv: **-3.. 100** **N-Rule:** **Do not use**
Fits: **150** **Charge:** **1**

**Mass**	**Theoretical Mass**	**Delta [ppm]**	**RDB**	**Composition**
503.2314	503.2314	-0.1	-1.5	$C_{9} H_{35} O_{10} N_{12} S_{1}$
	503.2316	-0.4	20.0	$C_{31} H_{29} O_{2} N_{5}$
	503.2316	-0.4	7.0	$C_{16} H_{33} O_{2} N_{13} S_{2}$
	503.2316	-0.4	1.5	$C_{17} H_{39} O_{7} N_{6} S_{2}$
	503.2311	0.5	1.0	$C_{16} H_{41} O_{1} N_{9} S_{4}$
	503.2311	0.6	14.0	$C_{31} H_{37} O_{1} N_{1} S_{2}$
	503.2318	-0.8	4.5	$C_{25} H_{43} O_{4} S_{3}$
	503.2310	0.9	-2.0	$C_{8} H_{37} O_{4} N_{15} S_{3}$
	503.2309	0.9	5.5	$C_{24} H_{39} O_{9} S_{1}$
	503.2309	0.9	11.0	$C_{23} H_{33} O_{4} N_{7} S_{1}$
	503.2307	1.3	2.5	$C_{16} H_{35} O_{12} N_{6}$
	503.2307	1.3	8.0	$C_{15} H_{29} O_{7} N_{13}$
	503.2321	-1.4	7.5	$C_{17} H_{31} O_{8} N_{10}$
	503.2321	-1.4	2.0	$C_{18} H_{37} O_{13} N_{3}$
	503.2323	-1.7	16.0	$C_{24} H_{29} N_{11} S_{1}$
	503.2323	-1.7	10.5	$C_{25} H_{35} O_{5} N_{4} S_{1}$
	503.2323	-1.8	-2.5	$C_{10} H_{39} O_{5} N_{12} S_{3}$
	503.2305	1.9	5.0	$C_{23} H_{41} O_{3} N_{3} S_{3}$
	503.2325	-2.2	0.5	$C_{18} H_{43} O_{2} N_{6} S_{4}$
	503.2303	2.2	2.0	$C_{15} H_{37} O_{6} N_{9} S_{2}$
	503.2302	2.3	15.0	$C_{30} H_{33} O_{6} N_{1}$
	503.2302	2.3	20.5	$C_{29} H_{27} O_{1} N_{8}$
	503.2301	2.6	-1.0	$C_{7} H_{33} O_{9} N_{15} S_{1}$
	503.2328	-2.7	-2.0	$C_{11} H_{37} O_{11} N_{9} S_{1}$
	503.2329	-3.0	19.5	$C_{33} H_{31} O_{3} N_{2}$
	503.2330	-3.1	6.5	$C_{18} H_{35} O_{3} N_{10} S_{2}$
	503.2330	-3.1	1.0	$C_{19} H_{41} O_{8} N_{3} S_{2}$
	503.2298	3.2	1.5	$C_{14} H_{39} N_{12} S_{4}$
	503.2298	3.2	14.5	$C_{29} H_{35} N_{4} S_{2}$
	503.2331	-3.4	9.5	$C_{26} H_{39} N_{4} S_{3}$
	503.2296	3.6	6.0	$C_{22} H_{37} O_{8} N_{3} S_{1}$

Mass	Theoretical Mass	Delta [ppm]	RDB	Composition
	503.2296	3.6	11.5	$C_{21} H_{31} O_3 N_{10} S_1$
	503.2294	4.0	3.0	$C_{14} H_{33} O_{11} N_9$
	503.2334	-4.0	12.5	$C_{18} H_{27} O_4 N_{14}$
	503.2334	-4.0	7.0	$C_{19} H_{33} O_9 N_7$
	503.2334	-4.0	1.5	$C_{20} H_{39} O_{14}$
	503.2336	-4.4	15.5	$C_{26} H_{31} O_1 N_8 S_1$
	503.2336	-4.4	10.0	$C_{27} H_{37} O_6 N_1 S_1$
	503.2336	-4.5	-3.0	$C_{12} H_{41} O_6 N_9 S_3$
	503.2291	4.5	5.5	$C_{21} H_{39} O_2 N_6 S_3$
	503.2338	-4.8	0.0	$C_{20} H_{45} O_3 N_3 S_4$
	503.2289	4.9	-3.0	$C_{14} H_{41} O_{10} N_5 S_2$
	503.2289	4.9	2.5	$C_{13} H_{35} O_5 N_{12} S_2$
	503.2289	5.0	15.5	$C_{28} H_{31} O_5 N_4$
	503.2289	5.0	21.0	$C_{27} H_{25} N_{11}$
