# Supplementary material for: Sidechain structure–activity relationships of cyclobutane-based small molecule αvβ3 antagonists
Source: RSC Med Chem. 2024 Sep 13;15(10):3616–24. doi: 10.1039/d4md00306c (PMC11393731; doi:10.1039/d4md00306c)
Supplement: MD-015-D4MD00306C-s003 [file MD-015-D4MD00306C-s003.pdf]

Supplementary Information (SI) for RSC Medicinal Chemistry.
This journal is © The Royal Society of Chemistry 2024

**Table S1** Cytotoxicity of compounds on the cell lines used in the functional assays (measured by MTT assay). Cells (2000 cells/well for Sk-Mel-2; 1000 cells/well for U87MG) were incubated for 96 hours at 37 °C in a total volume of 200 µL/well (180 µL medium + 20 µL test compound solution). After 96 hours, the medium was replaced by fresh medium containing 0.5 mg/ml (end conc.) MTT per well, incubated for 4 h then the plate processed and read as described in Sutherland et al.[1]

| Compound | $IC_{50}$/µM Sk-Mel-2 | U87MG $IC_{50}$/µM |
|---|---|---|
| **17** ICT9097 | > 50 | 70.1 ± 18.8 |
| **18** ICT 9096 | - | 59.2 ± 16.9 |
| **19** ICT 9100 | > 50 | - |
| **20**ICT 9093 | > 50 | 37.9 ± 9.1 |
| **21** ICT 9091 | > 50 | 30.9 ± 8.1 |
| **22** ICT 9092 | > 50 | 46.4 ± 3.5 |
| **23** ICT 9101 | > 50 | - |
| **24**ICT 9094 | 31.9 ± 2.0 | 46.7 ± 7.6 |
| **28** ICT 9082 | > 100 | 32.5 ± 14.1 |
| **29** ICT 9084 | > 50 | 36.2 ± 12.4 |
| **30** ICT 9081 | > 10 | 160.8 ± 61.0 |
| **31** ICT 9083 | > 100 | - |
| **32** ICT 9085 | 80.0 ± 19.6 | 40.9 ± 11.5 |
| **33** ICT 9087 | 40.0 ± 2.0 | 35.5 ± 2.0 |
| **34** ICT 9088 | 97.1 ± 3.2 | 51.3 ± 1.9 |
| **35** ICT 9089 | 15.2 ± 5.5 | - |
| **37**ICT 9098 | > 50 | - |
| **38** ICT 9102 | > 50 | - |
| **39** ICT 9099 | 35.2 ± 6.5 | - |
| **40** ICT 9103 | > 50 | - |
| **53** ICT 9072 | 33.5 ± 8.4 | 36.9 ± 8.0 |
| **54** ICT 9073 | 33.0 ± 10.5 | 18.9 ± 2.6 |
| **1** ICT9055 | 9.6 ± 5.7 | 36.9 ± 11.7 |
| cRGDfV | 42.5 ± 1.8 | 69.2 ± 25.5 |

1. M. Sutherland, A. Gordon, F. O. Al-Shammari, A. Throup, A. Cilia La Corte, H. Philippou, S. D. Shnyder, L. H. Patterson and H. M. Sheldrake, *Cancers*, 2023, **15**, 4023.

**Metabolic stability of ICT9055 (1) and its free acid metabolite ICT9064**

A

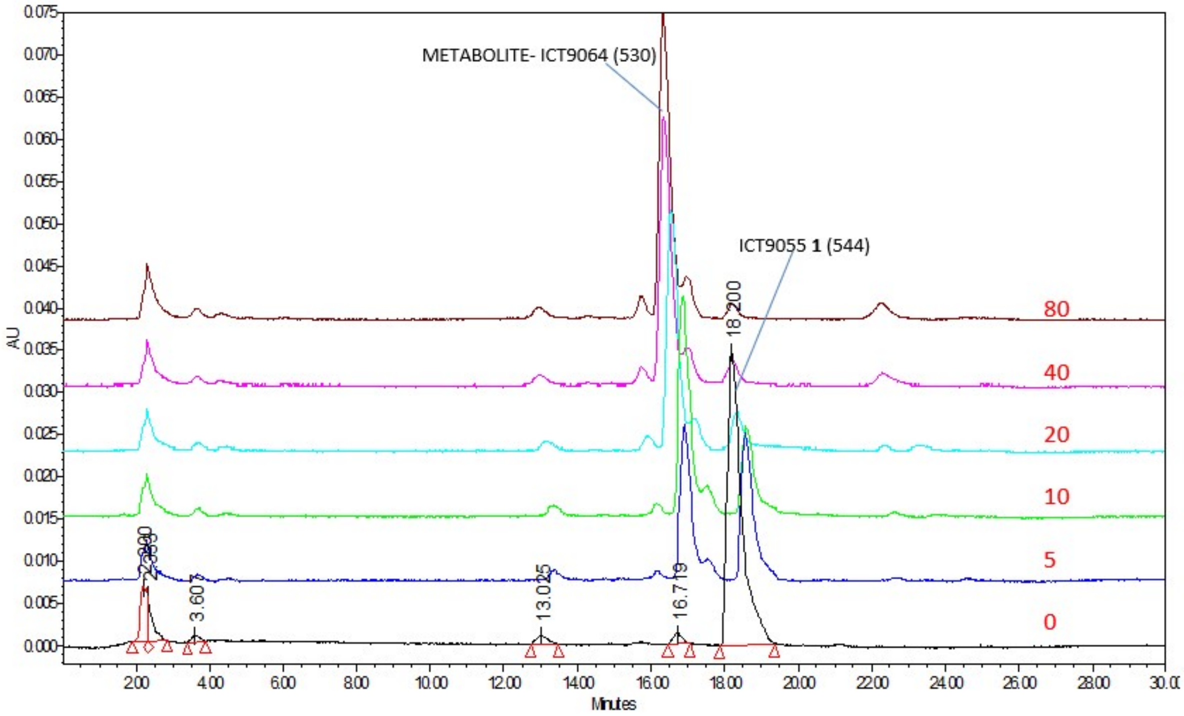


B

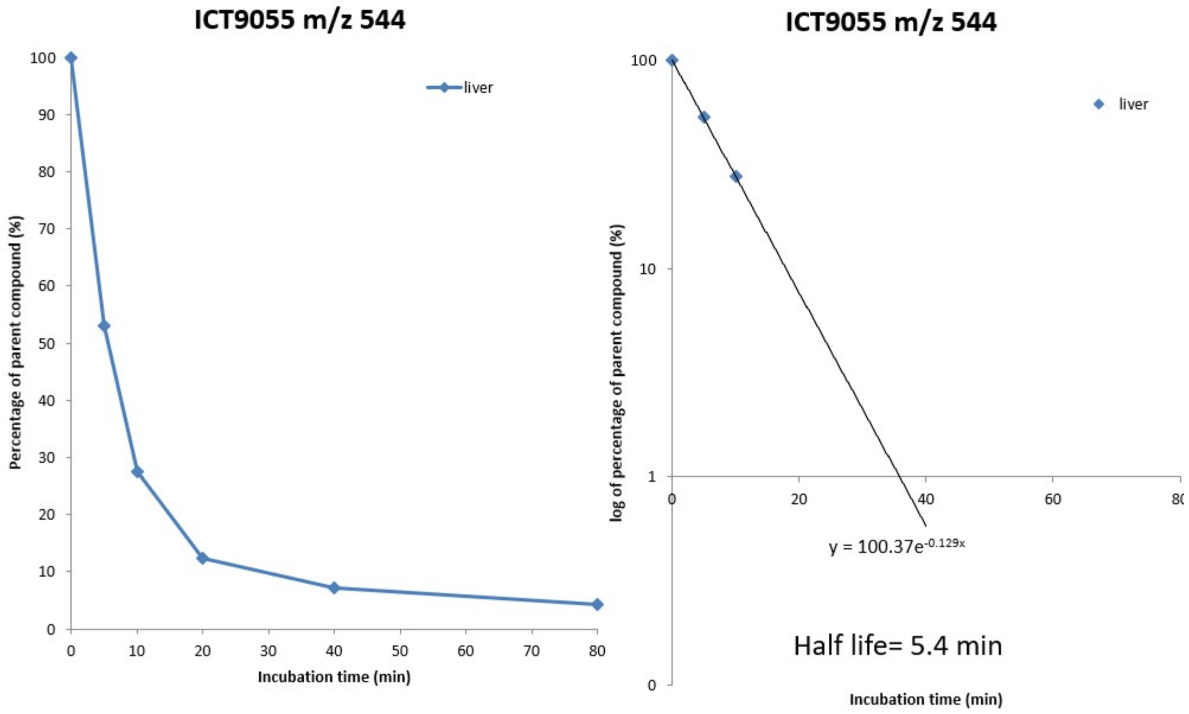


C

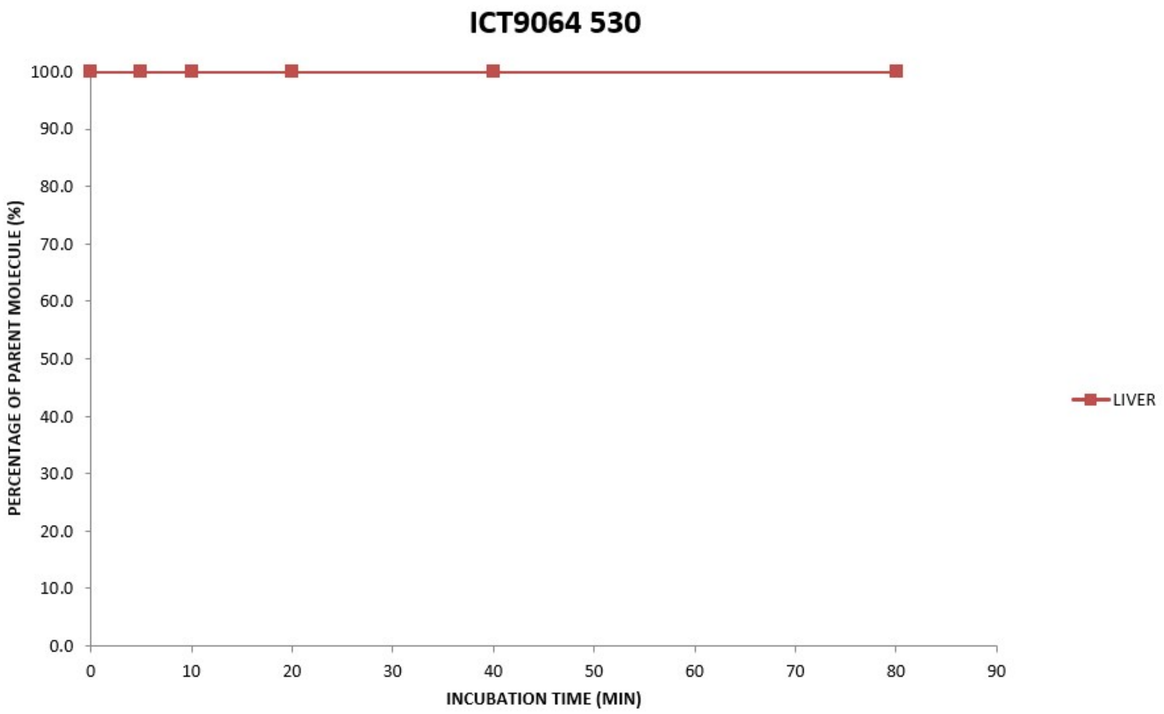


Figure S2: A. Timecourse of conversion of methyl ester **1** (ICT9055) to carboxylic acid (ICT9064) in mouse liver homogenate. B. Determination of half-life of **1** (ICT9055) **i**n mouse liver homogenate. C. The metabolite, carboxylic acid (ICT9064) is highly stable in liver, with a half life of >80 minutes.

**A**

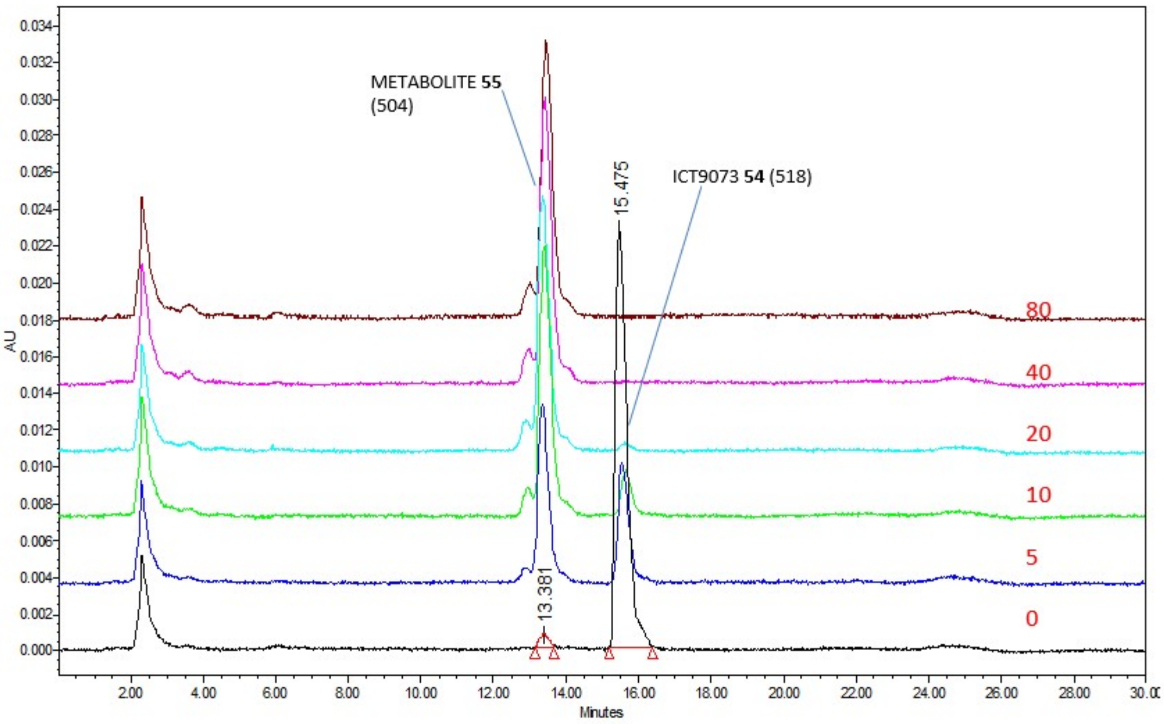


**B**

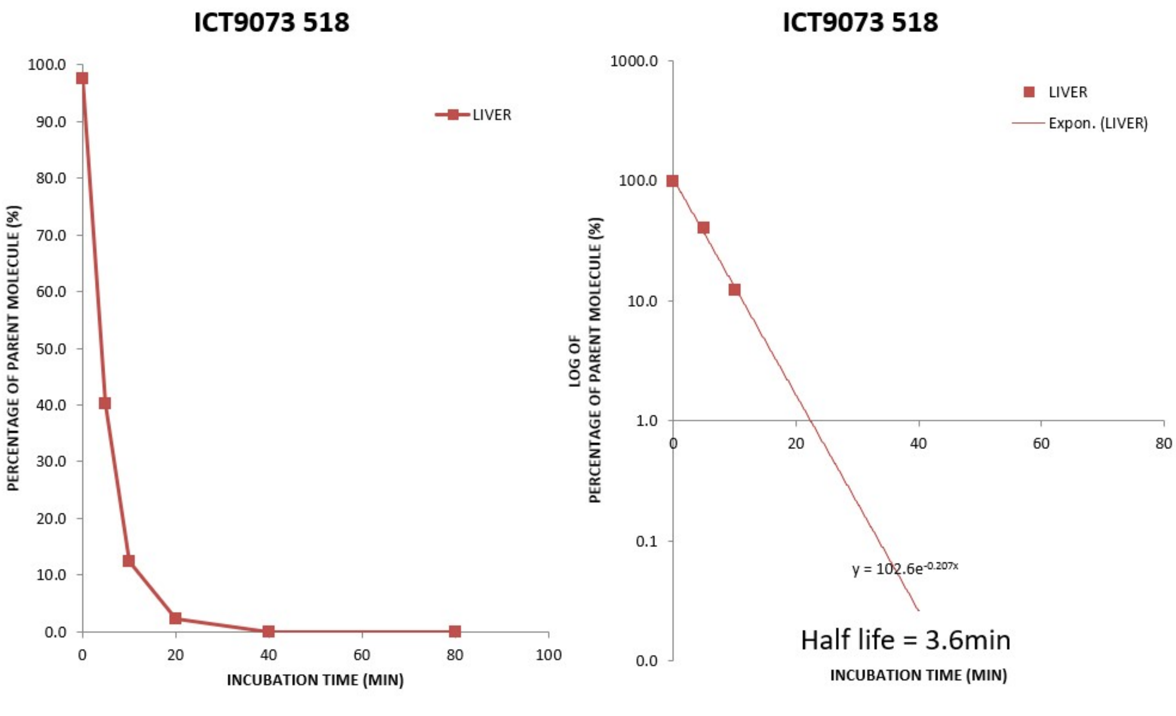


Figure S3. A. Timecourse of conversion of methyl ester **54** (ICT9073) to carboxylic acid **55** in mouse liver homogenate. The metabolite appears to be stable in liver. B. Determination of half-life of **54** in mouse liver homogenate.
